# Dysregulation of Arachidonic Acid Metabolism Drives Inflammatory Lipid Production in Localized Provoked Vulvodynia

**DOI:** 10.3390/nu17132233

**Published:** 2025-07-05

**Authors:** Sarah A. Fischer, Oluwademilade Oladele, Zahra Mahamed, Emanuelle Chrysilla, Anna Baumer, Tamari Bekauri, Krishna Rao Maddipati, Tanzy Love, Mitchell Linder, Megan Falsetta

**Affiliations:** 1 Department of Obstetrics and Gynecology, University of Rochester, Rochester, NY 14642, USA; sarahfischer787@gmail.com (S.A.F.); oluwademilade_oladele@urmc.rochester.edu (O.O.); anna.m.baumer@dmu.edu (A.B.); redw2020@gmail.com (T.B.);; 2 Department of Pharmacology and Physiology, University of Rochester, Rochester, NY 14642, USA; zahra_mahamed@urmc.rochester.edu (Z.M.); emanuelle_chrysilla@urmc.rochester.edu (E.C.); 3 Lipidomics Core Facility and Bioactive Lipids Research Program, Wayne State University, Detroit, MI 48202, USA; aj2642@wayne.edu; 4 Department of Biostatistics and Computational Biology, University of Rochester, Rochester, NY 14642, USA; tanzy_love@urmc.rochester.edu

**Keywords:** localized provoked vulvodynia, arachidonic acid, lipid metabolism, specialized pro-resolving mediators, inflammation, COX-1, COX-2, 12-LOX, chronic pain

## Abstract

**Background/Objectives**: Localized provoked vulvodynia (LPV) is characterized by chronic vulvar pain upon light touch to the vestibule, a specialized ring of tissue immediately surrounding the vaginal opening. LPV affects about 14 million people in the US, yet the etiopathology of the disease is unknown. In LPV, the vestibule expresses elevated levels of the pro-nociceptive pro-inflammatory mediators prostaglandin E_2_ (PGE2) and interleukin-6 (IL-6), which corresponds to lower pain thresholds. Previous studies have shown reduced amounts of arachidonic acid (AA)-derived pro-resolving lipid mediators in tissue biopsies from LPV patients that might impede the resolution of inflammation. AA is obtained from dietary linoleic acid, pointing to a defect in the metabolism of dietary polyunsaturated fatty acids in LPV. We aimed to further explore the involvement of AA metabolism in LPV, which appears dysregulated in the vestibule of LPV patients and culminates in chronic inflammation and chronic pain. **Methods**: Vestibular and vulvar tissue biopsies obtained from LPV and non-LPV patients were used to generate fibroblast strains and assessed for COX/LOX expression using qRT-PCR. Fibroblast strains were treated with inflammatory stimuli, and then COX-1 and COX-2 expression was assessed using Western blot analysis. Pro-inflammatory mediator production was assessed using enzyme-linked immunosorbent assays (ELISAs). *ALOX5* and *ALOX12* expression was assessed using qRT-PCR. Finally, lipidomic analysis was carried out to screen for 143 lipid metabolites following inflammatory challenge. **Results**: Tissue and fibroblasts from LPV patients exhibited altered expression of COX/LOX enzymes and production of AA-derived lipid mediators compared to non-LPV patients. **Conclusions**: Lipid profiles of tissue and vestibular fibroblasts from LPV patients differed from non-LPV patients, and this difference was attributed to differential COX/LOX expression and activity, which metabolizes AA derived from dietary linoleic acid. This dysregulation fosters chronic inflammation and reduced resolution capacity in LPV patients, causing chronic pain. While further work is needed, these findings suggest that dietary modifications could impact the LPV mechanism.

## 1. Introduction

Localized provoked vulvodynia (LPV) is characterized by pain upon light touch to the vulvar vestibule (specialized ring of tissue surrounding the vaginal opening), lasting for three or more months, and it is estimated to affect between 8 and 16% of women in the United States [1,2,3,4]. Patients with LPV reported having higher occurrences of depression, psychological distress, reduced sexual satisfaction, and lower self-esteem [5,6]. Currently, LPV is diagnosed by exclusion, requiring that patients have no current cervicovaginal infections or dermatological and gynecological conditions [7]. A study by Harlow et al. (2001) showed 30% of patients with LPV had to seek treatment from three or more physicians, and only 60% received a diagnosis, thus leading predictably to high healthcare costs as well as indirect costs related to delayed diagnosis [8]. A study by Xie et al. (2012) estimates that in six months, the cost of care averaged USD 8,862 per patient, including direct healthcare costs and non-direct costs [9].

Despite the financial and mental toll on patients, there is not yet a complete understanding of the etiopathogenesis of LPV. However, current research points to inflammation. Over 70% of LPV patients report a history of vulvovaginal candidiasis (yeast infections), and vaginal swabs from LPV patients show elevated expression of the pro-inflammatory mediators IL-3, 1L-12, TNF-β, IL-18, 1L-1α, and IL-15 [10,11]. In agreement, a study by Tommola et al. (2015) discovered more B lymphocytes and mature IgA plasma cells in the vestibular tissue of LPV patients than in controls and identified “vestibule-associated lymphoid tissue” in LPV patients, indicating a response to inflammation or infection [12]. Further, evidence of B-cell infiltration and possible concomitant epithelial nerve growth in the vulvar vestibule points towards inflammation and an inability to resolve it as the cause of pain in LPV patients [13].

Dietary polyunsaturated fats (PUFAs), such as arachidonic acid (AA; derived from dietary linoleic acid), docosahexaenoic acid (DHA), and eicosapentaenoic acid (EPA), modulate the immune response and its resolution either directly or as precursors to bioactive anti-/pro-inflammatory lipid mediators [14,15]. In the initial inflammatory response, the metabolites of dietary AA, including hydroxyeicosatetraenoic acids (HETEs), leukotrienes, and prostaglandins increase while neutrophils, lymphocytes, and monocytes migrate to the site of infection or damage [16,17]. In vulvodynia, dysregulation of AA metabolism appears linked to the inability to ablate inflammatory signaling and trigger resolution, thus leading to chronic inflammation. Our group has conducted lipidomic analysis on biopsies from LPV patients and healthy controls and found that three AA-derived lipids, 12-HETE, 8(9)-eicosatrienoic acid (EET), and 14(15)-EET, were significantly reduced in the painful vestibule of LPV patients compared to non-painful areas in cases and controls [18,19]. In conjunction with these findings, anecdotal evidence has suggested dietary modification can help alleviate vulvar pain, specifically adherence to a low-oxalate diet, which is also low in the AA precursor linoleic acid [20]. While dietary modification has not been extensively explored in this population, understanding PUFA metabolism in vulvodynia could lead to novel dietary and therapeutic interventions.

Dietary PUFAs are vital during the resolution phase of inflammation, as specialized pro-resolving mediators (SPMs) metabolized from AA, EPA, and DHA act as agonists to stop neutrophil influx and activate nonphlogistic responses by macrophages [21,22,23,24]. Dysregulation in the synthesis of SPMs has been implicated in inflammatory diseases like severe asthma, cystic fibrosis, and arthritis [16,25,26]. In a paper published by our group in 2021, we discovered that SPMs were able to reduce the amount of pro-inflammatory mediators produced by vulvar and vestibular fibroblasts from LPV patients [27]. Taken together, these findings indicate that there might be dysregulation in dietary lipid metabolism causal to enhanced inflammation and reduced resolution capacity in LPV patients.

Cyclooxygenases (COXs—also known as prostaglandin H synthases) are the main enzymes involved in the synthesis of prostaglandins from AA [28]. There are two isoforms of cyclooxygenase, COX-1, which is expressed constitutively and thought to maintain homeostatic levels of prostaglandins for physiological functions, and COX-2, which is inducible [29]. Lipoxygenases (LOXs) metabolize AA into leukotrienes and lipoxins and are generally categorized as 5-, 8-, 12-, and 15-lipoxygenases [30]. Therapies targeting the COX/LOX enzyme systems in AA metabolism are already available for use [16]. COXIBs, a class of NSAIDs, preferentially inhibit COX-2 activity to treat inflammation [31]. Leukotriene (derived from the 5-LOX pathway) antagonists have been developed for the treatment of asthma and seasonal allergies [16]. Understanding the apparent alteration in the lipid metabolism might be instrumental in understanding the pathology of LPV and developing clinical therapies to alleviate pain. We found that fibroblasts cultured from LPV patients had altered AA metabolism compared to controls, resulting in an exaggerated immune response to inflammatory stimuli and a diminished ability to produce appropriate amounts of SPMs to resolve inflammation.

## 2. Materials and Methods

### 2.1. Patient/Sample Selection

LPV-afflicted cases and age-/race-matched pain-free controls were recruited for this study from the Division of General Obstetrics and Gynecology clinical practice at the University of Rochester. All subjects provided informed consent, and the research was approved by the University of Rochester Institutional Review Board (RSRB #42136) as previously described [32]. In brief, LPV patients met Friederich’s criteria, denied the use of corticosteroids and non-steroidal anti-inflammatory medications, had no chronic inflammatory illnesses other than LPV, and were negative for any current vulvovaginal infections. Controls met the same criteria but were negative for Friedrich’s criteria and had no history of vulvar disease (Figure S1). A total of 23 LPV cases and 17 controls (Table S1) were recruited for this study, from which primary fibroblast strains were created and used for each experiment as described herein. Furthermore, 6 mm biopsies and vaginal lavages were collected immediately prior to gynecologic surgery under sterile conditions while the patient was anesthetized. One biopsy was collected from the vulvar vestibule (painful site in cases) and external vulva (non-painful site) from both LPV cases and healthy controls as previously described [32]. Vaginal lavages were used to quantify the ratio of superficial epithelial cells to parabasal epithelial cells for the maturity index (MI), and all patients were determined to have a higher MI, indicating higher concentrations of circulating estrogens indicative of normally cycling (non-menopausal) women.

### 2.2. RNA Extractions and cDNA Synthesis

RNA was extracted from vulvar tissue and fibroblast cells for qPCR analyses. Tissue samples were processed into 3 pieces, and 1 piece was stabilized in QIAzol Lysis Reagent (Qiagen, Carlsbad, CA, USA) for RNA extraction. Fibroblast cells were first grown to confluence in 6-well plates in MEM supplemented with 10% FBS, GlutaMAX, gentamicin, and antibiotic/antimycotic solution (Thermo Fisher, Waltham, MA, USA). Media was then replaced with QIAzol Lysis Reagent, and RNA was extracted from each well individually to generate triplicate RNA samples for each strain. Total mRNA was extracted using the Qiagen RNeasy kit following the manufacturer’s instructions, including the optional QIAshredder step for tissue homogenization and the on-column DNase I digest step (Qiagen). RNA was quantified by spectrophotometric analysis using a NanoDrop One spectrophotometer (Thermo Fisher, Waltham, MA, USA), and the quality of RNA was checked via gel electrophoresis. RNA that had a 260/280 ratio of around 2.0 and distinct 28S and 18S rRNA bands on the agarose gel was considered pure and was used for cDNA synthesis. RNA was reverse transcribed into cDNA using the iScript cDNA synthesis kit (BioRad, Hercules, CA, USA). Each reaction contained 300 ng of RNA template diluted in 15 μL of RNase-free molecular grade water (Qiagen), 1 μL of iScript reverse transcriptase, and 4 μL of 5x iScript reaction mix buffer. Negative reverse transcriptase controls that contained no iScript reverse transcriptase were also prepared to confirm the absence of DNA contamination. After amplification, cDNA samples were diluted 5-fold in RNase-free molecular grade water (Qiagen) and used as templates for qRT-PCR reactions (5 μL/reaction).

### 2.3. Quantitative Real-Time PCR

To assess mRNA expression of LOX and COX enzymes in human tissue and fibroblasts, qPCR was performed according to protocols provided by Bio-Rad. cDNA curves derived from tissue or fibroblast RNA were constructed using concentrations of 500 ng, 100 ng, 20 ng, 4 ng, 0.8 ng, and 0.16 ng of cDNA diluted in 5 μL of RNase-free molecular grade water (Qiagen). cDNA curves were analyzed alongside experimental samples to provide quantitative analyses of mRNA levels. Reaction conditions and primer concentrations were optimized prior to performing assays by identifying the combination of conditions that resulted in reaction efficiencies closest to 100% without non-specific amplification. Primers were designed using NCBI nucleotide databases and the IDT OligoAnalyzer Tool and are listed in Supplemental Table S2 [33,34]. Master mixes for each primer set were made by combining SsoAdvanced Universal SYBR Green Supermix (BioRad), forward and reverse primers, and RNase-free molecular grade water (Qiagen). Depending on conditions identified during optimization steps, primers were either diluted to a final concentration of either 150 nM, 200 nM, 250 nM, or 300 nM. Furthermore, 7 μL of each master mix was added to each 5 μL sample of cDNA, for a final volume of 12 μL/reaction. Reactions were amplified using a CFX Connect Real-Time PCR Detection System (BioRad). Denaturation temperatures were held consistent at 95 °C, and annealing temperatures of either 55 °C or 58 °C were used. Data were analyzed using the CFX Maestro Software version 2.3 (BioRad). Relative abundance of mRNA was quantified using comparisons to CT values of standard curves and was normalized to expression of housing keeping gene 18S.

### 2.4. Western Blot

To determine protein expression of COX-1 and COX-2 in fibroblast cells, cells were grown to confluence in 6-well plates containing Minimum Essential Medium (MEM) supplemented with 10% FBS, GlutaMAX, gentamicin, and antibiotic/antimycotic solution (Gibco/Invitrogen/Thermo Fisher Scientific, Grand Island, NY, USA), and were starved 24 h prior to sample collection. Cells were washed with 1X PBS and collected in 50 mM Tris with 2% SDS cell lysis buffer. Samples were homogenized by mechanical force using hypodermic needles with syringes or by sonication. Protein concentrations were quantified using a DC Protein Assay (Bio-Rad). Samples were diluted to the lowest concentration of protein identified, mixed with Laemmli buffer (Bio-Rad), and loaded into 10-well polyacrylamide gels for SDS-PAGE. A voltage of 300 V was applied for approximately 20 min or until loading dye migrated to the bottom of the gel. Protein gels were imaged and crosslinked using a ChemiDoc MP Imaging System (Bio-Rad) and then transferred to nitrocellulose membranes using a Trans-Blot Turbo Transfer System (Bio-Rad). Transfers were conducted at 10 V for 35 min to avoid overheating. Stain-free blots were imaged using the ChemiDoc and were saved for antibody probing. Protein bands were visualized using Clarity Western ECL Substrate (Bio-Rad). Bands were normalized to both total protein loading and α-tubulin using Image Lab software version 6.1 (Bio-Rad). 

#### 2.4.1. COX-1 Western Blot

Protein blots were probed with COX-1 rabbit mAB (Abclonal (Woburn, MA, USA) #A4301) at a dilution of 1:2,000 and the goat anti-rabbit IgG Starbright Blue 700 secondary antibody (Bio-Rad #12004161) at a dilution of 1:2,500. Membranes were incubated at room temperature for an hour.

#### 2.4.2. COX-2 Western Blot

Protein blots were probed with COX-2 mouse mAb (Invitrogen #35-8200) at a dilution of 1:500 and peroxidase AffiniPure goat anti-mouse secondary antibody (Jackson ImmunoResearch (West Grove, PA, USA) #115-035-003) at a dilution of 1:20,000. Membranes were incubated at room temperature for an hour.

### 2.5. Fibroblast Culture

Vestibular and vulvar fibroblast strains (each obtained from a different patient or healthy control) were cultured in Minimum Essential Medium (MEM) supplemented with 10% FBS, GlutaMAX, gentamicin, and antibiotic/antimycotic solution (Gibco/Invitrogen/Thermo Fisher Scientific). Early passage (4–10) external vulvar and vestibular fibroblast strains were seeded at 2.5 × 10^4^ cells/cm^2^. After cultures reached full confluence, cells were collected and used for enzymatic activity, gene expression, and protein expression assays. For experiments using treated fibroblasts, cells were serum-starved for 24 h in MEM lacking FBS and then were treated for 48 h in media containing IL-1β [500 pg/mL] (Invitrogen), AA [1 µM] (Cayman Chemical Company, Ann Arbor, MI, USA), or a combination treatment. Cells were then collected for subsequent experimentation as described below.

### 2.6. Lipidomic Analysis

Primary fibroblast strains were seeded into 12-well tissue culture plates in MEM supplemented with 10% FBS, GlutaMax, gentamycin, and antibiotic/antimycotic solution (Thermo Fisher). Once confluent, cells were transitioned to serum-free, phenol-free MEM for 4–24 h. The following treatments were then prepared in serum-free, phenol-free MEM and applied to cultures for 48 h prior to sample collection: rhIL-1β [500 pg/mL] (Invitrogen), arachidonic acid (AA) [1 µM] (Cayman Chemical Company), and Poly(I:C) [50 pg/mL] (Sigma Aldrich, St. Louis, MO, USA). After 48 h, fibroblasts were manually scraped off the culture plates, collected in 1.5 mL microcentrifuge tubes, and homogenized using a probe sonicator. Furthermore, 0.1% butylated hydroxytoluene (BHT, Sigma Aldrich) was added to each sample to stabilize lipids. Samples were then stored at −80 °C until analysis. Quantitative targeted lipidomic analysis was performed by the Wayne State University Lipidomics Core using liquid chromatography mass spectrometry (LC-MS). Briefly, samples were prepared using C18 reverse-phase cartridges (Phenomenex (Torrance, CA, USA) StrataX SPE cartridge, 30 mg sorbent) and then subjected to reverse-phase HPLC on a C18 column (Luna, C18, 3 μm, 2 mm × 150 mm, Phenomenex) as published earlier [19]. A set of internal standards that matched the chemical structure and HPLC retention times of analytes as closely as possible was used. The same internal standards are used across all batches, allowing high accuracy for comparison, and samples were analyzed together for the best statistical outcome.

### 2.7. Lipoxygenase Enzymatic Activity Assay

To quantify lipoxygenase activity in fibroblasts, a fluorometric enzymatic activity assay kit (Catalog Number MAK363; Sigma-Aldrich) was utilized. In brief, fibroblasts were grown to confluence in 100 mm tissue culture dishes containing Minimum Essential Medium (MEM) supplemented with 10% FBS, GlutaMAX, gentamicin, and antibiotic/antimycotic solution (Gibco/Invitrogen/Thermo Fisher Scientific). Furthermore, 24 h prior to sample collection, MEM was replaced with serum-free MEM lacking FBS to starve cells. On the day of the assay, MEM was removed, and cultures were washed with 1X PBS to remove residual media. LOX lysis buffer was added to each culture, and cells were collected using disposable cell scrapers. Samples were homogenized by mechanical disruption using hypodermic needles with syringes. Protein was then collected and quantified according to the manufacturer’s protocol, and samples were analyzed the same day. Background control, sample, and inhibited reactions were prepared for each strain of fibroblasts. Reactions were organized in a 96-well plate and were initiated with the addition of a mixture of the LOX probe and LOX substrate. Fluorescence was monitored at Ex 500 nm/Em 536 nm for 40 min and recorded every 30 s using a FlexStation Multi-Mode Microplate Reader (Molecular Devices, San Jose, CA, USA).

### 2.8. Statistical Analysis

For each comparison of mRNA expression, cytokine abundance, or lipid quantity, we fit a linear mixed effects model with fixed effects and their interactions for LPV status (case/control), location (vest/vulv), and treatment, and a random effect for patient ID to account for technical replicates. All tests used a significance level of α = 0.05. First, main effects for treatment differences were tested, then the pairwise contrasts between the four LPV status and location combinations were tested, and the significance of these is denoted in the figures. Heatmaps for the lipid quantities were sorted by average quantity of all vehicle-treated samples. Each lipid (row) was scaled by the maximum quantity for that lipid so the relative quantities of lipids on different treatments are apparent, but comparison of quantities between lipids is not possible in these figures. For the 120 lipids that were above the limit of detection in at least one sample, principal component analysis (PCA) was performed on the samples from vehicle, AA, IL1B + AA, and POLY IC + AA tissue. Plots of the samples along the first three principal components were created to illustrate the major differences between the tissue types.

## 3. Results

### 3.1. Painful Vestibular Tissue from LPV Patients Exhibits Altered Expression of Cyclooxygenase (COX) and Lipoxygenase (LOX) Enzymes

**Figure 1:**
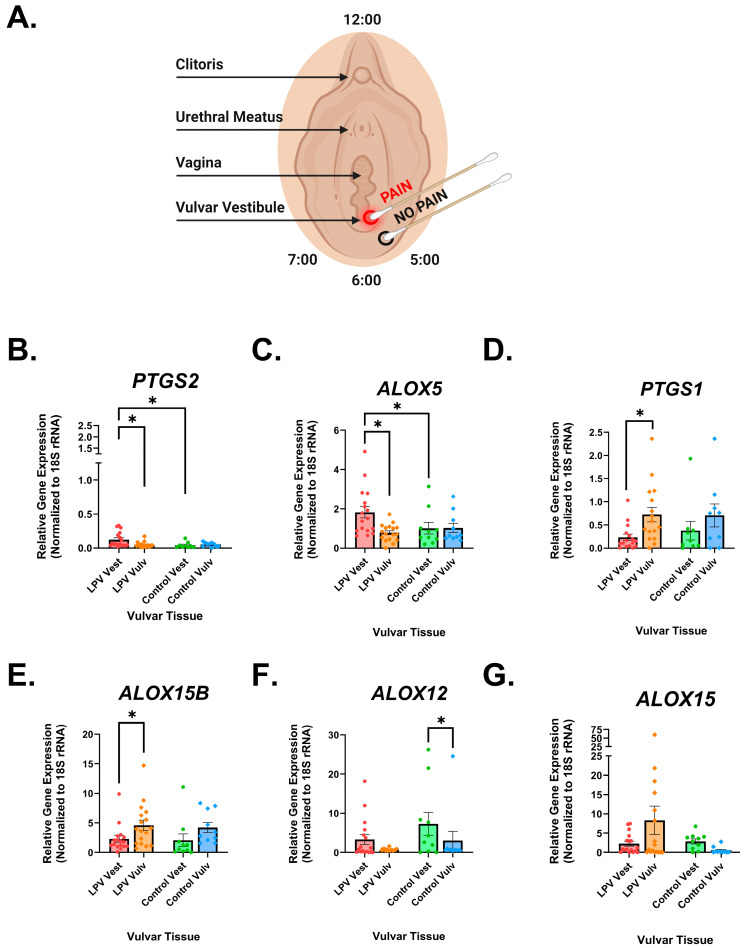
LPV vestibular tissue displays altered expression of cyclooxygenase (COX) and lipoxygenase (LOX) enzymes. (**A**) Illustration of the Q-tip test used to diagnose LPV. Light touch to the tissue surrounding the vaginal opening (the vulvar vestibule, referred to as “Vest” throughout) is immensely painful, whereas touching the adjacent external vulva (referred to as “Vulv”) elicits little pain. Moreover, 6 mm punch biopsies of vestibular and vulvar tissue were collected from LPV patients and healthy controls during routine gynecologic surgeries. Biopsies were cut into three pieces, with one piece being used to assess expression of COX/LOX enzymes. (**B**–**G**) Individual plots of qPCR results. COX-2 (*PTGS2*) and 5-LOX (*ALOX5*) mRNA levels were significantly elevated in the painful LPV vestibule compared to non-painful controls, while expressions of COX-1 (*PTGS1*) and 15-LOX-2 (*ALOX15B*) were decreased. 12-LOX (*ALOX12*) expression was enhanced in the vestibule of control patients with no significant changes identified in LPV tissue. No significant differences were observed in 15-LOX-1 (*ALOX15*) expression across the four groups. All data points were normalized to 18S rRNA expression, and statistical significance was determined utilizing linear mixed effects models. LPV/control and vest/vulva sampling sites were treated as fixed effects and biological replicates as random effects. Data are represented with mean ± SEM, *n* = 17 LPV and *n* = 10 controls. *p* < 0.05 was used as the threshold for significance, with * designating pairwise significance.

To investigate possible differences in the expression of COX/LOX enzymes, 6 mm punch biopsies were collected from the painful vestibule (vest) and non-painful vulva (vulv) of both LPV patients and control patients (Figure 1A). The mRNA expression of COX/LOX enzymes was evaluated using qRT-PCR and normalized to 18S rRNA expression. We found that COX-2 (*PTGS2*) mRNA expression was higher in the LPV vest compared to the control vest (Figure 1B).

There was also a significant increase in the mRNA expression of 5-LOX (*ALOX5*) in the LPV vest compared to the LPV vulv (Figure 1C). 5-LOX catalyzes the conversion of AA to leukotriene A4 (LTA4), a precursor for the powerful chemoattractant leukotriene B4 (LTB4), which is critical in recruiting neutrophils to the site of inflammation [35,36]. Overall, COX-2 and 5-LOX are overexpressed in the LPV vest compared to the control vest, suggesting a sustained inflammatory state in LPV patients.

The mRNA expression of COX-1 (*PTGS1*) (Figure 1D) and 15-LOX-2 (*ALOX15B*) (Figure 1E) was decreased in the LPV vestibule compared to the LPV vulva, whereas there were no discernible differences in expression of these enzymes between the control vest and the LPV vest. The expression of 12-LOX (*ALOX12*) was increased in the control vest compared to the control vulva, with no significant differences between the LPV vulva and LPV vest (Figure 1F). There were no significant differences in the expression of 15-LOX (*ALOX15*) (Figure 1G).

### 3.2. Vulvar Fibroblasts Show Altered Expression of Cyclooxygenase-1 (COX-1) and Cyclooxygenase-2 (COX-2) Expression upon Treatment with Inflammatory Stimuli

**Figure 2:**
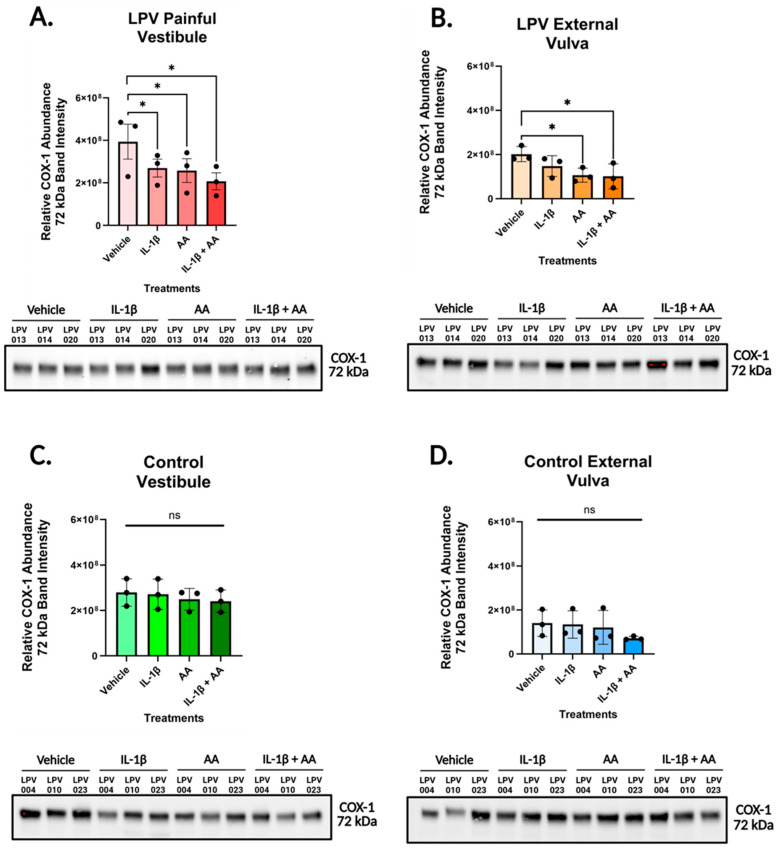
COX-1 levels decline in LPV cases with IL-1β and AA treatment. Vulvar fibroblasts were incubated with vehicle, IL-1β [500 pg/mL], arachidonic acid (AA) [1 µM], or a combination of IL-1β and AA treatments for 48 h prior to being lysed for Western blot analyses. Membranes were probed with COX-1 rabbit mAB (Abclonal # A4301) at a dilution of 1:2000 and the goat anti-rabbit IgG Starbright Blue 700 secondary antibody (Bio-Rad #12004161) at a dilution of 1:2500. COX-1 expression levels for (**A**) LPV vestibular, (**B**) LPV external vulvar, (**C**) control vestibular, and (**D**) control external vulvar fibroblasts were assessed for three cases and three controls. Stimulation with AA alone and in combination with IL-1β decreased expression of COX-1 in case vestibular and vulvar fibroblasts. No statistically significant expression changes were identified in controls across the four conditions. Cropped Western blot images used for analysis appear below each graph. COX-1 bands were identified at 72 kDa. Full blot images are available in Figure S3. Statistical significance was determined utilizing linear mixed-effect models where cell treatments were assigned as fixed effects, and patient ID was treated as a random effect to account for biological replicates. *p* < 0.05 was used as the threshold for significance, with * designating significant changes between the four treatments. ns denotes comparisons that did not reach significance (ns = not significant).

After establishing baseline expression in tissue, primary human vulvar fibroblast cells were treated with vehicle, IL-1β (500 pg/mL), AA (1 µM), or a combination of IL-1β and AA for 48 h, and cell lysates were collected for Western blot analysis. IL-1β is a potent pro-inflammatory cytokine and key mediator of inflammation used here to mimic endogenous inflammatory stimuli [37]. In the LPV vest (Figure 2A) and the LPV vulva (Figure 2B), stimulation with AA alone and in combination with IL-1β reduced the expression of COX-1 compared to the vehicle. However, in the control vest (Figure 2C) and the control vulva (Figure 2D), there were no significant differences in the expression of COX-1 across treatments. Overall, we did not denote any case- or site-specific differences in COX-1 expression, while there were significant effects in LPV cases in response to treatment.

**Figure 3:**
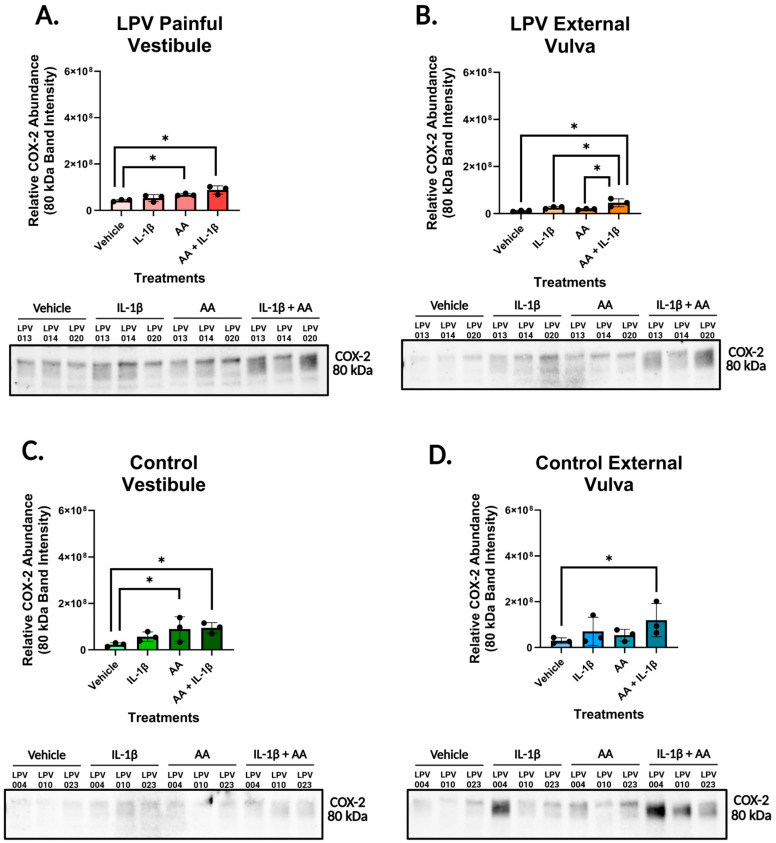
Arachidonic acid induces COX-2 expression in vulvar fibroblasts. Cells were incubated with vehicle, IL-1β [500 pg/mL], arachidonic acid (AA) [1 µM], or a combination of IL-1β and AA treatments for 48 h and then were collected for Western blot analyses. Membranes were probed with COX-2 mouse mAb (Invitrogen #35-8200) at a dilution of 1:500 and peroxidase AffiniPure goat anti-mouse secondary antibody (Jackson ImmunoResearch #115-035-003) at a dilution of 1:20,000. COX-2 expression levels for (**A**) LPV vestibular, (**B**) LPV external vulvar, (**C**) control vestibular, and (**D**) control external vulvar fibroblasts were assessed for three cases and three controls. Addition of AA alone and in combination with IL-1β stimulated COX-2 expression in LPV and control vestibular cells. Addition of AA with IL-1β also significantly increased COX-2 expression in LPV and control external vulvar fibroblasts. Cropped Western blot images used for analysis appear below each graph. COX-2 bands at 80 kDa were used for analysis. Full blot images are available in Figure S3. Statistical significance was determined utilizing linear mixed-effect models where cell treatments were assigned as fixed effects, and patient IDs were treated as random effects to account for biological replication. *p* < 0.05 was used as the threshold for significance, with * designating significant changes between the four treatments.

In the LPV vest (Figure 3A) and the control vest (Figure 3C), stimulation with AA to model dietary intake of AA, alone and in combination with IL-1β, significantly increased the expression of COX-2 compared to vehicle. In the LPV vulva (Figure 3B), treatment with AA and IL-1β significantly increased COX-2 expression compared to all other treatments. In the control vulva (Figure 3D), treatment with AA and IL-1β increased the expression of COX-2 compared to the vehicle.

Increased expression of COX-2 in combination with reduced expression of COX-1 could lead to the accumulation of PGE2 previously observed by our group in LPV vest fibroblasts [2].

### 3.3. Vulvar Fibroblasts Show Enhanced Production of Pro-Inflammatory Mediators upon Treatment with Arachidonic Acid

**Figure 4:**
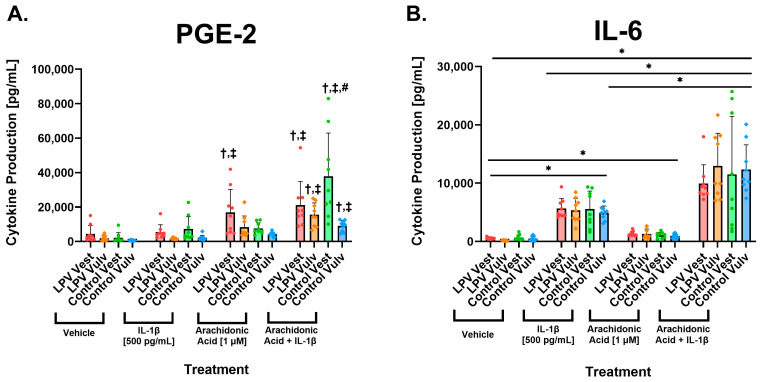
Pro-inflammatory cytokine production is enhanced with arachidonic acid treatment in vulvar fibroblasts. Cells were incubated with IL-1β [500 pg/mL], arachidonic acid (AA) [1 µM], and a combination of treatments for 48 h, and supernatants were collected for ELISA analyses. (**A**) Levels of prostaglandin-E2 (PGE_2_) and (**B**) interleukin-6 (IL-6) in vulvar cells after 48-h vehicle, IL-1β [500 pg/mL], arachidonic acid (AA) [1 µM], and combination treatments. Addition of AA significantly elevated production of PGE_2_ in LPV vestibular cells, and addition of AA in combination with IL-1β significantly stimulated production of PGE_2_ in all strains. IL-1β alone and in combination with AA also induced IL-6 production in both LPV and control fibroblasts. Statistical significance was determined utilizing linear mixed effects models treating LPV/control, location, and treatments as fixed effects and technical replicates as random effects. *p* < 0.05 was used as the threshold for statistical significance, and in the graphs, “†” denotes significant change from vehicle, “‡” denotes significant change from IL-1β, and “#” denotes significant change from arachidonic acid for a particular group (i.e., LPV vest). Data were measured in triplicate and represented with mean ± SEM, *n* = 3 LPV, and three controls per treatment. *p* < 0.05 was used as the threshold for statistical significance denoted by *.

To further explore how these differences in expression might affect the production of inflammatory mediators, vulvar fibroblasts were treated with IL-1β (500 pg/mL), AA (1 µM), and a combination of IL-1β and AA. *ALOX15* and *ALOX15B* are not consistently expressed in fibroblasts; thus, we quantified *ALOX5* and *ALOX12*. When treated with a combination of AA and IL-1β, all groups showed enhanced production of PGE2 compared to treatment with vehicle or IL-1β (Figure 4A). In the LPV vest, treatment with AA alone was sufficient to enhance the production of PGE2 compared to the vehicle and to IL1-β (Figure 4A). This might be due to the increased expression of COX-2 (*PTGS2*) observed in the LPV vest (Figure 1E).

Treatment with AA induced a small increase in the production of IL-6 in all groups compared to the vehicle (Figure 4B). However, when cells were treated with a combination of AA and IL-1β, IL-6 production was significantly enhanced compared to treatment with IL-1β or AA alone (Figure 4B).

### 3.4. Lipoxygenase Expression and Enzymatic Activity in Vulvar Fibroblasts

**Figure 5:**
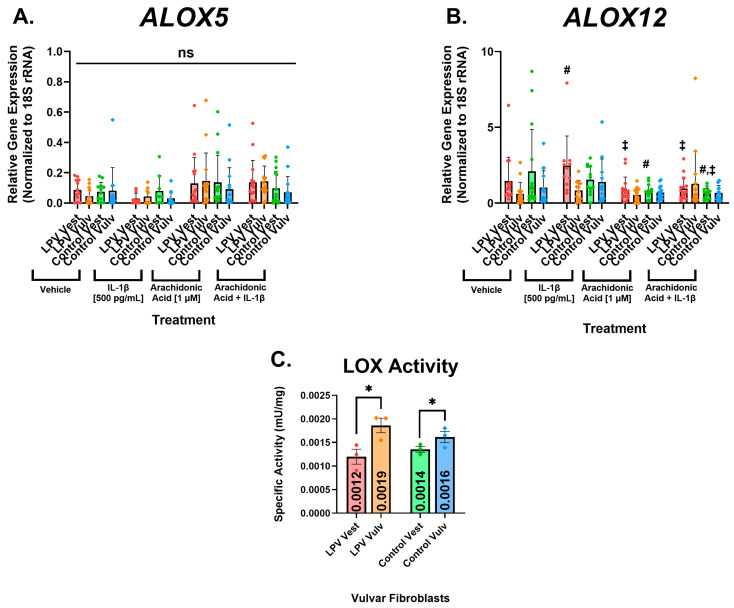
Lipoxygenase expression and enzymatic activity in vulvar fibroblasts. Cells were incubated with vehicle, IL-1β [500 pg/mL], arachidonic acid (AA), and AA in combination with IL-1β for 48 h prior to extracting RNA for gene expression analyses. Levels of (**A**) *ALOX5* and (**B**) *ALOX12* were assessed utilizing RT-qPCR. No significant changes in ALOX5 were observed, whereas *ALOX12* was enhanced upon treatment with IL-1β for LPV vestibular cells. The addition of arachidonic acid and IL-1β reduced *ALOX12* expression for both LPV and control vestibular fibroblasts. No changes in external vulvar *ALOX12* expression were observed. *ALOX15* and *ALOX15B* were undetectable in vulvar fibroblast cells. (**C**) Lipoxygenase activity assay of vulvar fibroblasts revealed reduced enzymatic activity of LOX enzymes in vestibular fibroblasts compared to cells cultured from the external vulva. Specific activity was calculated by subtracting the activity of samples incubated with a LOX inhibitor from uninhibited samples. One unit is defined as the amount of lipoxygenase that oxidizes 1 µmol of LOX probe per minute at a pH of 7.4 at room temperature. Statistical significance was determined utilizing linear mixed effects models with fixed effects for LPV/control, location, and treatment groups and random effects for patient ID. *p* < 0.05 was used as the threshold for statistical significance denoted by *, and in the graphs, “†” denotes significant change from vehicle, “‡” denotes significant change from IL-1β, and “#” denotes significant change from arachidonic acid for a particular group. Data are represented with mean ± SEM, *n* = 5 LPV, and six controls measured in triplicate for qPCR assays, and *n* = 3 for the LOX activity assay.

Lipoxygenase expression was also assessed upon treatment with vehicle, IL-1β (500 pg/mL), AA (1 µM), and a combination of AA and IL-1β. No groups showed significant differences in *ALOX5* (5-LOX) mRNA expression between treatments (Figure 5A). However, there was a slight trend towards increased *ALOX5* expression when fibroblasts were treated with AA alone or a combination of both AA and IL-1β compared to when they were treated with vehicle or IL-1β alone. However, this difference failed to reach significance for any group (Figure 5A).

The LPV vest showed enhanced mRNA expression of *ALOX12* (12-LOX) when treated with IL-1β compared to the vehicle, while all other groups showed no significant differences (Figure 5B). Interestingly, in the LPV vest, treatment with AA or a combination of AA and IL-1β decreased the expression of *ALOX12* compared to IL-1β alone (Figure 5B). The control vest also showed decreased mRNA expression of *ALOX12* when treated with AA or a combination of IL-1β or AA compared to the vehicle (Figure 5B).

While there was no significant difference between the LPV vestibule and the control vestibule, both the LPV vest and the control vest showed decreased LOX activity compared to their respective vulva fibroblasts (Figure 5C). This indicates that LOX activity is deficient in the vestibule and may contribute to lipid dysbiosis in LPV. While not statistically significantly different from the control vest, the LPV vest showed the overall lowest LOX activity, consistent with AA lipid dysbiosis in the painful vestibule.

### 3.5. Vulvar Fibroblasts Showed Altered Production of AA-Derived Lipid Mediators upon Treatment with Arachidonic Acid

Next, we evaluated how lipid synthesis might be impacted by the changes in expression we observed. LC-MS lipidomic analysis of 143 lipids was carried out on vulvar fibroblasts treated with vehicle, AA, a combination of AA and IL-1β, or a combination of AA and Poly(I:C) (50 pg/mL), a synthetic double-stranded RNA molecule (viral mimetic) known to induce an inflammatory response [38]. We chose to use both IL-1β and Poly(I:C) to mimic both endogenous and exogenous inflammatory stimuli, respectively, and to parse out any possible differences in inflammatory response.

**Figure 6:**
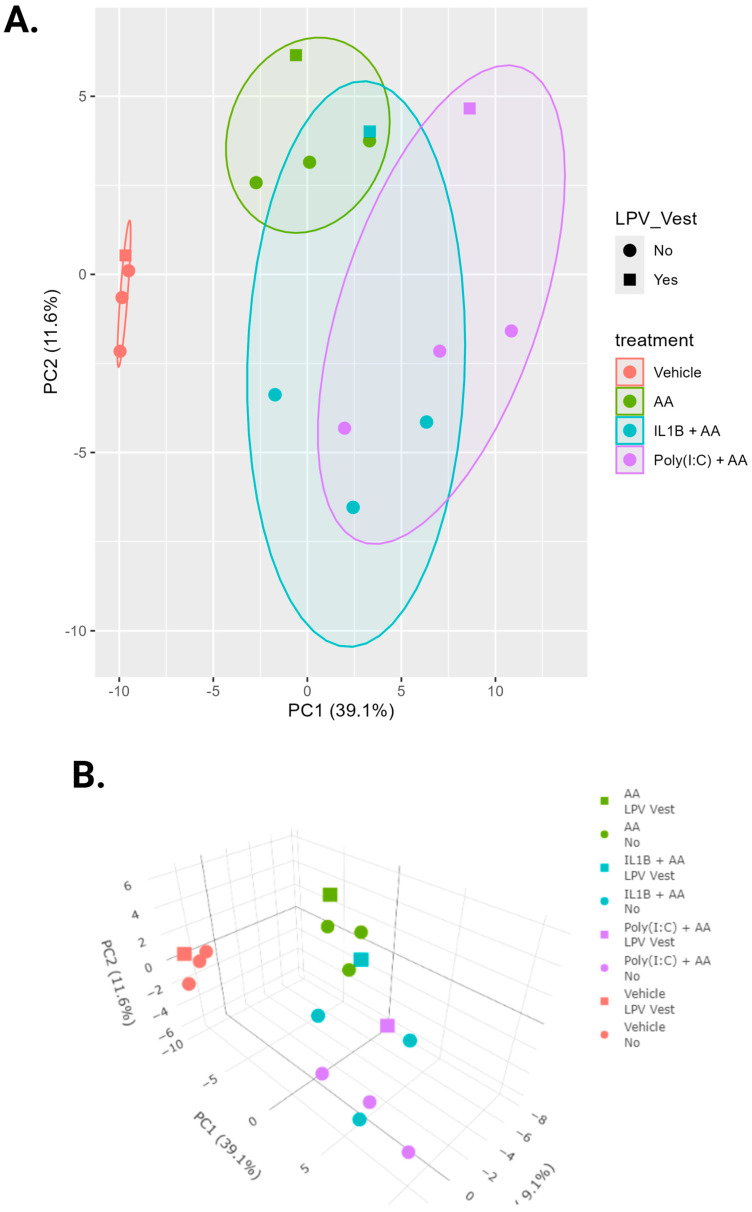

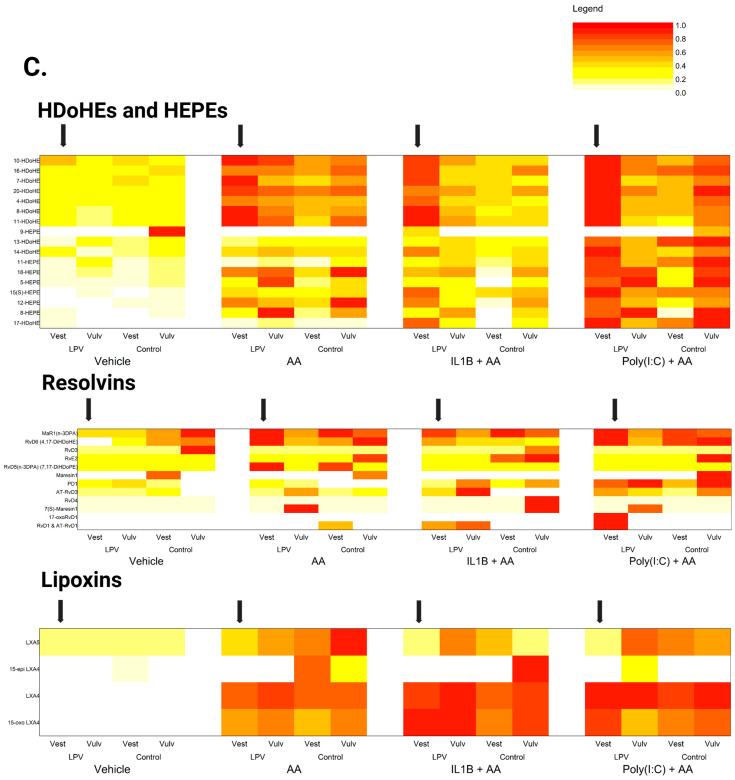
Targeted metabololipidomics of arachidonic acid-derived lipid mediators. LC-MS lipidomic analysis of >150 lipids in vulvar fibroblasts treated with arachidonic acid (AA) [1 µM], IL-1β [500 pg/mL] in combination with arachidonic acid, and Poly(I:C) [50 pg/mL] in combination with arachidonic acid. Principal component analysis (PCA) was performed on samples from VEHICLE, AA, IL-1β + AA, and Poly(I:C) + AA (shown in different colors). (**A**) 2D and (**B**) 3D plots of the samples along the first principal components were created to illustrate the major differences between the tissue types and denote LPV vest tissue with a different symbol than the other tissues. (**C**) Heatmaps of lipids separated by general eicosanoid type. The LPV vest is the fourth column in each group (denoted with a large arrow at the top); the LPV vest has increases in most HDoHEs and HEPEs under both endogenous and exogenous inflammatory stimuli. Heatmaps for the lipids were sorted by average quantity of all vehicle-treated samples. Each lipid (row) was scaled by the maximum quantity for that lipid so the relative quantities of lipids in different treatments are apparent. Heatmaps were constructed to visualize trends across groups/treatments, and comparison of quantities between lipids is not possible in these figures. Therefore, selected lipids are graphed in Figure 7. *n* = 3 cases and three controls run in triplicate.

**Figure 7:**
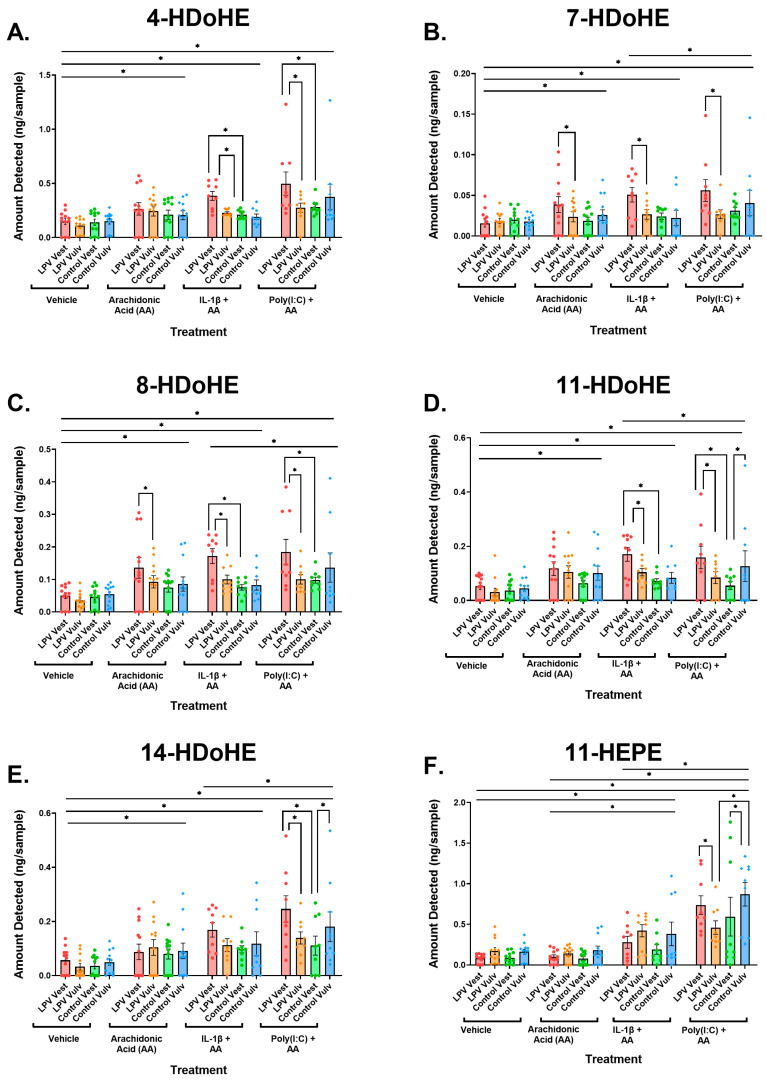

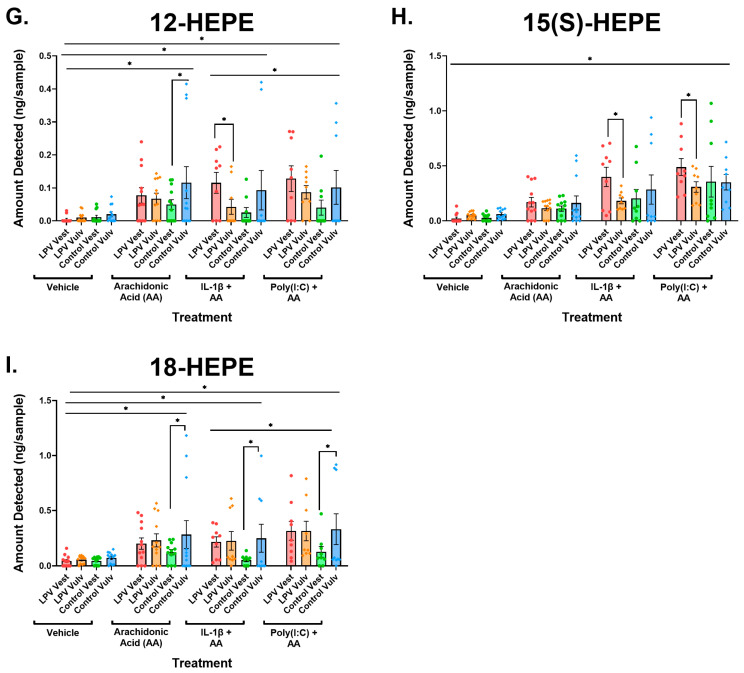
Individual lipid plots of metabolites of interest. (**A**–**I**) LC-MS lipidomic analysis of >150 lipid species for vulvar fibroblasts treated with vehicle, arachidonic acid (AA) [1 µM], IL-1β [500 pg/mL] in combination with arachidonic acid, and Poly(I:C) [50 pg/mL] in combination with arachidonic acid. Straight lines denote differences between two treatment groups at either end of the line. Bars with ticks denote significant differences between LPV/control locations under the ticks. Data were measured in triplicate and displayed with mean ± SEM, *n* = 3 LPV and three controls per treatment, run in triplicate. Statistical significance was determined utilizing linear mixed effects models with fixed effects for LPV/control, location, and treatment groups and random effects for patient ID. *p* < 0.05 was used as the threshold for significance, with * designating significant differences between groups.

Principal component analysis (PCA) analysis shows that the first principal component distinguishes the vehicle samples from the samples exposed to treatments (Figure 6A). The first two principal components explained 50.7% of the variability in these lipids. Within each treatment group, the samples taken from vestibular tissue in LPV (case) patients were different from the other three sample types within the same treatment (Figure 6A,B). The most striking case–control difference is in the production of HDoHEs and HEPEs, where the LPV vest showed increased production compared to the control vest, which did not correspond to case–control differences in the production of SPMs (Figure 6C). The LPV vest showed increased amounts of 4-, 7-, 8-, 11-, and 14-HDoHE in response to inflammatory stimuli compared to the control vest) (Figure 7A–E). The LPV vest also showed increased production of 11-, 12-, 18-, and 15(S)-HEPE in response to inflammatory stimuli compared to the control vest (Figure 7F–I).

HDoHEs and HEPEs are biosynthesized from dietary DHA and EPA, respectively, and many of those enriched in the LPV vest are metabolized into SPMs, such as maresin 1, resolvin E1, and resolvin E4, by the action of LOX enzymes [39,40,41,42,43,44]. The increased abundance of HDoHEs and HEPEs in LPV vests upon treatment with inflammatory stimuli suggests an exaggerated inflammatory response as well as an inability to metabolize HDoHEs and HEPEs into the SPMs that make up the resolution program. With increased pools of HDoHEs and HEPEs, we would anticipate increased pools of SPMs in the case vest. However, there were no significant differences in SPMs between LPV case and control fibroblasts (Figure S5). Figure S5 depicts graphs of all 143 lipids for detailed analysis of these results.

## 4. Discussion

**Figure 8:**
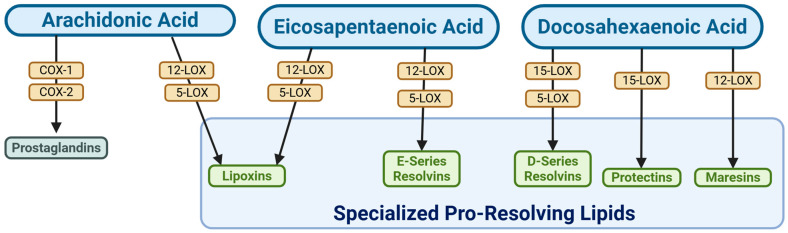
Simplified overview of SPM biosynthesis. Arachidonic acid, eicosapentaenoic acid, and docosahexaenoic acid metabolism by LOX and COX enzymes is depicted. Prostaglandins are inflammatory, while specialized pro-resolving mediators resolve inflammation.

Despite limited knowledge of the etiology of LPV, there is evidence of its link to inflammation [18,45], but more specifically an inability to resolve it. The metabolites of dietary PUFAs play a role in the initial inflammatory response and the resolution machinery. The bioactive metabolites of AA, such as prostaglandins, leukotrienes, HETEs, and thromboxanes, are largely involved in the immune system’s initial response to microbes and infection [16,40,46,47,48]. In this paper, we show that there are key differences in COX/LOX enzyme systems and dietary AA metabolism that contribute to the enhanced inflammation observed in the LPV vestibule. PUFAs are metabolized by LOX and COX enzymes into pro-inflammatory and pro-resolving lipids (Figure 8).

One such difference is the overexpression of *PTGS2* (COX-2) in LPV vestibular tissue, which indicates a system primed for an exaggerated inflammatory response. This could also point to a sustained inflammatory state. Vaginal swabs from LPV patients were found to have increased production of pro-inflammatory cytokines, including IL-1 and TNF-α, which have been shown to induce COX-2 expression [10,16,49]. Non-steroidal anti-inflammatory drugs (NSAIDs) inhibit the activity of both COX isoforms and exhibit anti-inflammatory effects by non-specifically blocking COX activity and have efficacy in treating acute pain conditions with limited success in treating chronic pain [50]. However, COX inhibition can impact other functions that require prostaglandins, affect blood pressure regulation, and increase the risk of gastroduodenal ulcers [51]. Furthermore, NSAIDs and steroids, which globally block inflammation, have been shown to be ineffective for vulvodynia, while there is at least modest efficacy for antidepressants and antiepileptics, although neither has proven more effective than placebo in randomized trials [1,18].

COXIBs are selective inhibitors of COX-2 and can lower the risk for gastrointestinal adverse outcomes but do not completely eliminate this risk [52]. COX-2′s role is not limited to the production of pro-inflammatory prostaglandins, but it is also important in the resolution phase, as other products of COX-2, that is, lipoxins, exert anti-inflammatory effects [53,54]. PGE2 itself can have anti-inflammatory effects depending on the cell and receptor type, rendering it vital in the resolution phase [53,55]. Addressing inflammation in LPV patients is unlikely to be as easy as “flipping a switch” to turn it off.

Other avenues for addressing inflammation may present themselves with our findings. Expression of *ALOX5* (5-LOX) was also significantly elevated in the case versus control vestibular tissue, which is consistent with enhanced leukotriene synthesis in case fibroblasts (Figure 6). The overexpression of 5-LOX has been associated with inflammation in systemic sclerosis arthritis, inflammatory bowel disease, ulcerative colitis, and gastrointestinal cancers [56,57,58,59]. Unlike prostaglandins, leukotrienes are almost exclusively expressed in inflammatory cells [60]. Dual inhibition of COX-2 and 5-LOX might prove to be a viable method to target inflammation. Other groups have found the products of COX-2 and 5-LOX both increased in diseases such as colorectal cancer and arthritis [61,62,63]. Dual inhibitors are already in development. Thus, targeting both enzymes in resolving inflammation might be a promising strategy. These dual inhibitors have fewer adverse side effects, are more effective, and are helpful for avoiding dosing issues and drug interactions that may occur with separate drugs [60,64,65]. More research is necessary to explore the efficacy of these dual inhibitors in treating LPV and providing relief for patients.

Levels of 15-LOX-1 and 15-LOX-2 were below the threshold of detection in fibroblasts and could only be assessed in tissue, suggesting that 5-LOX and 12-LOX are the prominent LOXs in vulvar fibroblasts. A notable difference in LOX expression was the increased mRNA expression of *ALOX12* (12-LOX) in the LPV vestibular fibroblasts in response to inflammatory stimuli, which likely accounts for the increased abundance of 11-HDoHE, 14-HDoHE, and 12-HEPE [66,67,68]. While we did not observe a case–control difference in LOX activity, the case vestibule showed significantly reduced activity compared to its respective external vulvar control. Previous work from our group has shown that 12-HETE, another metabolite of 12-LOX, is reduced in the LPV vestibular fibroblasts [18,19], which may be at least partially explained by reduced LOX activity. Likely, a larger patient and control sample would be needed to tease out any case–control differences.

This suggests that LOX activity may not be dictated by mRNA expression. While there could be post-translational or post-transcriptional modifications that might explain the disconnect between LOX expression and activity, another plausible explanation is the existence of a polymorphism in one or more of the LOX genes. SNPs in *ALOX12* have been associated with peripheral neuropathy and menstrual dysfunction and have already been identified [69,70]. However, there is a need for high-throughput exploration of genetic differences between cases and controls in LOX genes as well as testing the effects on LOX activity during inflammation. At present, obtaining a diagnosis for LPV can be a long and expensive process, but identification of a genetic component would significantly improve diagnosis criteria.

The vestibule is less able to metabolize AA via LOX, which can contribute to the observed lipid dysbiosis in LPV [18,19]. Furthermore, 12-LOX is involved in the generation of maresin 1 [71], which stimulates resolution and reduces pain [72], but we did not see an increase in this SPM. Many HDoHEs and HEPEs are the precursors to SPMs [73,74] and were enriched in the LPV vestibular fibroblasts treated with inflammatory stimuli, but again this did not translate to an increased abundance of SPMs, pointing to altered metabolism of dietary PUFAs in vulvodynia. SPMs are a critical part of the resolution machinery, and other researchers have found that SPMs can reduce inflammation and control pain [75,76,77,78]. In agreement, our group also found that SPMs were able to reduce the production of PGE2 and IL-6 in vulvar fibroblasts [27].

Altered SPM synthesis has been implicated in other disease states. Individuals with severe asthma show reduced capacity to synthesize lipoxin A_4_ and 15-HETE from AA [25]. In cystic fibrosis, patients with detectable levels of resolvin E1 show better lung function than patients that do not have detectable levels of this SPM [20]. Lipidomic analysis in eosinophil-depleted mice revealed a deficiency in protectin D1 [79]. However, the role of SPM deficiency in LPV patients is yet unclear and would require eventual clinical trials.

Regardless of the source of this incongruence in LPV, the apparent inability to biosynthesize SPMs in response to inflammatory stimuli may explain why LPV patients exhibit an enhanced inflammatory response. The effects of SPMs (which are classified into lipoxins, protectins, maresins, and resolvins) [80] are far-reaching. SPMs promote resolution by antagonizing cytokines, inhibiting degranulation of mast cells, limiting PMN infiltration, inducing macrophage phagocytosis, and enhancing tissue regeneration [80,81,82]. In fact, aspirin can trigger the resolution phase by generating epimers of SPMs [83]. SPMs are biosynthesized mainly through the action of ALOX15, ALOX15B, ALOX12, and ALOX5 [81,83,84], which can be targeted in future studies. By parsing out the possible genetic/translational differences in these genes between healthy controls and LPV patients, we might reveal more about how SPM biosynthesis can affect inflammation and thus pain. Again, identification of a genetic factor could also lead to widespread, simple, and objective testing for LPV.

There is growing evidence of the link between LPV and inflammation, requiring more exploration of how the inability to resolve inflammation can lead to pain. As more studies illuminate our understanding of how SPMs mediate resolution, we have the impetus to further explore the genetic and mechanistic basis for lipid dysregulation, which is already implicated in many other inflammatory diseases [85,86,87,88], in LPV patients in hopes of providing effective treatment for this disabling disease.

## 5. Conclusions

While it is still too early to predict the effects of dietary modification on vulvodynia, reducing sources of dietary AA may help to reduce the production of inflammatory mediators. However, there is still a missing pool of SPMs that are produced from AA in healthy individuals. Concomitant supplementation with other dietary PUFAs (e.g., DHA and EPA) might help to compensate for the loss of AA-derived resolving lipids in vulvodynia. In mice, topical DHA production reduces vulvar allodynia [27], but dietary supplementation remains to be explored. While our in vitro models have been validated to represent outcomes in the patient, a significant limitation of this work is that we have not yet tested these interventions in LPV patients. However, this work is necessary to establish the applicability and likely safety of such interventions before undergoing clinical trials.

## Data Availability

The data presented in this study can be downloaded from Zenodo at https://doi.org/10.5281/zenodo.15548333.

## Supplementary Materials

The following supporting information can be downloaded at: https://www.mdpi.com/article/10.3390/nu17132233/s1. Supplementary Figure S1: Flowchart for selecting study cases; Supplementary Figure S2: Flowchart for selecting study controls; Supplementary Table S1: Study subject demographic table; Supplementary Figure S3: Full Western blot data; Supplementary Figure S4: Lipoxygenase activity assay time course data; Supplementary Figure S5: All individual lipid bar plots; Supplementary Table S2: List of primers; Supplementary Table S3: List of lipids quantified using metabololipidomics; Supplementary Table S4: All arachidonic acid metabololipidomics data.

## Abbreviations

The following abbreviations are used in this manuscript:

AA	arachidonic acid
COX	cyclooxygenase
DHA	docosahexaenoic acid
EET	epoxyeicosatrienoic acid
EPA	eicosapentaenoic acid
HDoHE	hydroxydocosahexaenoic acid
HEPE	hydroxyeicosapentaenoate
HETE	hydroxyeicosatetraenoic acid
LOX	lipoxygenase
LPV	localized provoked vulvodynia
NSAID	non-ateroidal anti-inflammatory drug
PUFA	polyunsaturated fatty acid
SNP	single-nucleotide polymorphism
SPM	specialized pro-resolving mediator
