# Supplementary material for: Dysregulation of Arachidonic Acid Metabolism Drives Inflammatory Lipid Production in Localized Provoked Vulvodynia
Source: Nutrients. 2025 Jul 5;17(13):2233. doi: 10.3390/nu17132233 (PMC12251515; doi:10.3390/nu17132233)
Supplement: Supplementary file 1 [file nutrients-17-02233-s001.zip › nutrients-3703920-supplementary.pdf]

Supplementary Figure S1: Flowchart for Selecting Study Cases

FLOWSHEET FOR IDENTIFYING PARTICIPANTS FOR VULVODYNIA STUDY:

CASES

START HERE

Has this patient been diagnosed with vulvodynia?

NO

Cannot be used as CASE in study. See opposite side for criteria in selecting CONTROLS.*

*If this patient has not yet been diagnosed with vulvodynia but there is a high probability that they will be diagnosed in the future, do not use as a control.

YES

Is this patient 18 years of age or older?

NO

Cannot be used in study.

YES

Does this patient have a planned surgical procedure to manage localized provoked vulvodynia?

NO

Cannot be used in the study but once they schedule surgery can then be re-assessed for participation in study.

YES

Do any of the following apply to this patient?

- Other vulvar conditions (including dermatoses, vulvitis, vulvar papillomatosis, neoplasia, atrophic vaginitis)
- Previous vestibulectomy
- Active vaginal infection
- Greater than 5% parabasal cells on maturation index
- Neuropathology including small fiber, diabetic, or post-herpetic neuropathy
- Pregnancy
- Use of systemic immune modulators, including oral or parenteral corticosteroids, DMARD's, TNF inhibitors
- Topical corticosteroids, calcineurin inhibitors, imiquimod, or proinflammatory substances such as podophyllotoxin and tricholoroacetic acid (TCA)
- Lower genital tract surgery or vaginal delivery within 6 weeks of present planned procedure
- Medical disorder associated with immune compromise such as HIV (+) status or myelodysplasia

YES

Cannot be used in the study.

NO

Can be approached about participation in study.

Supplementary Figure S2: Flowchart for Selecting Study Controls

FLOWSHEET FOR IDENTIFYING PARTICIPANTS FOR VULVODYNIA STUDY:

CONTROLS

START HERE

Is this patient negative for all three of "Friedrich's Criteria"?

NO

Cannot be used as CONTROL in study. See opposite side for criteria in selecting CASES.

YES

Is this patient 18 years of age or older?

NO

Cannot be used in study.

YES

Does this patient have a planned surgical procedure to lower genital tract?

NO

Cannot be used in study.

YES

Do any of the following apply to this patient?

- Other vulvar conditions (including dermatoses, vulvitis, vulvar papillomatosis, neoplasia, atrophic vaginitis)
- Previous vestibulectomy
- Active vaginal infection
- Greater than 5% parabasal cells on maturation index
- Neuropathology including small fiber, diabetic, or post-herpetic neuropathy
- Pregnancy
- Use of systemic immune modulators, including oral or parenteral corticosteroids, DMARD's, TNF inhibitors
- Topical corticosteroids, calcineurin inhibitors, imiquimod, or proinflammatory substances such as podophyllotoxin and tricholoroacetic acid (TCA)
- Lower genital tract surgery or vaginal delivery within 6 weeks of present planned procedure
- Medical disorder associated with immune compromise such as HIV (+) status or myelodysplasia

YES

Cannot be used in the study.

NO

Can be approached about participation in study.

Supplementary Table S1: Study Subject Demographic Table

Subject/Strain ID	Sample Type	Age	Race/Ethnicity	Vestibule Threshold	Vulva Threshold
LPV004	CONTROL	41	White, Not Hispanic	2.64	3.5
LPV008	CONTROL	26	White, Not Hispanic	1.95	3.87
LPV009	CASE	22	White, Not Hispanic	0.73	1.37
LPV010	CONTROL	36	White, Not Hispanic	1.80	4.00
LPV013	CASE	20	White, Not Hispanic	0.53	1.13
LPV014	CASE	61	White, Not Hispanic	0.52	1.15
LPV016	CONTROL	49	White, Not Hispanic	1.27	4.37
LPV017	CONTROL	31	White, Not Hispanic	1.62	5.00
LPV019	CASE	31	White, Not Hispanic	0.85	7.90
LPV020	CASE	25	White, Not Hispanic	3.27	0.70
LPV021	CONTROL	50	White, Not Hispanic	2.50	5.00
LPV022	CASE	22	White, Not Hispanic	0.68	4.52
LPV023	CONTROL	20	White, Not Hispanic	4.17	4.50
LPV201	CASE	26	White, Not Hispanic	0.03	1.83
LPV202	CONTROL	27	White, Not Hispanic	0.70	5.17
LPV203	CASE	37	White, Not Hispanic	0.07	1.30
LPV204	CONTROL	37	White, Not Hispanic	4.23	>5
LPV205	CASE	28	White, Not Hispanic	1.04	2.70
LPV206	CASE	31	White, Not Hispanic	0.29	2.78
LPV207	CONTROL	32	White, Not Hispanic	0.81	3.65
LPV208	CASE	37	White, Not Hispanic	0.40	1.78
LPV209	CASE	28	White, Not Hispanic	0.13	3.70
LPV210	CONTROL	31	White, Not Hispanic	1.73	2.00
LPV211	CONTROL	21	White, Not Hispanic	0.90	4.73
LPV212	CASE	18	White, Not Hispanic	0.25	2.20
LPV213	CASE	27	White, Not Hispanic	0.14	4.87
LPV214	CASE	34	White, Not Hispanic	0.10	5.00
LPV215	CASE	34	White, Not Hispanic	0.10	3.29
LPV216	CONTROL	27	White, Not Hispanic	2.90	5.00
LPV217	CONTROL	31	White, Not Hispanic	3.98	3.67
LPV218	CONTROL	49	White, Not Hispanic	2.90	3.17
LPV219	CONTROL	30	White, Not Hispanic	3.17	3.40
LPV220	CONTROL	25	White, Not Hispanic	1.17	2.73
LVP221	CASE	28	White, Not Hispanic	0.73	0.80
LVP222	CASE	32	White, Not Hispanic	0.08	1.80
LVP223	CASE	23	White, Not Hispanic	0.21	5.00
LVP224	CASE	20	White, Not Hispanic	0.02	2.17
LVP225	CASE	28	Asian	0.79	4.23
LVP226	CASE	19	White, Not Hispanic	0.13	2.38
LVP227	CASE	28	White, Not Hispanic	0.07	2.34

Supplementary Figure S3. Raw Western Blot Data

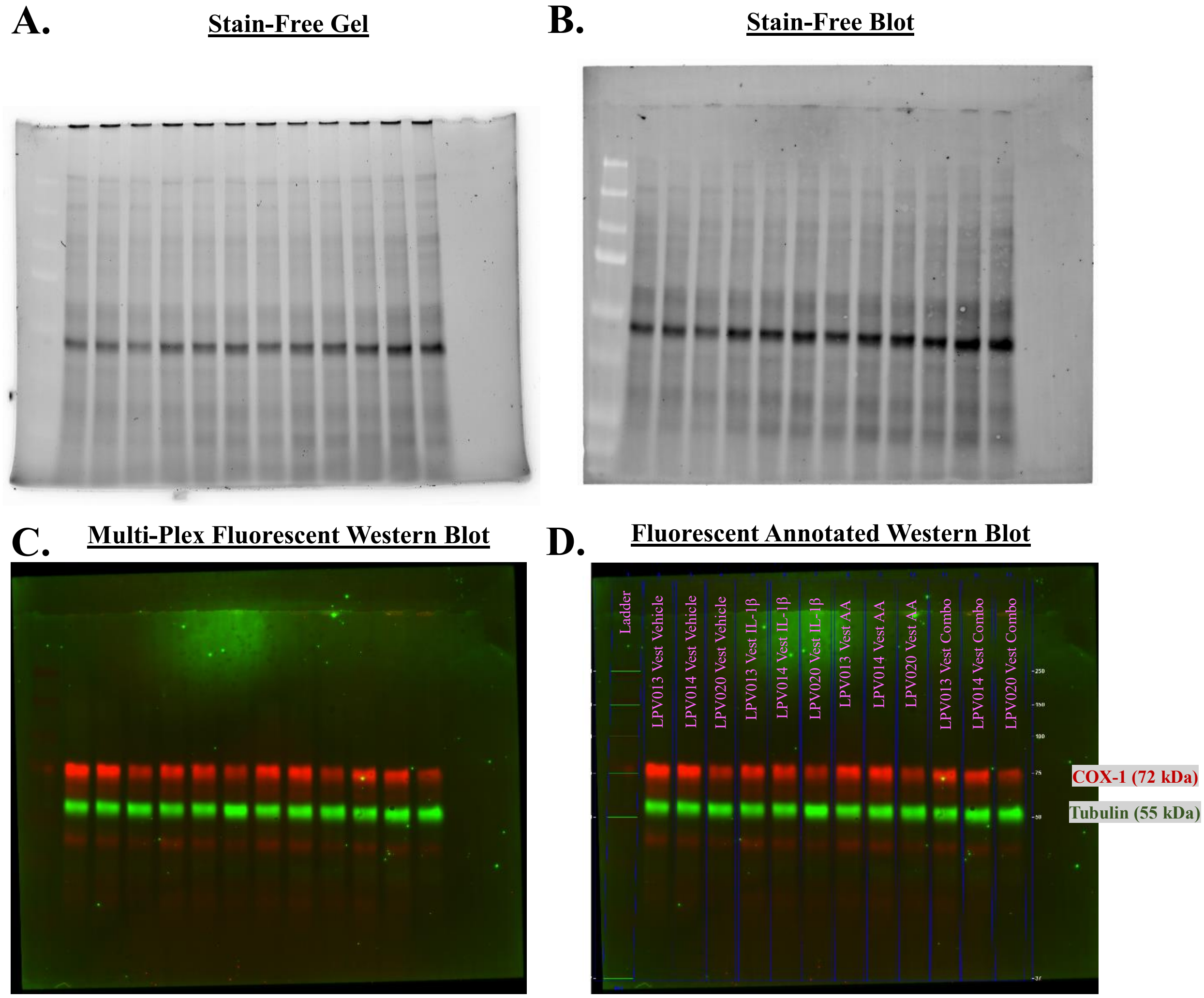

LPV Case Vest COX-1 Western blot from Fig 2E. (A) Stain-free gel image with each lane containing 10 µg of protein. Lysates were obtained from LPV vestibular fibroblast strains #013, #014, and #020. Cells were incubated with vehicle, IL-1β [500 pg/mL], arachidonic acid (AA) [1 µM], and a combination of both IL-1β [500 pg/mL] and AA [1 µM] (Combo) treatments for 48 hours prior to collecting protein lysates. After SDS-PAGE, UV trans illumination and 590/110 filter were used for excitation and emission to image the stain-free gel. (B) Stain-free blot image post transfer to a PVDF membrane. Image was acquired using UV trans illumination with a 590/110 filter for excitation/emission. The protein ladder on the stain-free blot was used for molecular weight analyses. (C) Multi-plex fluorescent western blot post antibody probing. Image was acquired utilizing Blue Epi Illumination and a 715/30 filter for the StarBright B700 (red) channel and Green Epi Illumination with a 602/50 Filter for the rhodamine (green) channel. Auto-optimal exposure times were used for all images. (D) Annotated Western blot analyzed using Image Lab 6.1 software (Bio-Rad). Lane number, sample name, molecular weights, and the proteins of interest are annotated. Analysis was performed automatically using Image Lab software and intensities of each band were normalized to levels of the housekeeping protein tubulin.

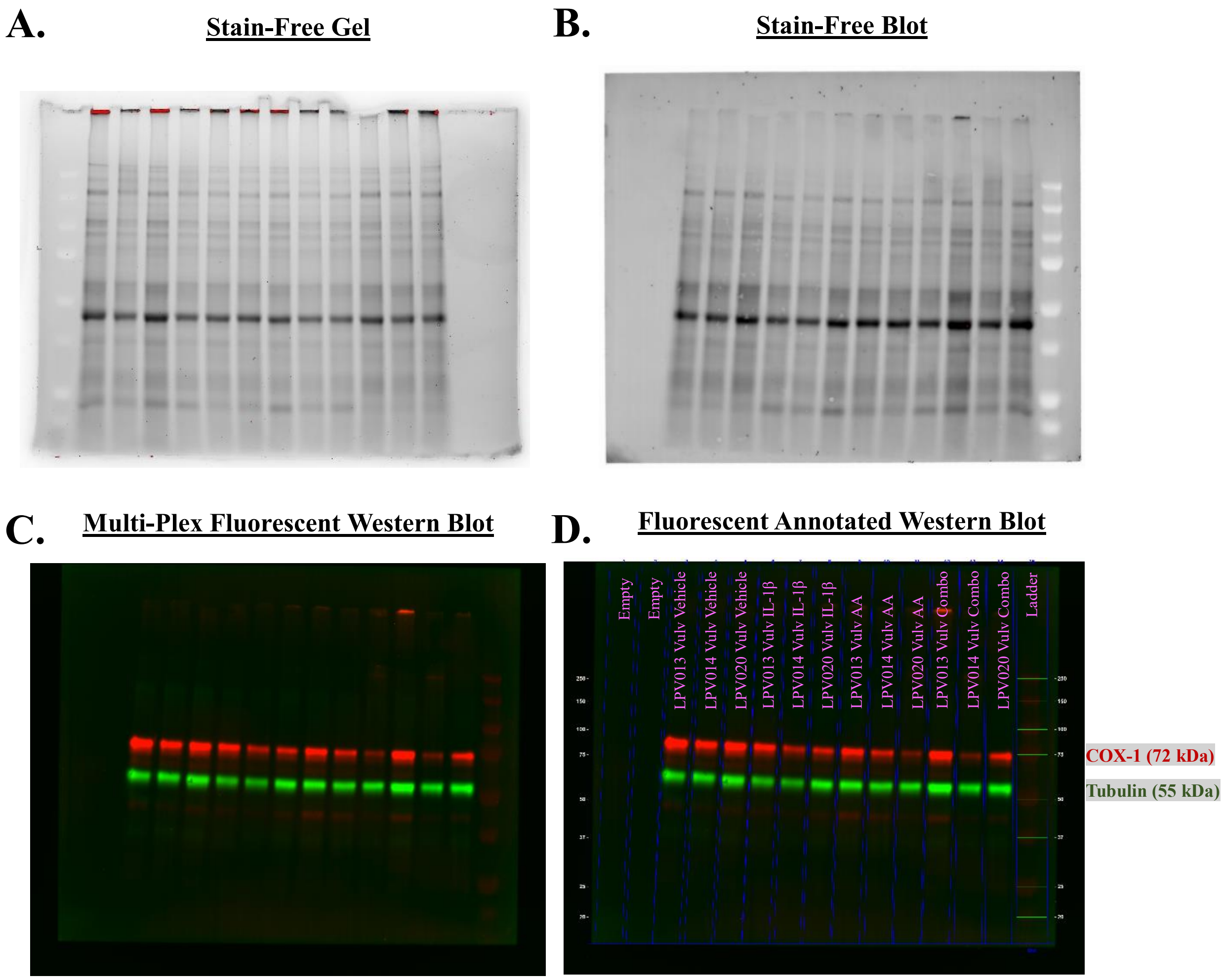

LPV Case Vulv COX-1 Western blot from Fig 2E. (A) Stain-free gel image with each lane containing 10 µg of protein. Lysates were obtained from LPV vulvar fibroblast strains #013, #014, and #020. Cells were incubated with vehicle, IL-1β [500 pg/mL], arachidonic acid (AA) [1 µM], and a combination of both IL-1β [500 pg/mL] and AA [1 µM] (Combo) treatments for 48 hours prior to collecting protein lysates. After SDS-PAGE, UV trans illumination and 590/110 filter were used for excitation and emission to image the stain-free gel. (B) Stain-free blot image post transfer to a PVDF membrane. Image was acquired using UV trans illumination with a 590/110 filter for excitation/emission. The protein ladder on the stain-free blot was used for molecular weight analyses. (C) Multi-plex fluorescent western blot post antibody probing. Image was acquired utilizing Blue Epi Illumination and a 715/30 filter for the StarBright B700 (red) channel and Green Epi Illumination with a 602/50 Filter for the rhodamine (green) channel. Auto-optimal exposure times were used for all images. (D) Annotated Western blot analyzed using Image Lab 6.1 software (Bio-Rad). Lane number, sample name, molecular weights, and the proteins of interest are annotated. Analysis was performed automatically using Image Lab software and intensities of each band were normalized to levels of the housekeeping protein tubulin.

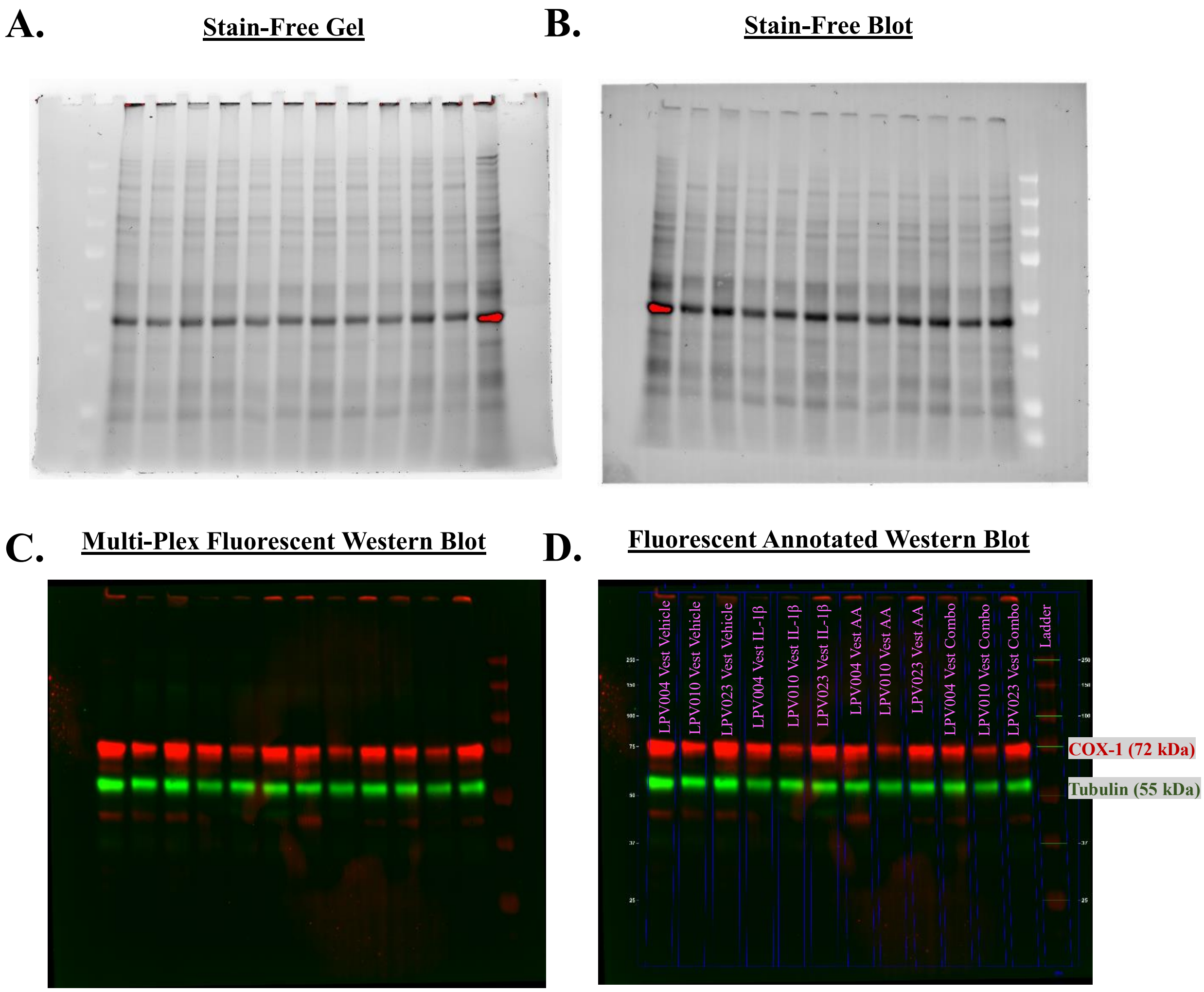

Control Vest COX-1 Western blot from Fig 2E. (A) Stain-free gel image with each lane containing 10 µg of protein. Lysates were obtained from control vestibular fibroblast strains #004, #010, and #023. Cells were incubated with vehicle, IL-1β [500 pg/mL], arachidonic acid (AA) [1 µM], and a combination of both IL-1β [500 pg/mL] and AA [1 µM] (Combo) treatments for 48 hours prior to collecting protein lysates. After SDS-PAGE, UV trans illumination and 590/110 filter were used for excitation and emission to image the stain-free gel. (B) Stain-free blot image post transfer to a PVDF membrane. Image was acquired using UV trans illumination with a 590/110 filter for excitation/emission. The protein ladder on the stain-free blot was used for molecular weight analyses. (C) Multi-plex fluorescent western blot post antibody probing. Image was acquired utilizing Blue Epi Illumination and a 715/30 filter for the StarBright B700 (red) channel and Green Epi Illumination with a 602/50 Filter for the rhodamine (green) channel. Auto-optimal exposure times were used for all images. (D) Annotated Western blot analyzed using Image Lab 6.1 software (Bio-Rad). Lane number, sample name, molecular weights, and the proteins of interest are annotated. Analysis was performed automatically using Image Lab software and intensities of each band were normalized to levels of the housekeeping protein tubulin.

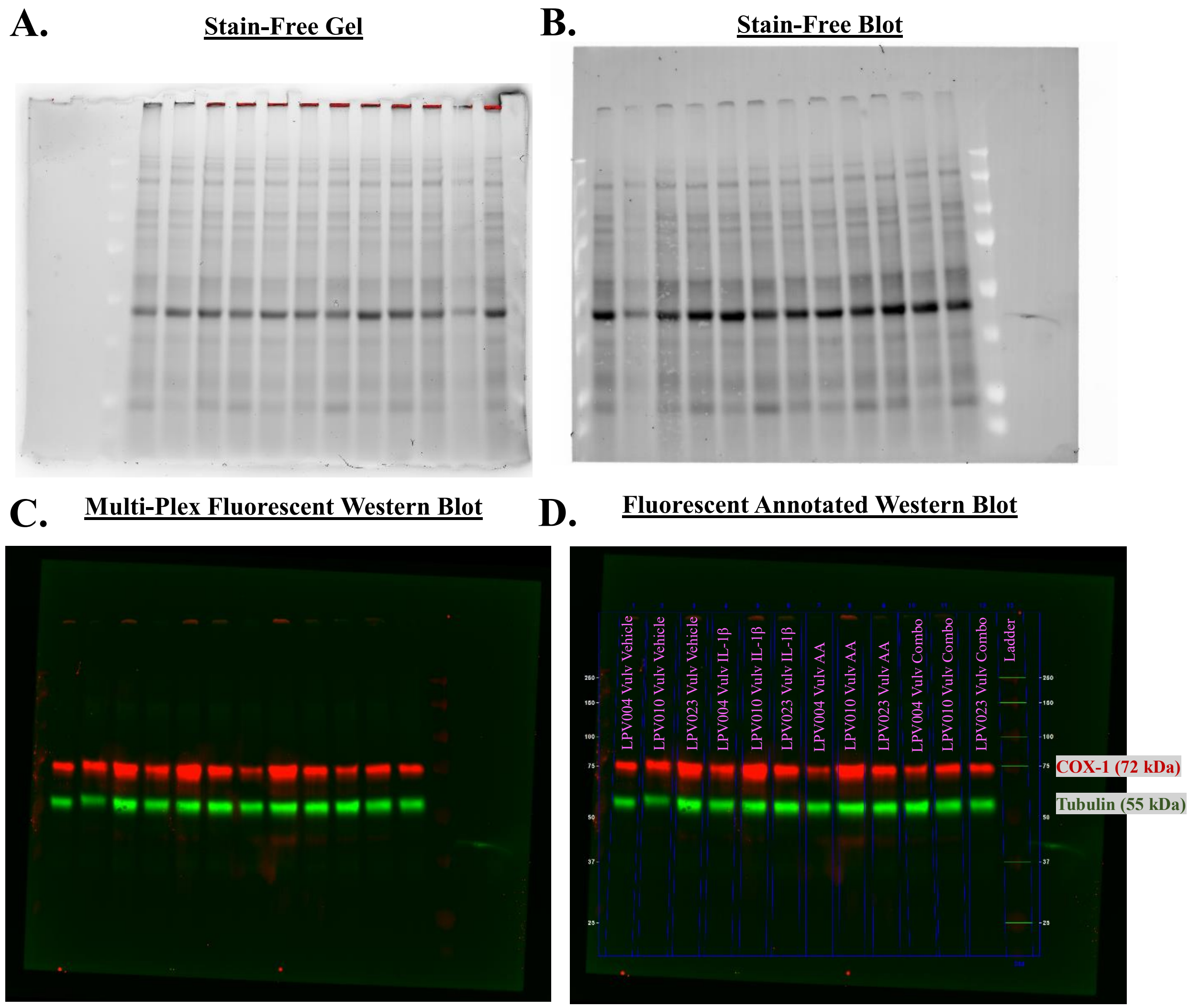

Control Vulv COX-1 Western blot from Fig 2E. (A) Stain-free gel image with each lane containing 10 µg of protein. Lysates were obtained from control vulvar fibroblast strains #004, #010 and #023. Cells were incubated with vehicle, IL-1β [500 pg/mL], arachidonic acid (AA) [1 µM], and a combination of both IL-1β [500 pg/mL] and AA [1 µM] (Combo) treatments for 48 hours prior to collecting protein lysates. After SDS-PAGE, UV trans illumination and 590/110 filter were used for excitation and emission to image the stain-free gel. (B) Stain-free blot image post transfer to a PVDF membrane. Image was acquired using UV trans illumination with a 590/110 filter for excitation/emission. The protein ladder on the stain-free blot was used for molecular weight analyses. (C) Multi-plex fluorescent western blot post antibody probing. Image was acquired utilizing Blue Epi Illumination and a 715/30 filter for the StarBright B700 (red) channel and Green Epi Illumination with a 602/50 Filter for the rhodamine (green) channel. Auto-optimal exposure times were used for all images. (D) Annotated Western blot analyzed using Image Lab 6.1 software (Bio-Rad). Lane number, sample name, molecular weights, and the proteins of interest are annotated. Analysis was performed automatically using Image Lab software and intensities of each band were normalized to levels of the housekeeping protein tubulin.

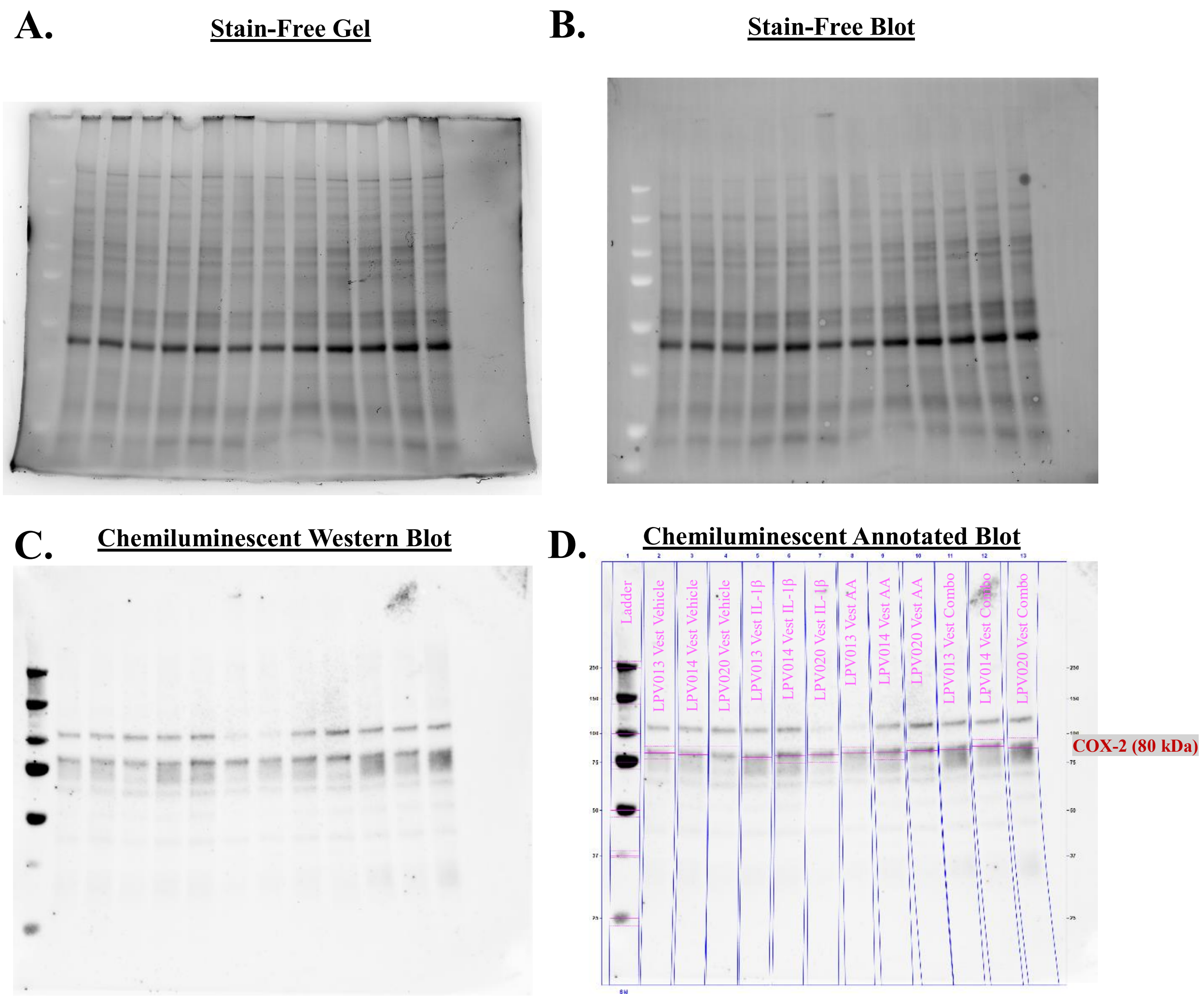

LPV Case Vest COX-2 Western blot from Fig 2F. (A) Stain-free gel image with each lane containing 10 µg of protein. Lysates were obtained from LPV vestibular fibroblast strains #013, #014, and #020. Cells were incubated with vehicle, IL-1β [500 pg/mL], arachidonic acid (AA) [1 µM], and a combination of both IL-1β [500 pg/mL] and AA [1 µM] (Combo) treatments for 48 hours prior to collecting protein lysates. After SDS-PAGE, UV trans illumination and 590/110 filter were used for excitation and emission to image the stain-free gel. (B) Stain-free blot image post transfer to a PVDF membrane. Image was acquired using UV trans illumination with a 590/110 filter for excitation/emission. The protein ladder on the stain-free blot was used for molecular weight analyses. (C) Chemiluminescence Western blot post probing with primary and secondary antibodies. Image was acquired using a 647SP filter using auto-optimal exposures. (D) Annotated blot image analyzed using Image Lab 6.1 software (Bio-Rad). Lane number, sample name, molecular weights, and the protein of interest are annotated. The most intense band at 80 kDa was analyzed as COX-2, as this was its expected size. The second non-specific band at 110 kDa was excluded from the analysis. Image Lab software was used to perform all analyses and intensities of each band were normalized to total protein levels of the stain-free blot.

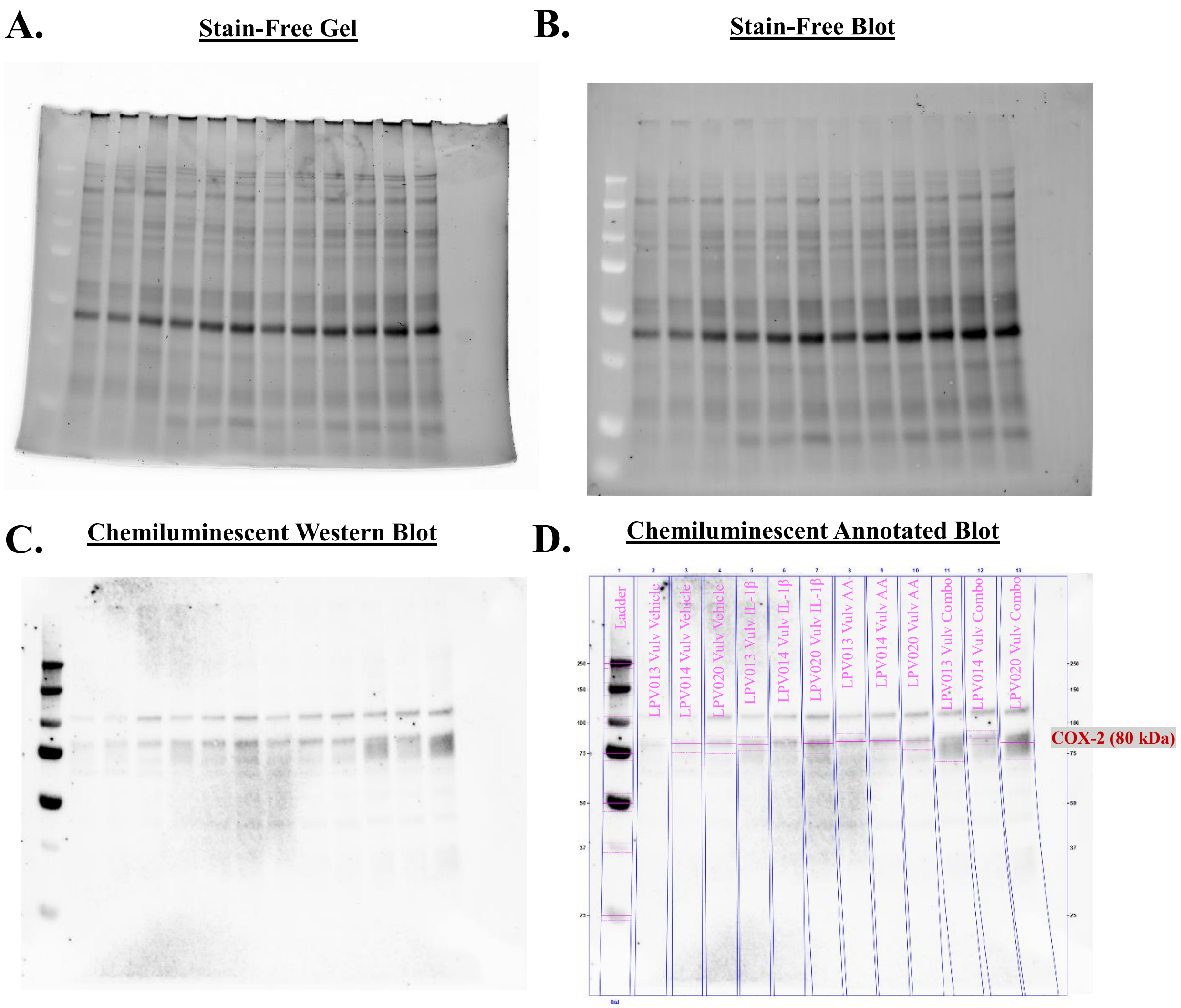

LPV Case Vulv COX-2 Western blot from Fig 2F. (A) Stain-free gel image with each lane containing 10 µg of protein. Lysates were obtained from LPV vulvar fibroblast strains #013, #014, and #020. Cells were incubated with vehicle, IL-1β [500 pg/mL], arachidonic acid (AA) [1 µM], and a combination of both IL-1β [500 pg/mL] and AA [1 µM] (Combo) treatments for 48 hours prior to collecting protein lysates. After SDS-PAGE, UV trans illumination and 590/110 filter were used for excitation and emission to image the stain-free gel. (B) Stain-free blot image post transfer to a PVDF membrane. Image was acquired using UV trans illumination with a 590/110 filter for excitation/emission. The protein ladder on the stain-free blot was used for molecular weight analyses. (C) Chemiluminescence Western blot post probing with primary and secondary antibodies. Image was acquired using a 647SP filter using auto-optimal exposures. (D) Annotated blot image analyzed using Image Lab 6.1 software (Bio-Rad). Lane number, sample name, molecular weights, and the protein of interest are annotated. The most intense band at 80 kDa was analyzed as COX-2, as this was its expected size. The second non-specific band at 110 kDa was excluded from the analysis. Image Lab software was used to perform all analyses and intensities of each band were normalized to total protein levels of the stain-free blot.

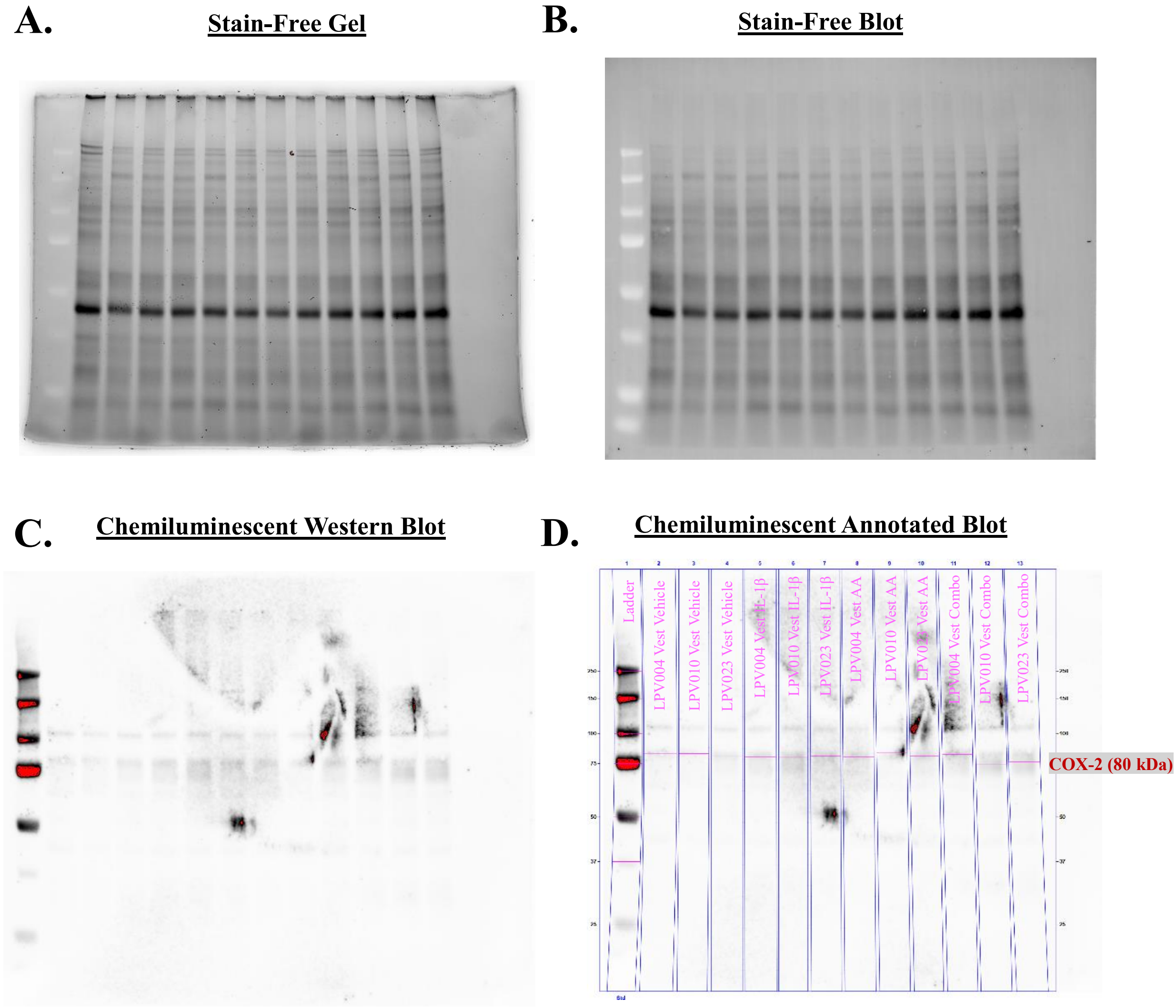

Control Vest COX-2 Western blot from Fig 2F. (A) Stain-free gel image with each lane containing 10 µg of protein. Lysates were obtained from control vestibular fibroblast strains #004, #010, and #023. Cells were incubated with vehicle, IL-1β [500 pg/mL], arachidonic acid (AA) [1 µM], and a combination of both IL-1β [500 pg/mL] and AA [1 µM] (Combo) treatments for 48 hours prior to collecting protein lysates. After SDS-PAGE, UV trans illumination and 590/110 filter were used for excitation and emission to image the stain-free gel. (B) Stain-free blot image post transfer to a PVDF membrane. Image was acquired using UV trans illumination with a 590/110 filter for excitation/emission. The protein ladder on the stain-free blot was used for molecular weight analyses. (C) Chemiluminescence Western blot post probing with primary and secondary antibodies. Image was acquired using a 647SP filter using auto-optimal exposures. (D) Annotated blot image analyzed using Image Lab 6.1 software (Bio-Rad). Lane number, sample name, molecular weights, and the protein of interest are annotated. The most intense band at 80 kDa was analyzed as COX-2, as this was its expected size. The second non-specific band at 110 kDa was excluded from the analysis. Image Lab software was used to perform all analyses and intensities of each band were normalized to total protein levels of the stain-free blot.

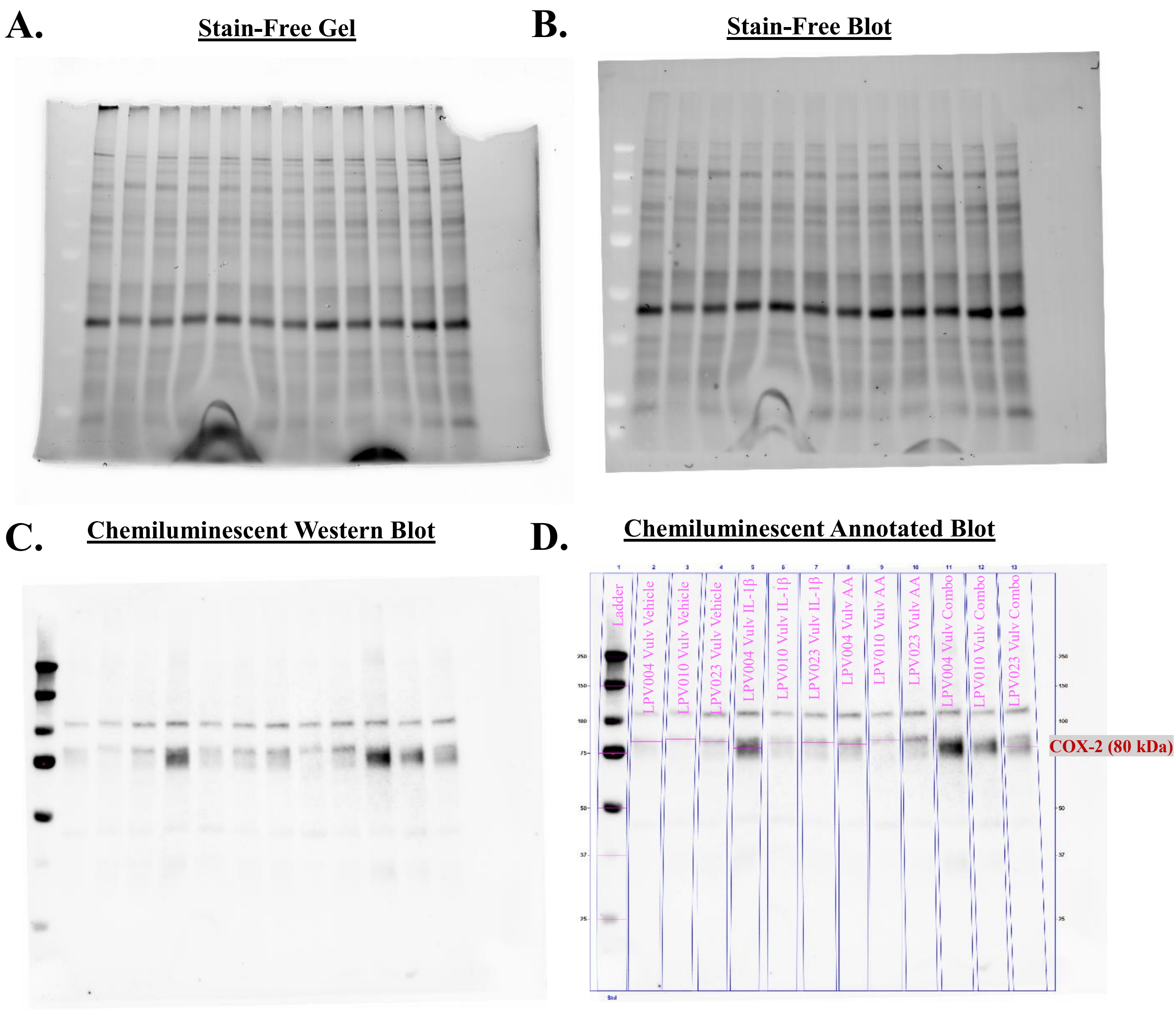

Control Vulv COX-2 Western blot from Fig 2F. (A) Stain-free gel image with each lane containing 10 µg of protein. Lysates were obtained from control vulvar fibroblast strains #004, #010, and #023. Cells were incubated with vehicle, IL-1β [500 pg/mL], arachidonic acid (AA) [1 µM], and a combination of both IL-1β [500 pg/mL] and AA [1 µM] (Combo) treatments for 48 hours prior to collecting protein lysates. After SDS-PAGE, UV trans illumination and 590/110 filter were used for excitation and emission to image the stain-free gel. (B) Stain-free blot image post transfer to a PVDF membrane. Image was acquired using UV trans illumination with a 590/110 filter for excitation/emission. The protein ladder on the stain-free blot was used for molecular weight analyses. (C) Chemiluminescence Western blot post probing with primary and secondary antibodies. Image was acquired using a 647SP filter using auto-optimal exposures. (D) Annotated blot image analyzed using Image Lab 6.1 software (Bio-Rad). Lane number, sample name, molecular weights, and the protein of interest are annotated. The most intense band at 80 kDa was analyzed as COX-2, as this was its expected size. The second non-specific band at 110 kDa was excluded from the analysis. Image Lab software was used to perform all analyses and intensities of each band were normalized to total protein levels of the stain-free blot.

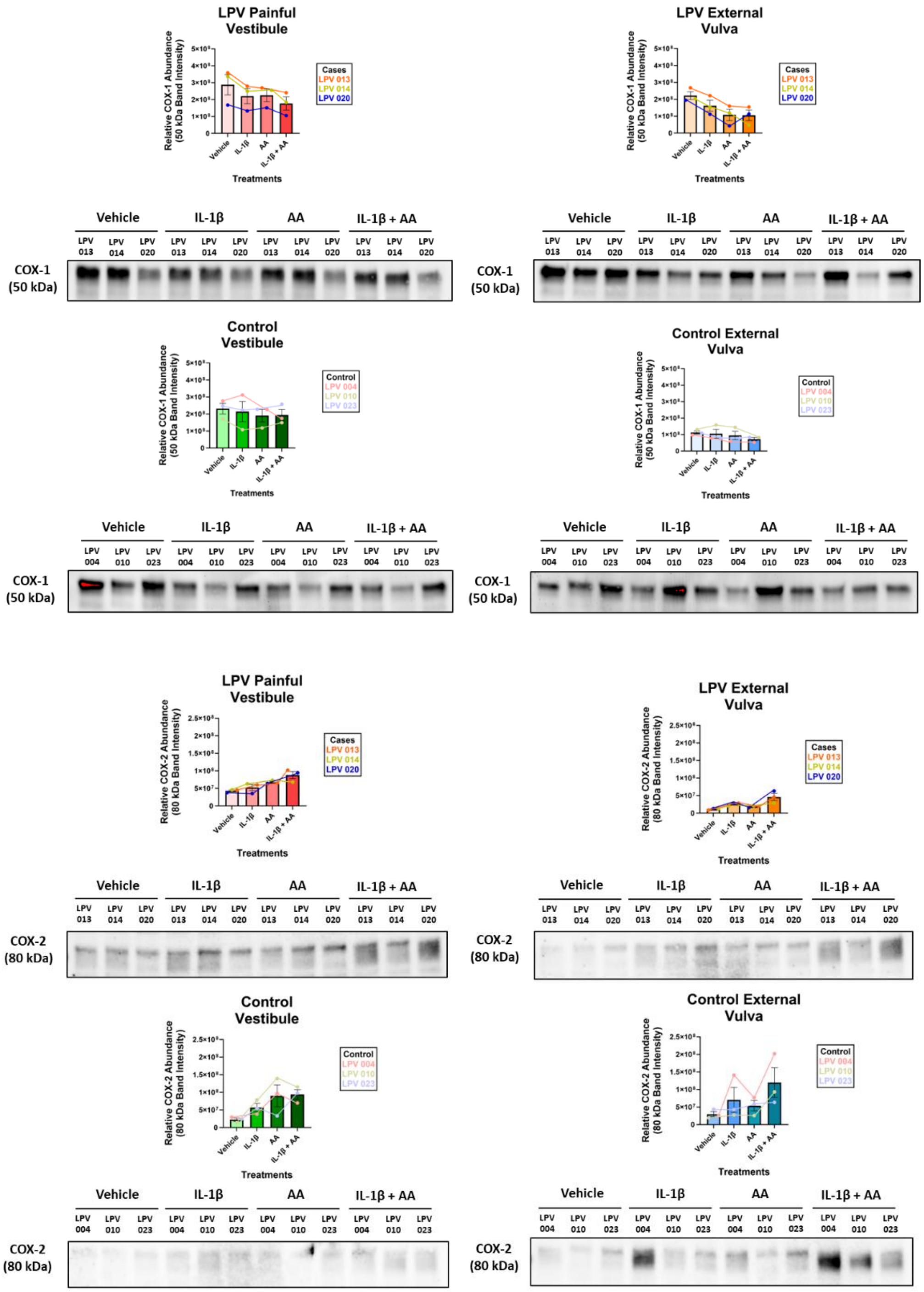

Individual quantifications for each western blot show trends of decreased COX-1 expression upon additional AA and IL-1β inflammatory stimuli and increased COX-2 expression. Data was analyzed using Image Lab 6.1 software (Bio-Rad) with COX-1 abundance normalized to the housekeeping protein tubulin and COX-2 abundance normalized to total protein levels of the stain-free blot. Different normalization methods were used since COX-1 was probed with fluorescent secondary antibodies that allowed for multiplexing whereas COX-2 was probed using chemiluminescence methods. Data for each of the 4 sampling sites were analyzed collectively in Figures 2C and 2D.

Supplementary Figure S4. Lipoxygenase Activity Assay Time Course Data

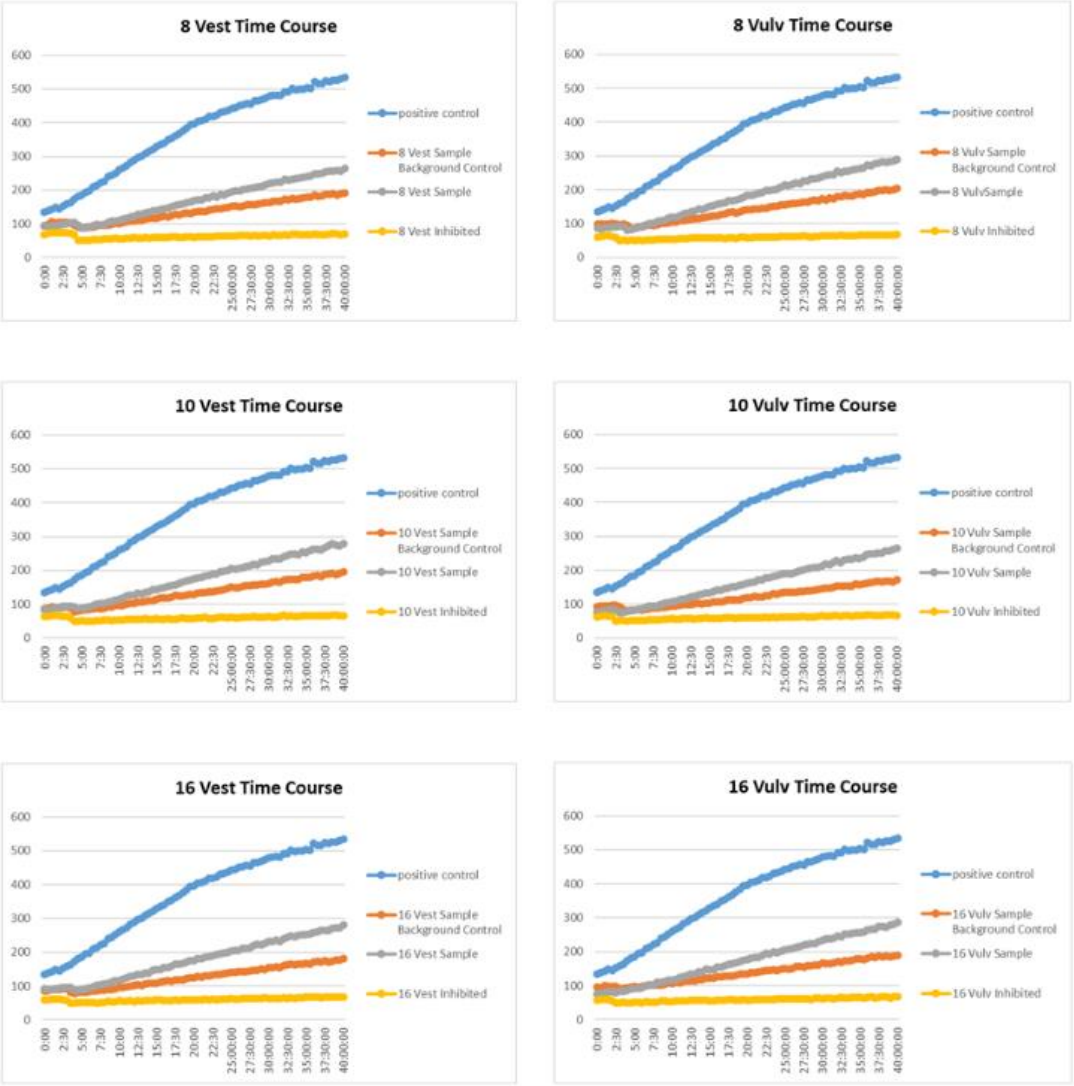

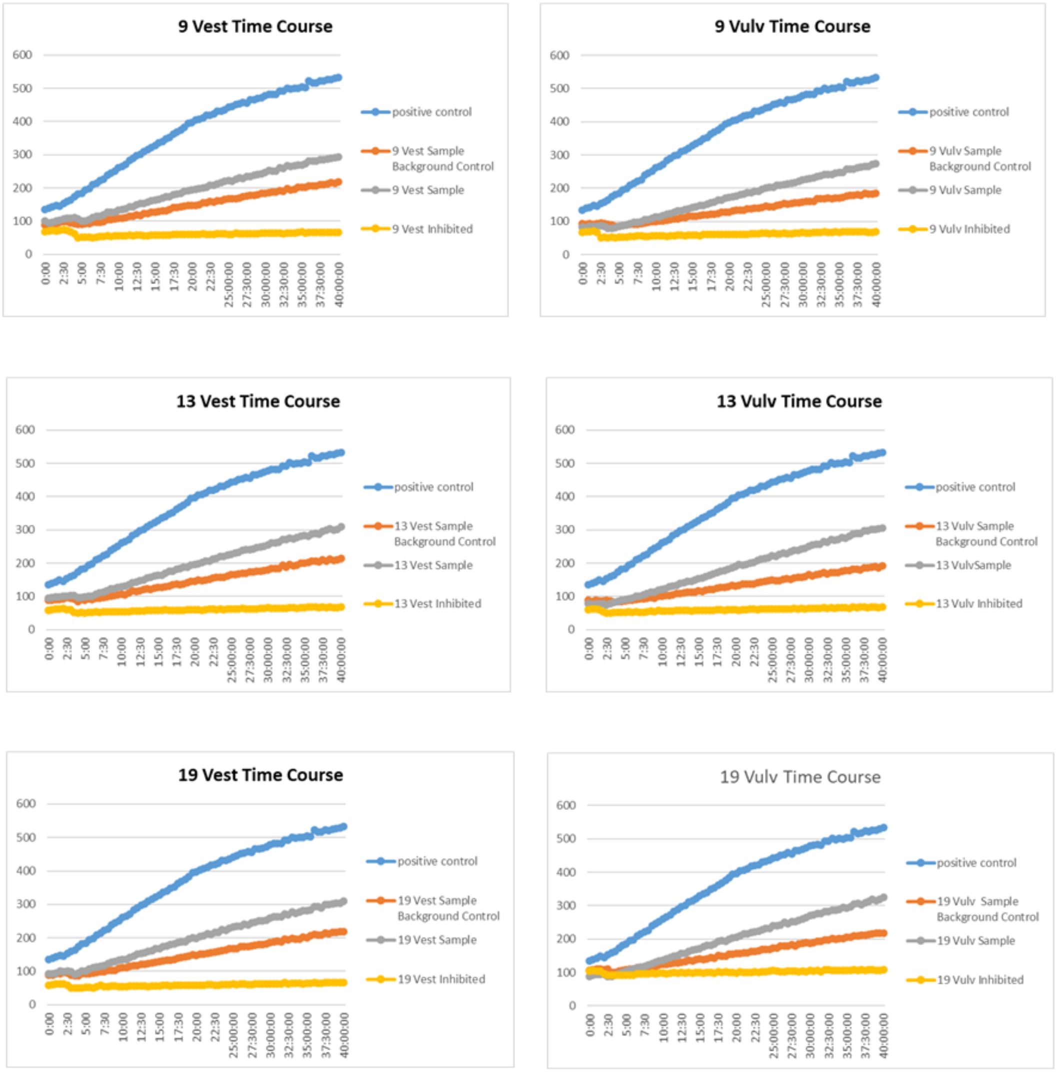

Supplementary Figure S4. Lipoxygenase Activity Assay (Sigma-Aldrich #MAK363) Time Course Data. Fibroblast lysates were incubated with a kit-provided LOX substrate and fluorescent probe to monitor epoxidation by lipoxygenase enzymes over time. Fluorescent readings (Ex 500 nm/ Em 536 nm) were recorded every 30 seconds for 40 minutes using a kinetic FlexStation Multi-Mode Microplate Reader (Molecular Devices). A 5-lipoxygenase enzyme was used as a positive control, and a LOX inhibitor was included to demonstrate specificity of the reactions to LOX enzymes. For every sample, a background control reaction that contained all of the components except the LOX substrate was also prepared to account for nonspecific background fluorescence. All reactions reached above background levels and were used to quantify LOX activity in Figure 3. n=3 per group.

Supplementary Figure S5. All Individual Lipid Bar Plots

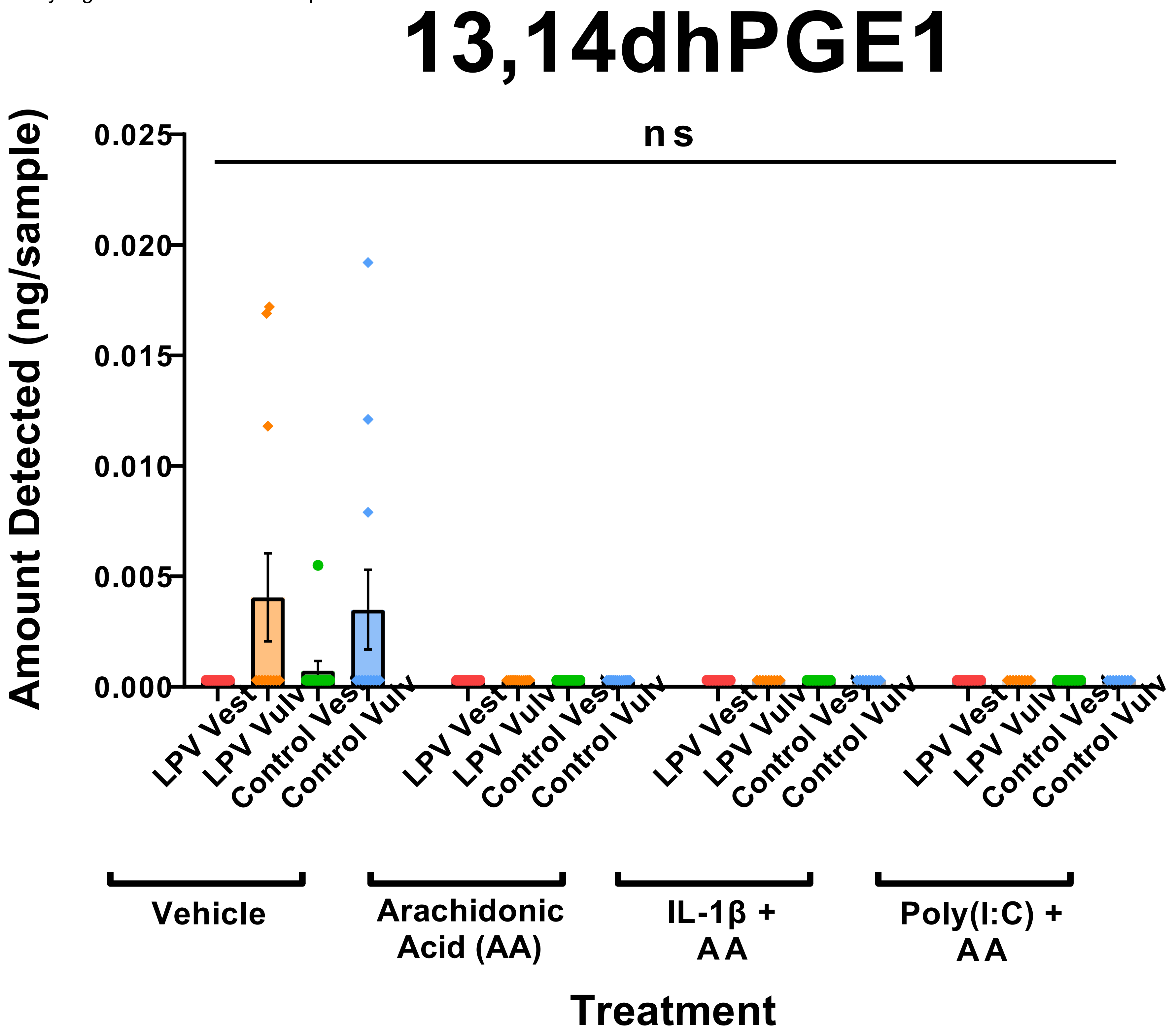

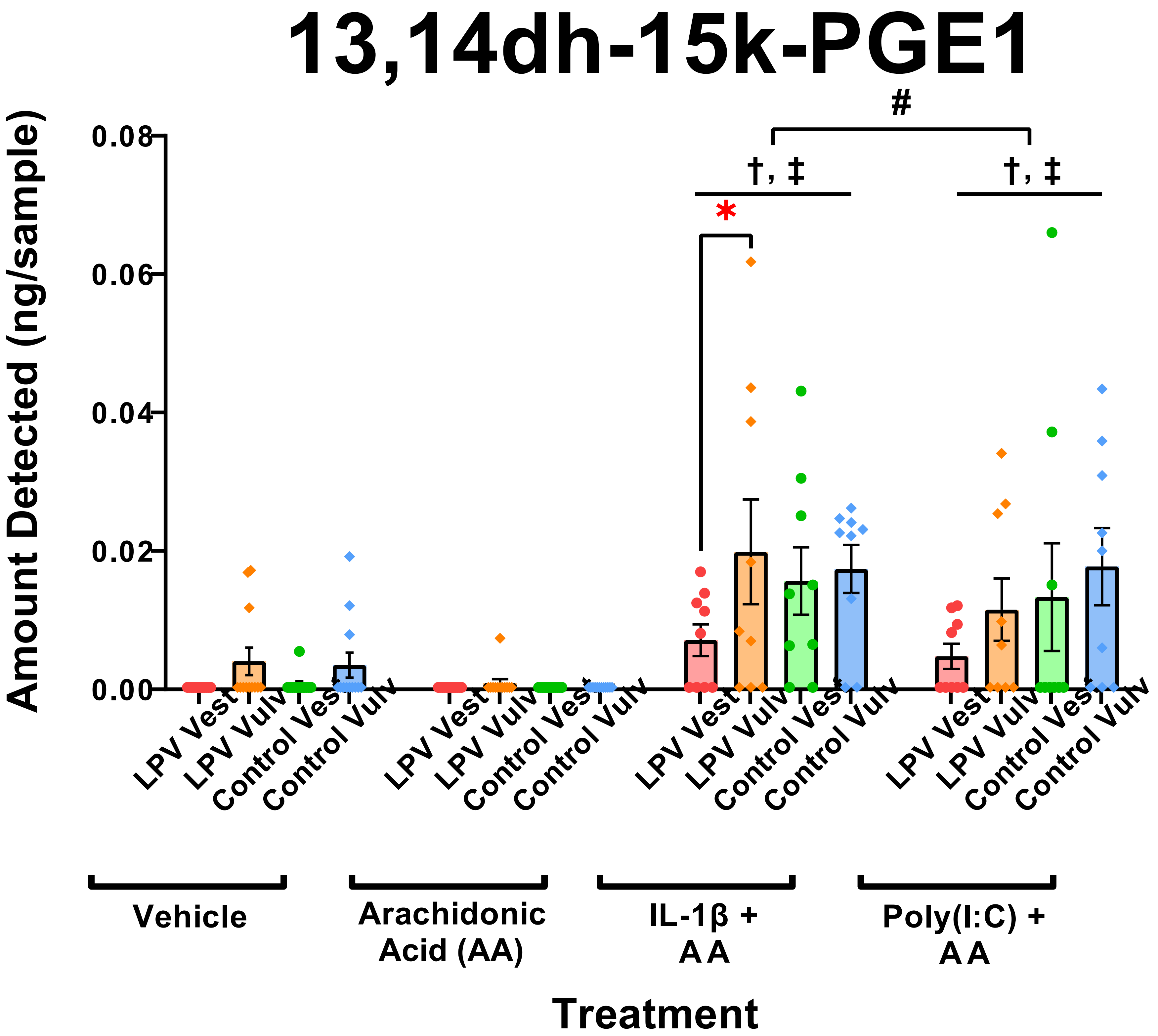
13,14dh-15k-PGE1
Amount Detected (ng/sample)
0.08
0.06
0.04
0.02
0.00
#
†, ‡
†, ‡
*
LPV Vest
LPV Vulv
Control Vest
Control Vulv
Vehicle
Arachidonic Acid (AA)
IL-1β + AA
Poly(I:C) + AA
Treatment

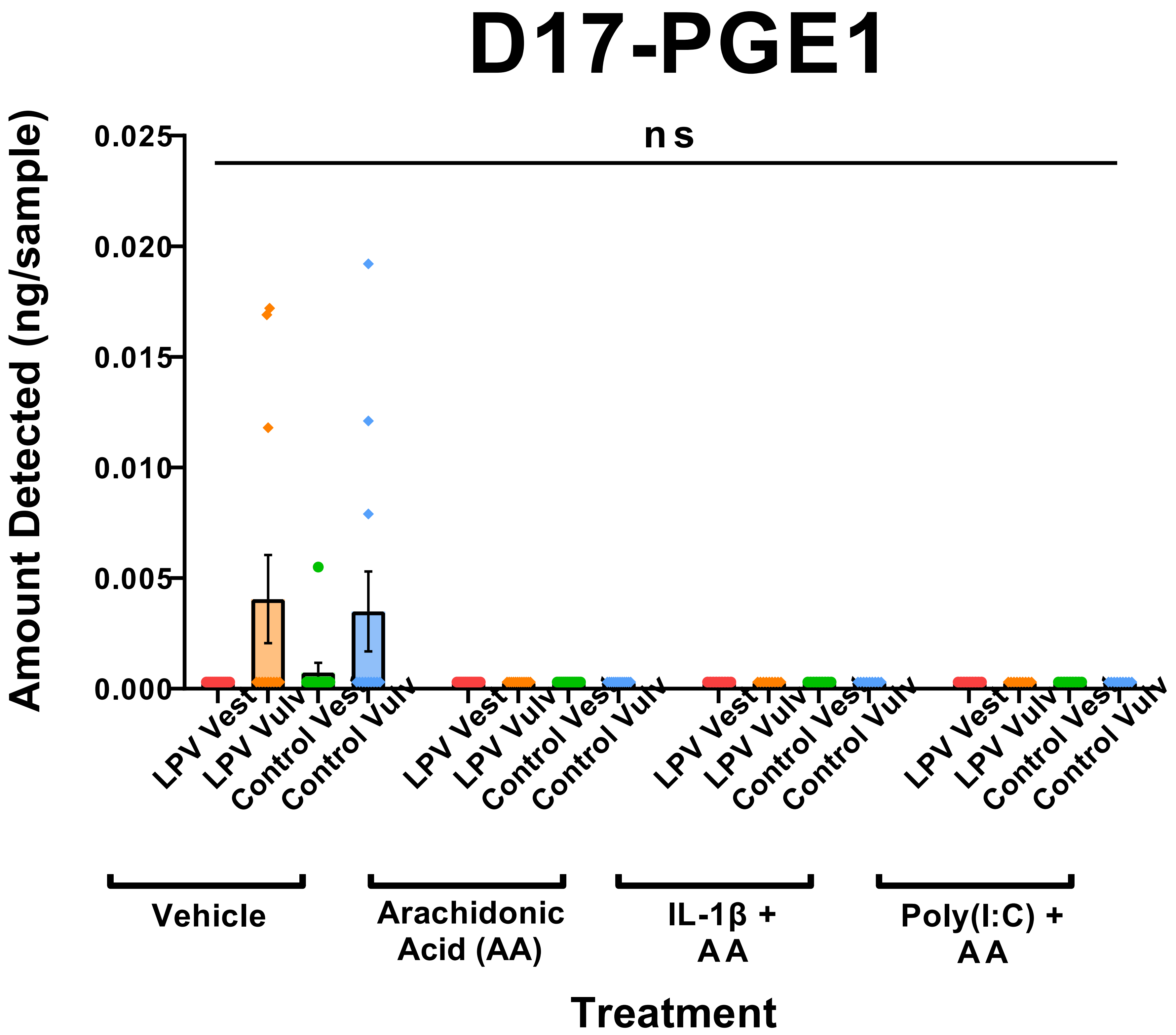
D17-PGE1
Amount Detected (ng/sample)
0.025
0.020
0.015
0.010
0.005
0.000
ns
LPV Vest
LPV Vulv
Control Vest
Control Vulv
Vehicle
Arachidonic Acid (AA)
IL-1β + AA
Poly(I:C) + AA
Treatment

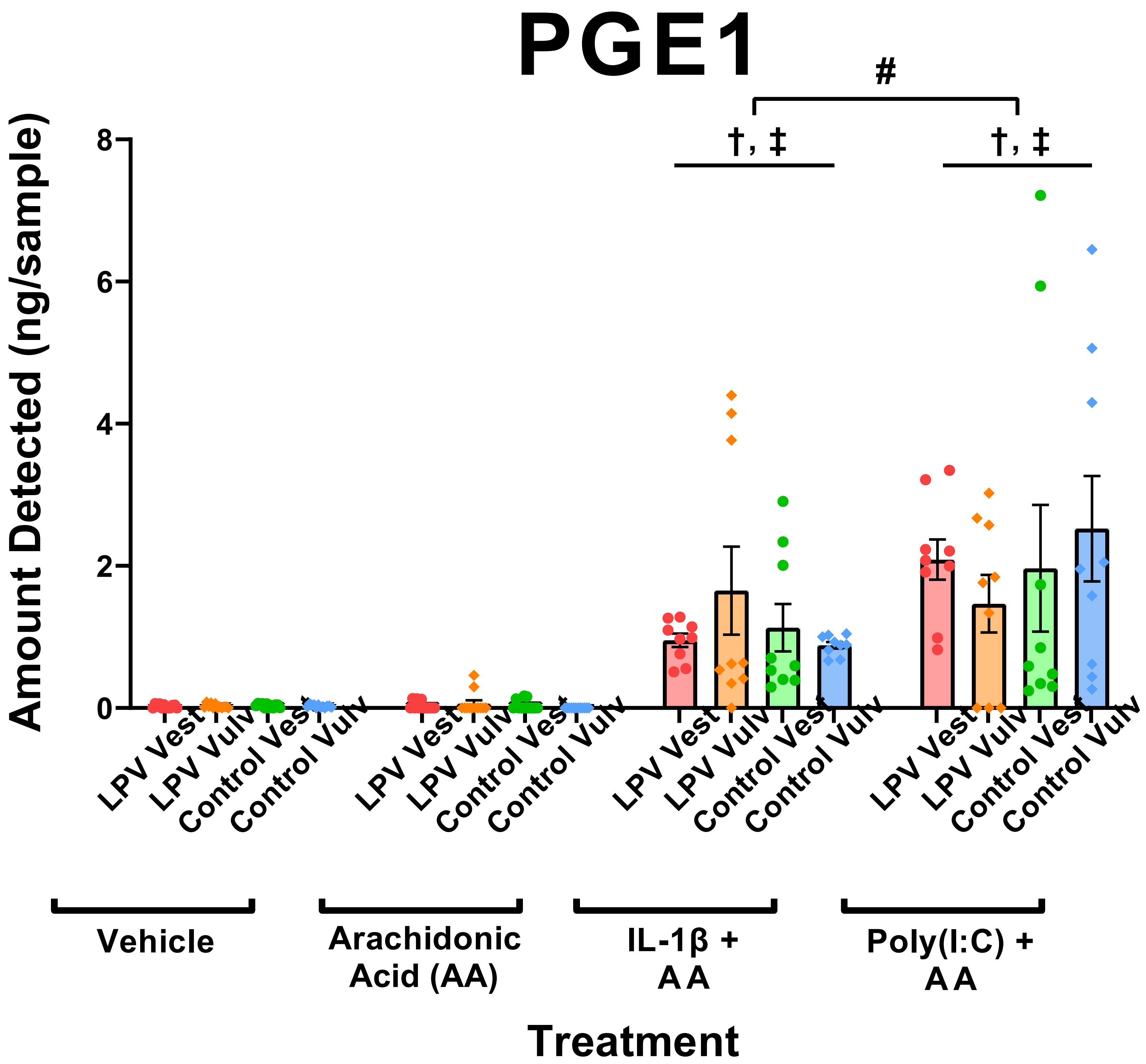
PGE1
#
†, ‡
†, ‡
Amount Detected (ng/sample)
8
6
4
2
0
LPV Vest
LPV Vulv
Control Vest
Control Vulv
Vehicle
Arachidonic Acid (AA)
IL-1β + AA
Poly(I:C) + AA
Treatment

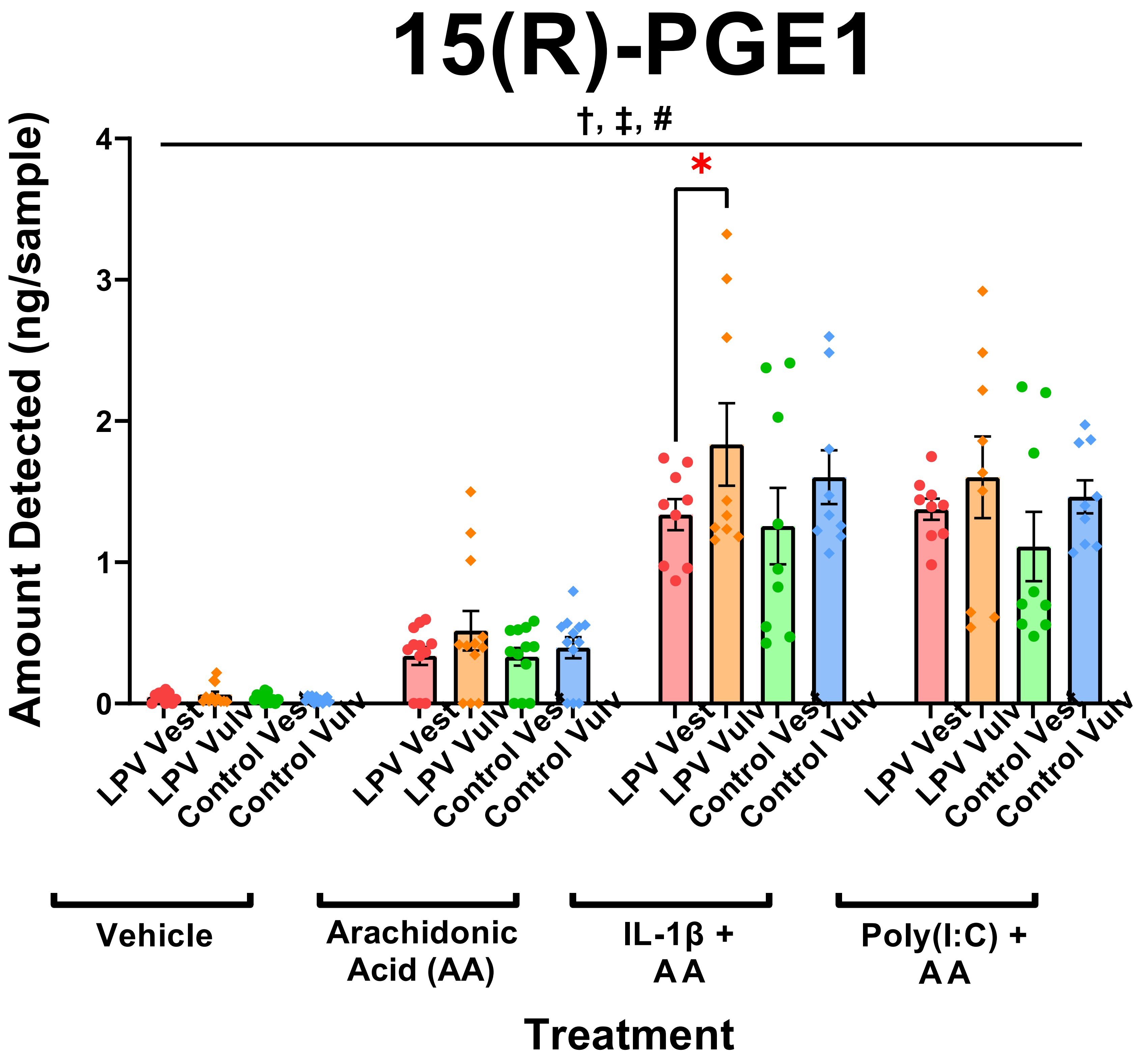

15(R)-PGE1
†, ‡, #
*
Amount Detected (ng/sample)
0
1
2
3
4
LPV Vest
LPV Vulv
Control Vest
Control Vulv
Vehicle
Arachidonic Acid (AA)
IL-1β + AA
Poly(I:C) + AA
Treatment

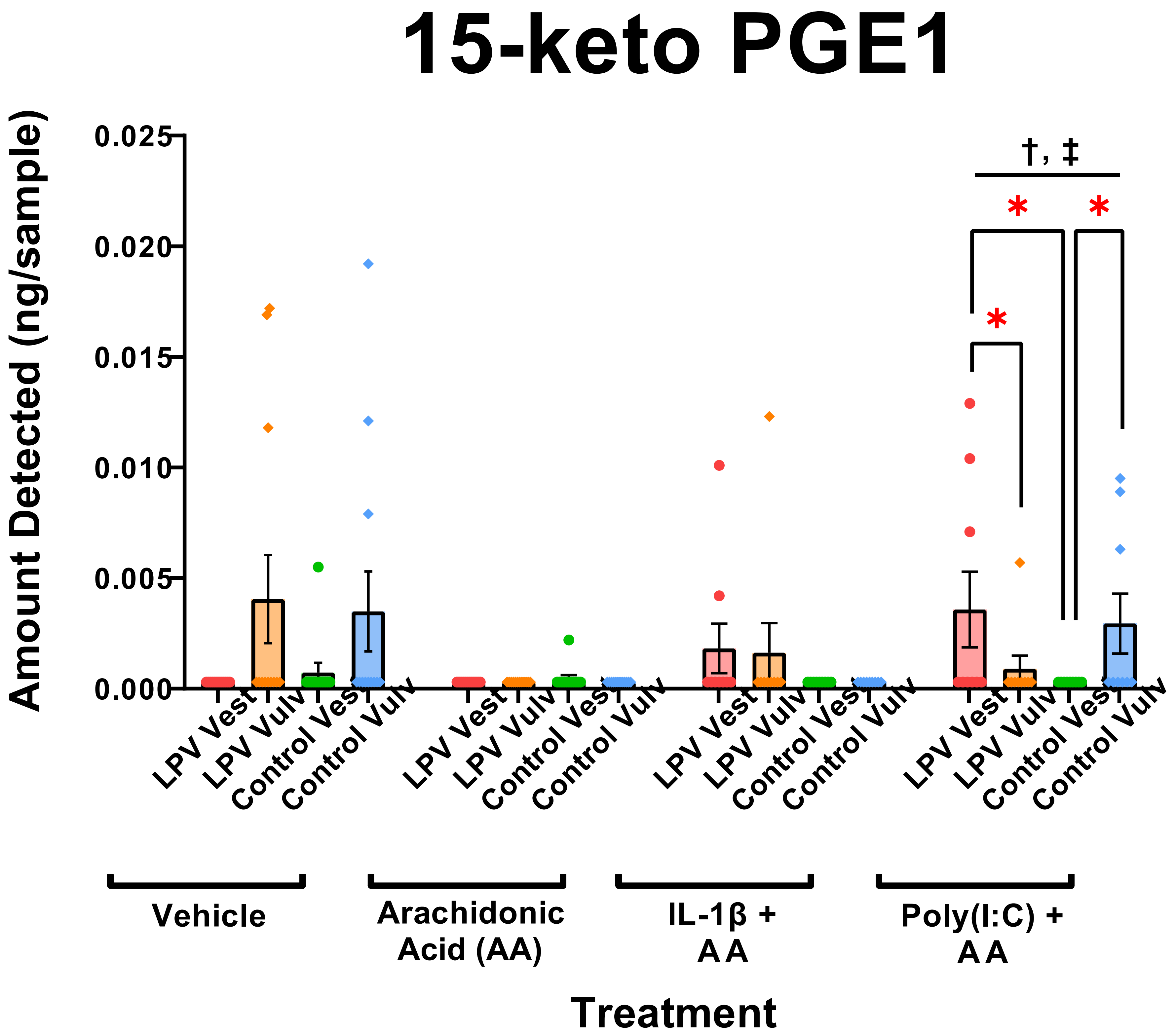

15-keto PGE1
Amount Detected (ng/sample)
0.025
0.020
0.015
0.010
0.005
0.000
†, ‡
*
*
*
LPV Vest
LPV Vulv
Control Vest
Control Vulv
Vehicle
Arachidonic Acid (AA)
IL-1β + AA
Poly(I:C) + AA
Treatment

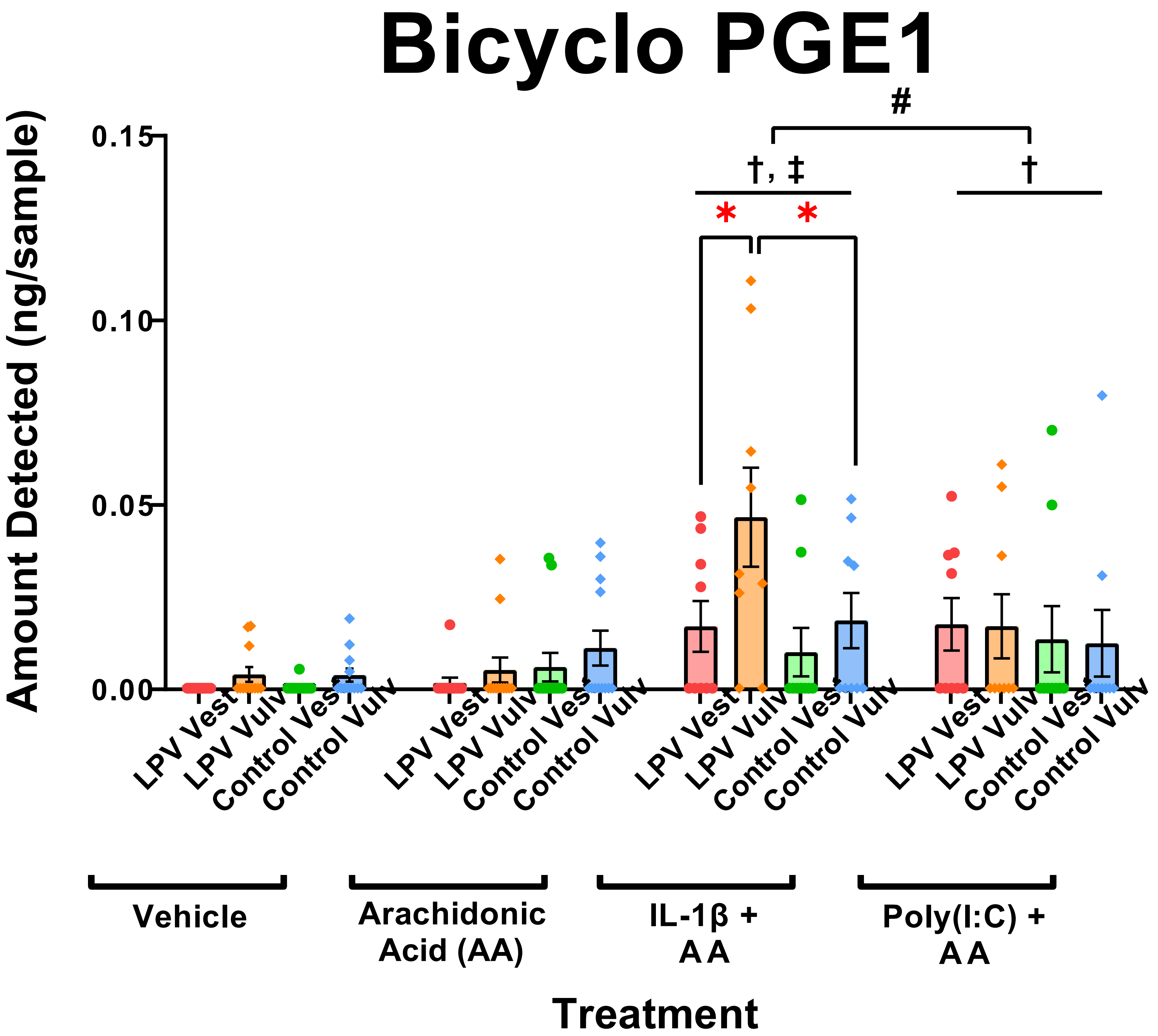
Bicyclo PGE1
Amount Detected (ng/sample)
0.15
0.10
0.05
0.00
#
†, ‡
†
*
*
LPV Vest
LPV Vulv
Control Vest
Control Vulv
Vehicle
Arachidonic Acid (AA)
IL-1β + AA
Poly(I:C) + AA
Treatment

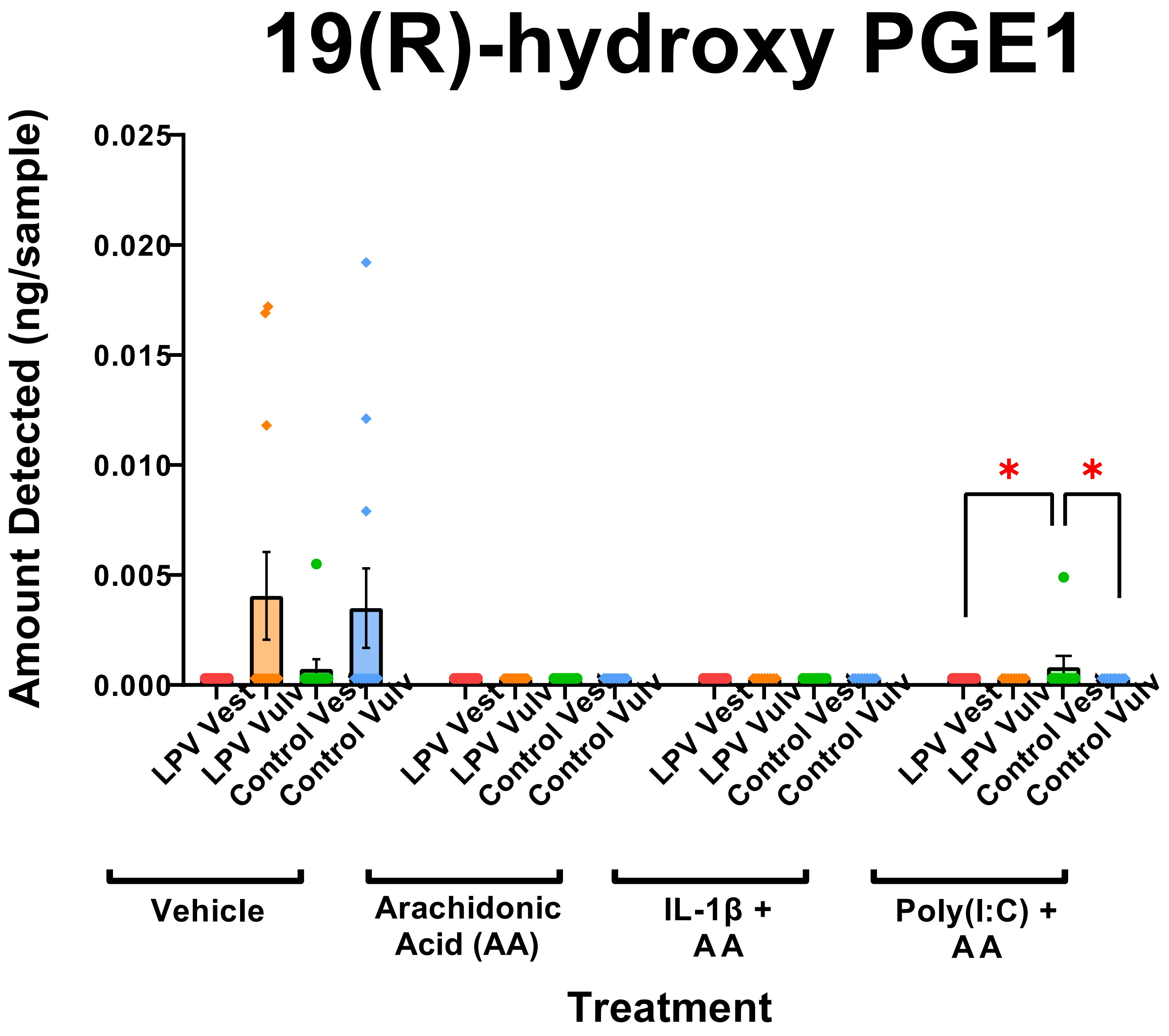
19(R)-hydroxy PGE1
Amount Detected (ng/sample)
0.025
0.020
0.015
0.010
0.005
0.000
*
*
LPV Vest
LPV Vulv
Control Vest
Control Vulv
Vehicle
Arachidonic Acid (AA)
IL-1β + AA
Poly(I:C) + AA
Treatment

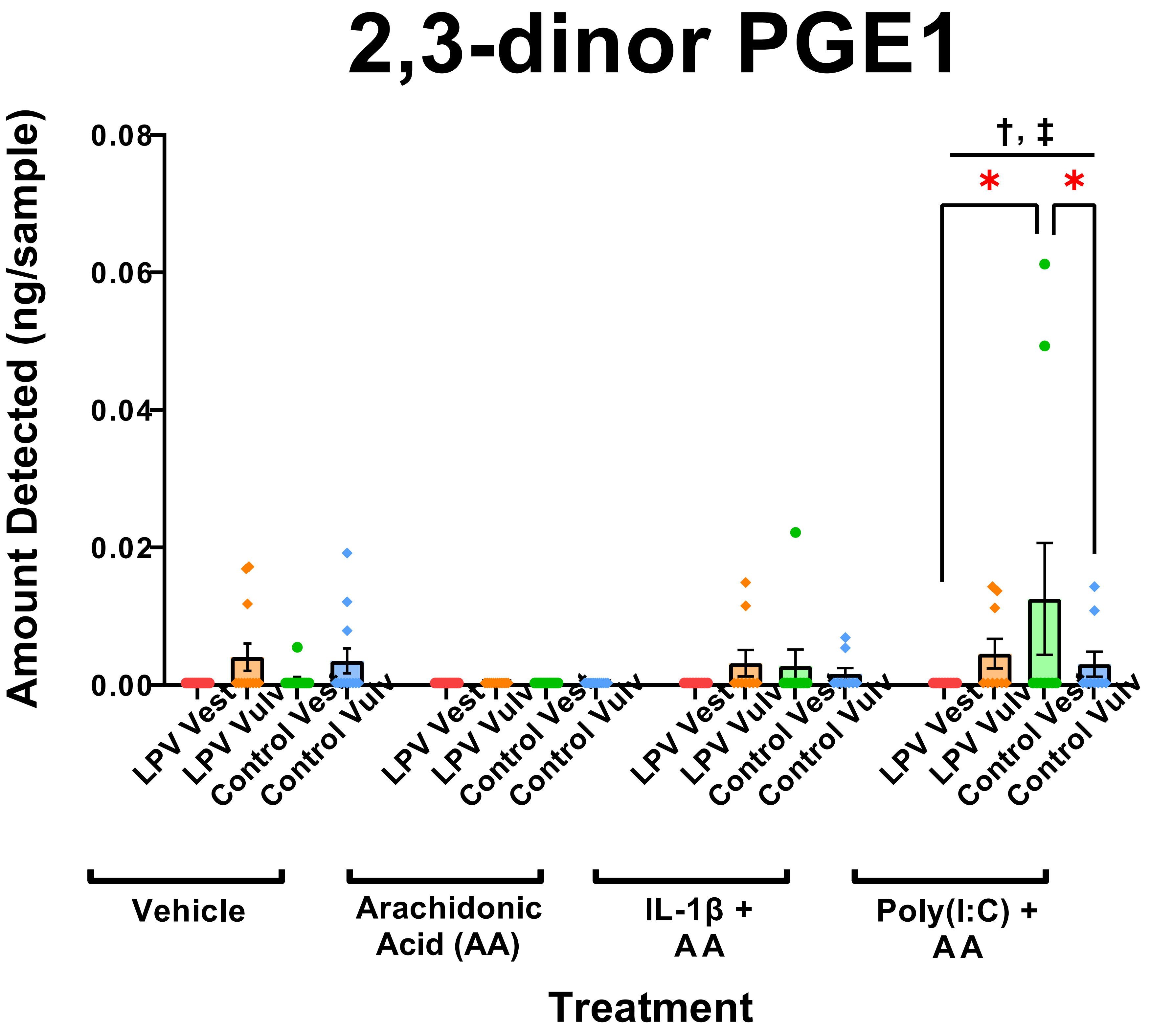
2,3-dinor PGE1
Amount Detected (ng/sample)
0.08
0.06
0.04
0.02
0.00
†, ‡
*
*
LPV Vest
LPV Vulv
Control Vest
Control Vulv
Vehicle
Arachidonic Acid (AA)
IL-1β + AA
Poly(I:C) + AA
Treatment

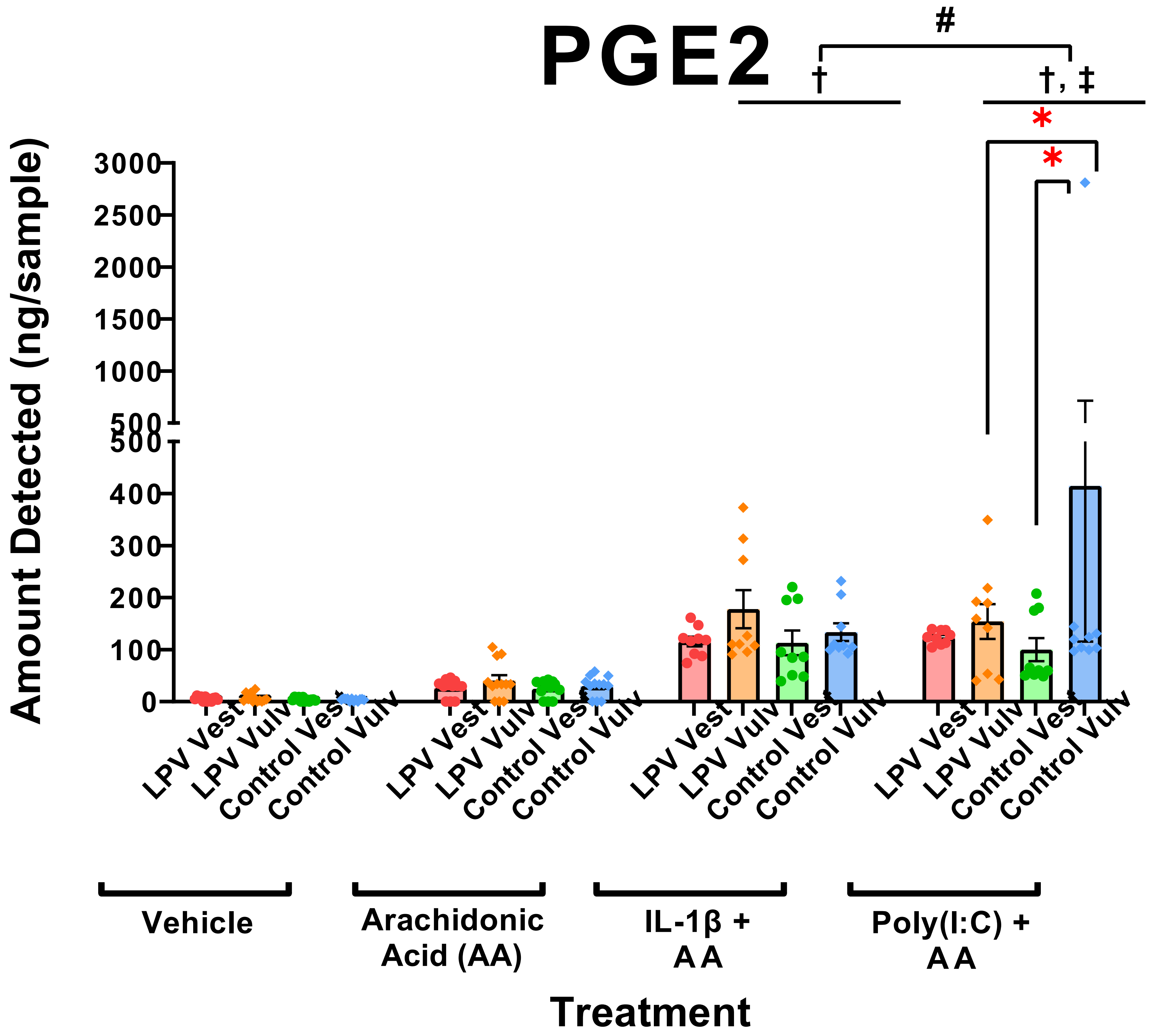

PGE2
#
†
†, ‡
*
*
Amount Detected (ng/sample)
3000
2500
2000
1500
1000
500
500
400
300
200
100
0
LPV Vest
LPV Vulv
Control Vest
Control Vulv
Vehicle
Arachidonic Acid (AA)
IL-1β + AA
Poly(I:C) + AA
Treatment

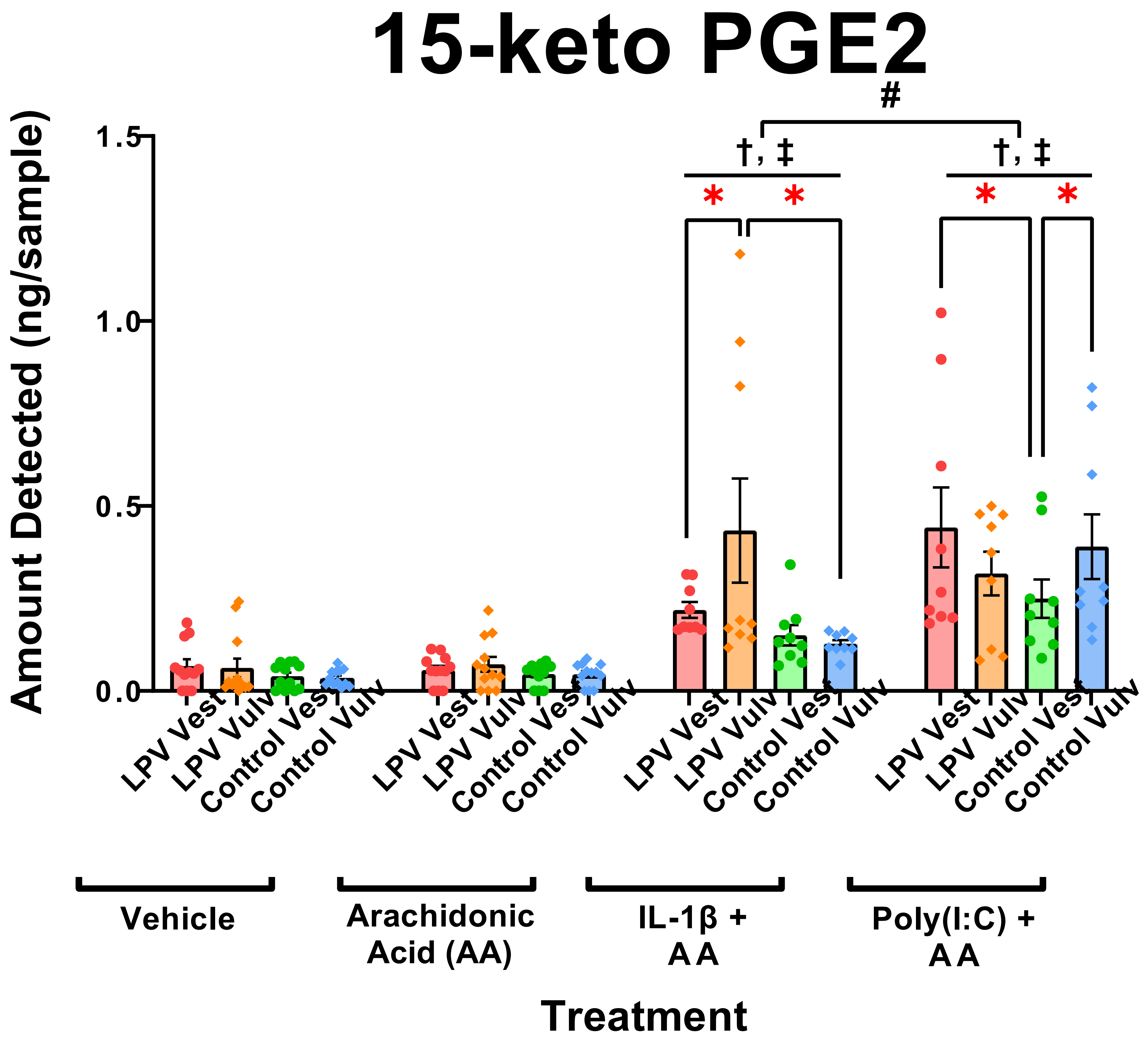

15-keto PGE2
Amount Detected (ng/sample)
0.0
0.5
1.0
1.5
#
†, ‡
†, ‡
*
*
*
*
LPV Vest
LPV Vulv
Control Vest
Control Vulv
LPV Vest
LPV Vulv
Control Vest
Control Vulv
LPV Vest
LPV Vulv
Control Vest
Control Vulv
LPV Vest
LPV Vulv
Control Vest
Control Vulv
Vehicle
Arachidonic Acid (AA)
IL-1β + AA
Poly(I:C) + AA
Treatment

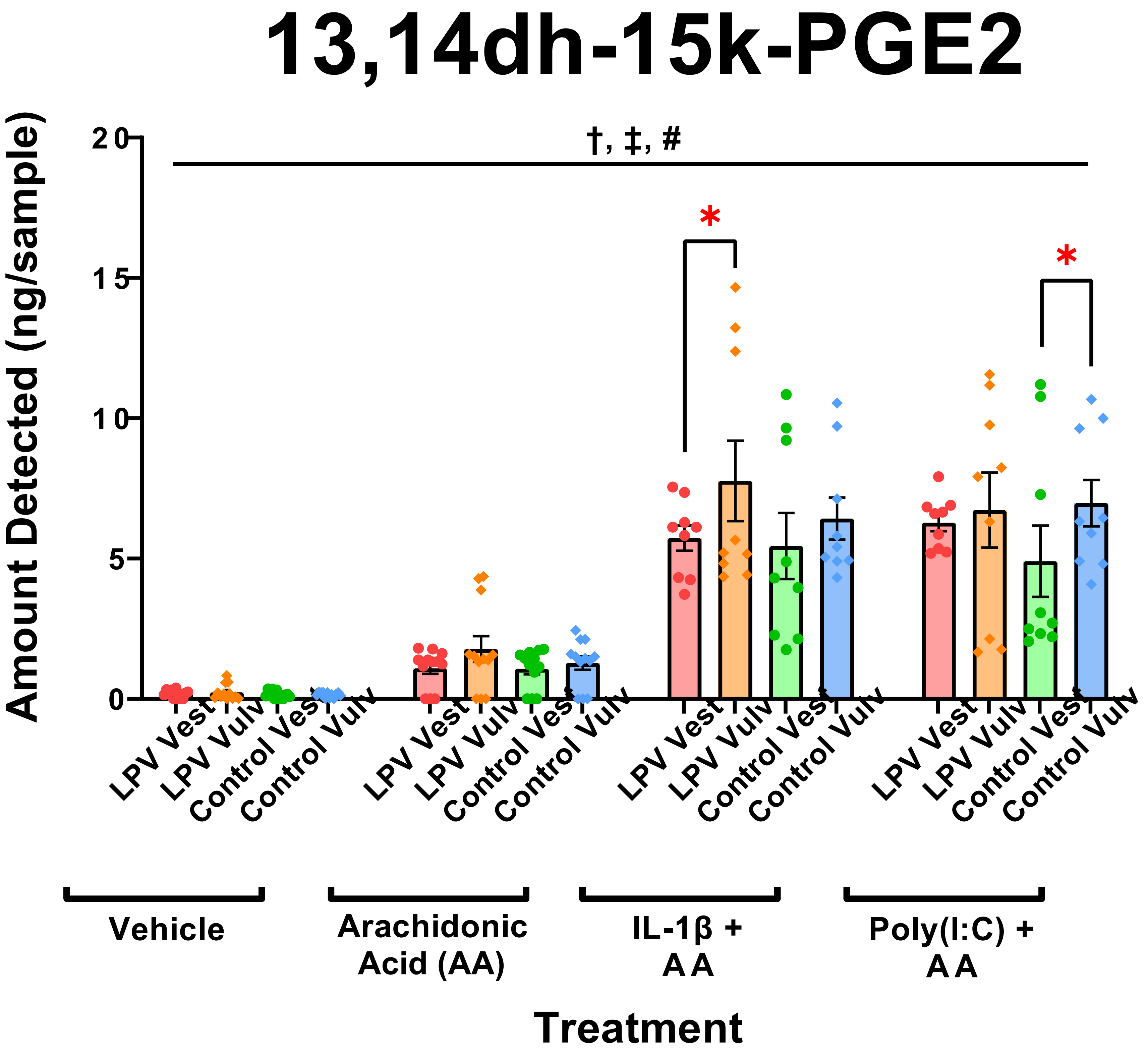

13,14dh-15k-PGE2
Amount Detected (ng/sample)
20
15
10
5
0
†, ‡, #
*
*
LPV Vest
LPV Vulv
Control Vest
Control Vulv
Vehicle
Arachidonic Acid (AA)
IL-1β + AA
Poly(I:C) + AA
Treatment

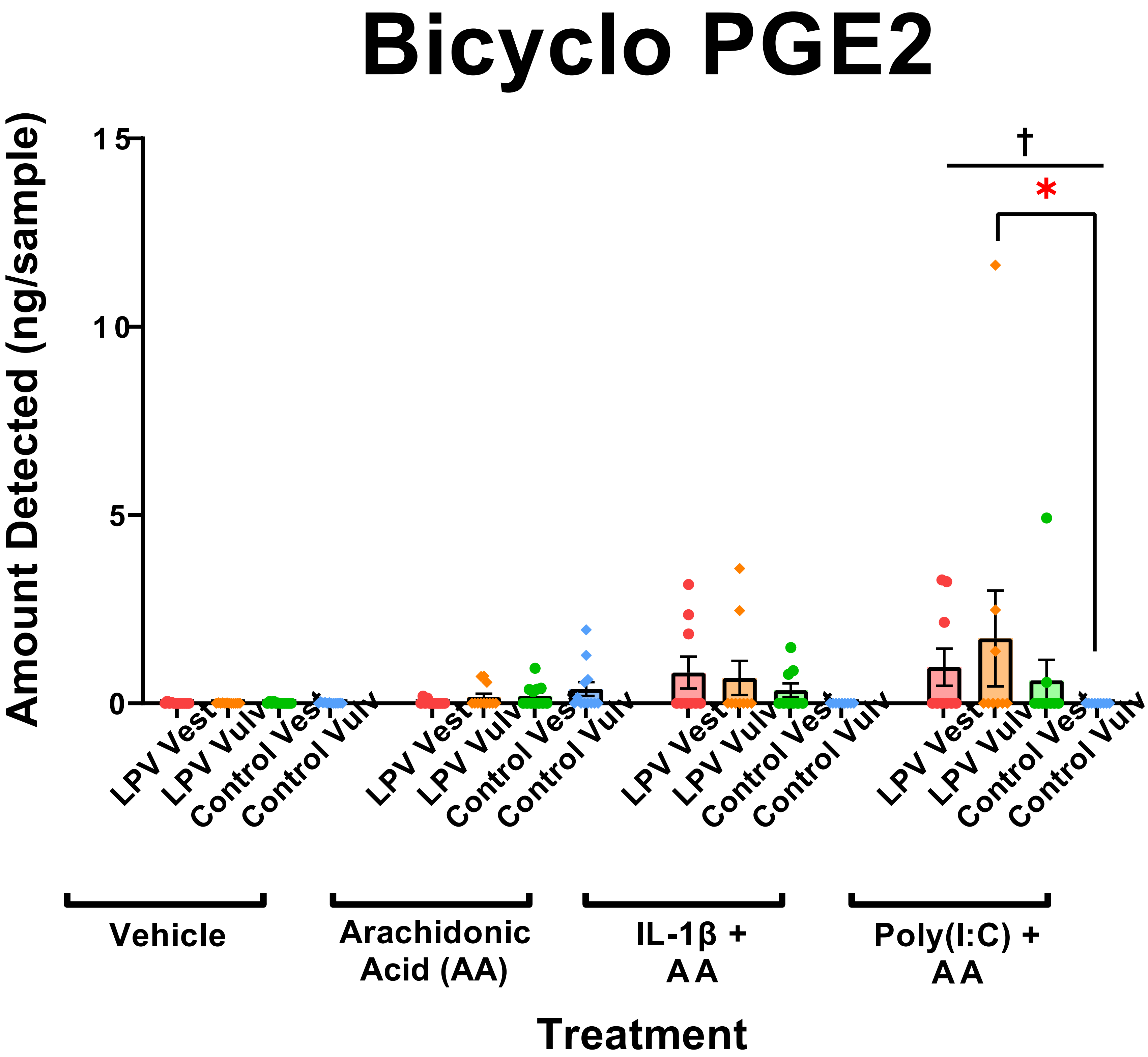

Bicyclo PGE2
Amount Detected (ng/sample)
15
10
5
0
†
*
LPV Vest
LPV Vulv
Control Vest
Control Vulv
Vehicle
Arachidonic Acid (AA)
IL-1β + AA
Poly(I:C) + AA
Treatment

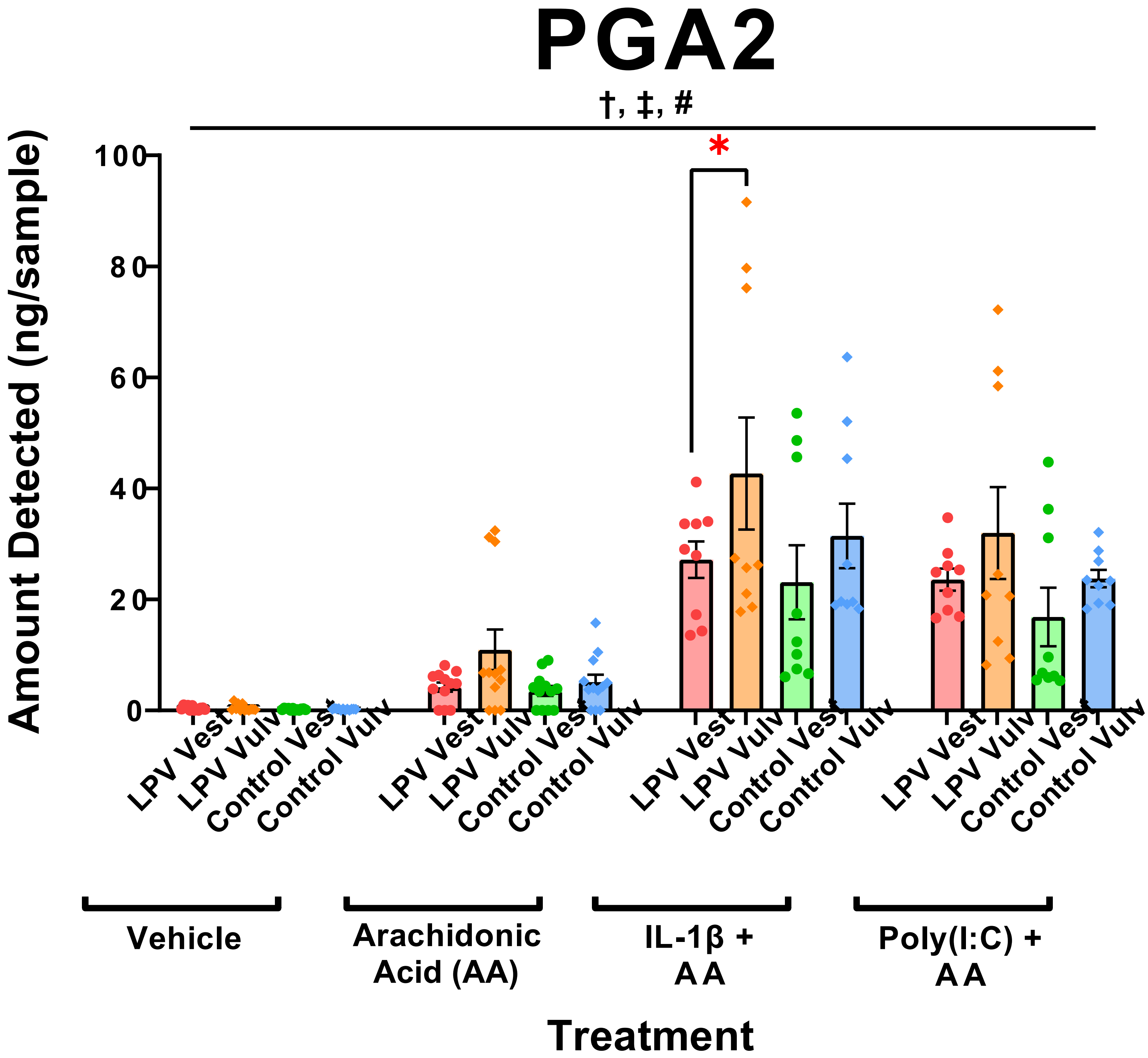

PGA2
†, ‡, #
*
Amount Detected (ng/sample)
100
80
60
40
20
0
LPV Vest
LPV Vulv
Control Vest
Control Vulv
Vehicle
Arachidonic Acid (AA)
IL-1β + AA
Poly(I:C) + AA
Treatment

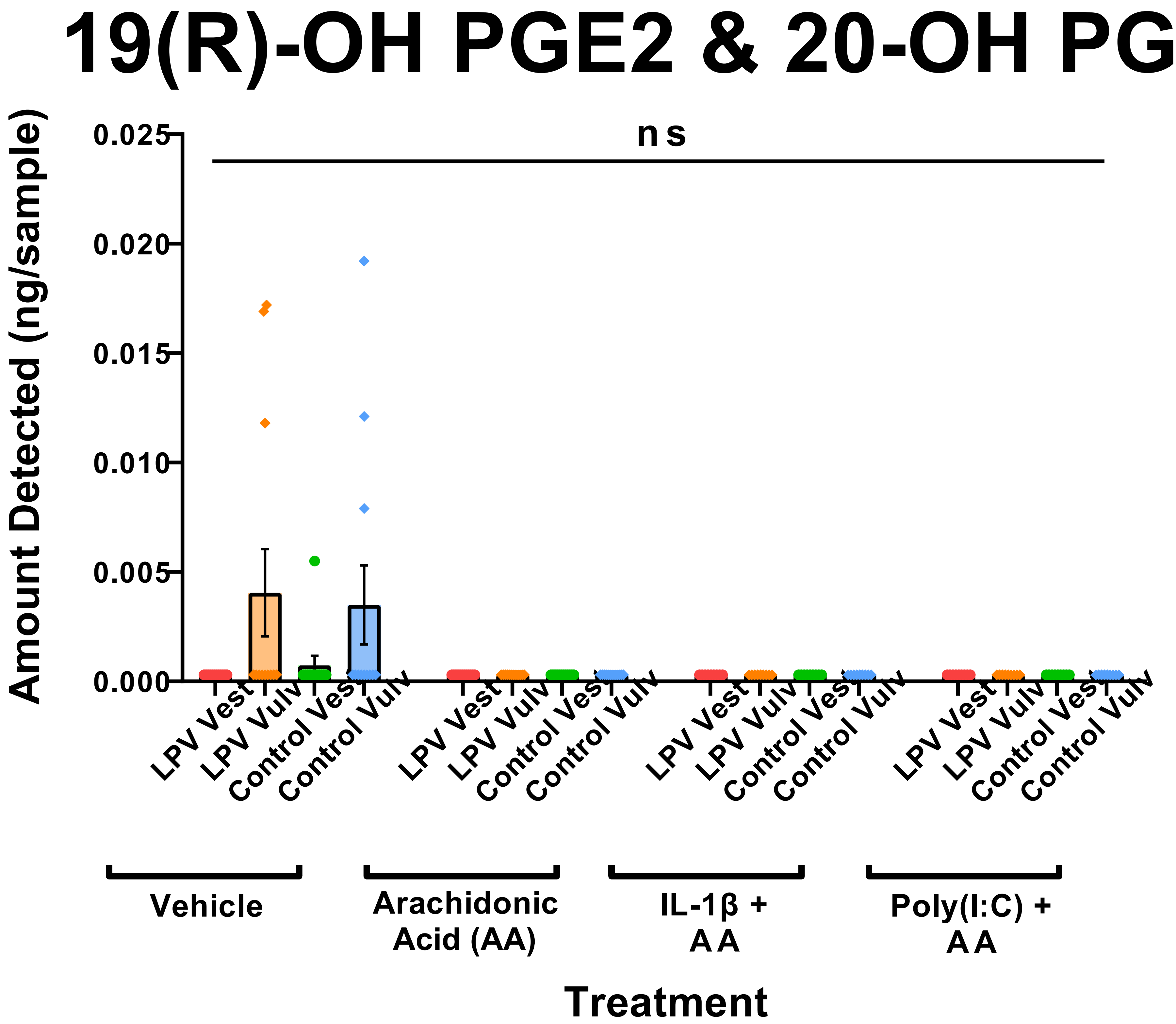

19(R)-OH PGE2 & 20-OH PGE2
Amount Detected (ng/sample)
0.025
0.020
0.015
0.010
0.005
0.000
ns
LPV Vest
LPV Vulv
Control Vest
Control Vulv
Vehicle
Arachidonic Acid (AA)
IL-1β + AA
Poly(I:C) + AA
Treatment

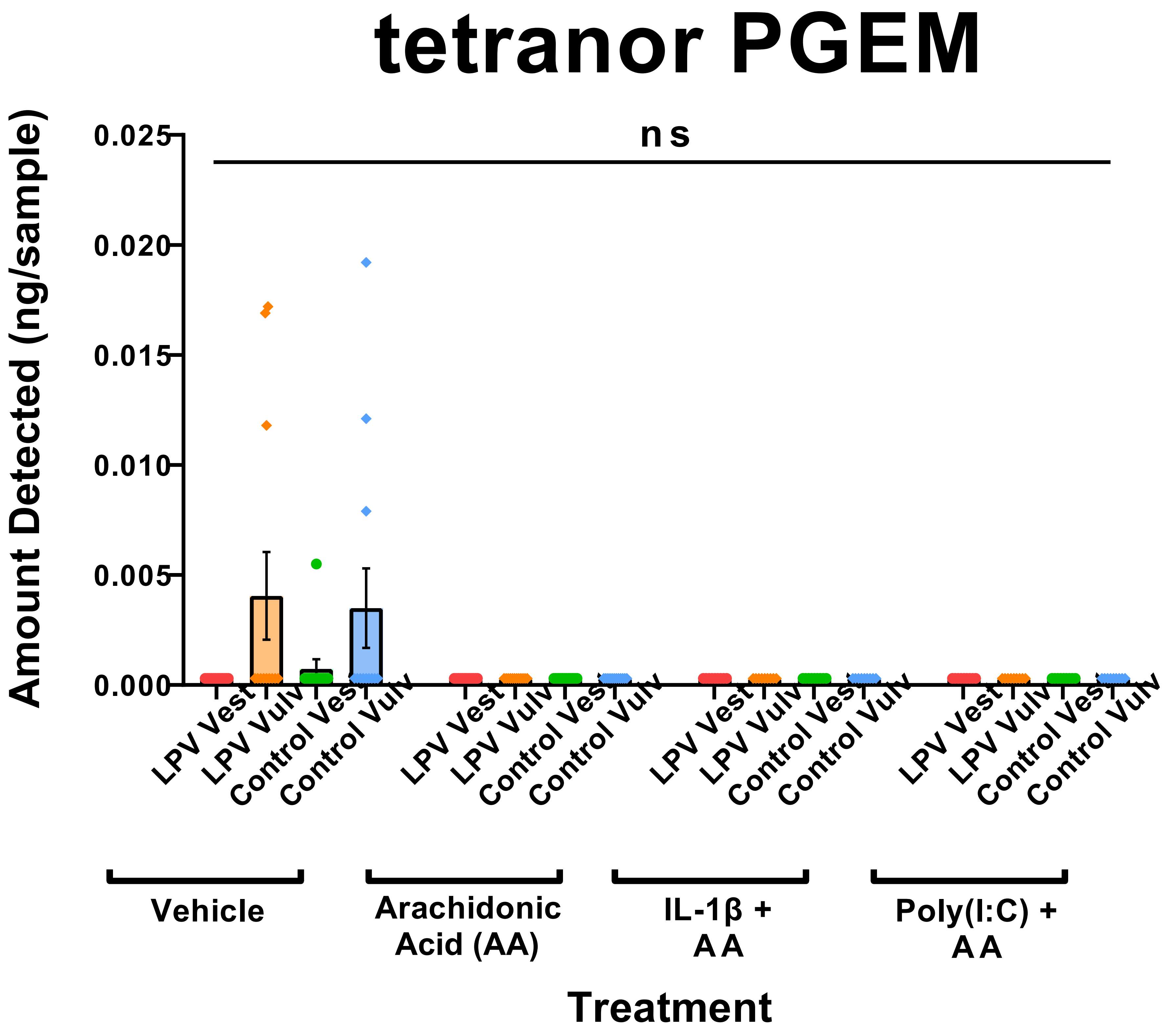

tetranor PGEM
ns
Amount Detected (ng/sample)
0.025
0.020
0.015
0.010
0.005
0.000
LPV Vest
LPV Vulv
Control Vest
Control Vulv
Vehicle
Arachidonic Acid (AA)
IL-1β + AA
Poly(I:C) + AA
Treatment

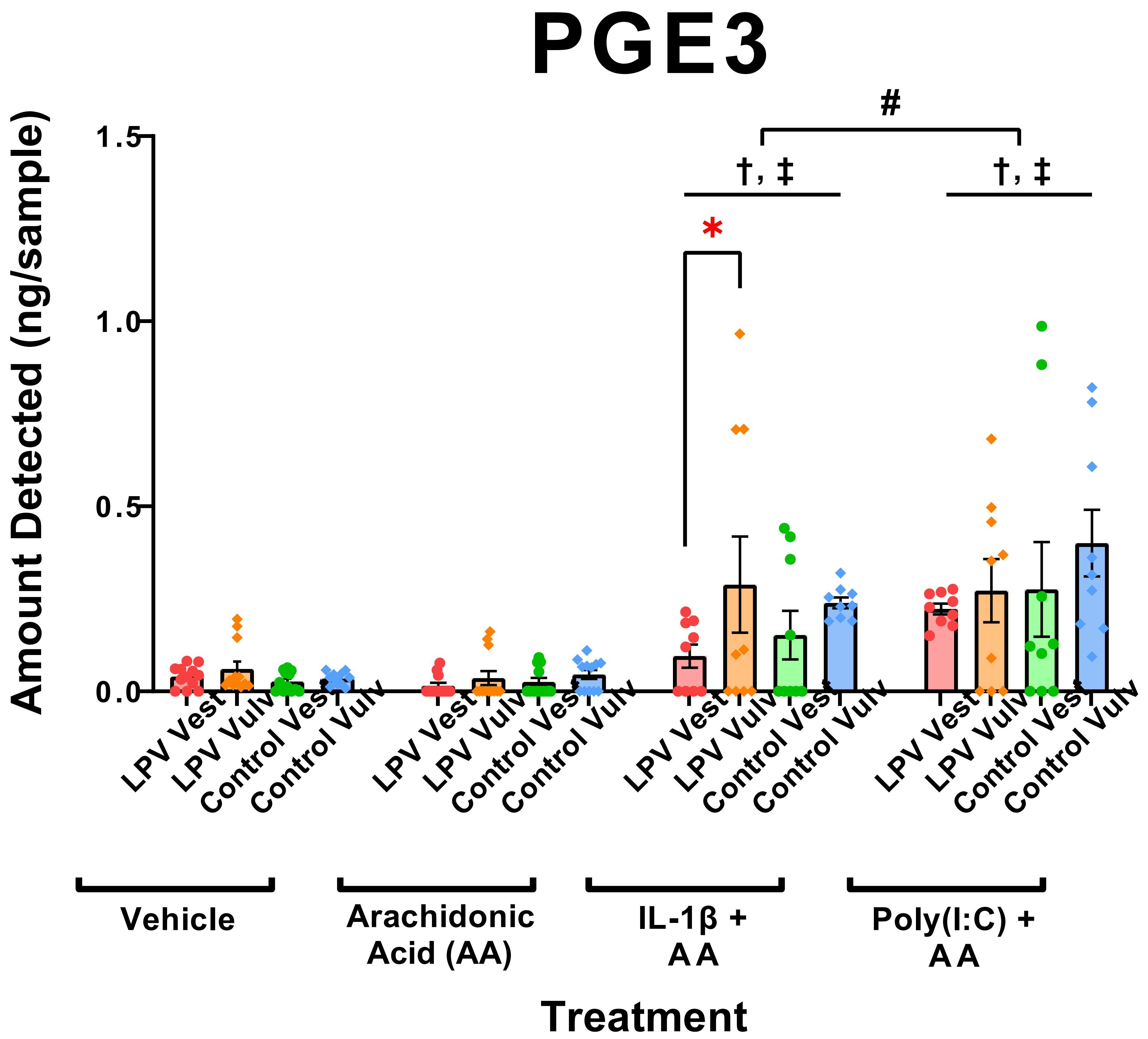

PGE3
Amount Detected (ng/sample)
1.5
1.0
0.5
0.0
#
†, ‡
†, ‡
*
LPV Vest
LPV Vulv
Control Vest
Control Vulv
Vehicle
Arachidonic Acid (AA)
IL-1β + AA
Poly(I:C) + AA
Treatment

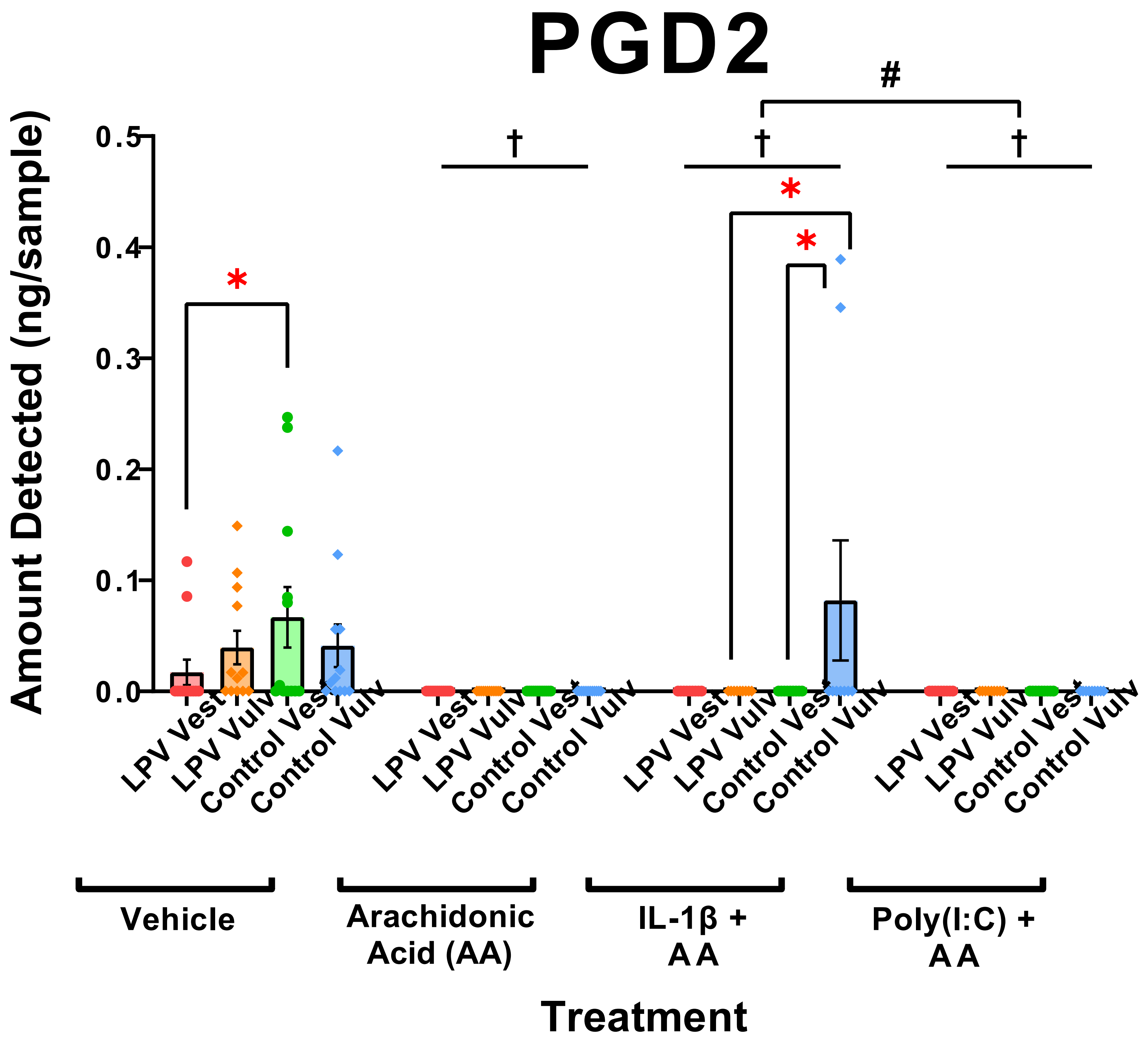

PGD2
#
†
†
†
*
*
*
Amount Detected (ng/sample)
0.5
0.4
0.3
0.2
0.1
0.0
LPV Vest
LPV Vulv
Control Vest
Control Vulv
Vehicle
Arachidonic Acid (AA)
IL-1β + AA
Poly(I:C) + AA
Treatment

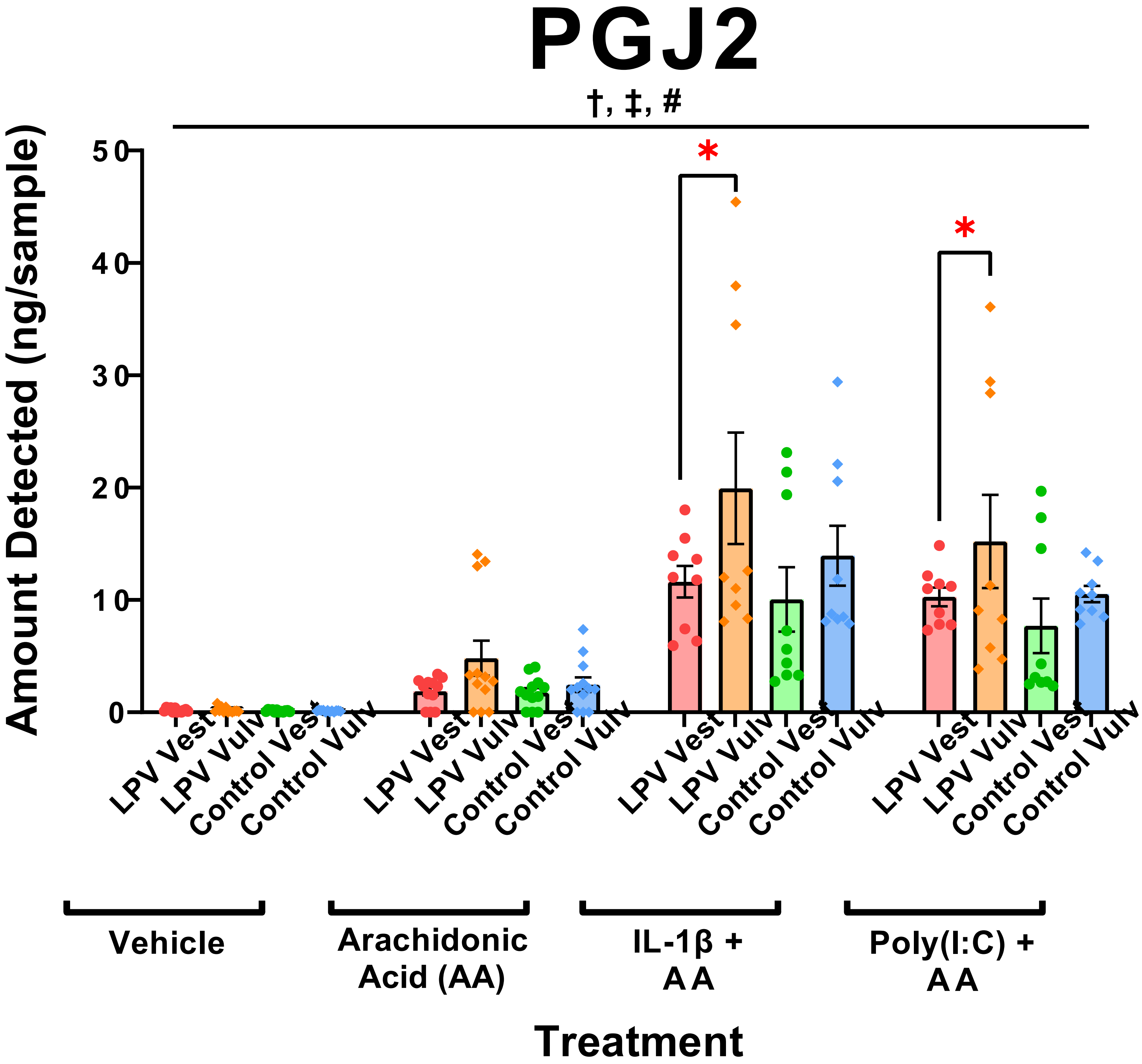

PGJ2
†, ‡, #
*
*
Amount Detected (ng/sample)
0
10
20
30
40
50
LPV Vest
LPV Vulv
Control Vest
Control Vulv
Vehicle
Arachidonic Acid (AA)
IL-1β + AA
Poly(I:C) + AA
Treatment

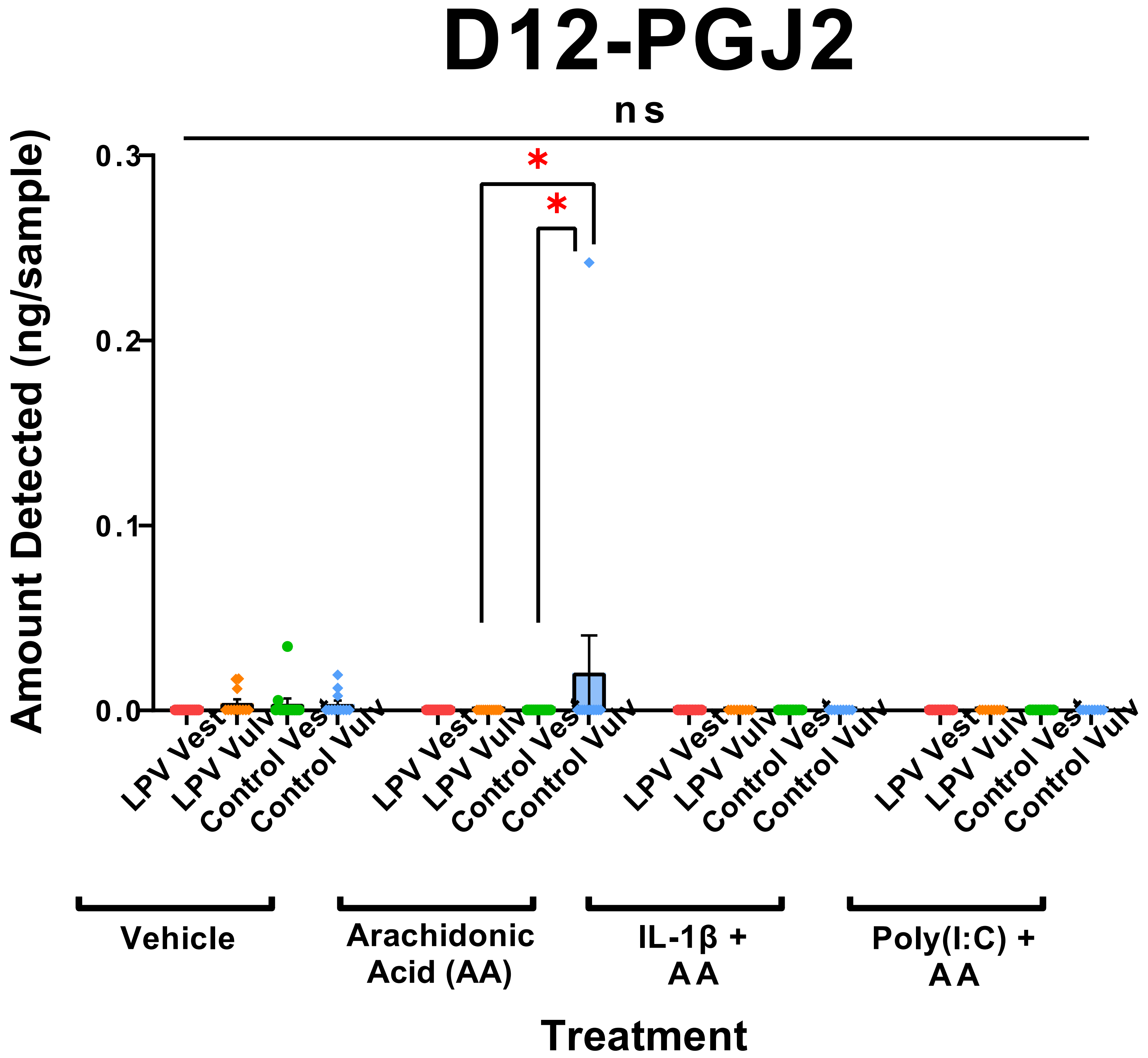

D12-PGJ2
ns
*
*
Amount Detected (ng/sample)
0.3
0.2
0.1
0.0
LPV Vest
LPV Vulv
Control Vest
Control Vulv
LPV Vest
LPV Vulv
Control Vest
Control Vulv
LPV Vest
LPV Vulv
Control Vest
Control Vulv
LPV Vest
LPV Vulv
Control Vest
Control Vulv
Vehicle
Arachidonic Acid (AA)
IL-1β + AA
Poly(I:C) + AA
Treatment

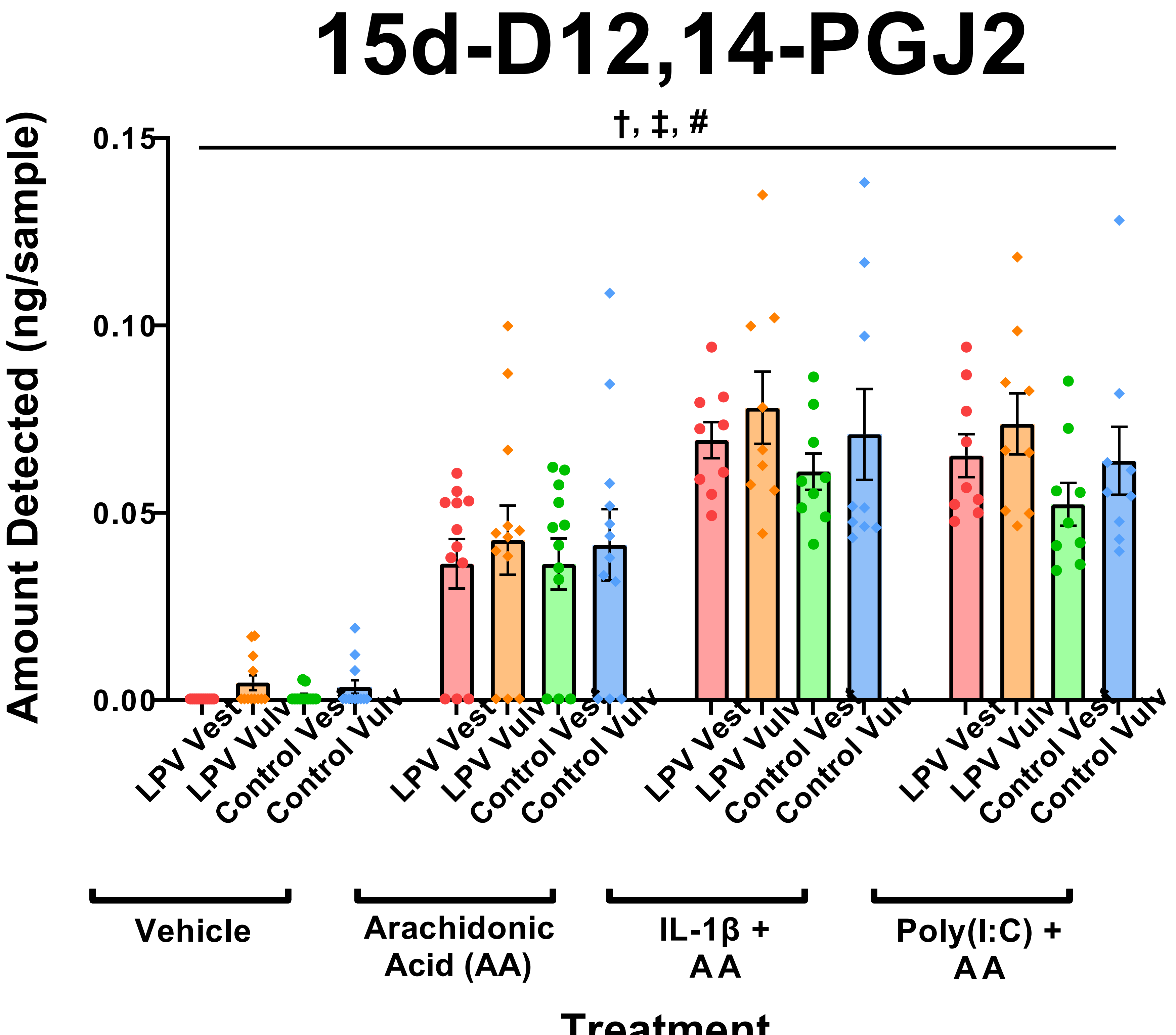
15d-D12,14-PGJ2
†, ‡, #
Amount Detected (ng/sample)
0.15
0.10
0.05
0.00
LPV Vest
LPV Vulv
Control Vest
Control Vulv
Vehicle
Arachidonic Acid (AA)
IL-1β + AA
Poly(I:C) + AA
Treatment

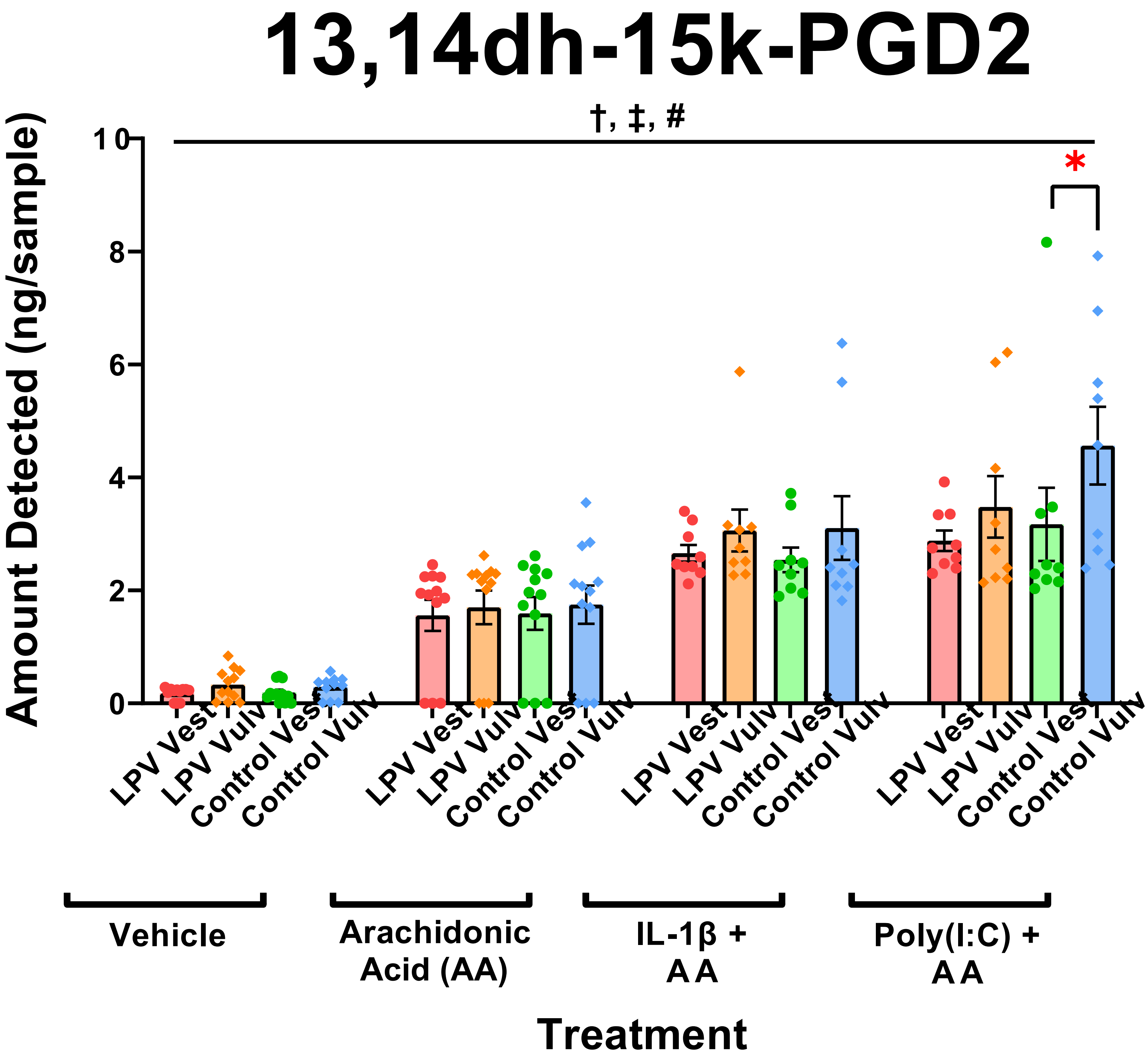

13,14dh-15k-PGD2
†, ‡, #
*
Amount Detected (ng/sample)
0
2
4
6
8
10
LPV Vest
LPV Vulv
Control Vest
Control Vulv
Vehicle
Arachidonic Acid (AA)
IL-1β + AA
Poly(I:C) + AA
Treatment

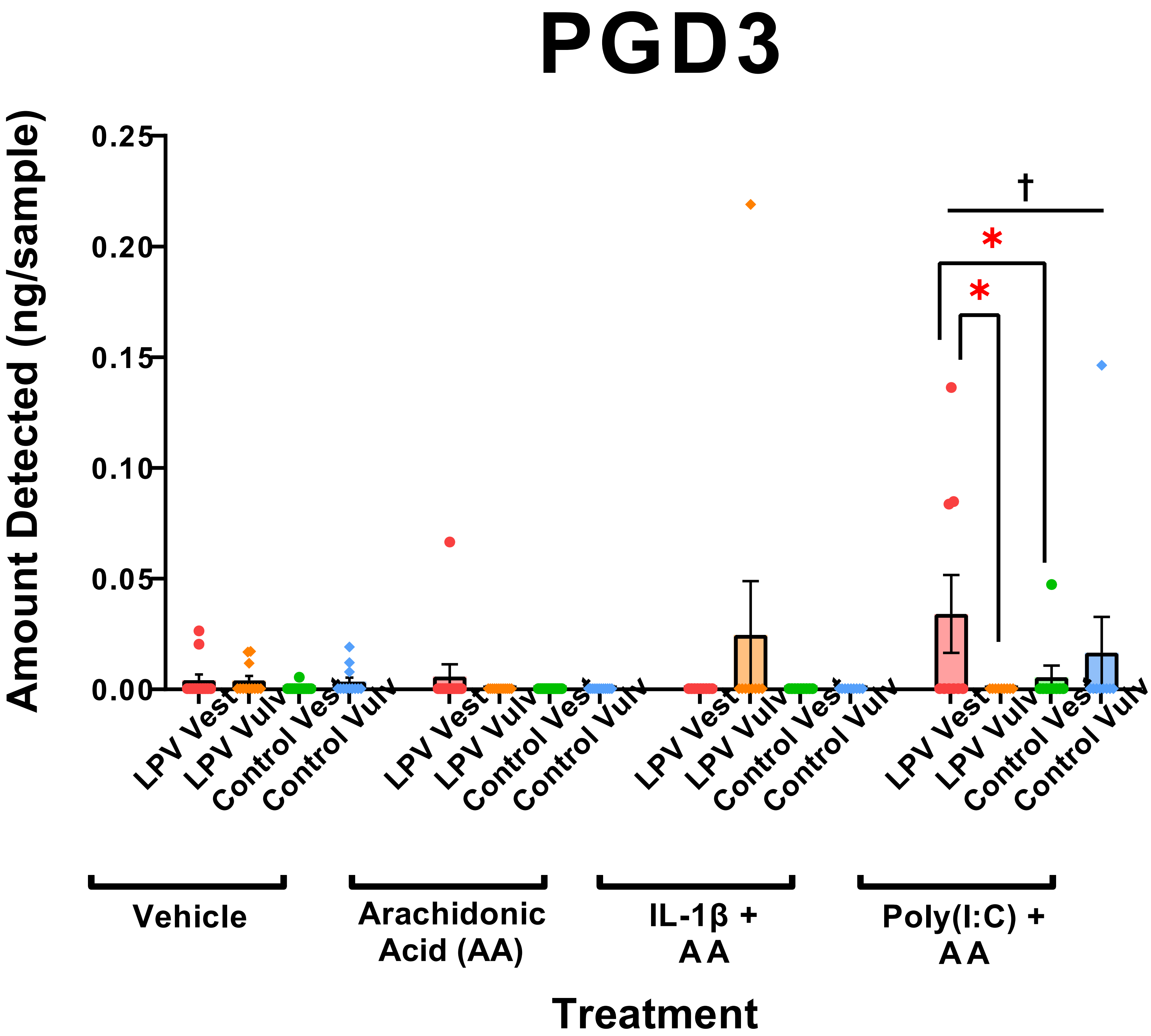

PGD3
Amount Detected (ng/sample)
0.25
0.20
0.15
0.10
0.05
0.00
†
*
*
LPV Vest
LPV Vulv
Control Vest
Control Vulv
Vehicle
Arachidonic Acid (AA)
IL-1β + AA
Poly(I:C) + AA
Treatment

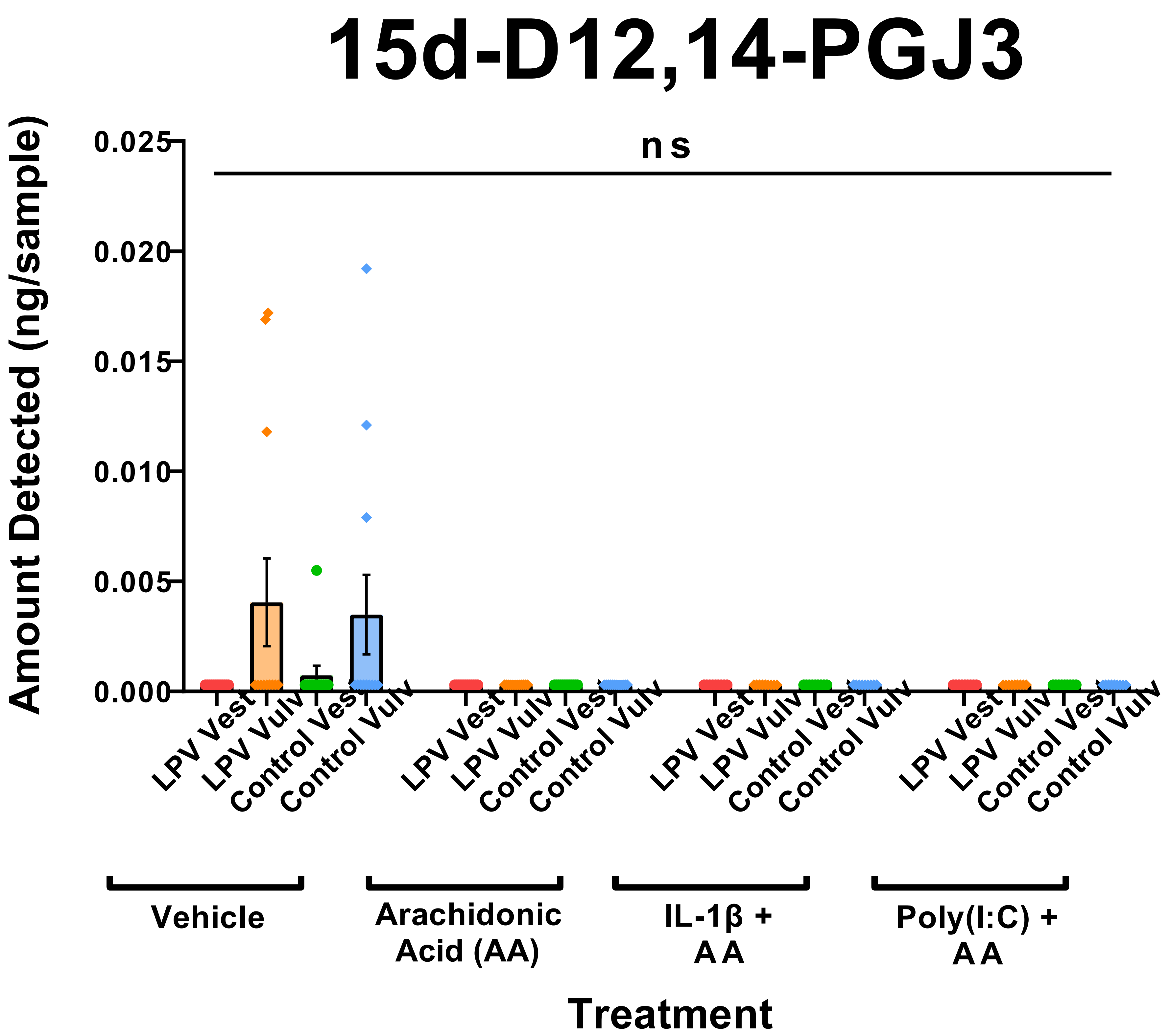
15d-D12,14-PGJ3
Amount Detected (ng/sample)
0.025
0.020
0.015
0.010
0.005
0.000
ns
LPV Vest
LPV Vulv
Control Vest
Control Vulv
Vehicle
Arachidonic Acid (AA)
IL-1β + AA
Poly(I:C) + AA
Treatment

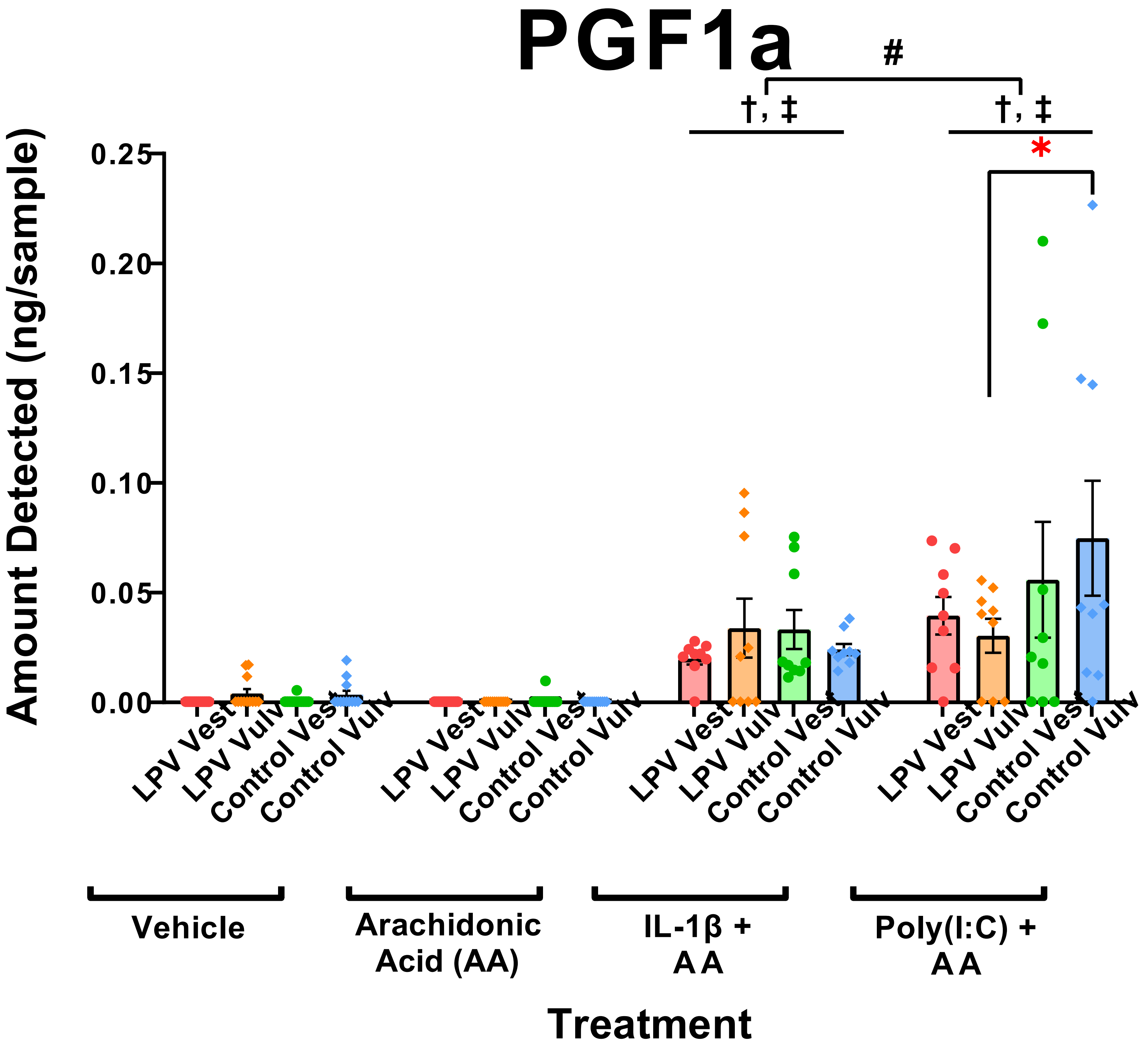

PGF1a
#
†, ‡
†, ‡
*
Amount Detected (ng/sample)
0.25
0.20
0.15
0.10
0.05
0.00
LPV Vest
LPV Vulv
Control Vest
Control Vulv
Vehicle
Arachidonic Acid (AA)
IL-1β + AA
Poly(I:C) + AA
Treatment

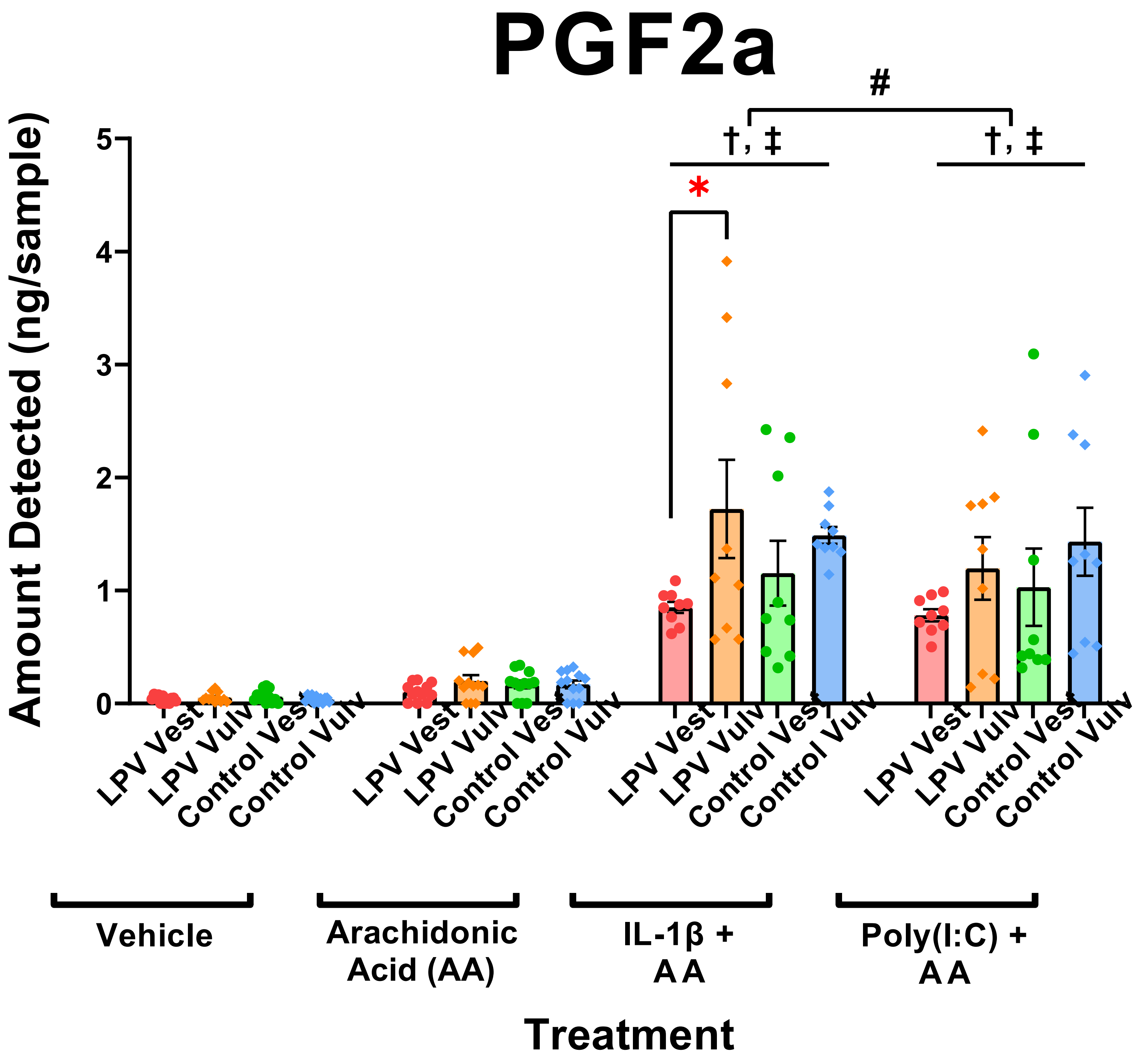
PGF2a
Amount Detected (ng/sample)
0
1
2
3
4
5
#
†, ‡
†, ‡
*
LPV Vest
LPV Vulv
Control Vest
Control Vulv
Vehicle
Arachidonic Acid (AA)
IL-1β + AA
Poly(I:C) + AA
Treatment

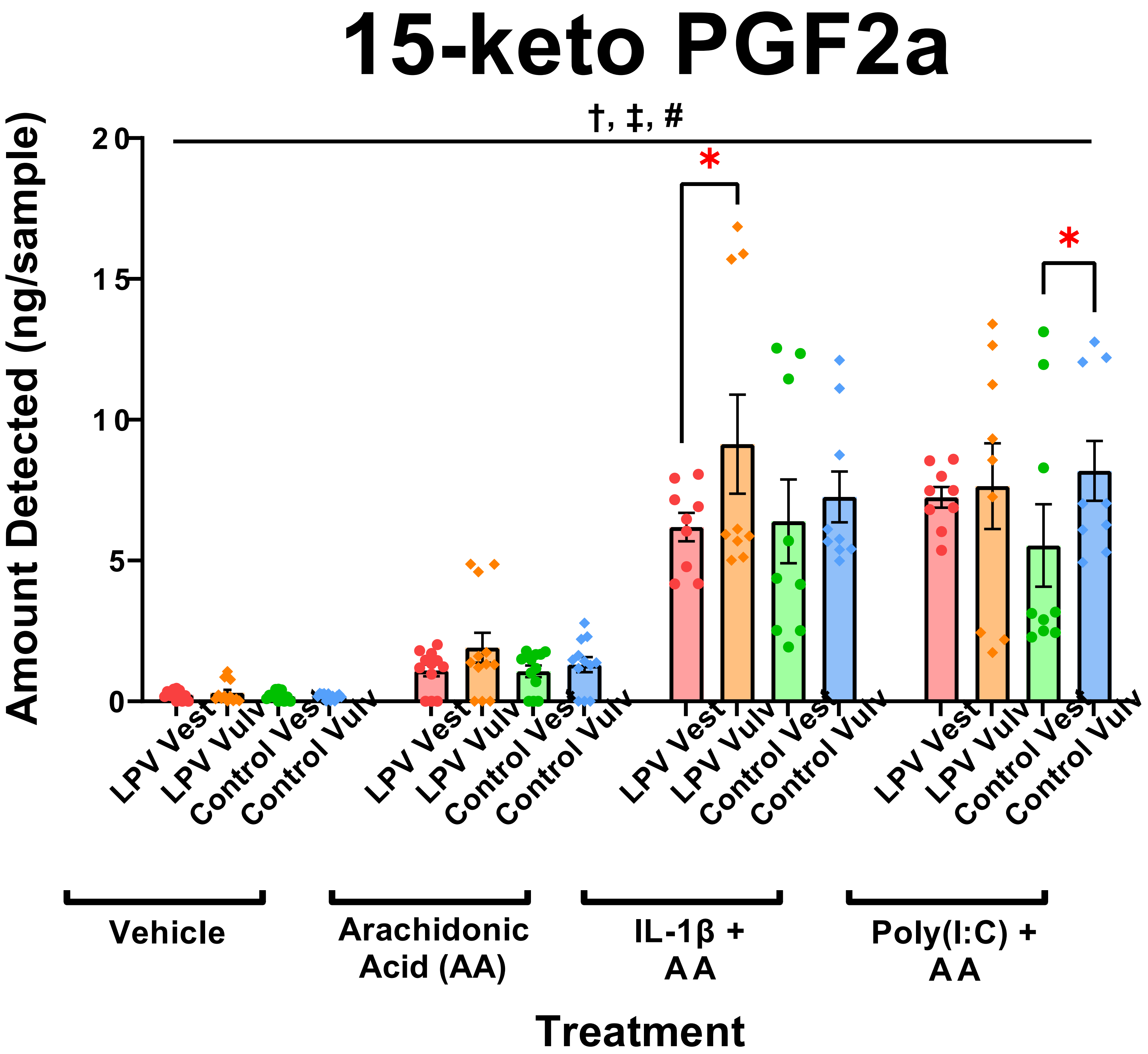

15-keto PGF2a
†, ‡, #
*
*
Amount Detected (ng/sample)
20
15
10
5
0
LPV Vest
LPV Vulv
Control Vest
Control Vulv
LPV Vest
LPV Vulv
Control Vest
Control Vulv
LPV Vest
LPV Vulv
Control Vest
Control Vulv
LPV Vest
LPV Vulv
Control Vest
Control Vulv
Vehicle
Arachidonic Acid (AA)
IL-1β + AA
Poly(I:C) + AA
Treatment

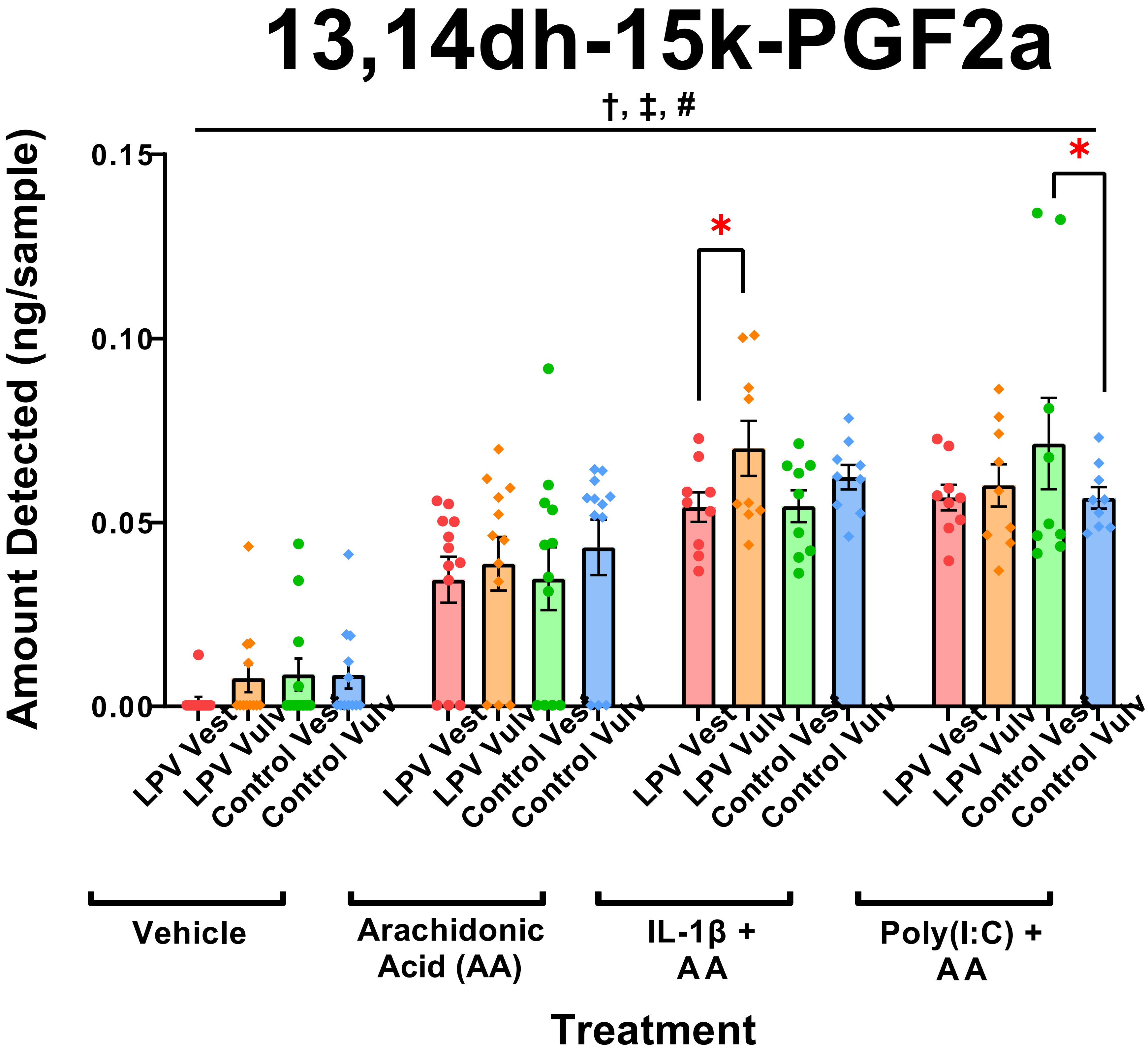

13,14dh-15k-PGF2a
†, ‡, #
*
*
Amount Detected (ng/sample)
0.15
0.10
0.05
0.00
LPV Vest
LPV Vulv
Control Vest
Control Vulv
Vehicle
Arachidonic Acid (AA)
IL-1β + AA
Poly(I:C) + AA
Treatment

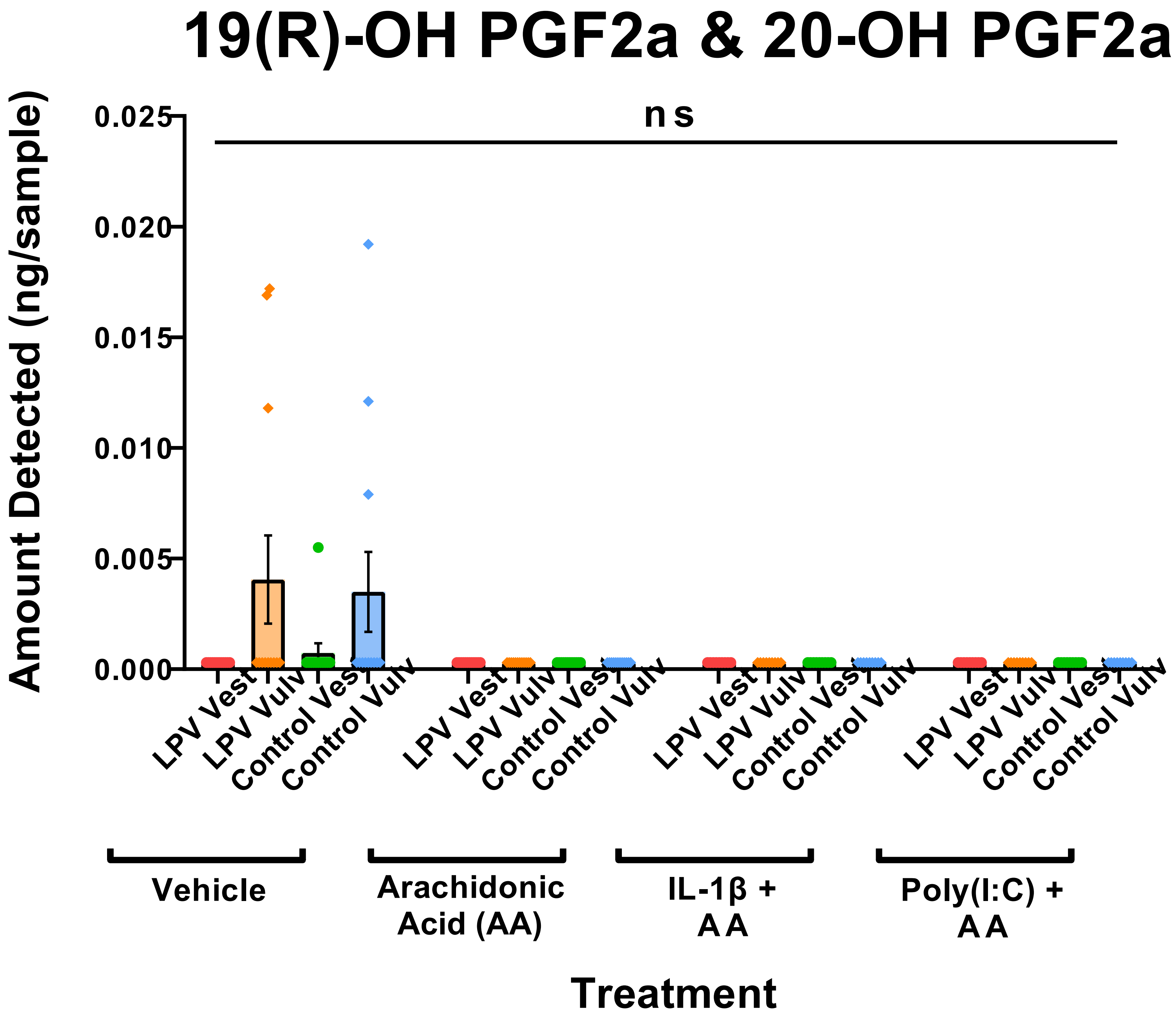
19(R)-OH PGF2a & 20-OH PGF2a
Amount Detected (ng/sample)
0.025
0.020
0.015
0.010
0.005
0.000
ns
LPV Vest
LPV Vulv
Control Vest
Control Vulv
Vehicle
Arachidonic Acid (AA)
IL-1β + AA
Poly(I:C) + AA
Treatment

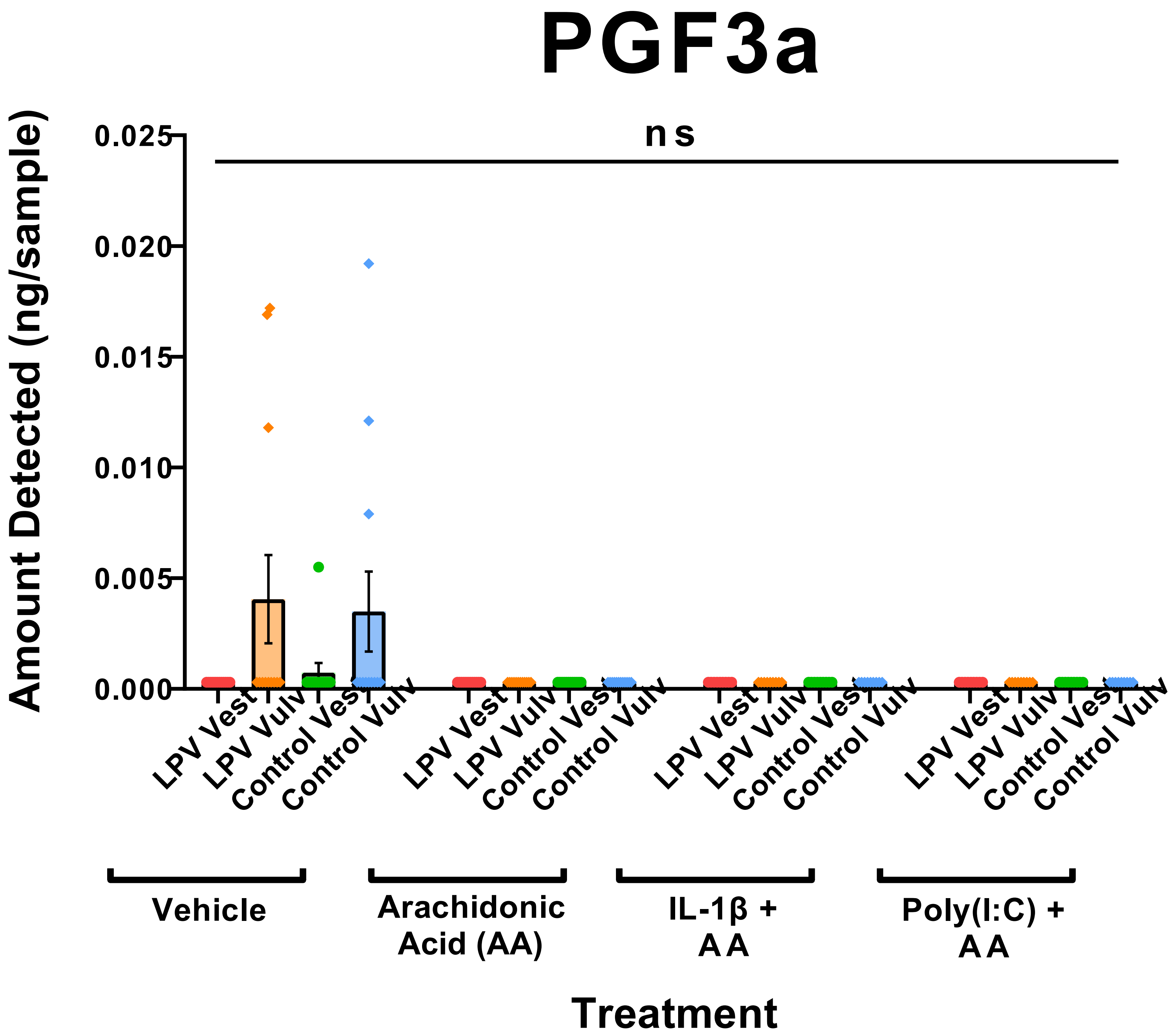

PGF3a
ns
Amount Detected (ng/sample)
0.025
0.020
0.015
0.010
0.005
0.000
LPV Vest
LPV Vulv
Control Vest
Control Vulv
LPV Vest
LPV Vulv
Control Vest
Control Vulv
LPV Vest
LPV Vulv
Control Vest
Control Vulv
LPV Vest
LPV Vulv
Control Vest
Control Vulv
Vehicle
Arachidonic Acid (AA)
IL-1β + AA
Poly(I:C) + AA
Treatment

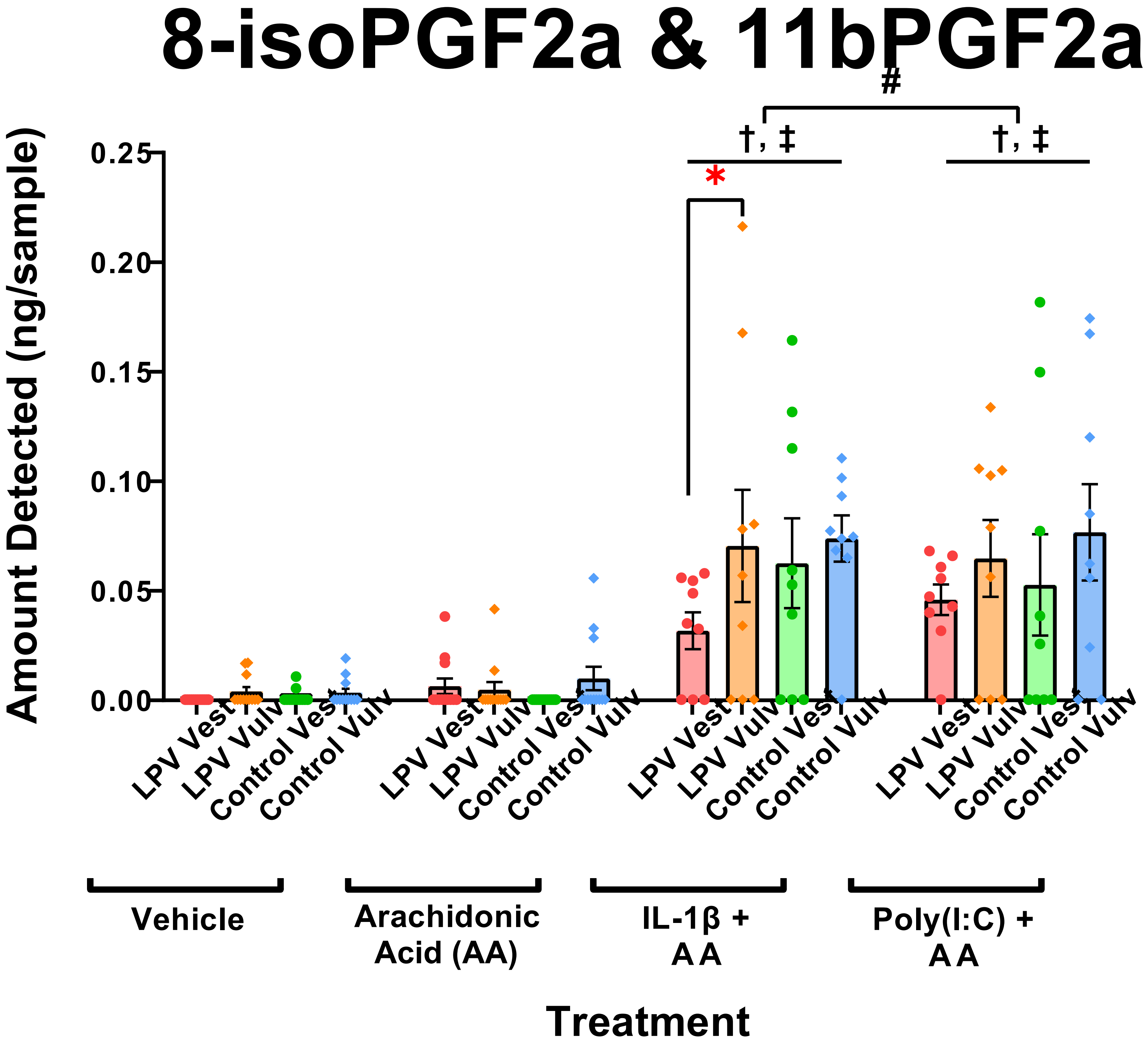

8-isoPGF2a & 11bPGF2a
Amount Detected (ng/sample)
0.25
0.20
0.15
0.10
0.05
0.00
#
†, ‡
†, ‡
*
LPV Vest
LPV Vulv
Control Vest
Control Vulv
Vehicle
Arachidonic Acid (AA)
IL-1β + AA
Poly(I:C) + AA
Treatment

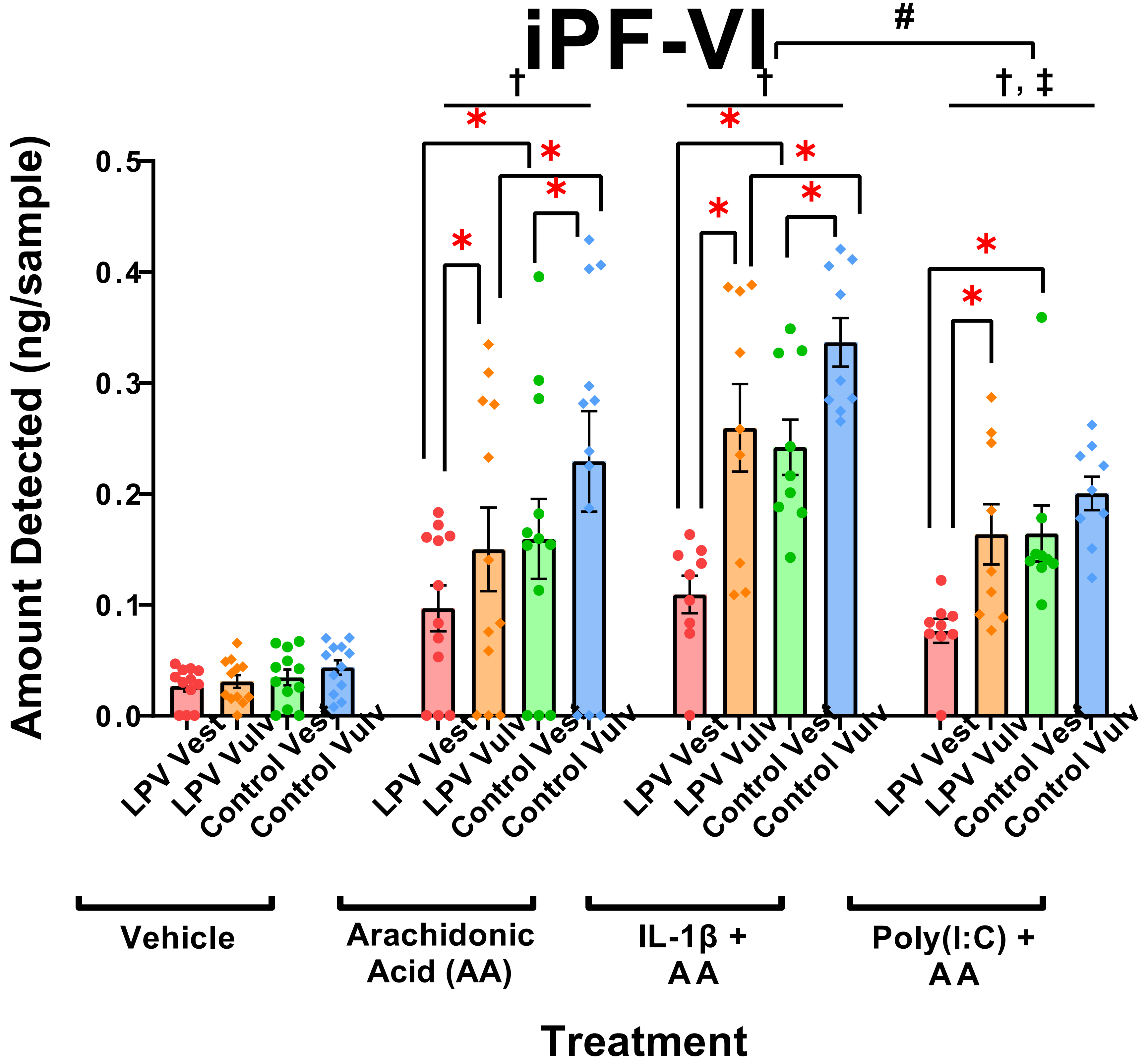

iPF-VI
#
†
†
†, ‡
*
Amount Detected (ng/sample)
0.0
0.1
0.2
0.3
0.4
0.5
LPV Vest
LPV Vulv
Control Vest
Control Vulv
Vehicle
Arachidonic Acid (AA)
IL-1β + AA
Poly(I:C) + AA
Treatment

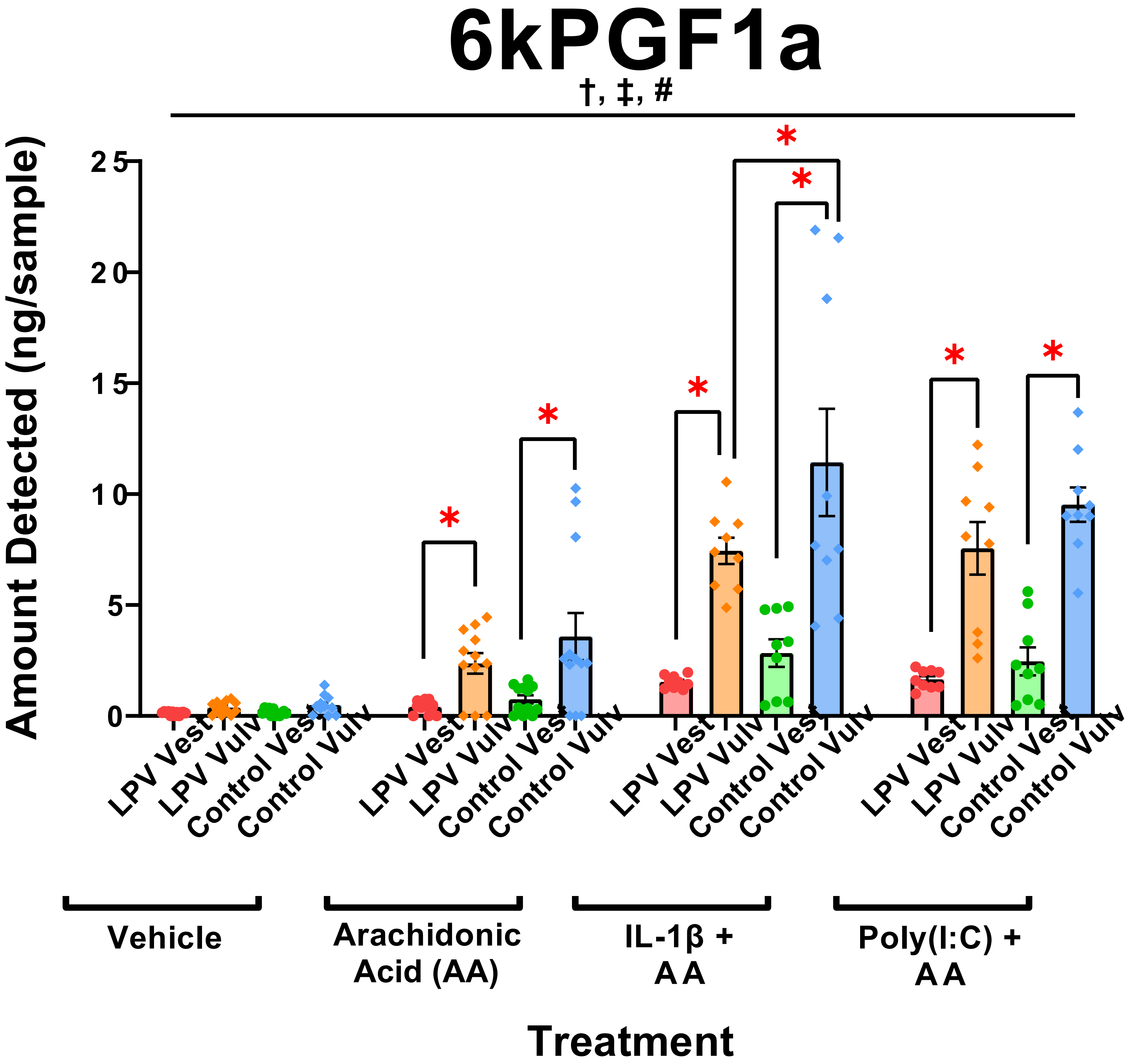

6kPGF1a
†, ‡, #
Amount Detected (ng/sample)
0
5
10
15
20
25
*
LPV Vest
LPV Vulv
Control Vest
Control Vulv
Vehicle
Arachidonic Acid (AA)
IL-1β + AA
Poly(I:C) + AA
Treatment

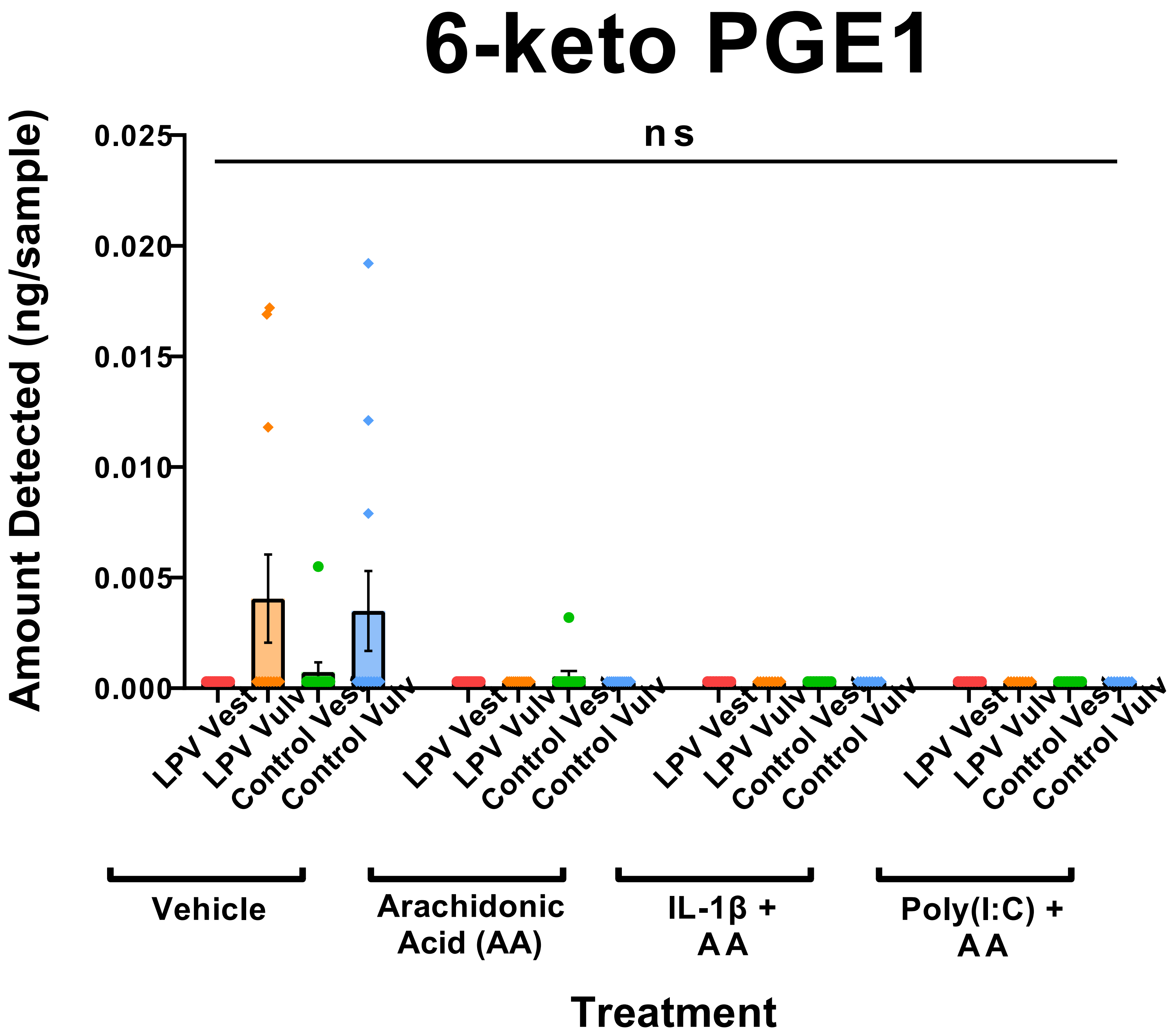
6-keto PGE1
ns
Amount Detected (ng/sample)
0.025
0.020
0.015
0.010
0.005
0.000
LPV Vest
LPV Vulv
Control Vest
Control Vulv
Vehicle
Arachidonic Acid (AA)
IL-1β + AA
Poly(I:C) + AA
Treatment

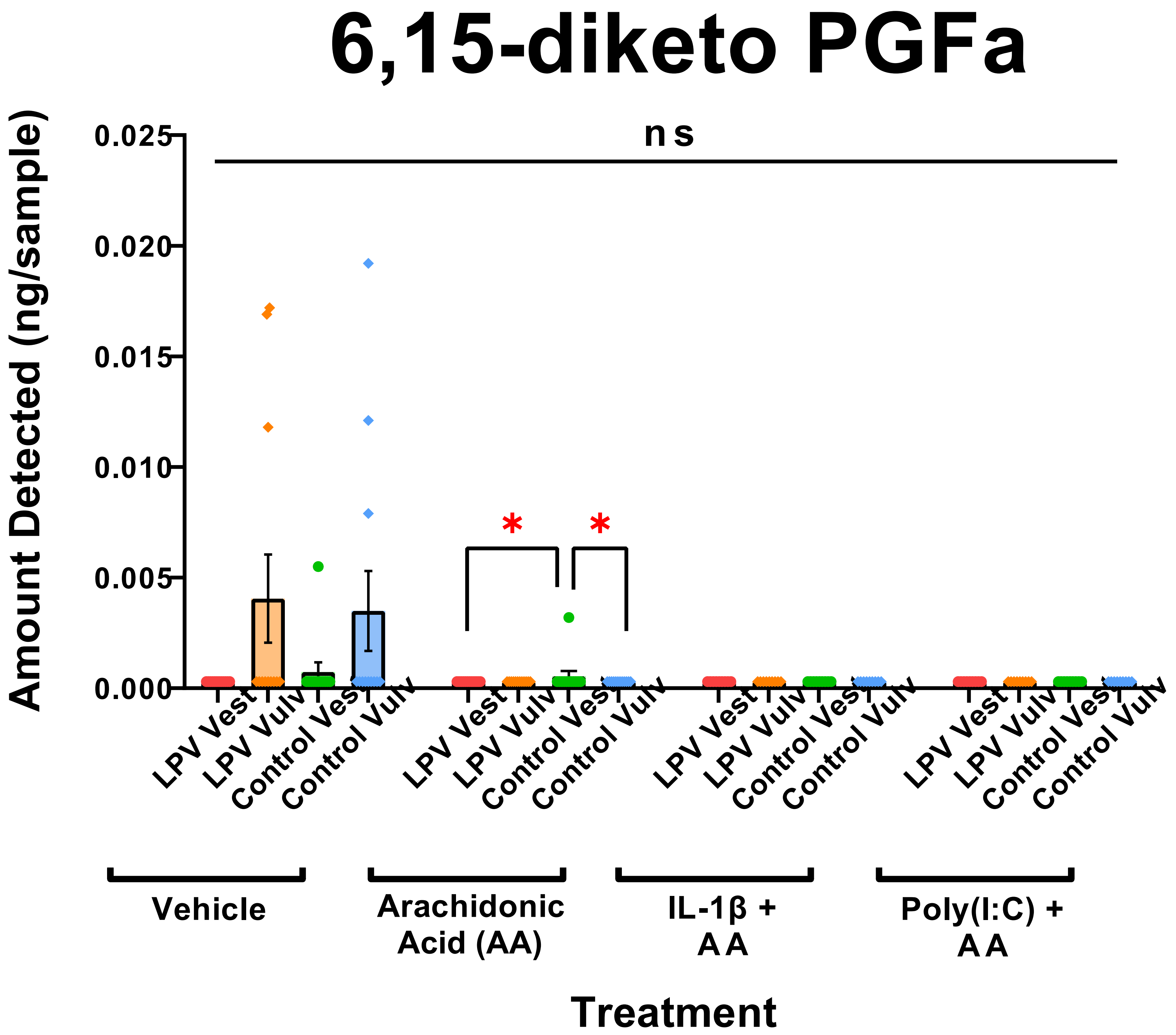
6,15-diketo PGFa
Amount Detected (ng/sample)
0.025
0.020
0.015
0.010
0.005
0.000
ns
*
*
LPV Vest
LPV Vulv
Control Vest
Control Vulv
Vehicle
Arachidonic Acid (AA)
IL-1β + AA
Poly(I:C) + AA
Treatment

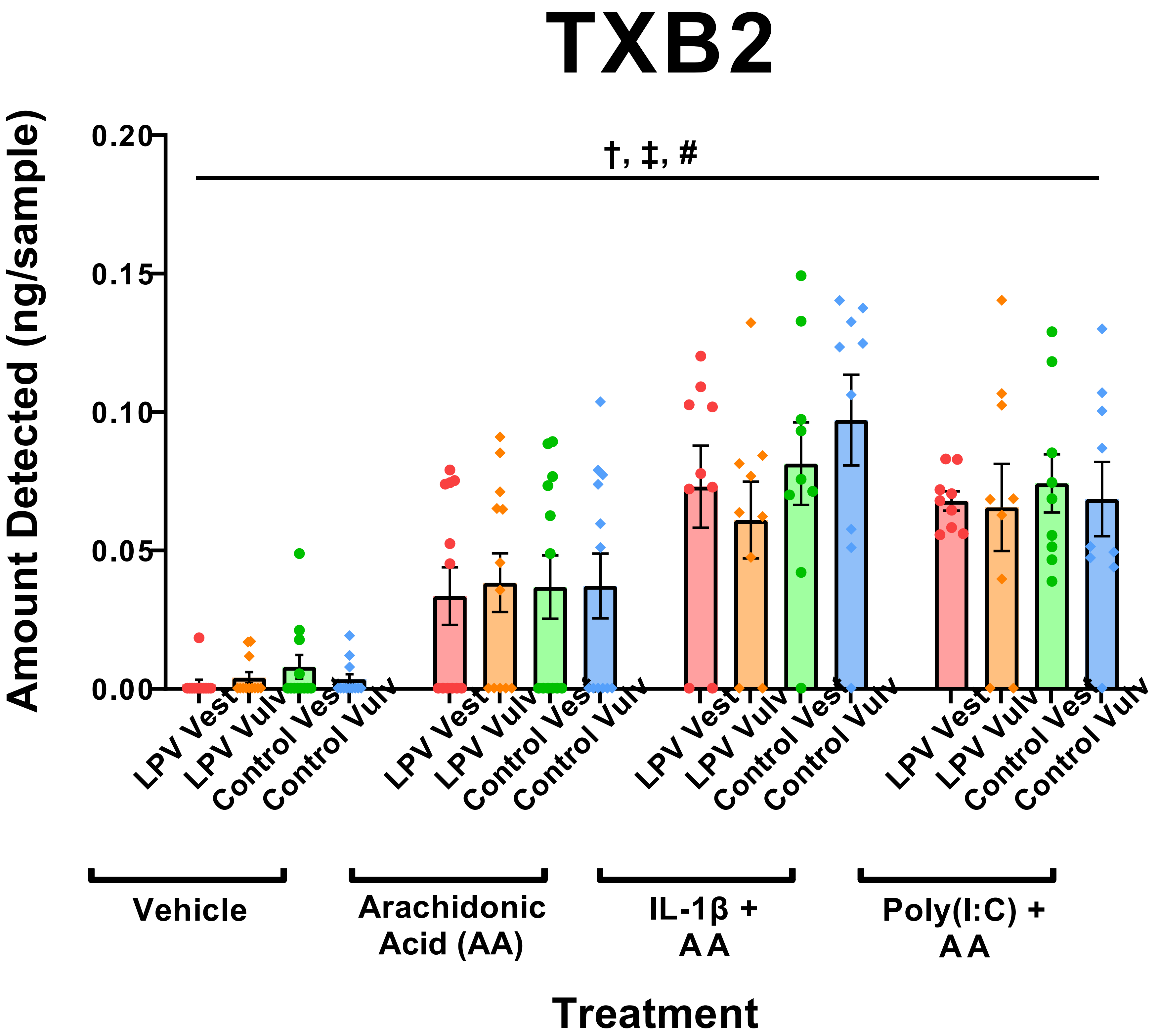
TXB2
Amount Detected (ng/sample)
0.20
0.15
0.10
0.05
0.00
†, ‡, #
LPV Vest
LPV Vulv
Control Vest
Control Vulv
Vehicle
Arachidonic Acid (AA)
IL-1β + AA
Poly(I:C) + AA
Treatment

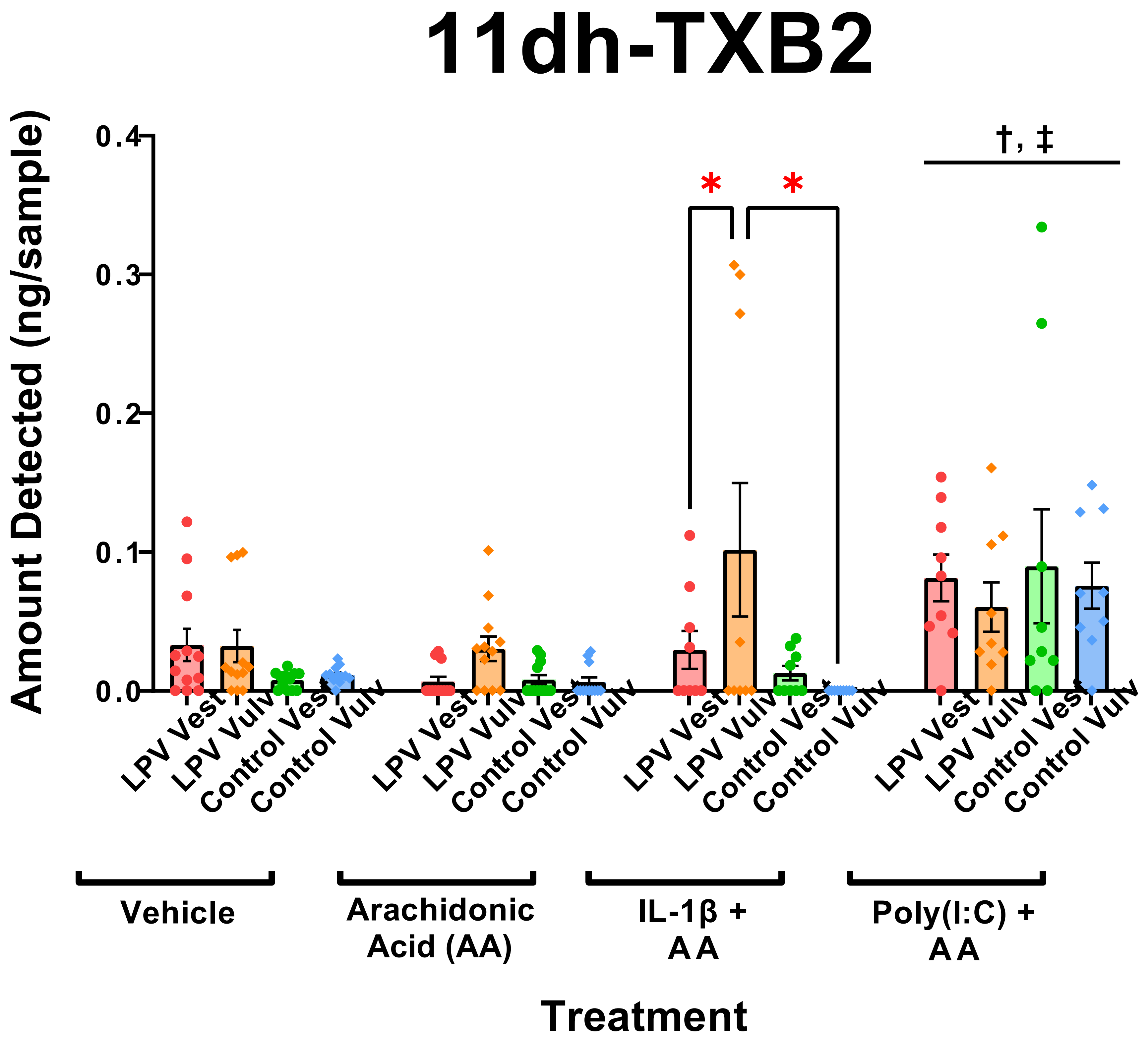

11dh-TXB2
Amount Detected (ng/sample)
0.0
0.1
0.2
0.3
0.4
*
*
†, ‡
LPV Vest
LPV Vulv
Control Vest
Control Vulv
Vehicle
Arachidonic Acid (AA)
IL-1β + AA
Poly(I:C) + AA
Treatment

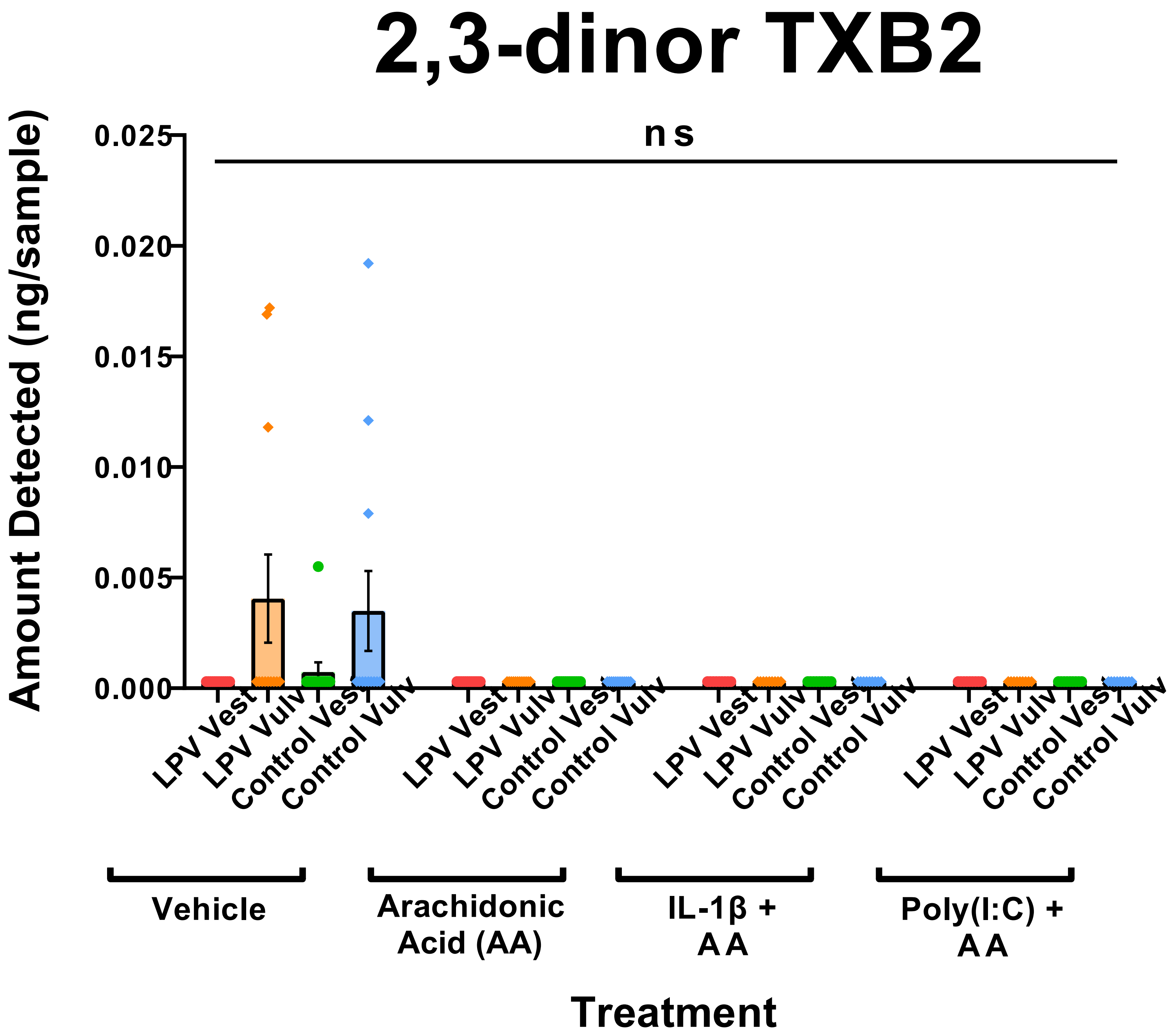

2,3-dinor TXB2
ns
Amount Detected (ng/sample)
0.025
0.020
0.015
0.010
0.005
0.000
LPV Vest
LPV Vulv
Control Vest
Control Vulv
Vehicle
Arachidonic Acid (AA)
IL-1β + AA
Poly(I:C) + AA
Treatment

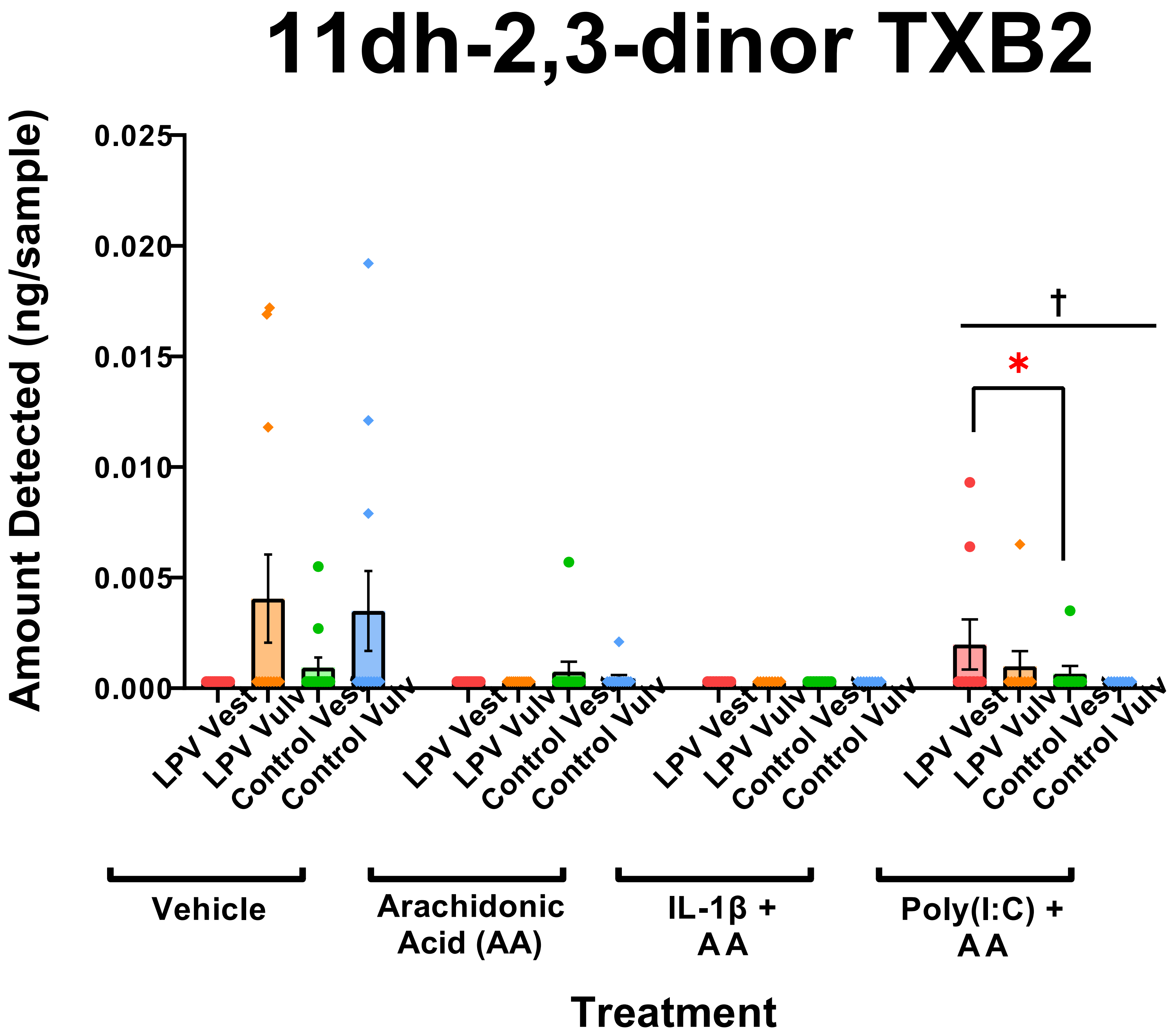
11dh-2,3-dinor TXB2
Amount Detected (ng/sample)
0.025
0.020
0.015
0.010
0.005
0.000
†
*
LPV Vest
LPV Vulv
Control Vest
Control Vulv
Vehicle
Arachidonic Acid (AA)
IL-1β + AA
Poly(I:C) + AA
Treatment

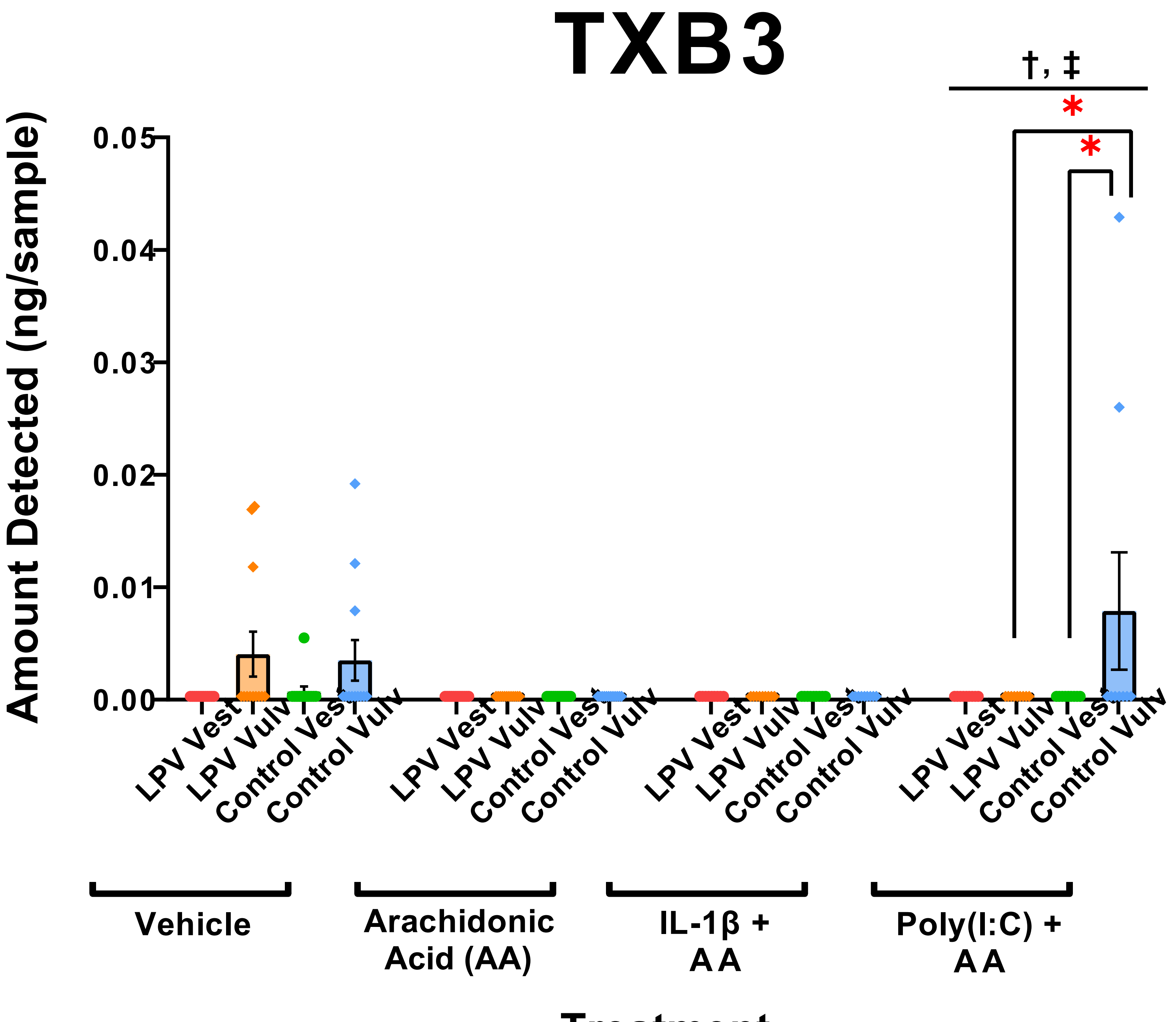

TXB3
†, ‡
*
*
Amount Detected (ng/sample)
0.05
0.04
0.03
0.02
0.01
0.00
LPV Vest
LPV Vulv
Control Vest
Control Vulv
Vehicle
Arachidonic Acid (AA)
IL-1β + AA
Poly(I:C) + AA
Treatment

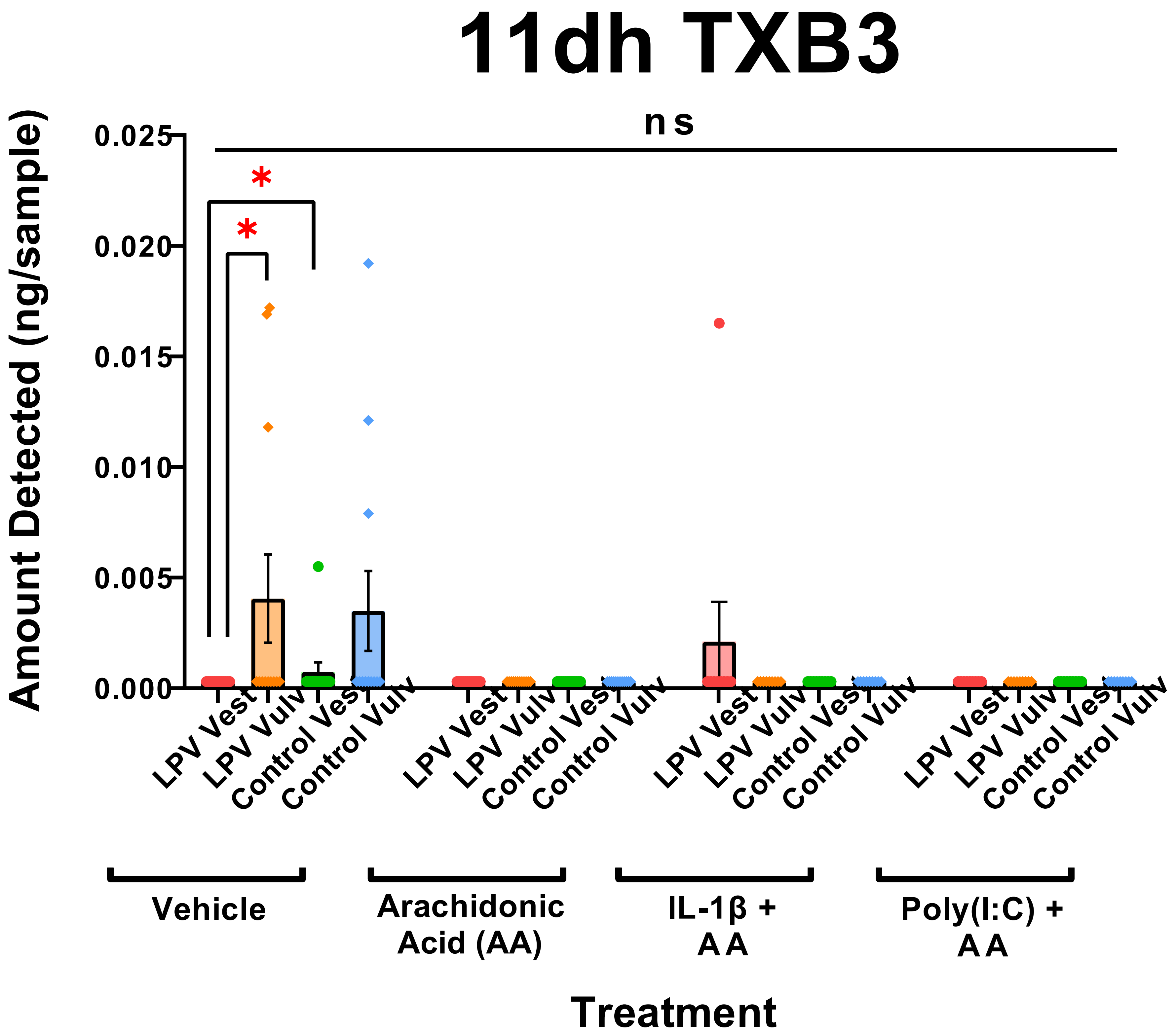

11dh TXB3
Amount Detected (ng/sample)
0.025
0.020
0.015
0.010
0.005
0.000
ns
*
*
LPV Vest
LPV Vulv
Control Vest
Control Vulv
Vehicle
Arachidonic Acid (AA)
IL-1β + AA
Poly(I:C) + AA
Treatment

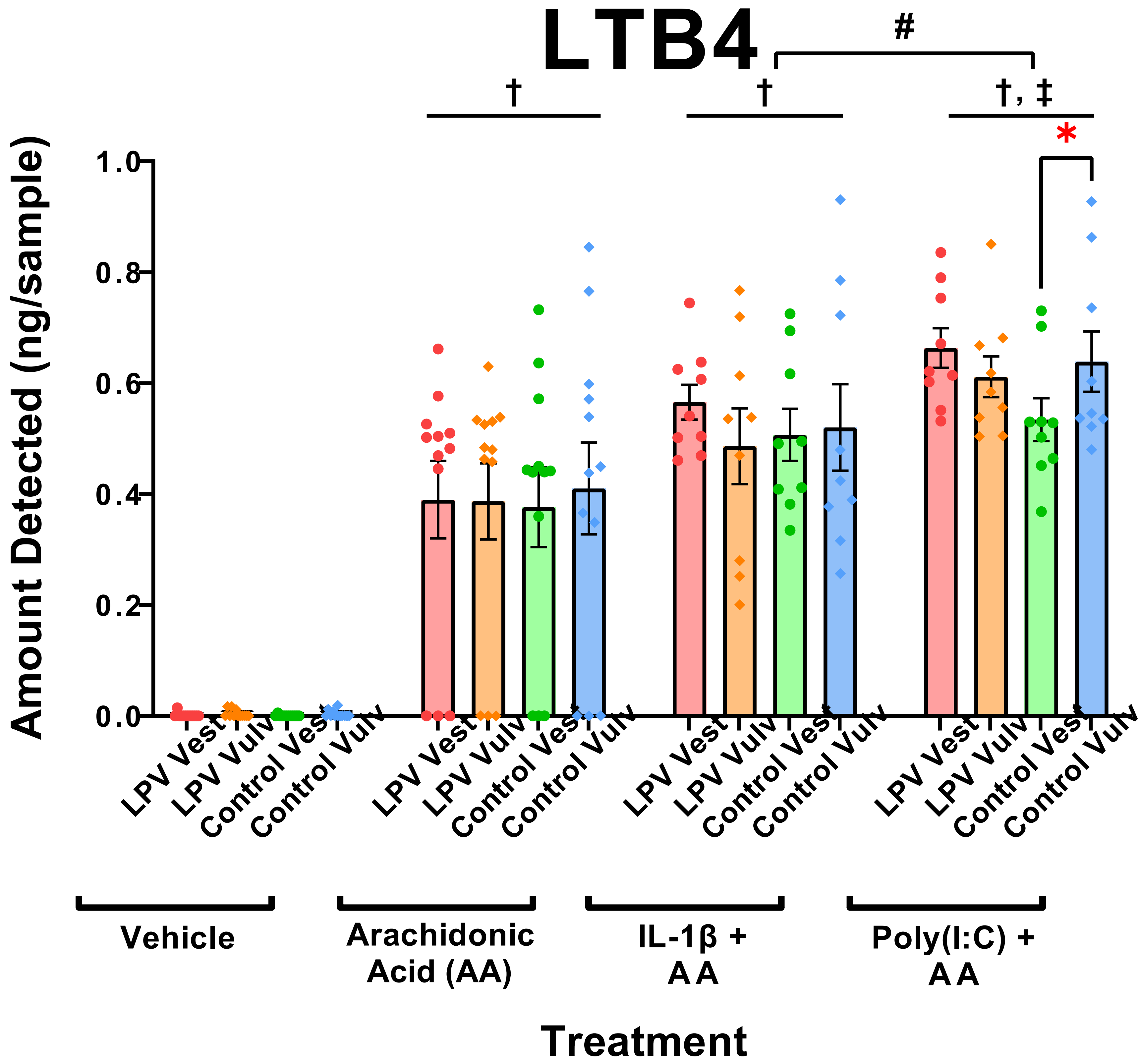
LTB4
#
†
†
†, ‡
*
Amount Detected (ng/sample)
1.0
0.8
0.6
0.4
0.2
0.0
LPV Vest
LPV Vulv
Control Vest
Control Vulv
Vehicle
Arachidonic Acid (AA)
IL-1β + AA
Poly(I:C) + AA
Treatment

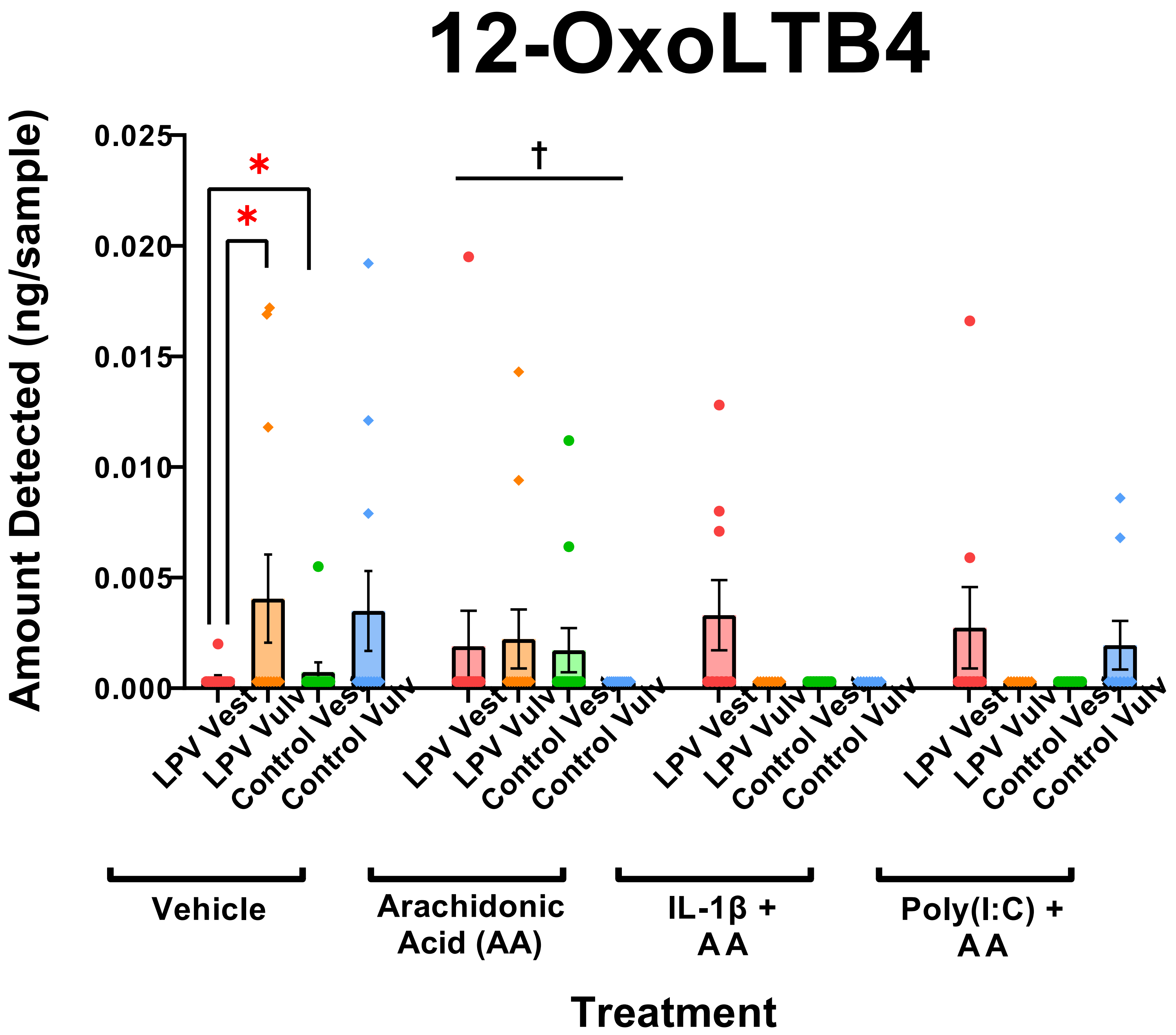

12-OxoLTB4
Amount Detected (ng/sample)
0.025
0.020
0.015
0.010
0.005
0.000
*
*
†
LPV Vest
LPV Vulv
Control Vest
Control Vulv
Vehicle
Arachidonic Acid (AA)
IL-1β + AA
Poly(I:C) + AA
Treatment

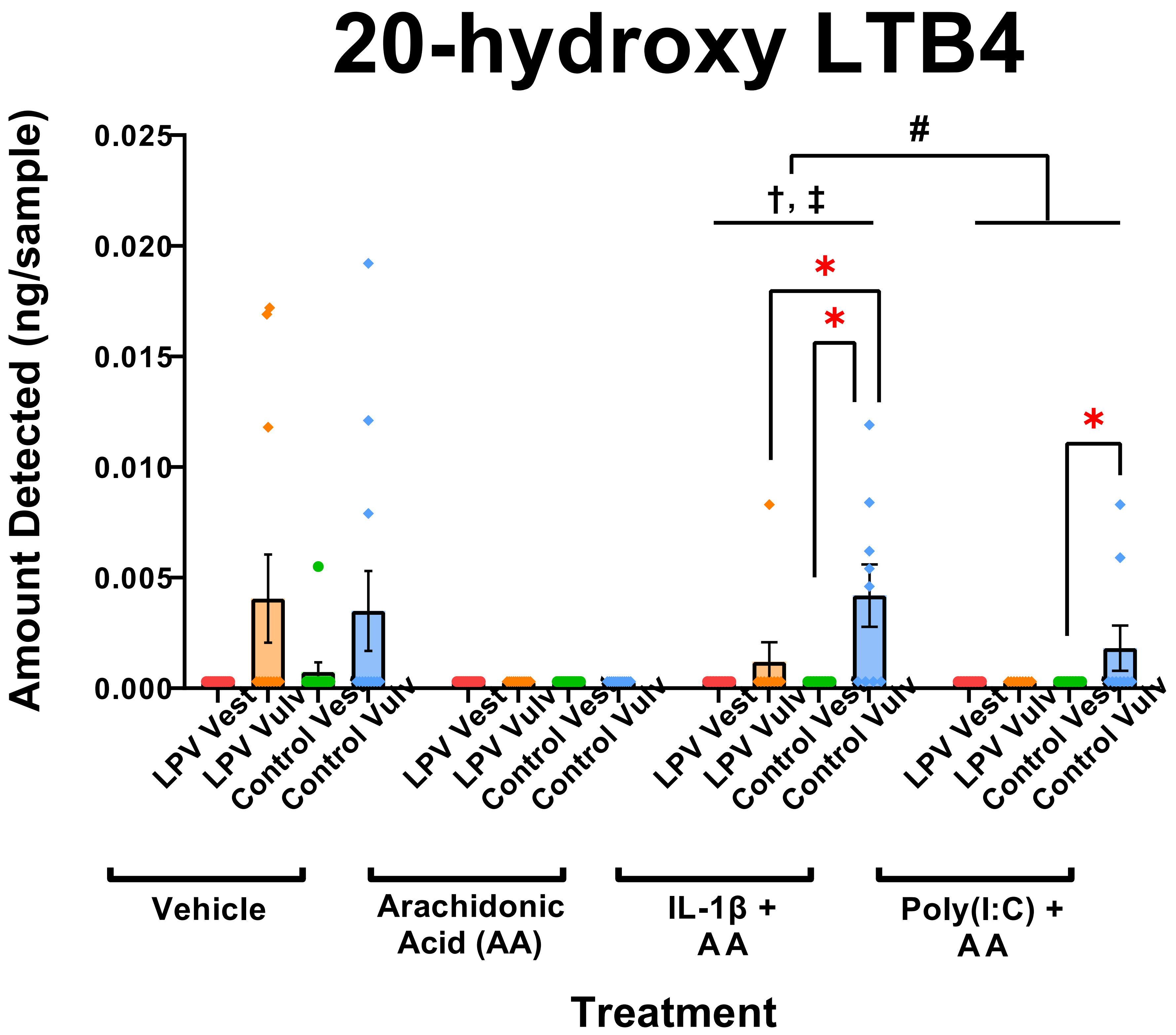

20-hydroxy LTB4
Amount Detected (ng/sample)
0.025
0.020
0.015
0.010
0.005
0.000
#
†, ‡
*
*
*
LPV Vest
LPV Vulv
Control Vest
Control Vulv
Vehicle
Arachidonic Acid (AA)
IL-1β + AA
Poly(I:C) + AA
Treatment

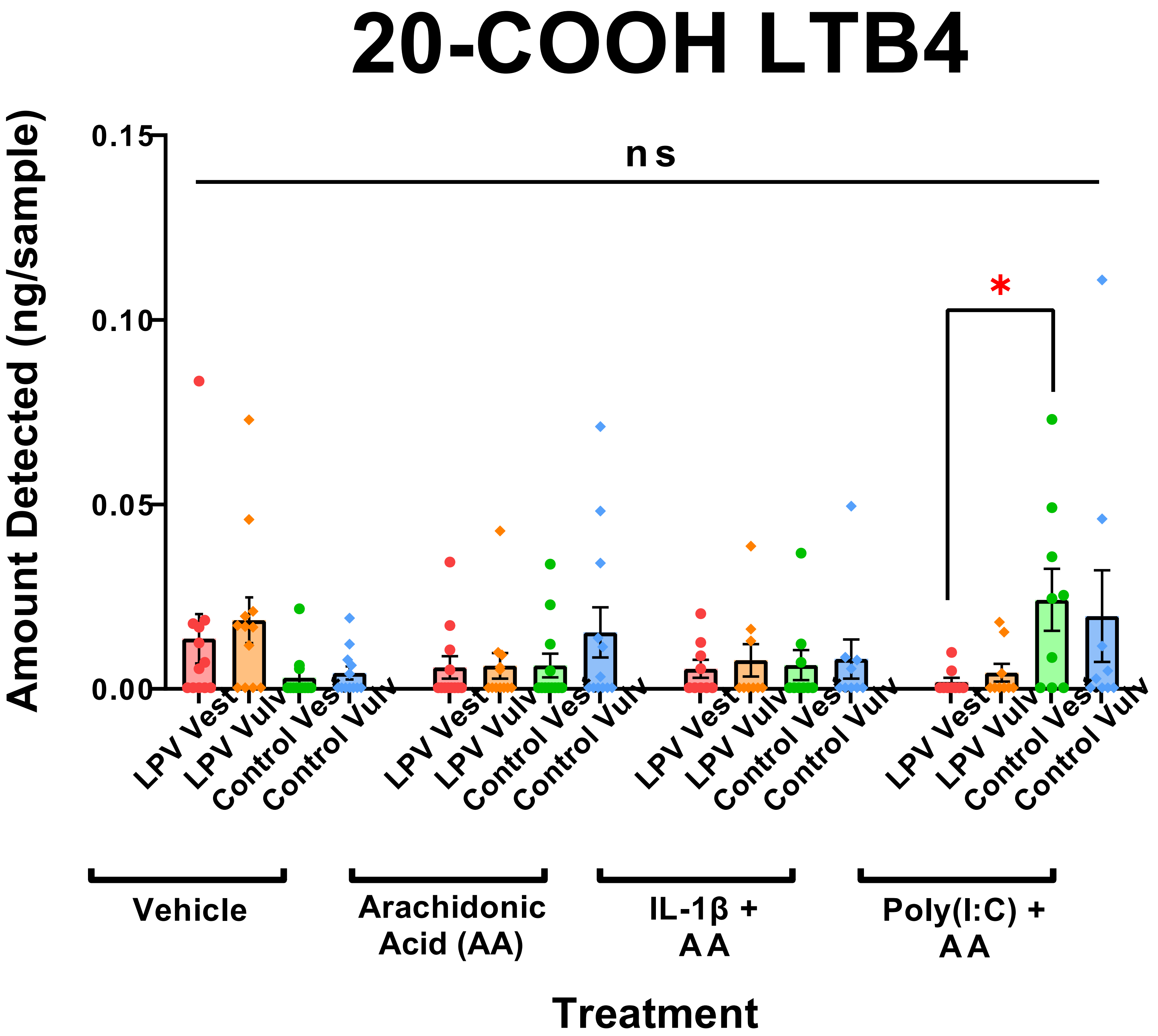

20-COOH LTB4
Amount Detected (ng/sample)
0.15
0.10
0.05
0.00
ns
*
LPV Vest
LPV Vulv
Control Vest
Control Vulv
Vehicle
Arachidonic Acid (AA)
IL-1β + AA
Poly(I:C) + AA
Treatment

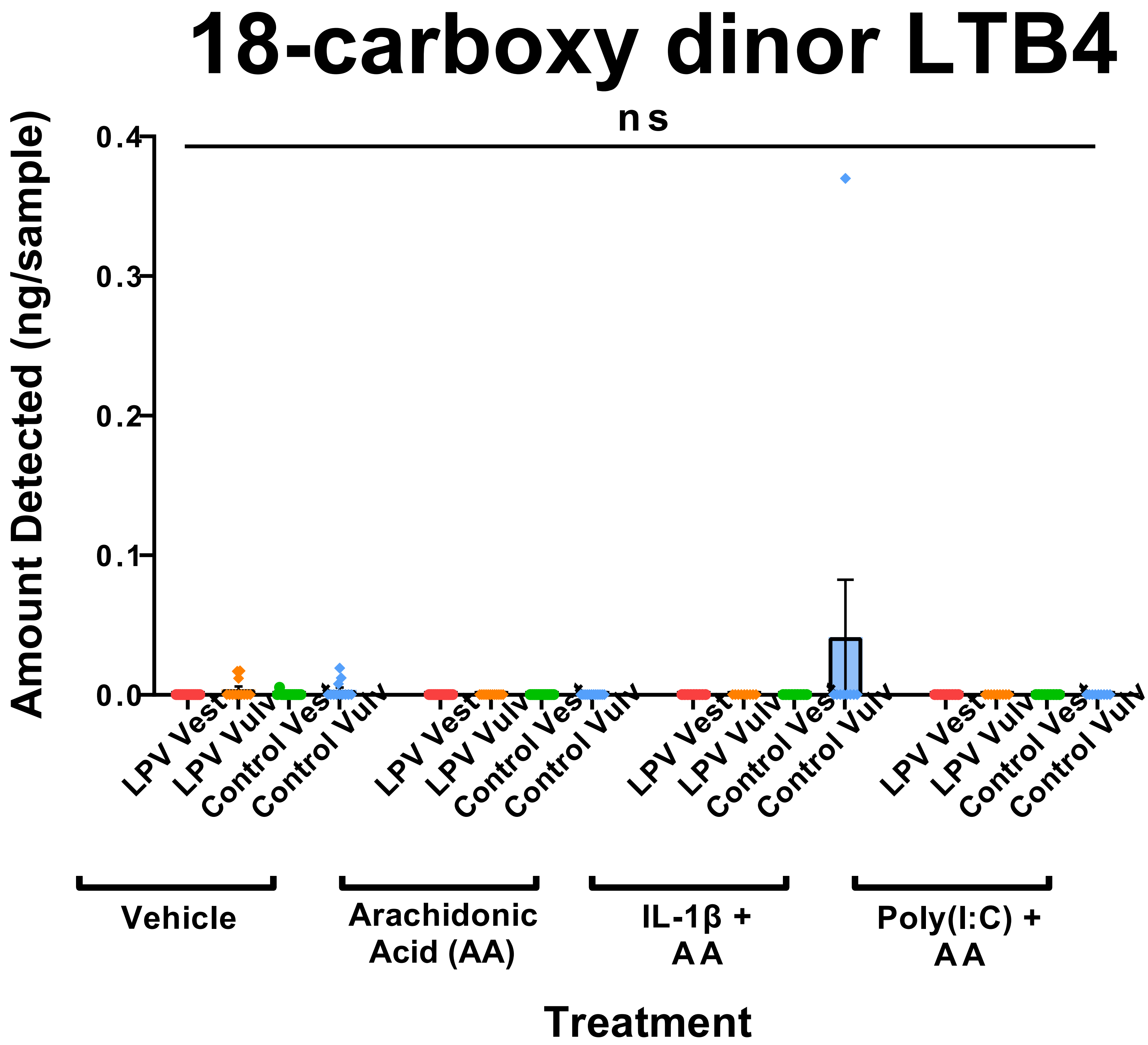

18-carboxy dinor LTB4
ns
Amount Detected (ng/sample)
0.0
0.1
0.2
0.3
0.4
LPV Vest
LPV Vulv
Control Vest
Control Vulv
Vehicle
Arachidonic Acid (AA)
IL-1β + AA
Poly(I:C) + AA
Treatment

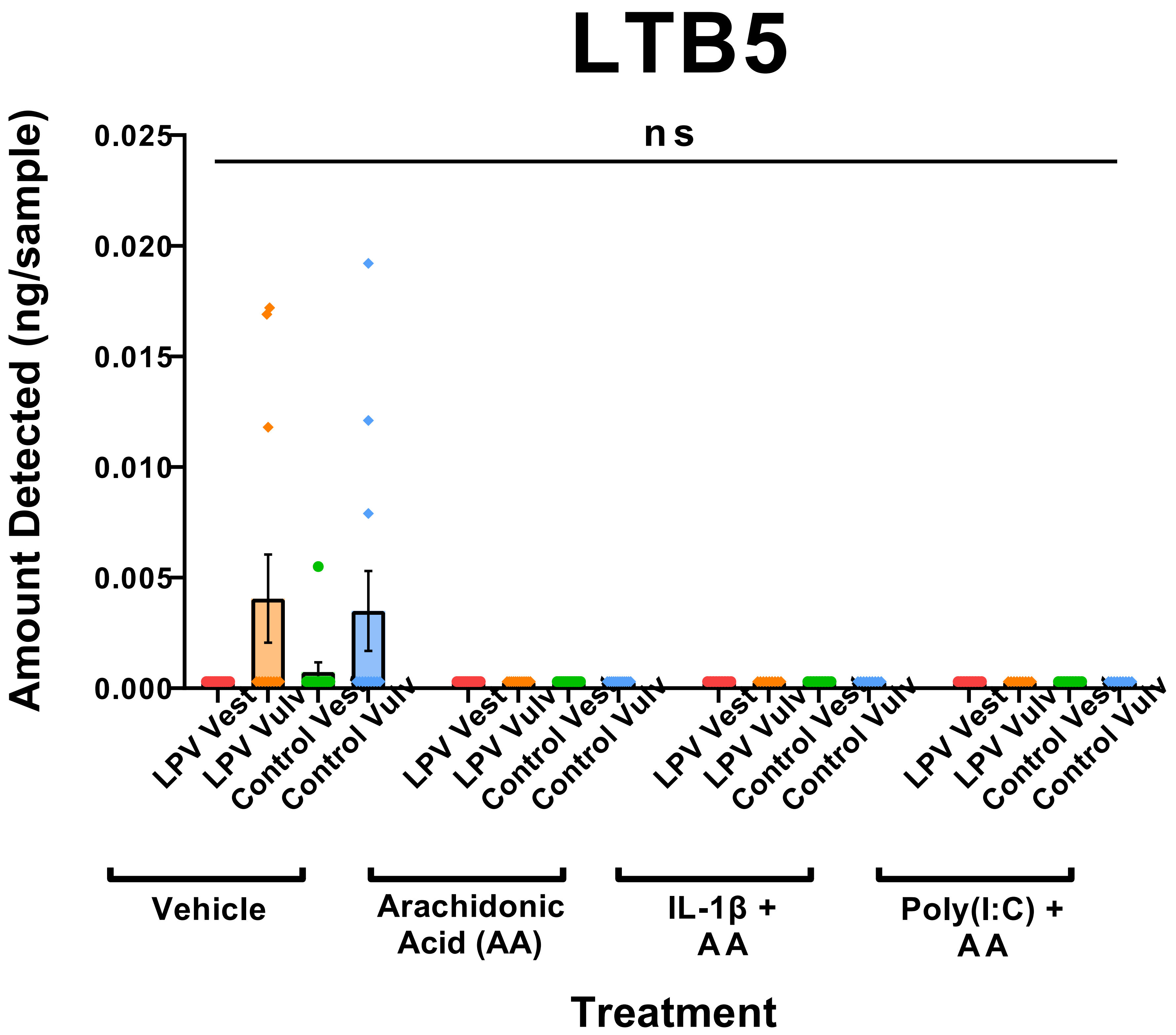
LTB5
ns
Amount Detected (ng/sample)
0.025
0.020
0.015
0.010
0.005
0.000
LPV Vest
LPV Vulv
Control Vest
Control Vulv
Vehicle
Arachidonic Acid (AA)
IL-1β + AA
Poly(I:C) + AA
Treatment

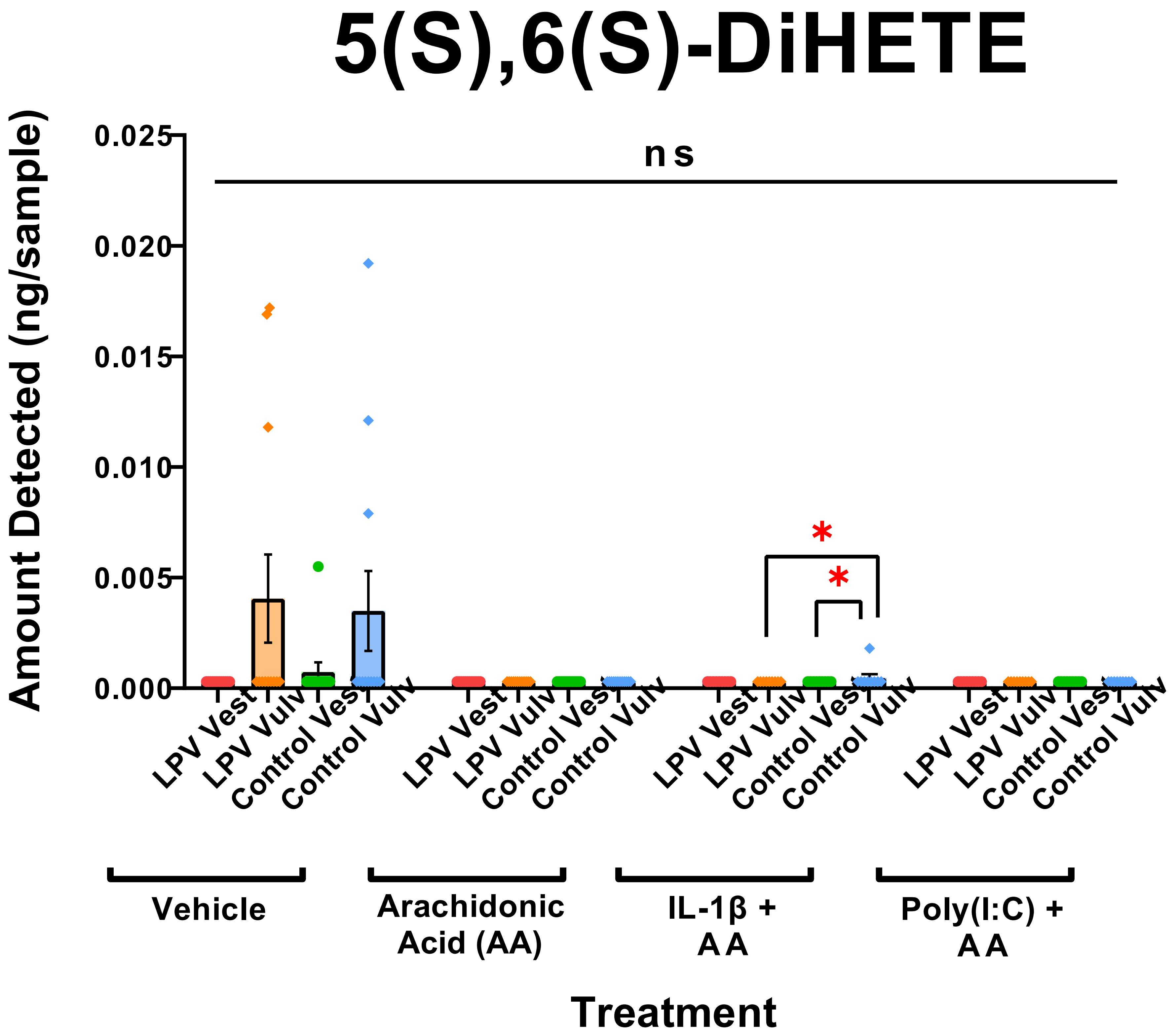

5(S),6(S)-DiHETE
Amount Detected (ng/sample)
0.025
0.020
0.015
0.010
0.005
0.000
ns
*
*
LPV Vest
LPV Vulv
Control Vest
Control Vulv
Vehicle
Arachidonic Acid (AA)
IL-1β + AA
Poly(I:C) + AA
Treatment

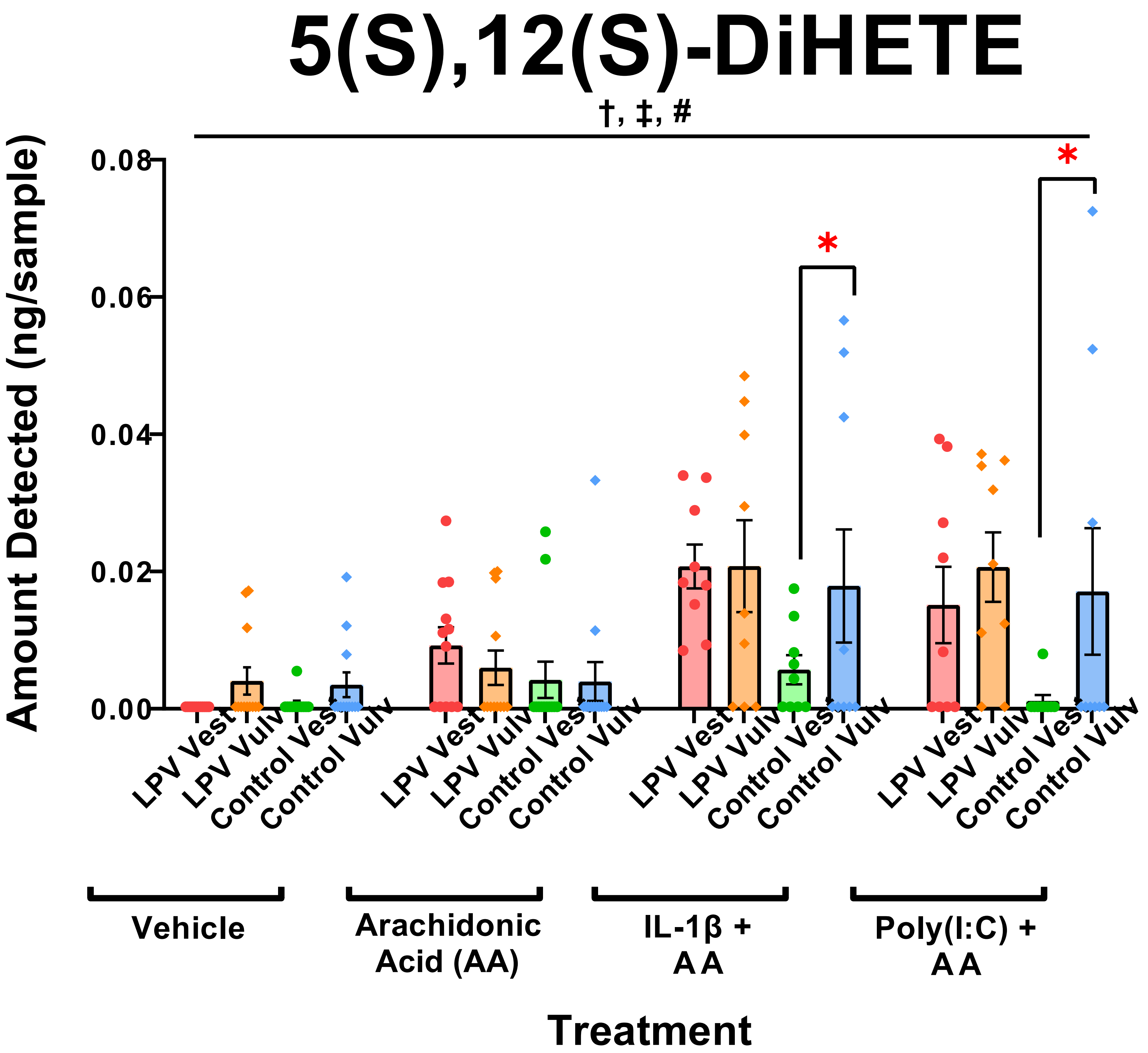

5(S),12(S)-DiHETE
†, ‡, #
Amount Detected (ng/sample)
0.08
0.06
0.04
0.02
0.00
*
*
LPV Vest
LPV Vulv
Control Vest
Control Vulv
Vehicle
Arachidonic Acid (AA)
IL-1β + AA
Poly(I:C) + AA
Treatment

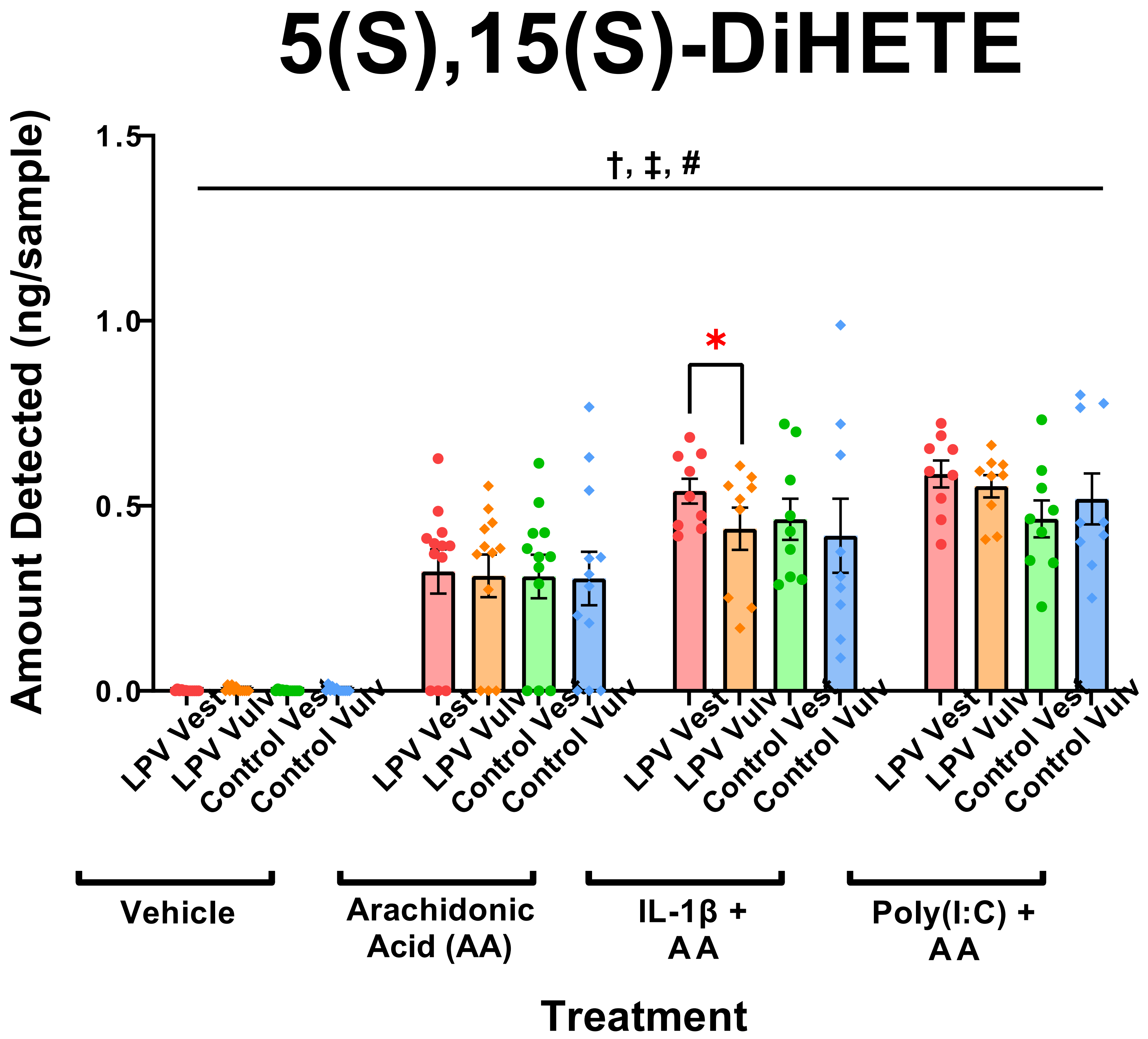

5(S),15(S)-DiHETE
Amount Detected (ng/sample)
1.5
1.0
0.5
0.0
†, ‡, #
*
LPV Vest
LPV Vulv
Control Vest
Control Vulv
Vehicle
Arachidonic Acid (AA)
IL-1β + AA
Poly(I:C) + AA
Treatment

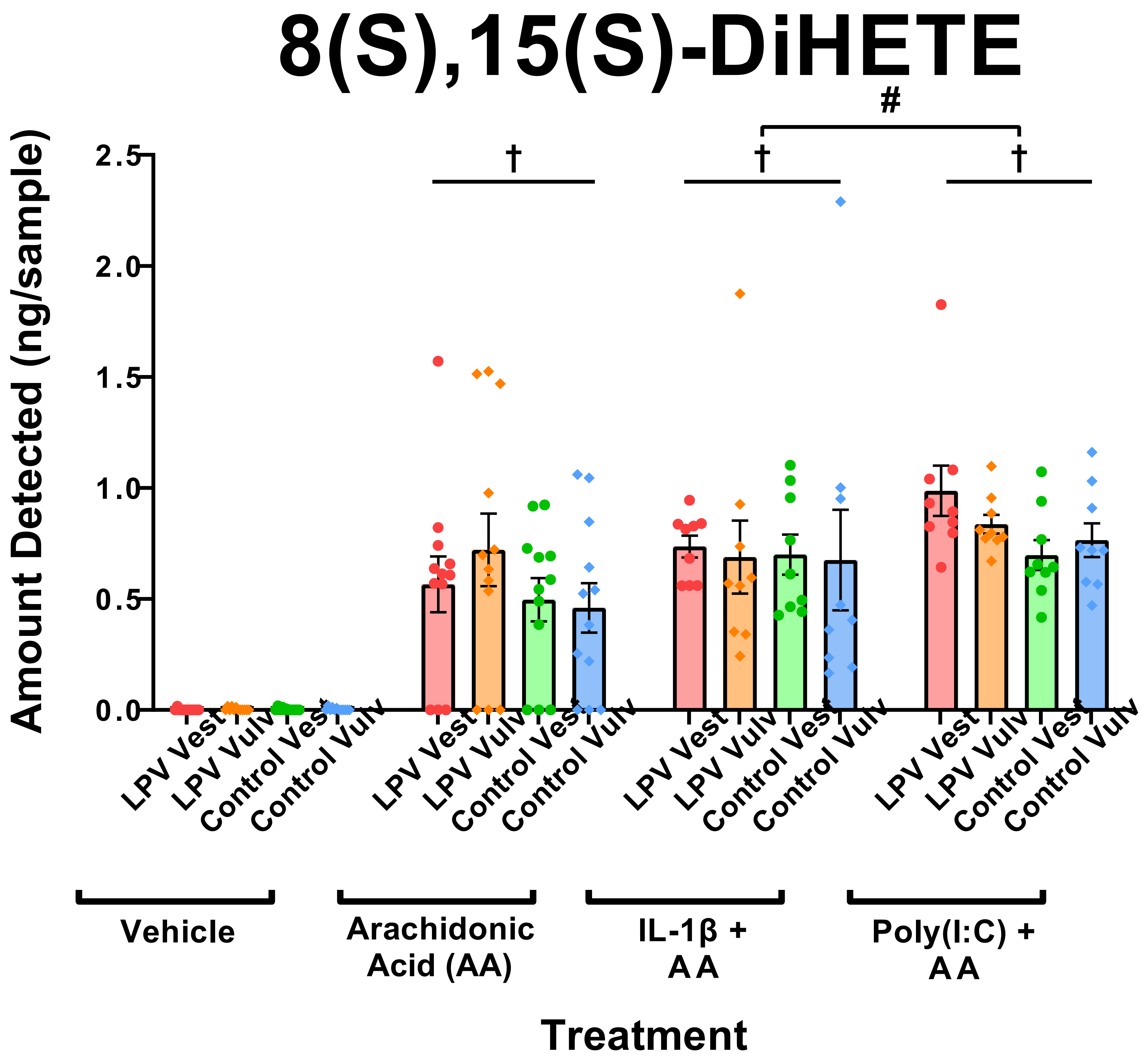
8(S),15(S)-DiHETE
#
†
†
†
Amount Detected (ng/sample)
2.5
2.0
1.5
1.0
0.5
0.0
LPV Vest
LPV Vulv
Control Vest
Control Vulv
Vehicle
Arachidonic Acid (AA)
IL-1β + AA
Poly(I:C) + AA
Treatment

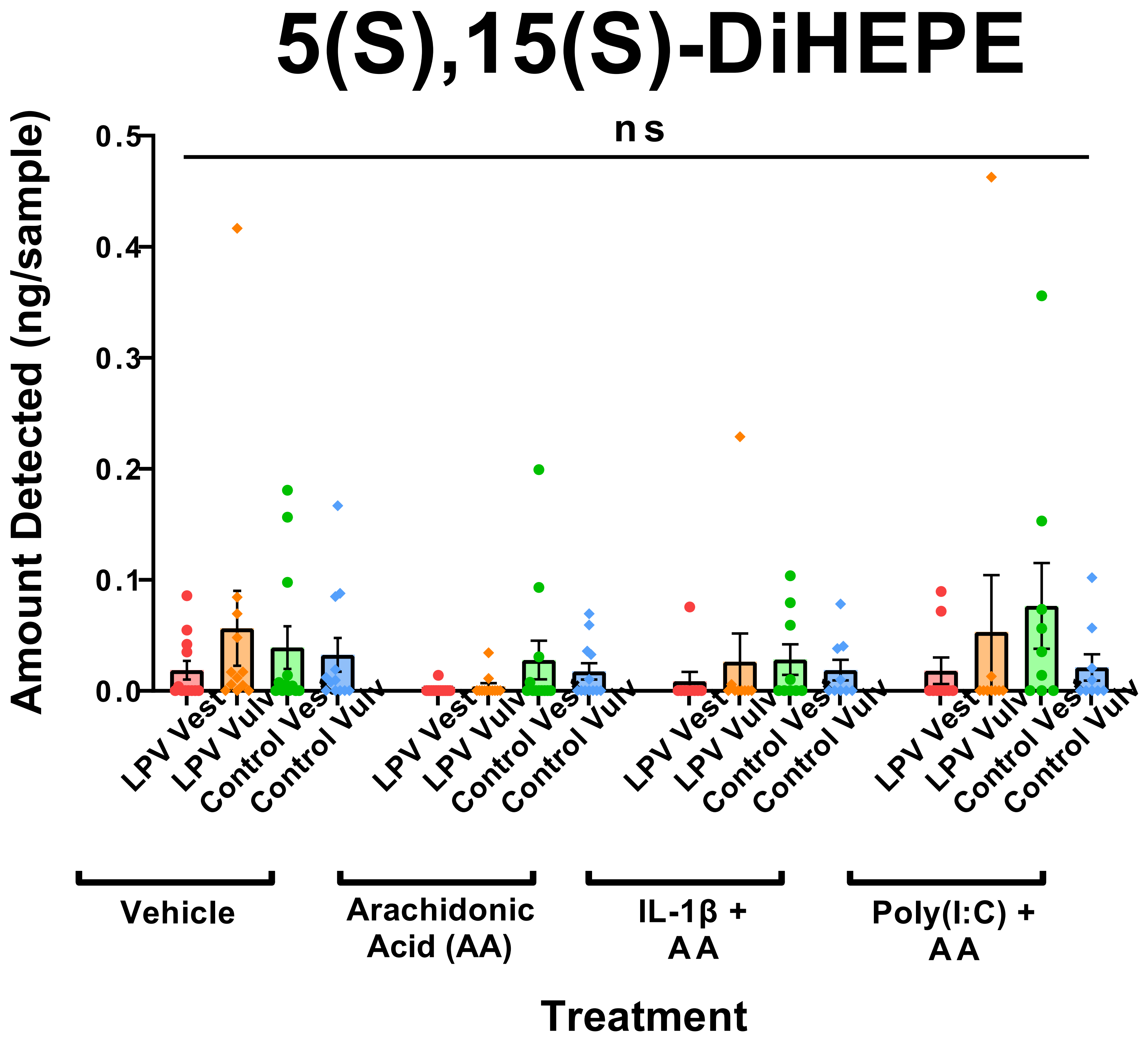

5(S),15(S)-DiHEPE
Amount Detected (ng/sample)
0.0
0.1
0.2
0.3
0.4
0.5
ns
LPV Vest
LPV Vulv
Control Vest
Control Vulv
Vehicle
Arachidonic Acid (AA)
IL-1β + AA
Poly(I:C) + AA
Treatment

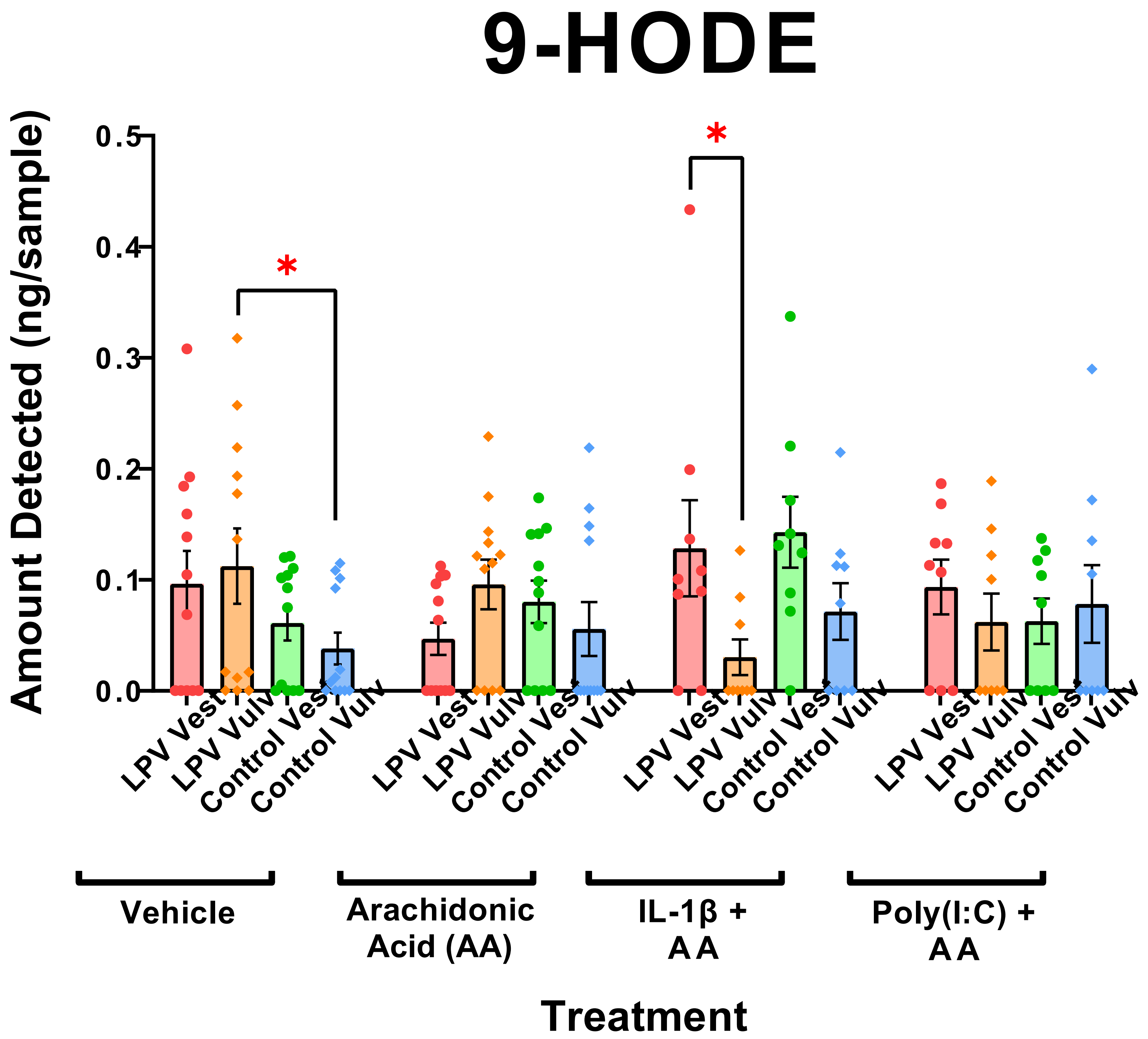

9-HODE
Amount Detected (ng/sample)
0.5
0.4
0.3
0.2
0.1
0.0
*
*
LPV Vest
LPV Vulv
Control Vest
Control Vulv
Vehicle
Arachidonic Acid (AA)
IL-1β + AA
Poly(I:C) + AA
Treatment

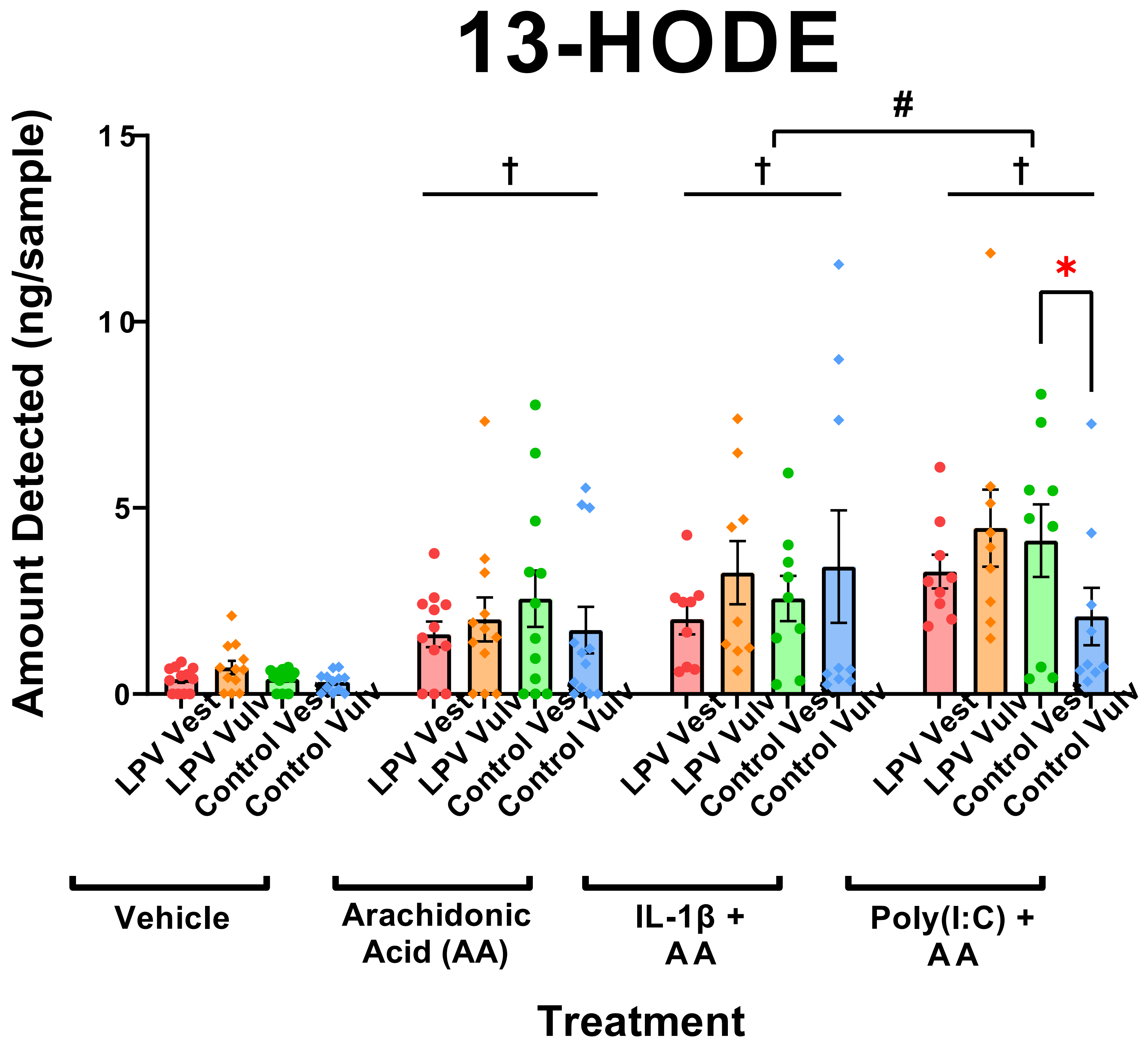
13-HODE
Amount Detected (ng/sample)
15
10
5
0
#
†
†
†
*
LPV Vest
LPV Vulv
Control Vest
Control Vulv
Vehicle
Arachidonic Acid (AA)
IL-1β + AA
Poly(I:C) + AA
Treatment

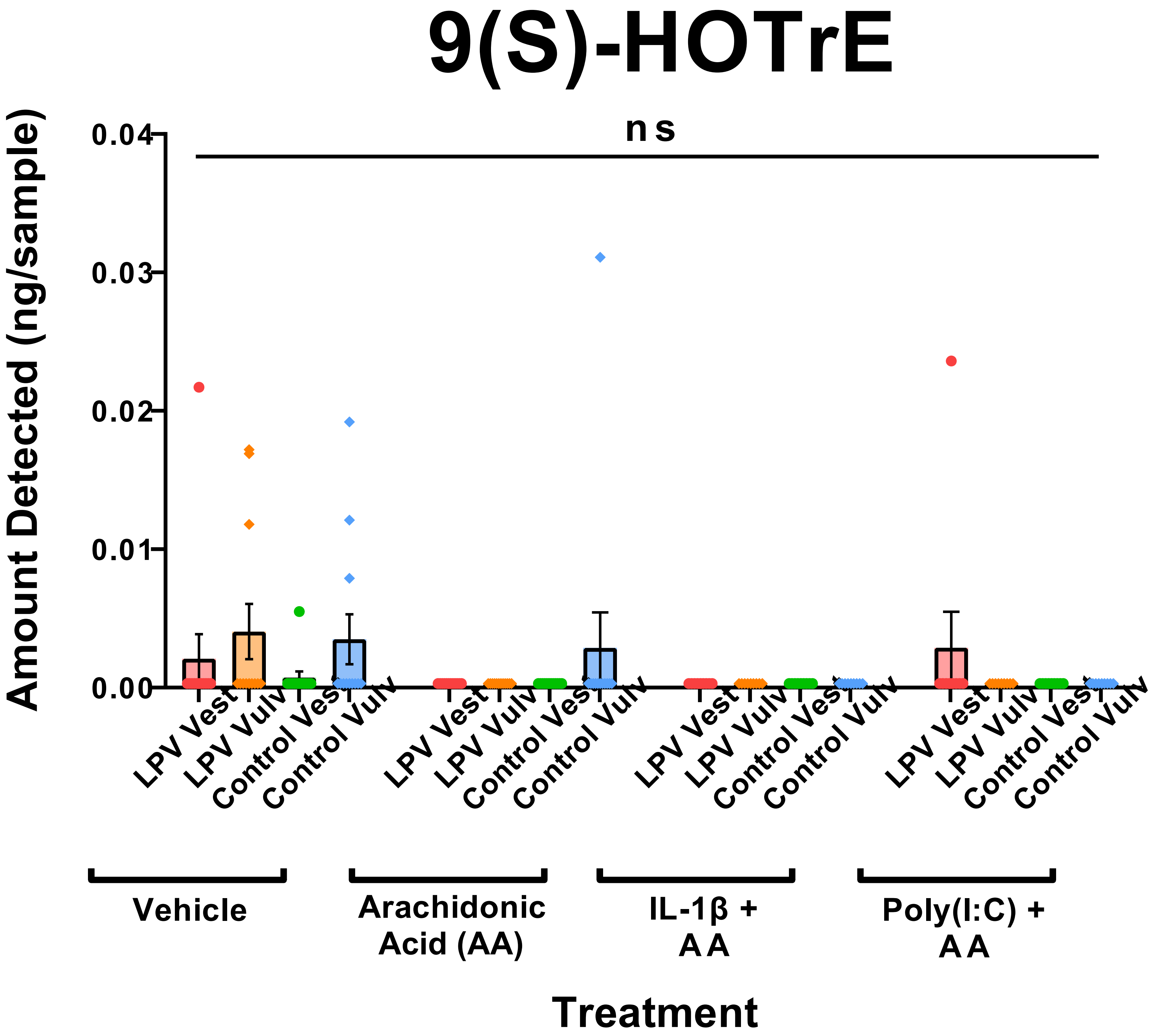

9(S)-HOTrE
ns
Amount Detected (ng/sample)
0.04
0.03
0.02
0.01
0.00
LPV Vest
LPV Vulv
Control Vest
Control Vulv
Vehicle
Arachidonic Acid (AA)
IL-1β + AA
Poly(I:C) + AA
Treatment

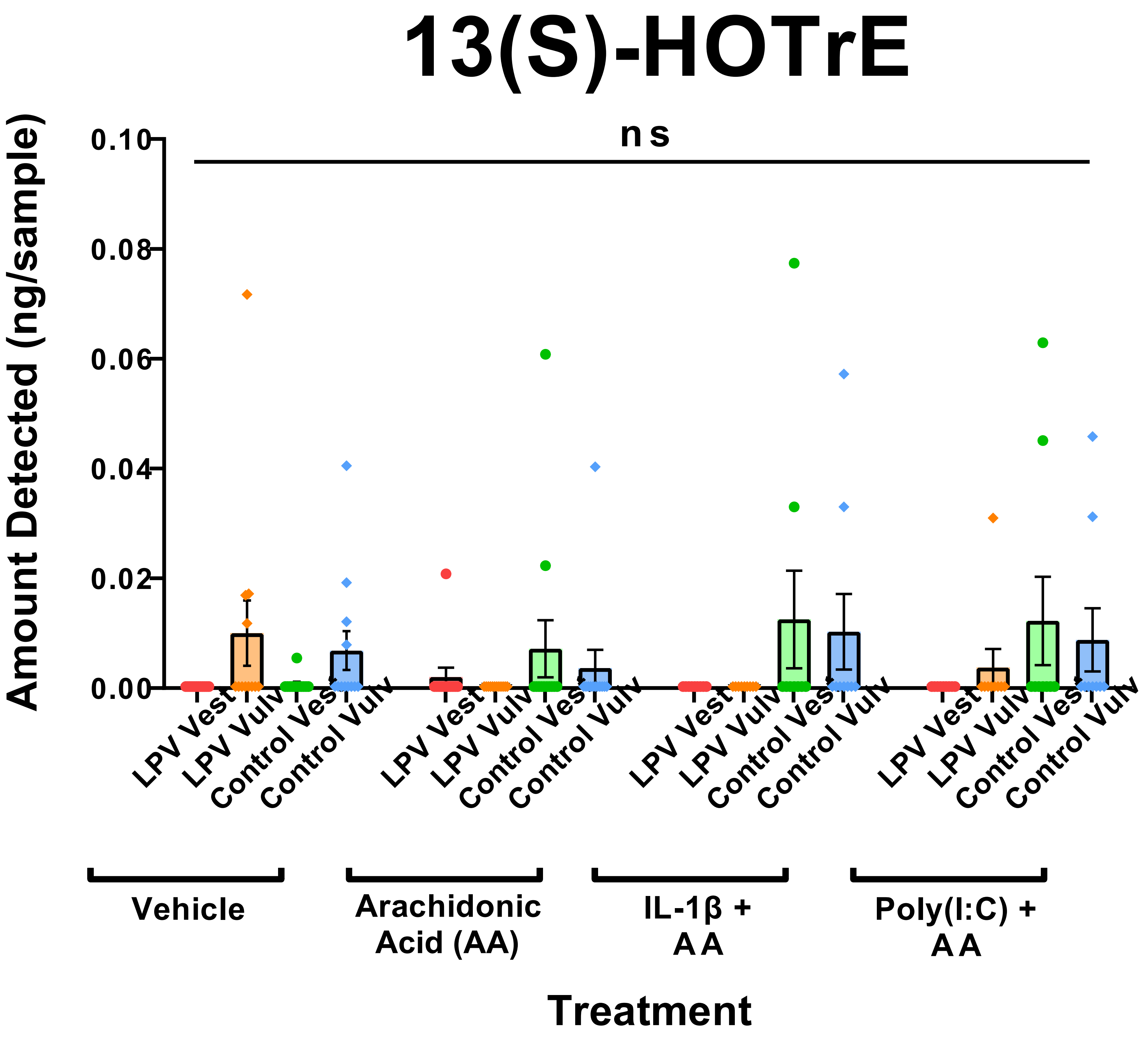
13(S)-HOTrE
Amount Detected (ng/sample)
0.10
0.08
0.06
0.04
0.02
0.00
ns
LPV Vest
LPV Vulv
Control Vest
Control Vulv
Vehicle
Arachidonic Acid (AA)
IL-1β + AA
Poly(I:C) + AA
Treatment

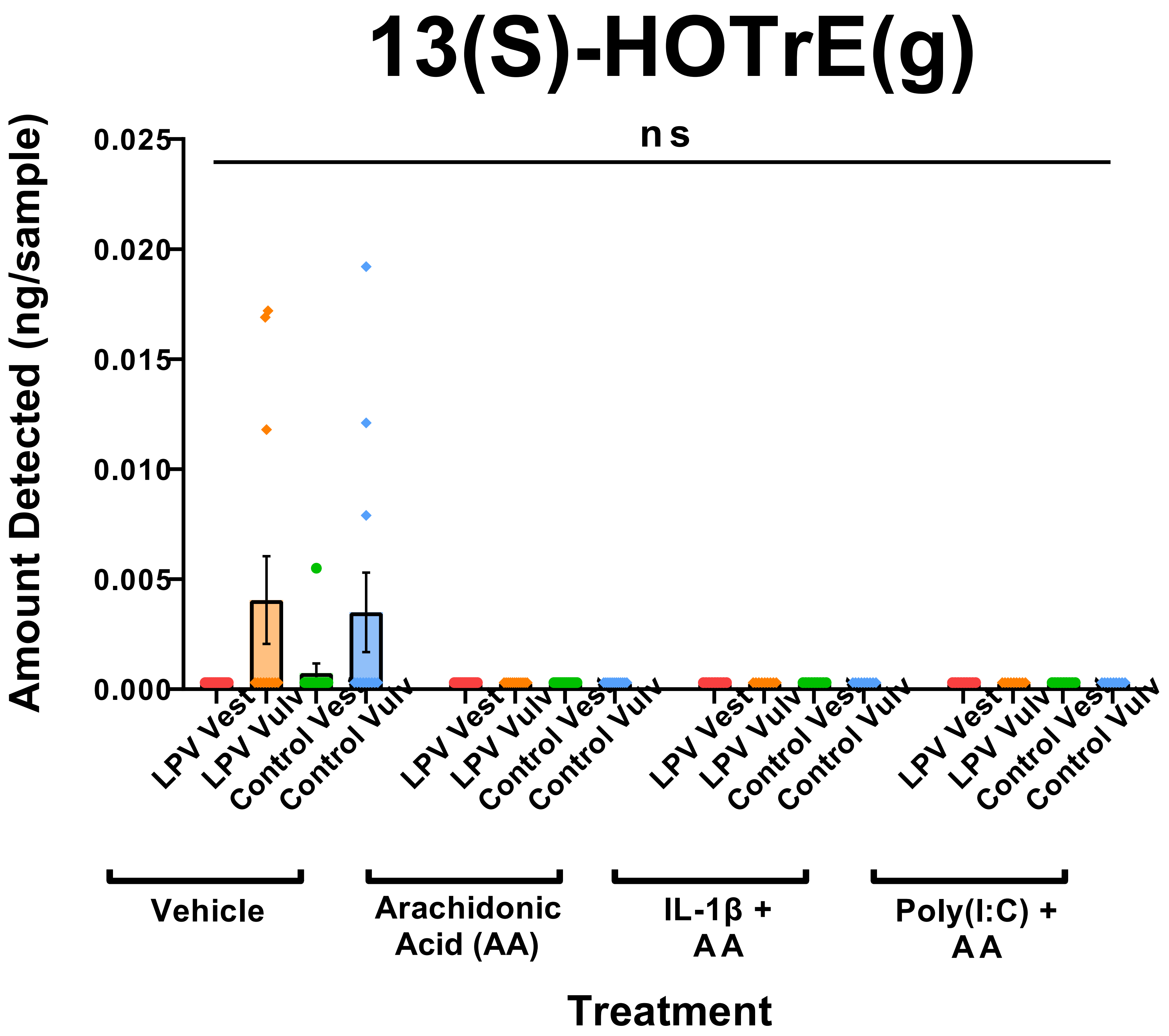
13(S)-HOTrE(g)
ns
Amount Detected (ng/sample)
0.025
0.020
0.015
0.010
0.005
0.000
LPV Vest
LPV Vulv
Control Vest
Control Vulv
Vehicle
Arachidonic Acid (AA)
IL-1β + AA
Poly(I:C) + AA
Treatment

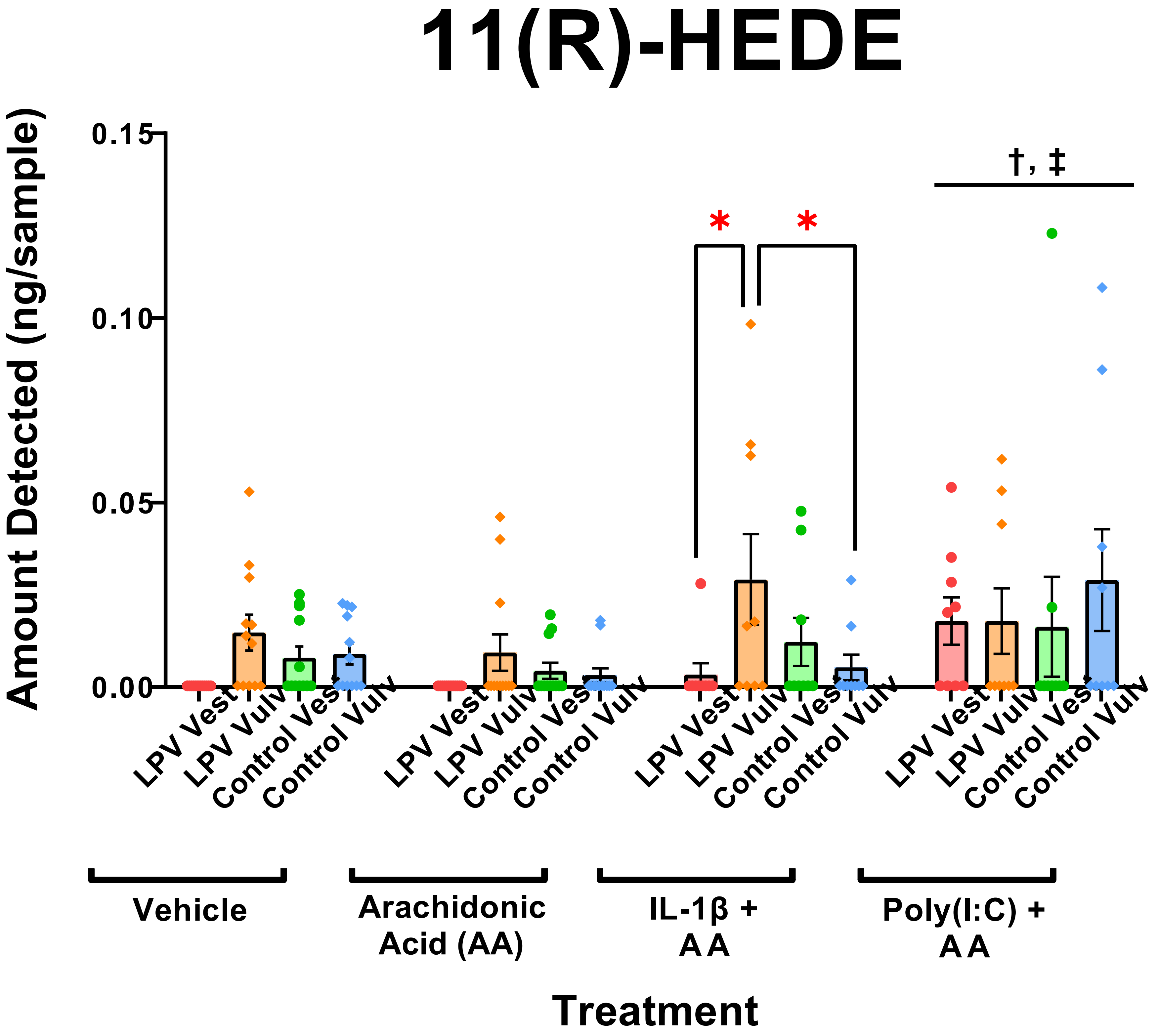

11(R)-HEDE
Amount Detected (ng/sample)
0.15
0.10
0.05
0.00
*
*
†, ‡
LPV Vest
LPV Vulv
Control Vest
Control Vulv
Vehicle
Arachidonic Acid (AA)
IL-1β + AA
Poly(I:C) + AA
Treatment

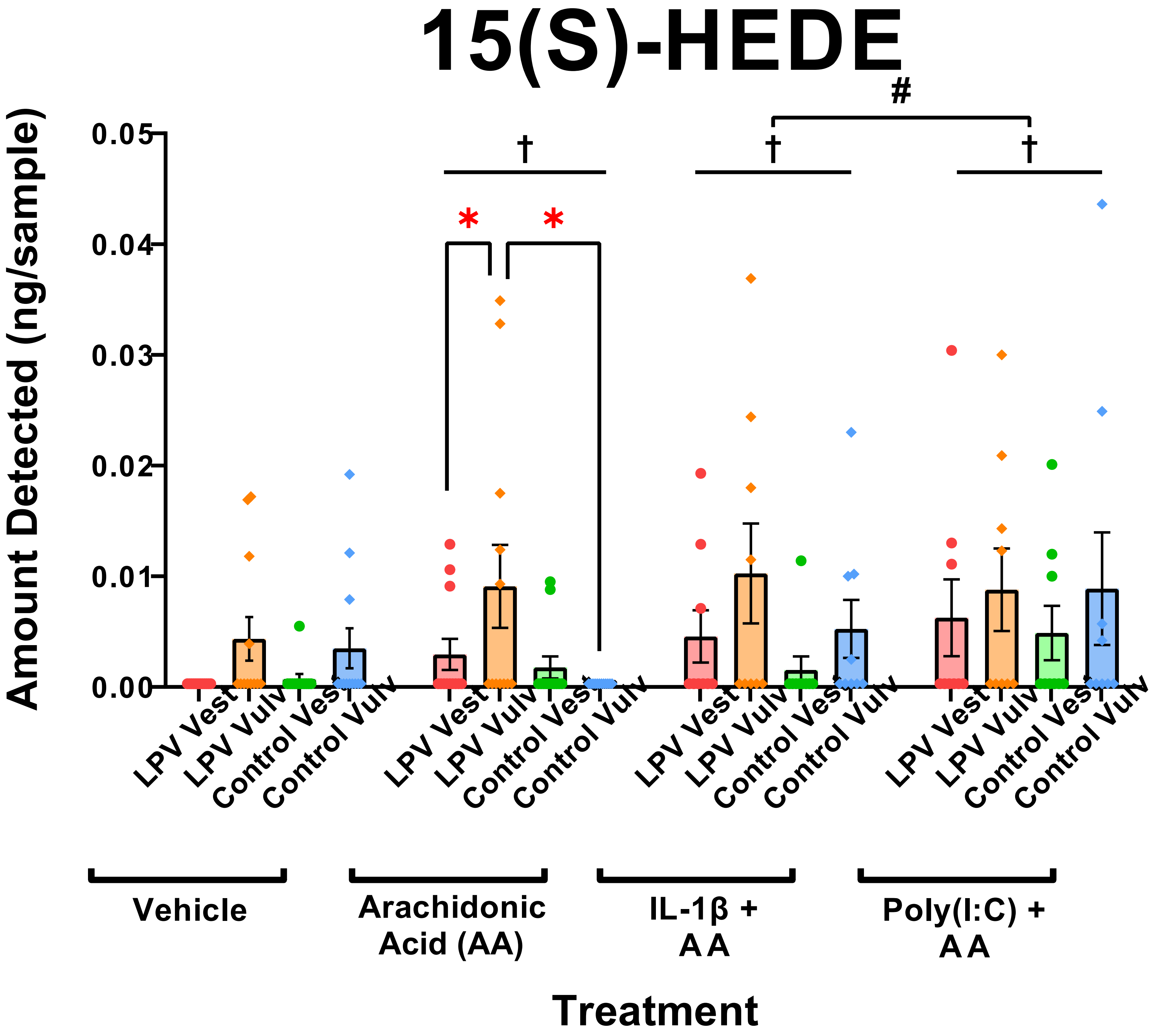
15(S)-HEDE
Amount Detected (ng/sample)
0.05
0.04
0.03
0.02
0.01
0.00
#
†
†
†
*
*
LPV Vest
LPV Vulv
Control Vest
Control Vulv
Vehicle
Arachidonic Acid (AA)
IL-1β + AA
Poly(I:C) + AA
Treatment

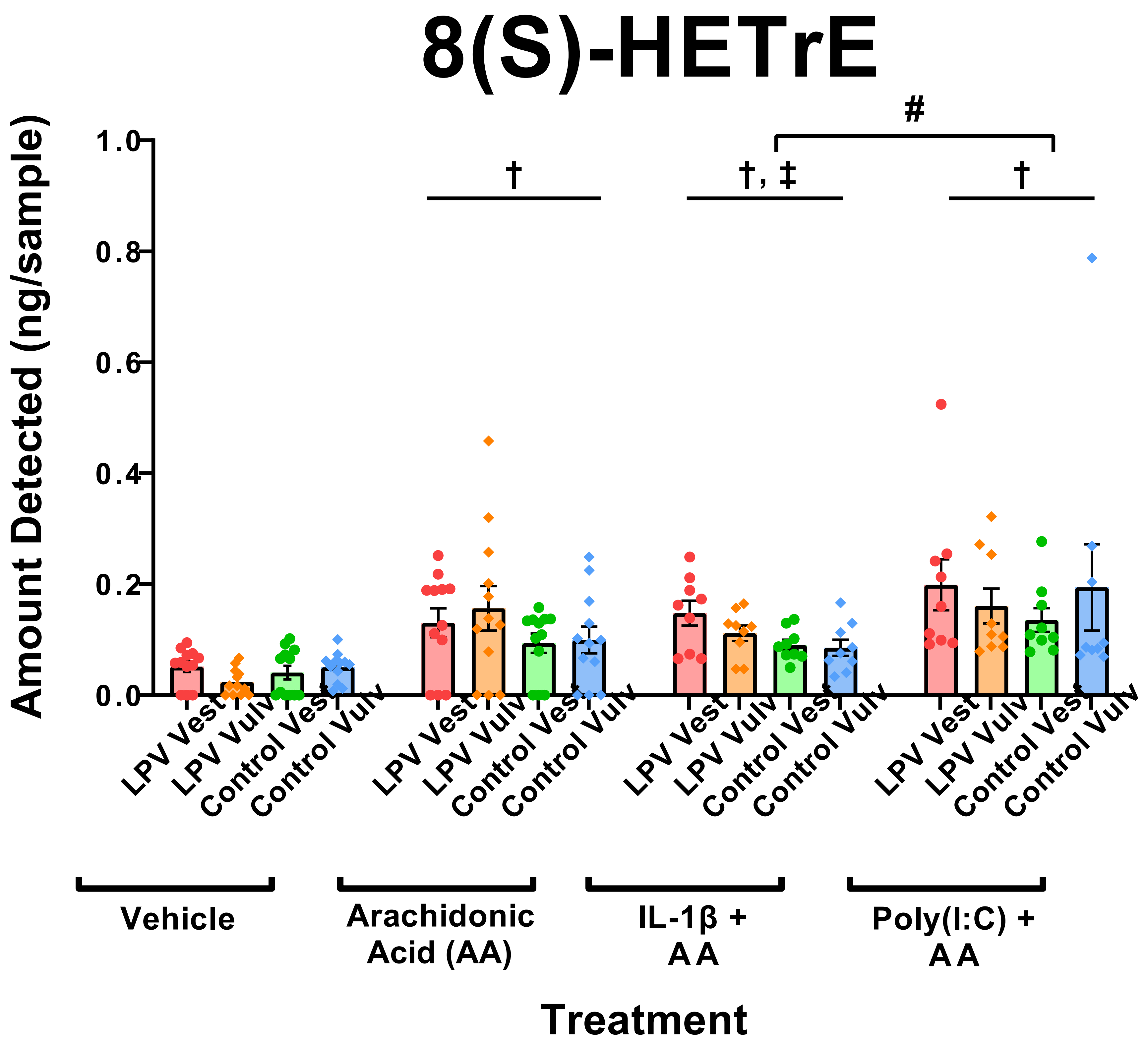

8(S)-HETrE
Amount Detected (ng/sample)
1.0
0.8
0.6
0.4
0.2
0.0
#
†
†, ‡
†
LPV Vest
LPV Vulv
Control Vest
Control Vulv
Vehicle
Arachidonic Acid (AA)
IL-1β + AA
Poly(I:C) + AA
Treatment

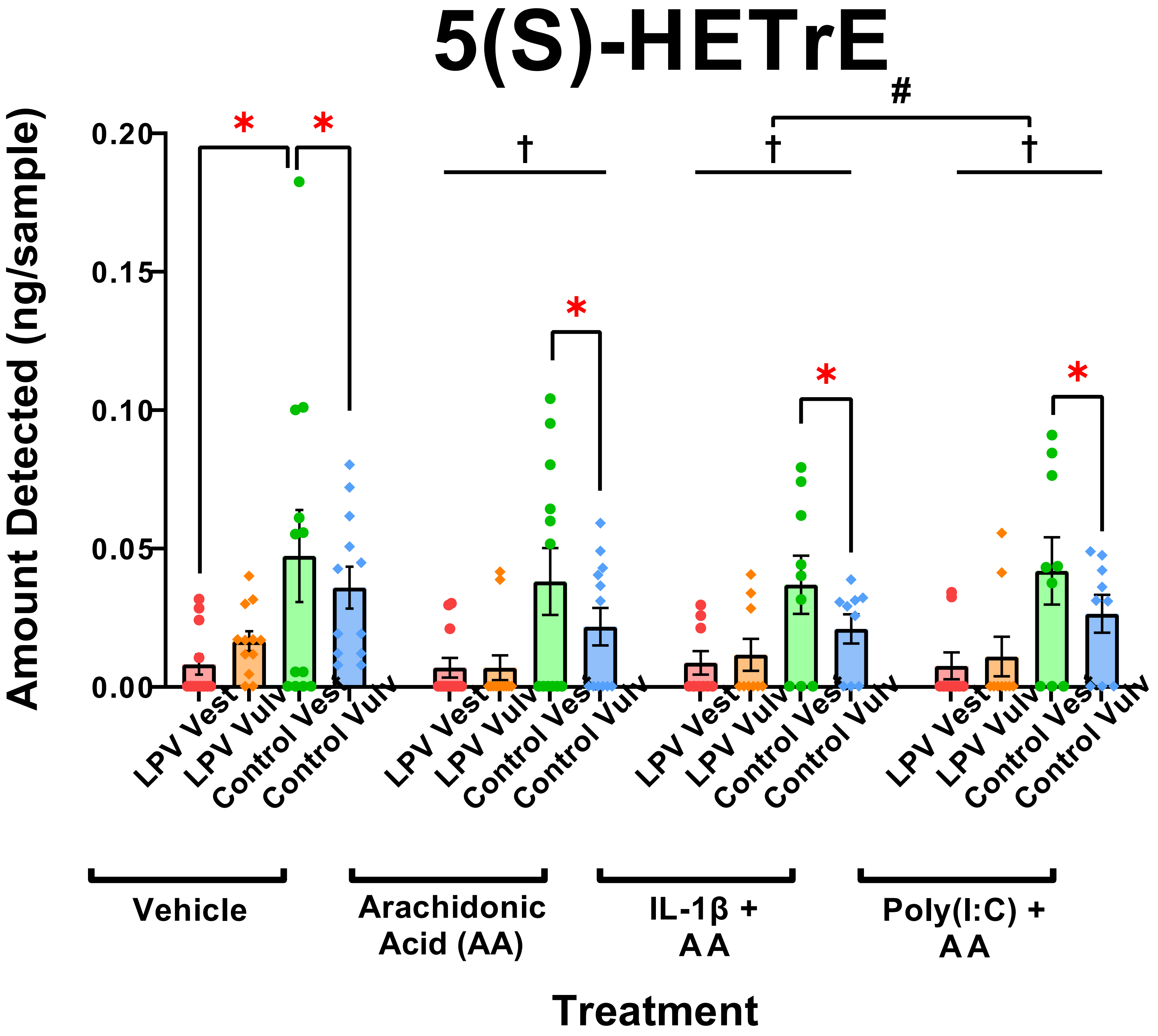

5(S)-HETrE
Amount Detected (ng/sample)
0.20
0.15
0.10
0.05
0.00
*
†
#
LPV Vest
LPV Vulv
Control Vest
Control Vulv
Vehicle
Arachidonic Acid (AA)
IL-1β + AA
Poly(I:C) + AA
Treatment

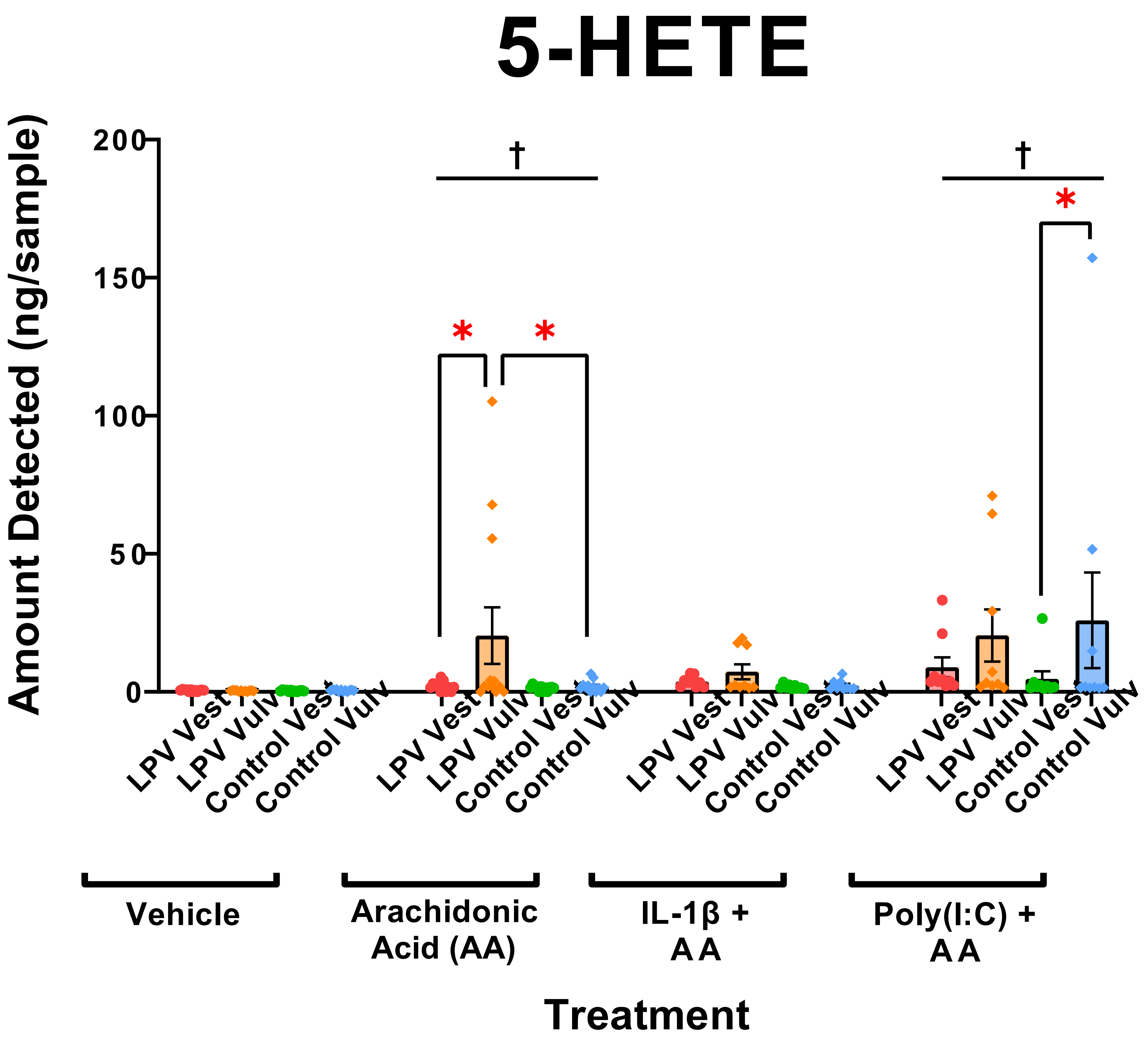

5-HETE
Amount Detected (ng/sample)
200
150
100
50
0
†
†
*
*
*
LPV Vest
LPV Vulv
Control Vest
Control Vulv
Vehicle
Arachidonic Acid (AA)
IL-1β + AA
Poly(I:C) + AA
Treatment

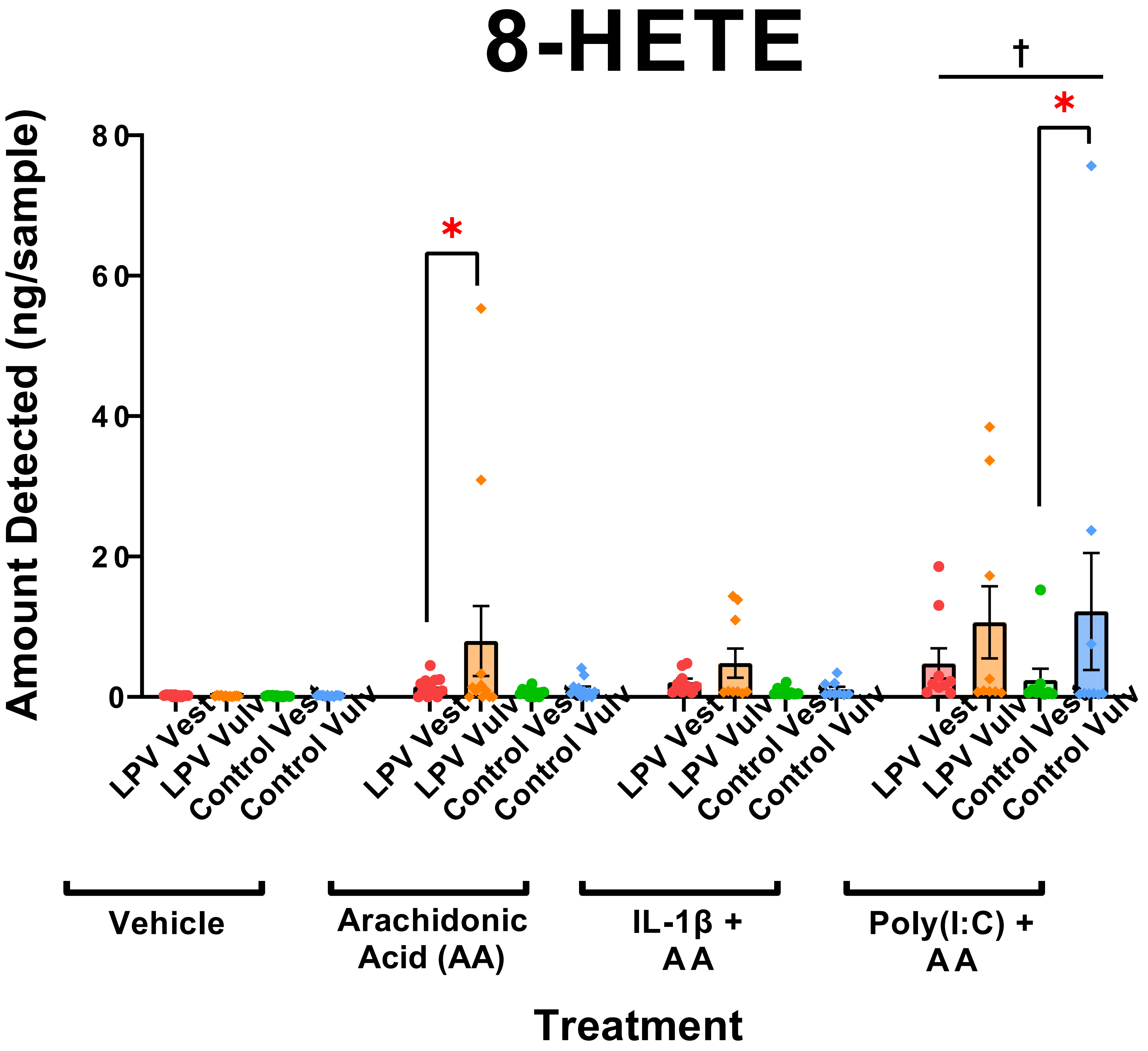

8-HETE
†
*
*
Amount Detected (ng/sample)
80
60
40
20
0
LPV Vest
LPV Vulv
Control Vest
Control Vulv
Vehicle
Arachidonic Acid (AA)
IL-1β + AA
Poly(I:C) + AA
Treatment

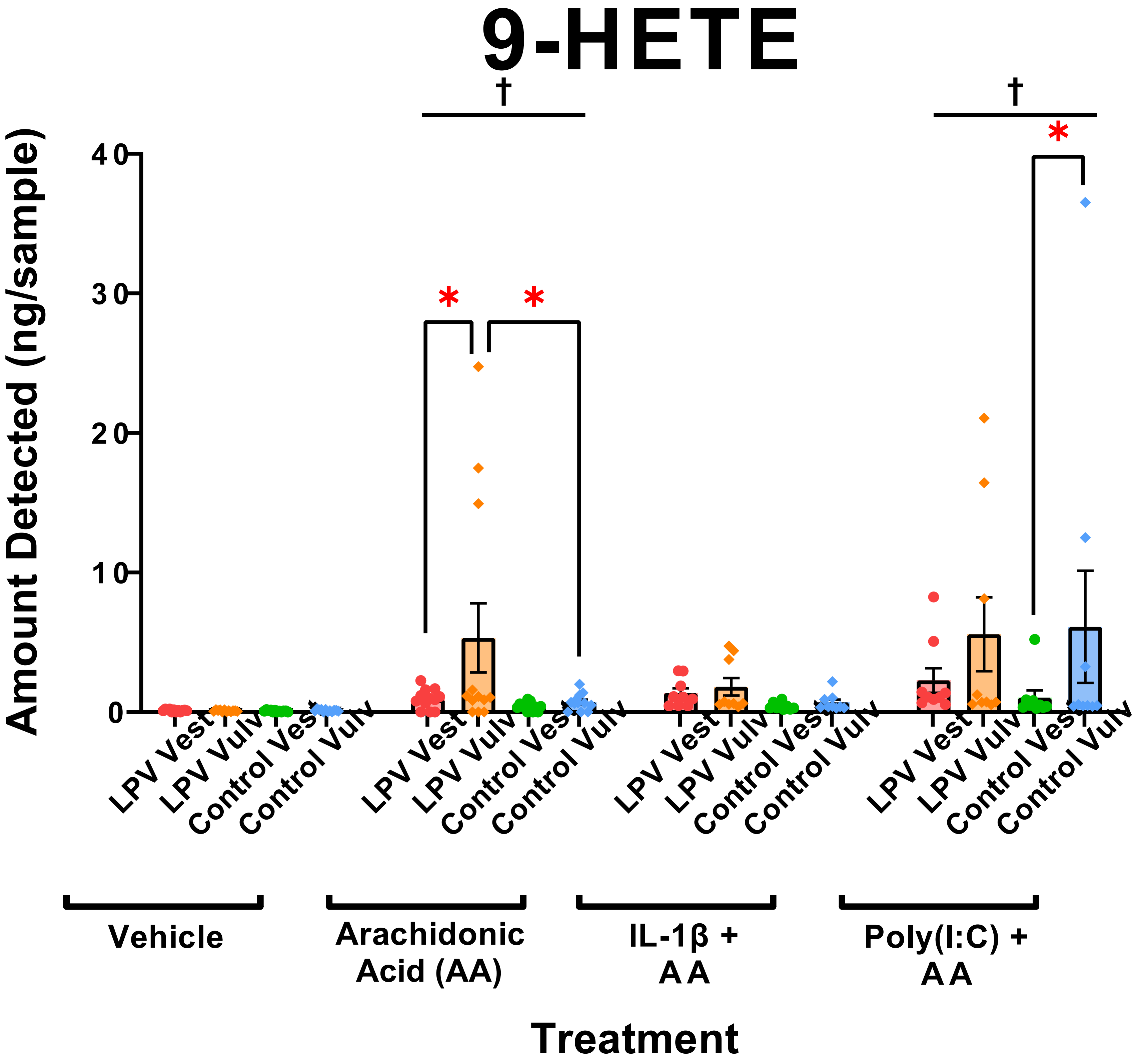

9-HETE
†
†
*
*
*
Amount Detected (ng/sample)
40
30
20
10
0
LPV Vest
LPV Vulv
Control Vest
Control Vulv
Vehicle
Arachidonic Acid (AA)
IL-1β + AA
Poly(I:C) + AA
Treatment

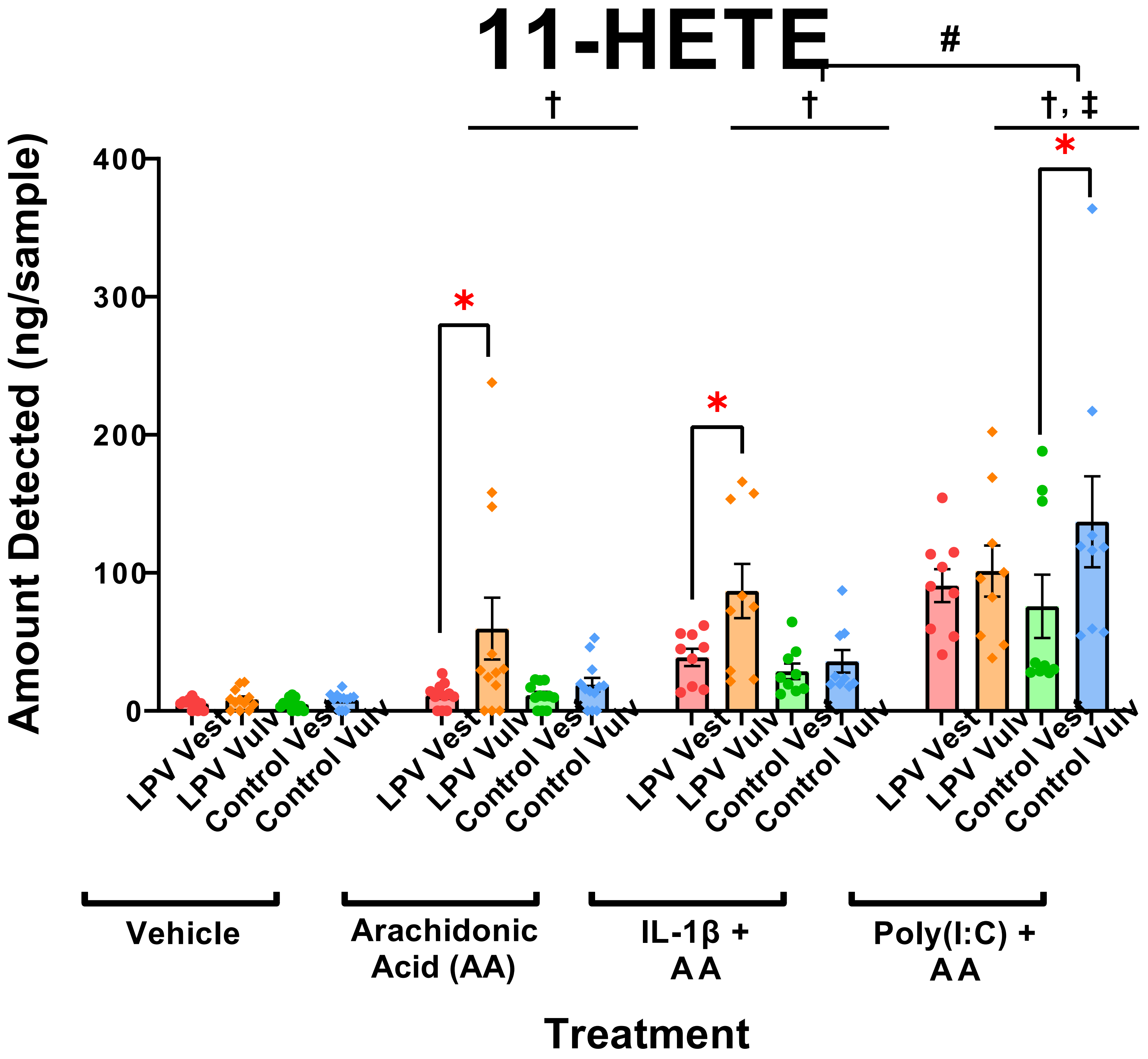

11-HETE
#
†
†
†, ‡
*
*
*
Amount Detected (ng/sample)
400
300
200
100
0
LPV Vest
LPV Vulv
Control Vest
Control Vulv
Vehicle
Arachidonic Acid (AA)
IL-1β + AA
Poly(I:C) + AA
Treatment

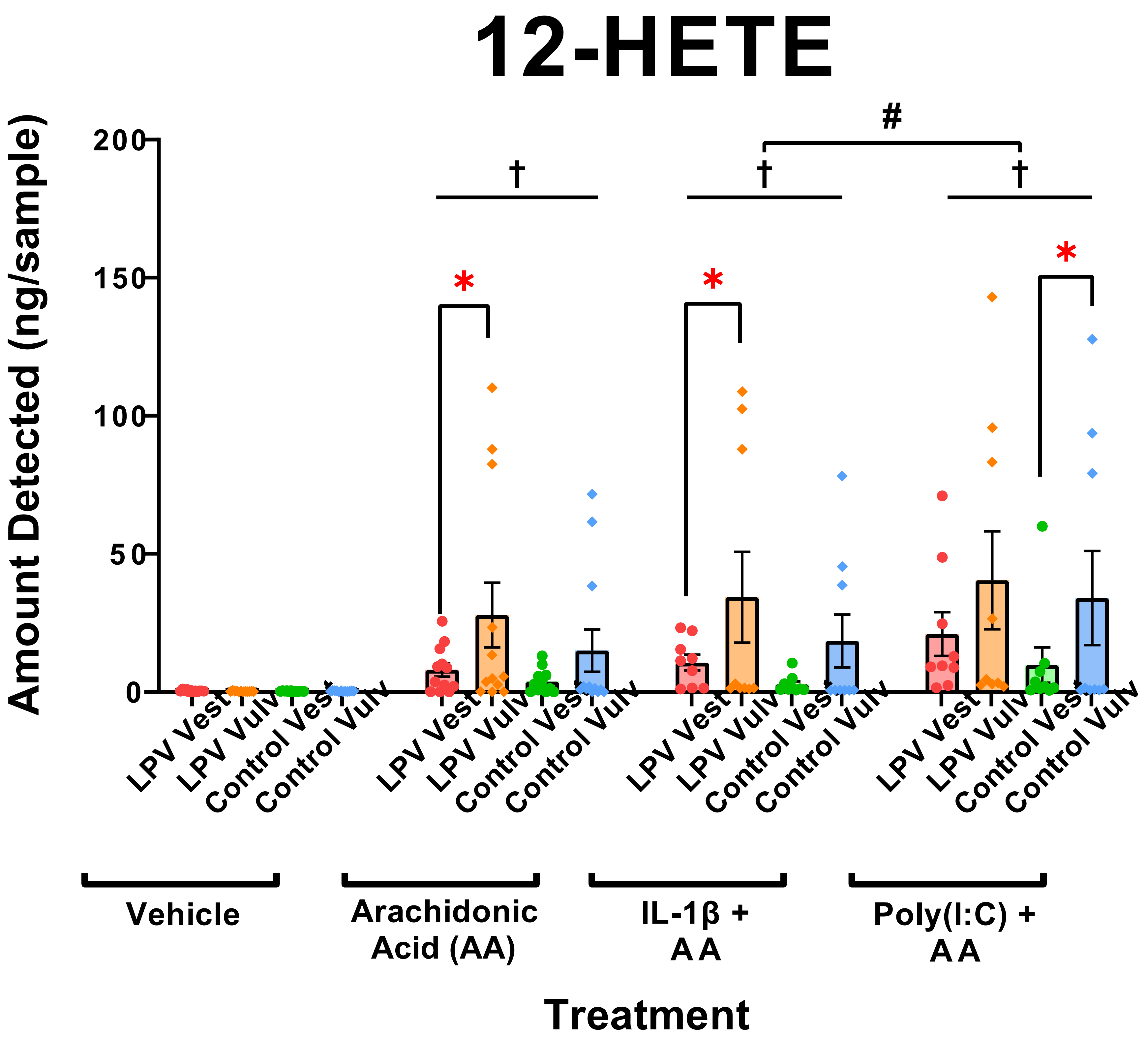

12-HETE
Amount Detected (ng/sample)
200
150
100
50
0
#
†
†
†
*
*
*
LPV Vest
LPV Vulv
Control Vest
Control Vulv
Vehicle
Arachidonic Acid (AA)
IL-1β + AA
Poly(I:C) + AA
Treatment

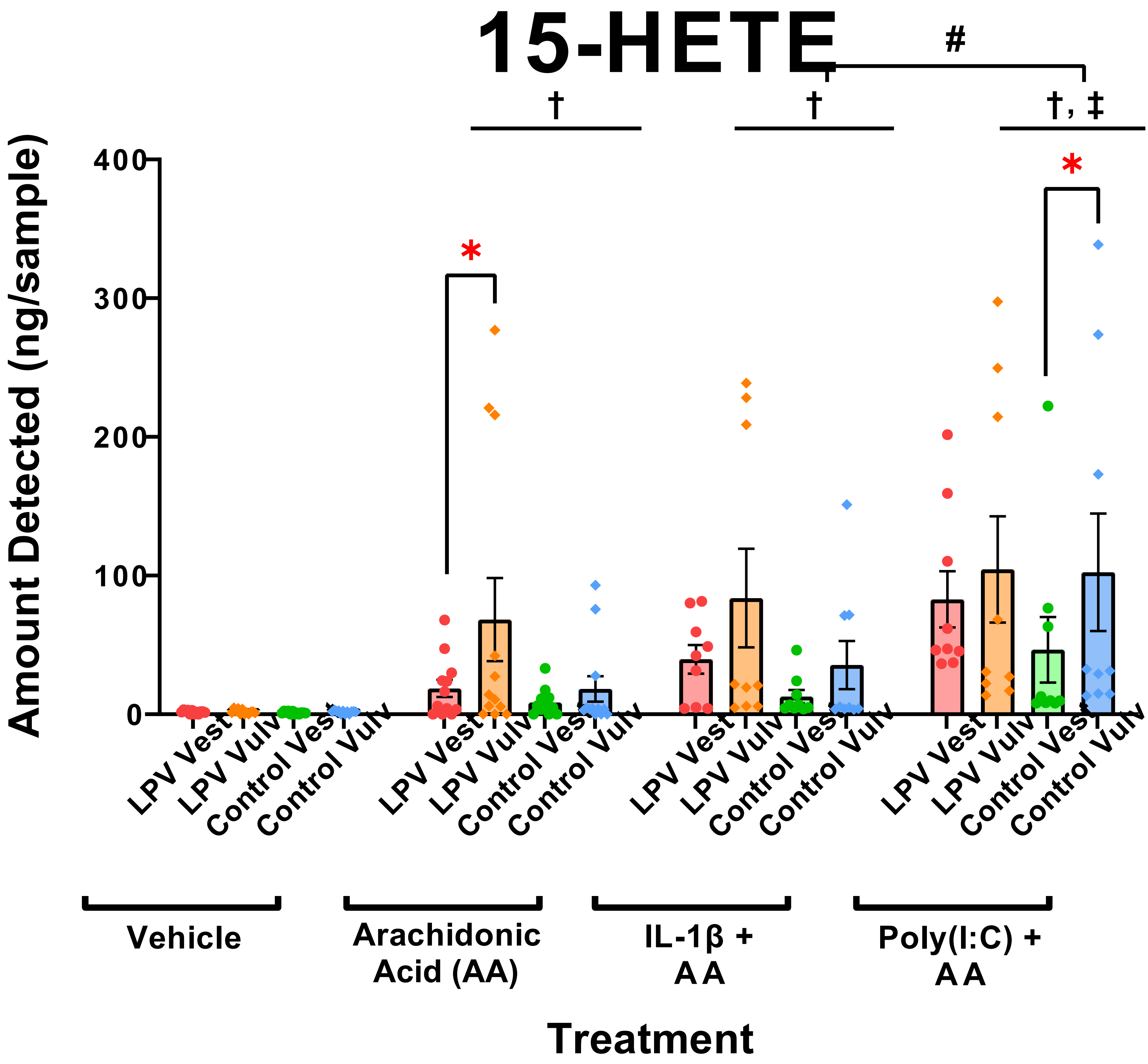

15-HETE
#
†
†
†, ‡
*
*
Amount Detected (ng/sample)
400
300
200
100
0
LPV Vest
LPV Vulv
Control Vest
Control Vulv
Vehicle
Arachidonic Acid (AA)
IL-1β + AA
Poly(I:C) + AA
Treatment

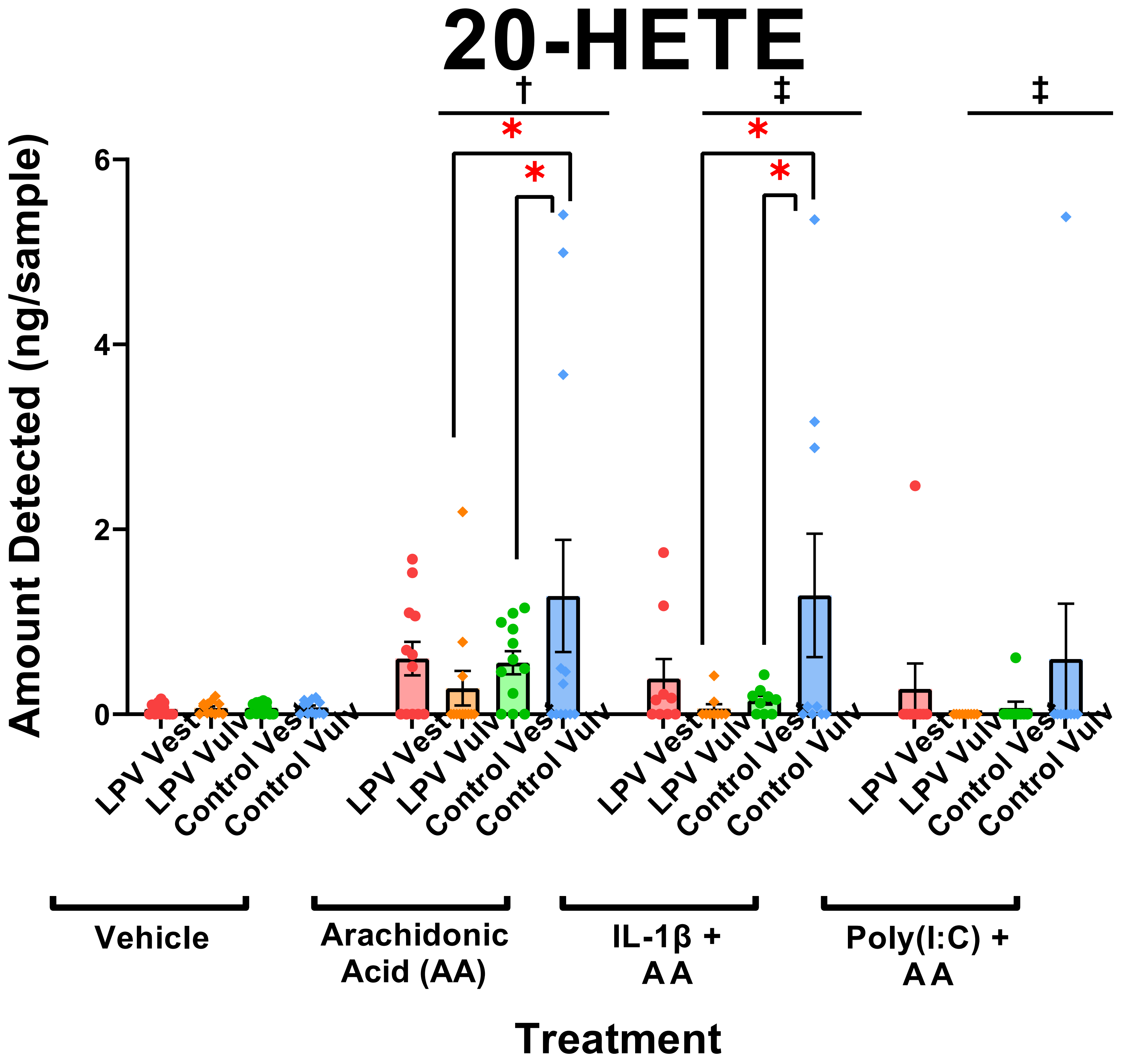

20-HETE
†
‡
‡
*
*
*
*
Amount Detected (ng/sample)
0
2
4
6
LPV Vest
LPV Vulv
Control Vest
Control Vulv
Vehicle
Arachidonic Acid (AA)
IL-1β + AA
Poly(I:C) + AA
Treatment

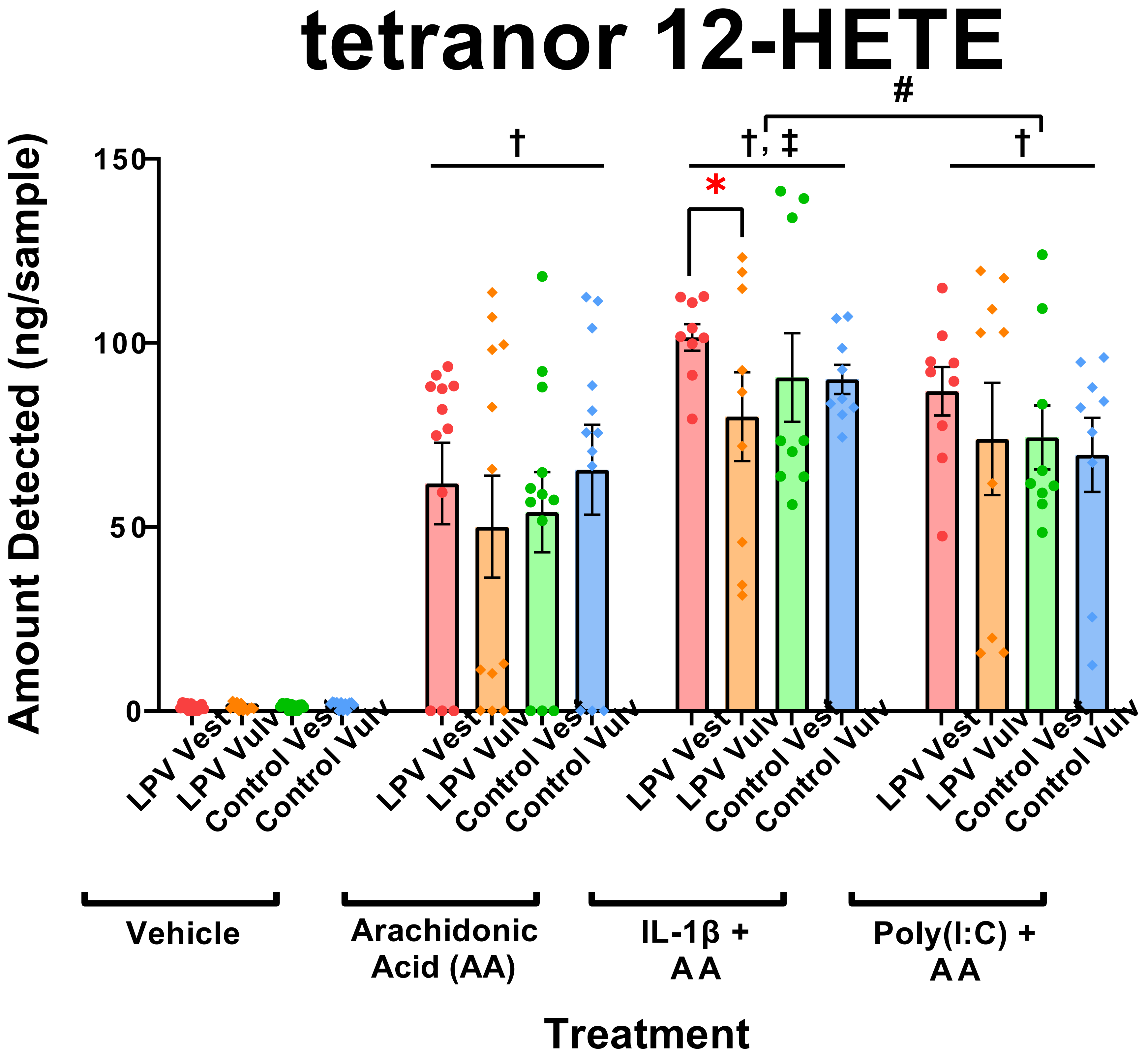
tetranor 12-HETE
Amount Detected (ng/sample)
150
100
50
0
#
†
†, ‡
†
*
LPV Vest
LPV Vulv
Control Vest
Control Vulv
Vehicle
Arachidonic Acid (AA)
IL-1β + AA
Poly(I:C) + AA
Treatment

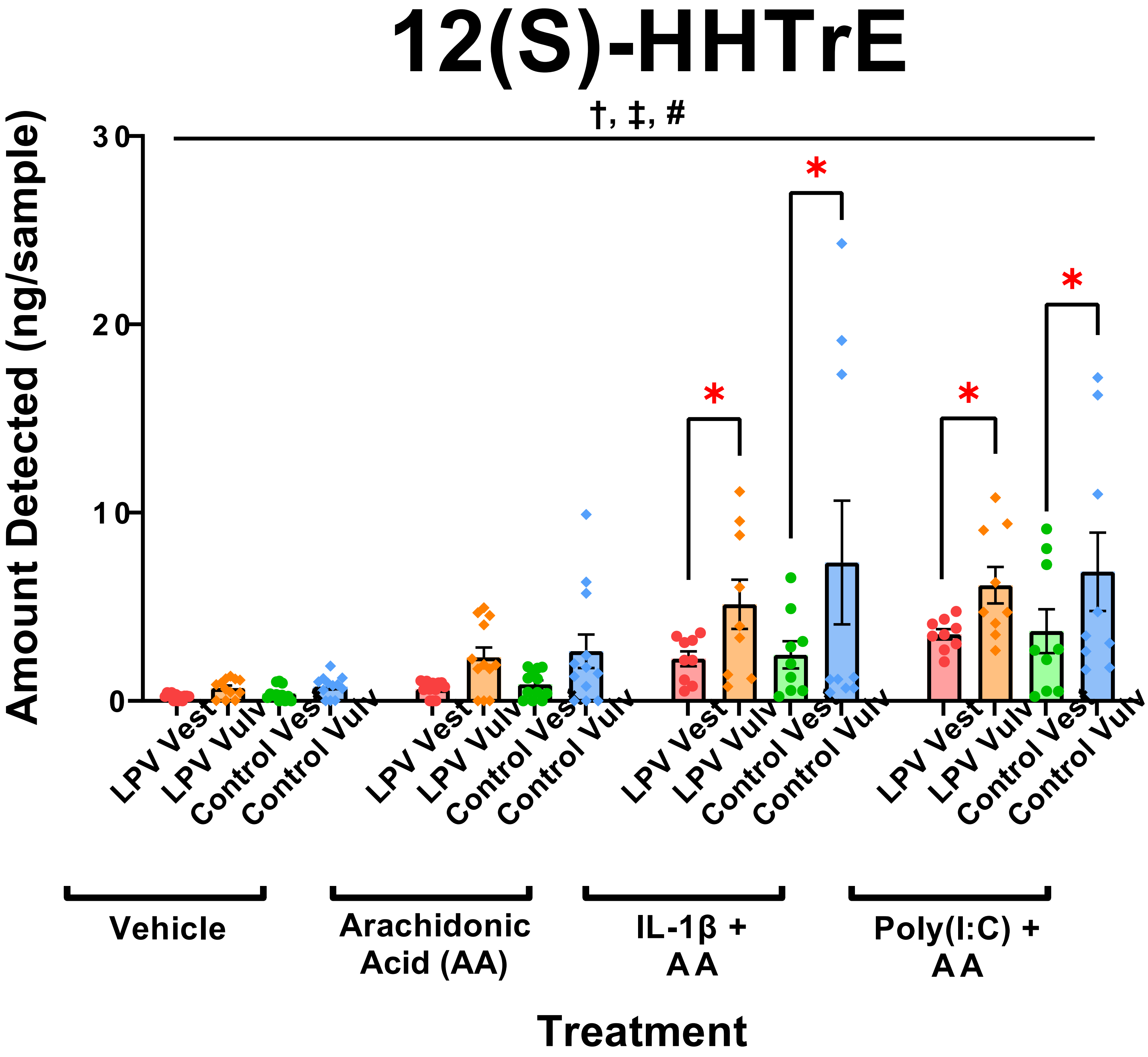

12(S)-HHTrE
†, ‡, #
30
20
10
0
Amount Detected (ng/sample)
*
*
*
*
LPV Vest
LPV Vulv
Control Vest
Control Vulv
Vehicle
Arachidonic Acid (AA)
IL-1β + AA
Poly(I:C) + AA
Treatment

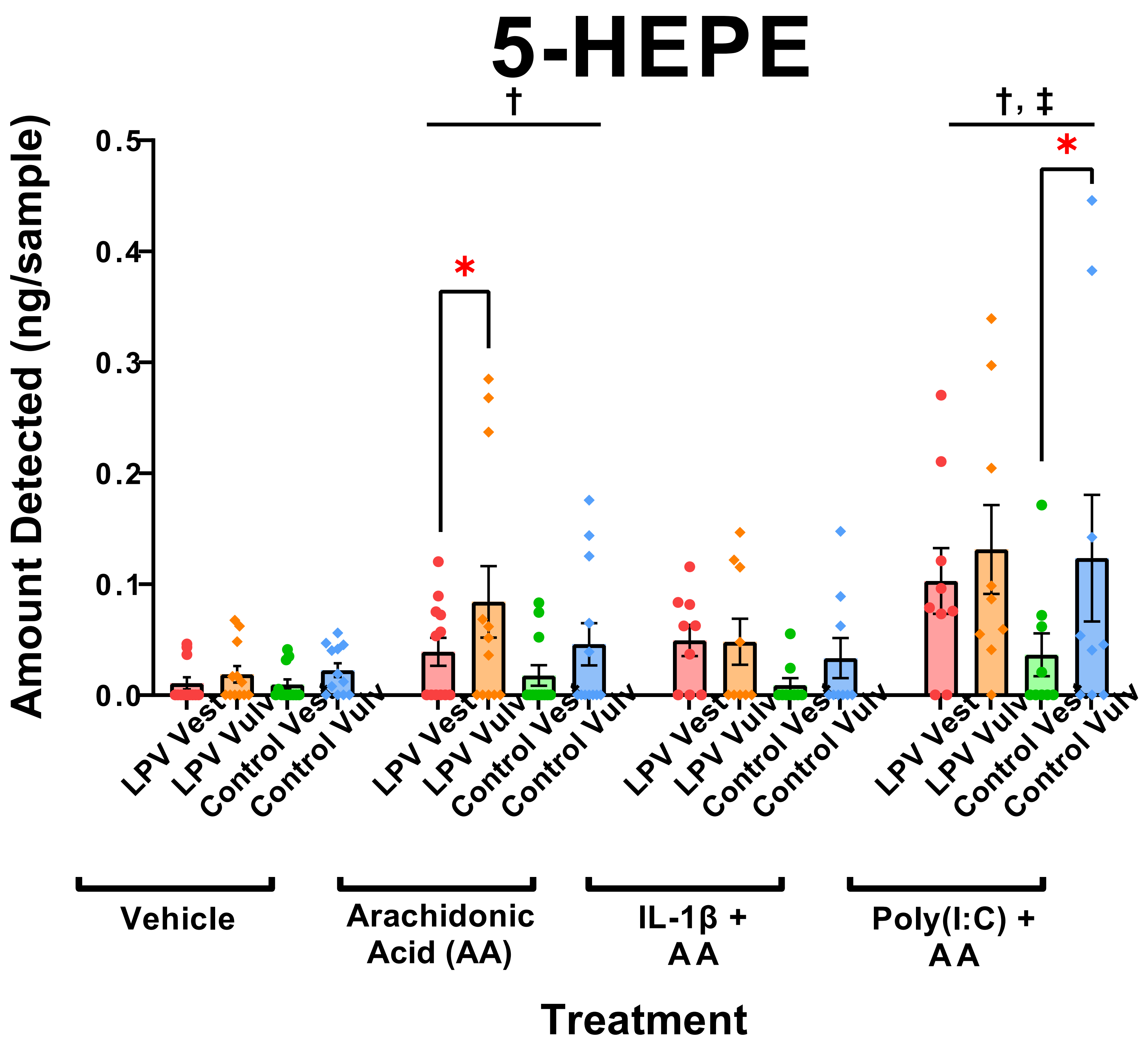

5-HEPE
Amount Detected (ng/sample)
0.0
0.1
0.2
0.3
0.4
0.5
†
†, ‡
*
*
LPV Vest
LPV Vulv
Control Vest
Control Vulv
Vehicle
Arachidonic Acid (AA)
IL-1β + AA
Poly(I:C) + AA
Treatment

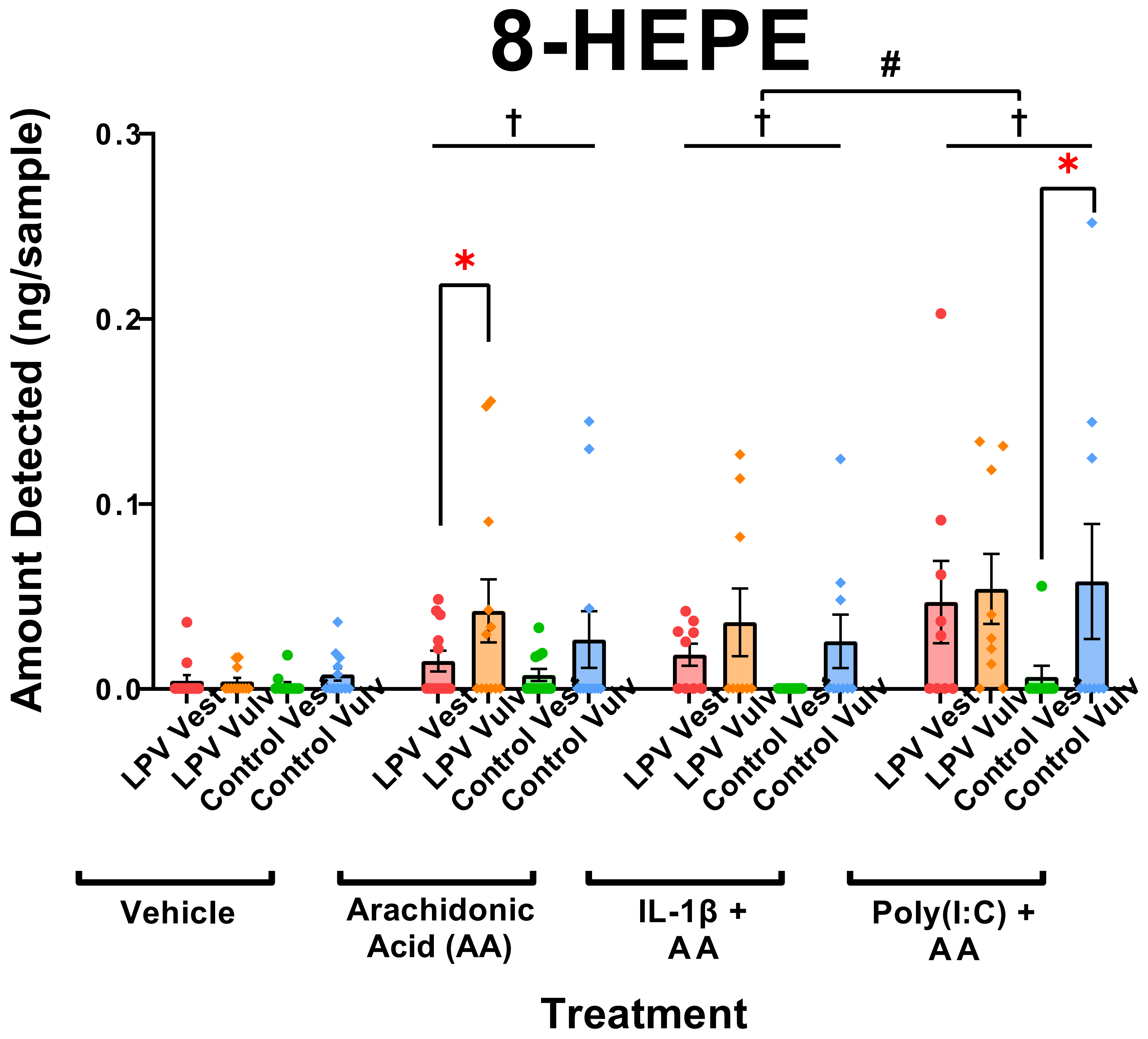

8-HEPE
#
†
†
†
*
*
Amount Detected (ng/sample)
0.3
0.2
0.1
0.0
LPV Vest
LPV Vulv
Control Vest
Control Vulv
Vehicle
Arachidonic Acid (AA)
IL-1β + AA
Poly(I:C) + AA
Treatment

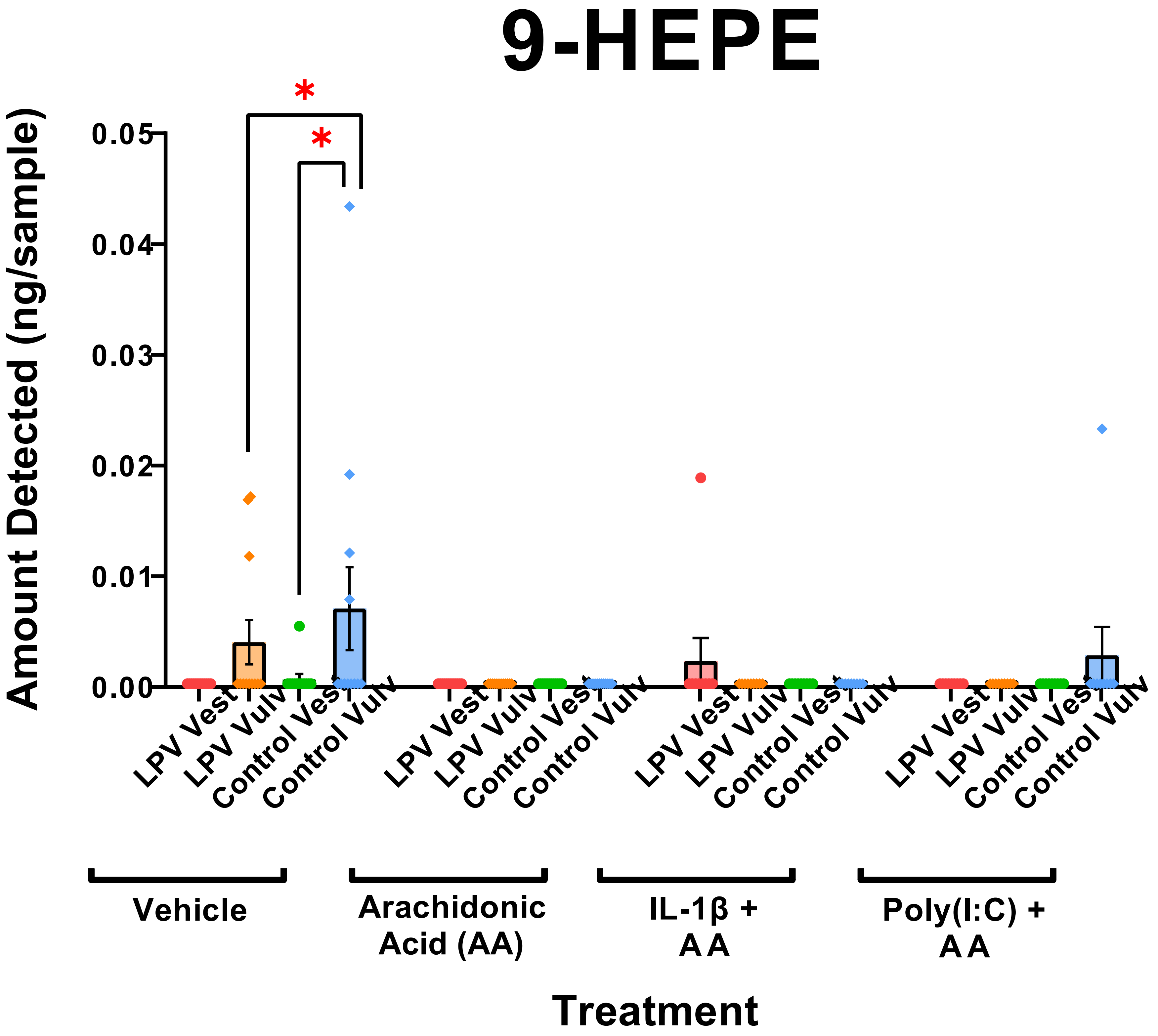
9-HEPE
Amount Detected (ng/sample)
0.05
0.04
0.03
0.02
0.01
0.00
*
*
LPV Vest
LPV Vulv
Control Vest
Control Vulv
Vehicle
Arachidonic Acid (AA)
IL-1β + AA
Poly(I:C) + AA
Treatment

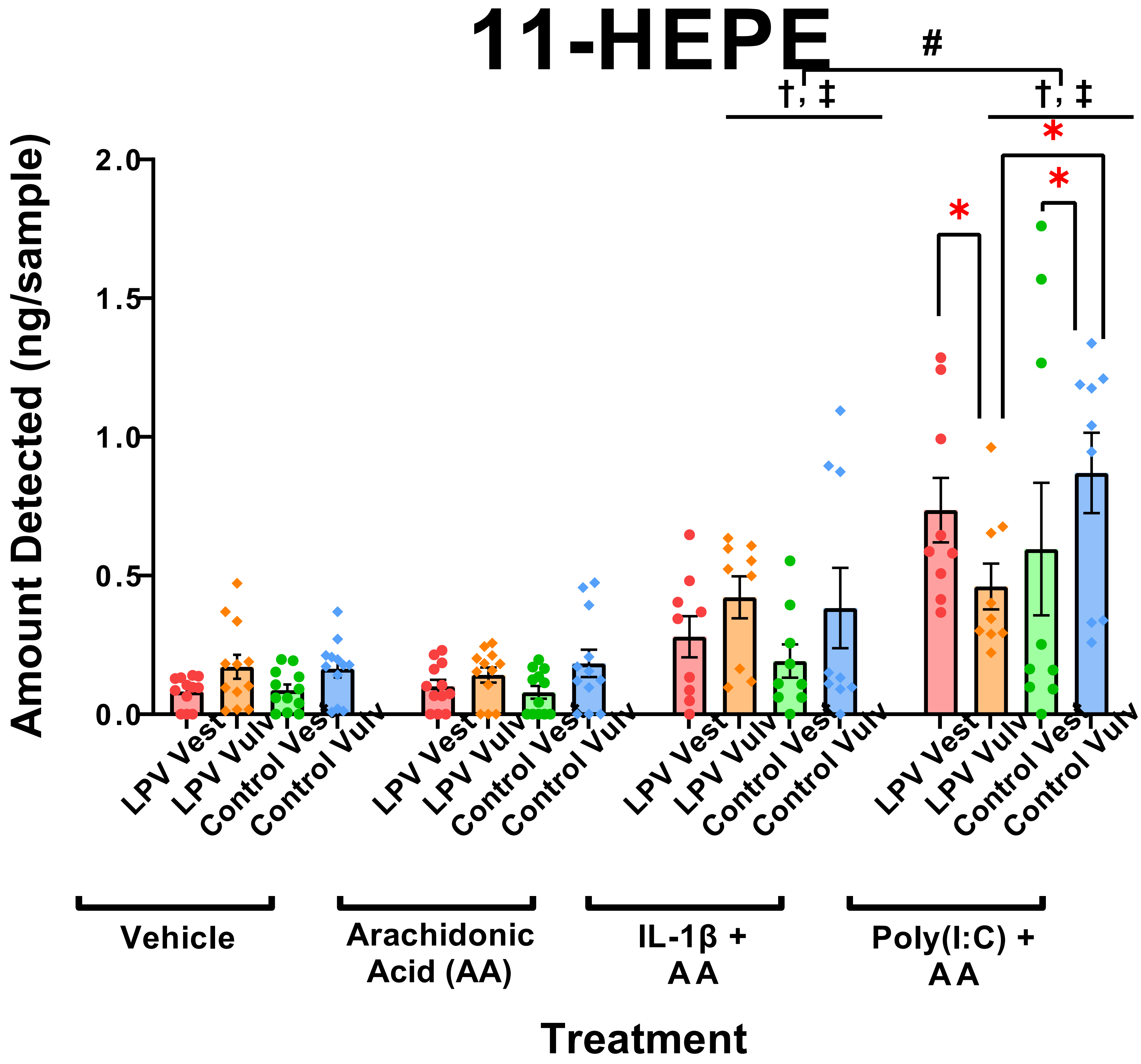

11-HEPE
#
†, ‡
†, ‡
*
*
*
Amount Detected (ng/sample)
2.0
1.5
1.0
0.5
0.0
LPV Vest
LPV Vulv
Control Vest
Control Vulv
Vehicle
Arachidonic Acid (AA)
IL-1β + AA
Poly(I:C) + AA
Treatment

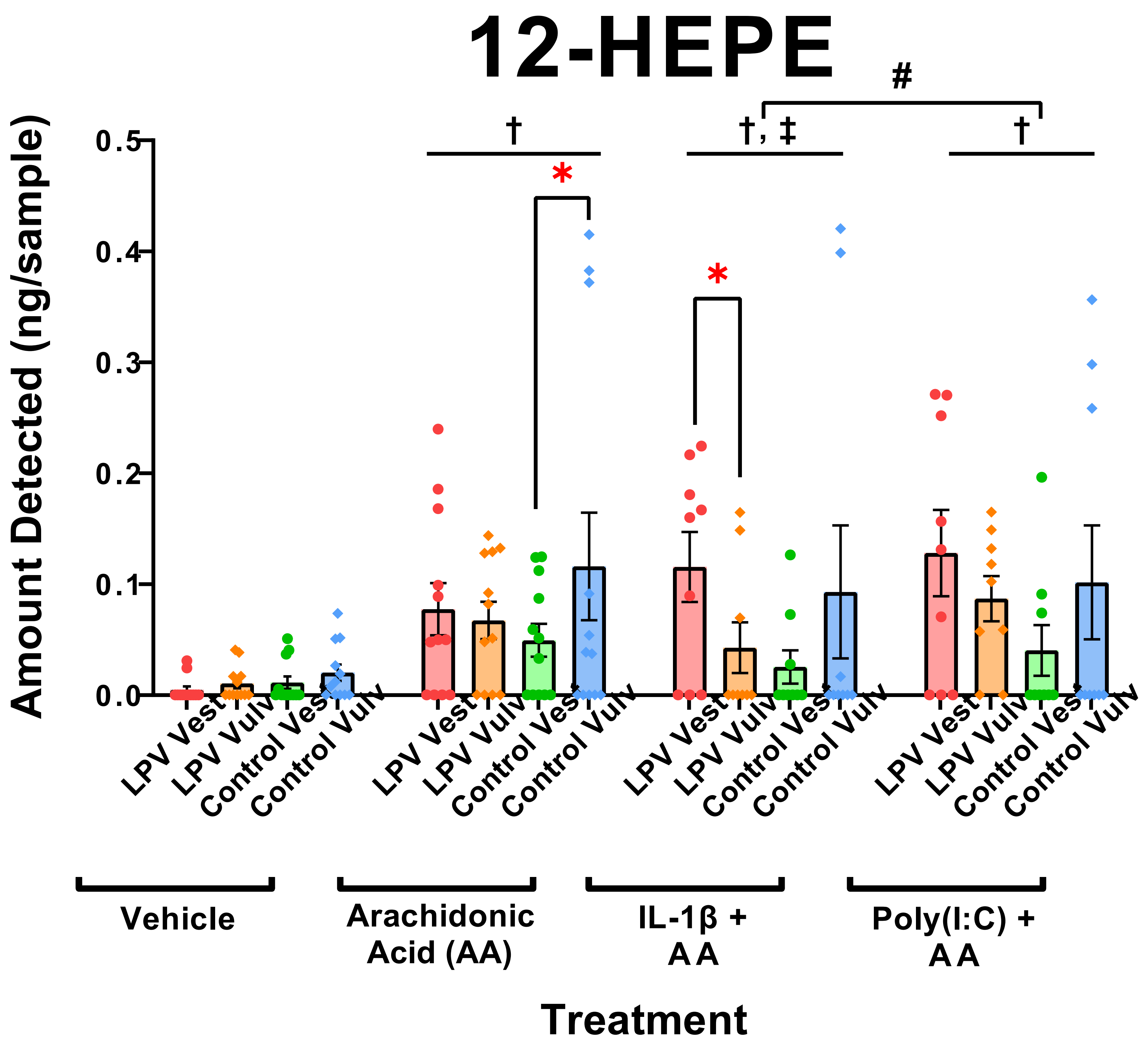

12-HEPE
Amount Detected (ng/sample)
0.0
0.1
0.2
0.3
0.4
0.5
†
†, ‡
†
#
*
*
LPV Vest
LPV Vulv
Control Vest
Control Vulv
Vehicle
Arachidonic Acid (AA)
IL-1β + AA
Poly(I:C) + AA
Treatment

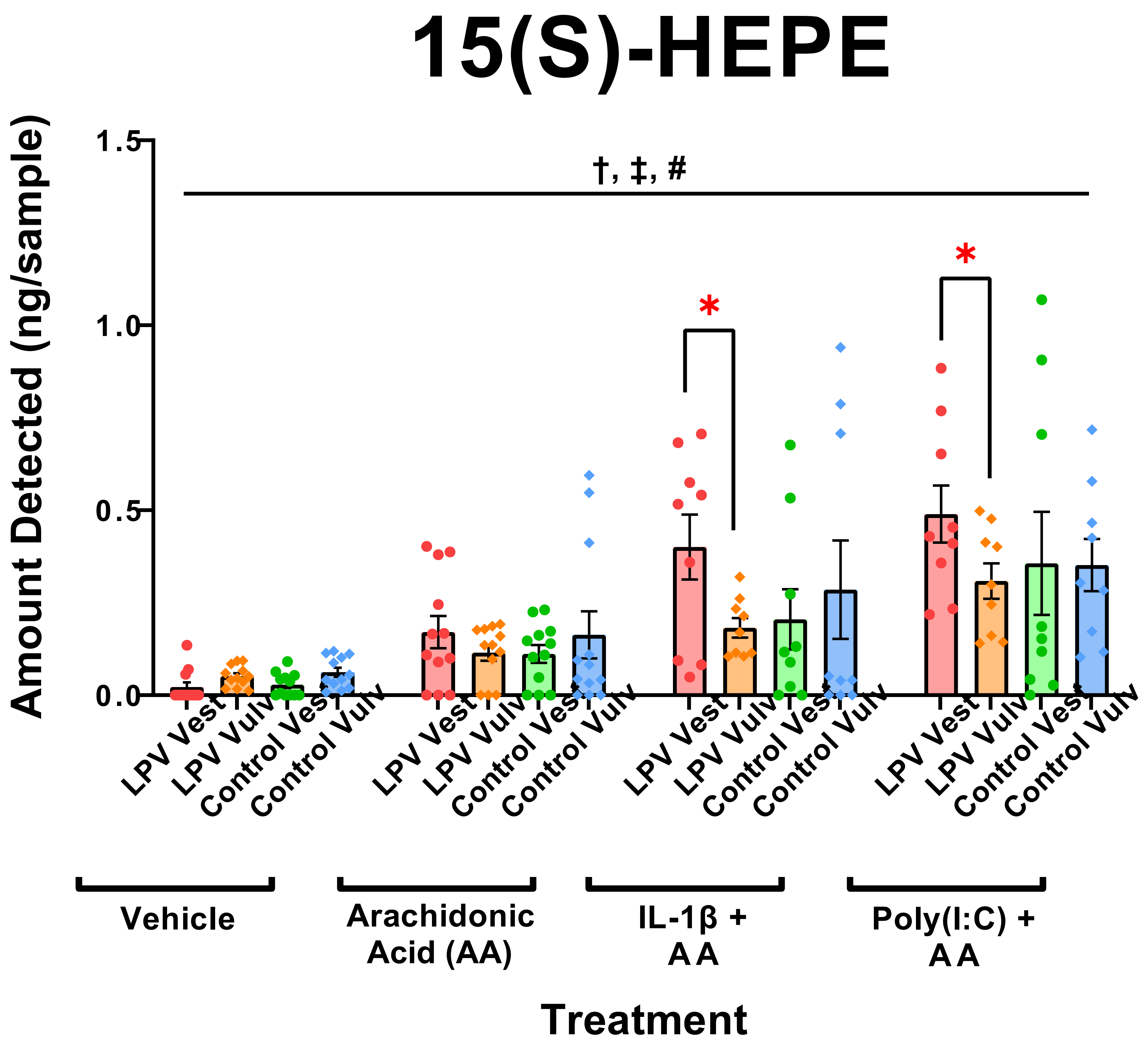

15(S)-HEPE
Amount Detected (ng/sample)
1.5
1.0
0.5
0.0
†, ‡, #
*
*
LPV Vest
LPV Vulv
Control Vest
Control Vulv
Vehicle
Arachidonic Acid (AA)
IL-1β + AA
Poly(I:C) + AA
Treatment

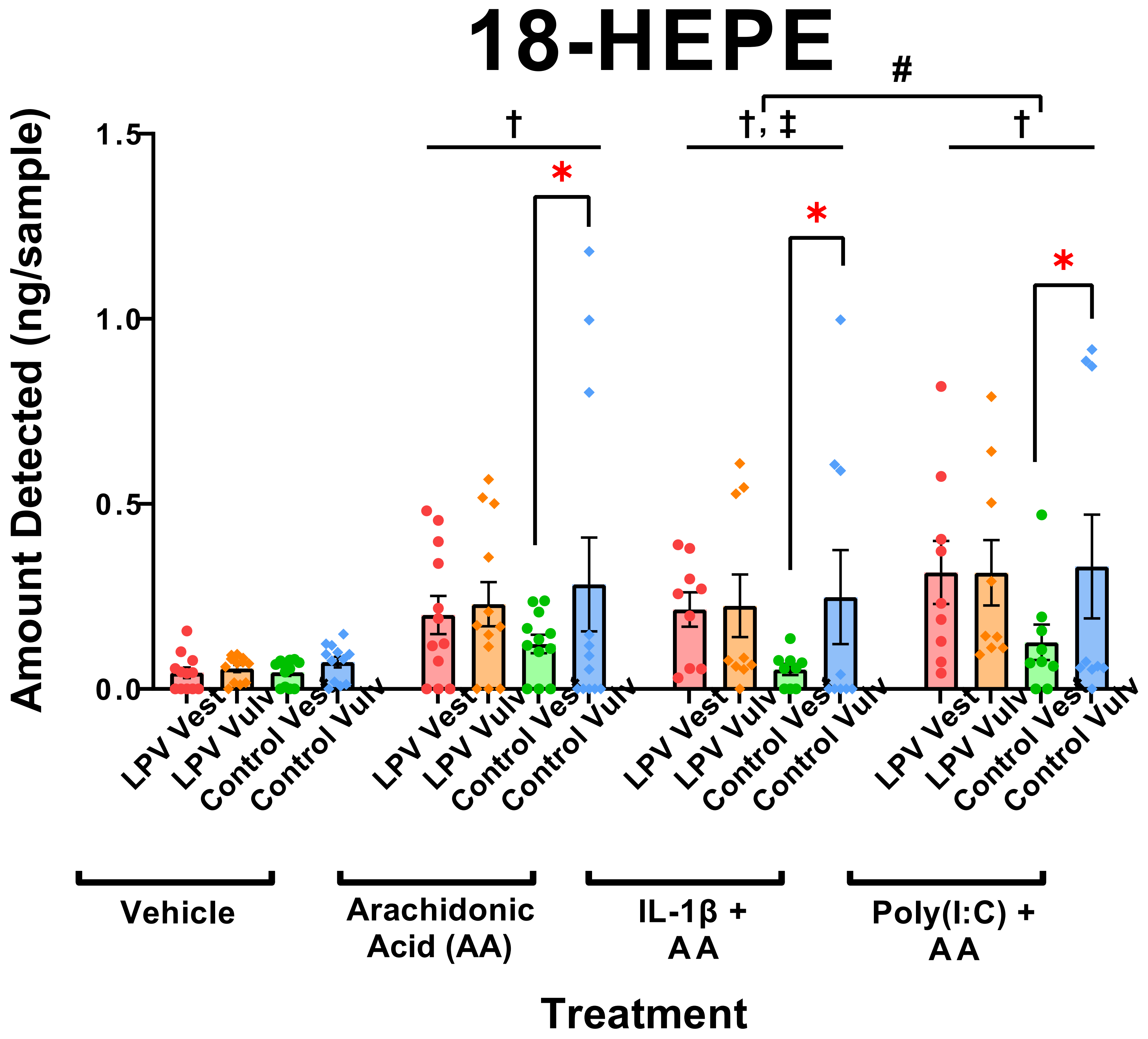

18-HEPE
Amount Detected (ng/sample)
1.5
1.0
0.5
0.0
#
†
†, ‡
†
*
*
*
LPV Vest
LPV Vulv
Control Vest
Control Vulv
Vehicle
Arachidonic Acid (AA)
IL-1β + AA
Poly(I:C) + AA
Treatment

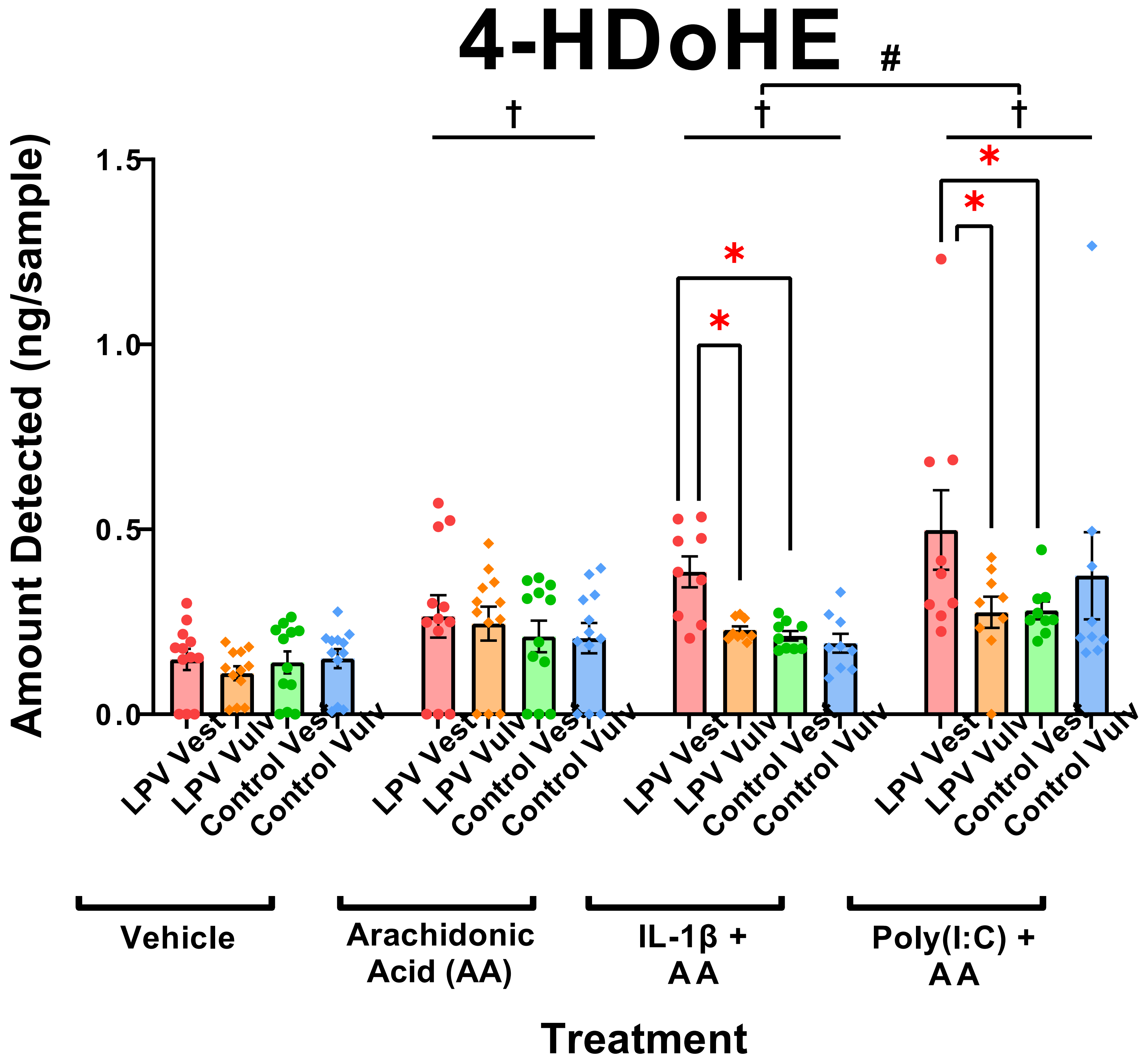

4-HDoHE
#
†
†
†
*
*
*
*
Amount Detected (ng/sample)
1.5
1.0
0.5
0.0
LPV Vest
LPV Vulv
Control Vest
Control Vulv
LPV Vest
LPV Vulv
Control Vest
Control Vulv
LPV Vest
LPV Vulv
Control Vest
Control Vulv
LPV Vest
LPV Vulv
Control Vest
Control Vulv
Vehicle
Arachidonic Acid (AA)
IL-1β + AA
Poly(I:C) + AA
Treatment

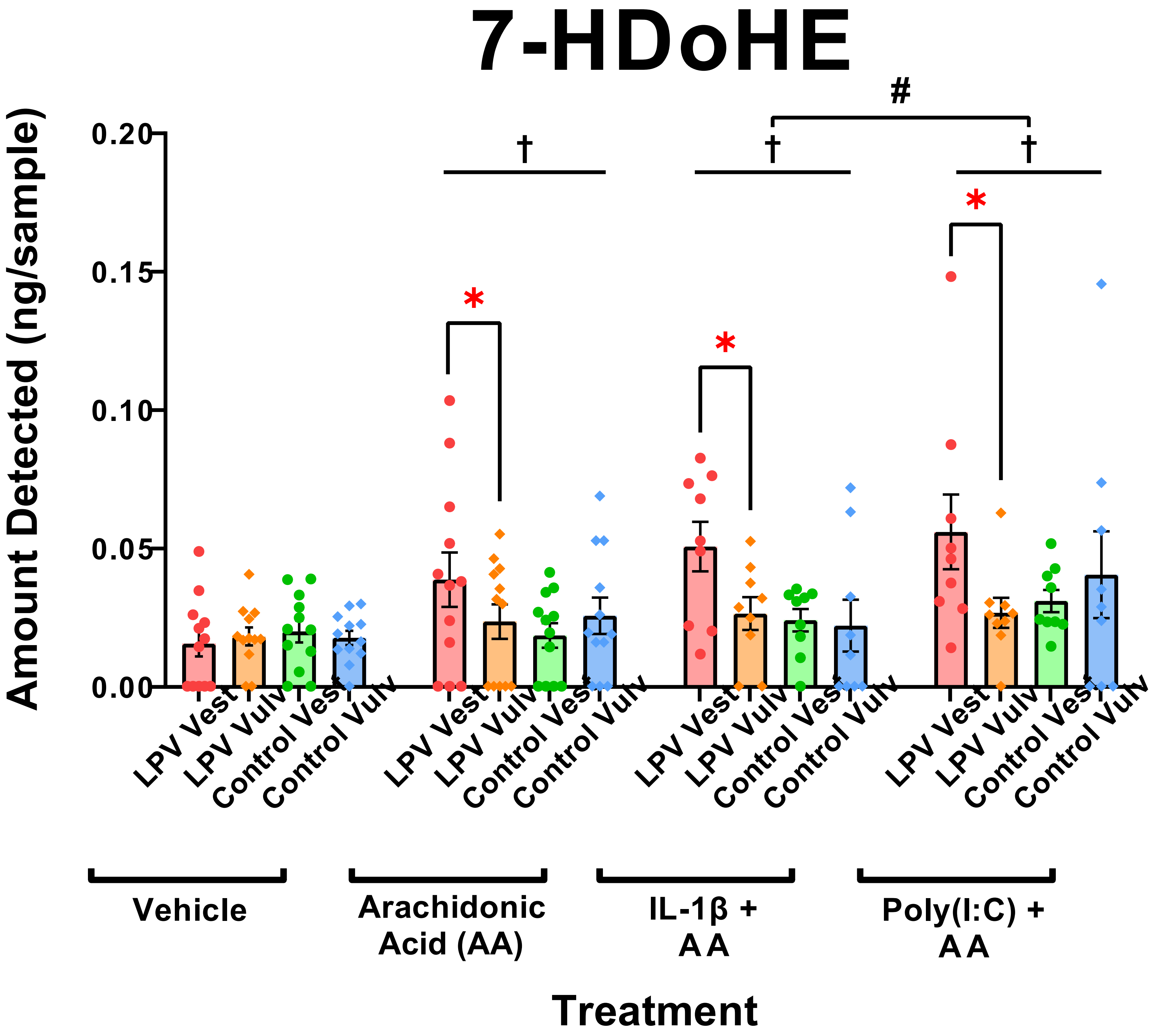

7-HDoHE
Amount Detected (ng/sample)
0.00
0.05
0.10
0.15
0.20
#
†
†
†
*
*
*
LPV Vest
LPV Vulv
Control Vest
Control Vulv
Vehicle
Arachidonic Acid (AA)
IL-1β + AA
Poly(I:C) + AA
Treatment

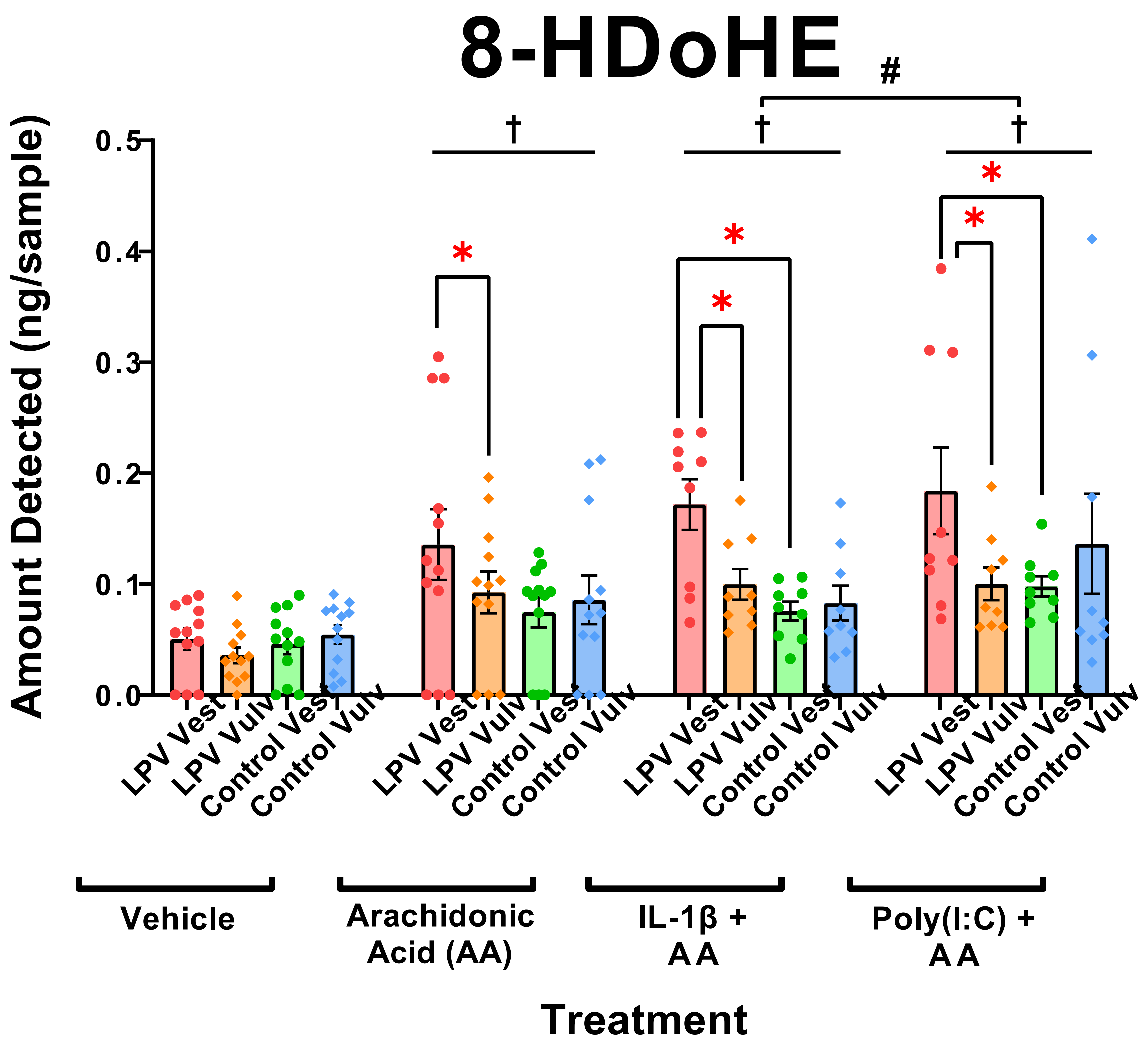

8-HDoHE
#
†
*
Amount Detected (ng/sample)
0.0
0.1
0.2
0.3
0.4
0.5
LPV Vest
LPV Vulv
Control Vest
Control Vulv
Vehicle
Arachidonic Acid (AA)
IL-1β + AA
Poly(I:C) + AA
Treatment

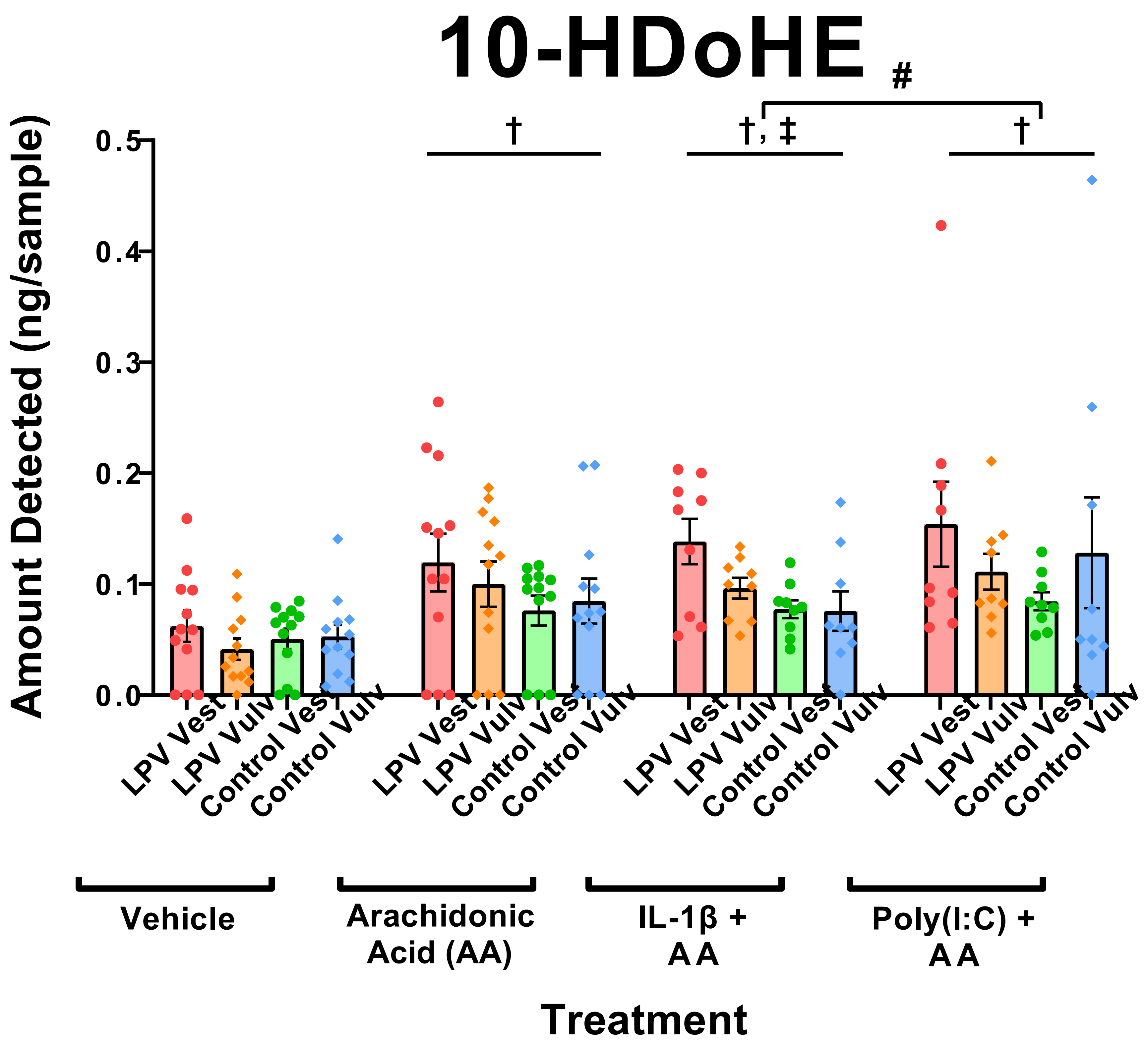

10-HDoHE #
Amount Detected (ng/sample)
0.0
0.1
0.2
0.3
0.4
0.5
†
†, ‡
†
LPV Vest
LPV Vulv
Control Vest
Control Vulv
Vehicle
Arachidonic Acid (AA)
IL-1β + AA
Poly(I:C) + AA
Treatment

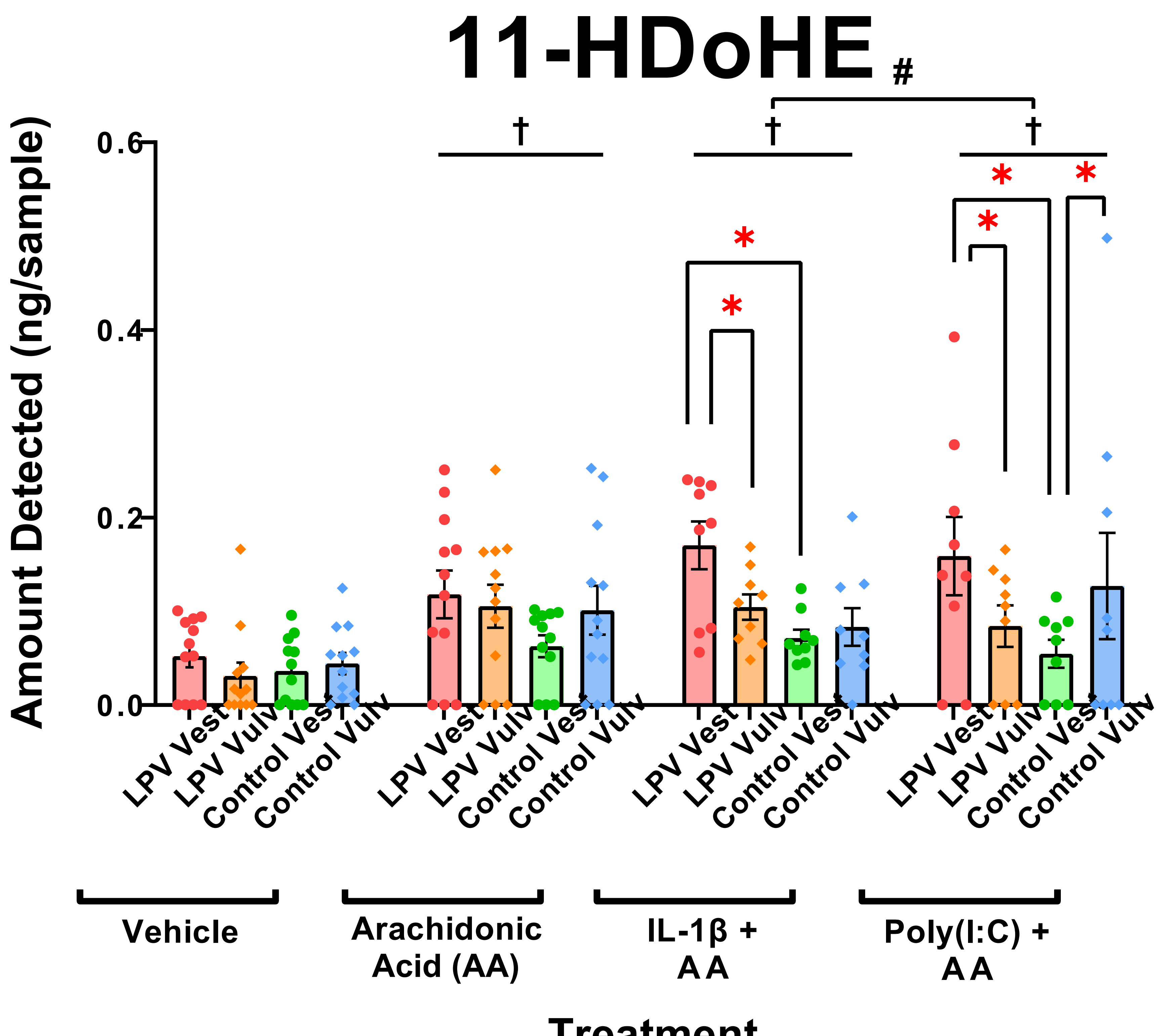

11-HDoHE #
Amount Detected (ng/sample)
0.0
0.2
0.4
0.6
†
†
†
*
*
*
*
*
LPV Vest
LPV Vulv
Control Vest
Control Vulv
Vehicle
Arachidonic Acid (AA)
IL-1β + AA
Poly(I:C) + AA
Treatment

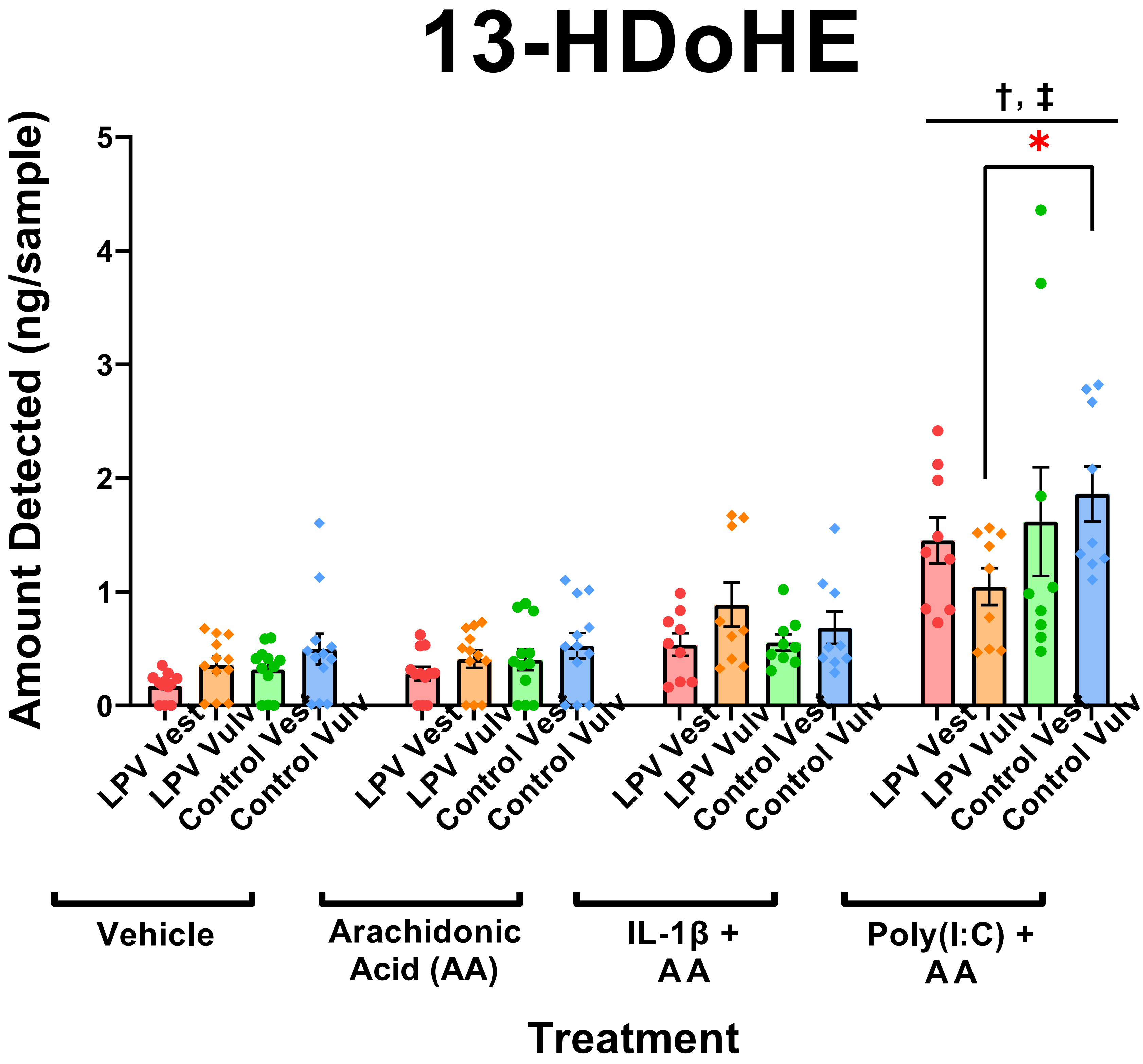
13-HDoHE
†, ‡
*
Amount Detected (ng/sample)
5
4
3
2
1
0
LPV Vest
LPV Vulv
Control Vest
Control Vulv
Vehicle
Arachidonic Acid (AA)
IL-1β + AA
Poly(I:C) + AA
Treatment

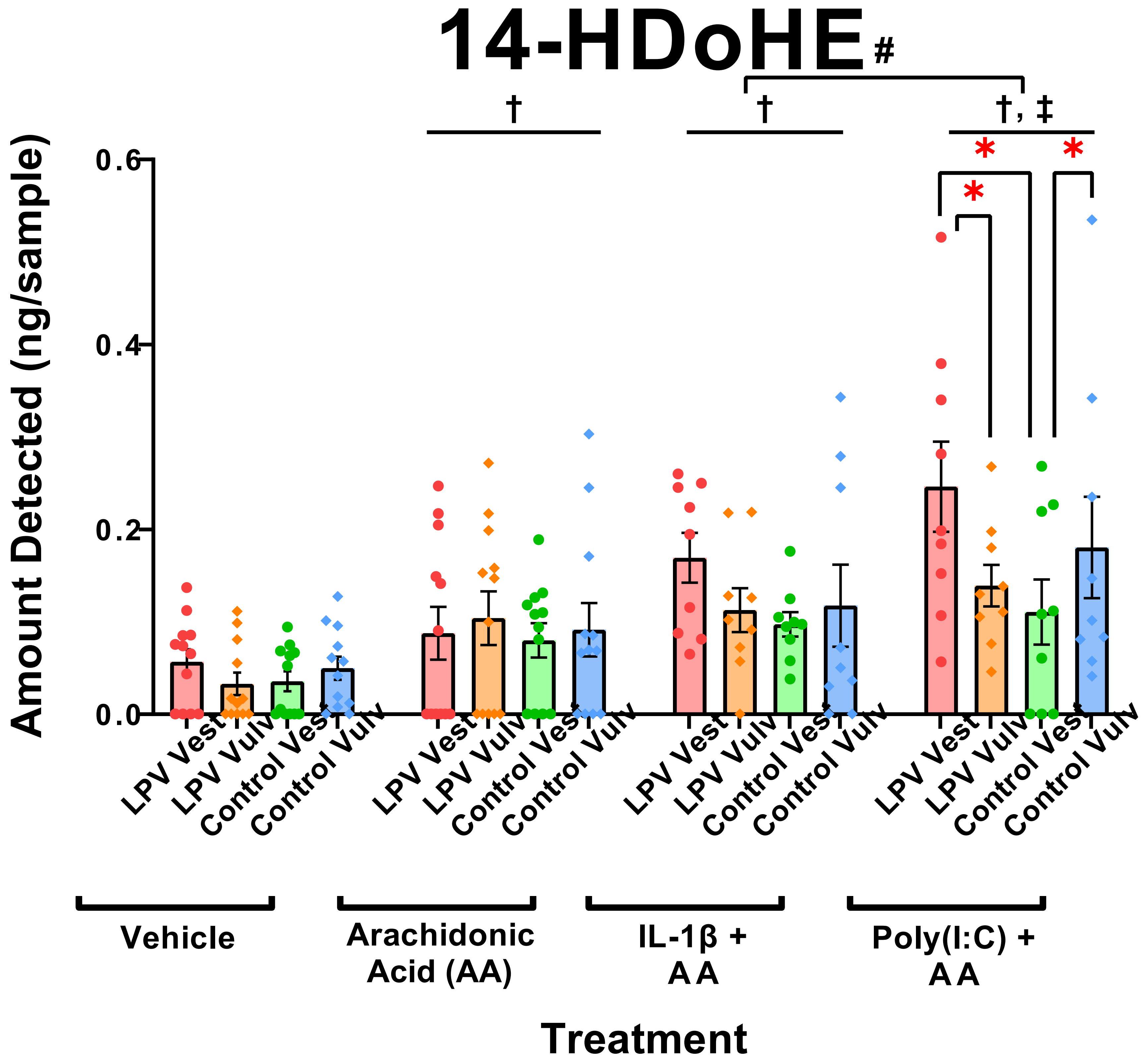

14-HDoHE #
†
†
†, ‡
*
*
*
Amount Detected (ng/sample)
0.6
0.4
0.2
0.0
LPV Vest
LPV Vulv
Control Vest
Control Vulv
Vehicle
Arachidonic Acid (AA)
IL-1β + AA
Poly(I:C) + AA
Treatment

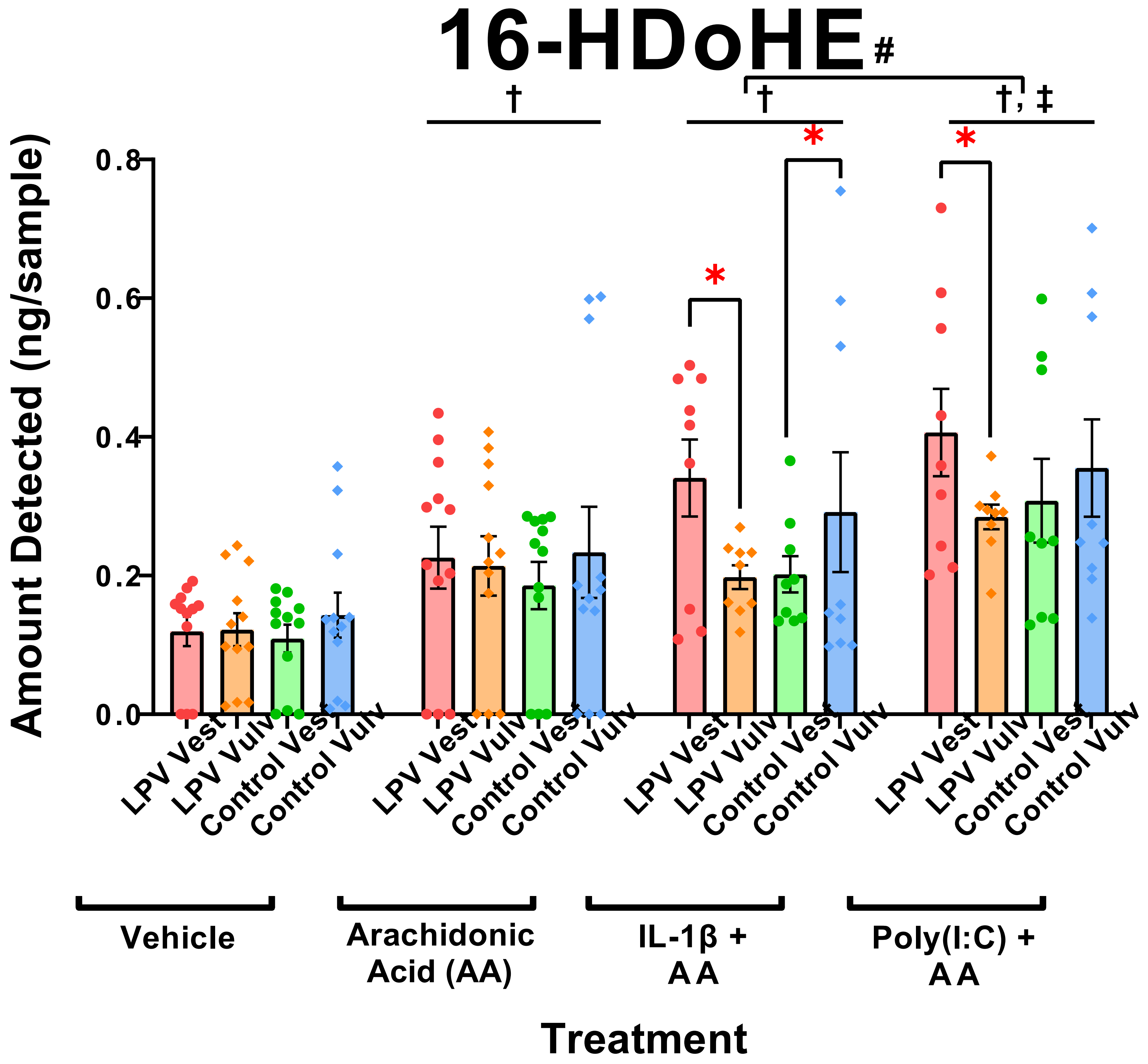

16-HDoHE #
†
†
†, ‡
*
*
*
Amount Detected (ng/sample)
0.8
0.6
0.4
0.2
0.0
LPV Vest
LPV Vulv
Control Vest
Control Vulv
Vehicle
Arachidonic Acid (AA)
IL-1β + AA
Poly(I:C) + AA
Treatment

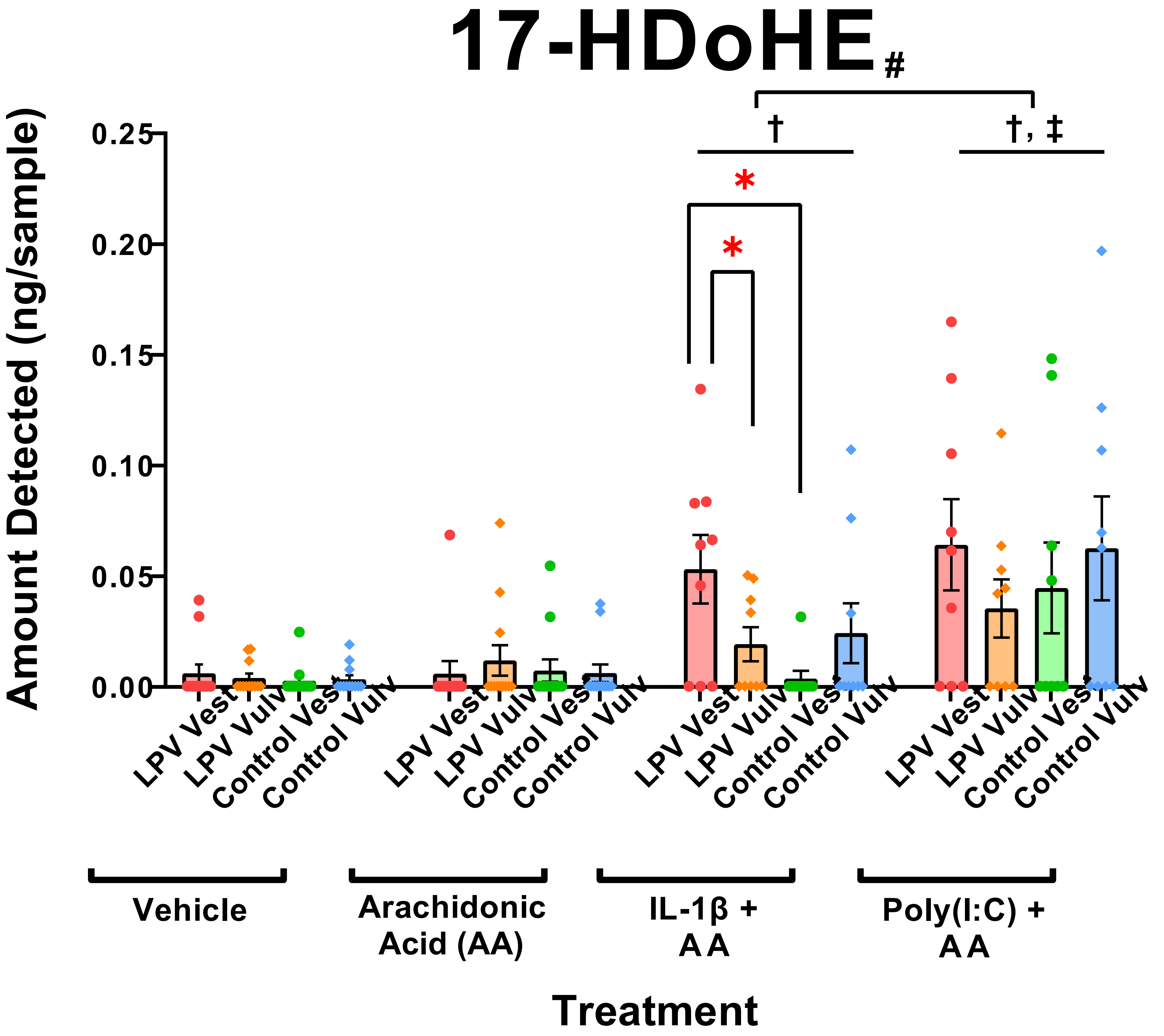

17-HDoHE#
Amount Detected (ng/sample)
0.25
0.20
0.15
0.10
0.05
0.00
†
†, ‡
*
*
LPV Vest
LPV Vulv
Control Vest
Control Vulv
Vehicle
Arachidonic Acid (AA)
IL-1β + AA
Poly(I:C) + AA
Treatment

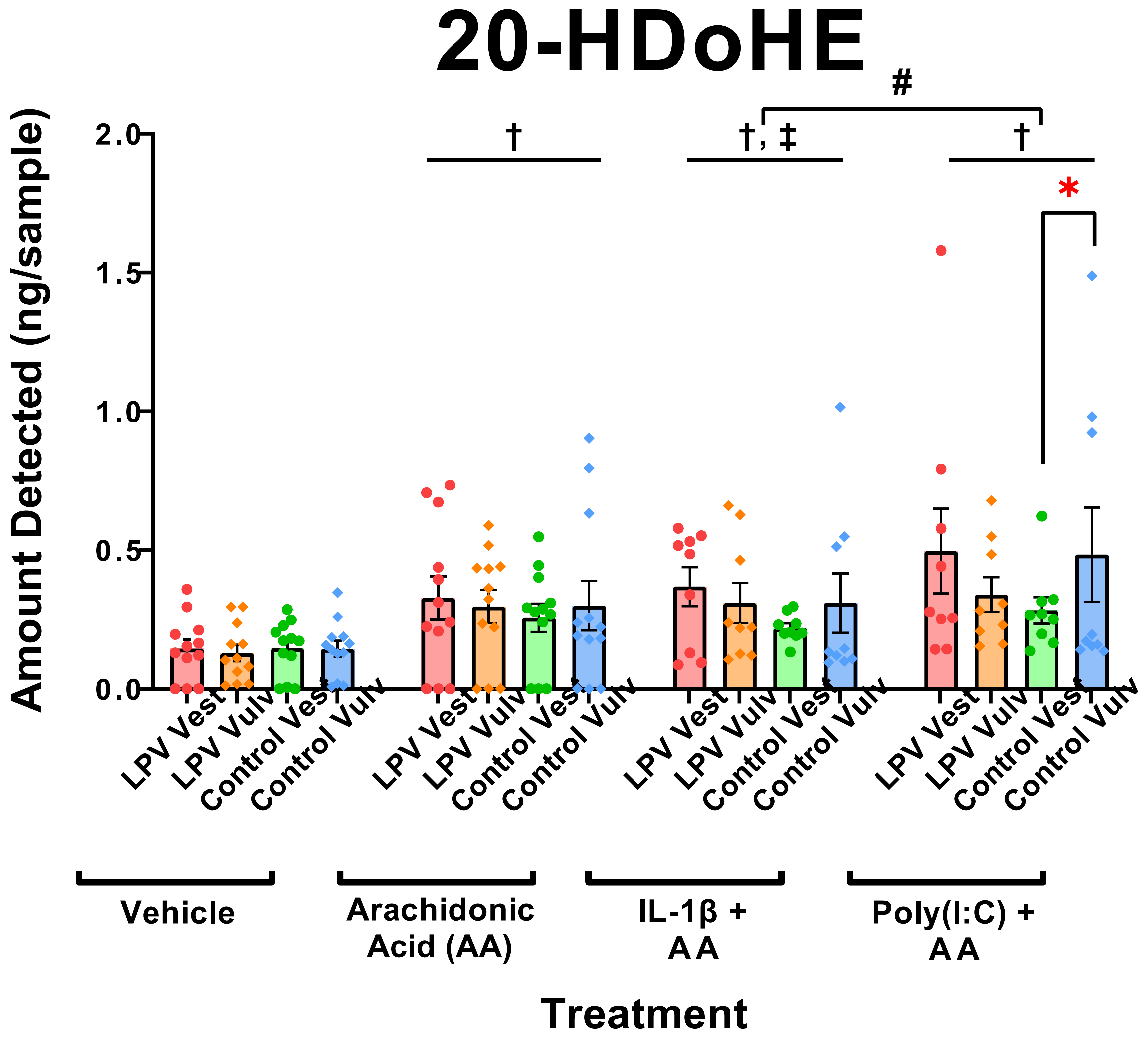
20-HDoHE
Amount Detected (ng/sample)
2.0
1.5
1.0
0.5
0.0
#
†
†, ‡
†
*
LPV Vest
LPV Vulv
Control Vest
Control Vulv
Vehicle
Arachidonic Acid (AA)
IL-1β + AA
Poly(I:C) + AA
Treatment

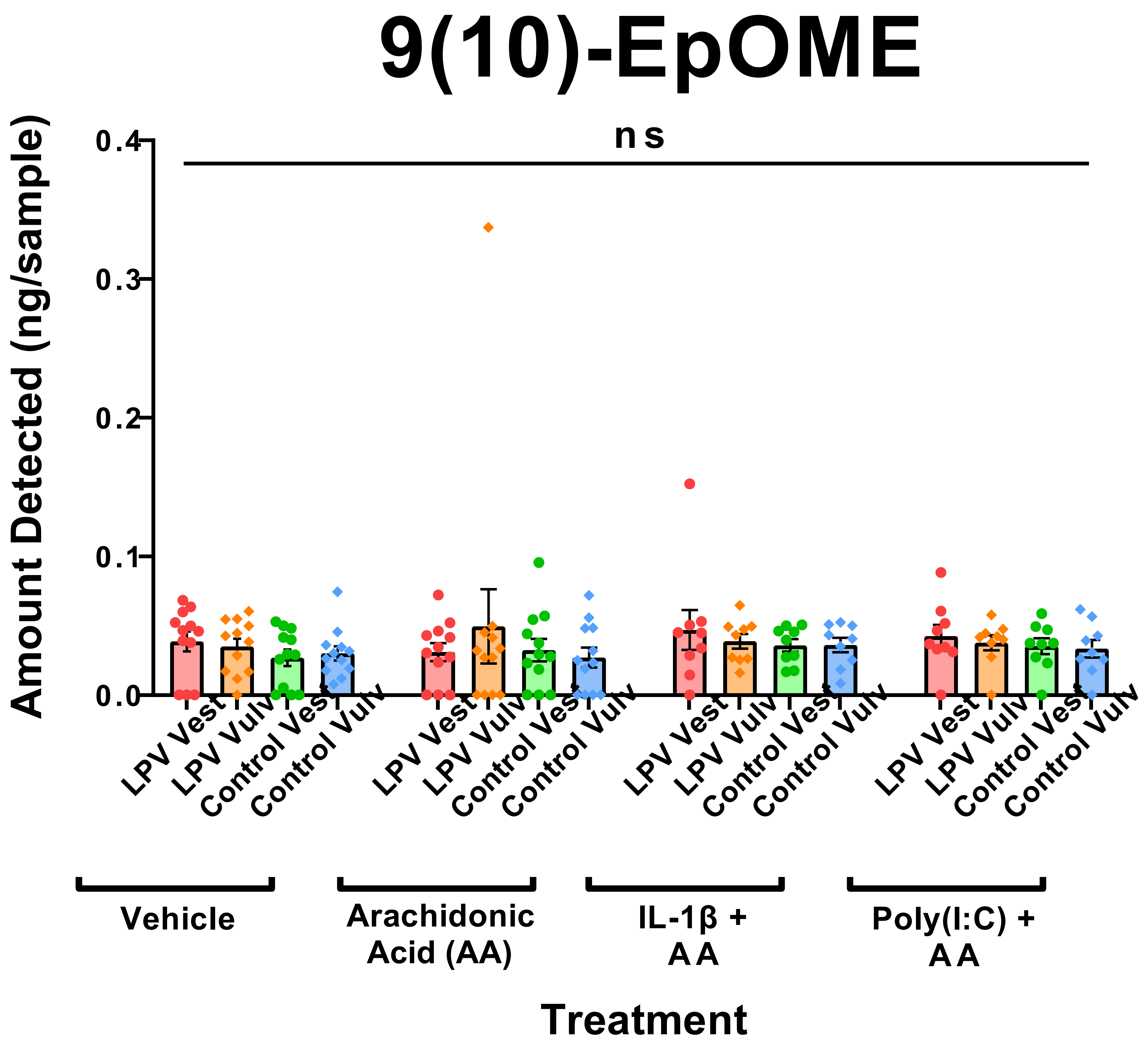

9(10)-EpOME
ns
Amount Detected (ng/sample)
0.0
0.1
0.2
0.3
0.4
LPV Vest
LPV Vulv
Control Vest
Control Vulv
Vehicle
Arachidonic Acid (AA)
IL-1β + AA
Poly(I:C) + AA
Treatment

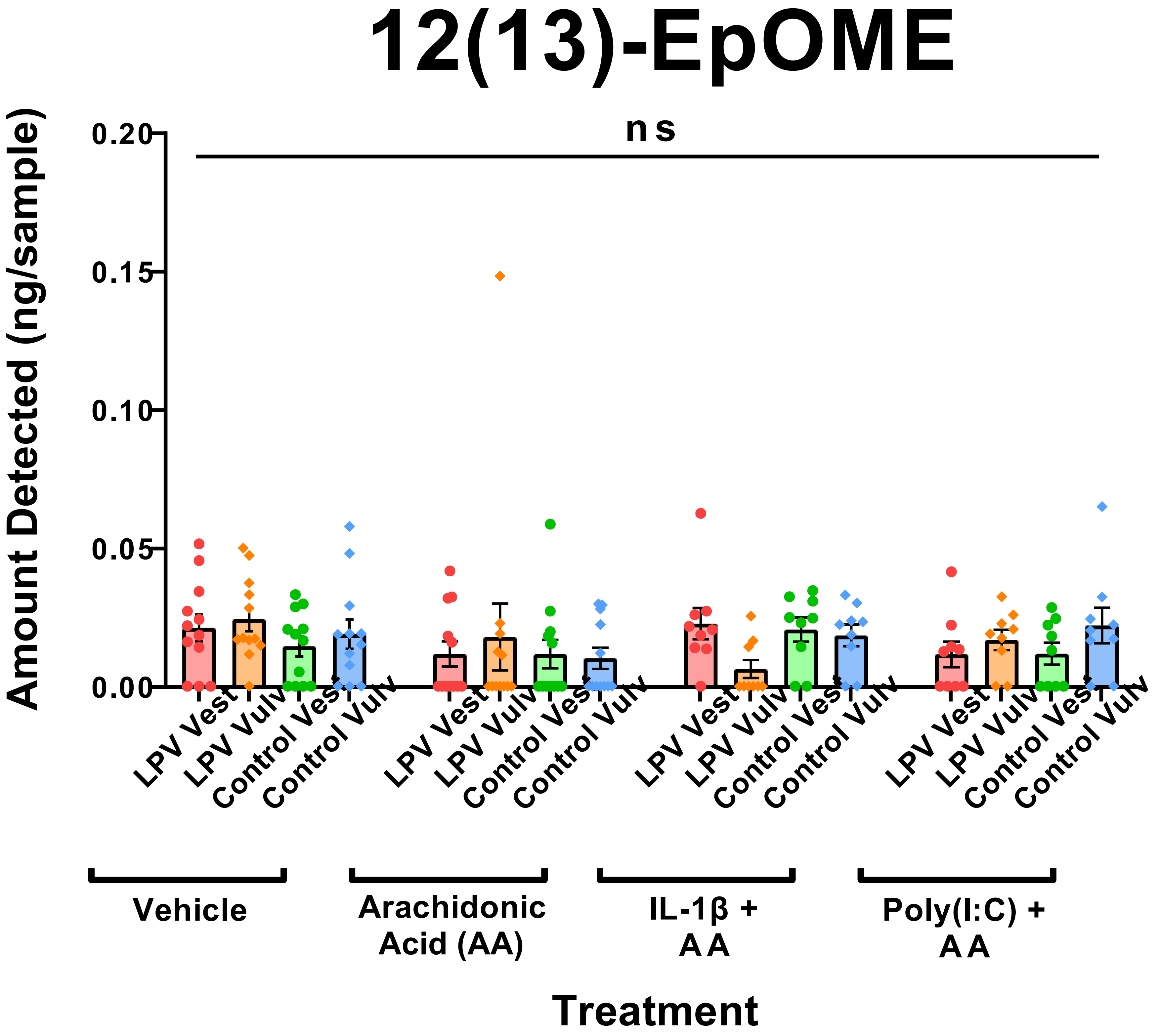

12(13)-EpOME
ns
Amount Detected (ng/sample)
0.20
0.15
0.10
0.05
0.00
LPV Vest
LPV Vulv
Control Vest
Control Vulv
Vehicle
Arachidonic Acid (AA)
IL-1β + AA
Poly(I:C) + AA
Treatment

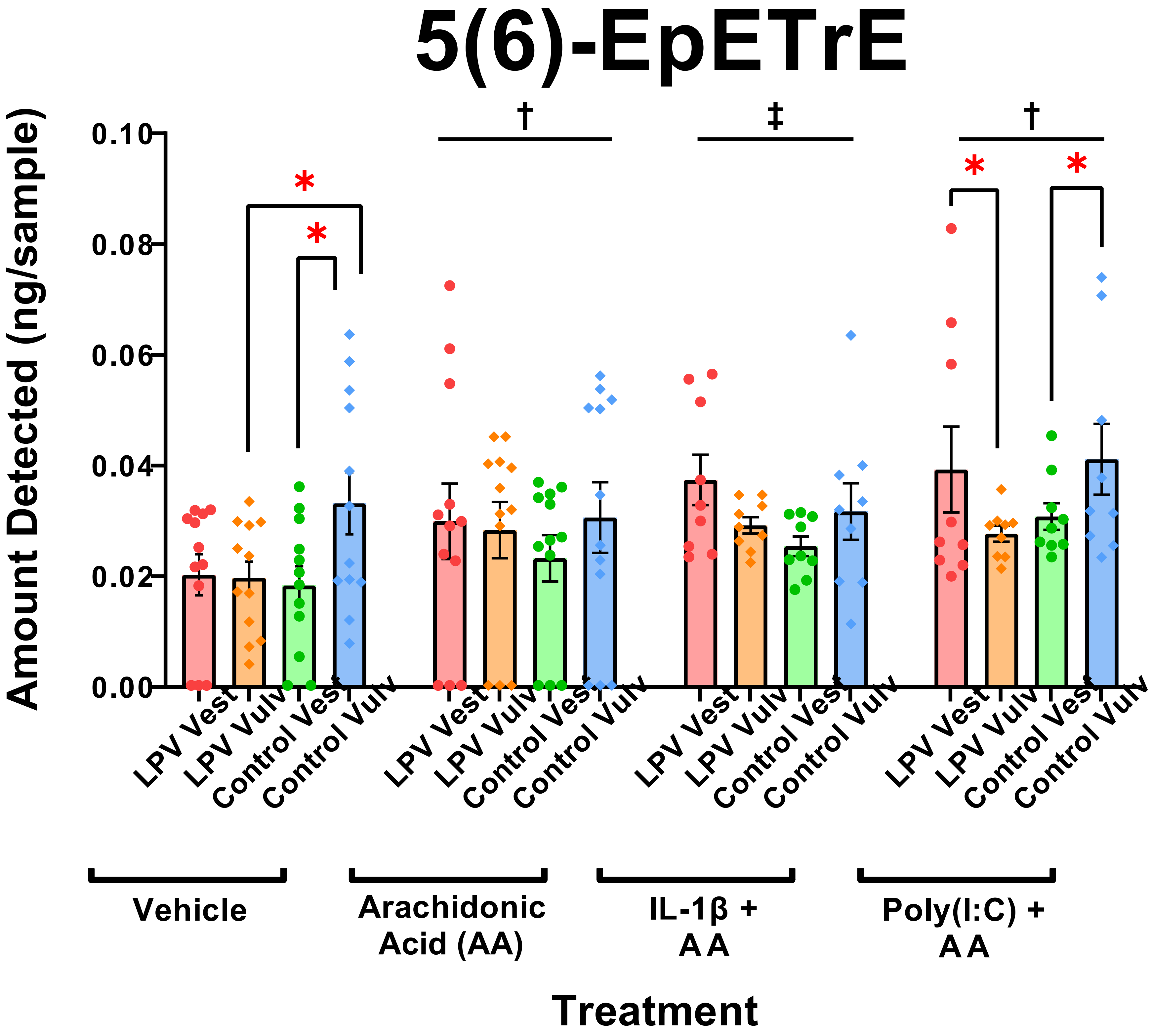

5(6)-EpETrE
Amount Detected (ng/sample)
0.10
0.08
0.06
0.04
0.02
0.00
†
‡
†
*
LPV Vest
LPV Vulv
Control Vest
Control Vulv
Vehicle
Arachidonic Acid (AA)
IL-1β + AA
Poly(I:C) + AA
Treatment

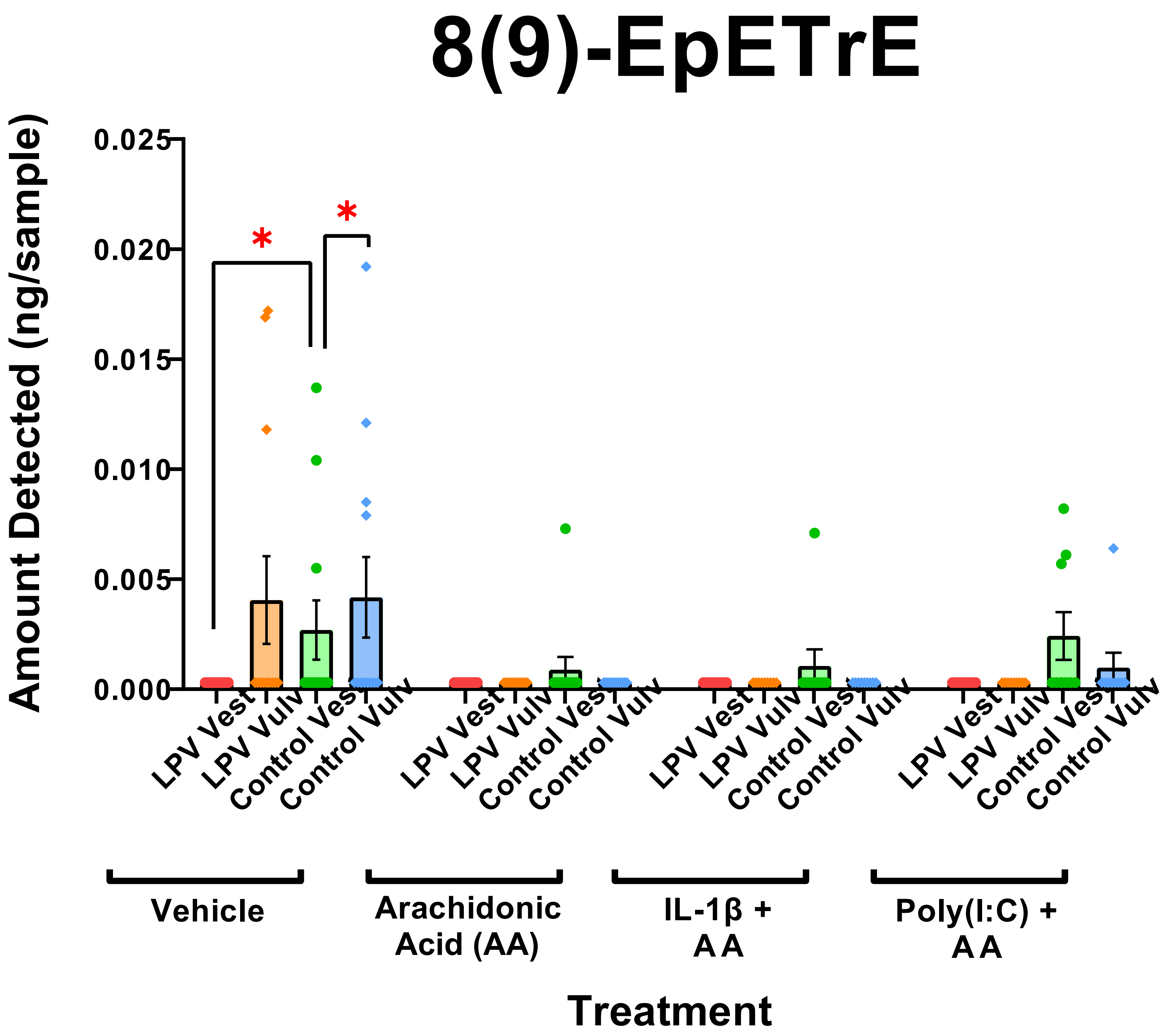

8(9)-EpETrE
Amount Detected (ng/sample)
0.025
0.020
0.015
0.010
0.005
0.000
*
*
LPV Vest
LPV Vulv
Control Vest
Control Vulv
Vehicle
Arachidonic Acid (AA)
IL-1β + AA
Poly(I:C) + AA
Treatment

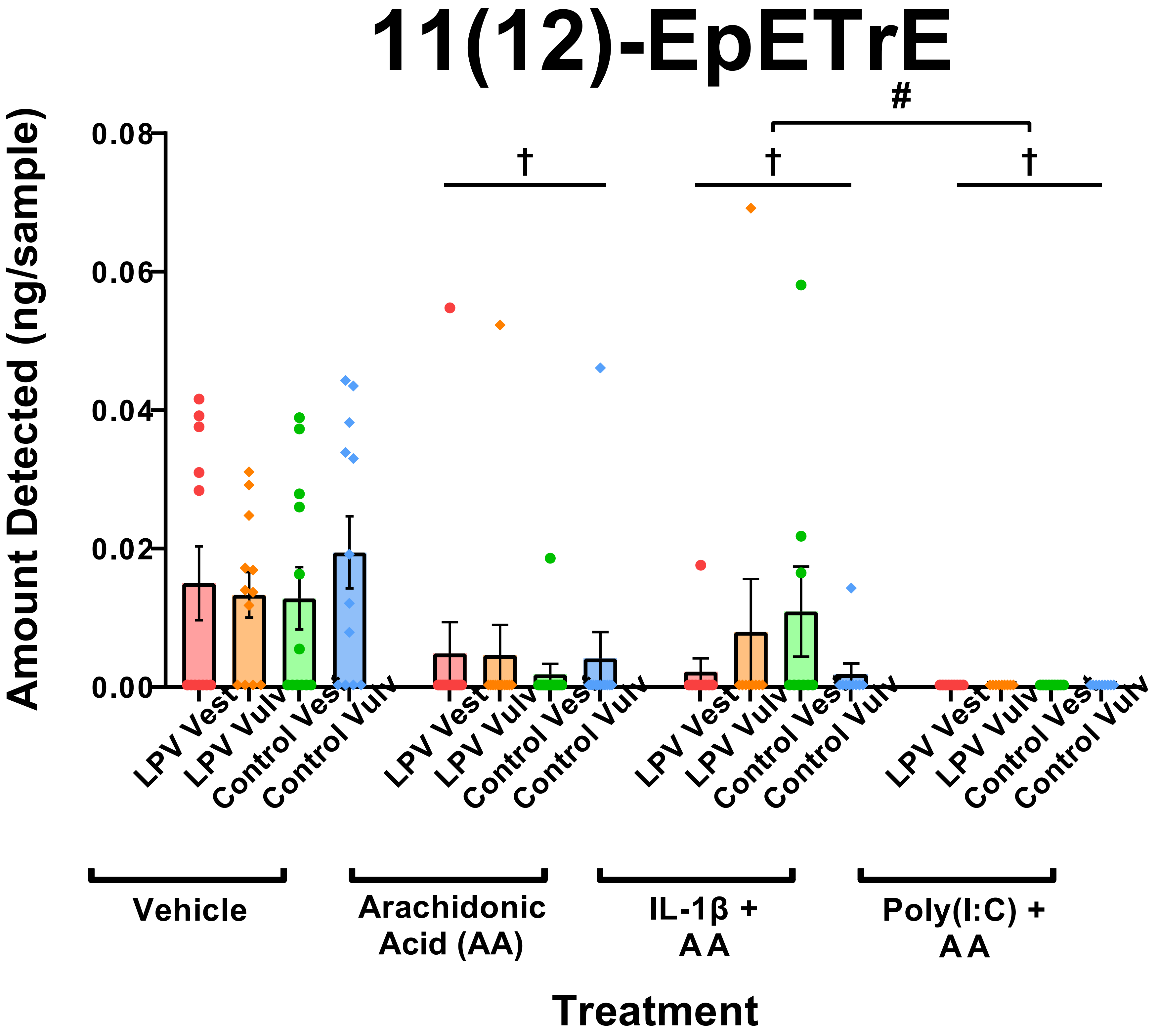

11(12)-EpETrE
Amount Detected (ng/sample)
0.08
0.06
0.04
0.02
0.00
#
†
†
†
LPV Vest
LPV Vulv
Control Vest
Control Vulv
Vehicle
Arachidonic Acid (AA)
IL-1β + AA
Poly(I:C) + AA
Treatment

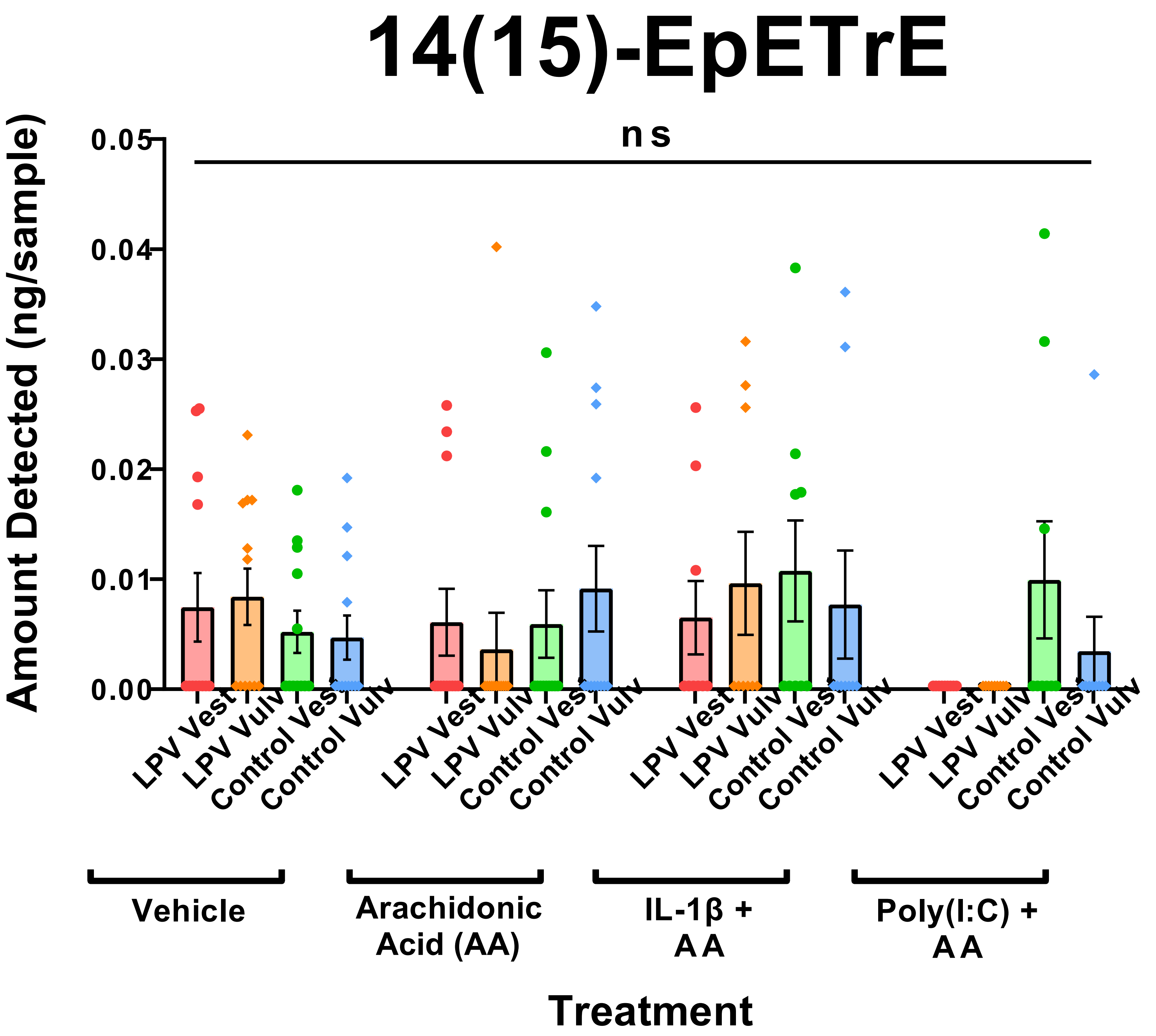

14(15)-EpETrE
ns
Amount Detected (ng/sample)
0.05
0.04
0.03
0.02
0.01
0.00
LPV Vest
LPV Vulv
Control Vest
Control Vulv
Vehicle
Arachidonic Acid (AA)
IL-1β + AA
Poly(I:C) + AA
Treatment

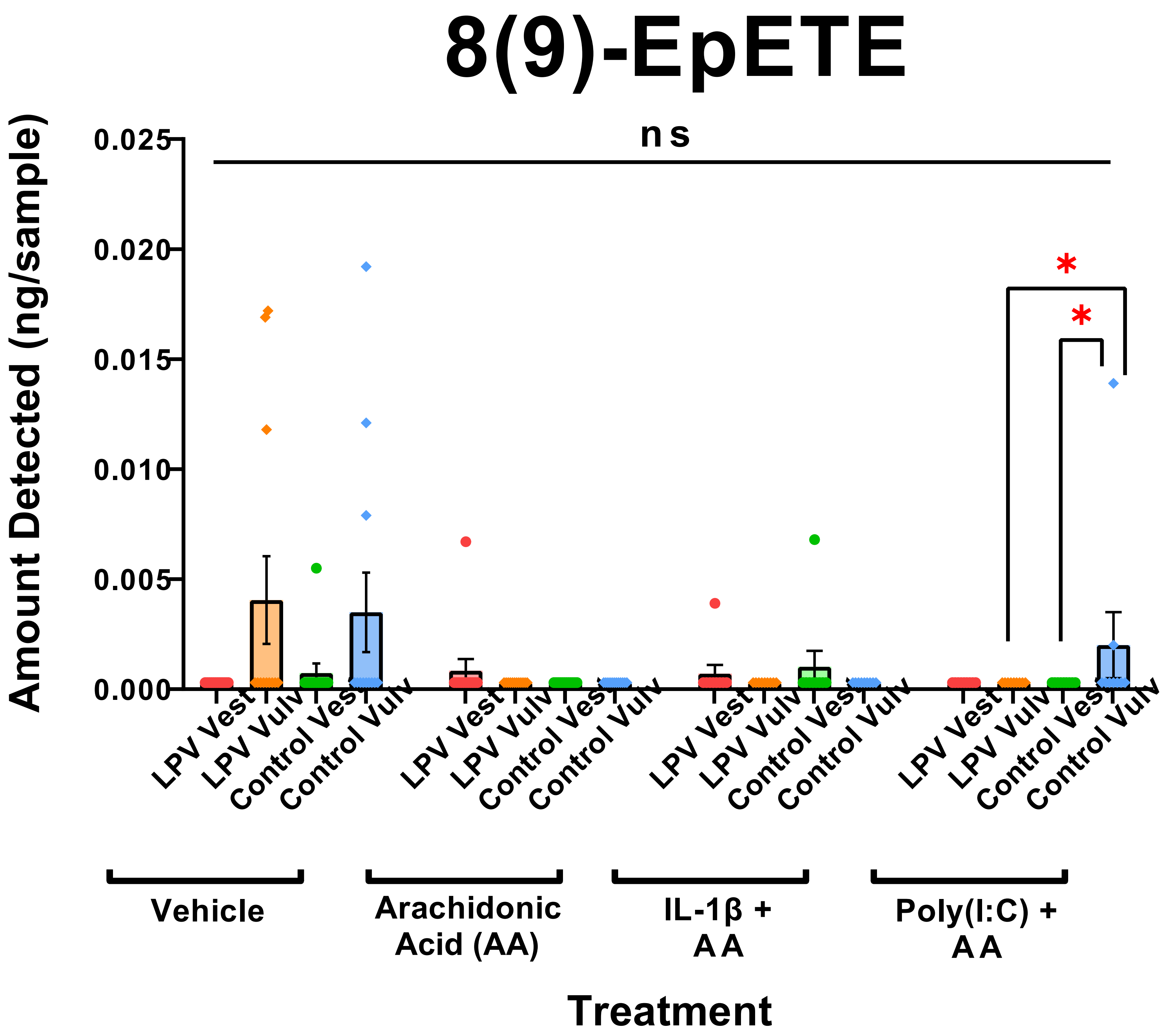

8(9)-EpETE
Amount Detected (ng/sample)
0.025
0.020
0.015
0.010
0.005
0.000
ns
*
*
LPV Vest
LPV Vulv
Control Vest
Control Vulv
Vehicle
Arachidonic Acid (AA)
IL-1β + AA
Poly(I:C) + AA
Treatment

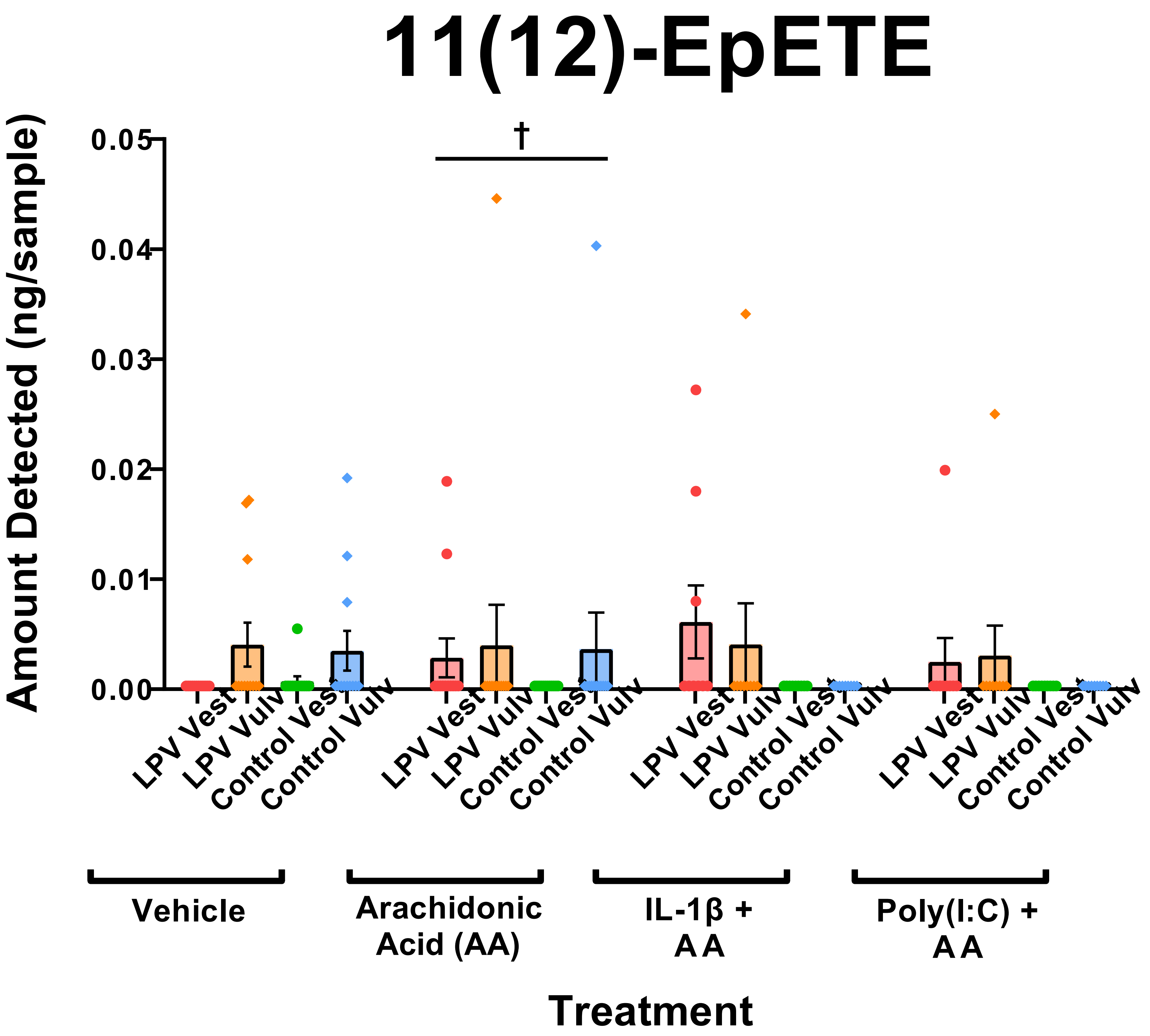

11(12)-EpETE
Amount Detected (ng/sample)
0.05
0.04
0.03
0.02
0.01
0.00
†
LPV Vest
LPV Vulv
Control Vest
Control Vulv
Vehicle
Arachidonic Acid (AA)
IL-1β + AA
Poly(I:C) + AA
Treatment

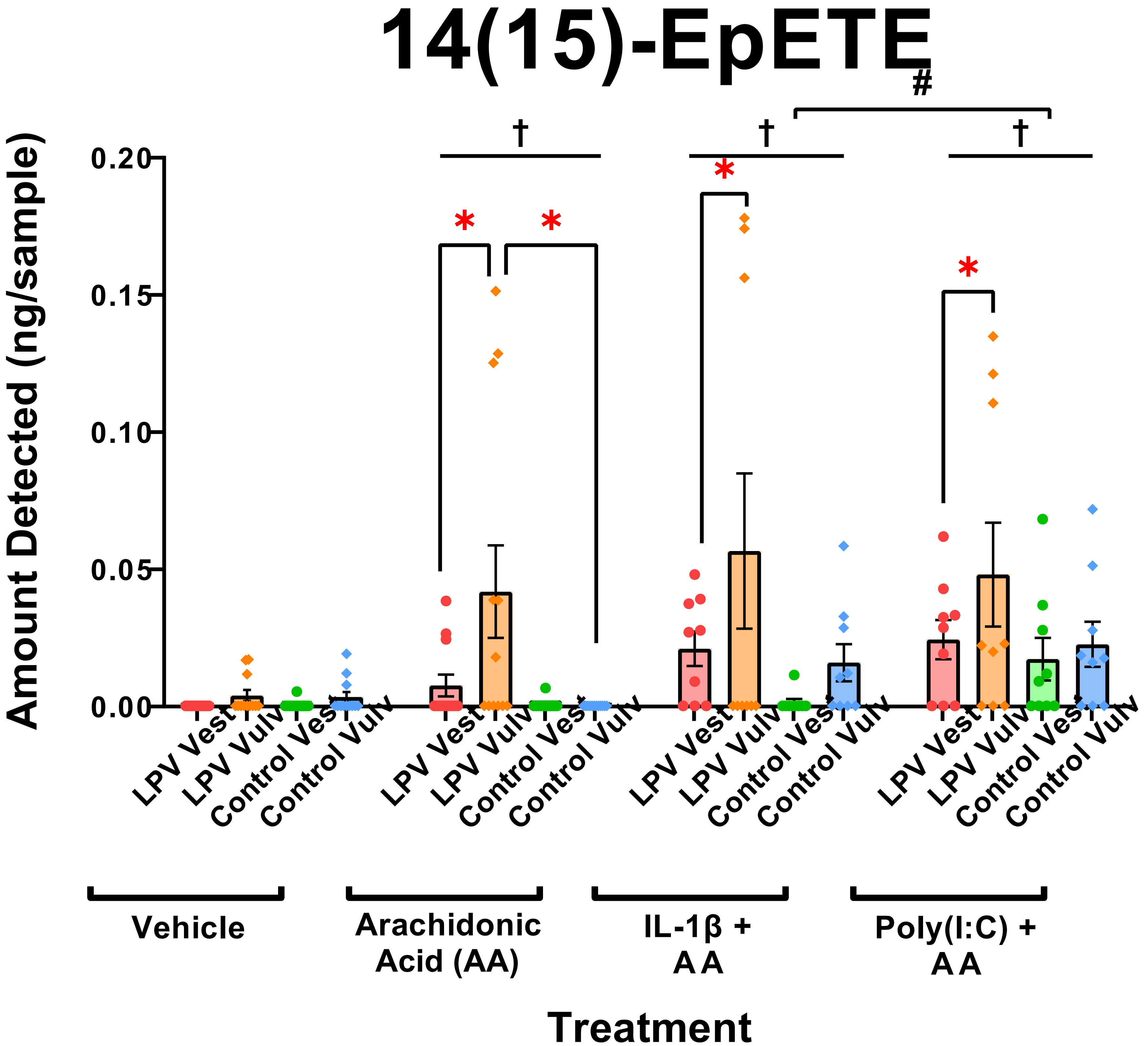

14(15)-EpETE
Amount Detected (ng/sample)
0.00
0.05
0.10
0.15
0.20
#
†
†
†
*
*
*
*
LPV Vest
LPV Vulv
Control Vest
Control Vulv
Vehicle
Arachidonic Acid (AA)
IL-1β + AA
Poly(I:C) + AA
Treatment

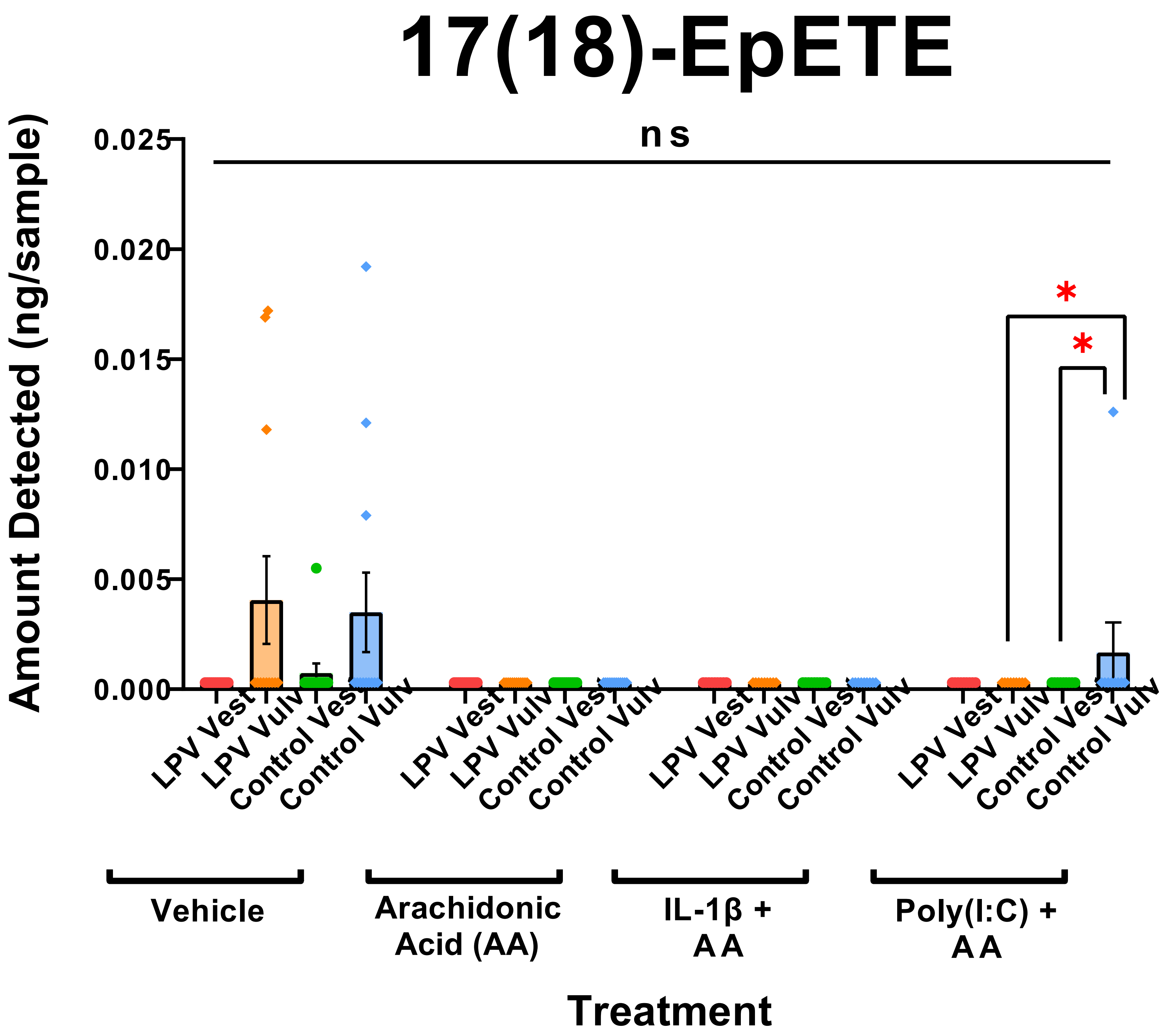

17(18)-EpETE
Amount Detected (ng/sample)
0.025
0.020
0.015
0.010
0.005
0.000
ns
*
*
LPV Vest
LPV Vulv
Control Vest
Control Vulv
Vehicle
Arachidonic Acid (AA)
IL-1β + AA
Poly(I:C) + AA
Treatment

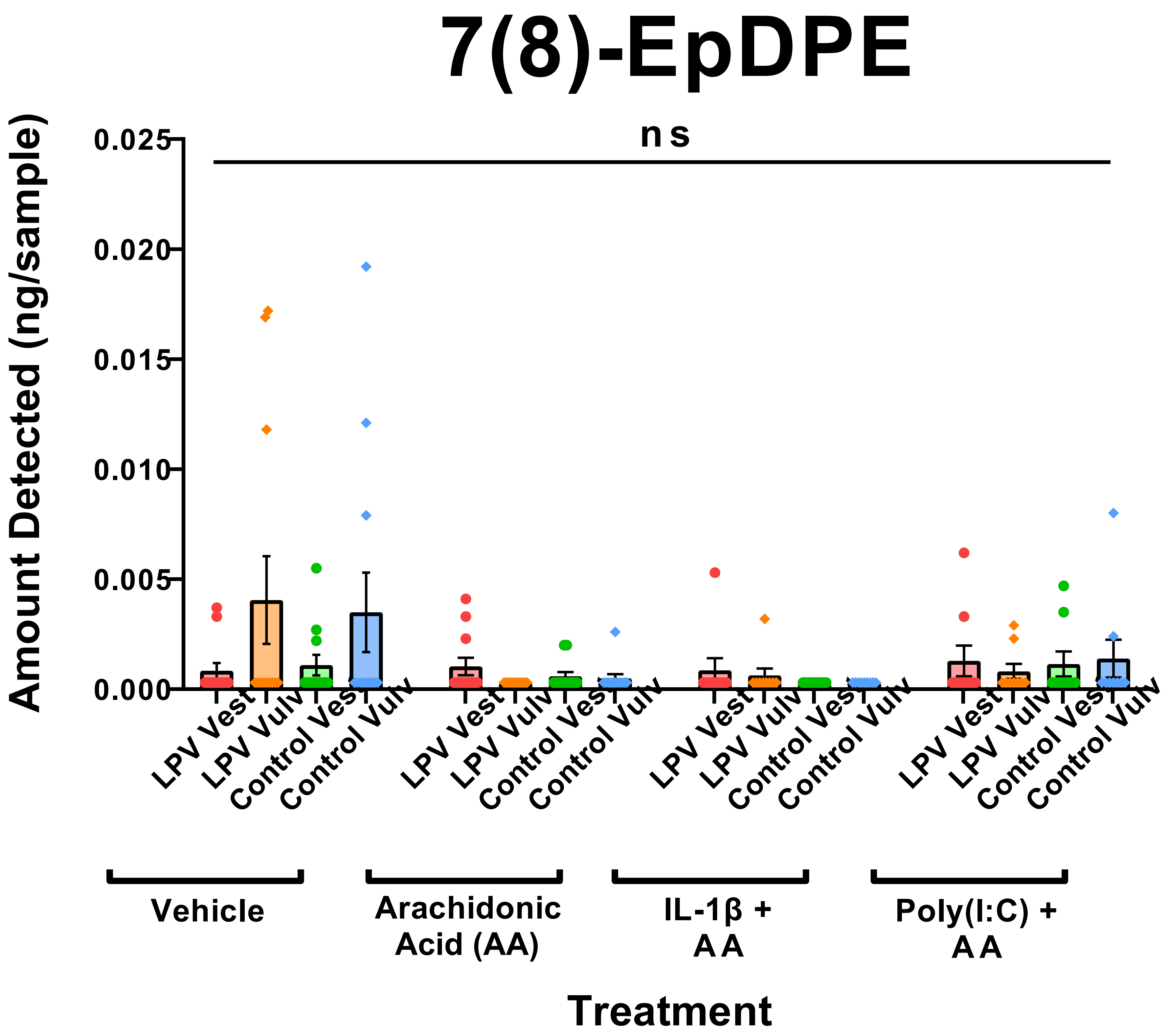

7(8)-EpDPE
ns
Amount Detected (ng/sample)
0.025
0.020
0.015
0.010
0.005
0.000
LPV Vest
LPV Vulv
Control Vest
Control Vulv
Vehicle
Arachidonic Acid (AA)
IL-1β + AA
Poly(I:C) + AA
Treatment

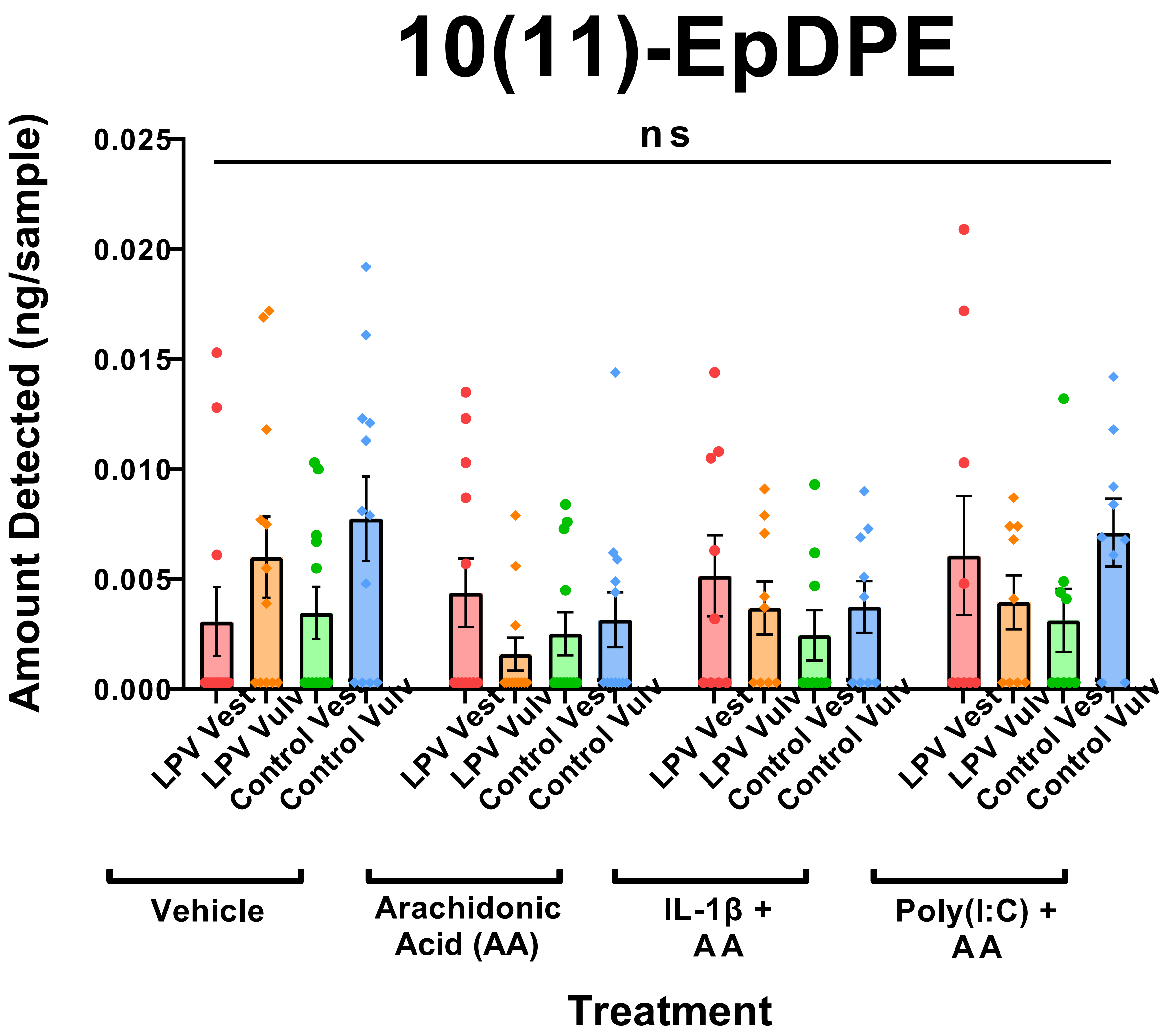
10(11)-EpDPE
Amount Detected (ng/sample)
0.025
0.020
0.015
0.010
0.005
0.000
ns
LPV Vest
LPV Vulv
Control Vest
Control Vulv
Vehicle
Arachidonic Acid (AA)
IL-1β + AA
Poly(I:C) + AA
Treatment

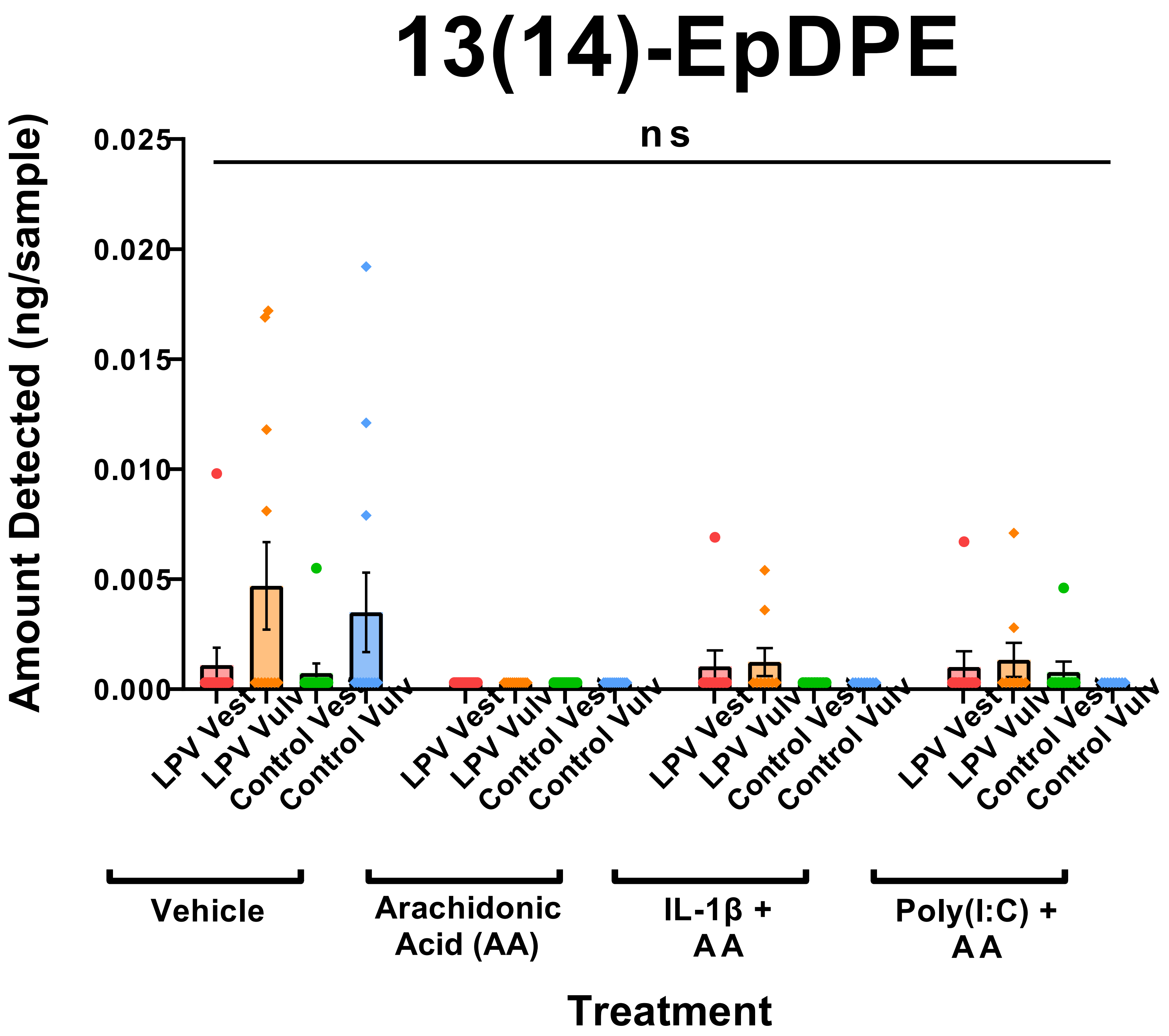

13(14)-EpDPE
ns
Amount Detected (ng/sample)
0.025
0.020
0.015
0.010
0.005
0.000
LPV Vest
LPV Vulv
Control Vest
Control Vulv
Vehicle
Arachidonic Acid (AA)
IL-1β + AA
Poly(I:C) + AA
Treatment

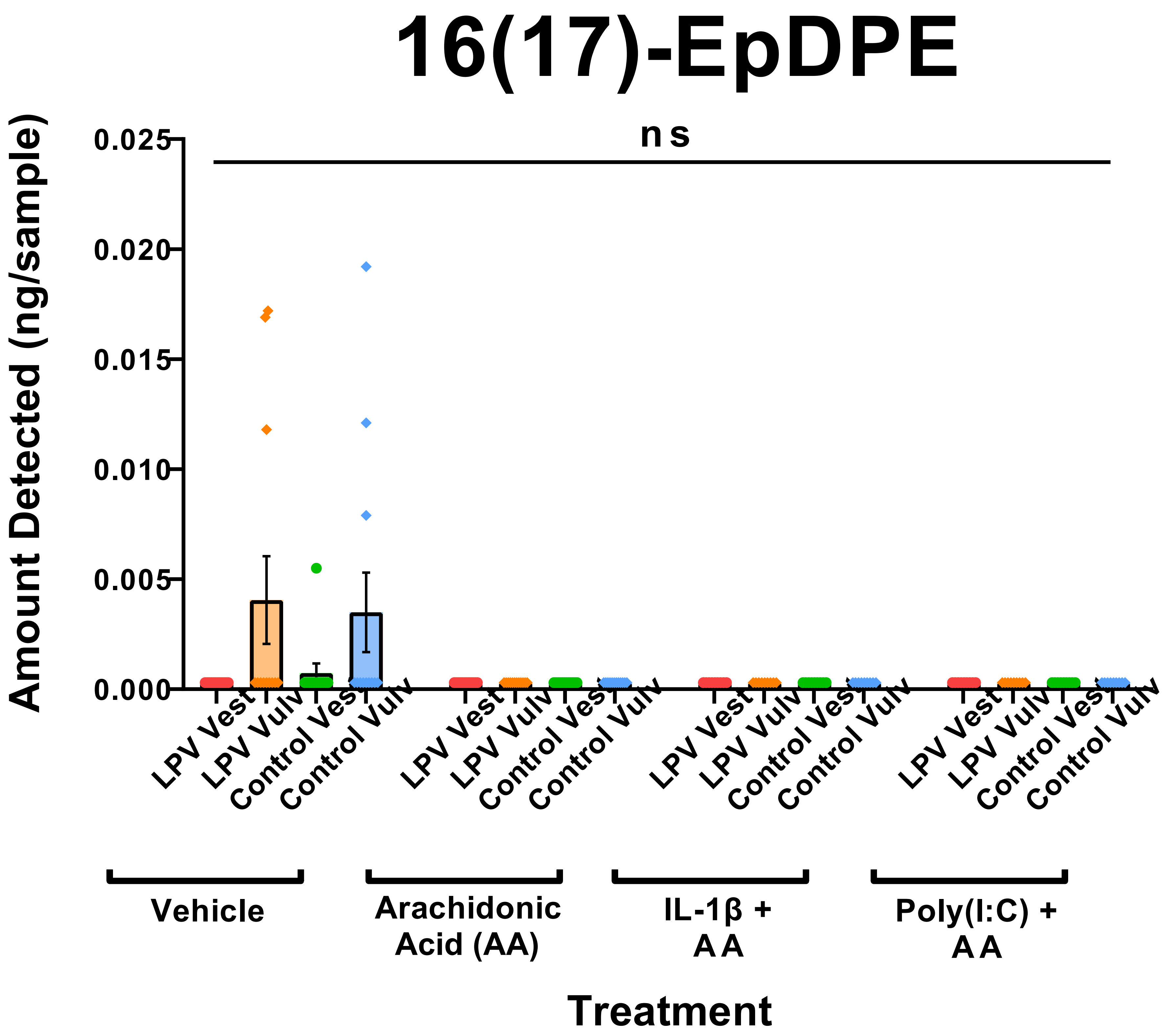
16(17)-EpDPE
ns
Amount Detected (ng/sample)
0.025
0.020
0.015
0.010
0.005
0.000
LPV Vest
LPV Vulv
Control Vest
Control Vulv
LPV Vest
LPV Vulv
Control Vest
Control Vulv
LPV Vest
LPV Vulv
Control Vest
Control Vulv
LPV Vest
LPV Vulv
Control Vest
Control Vulv
Vehicle
Arachidonic Acid (AA)
IL-1β + AA
Poly(I:C) + AA
Treatment

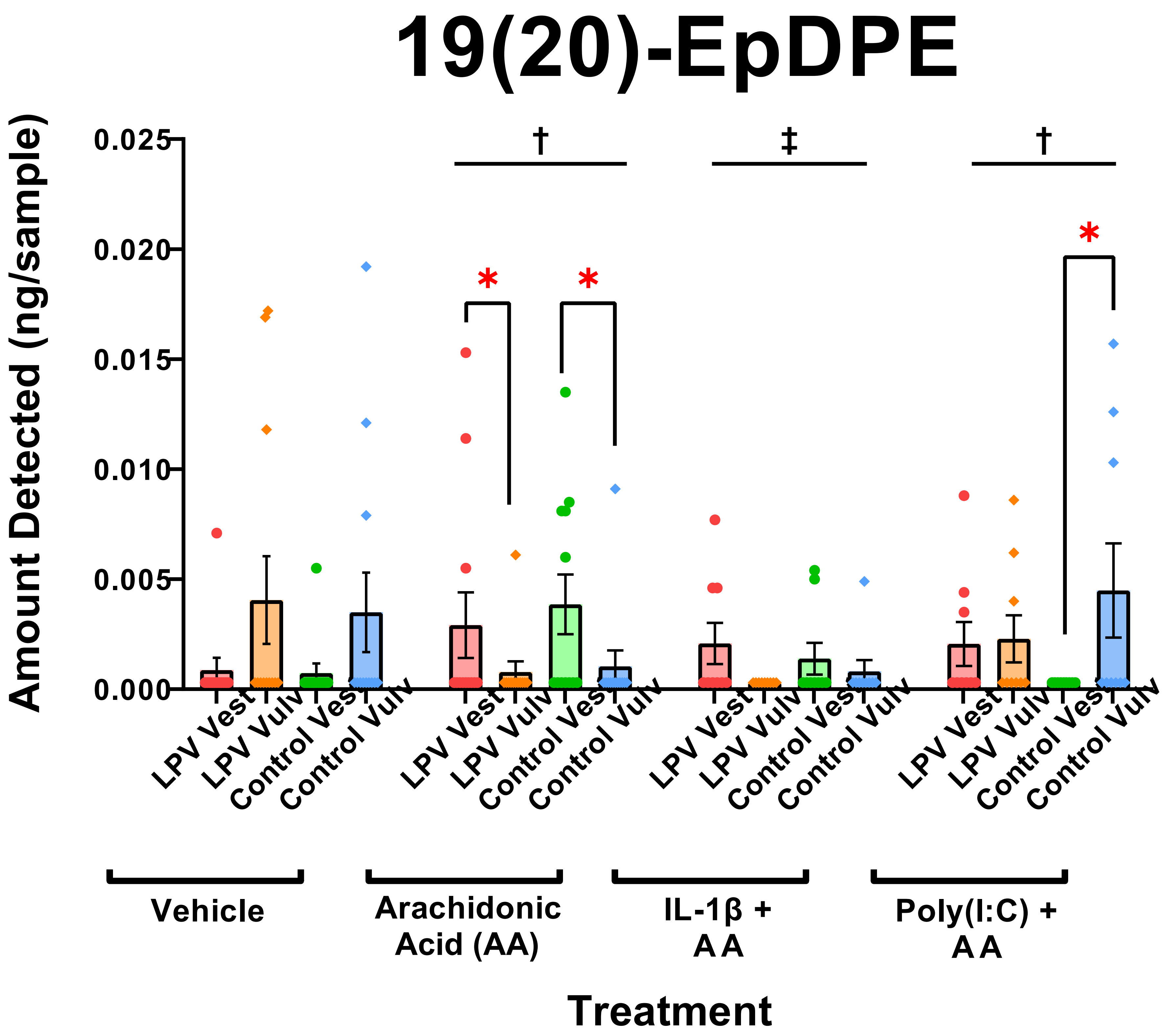

19(20)-EpDPE
Amount Detected (ng/sample)
0.025
0.020
0.015
0.010
0.005
0.000
†
‡
†
*
*
*
LPV Vest
LPV Vulv
Control Vest
Control Vulv
Vehicle
Arachidonic Acid (AA)
IL-1β + AA
Poly(I:C) + AA
Treatment

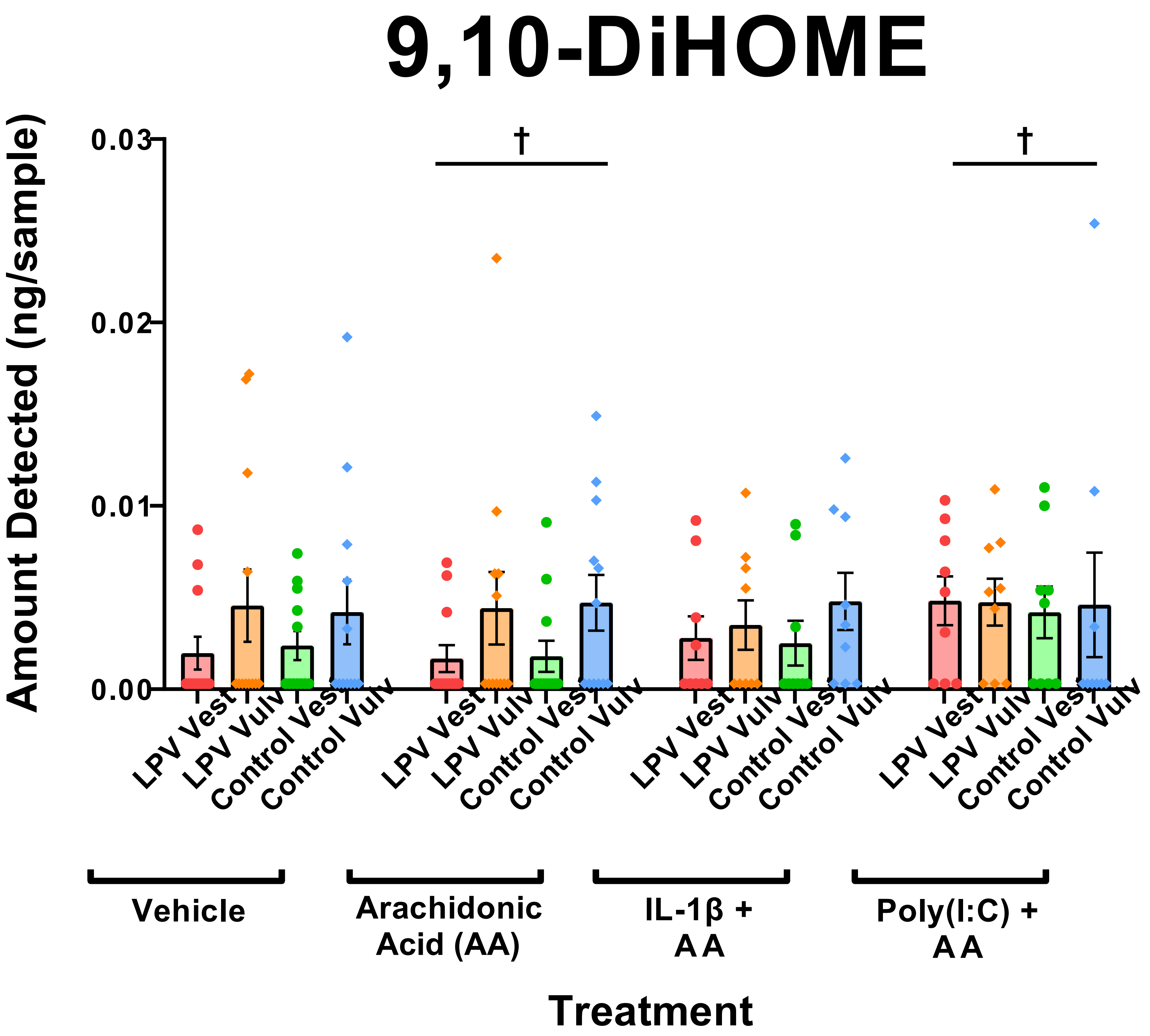

9,10-DiHOME
Amount Detected (ng/sample)
0.03
0.02
0.01
0.00
†
†
LPV Vest
LPV Vulv
Control Vest
Control Vulv
Vehicle
Arachidonic Acid (AA)
IL-1β + AA
Poly(I:C) + AA
Treatment

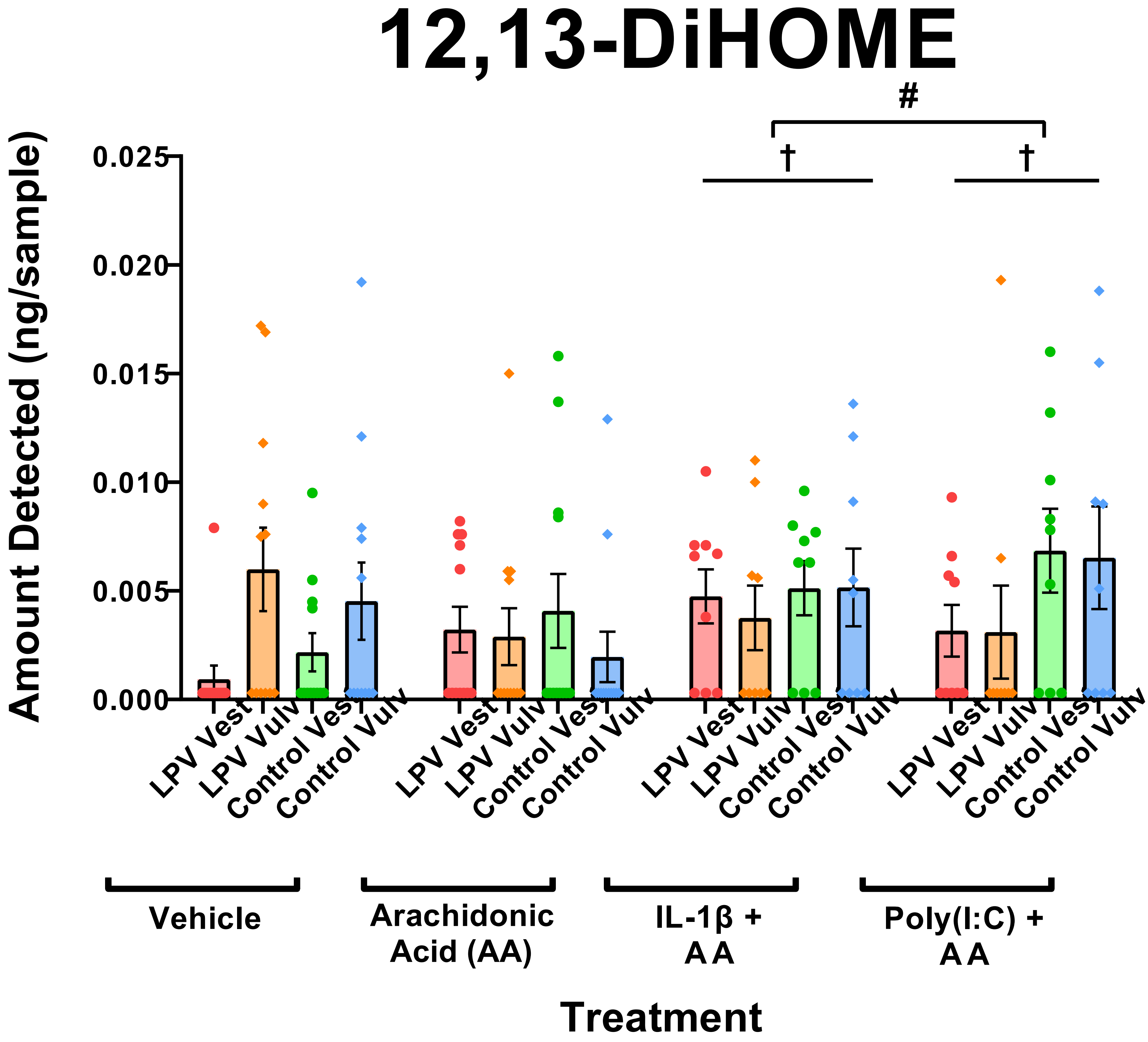
12,13-DiHOME
#
†
†
Amount Detected (ng/sample)
0.025
0.020
0.015
0.010
0.005
0.000
LPV Vest
LPV Vulv
Control Vest
Control Vulv
Vehicle
Arachidonic Acid (AA)
IL-1β + AA
Poly(I:C) + AA
Treatment

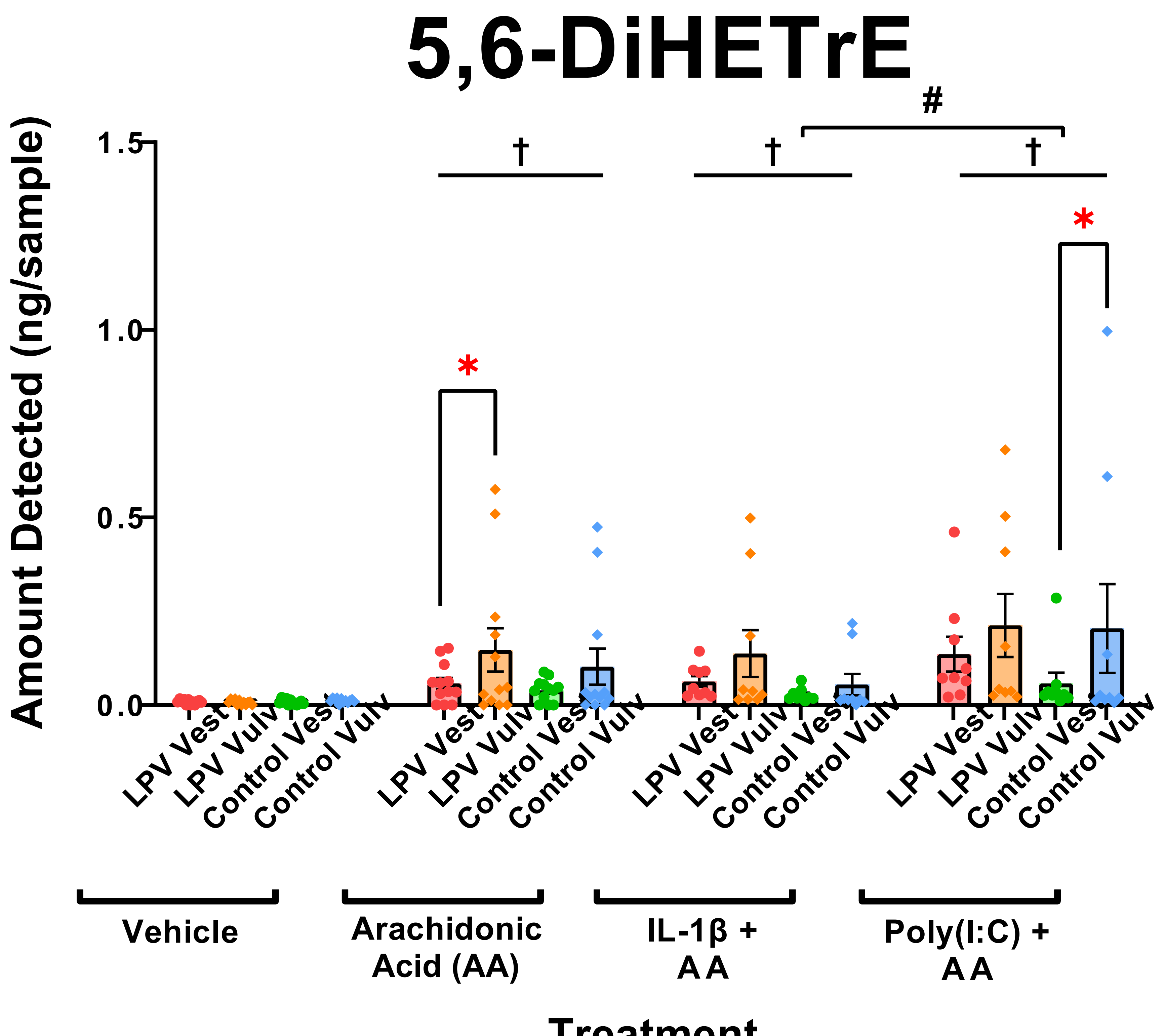
5,6-DiHETrE
Amount Detected (ng/sample)
1.5
1.0
0.5
0.0
#
†
†
†
*
*
LPV Vest
LPV Vulv
Control Vest
Control Vulv
Vehicle
Arachidonic Acid (AA)
IL-1β + AA
Poly(I:C) + AA
Treatment

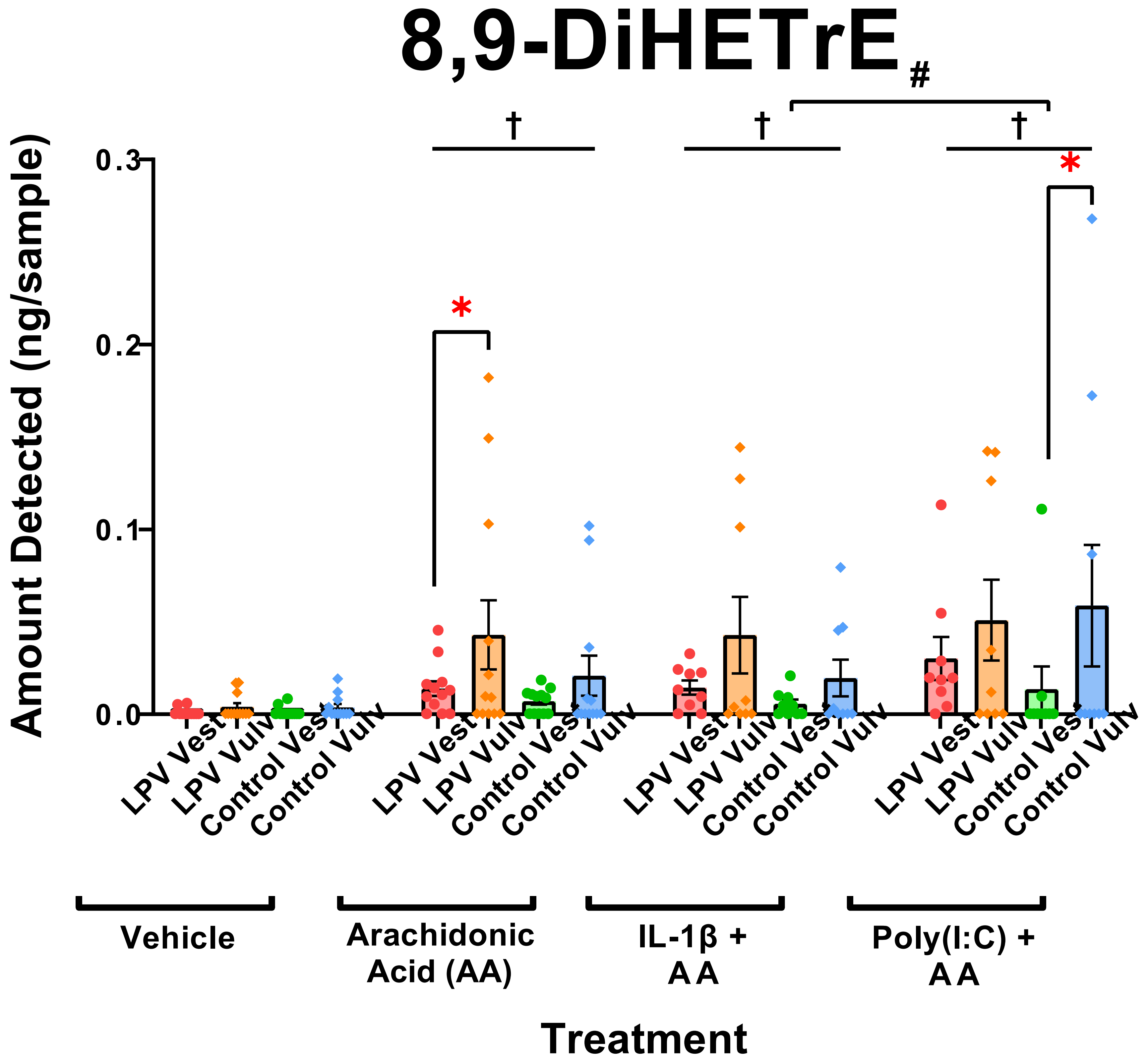

8,9-DiHETrE #
Amount Detected (ng/sample)
0.0
0.1
0.2
0.3
†
†
†
*
*
LPV Vest
LPV Vulv
Control Vest
Control Vulv
Vehicle
Arachidonic Acid (AA)
IL-1β + AA
Poly(I:C) + AA
Treatment

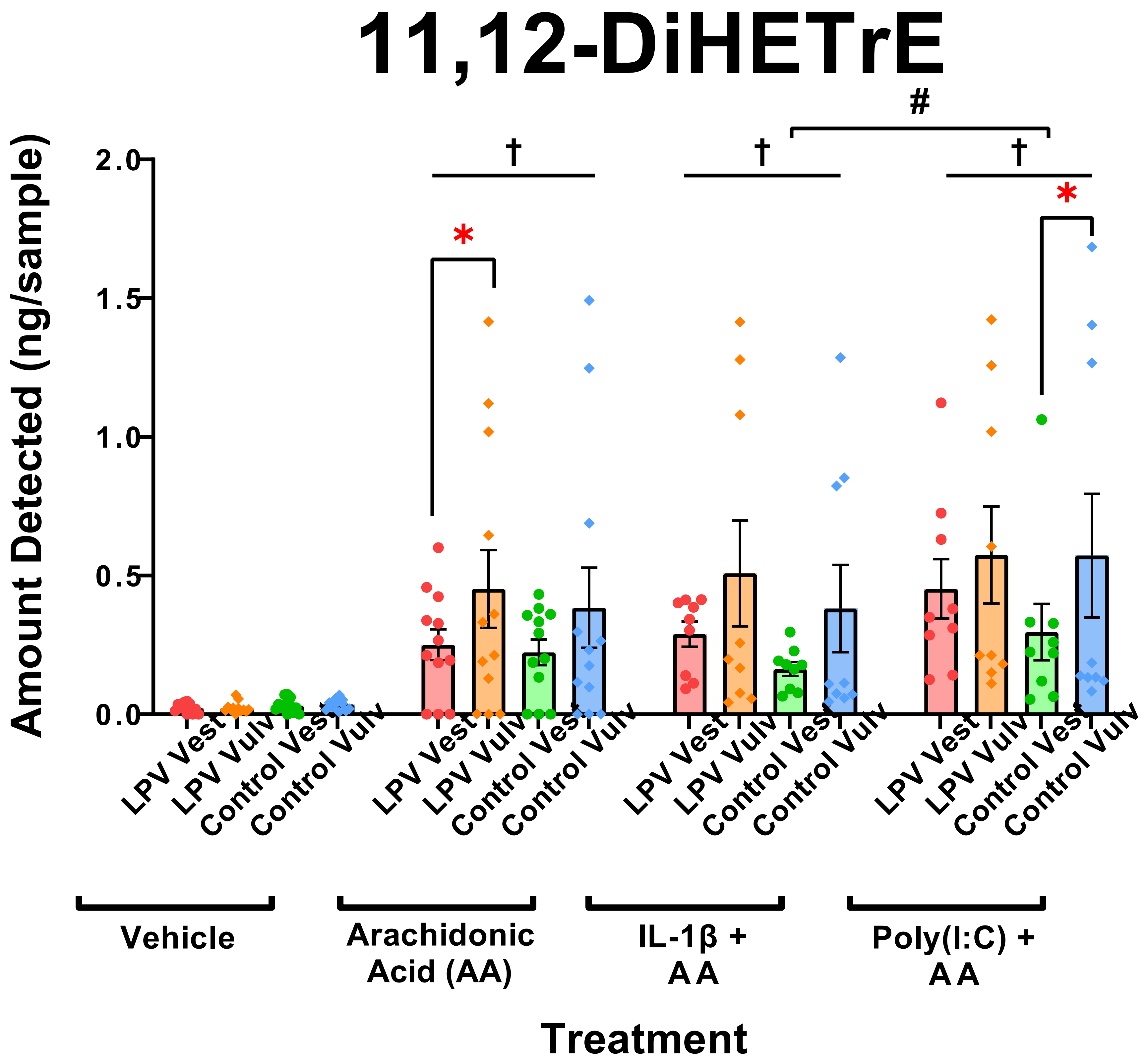

11,12-DiHETrE
Amount Detected (ng/sample)
2.0
1.5
1.0
0.5
0.0
#
†
†
†
*
*
LPV Vest
LPV Vulv
Control Vest
Control Vulv
Vehicle
Arachidonic Acid (AA)
IL-1β + AA
Poly(I:C) + AA
Treatment

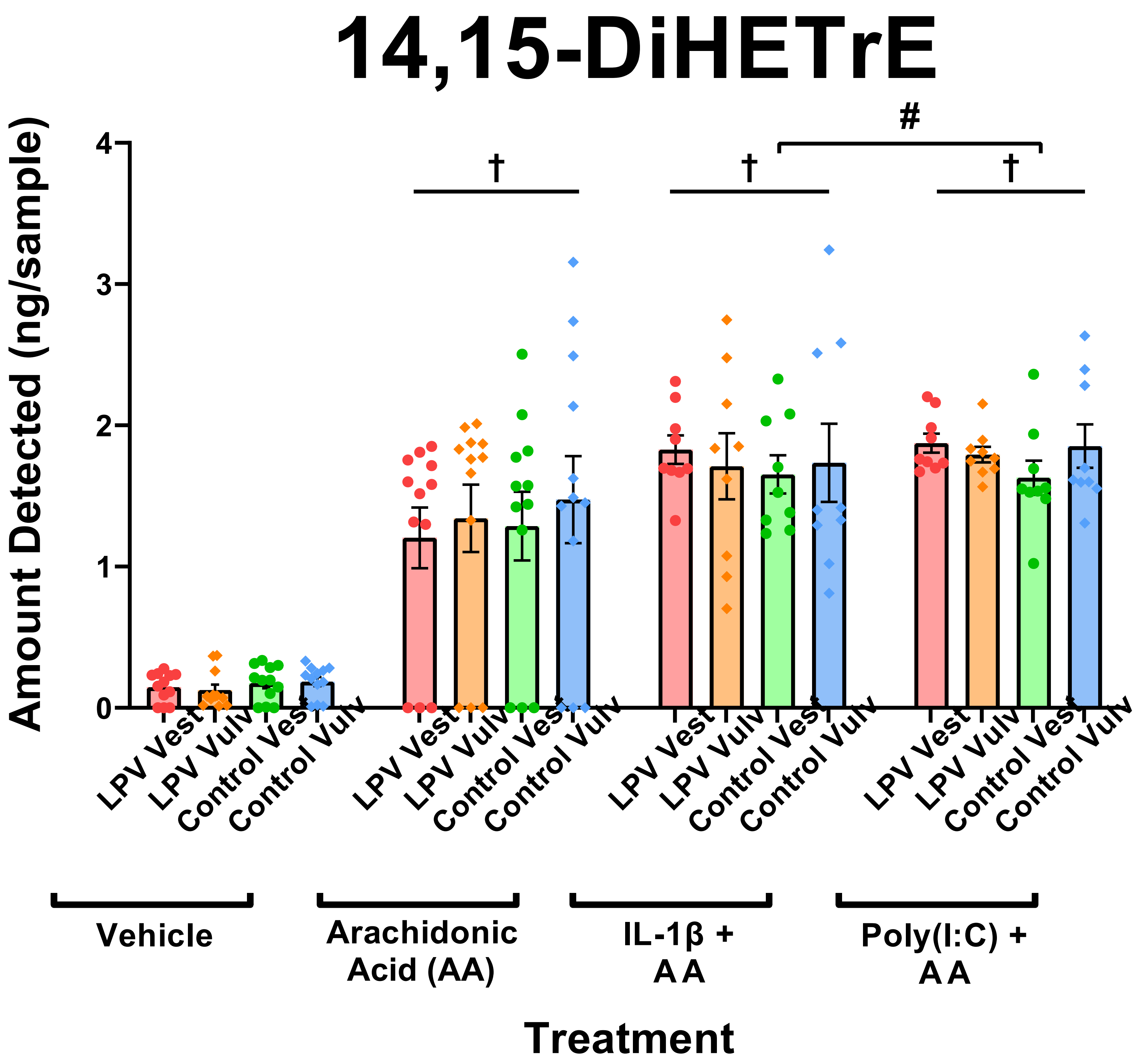

14,15-DiHETrE
Amount Detected (ng/sample)
0
1
2
3
4
†
†
†
#
LPV Vest
LPV Vulv
Control Vest
Control Vulv
Vehicle
Arachidonic Acid (AA)
IL-1β + AA
Poly(I:C) + AA
Treatment

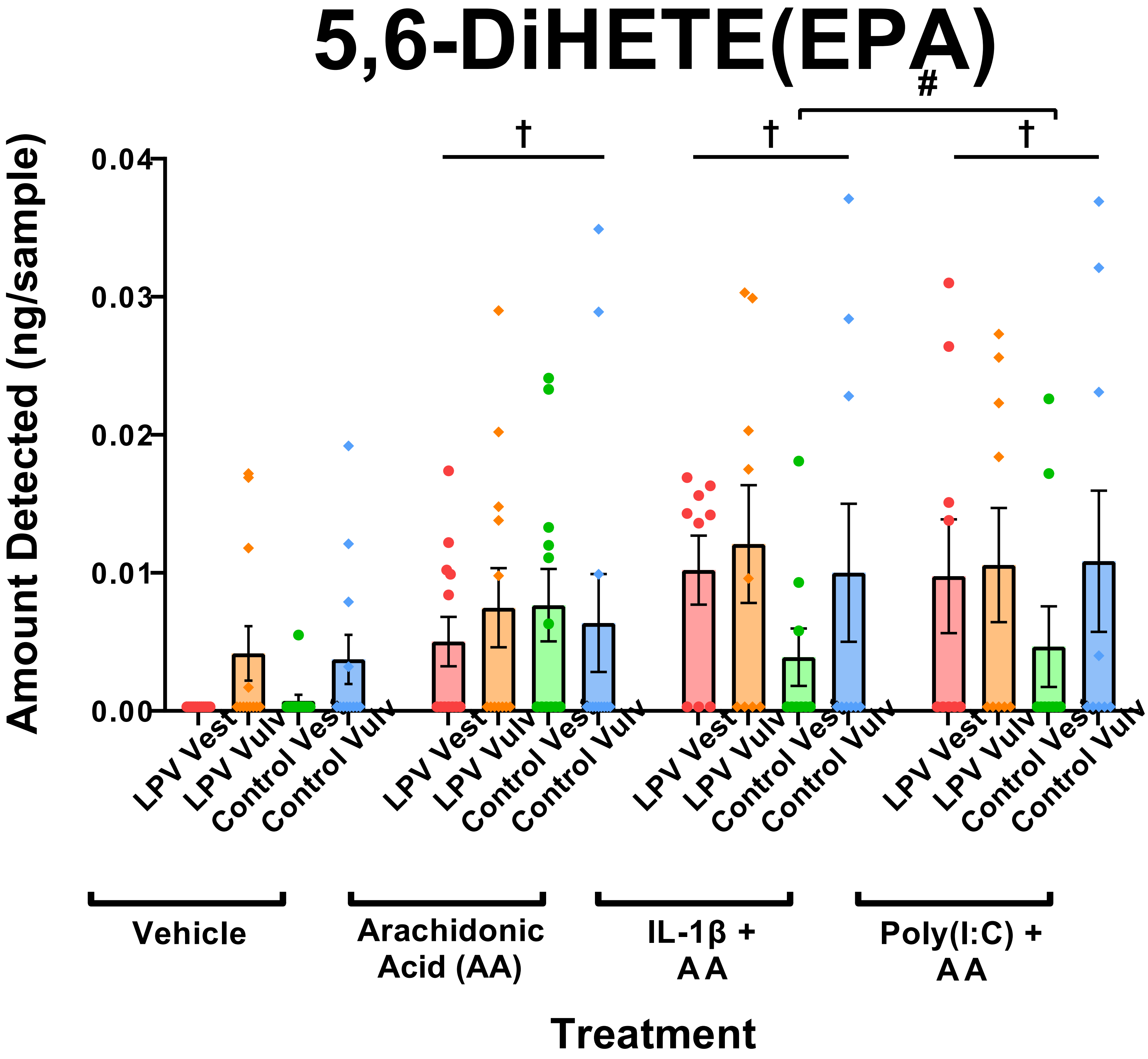
5,6-DiHETE(EPA)
#
†
†
†
Amount Detected (ng/sample)
0.04
0.03
0.02
0.01
0.00
LPV Vest
LPV Vulv
Control Vest
Control Vulv
Vehicle
Arachidonic Acid (AA)
IL-1β + AA
Poly(I:C) + AA
Treatment

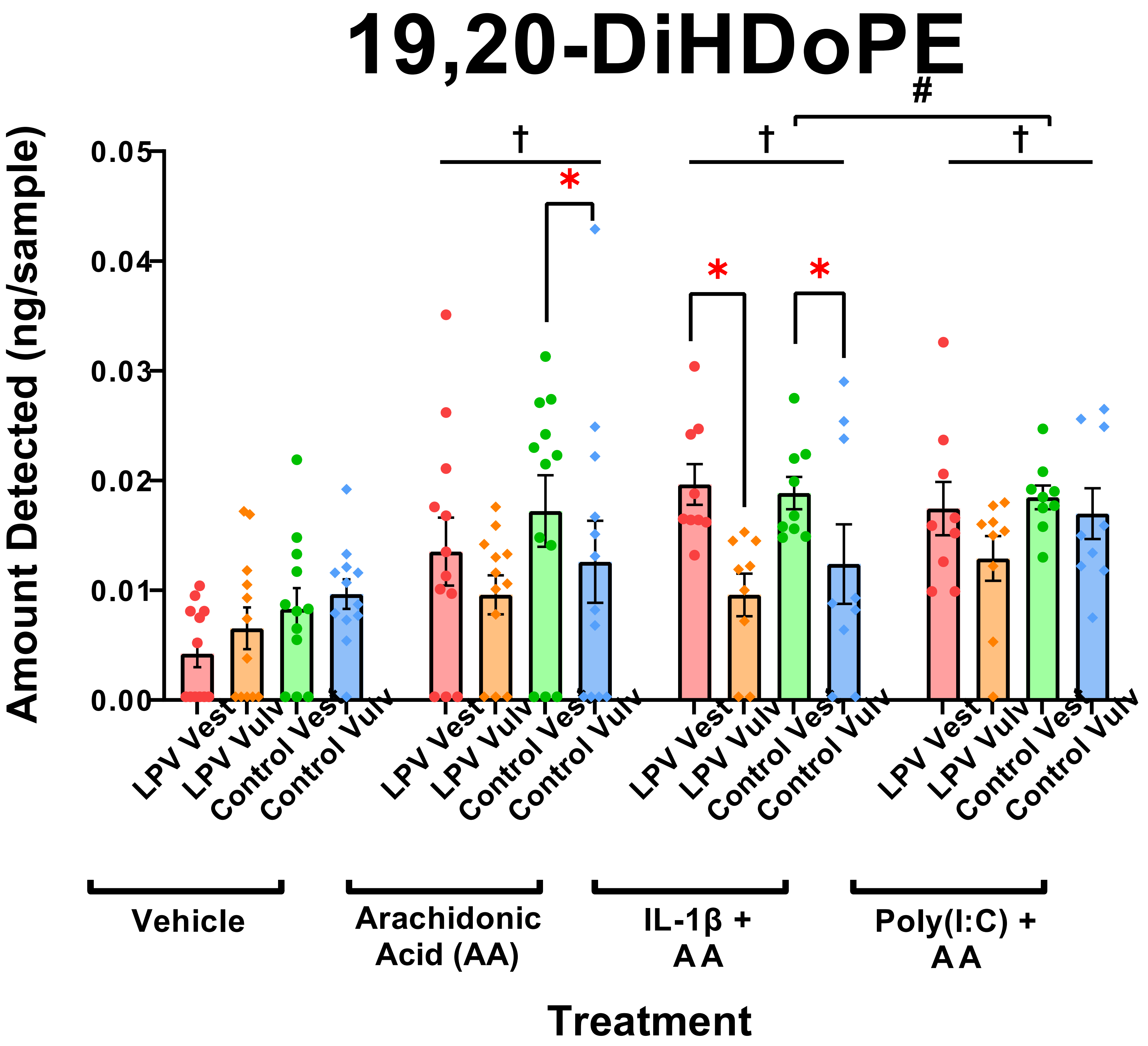

19,20-DiHDoPE
Amount Detected (ng/sample)
0.05
0.04
0.03
0.02
0.01
0.00
#
†
†
†
*
*
*
LPV Vest
LPV Vulv
Control Vest
Control Vulv
Vehicle
Arachidonic Acid (AA)
IL-1β + AA
Poly(I:C) + AA
Treatment

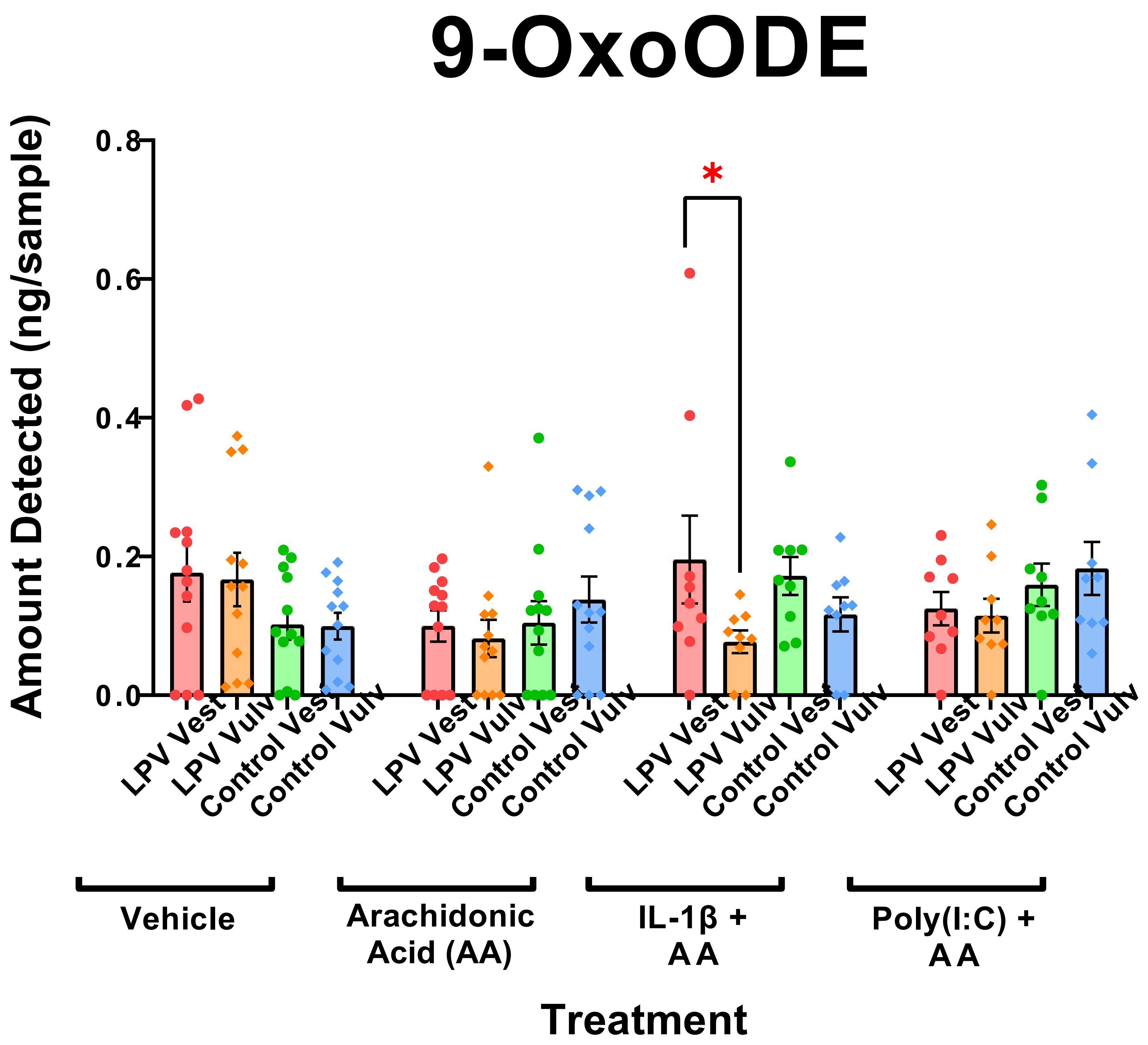

9-OxoODE
Amount Detected (ng/sample)
0.0
0.2
0.4
0.6
0.8
*
LPV Vest
LPV Vulv
Control Vest
Control Vulv
Vehicle
Arachidonic Acid (AA)
IL-1β + AA
Poly(I:C) + AA
Treatment

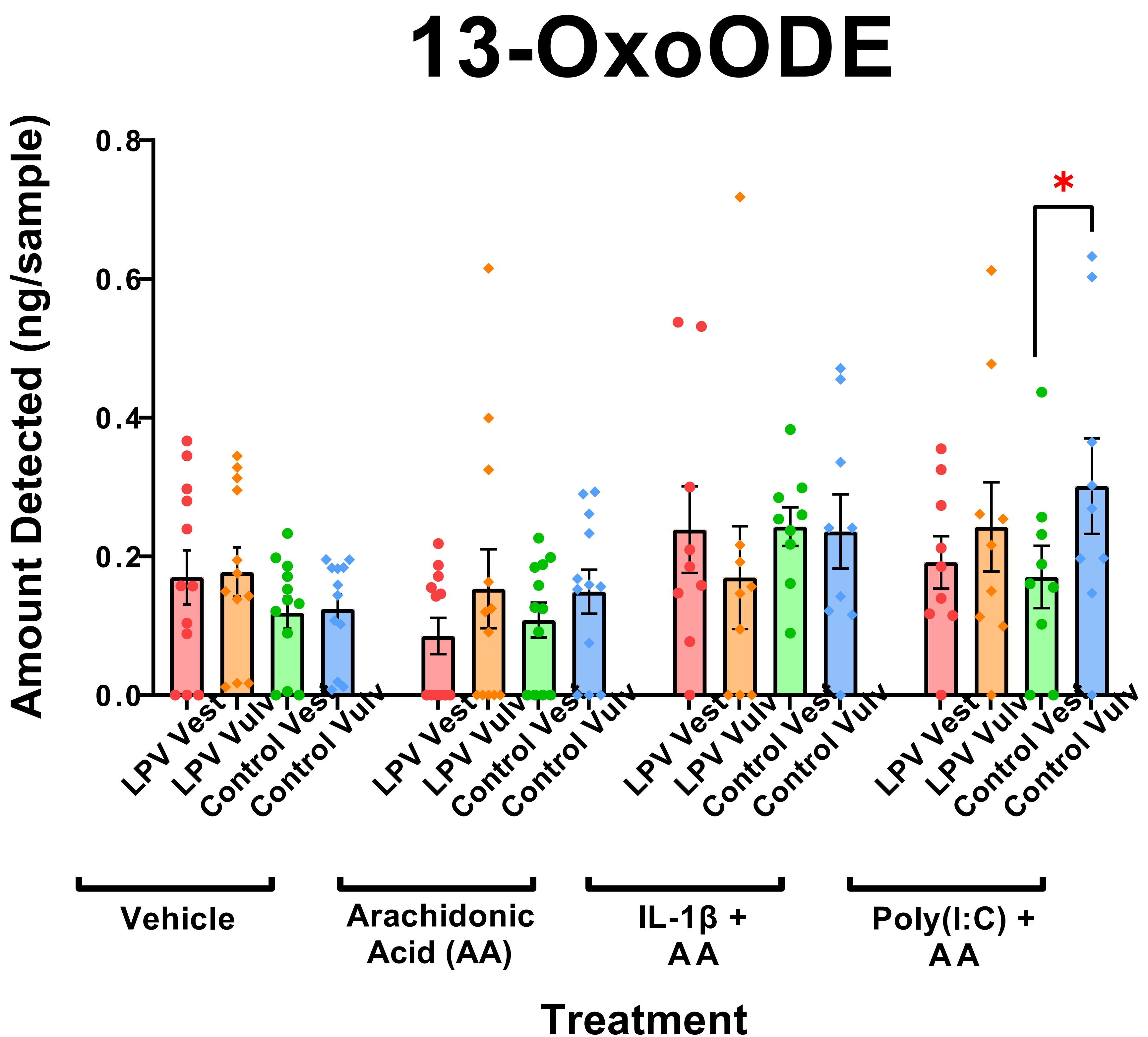

13-OxoODE
Amount Detected (ng/sample)
0.8
0.6
0.4
0.2
0.0
*
LPV Vest
LPV Vulv
Control Vest
Control Vulv
Vehicle
Arachidonic Acid (AA)
IL-1β + AA
Poly(I:C) + AA
Treatment

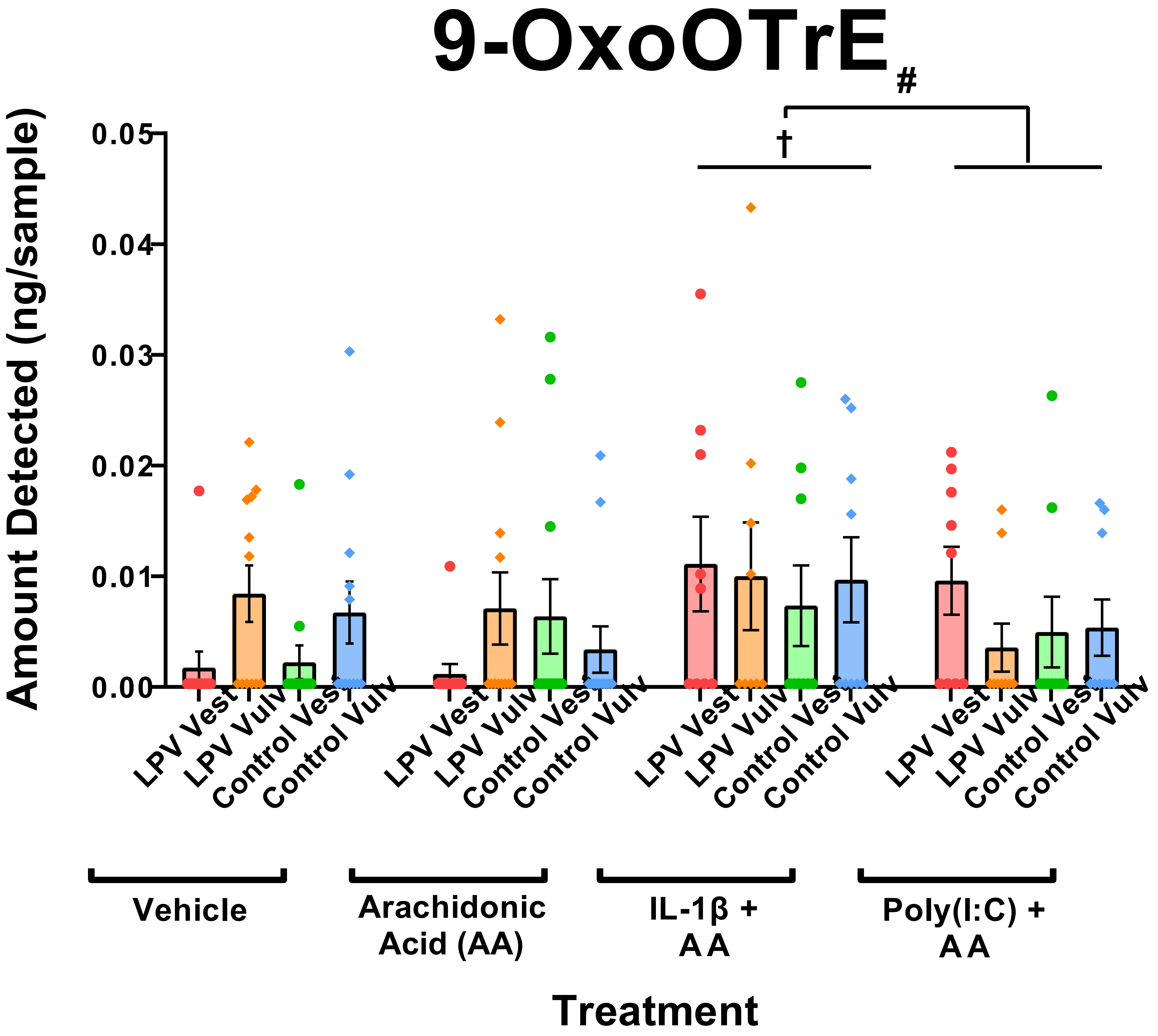

9-OxoOTrE#
†
Amount Detected (ng/sample)
0.05
0.04
0.03
0.02
0.01
0.00
LPV Vest
LPV Vulv
Control Vest
Control Vulv
Vehicle
Arachidonic Acid (AA)
IL-1β + AA
Poly(I:C) + AA
Treatment

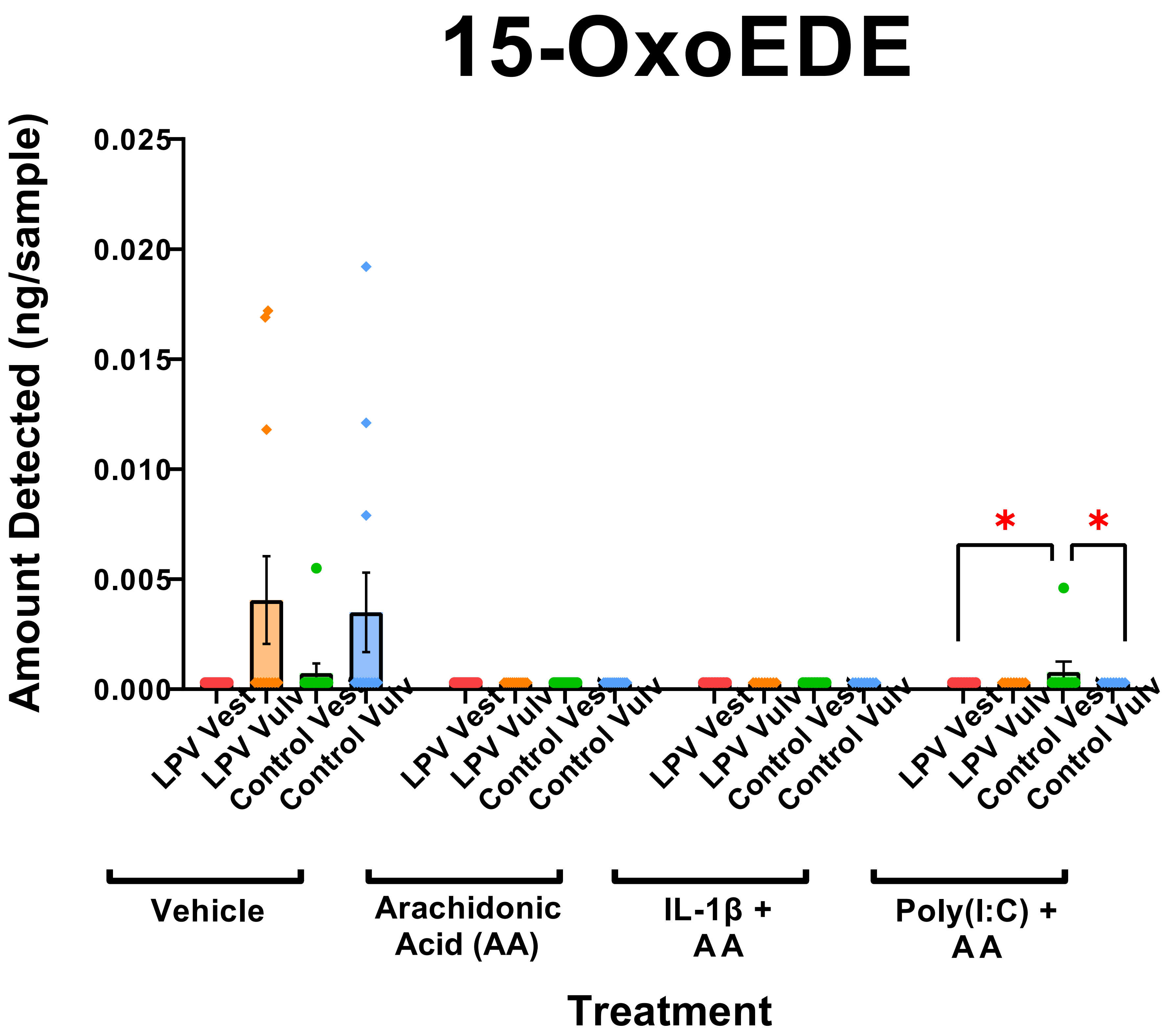
15-OxoEDE
Amount Detected (ng/sample)
0.025
0.020
0.015
0.010
0.005
0.000
LPV Vest
LPV Vulv
Control Vest
Control Vulv
*
*
Vehicle
Arachidonic Acid (AA)
IL-1β + AA
Poly(I:C) + AA
Treatment

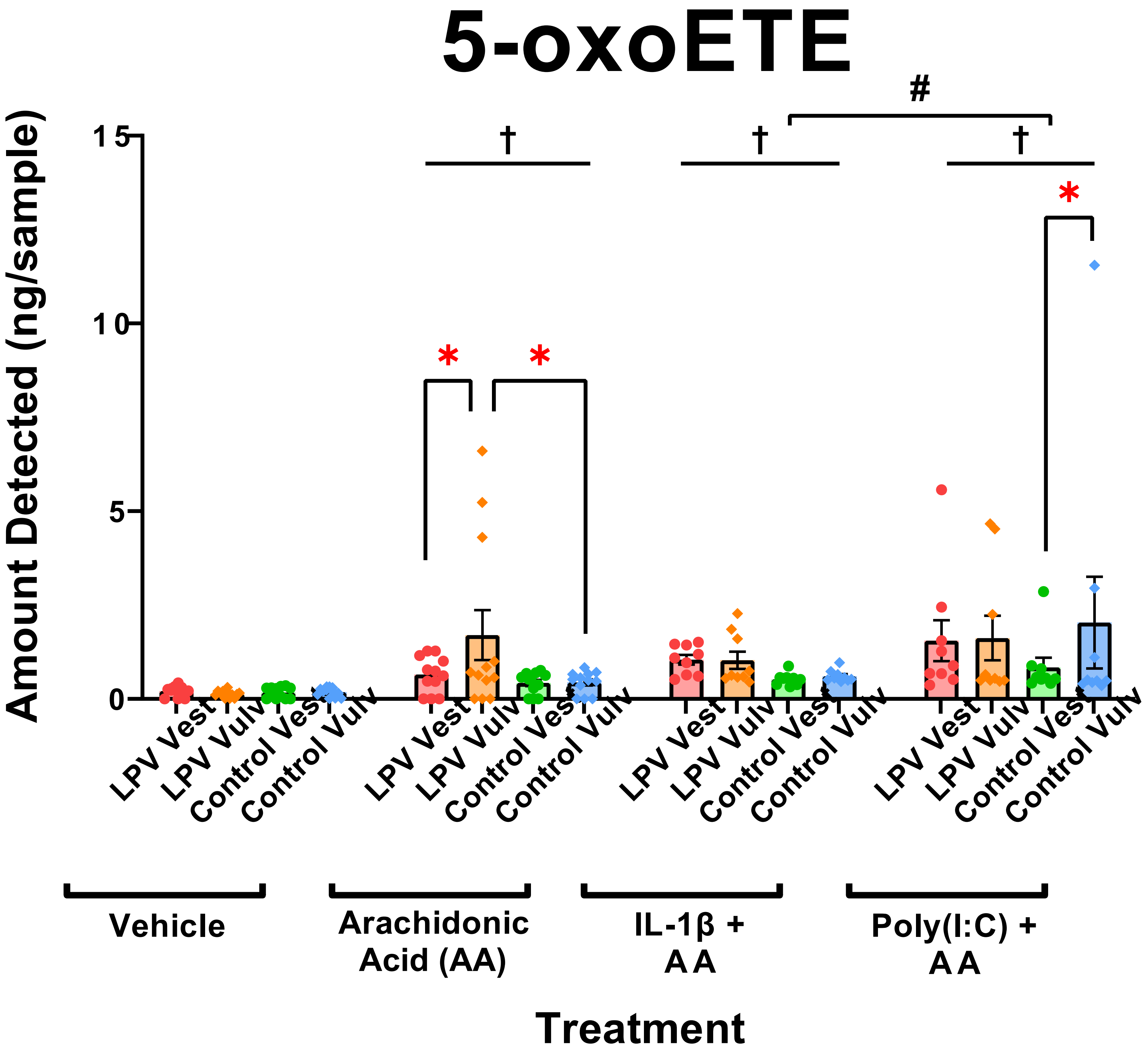

5-oxoETE
Amount Detected (ng/sample)
15
10
5
0
#
†
†
†
*
*
*
LPV Vest
LPV Vulv
Control Vest
Control Vulv
Vehicle
Arachidonic Acid (AA)
IL-1β + AA
Poly(I:C) + AA
Treatment

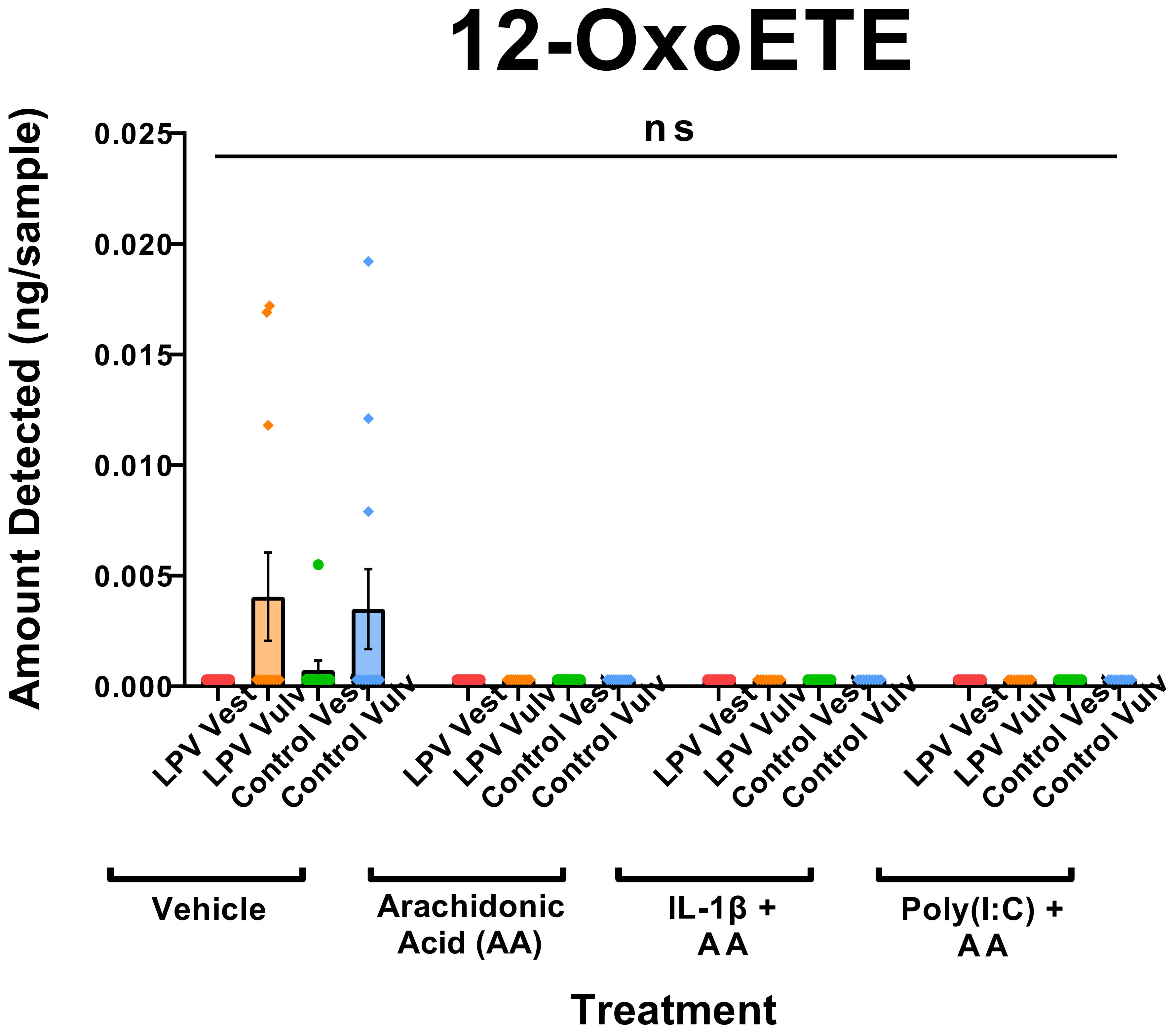
12-OxoETE
ns
Amount Detected (ng/sample)
0.025
0.020
0.015
0.010
0.005
0.000
LPV Vest
LPV Vulv
Control Vest
Control Vulv
Vehicle
Arachidonic Acid (AA)
IL-1β + AA
Poly(I:C) + AA
Treatment

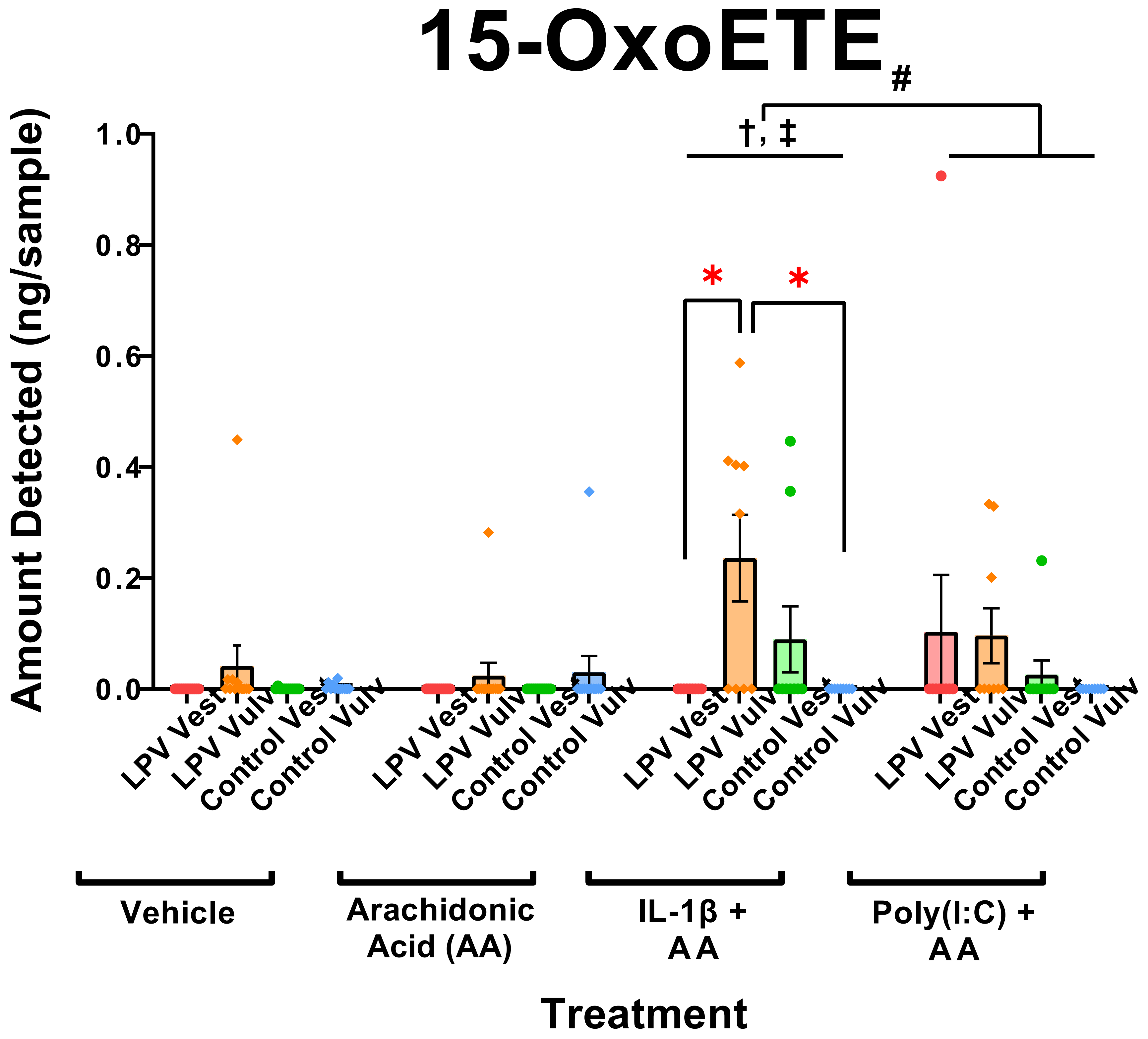

15-OxoETE#
Amount Detected (ng/sample)
1.0
0.8
0.6
0.4
0.2
0.0
†, ‡
*
*
LPV Vest
LPV Vulv
Control Vest
Control Vulv
Vehicle
Arachidonic Acid (AA)
IL-1β + AA
Poly(I:C) + AA
Treatment

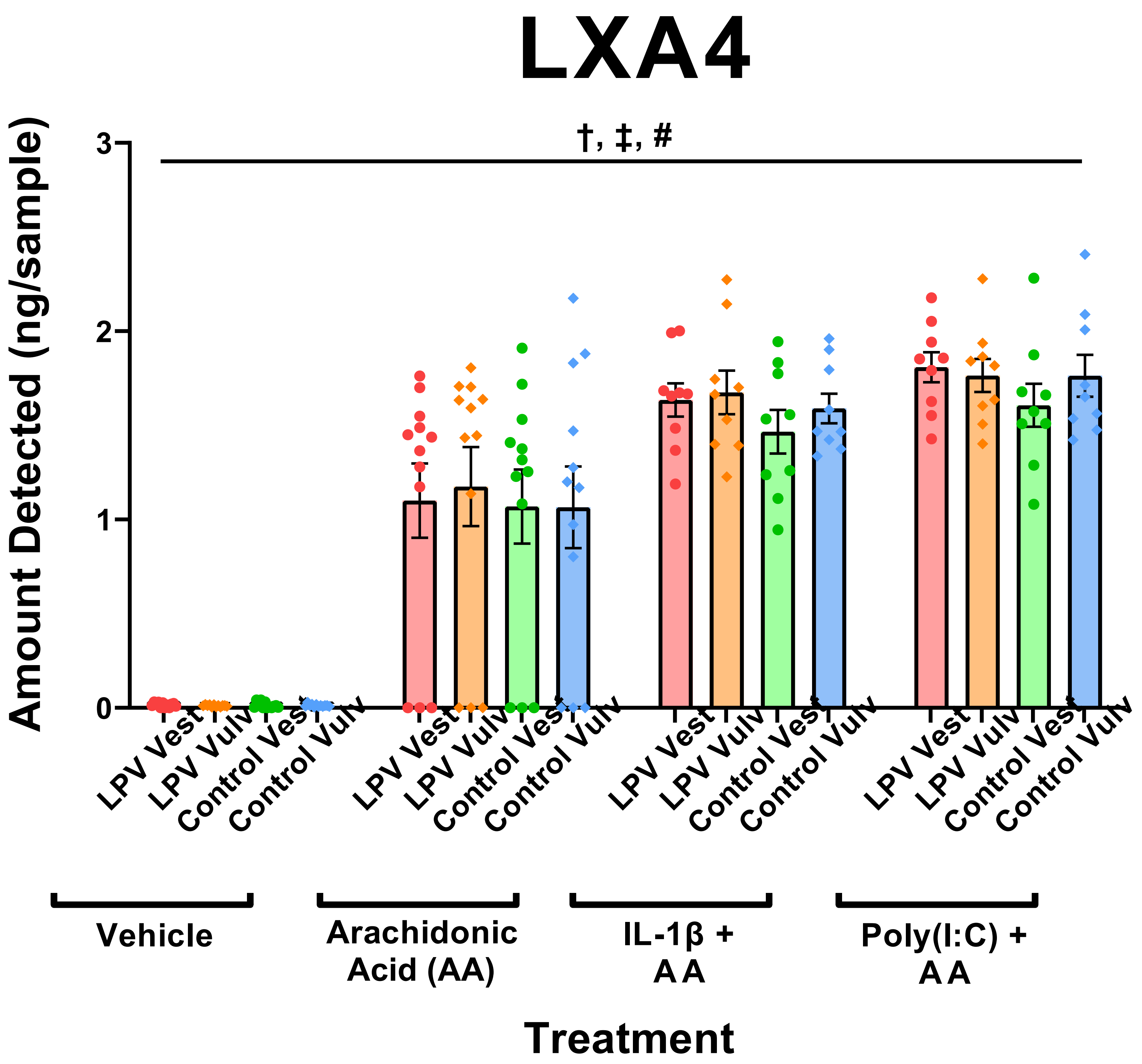

LXA4
†, ‡, #
Amount Detected (ng/sample)
0
1
2
3
LPV Vest
LPV Vulv
Control Vest
Control Vulv
Vehicle
Arachidonic Acid (AA)
IL-1β + AA
Poly(I:C) + AA
Treatment

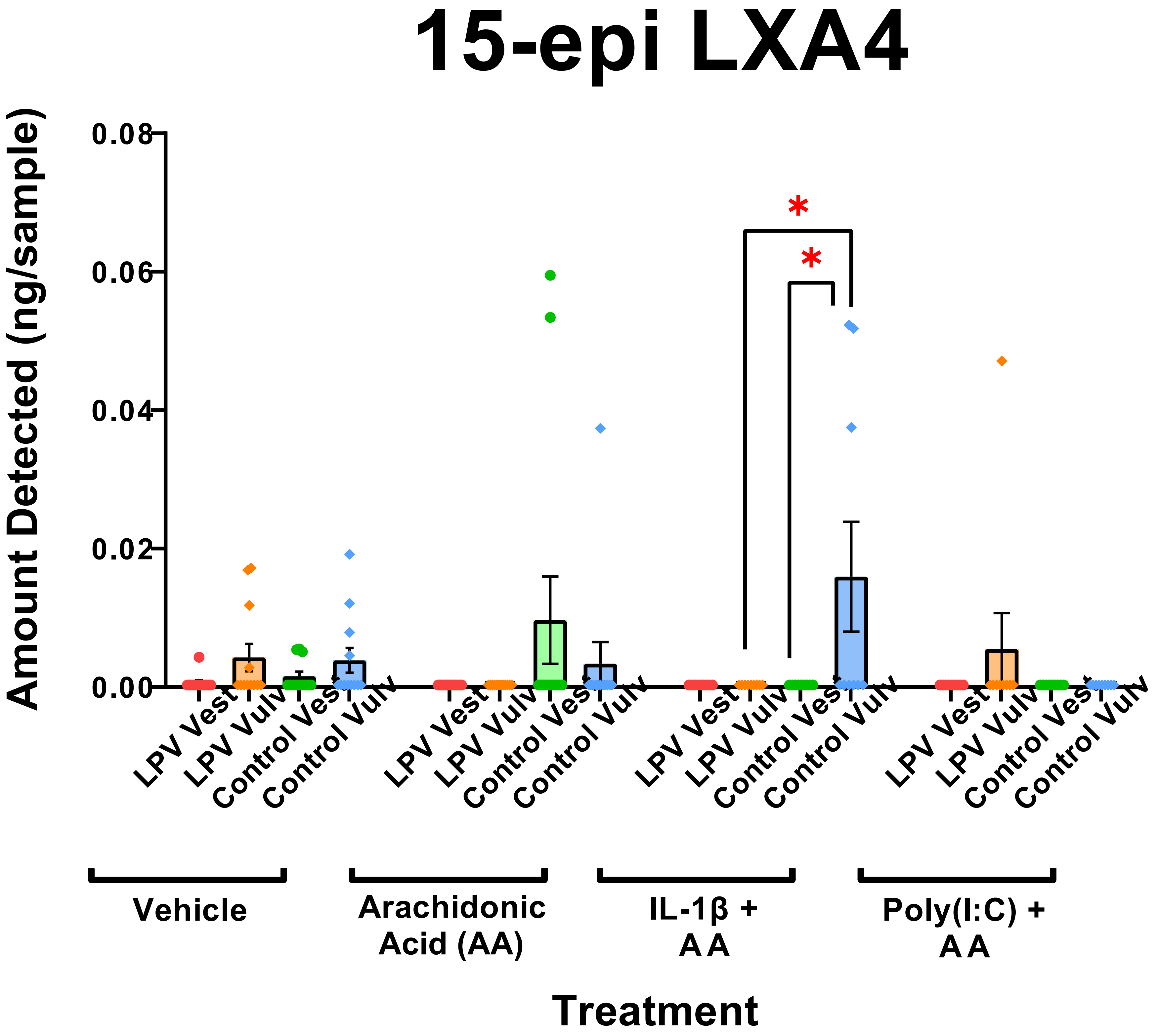

15-epi LXA4
Amount Detected (ng/sample)
0.08
0.06
0.04
0.02
0.00
*
*
LPV Vest
LPV Vulv
Control Vest
Control Vulv
Vehicle
Arachidonic Acid (AA)
IL-1β + AA
Poly(I:C) + AA
Treatment

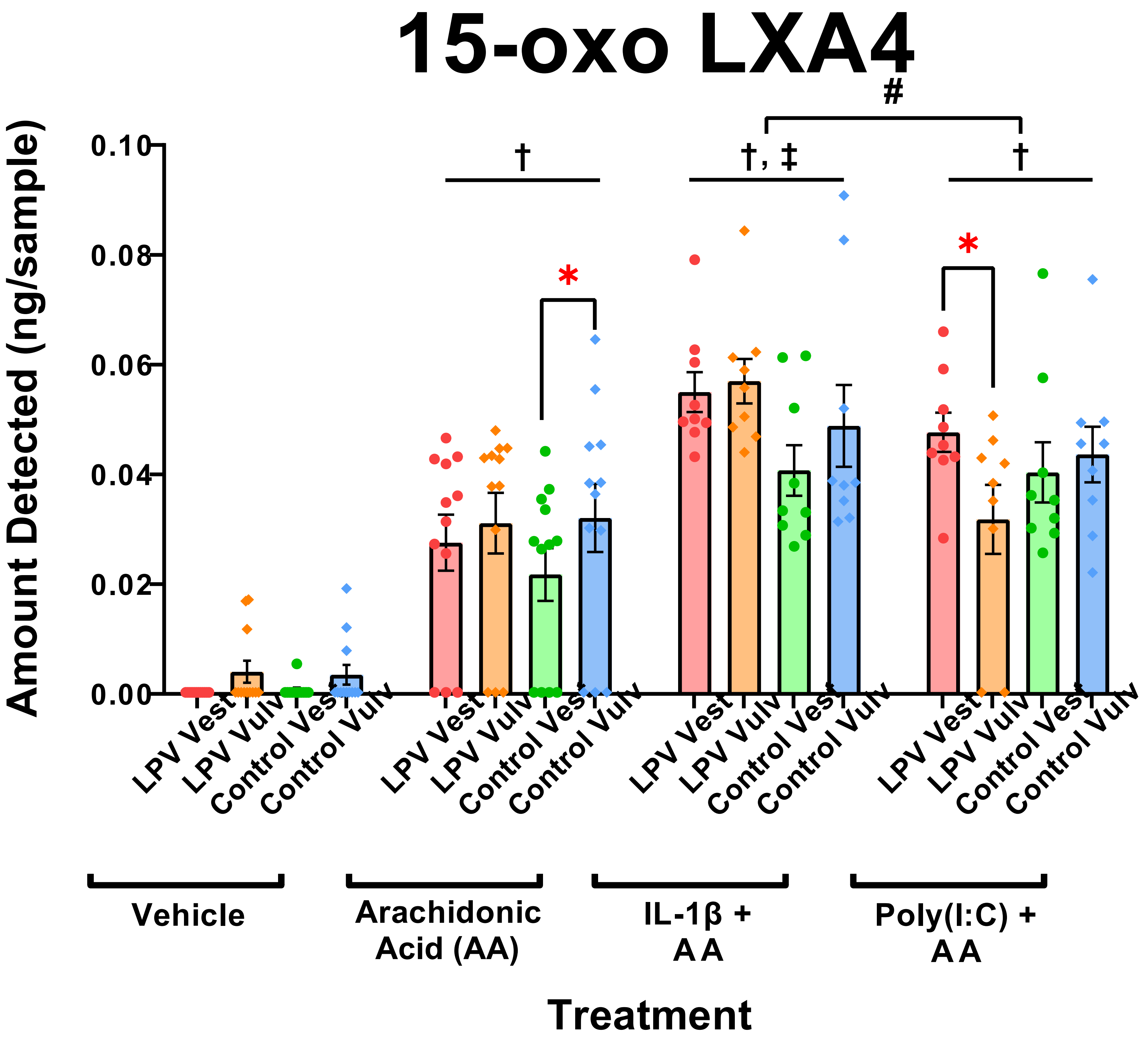

15-oxo LXA4
Amount Detected (ng/sample)
0.10
0.08
0.06
0.04
0.02
0.00
#
†
†, ‡
†
*
*
LPV Vest
LPV Vulv
Control Vest
Control Vulv
Vehicle
Arachidonic Acid (AA)
IL-1β + AA
Poly(I:C) + AA
Treatment

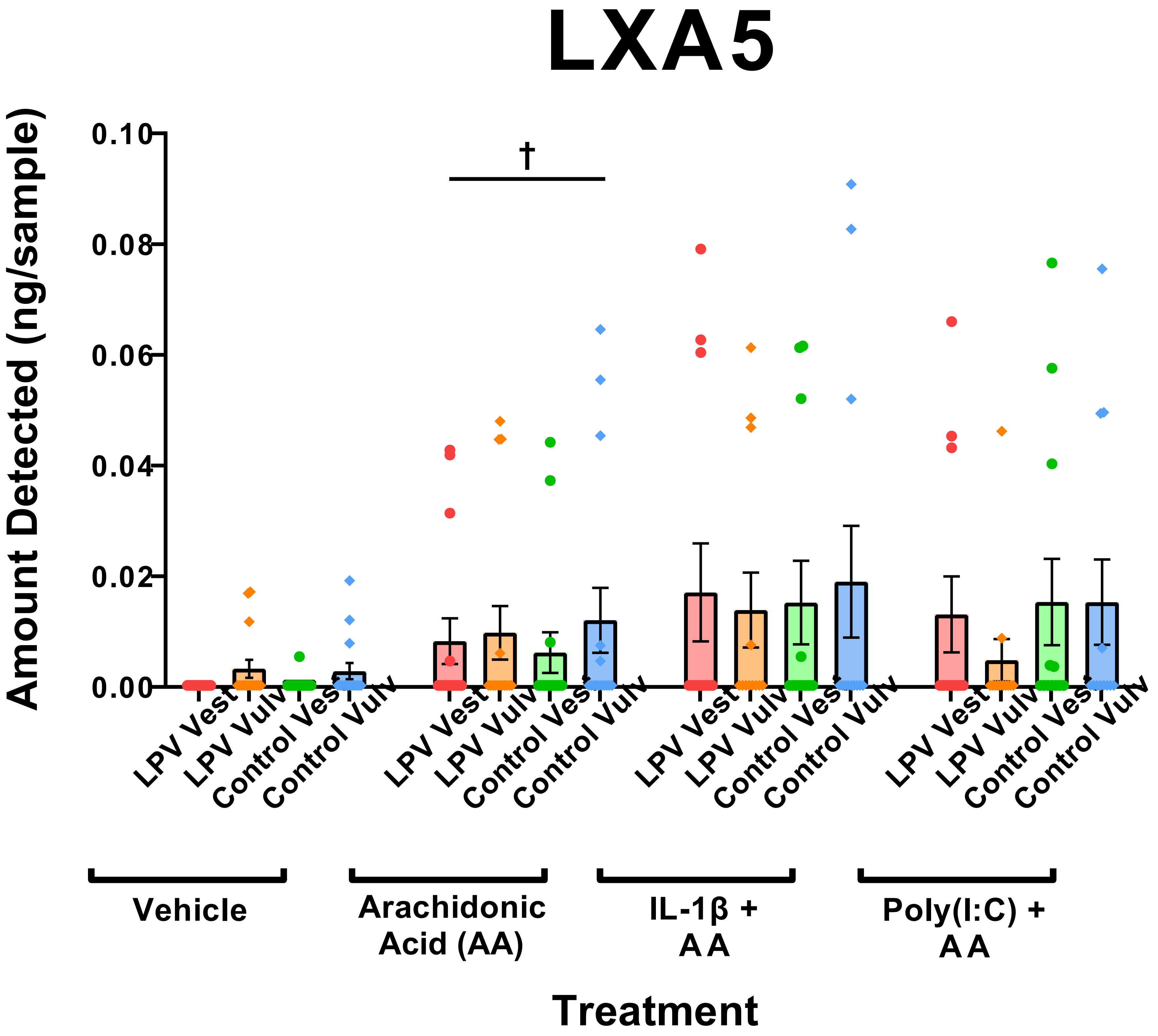

LXA5
Amount Detected (ng/sample)
0.10
0.08
0.06
0.04
0.02
0.00
†
LPV Vest
LPV Vulv
Control Vest
Control Vulv
Vehicle
Arachidonic Acid (AA)
IL-1β + AA
Poly(I:C) + AA
Treatment

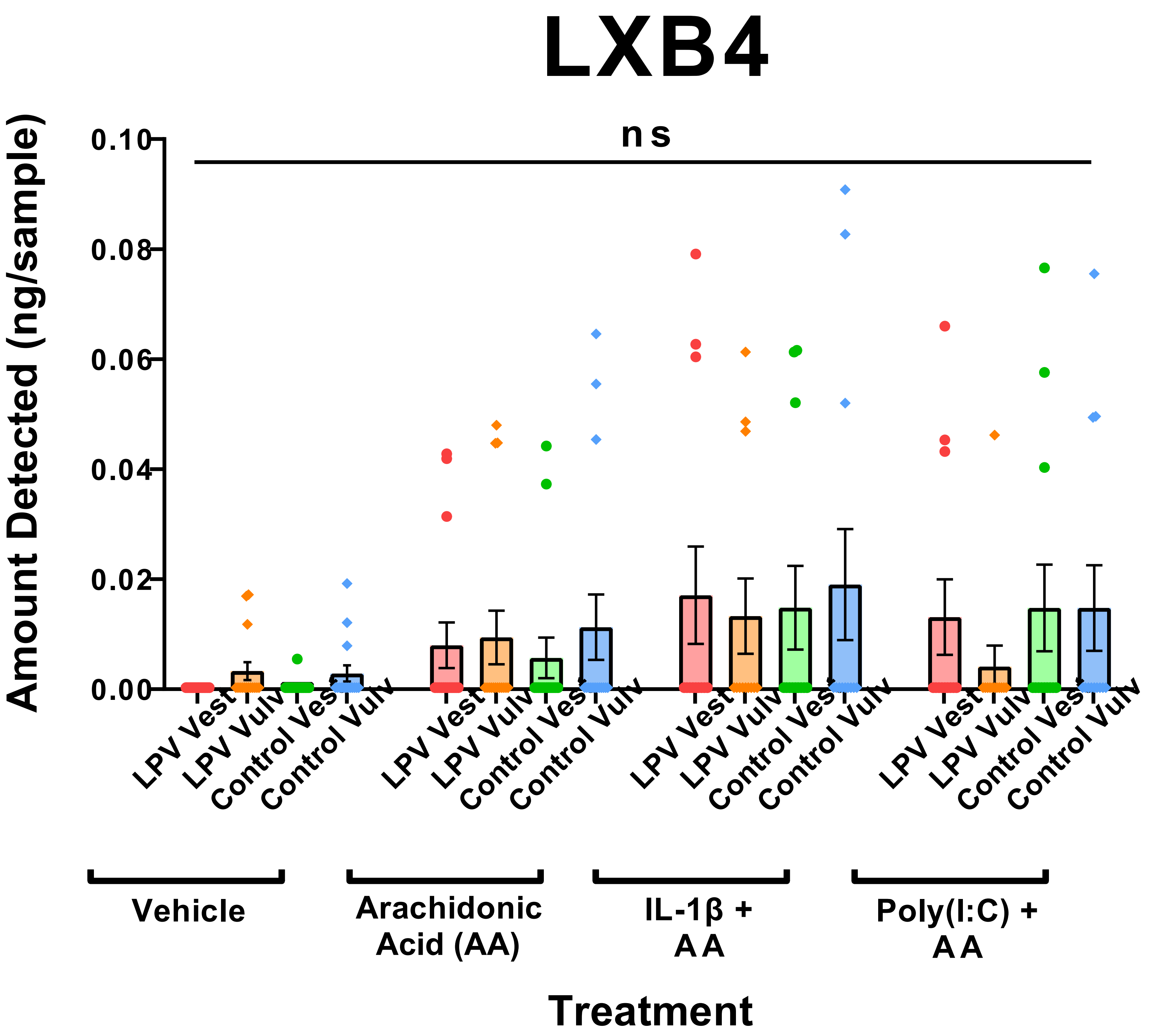
LXB4
ns
Amount Detected (ng/sample)
0.10
0.08
0.06
0.04
0.02
0.00
LPV Vest
LPV Vulv
Control Vest
Control Vulv
Vehicle
Arachidonic Acid (AA)
IL-1β + AA
Poly(I:C) + AA
Treatment

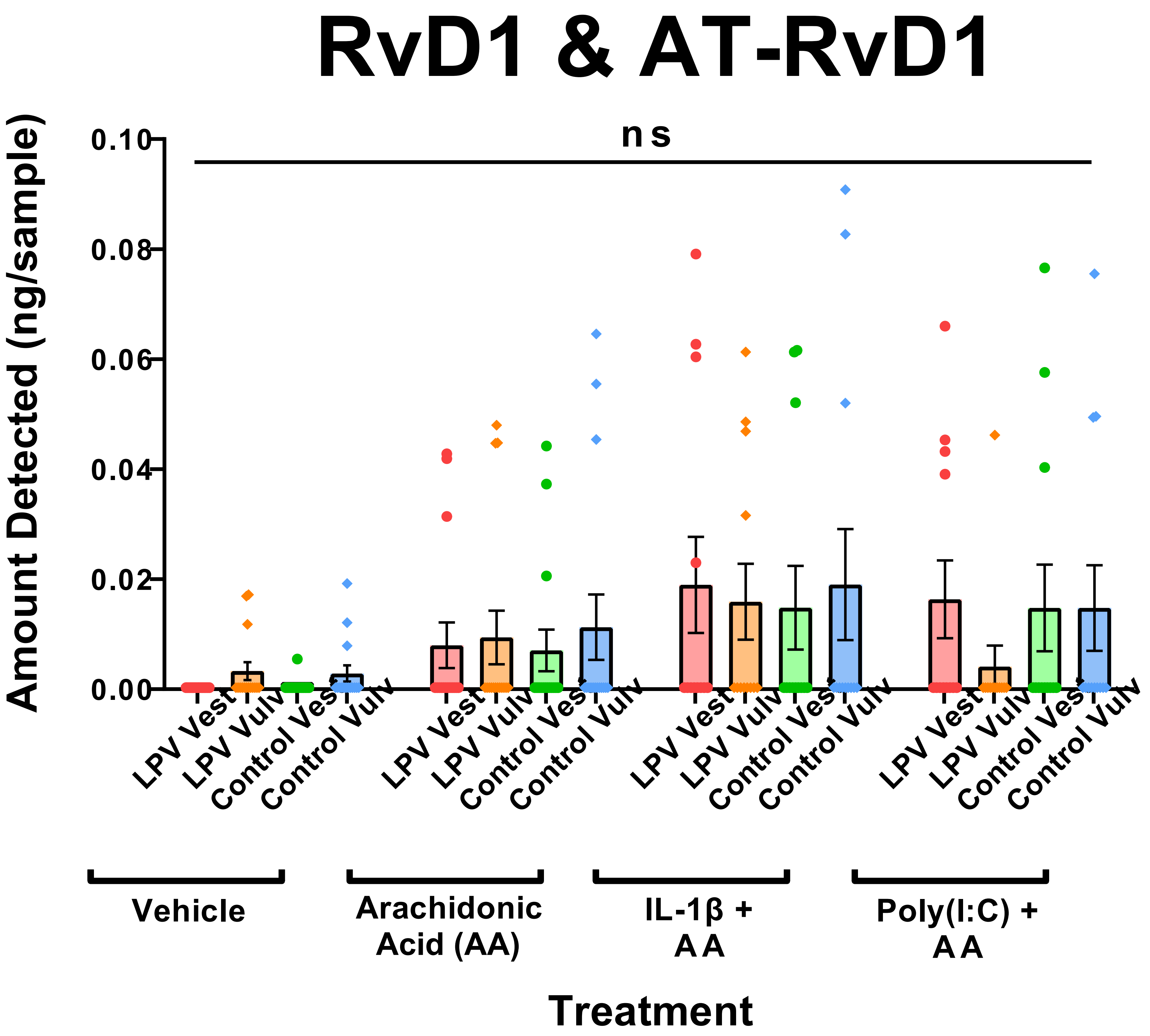

RvD1 & AT-RvD1
Amount Detected (ng/sample)
0.10
0.08
0.06
0.04
0.02
0.00
ns
LPV Vest
LPV Vulv
Control Vest
Control Vulv
Vehicle
Arachidonic Acid (AA)
IL-1β + AA
Poly(I:C) + AA
Treatment

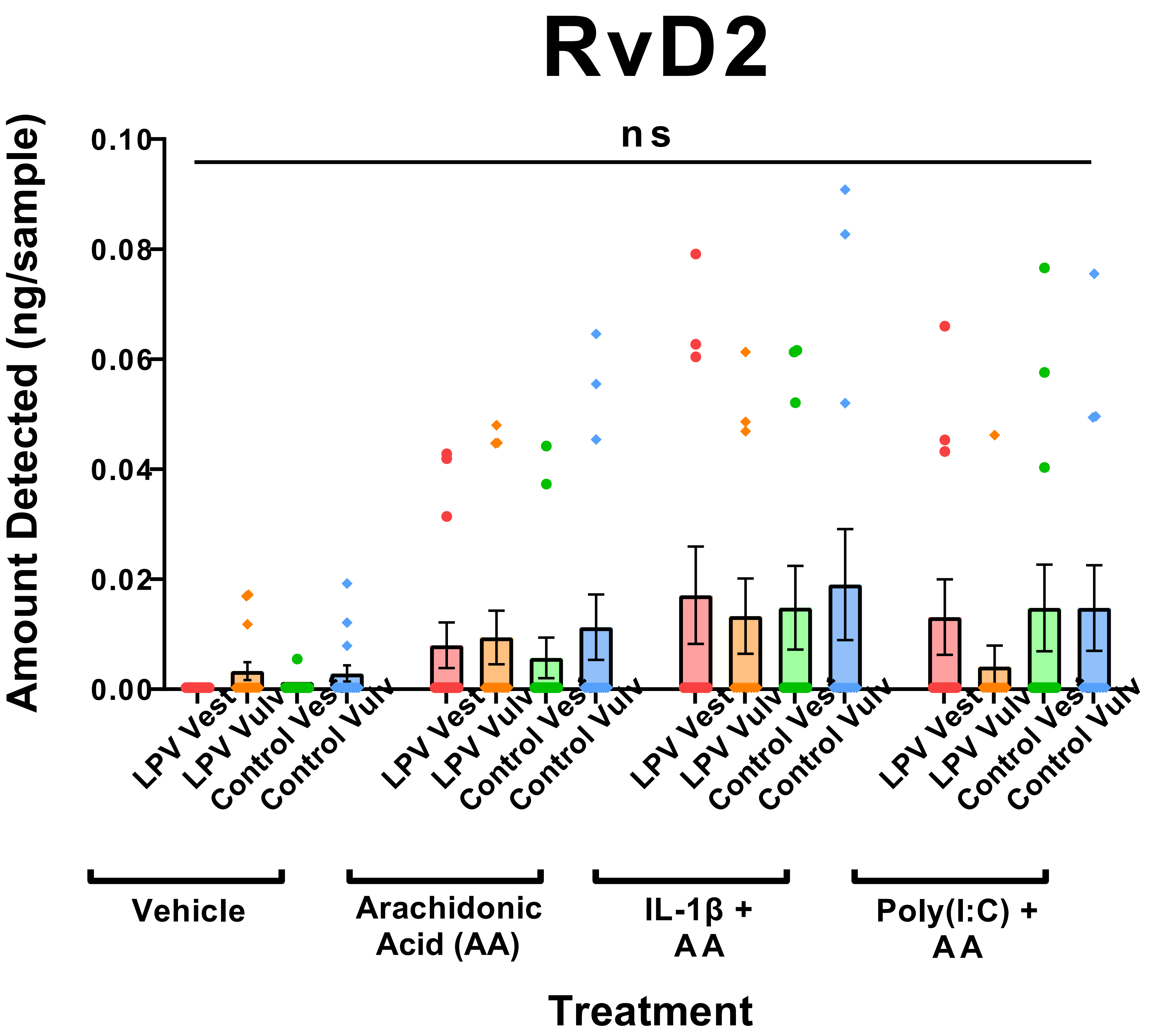
RvD2
ns
Amount Detected (ng/sample)
0.10
0.08
0.06
0.04
0.02
0.00
LPV Vest
LPV Vulv
Control Vest
Control Vulv
Vehicle
Arachidonic Acid (AA)
IL-1β + AA
Poly(I:C) + AA
Treatment

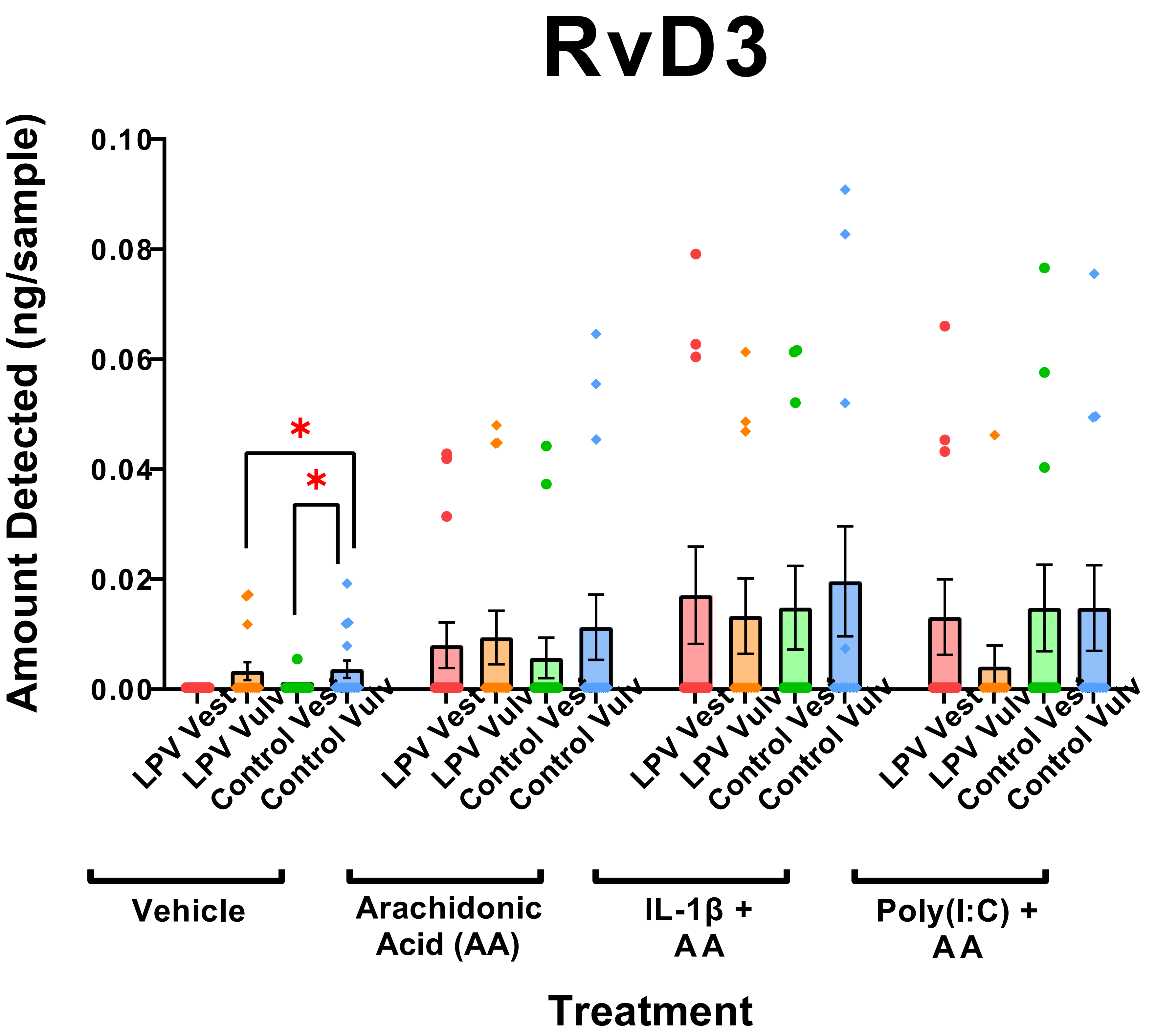
RvD3
Amount Detected (ng/sample)
0.10
0.08
0.06
0.04
0.02
0.00
*
*
LPV Vest
LPV Vulv
Control Vest
Control Vulv
Vehicle
Arachidonic Acid (AA)
IL-1β + AA
Poly(I:C) + AA
Treatment

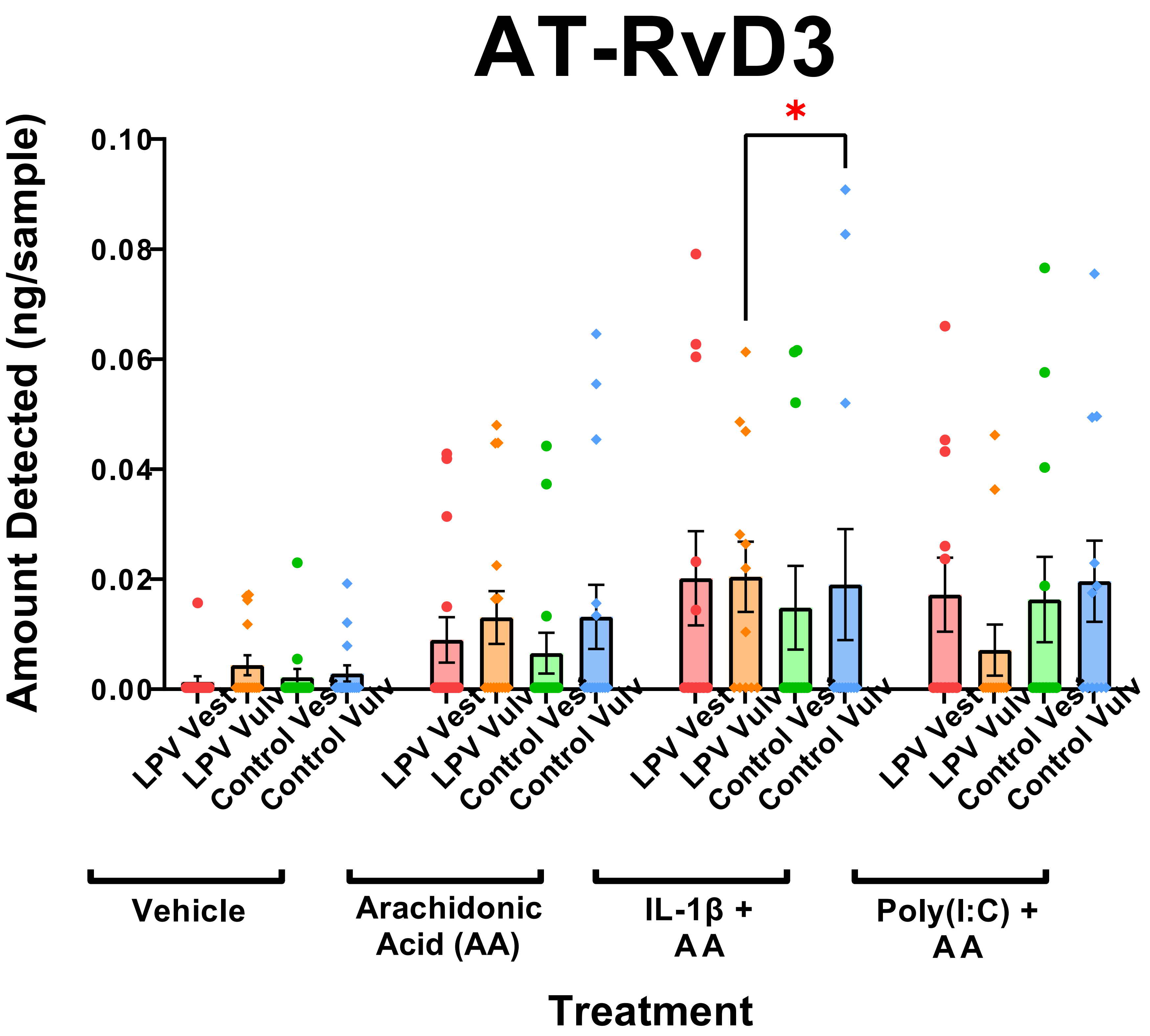
AT-RvD3
Amount Detected (ng/sample)
0.10
0.08
0.06
0.04
0.02
0.00
*
LPV Vest
LPV Vulv
Control Vest
Control Vulv
Vehicle
Arachidonic Acid (AA)
IL-1β + AA
Poly(I:C) + AA
Treatment

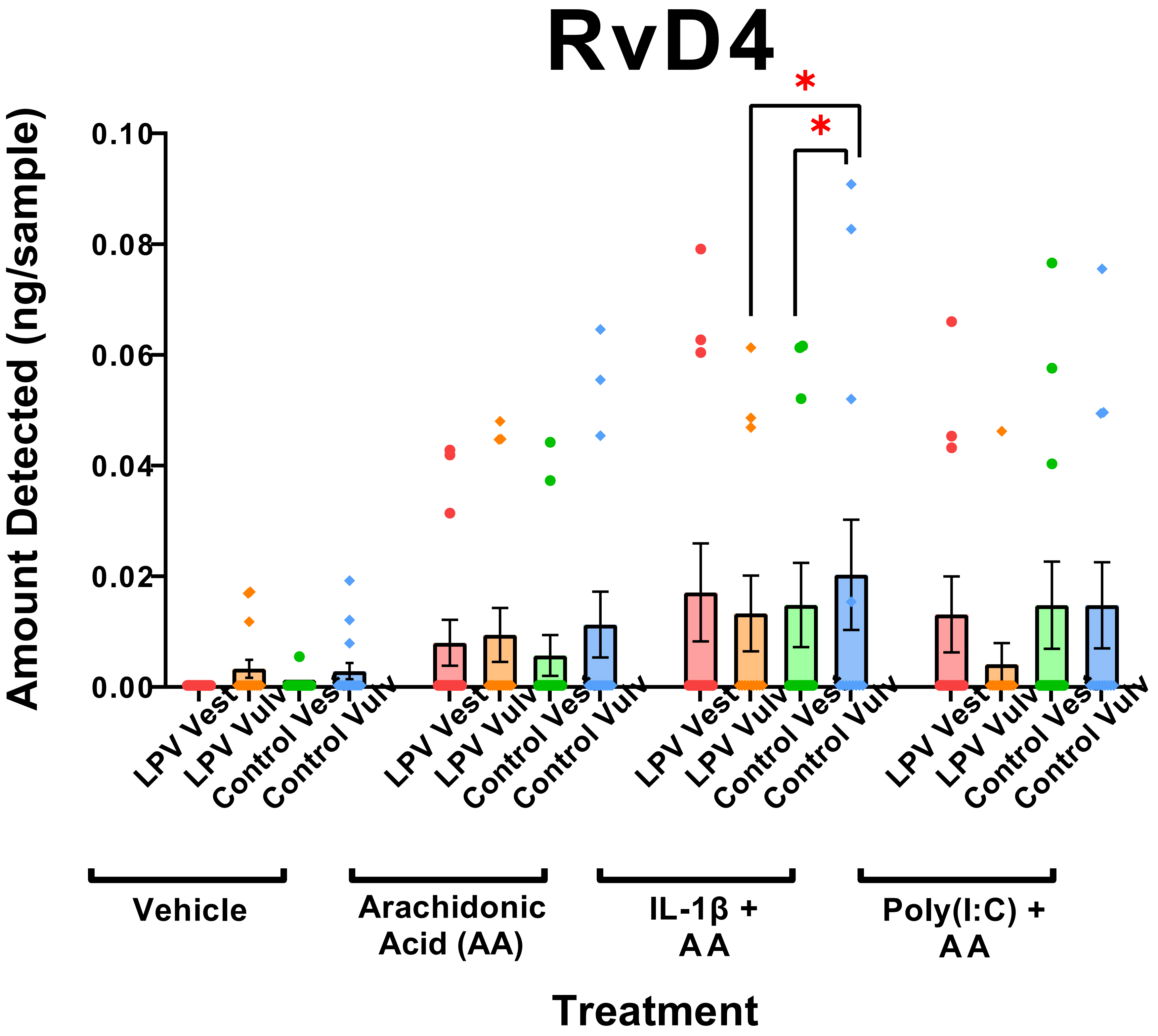

RvD4
*
*
Amount Detected (ng/sample)
0.10
0.08
0.06
0.04
0.02
0.00
LPV Vest
LPV Vulv
Control Vest
Control Vulv
Vehicle
Arachidonic Acid (AA)
IL-1β + AA
Poly(I:C) + AA
Treatment

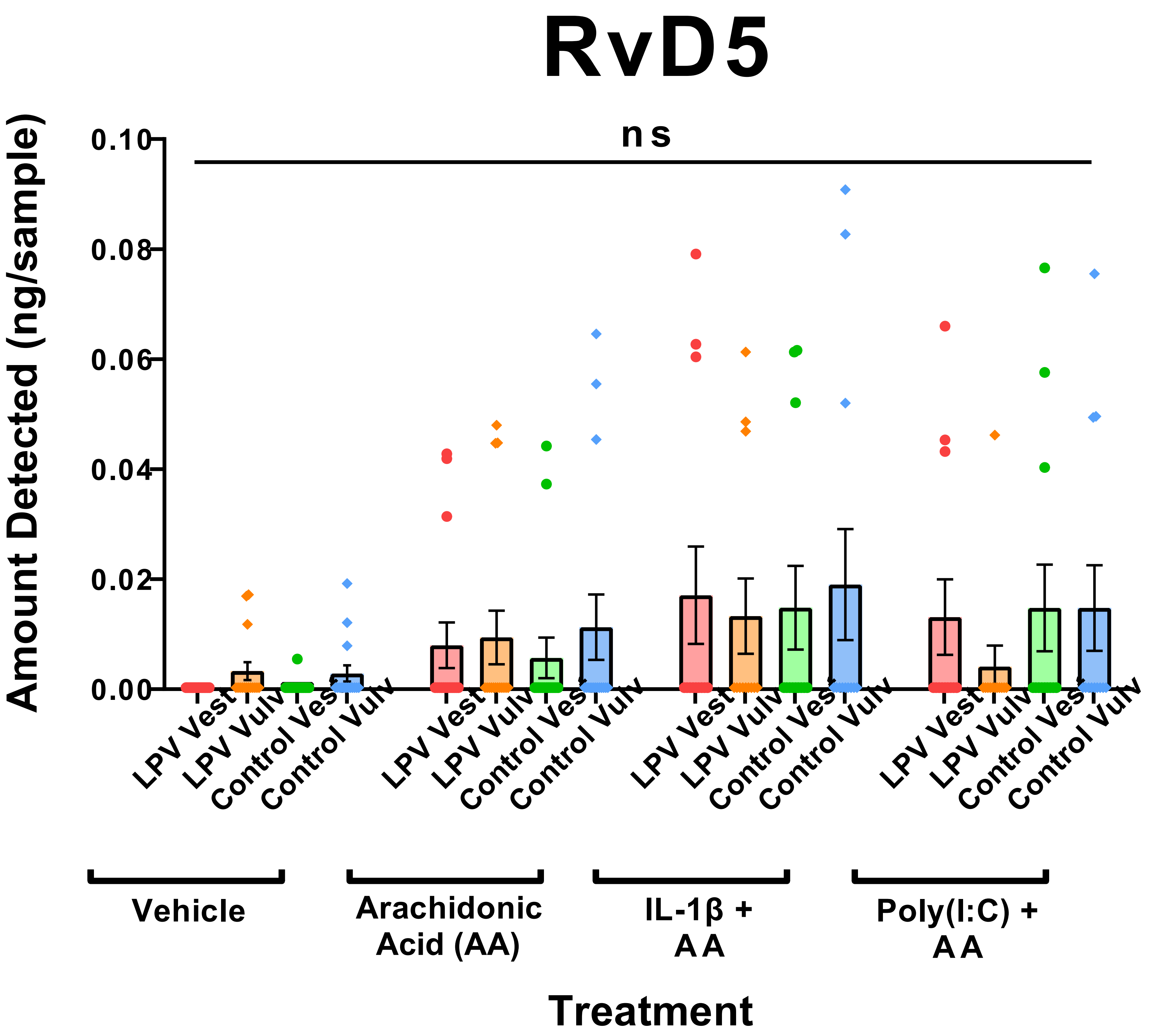

RvD5
Amount Detected (ng/sample)
0.10
0.08
0.06
0.04
0.02
0.00
ns
LPV Vest
LPV Vulv
Control Vest
Control Vulv
Vehicle
Arachidonic Acid (AA)
IL-1β + AA
Poly(I:C) + AA
Treatment

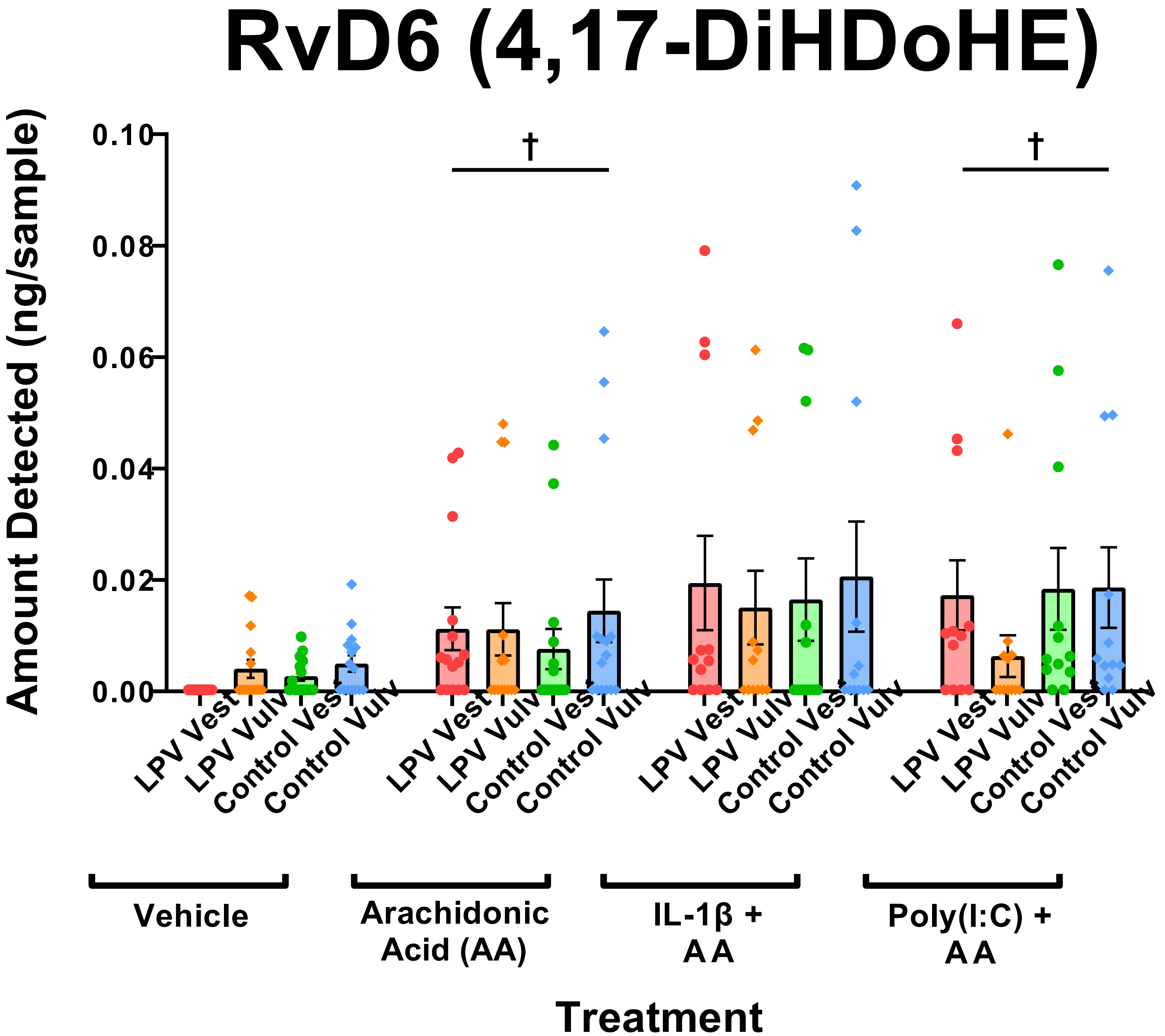
RvD6 (4,17-DiHDoHE)
Amount Detected (ng/sample)
0.10
0.08
0.06
0.04
0.02
0.00
†
†
LPV Vest
LPV Vulv
Control Vest
Control Vulv
Vehicle
Arachidonic Acid (AA)
IL-1β + AA
Poly(I:C) + AA
Treatment

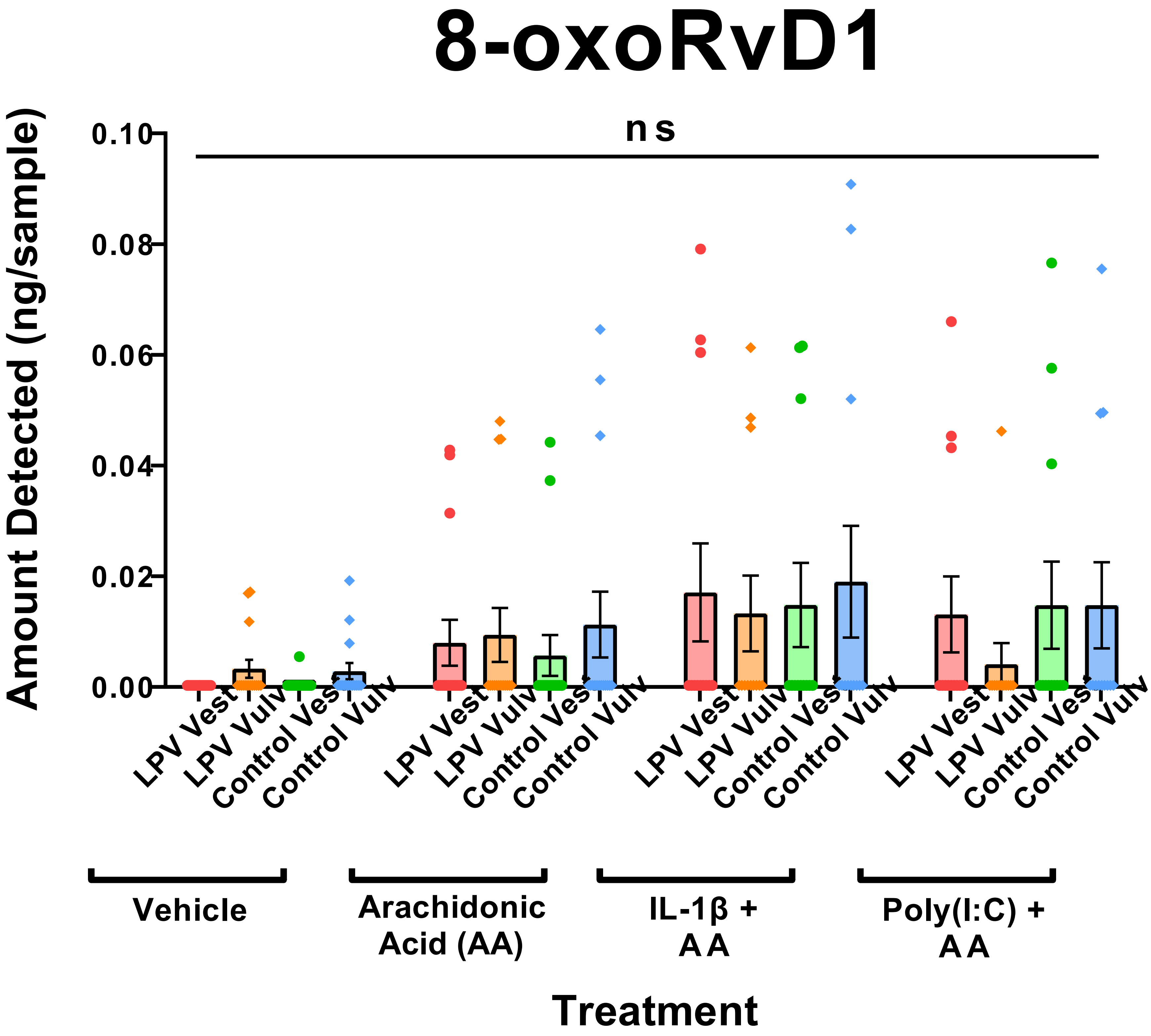

8-oxoRvD1
ns
Amount Detected (ng/sample)
0.10
0.08
0.06
0.04
0.02
0.00
LPV Vest
LPV Vulv
Control Vest
Control Vulv
Vehicle
Arachidonic Acid (AA)
IL-1β + AA
Poly(I:C) + AA
Treatment

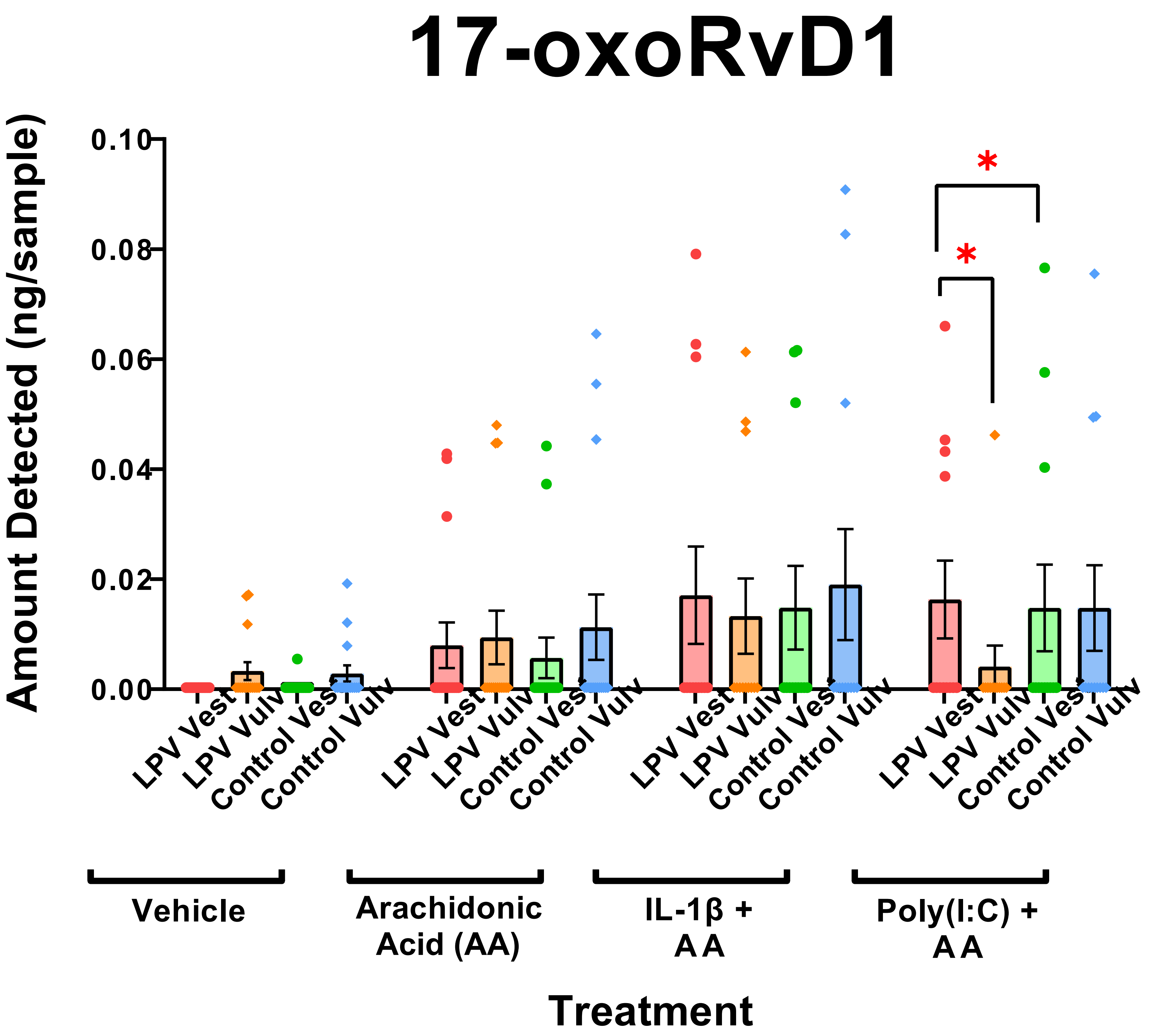

17-oxoRvD1
Amount Detected (ng/sample)
0.10
0.08
0.06
0.04
0.02
0.00
*
*
LPV Vest
LPV Vulv
Control Vest
Control Vulv
Vehicle
Arachidonic Acid (AA)
IL-1β + AA
Poly(I:C) + AA
Treatment

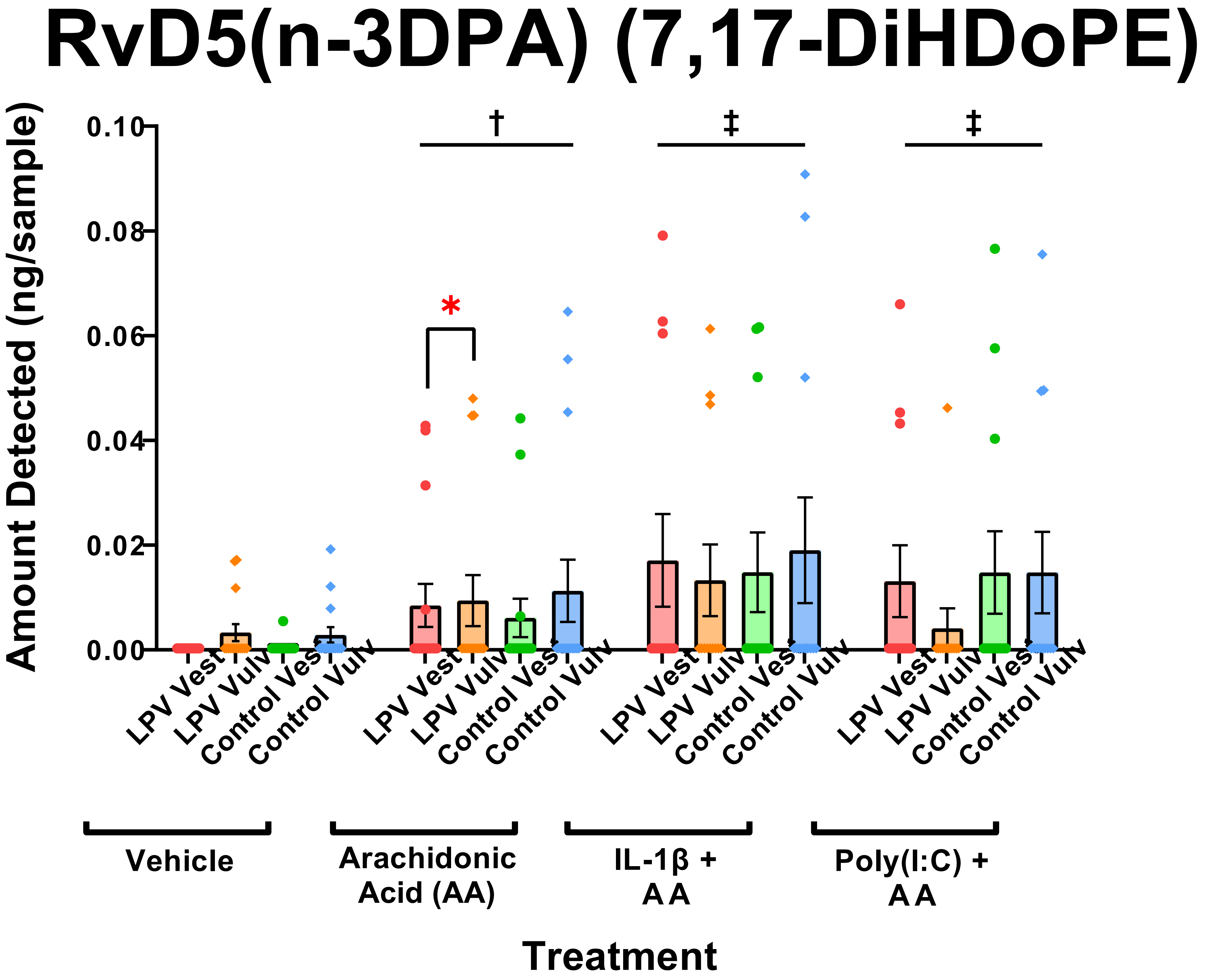

RvD5(n-3DPA) (7,17-DiHDoPE)
Amount Detected (ng/sample)
0.10
0.08
0.06
0.04
0.02
0.00
†
‡
‡
*
LPV Vest
LPV Vulv
Control Vest
Control Vulv
Vehicle
Arachidonic Acid (AA)
IL-1β + AA
Poly(I:C) + AA
Treatment

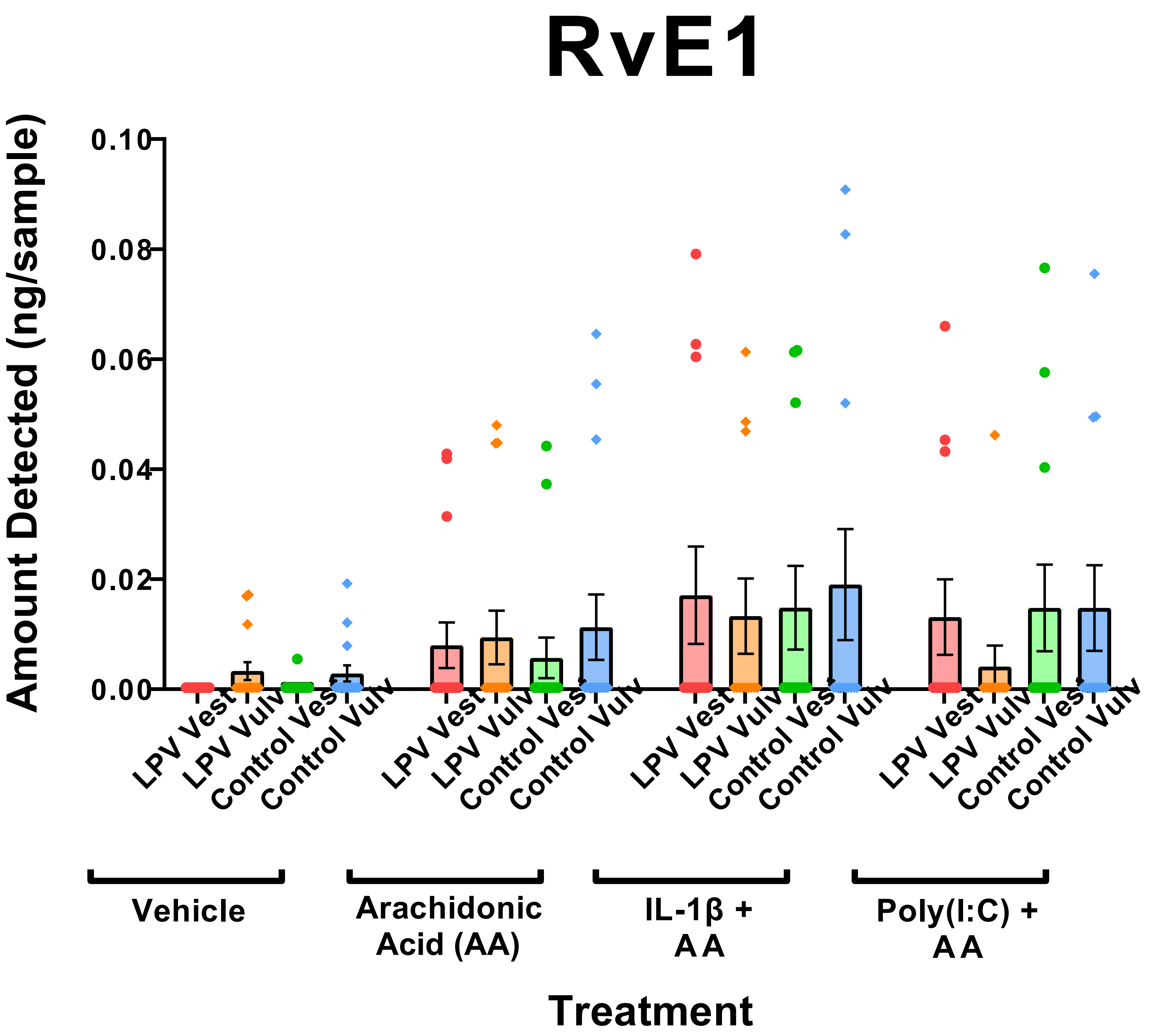

RvE1
Amount Detected (ng/sample)
0.10
0.08
0.06
0.04
0.02
0.00
LPV Vest
LPV Vulv
Control Vest
Control Vulv
LPV Vest
LPV Vulv
Control Vest
Control Vulv
LPV Vest
LPV Vulv
Control Vest
Control Vulv
LPV Vest
LPV Vulv
Control Vest
Control Vulv
Vehicle
Arachidonic Acid (AA)
IL-1β + AA
Poly(I:C) + AA
Treatment

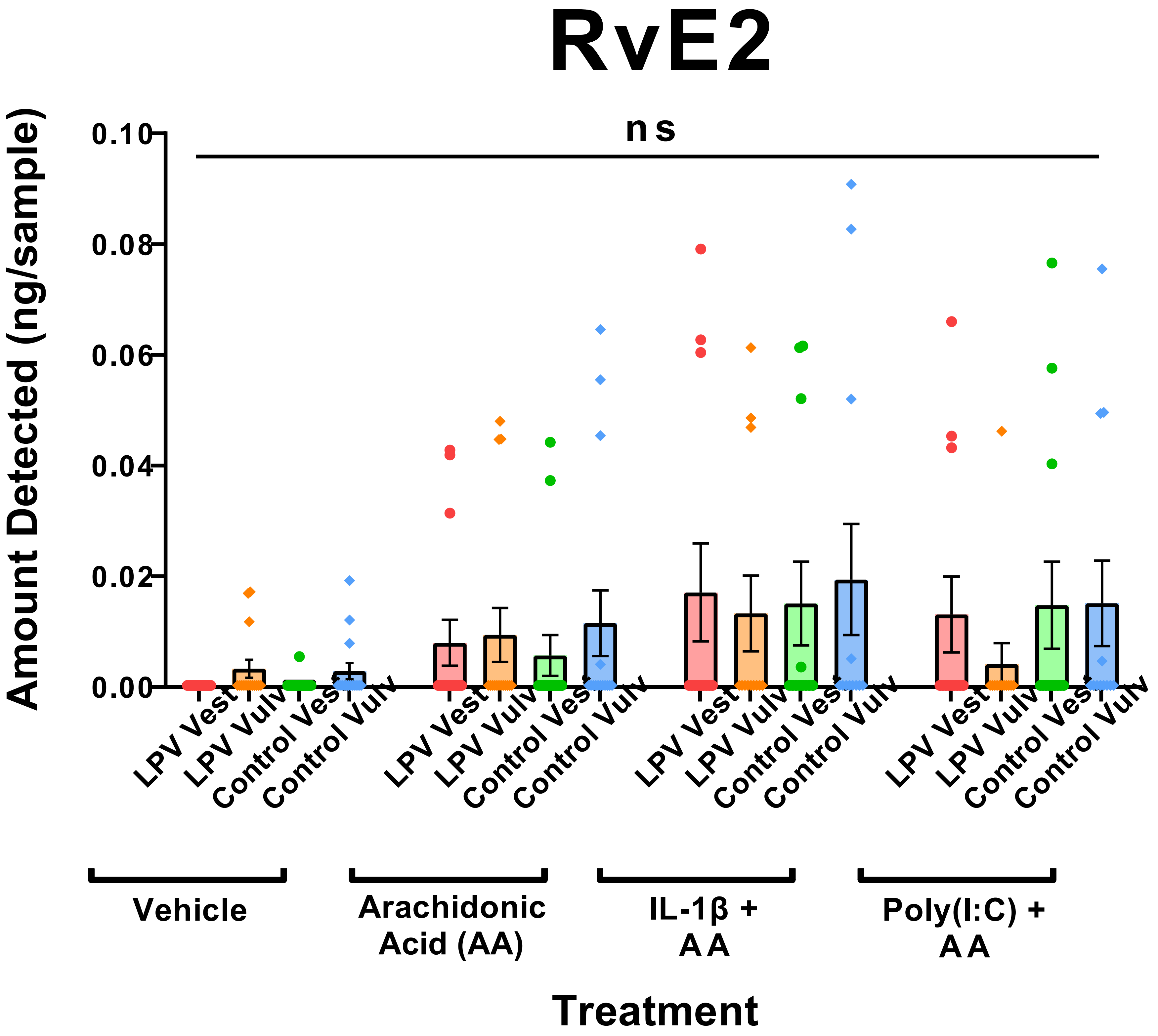

RvE2
ns
Amount Detected (ng/sample)
0.10
0.08
0.06
0.04
0.02
0.00
LPV Vest
LPV Vulv
Control Vest
Control Vulv
Vehicle
Arachidonic Acid (AA)
IL-1β + AA
Poly(I:C) + AA
Treatment

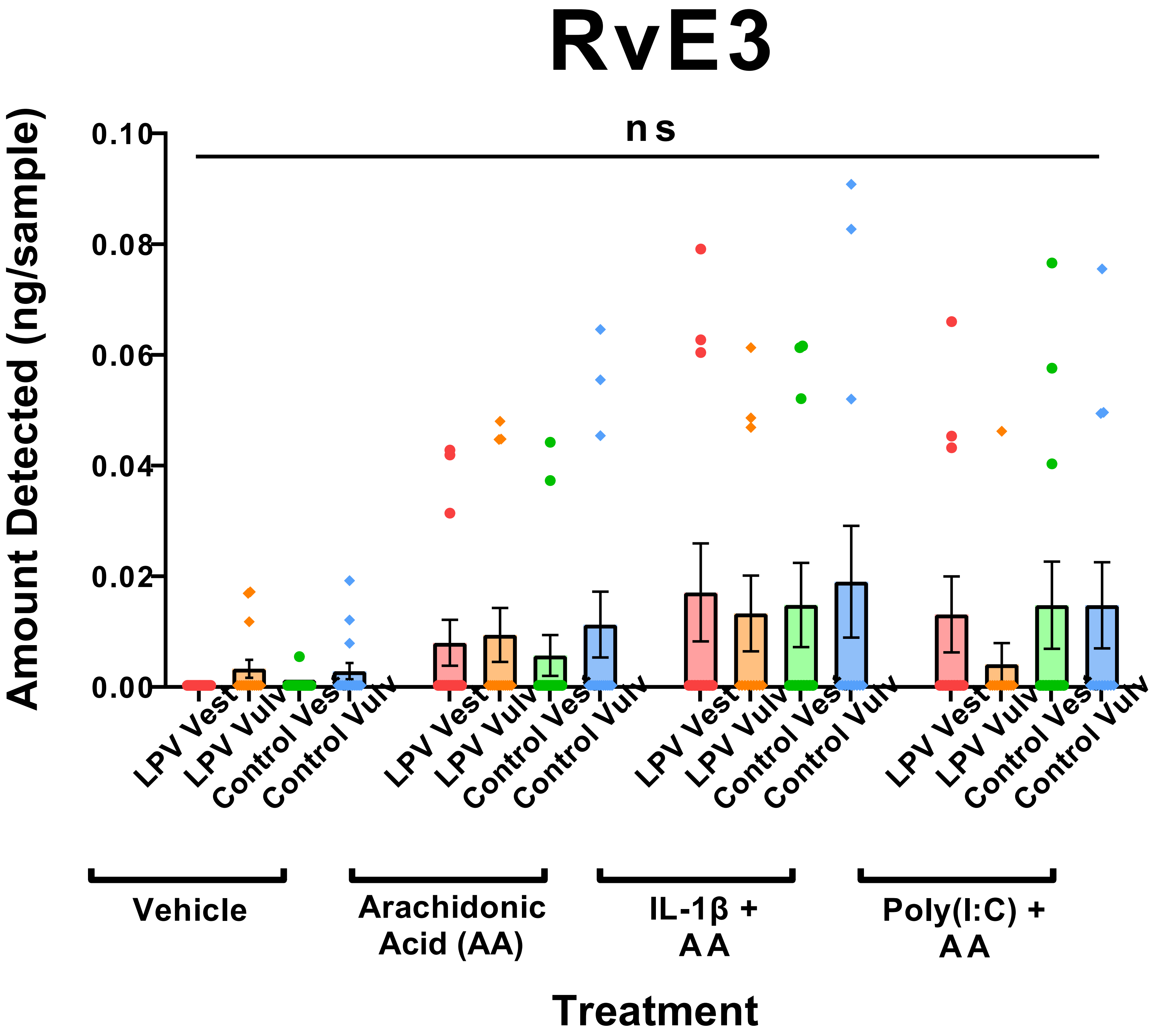

RvE3
ns
Amount Detected (ng/sample)
0.10
0.08
0.06
0.04
0.02
0.00
LPV Vest
LPV Vulv
Control Vest
Control Vulv
Vehicle
Arachidonic Acid (AA)
IL-1β + AA
Poly(I:C) + AA
Treatment

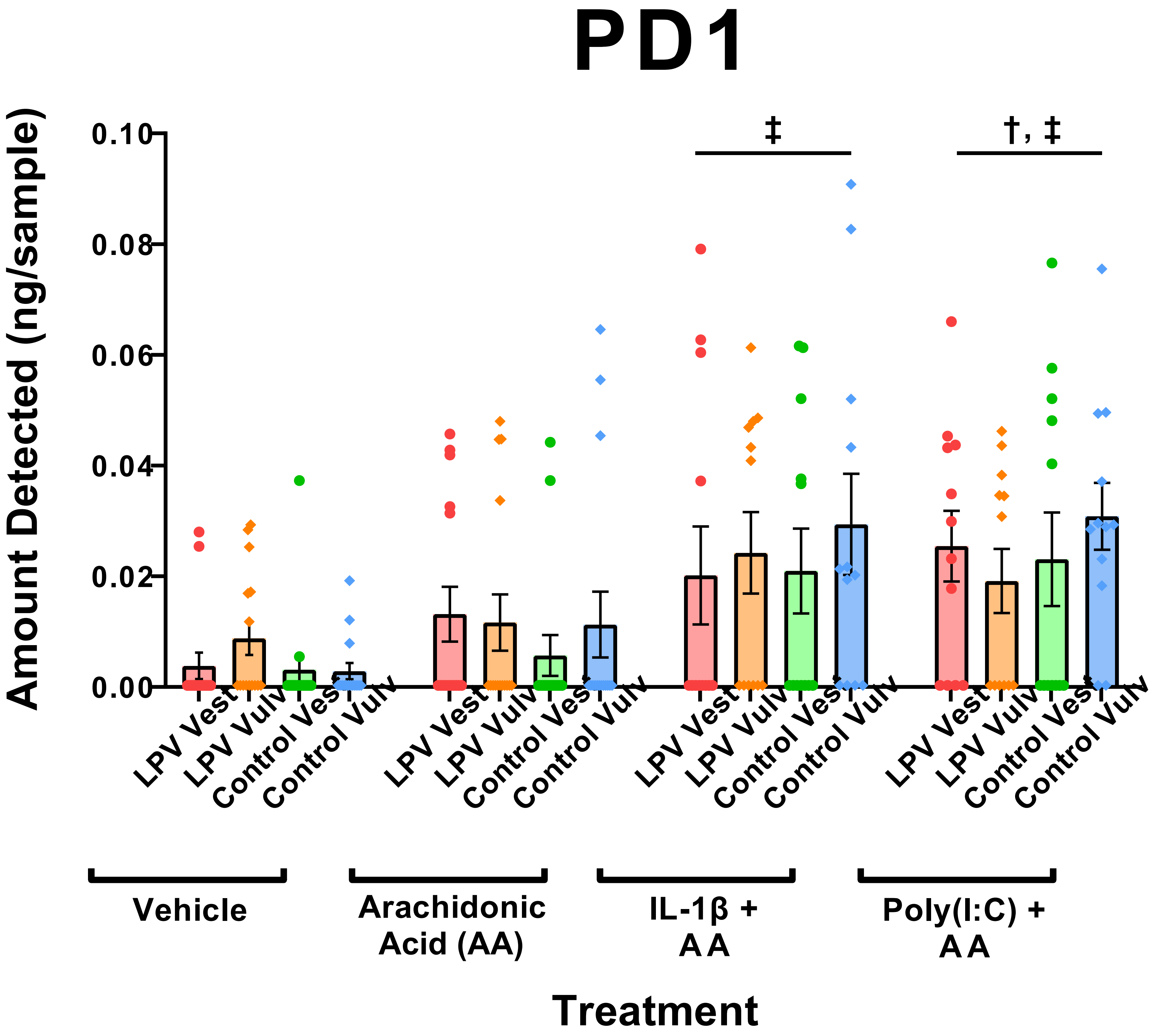
PD1
Amount Detected (ng/sample)
0.10
0.08
0.06
0.04
0.02
0.00
‡
†, ‡
LPV Vest
LPV Vulv
Control Vest
Control Vulv
Vehicle
Arachidonic Acid (AA)
IL-1β + AA
Poly(I:C) + AA
Treatment

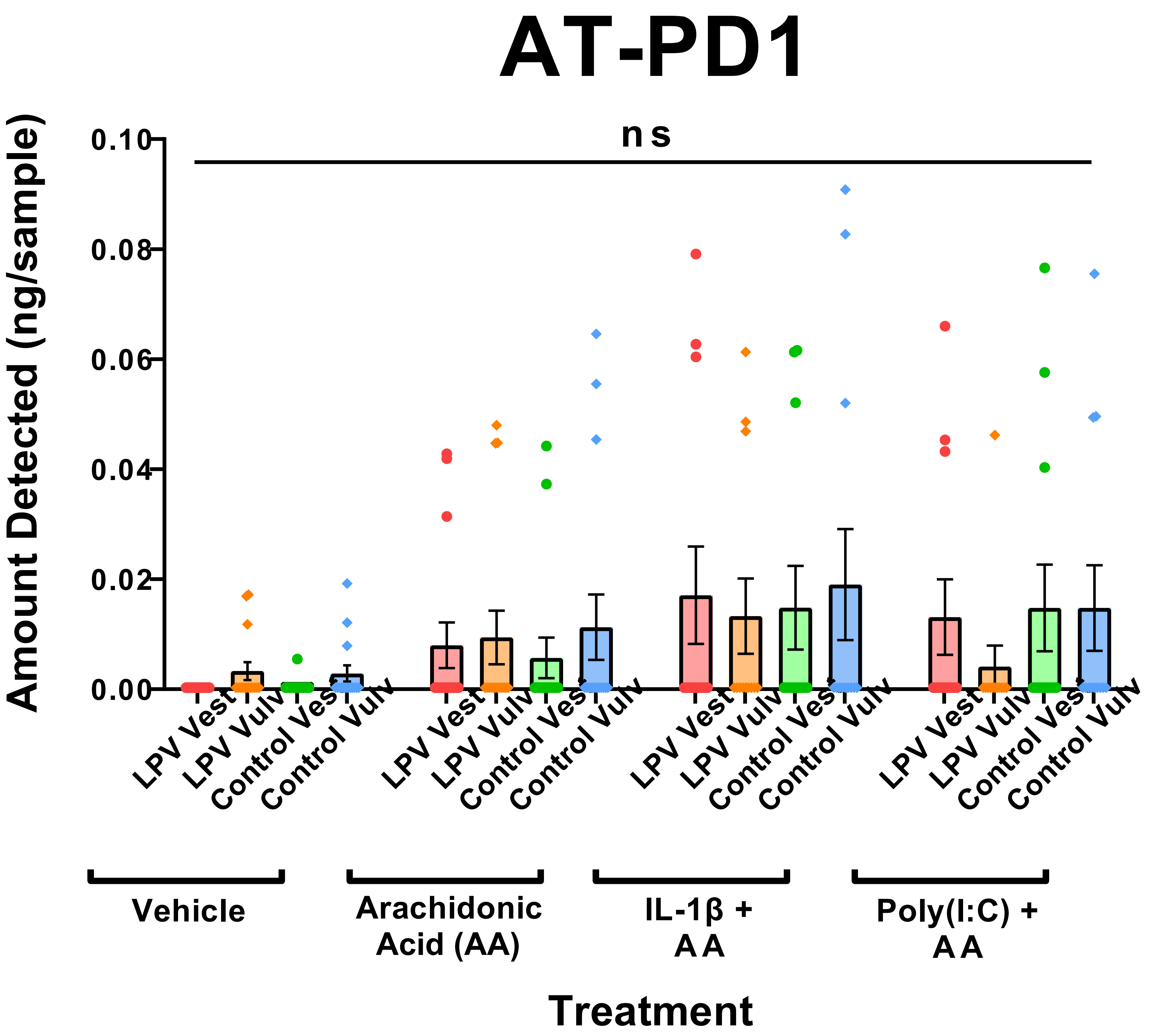
AT-PD1
ns
Amount Detected (ng/sample)
0.10
0.08
0.06
0.04
0.02
0.00
LPV Vest
LPV Vulv
Control Vest
Control Vulv
Vehicle
Arachidonic Acid (AA)
IL-1β + AA
Poly(I:C) + AA
Treatment

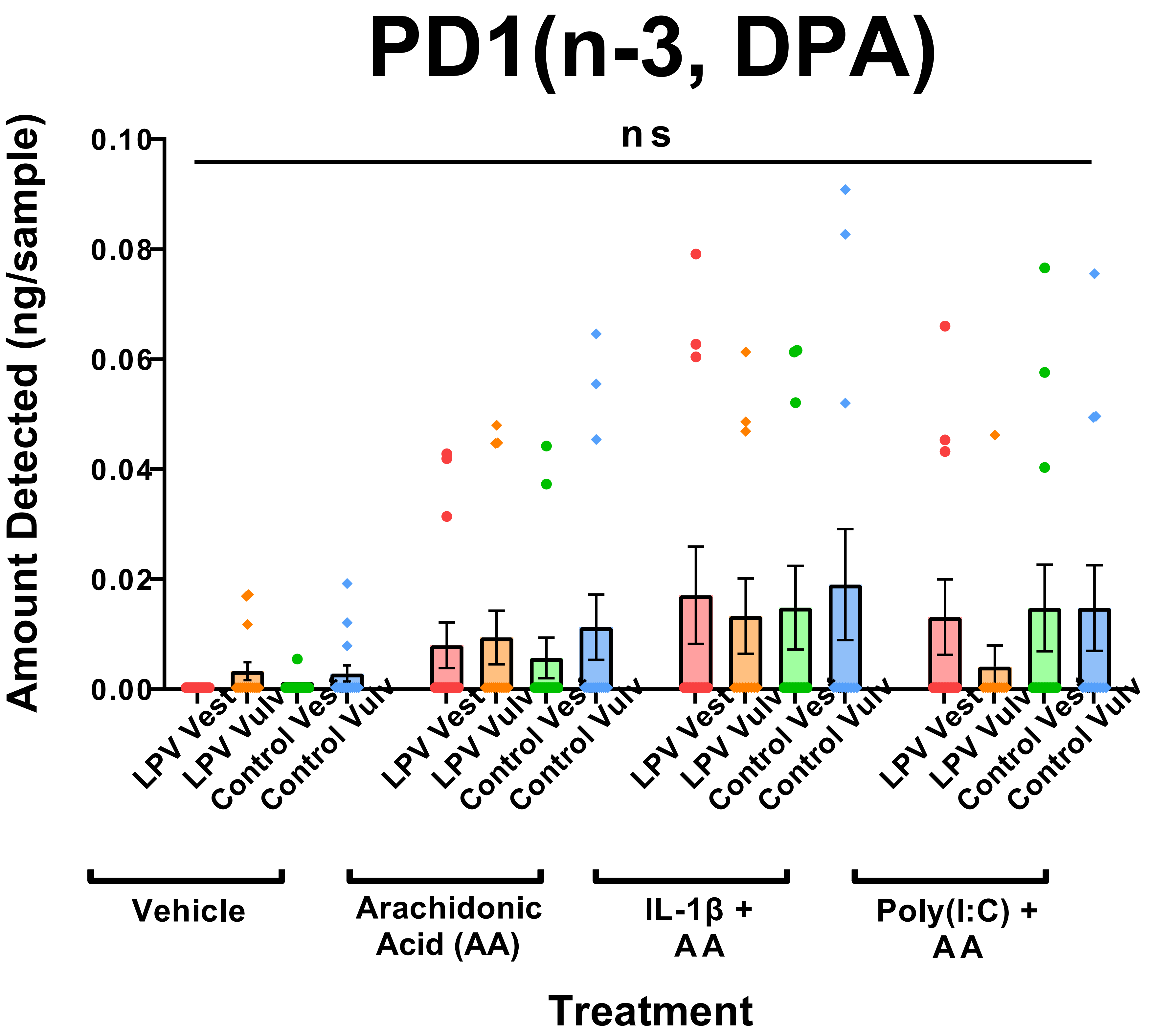

PD1(n-3, DPA)
Amount Detected (ng/sample)
0.10
0.08
0.06
0.04
0.02
0.00
ns
LPV Vest
LPV Vulv
Control Vest
Control Vulv
Vehicle
Arachidonic Acid (AA)
IL-1β + AA
Poly(I:C) + AA
Treatment

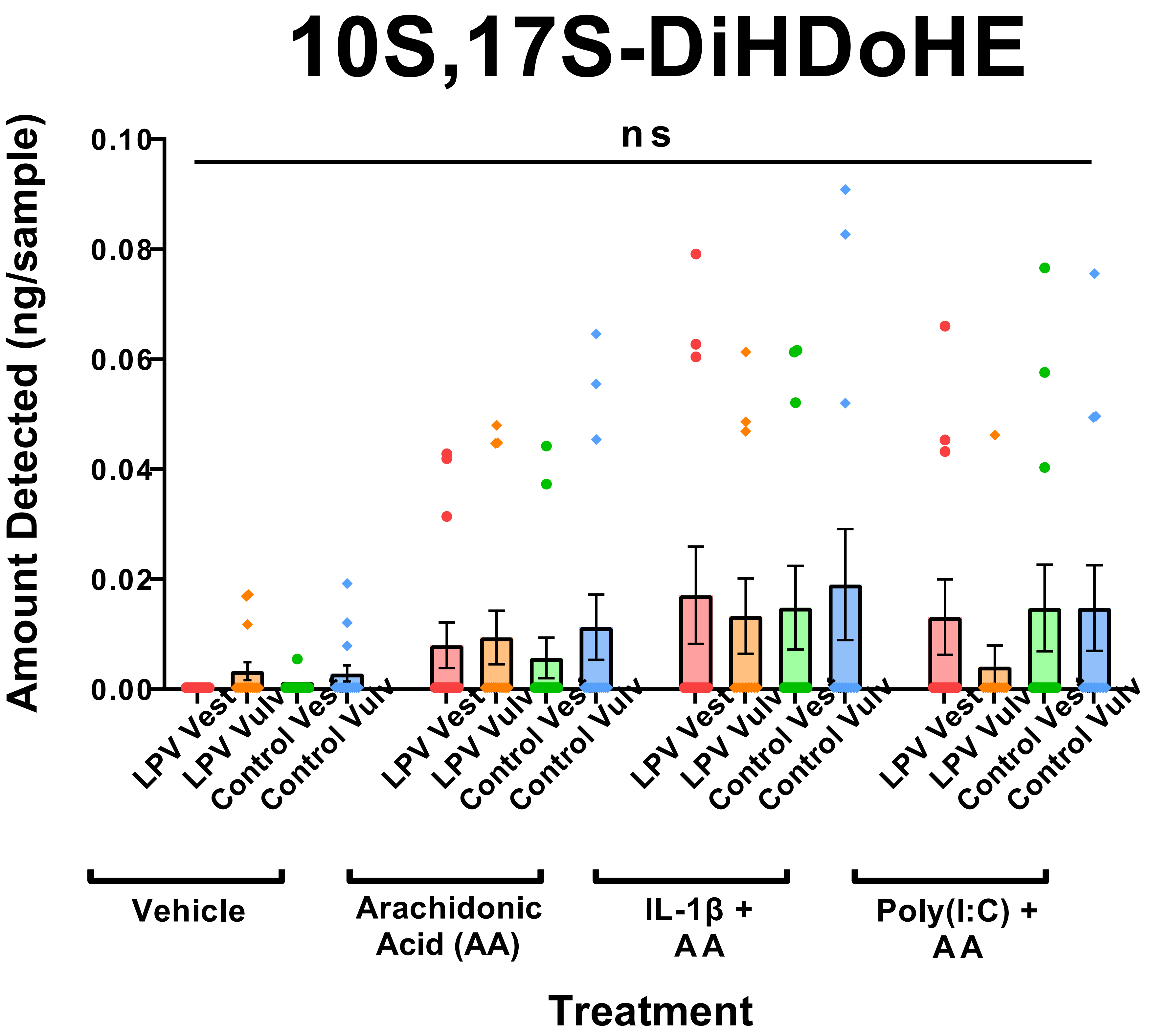
10S,17S-DiHDoHE
Amount Detected (ng/sample)
0.10
0.08
0.06
0.04
0.02
0.00
ns
LPV Vest
LPV Vulv
Control Vest
Control Vulv
Vehicle
Arachidonic Acid (AA)
IL-1β + AA
Poly(I:C) + AA
Treatment

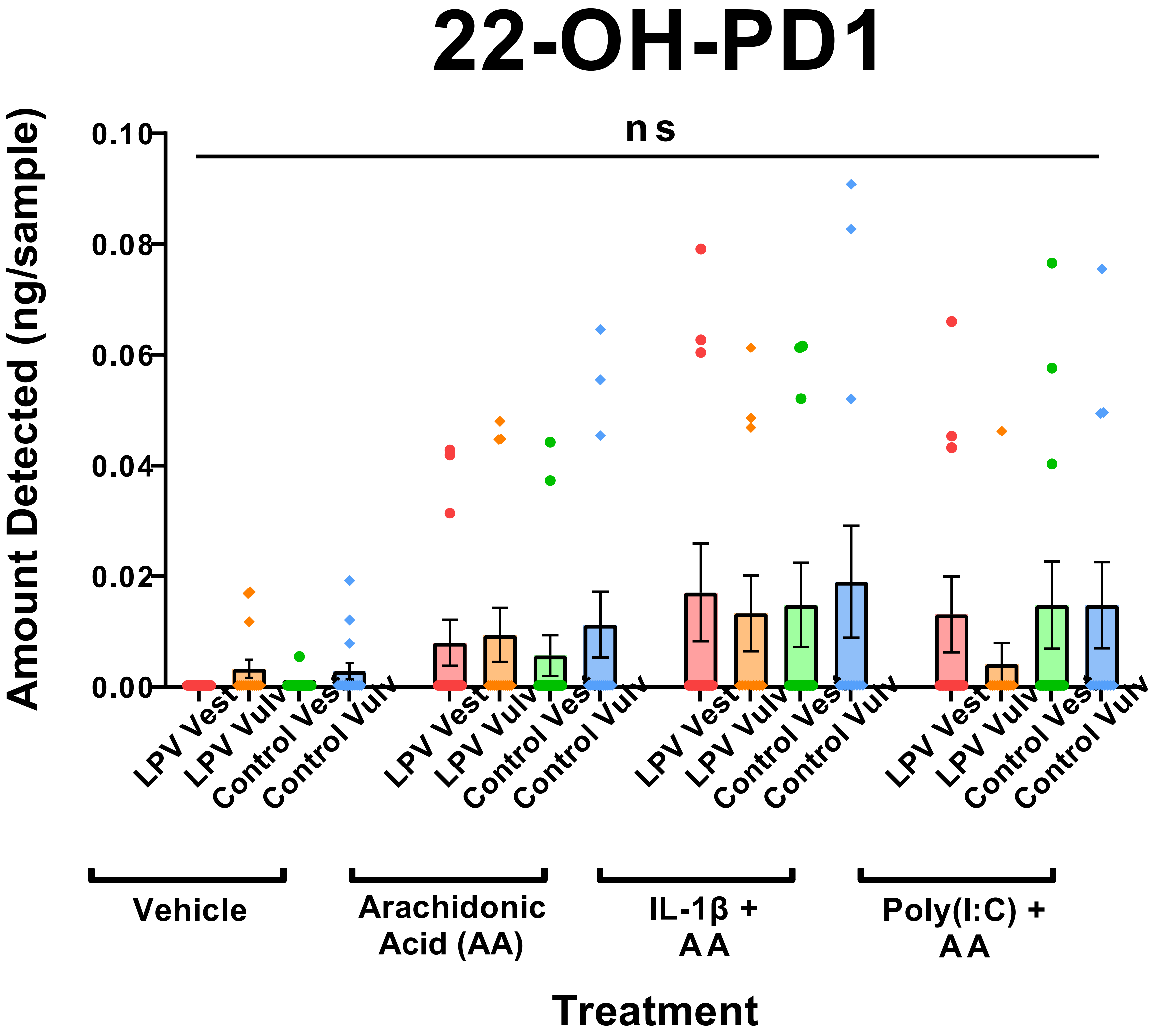

22-OH-PD1
Amount Detected (ng/sample)
0.10
0.08
0.06
0.04
0.02
0.00
ns
LPV Vest
LPV Vulv
Control Vest
Control Vulv
Vehicle
Arachidonic Acid (AA)
IL-1β + AA
Poly(I:C) + AA
Treatment

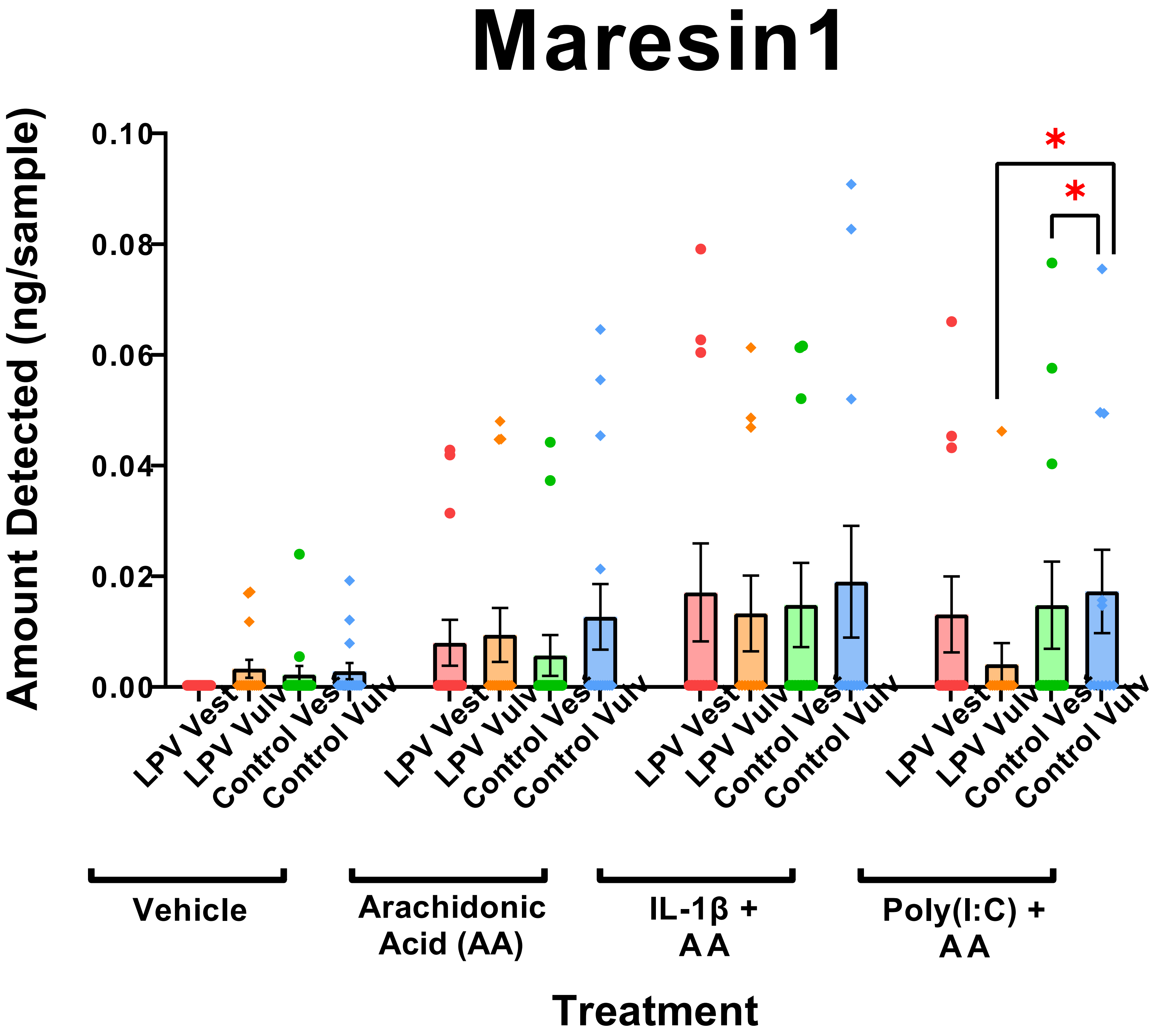

Maresin1
Amount Detected (ng/sample)
0.10
0.08
0.06
0.04
0.02
0.00
*
*
LPV Vest
LPV Vulv
Control Vest
Control Vulv
Vehicle
Arachidonic Acid (AA)
IL-1β + AA
Poly(I:C) + AA
Treatment

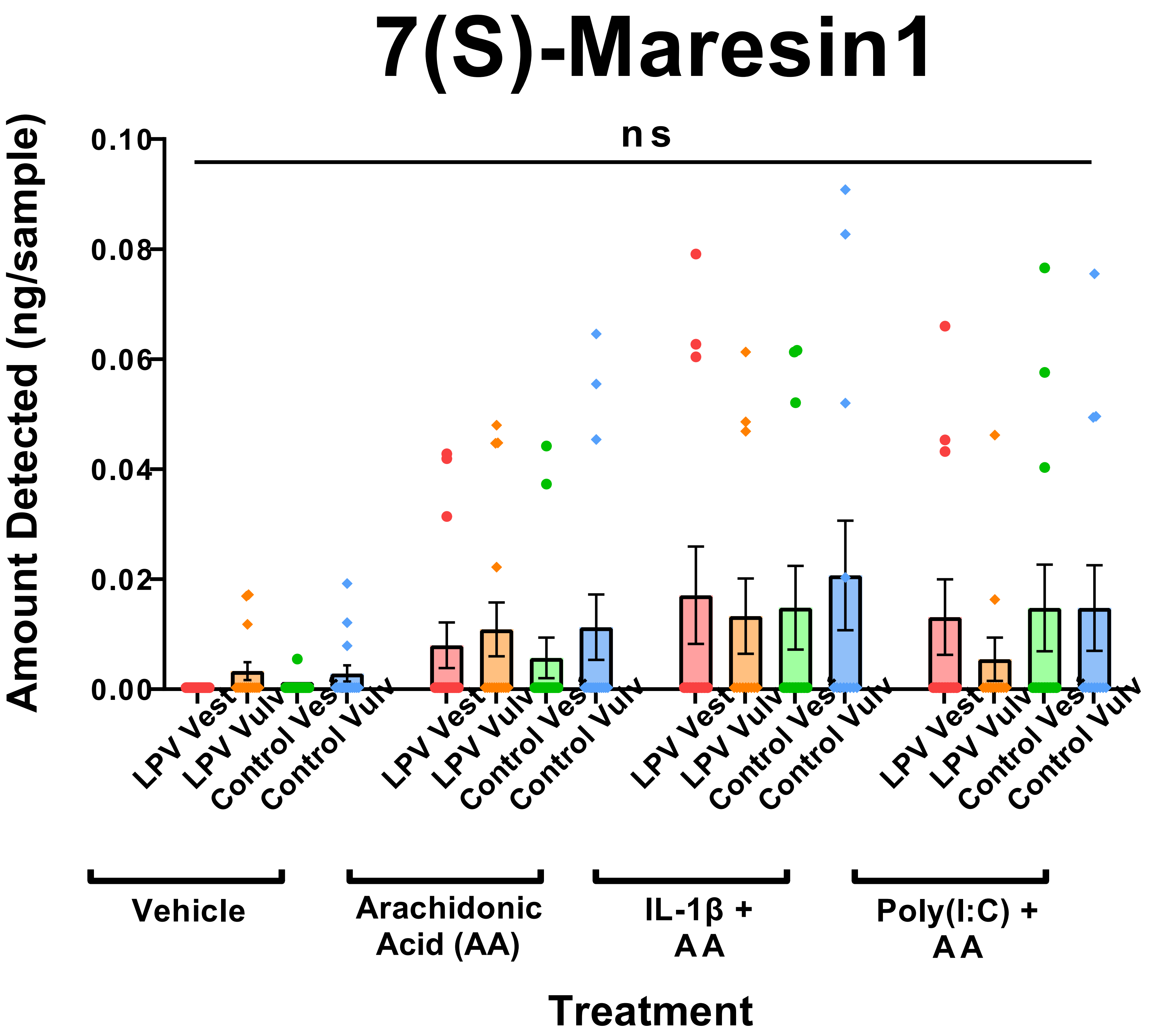

7(S)-Maresin1
ns
Amount Detected (ng/sample)
0.10
0.08
0.06
0.04
0.02
0.00
LPV Vest
LPV Vulv
Control Vest
Control Vulv
Vehicle
Arachidonic Acid (AA)
IL-1β + AA
Poly(I:C) + AA
Treatment

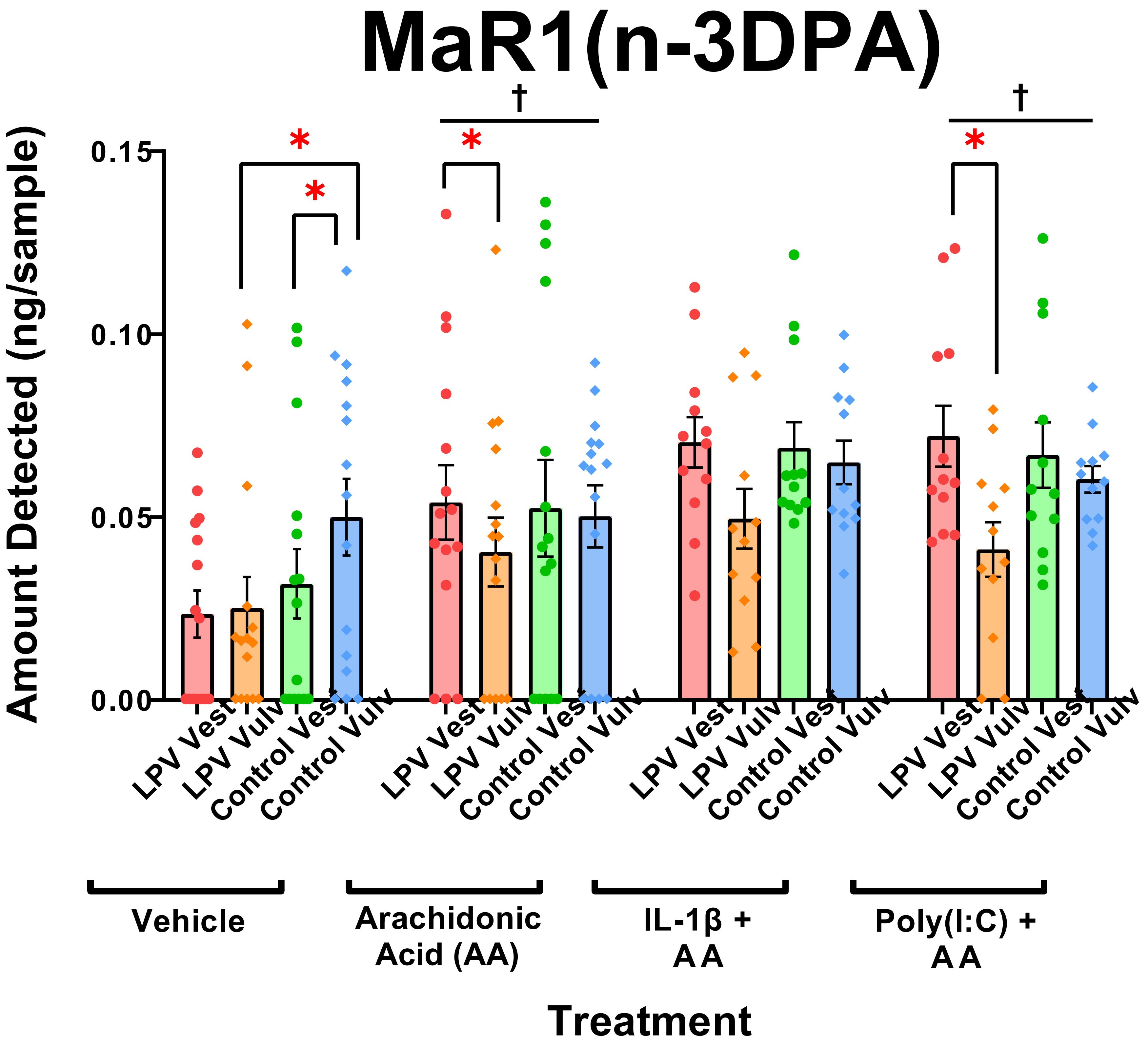
MaR1(n-3DPA)
Amount Detected (ng/sample)
0.15
0.10
0.05
0.00
*
*
*
†
*
†
LPV Vest
LPV Vulv
Control Vest
Control Vulv
Vehicle
Arachidonic Acid (AA)
IL-1β + AA
Poly(I:C) + AA
Treatment

Supplementary Figure S5: All Individual Lipid Bar Plots. For lipid bar graphs the following treatments were plotted: vehicle, AA, IL-1B + AA, PolyIC + IL-1B to align with the lipid heatmap and Tanzy's formal analyses. Statistically significant location differences (case vs. control and vest vs. vulv) with
p<0.05 were denoted with a red *. Statistically significant treatment differences (vehicle vs. AA vs. IL-1B + AA vs. PolyIC + AA) with p<0.05 were denoted with the following symbols († = significant from vehicle; ‡ = significant from AA; # = significant between IL1B + AA and PolyIC + AA).

Target	Gene	Forward Primer (5'->3')	Reverse primer (5’->3’)	Product Length (BP)	Source
18S Ribosomal RNA	*18S*	GTAACCCGTTG AACCCCATT	CCATCCAATCGG TAGTAGCG	151	[1]
Cyclooxygenase-1	*PTGS1*	TGCGCTCCAAC CTTATCCCC	CTTGGTGAAGC CAGGACCCA	311	NCBI
Cyclooxygenase-2	*PTGS2*	TCACAGGCTTC CATTGACCAG	CCGAGGCTTTTC TACCAGA	198	[2]
Arachidonate 5-Lipoxygenase	*ALOX5*	ATGCCCTCCTA CACGGTCAC	GTATGAATCCAC CGCGCCAC	162	NCBI
Arachidonate 12-Lipoxygenase	*ALOX12*	CTCTGGAGATG GCCCTCAAA	GAAGCTCTTCC ATCCCCGAG	237	NCBI
Arachidonate 15-Lipoxygenase	*ALOX15*	CACGGGGCAA GGAGACAGAA	TCCACCCAGCG GTAACAAGG	178	NCBI
Arachidonate 15-Lipoxygenase Type B	*ALOX15B*	CTGATCCCGCA CACCCGATA	GCCTTCAATGCC GATGCCTG	108	NCBI

Supplementary Table S2. List of primers used for quantitative polymerase chain reaction (qPCR). Amplification targets and corresponding gene names are provided. All forward and reverse primer sequences are listed with 5’ to 3’ directionality and product lengths are listed in terms of base pairs (BP). Primer sequences were identified using National Center for Biotechnology Information (NCBI) blast and Integrated DNA Technology (IDT) OligoAnalyzer™ tools or from the cited references.

Primer References

1. Schmittgen, T.D.; Zakrajsek, B.A. Effect of Experimental Treatment on Housekeeping Gene Expression: Validation by Real-Time, Quantitative RT-PCR. *J Biochem Biophys Methods* **2000**, *46*, 69–81, doi:10.1016/s0165-022x(00)00129-9.

2. Sheridan, J.A.; Zago, M.; Nair, P.; Li, P.Z.; Bourbeau, J.; Tan, W.C.; Hamid, Q.; Eidelman, D.H.; Benedetti, A.L.; Baglole, C.J. Decreased Expression of the NF-KB Family Member RelB in Lung Fibroblasts from Smokers with and without COPD Potentiates Cigarette Smoke-Induced COX-2 Expression. *Respir Res* **2015**, *16*, 54, doi:10.1186/s12931-015-0214-6.

Supplementary Table S3. List of lipids quantified using metabololipidomics:

Number	Lipid Name
1	13,14dhPGE1
2	13,14dh-15k-PGE1
3	D17-PGE1
4	PGE1
5	15(R)-PGE1
6	15-keto PGE1
7	Bicyclo PGE1
8	19(R)-hydroxy PGE1
9	2,3-dinor PGE1
10	PGE2
11	15-keto PGE2
12	13,14dh-15k-PGE2
13	Bicyclo PGE2
14	PGA2
15	19(R)-OH PGE2 & 20-OH PGE2
16	tetranor PGEM
17	PGE3
18	PGD2
19	PGJ2
20	D12-PGJ2
21	15d-D12,14-PGJ2
22	13,14dh-15k-PGD2
23	PGD3
24	15d-D12,14-PGJ3
25	PGF1a
26	PGF2a
27	15-keto PGF2a
28	13,14dh-15k-PGF2a
29	19(R)-OH PGF2a & 20-OH PGF2a
30	PGF3a
31	8-isoPGF2a & 11bPGF2a
32	iPF-VI
33	6kPGF1a
34	6-keto PGE1
35	6,15-diketo PGFa
36	TXB2
37	11dh-TXB2

38	2,3-dinor TXB2
39	11dh-2,3-dinor TXB2
40	TXB3
41	11dh TXB3
42	LTB4
43	12-OxoLTB4
44	20-hydroxy LTB4
45	20-COOH LTB4
46	18-carboxy dinor LTB4
47	LTB5
48	5(S),6(S)-DiHETE
49	5(S),12(S)-DiHETE
50	5(S),15(S)-DiHETE
51	8(S),15(S)-DiHETE
52	5(S),15(S)-DiHEPE
53	9-HODE
54	13-HODE
55	9(S)-HOTrE
56	13(S)-HOTrE
57	13(S)-HOTrE(g)
58	11(R)-HEDE
59	15(S)-HEDE
60	8(S)-HETrE
61	5(S)-HETrE
62	5-HETE
63	8-HETE
64	9-HETE
65	11-HETE
66	12-HETE
67	15-HETE
68	20-HETE
69	tetranor 12-HETE
70	12(S)-HHTrE
71	5-HEPE
72	8-HEPE
73	9-HEPE
74	11-HEPE
75	12-HEPE
76	15(S)-HEPE
77	18-HEPE
78	4-HDoHE

79	7-HDoHE
80	8-HDoHE
81	10-HDoHE
82	11-HDoHE
83	13-HDoHE
84	14-HDoHE
85	16-HDoHE
86	17-HDoHE
87	20-HDoHE
88	9(10)-EpOME
89	12(13)-EpOME
90	5(6)-EpETrE
91	8(9)-EpETrE
92	11(12)-EpETrE
93	14(15)-EpETrE
94	8(9)-EpETE
95	11(12)-EpETE
96	14(15)-EpETE
97	17(18)-EpETE
98	7(8)-EpDPE
99	10(11)-EpDPE
100	13(14)-EpDPE
101	16(17)-EpDPE
102	19(20)-EpDPE
103	9,10-DiHOME
104	12,13-DiHOME
105	5,6-DiHETrE
106	8,9-DiHETrE
107	11,12-DiHETrE
108	14,15-DiHETrE
109	5,6-DiHETE(EPA)
110	19,20-DiHDoPE
111	9-OxoODE
112	13-OxoODE
113	9-OxoOTrE
114	15-OxoEDE
115	5-oxoETE
116	12-OxoETE
117	15-OxoETE
118	LXA4
119	15-epi LXA4

120	15-oxo LXA4
121	LXA5
122	LXB4
123	RvD1 & AT-RvD1
124	RvD2
125	RvD3
126	AT-RvD3
127	RvD4
128	RvD5
129	RvD6 (4,17-DiHDoHE)
130	8-oxoRvD1
131	17-oxoRvD1
132	RvD5(n-3DPA) (7,17-DiHDoPE)
133	RvE1
134	RvE2
135	RvE3
136	PD1
137	AT-PD1
138	PD1(n-3, DPA)
139	10S,17S-DiHDoHE
140	22-OH-PD1
141	Maresin1
142	7(S)-Maresin1
143	MaR1(n-3DPA)

Supplementary Table S4: All Arachidonic Acid Metabololipidomics Data

Lipid	CO 10 VEST AA 1 19	CO 10 VEST AA 2 20	CO 10 VEST AA 3 21	CO 10 VULV AA 1 22	CO 10 VULV AA 2 23
13,14dhPGE1	0.0003	0.0003	0.0003	0.0003	0.0003
13,14dh-15k-PGE1	0.0003	0.0003	0.0003	0.0003	0.0003
D17-PGE1	0.0003	0.0003	0.0003	0.0003	0.0003
PGE1	0.0003	0.1602	0.0003	0.0003	0.0003
15(R)-PGE1	0.5226	0.5182	0.5839	0.5407	0.7937
15-keto PGE1	0.0003	0.0003	0.0003	0.0003	0.0003
Bicyclo PGE1	0.0003	0.0356	0.0337	0.0264	0.0299
19(R)-hydroxy PGE1	0.0003	0.0003	0.0003	0.0003	0.0003
2,3-dinor PGE1	0.0003	0.0003	0.0003	0.0003	0.0003
PGE2	37.7023	42.0307	38.6764	49.5230	58.1266
15-keto PGE2	0.0559	0.0572	0.0681	0.0717	0.0693
13,14dh-15k-PGE2	1.5602	1.7670	1.6603	2.1242	2.4441
Bicyclo PGE2	0.0003	0.9316	0.0003	1.2754	1.9524
PGA2	8.3877	9.0334	5.2827	10.5044	15.7542
19(R)-OH PGE2 & 20-OH PGE2	0.0003	0.0003	0.0003	0.0003	0.0003
tetranor PGEM	0.0003	0.0003	0.0003	0.0003	0.0003
PGE3	0.0003	0.0003	0.0529	0.0644	0.1102
PGD2	0.0003	0.0003	0.0003	0.0003	0.0003
PGJ2	3.8295	4.0320	2.6282	5.4039	7.3620
D12-PGJ2	0.0003	0.0003	0.0003	0.0003	0.0003
15d-D12,14-PGJ2	0.0621	0.0614	0.0322	0.0843	0.1086
13,14dh-15k-PGD2	2.2969	2.6134	2.4422	2.8483	3.5544
PGD3	0.0003	0.0003	0.0003	0.0003	0.0003
15d-D12,14-PGJ3	0.0003	0.0003	0.0003	0.0003	0.0003
PGF1a	0.0003	0.0003	0.0003	0.0003	0.0003
PGF2a	0.1790	0.1753	0.1829	0.2860	0.3231
15-keto PGF2a	1.6656	1.7621	1.6694	2.2940	2.7788
13,14dh-15k-PGF2a	0.0003	0.0918	0.0553	0.0570	0.0549
19(R)-OH PGF2a & 20-OH PGF2a	0.0003	0.0003	0.0003	0.0003	0.0003
PGF3a	0.0003	0.0003	0.0003	0.0003	0.0003
8-isoPGF2a & 11bPGF2a	0.0003	0.0003	0.0003	0.0329	0.0558
iPF-VI	0.2858	0.3958	0.3022	0.1870	0.2382
6kPGF1a	1.3300	1.4245	1.6401	8.0575	10.2616

6-keto PGE1	0.0003	0.0003	0.0003	0.0003	0.0003
6,15-diketo PGFa	0.0003	0.0003	0.0032	0.0003	0.0003
TXB2	0.0003	0.0734	0.0886	0.0773	0.1037
11dh-TXB2	0.0003	0.0261	0.0003	0.0003	0.0003
2,3-dinor TXB2	0.0003	0.0003	0.0003	0.0003	0.0003
11dh-2,3-dinor TXB2	0.0003	0.0057	0.0003	0.0003	0.0003
TXB3	0.0003	0.0003	0.0003	0.0003	0.0003
11dh TXB3	0.0003	0.0003	0.0003	0.0003	0.0003
LTB4	0.5715	0.7321	0.6361	0.7655	0.8451
12-OxoLTB4	0.0003	0.0003	0.0003	0.0003	0.0003
20-hydroxy LTB4	0.0003	0.0003	0.0003	0.0003	0.0003
20-COOH LTB4	0.0003	0.0003	0.0338	0.0482	0.0033
18-carboxy dinor LTB4	0.0003	0.0003	0.0003	0.0003	0.0003
LTB5	0.0003	0.0003	0.0003	0.0003	0.0003
5(S),6(S)-DiHETE	0.0003	0.0003	0.0003	0.0003	0.0003
5(S),12(S)-DiHETE	0.0003	0.0218	0.0258	0.0333	0.0003
5(S),15(S)-DiHETE	0.4273	0.5087	0.6151	0.6312	0.7668
8(S),15(S)-DiHETE	0.7279	0.9179	0.9236	1.0602	1.0446
5(S),15(S)-DiHEPE	0.0932	0.1992	0.0306	0.0594	0.0328
9-HODE	0.0988	0.1410	0.1465	0.1484	0.0003
13-HODE	2.4412	3.2791	3.2424	5.0042	5.5372
9(S)-HOTrE	0.0003	0.0003	0.0003	0.0003	0.0311
13(S)-HOTrE	0.0003	0.0003	0.0003	0.0003	0.0003
13(S)-HOTrE(g)	0.0003	0.0003	0.0003	0.0003	0.0003
11(R)-HEDE	0.0003	0.0003	0.0003	0.0003	0.0003
15(S)-HEDE	0.0003	0.0003	0.0003	0.0003	0.0003
8(S)-HETrE	0.1370	0.1093	0.0794	0.2491	0.2250
5(S)-HETrE	0.0003	0.0003	0.0003	0.0003	0.0003
5-HETE	2.9525	1.4634	1.4124	6.5518	5.1787
8-HETE	1.8841	1.0991	0.9839	4.1214	3.1025
9-HETE	0.9324	0.7969	0.5687	1.9901	1.3801
11-HETE	17.0505	9.7057	10.8246	52.8247	46.2813
12-HETE	13.0208	9.8686	5.6801	61.6053	71.6140
15-HETE	33.0964	17.4617	12.0040	93.0322	75.6944

20-HETE	1.1493	1.0924	0.9947	5.4022	4.9917
tetranor 12-HETE	88.0156	92.2540	118.0208	70.4912	66.5141
12(S)-HHTrE	1.8179	1.1792	1.7994	5.7179	9.9079
5-HEPE	0.0003	0.0522	0.0832	0.1758	0.1439
8-HEPE	0.0003	0.0331	0.0003	0.1446	0.1297
9-HEPE	0.0003	0.0003	0.0003	0.0003	0.0003
11-HEPE	0.1247	0.1359	0.1137	0.4565	0.4743
12-HEPE	0.1122	0.1241	0.1247	0.3718	0.4150
15(S)-HEPE	0.2307	0.2253	0.1621	0.5943	0.5472
18-HEPE	0.2385	0.2362	0.2076	1.1823	0.9967
4-HDoHE	0.1955	0.1562	0.1421	0.3949	0.3784
7-HDoHE	0.0270	0.0358	0.0003	0.0529	0.0529
8-HDoHE	0.0934	0.0935	0.1120	0.2122	0.2086
10-HDoHE	0.0954	0.1146	0.1168	0.2073	0.2064
11-HDoHE	0.0615	0.1018	0.0904	0.2523	0.2434
13-HDoHE	0.3522	0.3706	0.4593	0.9892	1.1019
14-HDoHE	0.1082	0.1263	0.0942	0.2451	0.3031
16-HDoHE	0.2848	0.2856	0.2811	0.6024	0.5985
17-HDoHE	0.0003	0.0003	0.0003	0.0003	0.0003
20-HDoHE	0.3103	0.2776	0.2925	0.7954	0.9022
9(10)-EpOME	0.0442	0.0956	0.0545	0.0559	0.0486
12(13)-EpOME	0.0185	0.0588	0.0003	0.0282	0.0003
5(6)-EpETrE	0.0238	0.0254	0.0265	0.0502	0.0519
8(9)-EpETrE	0.0003	0.0003	0.0003	0.0003	0.0003
11(12)-EpETrE	0.0003	0.0003	0.0003	0.0003	0.0003
14(15)-EpETrE	0.0003	0.0003	0.0003	0.0003	0.0003
8(9)-EpETE	0.0003	0.0003	0.0003	0.0003	0.0003
11(12)-EpETE	0.0003	0.0003	0.0003	0.0403	0.0003
14(15)-EpETE	0.0003	0.0003	0.0003	0.0003	0.0003
17(18)-EpETE	0.0003	0.0003	0.0003	0.0003	0.0003
7(8)-EpDPE	0.0003	0.0003	0.0003	0.0003	0.0003
10(11)-EpDPE	0.0003	0.0003	0.0003	0.0144	0.0003
13(14)-EpDPE	0.0003	0.0003	0.0003	0.0003	0.0003
16(17)-EpDPE	0.0003	0.0003	0.0003	0.0003	0.0003

19(20)-EpDPE	0.0060	0.0003	0.0003	0.0003	0.0003
9,10-DiHOME	0.0003	0.0091	0.0003	0.0149	0.0113
12,13-DiHOME	0.0003	0.0137	0.0158	0.0129	0.0003
5,6-DiHETrE	0.0804	0.0880	0.0541	0.4075	0.4742
8,9-DiHETrE	0.0143	0.0114	0.0107	0.0941	0.1019
11,12-DiHETrE	0.3564	0.4320	0.3337	1.2477	1.4919
14,15-DiHETrE	1.8183	2.0759	2.5034	2.4909	3.1551
5,6-DiHETE(EPA)	0.0241	0.0003	0.0233	0.0003	0.0289
19,20-DiHDoPE	0.0141	0.0223	0.0215	0.0249	0.0429
9-OxoODE	0.0933	0.1435	0.3707	0.2941	0.2958
13-OxoODE	0.1987	0.1882	0.2264	0.2614	0.2902
9-OxoOTrE	0.0316	0.0003	0.0278	0.0003	0.0003
15-OxoEDE	0.0003	0.0003	0.0003	0.0003	0.0003
5-oxoETE	0.5638	0.3734	0.2921	0.8325	0.7070
12-OxoETE	0.0003	0.0003	0.0003	0.0003	0.0003
15-OxoETE	0.0003	0.0003	0.0003	0.0003	0.3553
LXA4	1.3176	1.7184	1.9096	1.8804	2.1744
15-epi LXA4	0.0003	0.0003	0.0595	0.0003	0.0003
15-oxo LXA4	0.0373	0.0442	0.0003	0.0646	0.0555
LXA5	0.0003	0.0003	0.0081	0.0003	0.0003
LXB4	0.0003	0.0003	0.0003	0.0003	0.0003
RvD1 & AT-RvD1	0.0003	0.0003	0.0003	0.0003	0.0003
RvD2	0.0003	0.0003	0.0003	0.0003	0.0003
RvD3	0.0003	0.0003	0.0003	0.0003	0.0003
AT-RvD3	0.0003	0.0003	0.0003	0.0003	0.0003
RvD4	0.0003	0.0003	0.0003	0.0003	0.0003
RvD5	0.0003	0.0003	0.0003	0.0003	0.0003
RvD6 (4,17-DiHDoHE)	0.0003	0.0003	0.0003	0.0099	0.0099
8-oxoRvD1	0.0003	0.0003	0.0003	0.0003	0.0003
17-oxoRvD1	0.0003	0.0003	0.0003	0.0003	0.0003
RvD5(n-3DPA) (7,17-DiHDoPE)	0.0003	0.0003	0.0003	0.0003	0.0003
RvE1	0.0003	0.0003	0.0003	0.0003	0.0003
RvE2	0.0003	0.0003	0.0003	0.0041	0.0003
RvE3	0.0003	0.0003	0.0003	0.0003	0.0003

PD1	0.0003	0.0003	0.0003	0.0003	0.0003
AT-PD1	0.0003	0.0003	0.0003	0.0003	0.0003
PD1(n-3, DPA)	0.0003	0.0003	0.0003	0.0003	0.0003
10S,17S-DiHDoHE	0.0003	0.0003	0.0003	0.0003	0.0003
22-OH-PD1	0.0003	0.0003	0.0003	0.0003	0.0003
Maresin1	0.0003	0.0003	0.0003	0.0003	0.0003
7(S)-Maresin1	0.0003	0.0003	0.0003	0.0003	0.0003
MaR1(n-3DPA)	0.0003	0.0419	0.0353	0.0630	0.0003

CO 10 VULV AA 3 24	CO 10 VEST IL + AA 1 25	CO 10 VEST IL + AA 2 26	CO 10 VEST IL + AA 3 27	CO 10 VULV IL + AA 1 28
0.0003	0.0003	0.0003	0.0003	0.0003
0.0003	0.0251	0.0305	0.0431	0.0262
0.0003	0.0003	0.0003	0.0003	0.0003
0.0003	2.9055	2.3374	2.0063	1.0005
0.5556	2.4108	2.0275	2.3773	1.7999
0.0003	0.0003	0.0003	0.0003	0.0003
0.0003	0.0003	0.0003	0.0003	0.0335
0.0003	0.0003	0.0003	0.0003	0.0003
0.0003	0.0003	0.0222	0.0003	0.0003
50.8957	220.1853	195.1764	197.5979	144.1351
0.0528	0.1943	0.3415	0.1785	0.1624
2.1171	10.8454	9.6484	9.2184	7.1287
0.0003	0.0003	0.0003	0.0003	0.0003
9.0394	53.5184	48.6412	45.6445	45.3728
0.0003	0.0003	0.0003	0.0003	0.0003
0.0003	0.0003	0.0003	0.0003	0.0003
0.0732	0.4179	0.4405	0.3572	0.2750
0.0003	0.0003	0.0003	0.0003	0.3456
4.1228	23.1137	21.3793	19.3621	20.5644
0.0003	0.0003	0.0003	0.0003	0.0003
0.0578	0.0789	0.0594	0.0862	0.0971
2.7880	2.4879	3.5111	3.7173	2.7130
0.0003	0.0003	0.0003	0.0003	0.0003
0.0003	0.0003	0.0003	0.0003	0.0003
0.0003	0.0708	0.0754	0.0585	0.0206
0.2961	2.3536	2.4238	2.0137	1.3399
2.2047	12.5390	12.3541	11.4502	8.7462
0.0613	0.0714	0.0654	0.0634	0.0720
0.0003	0.0003	0.0003	0.0003	0.0003
0.0003	0.0003	0.0003	0.0003	0.0003
0.0003	0.1643	0.1316	0.1150	0.0738
0.2252	0.3269	0.3290	0.3487	0.2746
9.6565	4.7954	4.9302	4.8483	18.8007

0.0003	0.0003	0.0003	0.0003	0.0003
0.0003	0.0003	0.0003	0.0003	0.0003
0.0739	0.0757	0.1328	0.0932	0.1376
0.0003	0.0379	0.0003	0.0003	0.0003
0.0003	0.0003	0.0003	0.0003	0.0003
0.0003	0.0003	0.0003	0.0003	0.0003
0.0003	0.0003	0.0003	0.0003	0.0003
0.0003	0.0003	0.0003	0.0003	0.0003
0.5982	0.6944	0.6166	0.7249	0.7222
0.0003	0.0003	0.0003	0.0003	0.0003
0.0003	0.0003	0.0003	0.0003	0.0062
0.0711	0.0003	0.0122	0.0003	0.0003
0.0003	0.0003	0.0003	0.0003	0.0003
0.0003	0.0003	0.0003	0.0003	0.0003
0.0003	0.0003	0.0003	0.0003	0.0003
0.0003	0.0175	0.0003	0.0003	0.0425
0.5417	0.7209	0.7001	0.5695	0.6373
0.8471	1.0331	0.7649	0.9562	1.0008
0.0359	0.0591	0.0795	0.1036	0.0402
0.2189	0.2204	0.1715	0.0003	0.1235
5.0803	4.0023	2.5962	1.7600	7.3570
0.0003	0.0003	0.0003	0.0003	0.0003
0.0003	0.0003	0.0003	0.0003	0.0003
0.0003	0.0003	0.0003	0.0003	0.0003
0.0003	0.0003	0.0425	0.0476	0.0290
0.0003	0.0003	0.0003	0.0114	0.0102
0.1690	0.1298	0.1366	0.0921	0.1135
0.0003	0.0003	0.0003	0.0003	0.0003
2.4564	3.5431	2.5852	2.3655	3.5611
1.4550	2.0806	1.2241	1.0848	1.8350
0.9140	0.9309	0.7056	0.6067	0.9173
29.8799	64.3983	42.9478	37.8877	54.5685
38.3409	10.4321	4.8986	2.9077	38.6181
27.6921	46.2463	24.1454	14.1583	71.6235

3.6725	0.0003	0.0003	0.4279	2.8829
112.4306	141.1739	139.2134	133.9623	98.5152
6.3115	6.5407	4.8969	3.1571	17.3446
0.1253	0.0553	0.0003	0.0003	0.0890
0.0003	0.0003	0.0003	0.0003	0.0574
0.0003	0.0003	0.0003	0.0003	0.0003
0.3935	0.5534	0.3942	0.2563	0.8953
0.3826	0.1264	0.0003	0.0727	0.0003
0.4120	0.6763	0.5327	0.2733	0.7868
0.8015	0.1364	0.0003	0.0768	0.5899
0.3224	0.2042	0.1784	0.1774	0.2695
0.0690	0.0003	0.0309	0.0333	0.0326
0.1758	0.1051	0.0898	0.1063	0.1097
0.1265	0.1002	0.0834	0.1193	0.1004
0.1918	0.1243	0.0609	0.1033	0.1256
1.0167	1.0203	0.7057	0.6542	0.9904
0.1707	0.1763	0.1050	0.0973	0.2450
0.5703	0.3657	0.2754	0.2375	0.5308
0.0003	0.0003	0.0003	0.0003	0.0003
0.6328	0.2843	0.2316	0.2349	0.5486
0.0720	0.0507	0.0398	0.0456	0.0408
0.0300	0.0249	0.0232	0.0326	0.0236
0.0538	0.0237	0.0315	0.0289	0.0383
0.0003	0.0003	0.0003	0.0003	0.0003
0.0003	0.0003	0.0003	0.0003	0.0003
0.0003	0.0003	0.0003	0.0383	0.0003
0.0003	0.0003	0.0068	0.0003	0.0003
0.0003	0.0003	0.0003	0.0003	0.0003
0.0003	0.0003	0.0115	0.0003	0.0287
0.0003	0.0003	0.0003	0.0003	0.0003
0.0003	0.0003	0.0003	0.0003	0.0003
0.0003	0.0003	0.0003	0.0003	0.0090
0.0003	0.0003	0.0003	0.0003	0.0003
0.0003	0.0003	0.0003	0.0003	0.0003

0.0003	0.0054	0.0003	0.0003	0.0003
0.0103	0.0090	0.0003	0.0003	0.0098
0.0003	0.0096	0.0073	0.0003	0.0091
0.1870	0.0660	0.0311	0.0405	0.1901
0.0362	0.0208	0.0003	0.0003	0.0453
0.6881	0.2966	0.2281	0.1780	0.8228
2.7365	2.0799	2.0307	2.3287	2.5839
0.0349	0.0181	0.0003	0.0003	0.0228
0.0222	0.0168	0.0156	0.0149	0.0238
0.2875	0.3366	0.2090	0.2090	0.1294
0.1591	0.2601	0.3829	0.2848	0.3358
0.0167	0.0275	0.0003	0.0170	0.0156
0.0003	0.0003	0.0003	0.0003	0.0003
0.3650	0.8736	0.5186	0.5689	0.6531
0.0003	0.0003	0.0003	0.0003	0.0003
0.0003	0.4464	0.0003	0.3559	0.0003
1.8308	1.7750	1.2380	1.9447	1.9020
0.0003	0.0003	0.0003	0.0003	0.0003
0.0454	0.0613	0.0616	0.0521	0.0827
0.0003	0.0055	0.0003	0.0003	0.0003
0.0003	0.0003	0.0003	0.0003	0.0003
0.0003	0.0003	0.0003	0.0003	0.0003
0.0003	0.0003	0.0003	0.0003	0.0003
0.0003	0.0003	0.0003	0.0003	0.0074
0.0003	0.0003	0.0003	0.0003	0.0003
0.0003	0.0003	0.0003	0.0003	0.0003
0.0003	0.0003	0.0003	0.0003	0.0003
0.0094	0.0003	0.0003	0.0003	0.0123
0.0003	0.0003	0.0003	0.0003	0.0003
0.0003	0.0003	0.0003	0.0003	0.0003
0.0003	0.0003	0.0003	0.0003	0.0003
0.0003	0.0003	0.0003	0.0003	0.0003
0.0003	0.0003	0.0003	0.0003	0.0051
0.0003	0.0003	0.0003	0.0003	0.0003

0.0003	0.0376	0.0003	0.0367	0.0433
0.0003	0.0003	0.0003	0.0003	0.0003
0.0003	0.0003	0.0003	0.0003	0.0003
0.0003	0.0003	0.0003	0.0003	0.0003
0.0003	0.0003	0.0003	0.0003	0.0003
0.0003	0.0003	0.0003	0.0003	0.0003
0.0003	0.0003	0.0003	0.0003	0.0003
0.0700	0.0483	0.0541	0.0583	0.0782

CO 10 VULV IL + AA 2 29	CO 10 VULV IL + AA 3 30	CO 10 VEST POLY IC + AA 1 31	CO 10 VEST POLY IC + AA 2 32	CO 10 VEST POLY IC + AA 3 33
0.0003	0.0003	0.0003	0.0003	0.0003
0.0231	0.0241	0.0151	0.0660	0.0372
0.0003	0.0003	0.0003	0.0003	0.0003
0.8184	1.0250	1.7370	7.2133	5.9387
2.4842	2.5988	1.7728	2.2426	2.2018
0.0003	0.0003	0.0003	0.0003	0.0003
0.0003	0.0465	0.0003	0.0003	0.0702
0.0003	0.0003	0.0003	0.0003	0.0003
0.0003	0.0003	0.0003	0.0493	0.0612
206.1644	231.8310	175.3267	207.8182	180.4185
0.1508	0.1606	0.2422	0.5248	0.4895
9.7127	10.5368	7.2787	11.2048	10.7776
0.0003	0.0003	0.0003	0.0003	4.9248
52.0336	63.6526	31.0902	44.7254	36.2737
0.0003	0.0003	0.0003	0.0003	0.0003
0.0003	0.0003	0.0003	0.0003	0.0003
0.2635	0.3199	0.2566	0.9863	0.8828
0.0003	0.0003	0.0003	0.0003	0.0003
22.0947	29.4066	14.5643	19.6681	17.3311
0.0003	0.0003	0.0003	0.0003	0.0003
0.1167	0.1381	0.0851	0.0725	0.0558
5.6867	6.3761	3.3608	8.1623	3.4780
0.0003	0.0003	0.0003	0.0003	0.0003
0.0003	0.0003	0.0003	0.0003	0.0003
0.0143	0.0181	0.0514	0.2101	0.1725
1.5267	1.3873	1.2704	3.0928	2.3834
11.1148	12.1125	8.2908	13.1212	11.9578
0.0783	0.0525	0.0810	0.0677	0.0464
0.0003	0.0003	0.0003	0.0003	0.0003
0.0003	0.0003	0.0003	0.0003	0.0003
0.0685	0.0932	0.0773	0.1817	0.1498
0.2847	0.2653	0.1395	0.3591	0.1782
21.9014	21.5465	3.3996	5.6013	5.0721

0.0003	0.0003	0.0003	0.0003	0.0003
0.0003	0.0003	0.0003	0.0003	0.0003
0.1248	0.1326	0.0687	0.1290	0.0853
0.0003	0.0003	0.0896	0.3342	0.2648
0.0003	0.0003	0.0003	0.0003	0.0003
0.0003	0.0003	0.0003	0.0003	0.0003
0.0003	0.0003	0.0003	0.0003	0.0003
0.0003	0.0003	0.0003	0.0003	0.0003
0.9305	0.7853	0.7024	0.7304	0.5285
0.0003	0.0003	0.0003	0.0003	0.0003
0.0003	0.0003	0.0003	0.0003	0.0003
0.0495	0.0003	0.0491	0.0254	0.0730
0.0003	0.0003	0.0003	0.0003	0.0003
0.0003	0.0003	0.0003	0.0003	0.0003
0.0003	0.0003	0.0003	0.0003	0.0003
0.0566	0.0519	0.0003	0.0003	0.0003
0.9884	0.7208	0.7324	0.5954	0.4881
2.2895	0.9517	1.0730	0.9403	0.7679
0.0382	0.0783	0.0734	0.0562	0.0353
0.2147	0.1119	0.0003	0.0003	0.0003
11.5390	8.9885	4.7149	5.4788	4.5026
0.0003	0.0003	0.0003	0.0003	0.0003
0.0003	0.0003	0.0003	0.0003	0.0003
0.0003	0.0003	0.0003	0.0003	0.0003
0.0003	0.0003	0.0003	0.0003	0.1229
0.0230	0.0100	0.0201	0.0100	0.0120
0.1664	0.1298	0.2769	0.1864	0.1623
0.0003	0.0003	0.0003	0.0003	0.0003
6.5463	3.6567	26.5934	3.4915	2.4428
3.4442	1.9795	15.2491	1.8735	1.0190
2.1765	1.0132	5.2023	0.8861	0.8240
87.2932	56.1794	188.1372	159.8989	151.9548
78.1676	45.3612	59.9600	10.3254	7.3619
151.1094	71.1750	222.2219	76.4869	63.1599

5.3492	3.1651	0.0003	0.0003	0.0003
107.1376	106.5992	65.2662	123.9144	109.2941
24.2978	19.1407	8.0945	9.1285	7.2394
0.1477	0.0624	0.1714	0.0720	0.0003
0.1243	0.0481	0.0556	0.0003	0.0003
0.0003	0.0003	0.0003	0.0003	0.0003
1.0945	0.8746	1.2667	1.5683	1.7600
0.4204	0.3987	0.1963	0.0911	0.0741
0.9399	0.7072	0.7052	1.0689	0.9061
0.9974	0.6063	0.4702	0.1945	0.1576
0.3300	0.2492	0.4447	0.3165	0.2557
0.0633	0.0720	0.0518	0.0244	0.0359
0.1731	0.1366	0.1542	0.0926	0.1167
0.1739	0.1379	0.1291	0.0813	0.1109
0.2009	0.1289	0.0003	0.0003	0.0003
1.5566	1.0715	1.8416	3.7121	4.3577
0.3430	0.2791	0.2682	0.2268	0.2193
0.7545	0.5964	0.4969	0.5989	0.5160
0.1072	0.0762	0.0638	0.1483	0.1407
1.0153	0.5125	0.6227	0.3226	0.2659
0.0525	0.0352	0.0588	0.0494	0.0376
0.0003	0.0003	0.0003	0.0133	0.0287
0.0635	0.0400	0.0454	0.0324	0.0392
0.0003	0.0003	0.0003	0.0003	0.0003
0.0003	0.0003	0.0003	0.0003	0.0003
0.0003	0.0003	0.0003	0.0003	0.0003
0.0003	0.0003	0.0003	0.0003	0.0003
0.0003	0.0003	0.0003	0.0003	0.0003
0.0585	0.0328	0.0683	0.0279	0.0370
0.0003	0.0003	0.0003	0.0003	0.0003
0.0003	0.0003	0.0047	0.0003	0.0003
0.0003	0.0073	0.0132	0.0003	0.0003
0.0003	0.0003	0.0003	0.0003	0.0003
0.0003	0.0003	0.0003	0.0003	0.0003

0.0003	0.0003	0.0003	0.0003	0.0003
0.0126	0.0094	0.0003	0.0110	0.0100
0.0136	0.0121	0.0101	0.0132	0.0160
0.0003	0.2176	0.2850	0.0550	0.0327
0.0794	0.0471	0.1109	0.0098	0.0003
1.2855	0.8522	1.0621	0.3322	0.2247
3.2425	2.5113	2.3612	1.9374	1.5266
0.0284	0.0371	0.0003	0.0226	0.0172
0.0290	0.0254	0.0175	0.0190	0.0158
0.1587	0.2276	0.0003	0.3030	0.2846
0.4712	0.4556	0.4369	0.0003	0.1888
0.0252	0.0003	0.0003	0.0003	0.0162
0.0003	0.0003	0.0003	0.0003	0.0003
0.9640	0.7329	2.8573	0.8862	0.5781
0.0003	0.0003	0.0003	0.0003	0.0003
0.0003	0.0003	0.0003	0.0003	0.0003
1.9603	1.7953	2.2811	1.8748	1.2888
0.0003	0.0003	0.0003	0.0003	0.0003
0.0908	0.0520	0.0766	0.0576	0.0403
0.0003	0.0003	0.0003	0.0003	0.0003
0.0003	0.0003	0.0003	0.0003	0.0003
0.0003	0.0003	0.0003	0.0003	0.0003
0.0003	0.0003	0.0003	0.0003	0.0003
0.0003	0.0003	0.0003	0.0003	0.0003
0.0003	0.0003	0.0003	0.0003	0.0003
0.0003	0.0154	0.0003	0.0003	0.0003
0.0003	0.0003	0.0003	0.0003	0.0003
0.0003	0.0003	0.0063	0.0003	0.0003
0.0003	0.0003	0.0003	0.0003	0.0003
0.0003	0.0003	0.0003	0.0003	0.0003
0.0003	0.0003	0.0003	0.0003	0.0003
0.0003	0.0003	0.0003	0.0003	0.0003
0.0003	0.0003	0.0003	0.0003	0.0003
0.0003	0.0003	0.0003	0.0003	0.0003

0.0217	0.0202	0.0003	0.0481	0.0521
0.0003	0.0003	0.0003	0.0003	0.0003
0.0003	0.0003	0.0003	0.0003	0.0003
0.0003	0.0003	0.0003	0.0003	0.0003
0.0003	0.0003	0.0003	0.0003	0.0003
0.0003	0.0003	0.0003	0.0003	0.0003
0.0003	0.0003	0.0003	0.0003	0.0003
0.0998	0.0820	0.0495	0.0356	0.0564

CO 10 VULV POLY IC + AA 1 34	CO 10 VULV POLY IC + AA 2 35	CO 10 VULV POLY IC + AA 3 36	CA 13 VEST AA 1 55	CA 13 VEST AA 2 56
0.0003	0.0003	0.0003	0.0003	0.0003
0.0003	0.0003	0.0060	0.0003	0.0003
0.0003	0.0003	0.0003	0.0003	0.0003
0.4382	0.6177	0.2639	0.0003	0.0003
1.3081	1.1121	1.1276	0.3811	0.3367
0.0003	0.0003	0.0003	0.0003	0.0003
0.0003	0.0308	0.0003	0.0003	0.0003
0.0003	0.0003	0.0003	0.0003	0.0003
0.0003	0.0003	0.0003	0.0003	0.0003
97.1516	102.9519	99.6663	30.1064	29.6813
0.1379	0.1724	0.2804	0.0797	0.0886
4.8096	4.9158	4.0875	1.2453	1.1677
0.0003	0.0003	0.0003	0.0003	0.0003
23.3477	22.5112	23.4933	6.1241	6.3480
0.0003	0.0003	0.0003	0.0003	0.0003
0.0003	0.0003	0.0003	0.0003	0.0003
0.1820	0.1702	0.0939	0.0003	0.0003
0.0003	0.0003	0.0003	0.0003	0.0003
10.4549	9.0561	10.6242	2.6666	2.8119
0.0003	0.0003	0.0003	0.0003	0.0003
0.0818	0.0613	0.1280	0.0366	0.0557
4.5700	5.3967	2.9977	1.8649	1.7876
0.0003	0.0003	0.0003	0.0003	0.0003
0.0003	0.0003	0.0003	0.0003	0.0003
0.0136	0.0124	0.0003	0.0003	0.0003
0.5410	0.5075	0.4432	0.0840	0.0755
6.0880	5.2944	4.9359	1.3371	1.4614
0.0526	0.0561	0.0731	0.0391	0.0461
0.0003	0.0003	0.0003	0.0003	0.0003
0.0003	0.0003	0.0003	0.0003	0.0003
0.0242	0.0003	0.0003	0.0172	0.0003
0.1508	0.1824	0.1244	0.0531	0.0701
12.0062	13.6763	8.9929	0.3843	0.4178

0.0003	0.0003	0.0003	0.0003	0.0003
0.0003	0.0003	0.0003	0.0003	0.0003
0.0870	0.1004	0.0474	0.0753	0.0525
0.0003	0.0365	0.0458	0.0234	0.0003
0.0003	0.0003	0.0003	0.0003	0.0003
0.0003	0.0003	0.0003	0.0003	0.0003
0.0429	0.0260	0.0003	0.0003	0.0003
0.0003	0.0003	0.0003	0.0003	0.0003
0.7357	0.8630	0.9272	0.4694	0.4824
0.0003	0.0086	0.0003	0.0003	0.0003
0.0003	0.0003	0.0003	0.0003	0.0003
0.0461	0.1108	0.0003	0.0052	0.0003
0.0003	0.0003	0.0003	0.0003	0.0003
0.0003	0.0003	0.0003	0.0003	0.0003
0.0003	0.0003	0.0003	0.0003	0.0003
0.0524	0.0271	0.0725	0.0274	0.0003
0.7994	0.7653	0.7768	0.3920	0.4117
0.9091	1.0308	1.1608	0.5702	0.6578
0.0003	0.0205	0.0567	0.0003	0.0003
0.1353	0.1720	0.2898	0.0638	0.1125
4.3289	7.2578	2.3922	2.4194	1.5121
0.0003	0.0003	0.0003	0.0003	0.0003
0.0003	0.0003	0.0003	0.0003	0.0003
0.0003	0.0003	0.0003	0.0003	0.0003
0.0003	0.0003	0.0003	0.0003	0.0003
0.0249	0.0003	0.0436	0.0129	0.0003
0.2045	0.2686	0.7882	0.1907	0.1885
0.0003	0.0003	0.0003	0.0003	0.0003
51.6707	14.8063	157.1554	5.4033	2.1651
23.7261	7.5253	75.6407	4.4822	2.4994
12.4943	3.2491	36.5263	2.2577	1.5940
217.2424	119.1361	363.8810	27.1240	12.4206
93.7023	79.1761	127.7025	25.5790	10.1072
273.8163	172.9817	338.6426	67.9619	24.1632

0.0003	5.3797	0.0003	0.0003	1.0639
25.4958	67.4387	12.3967	59.3657	76.6556
16.2478	17.1775	10.9800	1.0678	0.9553
0.3826	0.1423	0.4458	0.1203	0.0534
0.1442	0.1247	0.2519	0.0217	0.0401
0.0003	0.0003	0.0003	0.0003	0.0003
1.3378	1.2101	1.1886	0.1626	0.0671
0.2980	0.3563	0.2585	0.1681	0.0889
0.5783	0.7178	0.4661	0.2451	0.1656
0.8717	0.9170	0.8859	0.3392	0.1908
0.4951	0.4001	1.2666	0.3002	0.2484
0.0738	0.0565	0.1456	0.0519	0.0367
0.3062	0.1783	0.4110	0.1213	0.1550
0.2599	0.1714	0.4644	0.1528	0.1511
0.2651	0.2055	0.4979	0.1631	0.1656
1.2921	1.4306	2.0812	0.3163	0.2866
0.3419	0.2349	0.5348	0.2047	0.1490
0.5732	0.6073	0.7012	0.2952	0.3108
0.1261	0.1069	0.1969	0.0003	0.0003
0.9812	0.9229	1.4892	0.4374	0.3123
0.0618	0.0393	0.0567	0.0238	0.0522
0.0652	0.0205	0.0225	0.0003	0.0321
0.0707	0.0482	0.0740	0.0291	0.0228
0.0003	0.0003	0.0003	0.0003	0.0003
0.0003	0.0003	0.0003	0.0003	0.0003
0.0003	0.0003	0.0003	0.0003	0.0003
0.0139	0.0003	0.0003	0.0003	0.0003
0.0003	0.0003	0.0003	0.0003	0.0123
0.0513	0.0003	0.0719	0.0266	0.0003
0.0126	0.0003	0.0003	0.0003	0.0003
0.0003	0.0003	0.0080	0.0003	0.0003
0.0118	0.0003	0.0142	0.0057	0.0003
0.0003	0.0003	0.0003	0.0003	0.0003
0.0003	0.0003	0.0003	0.0003	0.0003

0.0126	0.0103	0.0157	0.0003	0.0003
0.0003	0.0108	0.0254	0.0003	0.0062
0.0003	0.0155	0.0188	0.0003	0.0076
0.6094	0.1352	0.9962	0.1439	0.0635
0.1723	0.0865	0.2679	0.0455	0.0143
1.4033	1.2663	1.6850	0.4578	0.2662
2.2815	2.3949	2.6335	1.5167	1.3163
0.0321	0.0231	0.0369	0.0122	0.0099
0.0249	0.0256	0.0265	0.0101	0.0097
0.1042	0.3342	0.4043	0.0987	0.1510
0.6325	0.6028	0.3647	0.1459	0.0003
0.0003	0.0166	0.0003	0.0003	0.0003
0.0003	0.0003	0.0003	0.0003	0.0003
2.9483	1.1046	11.5496	1.2738	0.7772
0.0003	0.0003	0.0003	0.0003	0.0003
0.0003	0.0003	0.0003	0.0003	0.0003
2.0893	2.0070	2.4082	1.1738	1.2790
0.0003	0.0003	0.0003	0.0003	0.0003
0.0494	0.0496	0.0755	0.0419	0.0428
0.0003	0.0070	0.0003	0.0003	0.0047
0.0003	0.0003	0.0003	0.0003	0.0003
0.0003	0.0003	0.0003	0.0003	0.0003
0.0003	0.0003	0.0003	0.0003	0.0003
0.0003	0.0003	0.0003	0.0003	0.0003
0.0003	0.0175	0.0003	0.0003	0.0003
0.0003	0.0003	0.0003	0.0003	0.0003
0.0003	0.0003	0.0003	0.0003	0.0003
0.0048	0.0087	0.0174	0.0003	0.0057
0.0003	0.0003	0.0003	0.0003	0.0003
0.0003	0.0003	0.0003	0.0003	0.0003
0.0003	0.0003	0.0003	0.0003	0.0003
0.0003	0.0003	0.0003	0.0003	0.0003
0.0003	0.0003	0.0003	0.0003	0.0003
0.0003	0.0003	0.0003	0.0003	0.0003

0.0285	0.0371	0.0003	0.0003	0.0457
0.0003	0.0003	0.0003	0.0003	0.0003
0.0003	0.0003	0.0003	0.0003	0.0003
0.0003	0.0003	0.0003	0.0003	0.0003
0.0003	0.0003	0.0003	0.0003	0.0003
0.0003	0.0003	0.0147	0.0003	0.0003
0.0003	0.0003	0.0003	0.0003	0.0003
0.0597	0.0649	0.0855	0.0411	0.0510

CA 13 VEST AA 3 57	CA 13 VULV AA 1 58	CA 13 VULV AA 2 59	CA 13 VULV AA 3 60	CA 13 VEST IL + AA 1 61	CA 13 VEST IL + AA 2 62
0.0003	0.0003	0.0003	0.0003	0.0003	0.0003
0.0003	0.0003	0.0003	0.0074	0.0003	0.0170
0.0003	0.0003	0.0003	0.0003	0.0003	0.0003
0.0003	0.2995	0.4599	0.0003	0.9903	1.2789
0.4160	1.0134	1.4996	1.2072	1.4083	1.7094
0.0003	0.0003	0.0003	0.0003	0.0101	0.0003
0.0175	0.0003	0.0003	0.0353	0.0003	0.0278
0.0003	0.0003	0.0003	0.0003	0.0003	0.0003
0.0003	0.0003	0.0003	0.0003	0.0003	0.0003
34.5257	88.5637	91.7206	104.5226	121.9745	161.2953
0.1135	0.1569	0.2178	0.1503	0.3142	0.3152
1.3855	3.8823	4.2846	4.3631	5.8192	7.5513
0.0003	0.0003	0.0003	0.0003	0.0003	0.0003
5.5981	31.2122	30.4187	32.3906	33.6333	41.1501
0.0003	0.0003	0.0003	0.0003	0.0003	0.0003
0.0003	0.0003	0.0003	0.0003	0.0003	0.0003
0.0003	0.1254	0.1612	0.1413	0.0003	0.2146
0.0003	0.0003	0.0003	0.0003	0.0003	0.0003
2.5941	13.0055	13.4285	14.0664	15.4904	18.0233
0.0003	0.0003	0.0003	0.0003	0.0003	0.0003
0.0531	0.0998	0.0667	0.0871	0.0809	0.0794
1.9864	2.3344	2.6155	2.2956	2.5934	3.3992
0.0666	0.0003	0.0003	0.0003	0.0003	0.0003
0.0003	0.0003	0.0003	0.0003	0.0003	0.0003
0.0003	0.0003	0.0003	0.0003	0.0167	0.0003
0.1069	0.4514	0.4932	0.4611	0.7660	0.8769
1.4492	4.6012	4.8687	4.8722	6.0466	8.0601
0.0343	0.0594	0.0619	0.0699	0.0582	0.0728
0.0003	0.0003	0.0003	0.0003	0.0003	0.0003
0.0003	0.0003	0.0003	0.0003	0.0003	0.0003
0.0003	0.0003	0.0003	0.0416	0.0489	0.0580
0.0835	0.2807	0.3345	0.3092	0.0742	0.0836
0.4368	2.9280	3.4289	2.7119	1.5419	1.7751

0.0003	0.0003	0.0003	0.0003	0.0003	0.0003
0.0003	0.0003	0.0003	0.0003	0.0003	0.0003
0.0003	0.0003	0.0910	0.0712	0.0722	0.1202
0.0261	0.0453	0.0686	0.1011	0.0456	0.0752
0.0003	0.0003	0.0003	0.0003	0.0003	0.0003
0.0003	0.0003	0.0003	0.0003	0.0003	0.0003
0.0003	0.0003	0.0003	0.0003	0.0003	0.0003
0.0003	0.0003	0.0003	0.0003	0.0165	0.0003
0.5020	0.4798	0.5334	0.4582	0.6248	0.6379
0.0003	0.0143	0.0003	0.0003	0.0003	0.0003
0.0003	0.0003	0.0003	0.0003	0.0003	0.0003
0.0106	0.0003	0.0059	0.0003	0.0126	0.0204
0.0003	0.0003	0.0003	0.0003	0.0003	0.0003
0.0003	0.0003	0.0003	0.0003	0.0003	0.0003
0.0003	0.0003	0.0003	0.0003	0.0003	0.0003
0.0184	0.0003	0.0190	0.0003	0.0340	0.0289
0.4282	0.3904	0.3691	0.2737	0.6340	0.6852
0.6373	0.6982	0.7219	0.5826	0.8393	0.8277
0.0003	0.0112	0.0003	0.0003	0.0003	0.0003
0.0003	0.1214	0.1434	0.1152	0.1083	0.4334
1.2952	2.1547	1.3918	1.0958	2.4694	2.6485
0.0003	0.0003	0.0003	0.0003	0.0003	0.0003
0.0003	0.0003	0.0003	0.0003	0.0003	0.0003
0.0003	0.0003	0.0003	0.0003	0.0003	0.0003
0.0003	0.0003	0.0400	0.0461	0.0003	0.0003
0.0106	0.0093	0.0003	0.0003	0.0129	0.0193
0.1891	0.1775	0.2016	0.1384	0.2115	0.2489
0.0003	0.0003	0.0003	0.0003	0.0003	0.0003
2.5252	3.9005	1.9037	1.8747	6.7404	6.5443
2.3961	3.3010	1.3613	1.2972	4.7935	4.4954
1.6790	1.5861	1.1044	1.0517	2.9651	2.9431
12.4045	41.2494	29.4389	30.3020	55.9941	55.2601
9.1660	13.3487	5.5194	3.6023	23.1826	22.1006
23.7599	42.0737	14.1212	10.6102	81.4541	80.1401

1.0974	0.0003	0.0003	0.0003	1.7487	0.0003
91.1711	82.5140	106.9491	99.5124	99.7705	101.3555
0.9438	1.6993	1.9046	1.6981	3.4309	3.6190
0.0894	0.0003	0.0361	0.0517	0.1158	0.0625
0.0003	0.0336	0.0003	0.0295	0.0368	0.0305
0.0003	0.0003	0.0003	0.0003	0.0003	0.0003
0.1051	0.2017	0.1819	0.1534	0.3689	0.3442
0.0991	0.0923	0.0513	0.0481	0.1670	0.1600
0.1674	0.1784	0.1356	0.1761	0.5164	0.5409
0.2180	0.2090	0.1685	0.1146	0.2975	0.2704
0.2902	0.3041	0.2759	0.2568	0.3843	0.4754
0.0408	0.0355	0.0428	0.0317	0.0528	0.0735
0.1681	0.1035	0.0991	0.1023	0.2058	0.2194
0.1460	0.1772	0.1650	0.1255	0.1311	0.1671
0.1390	0.1631	0.1393	0.1246	0.2402	0.2341
0.2779	0.6833	0.7047	0.7327	0.5461	0.6694
0.1415	0.1528	0.1582	0.1471	0.1947	0.2600
0.2985	0.3609	0.4072	0.3838	0.5031	0.4842
0.0003	0.0003	0.0428	0.0003	0.0837	0.1345
0.3941	0.4322	0.3625	0.3238	0.5520	0.4859
0.0431	0.0337	0.0497	0.0273	0.0287	0.1523
0.0003	0.0115	0.0230	0.0003	0.0138	0.0627
0.0330	0.0320	0.0396	0.0291	0.0328	0.0374
0.0003	0.0003	0.0003	0.0003	0.0003	0.0003
0.0003	0.0003	0.0003	0.0003	0.0003	0.0003
0.0003	0.0003	0.0003	0.0003	0.0003	0.0003
0.0003	0.0003	0.0003	0.0003	0.0003	0.0003
0.0003	0.0003	0.0003	0.0003	0.0003	0.0003
0.0003	0.0389	0.0003	0.0180	0.0375	0.0271
0.0003	0.0003	0.0003	0.0003	0.0003	0.0003
0.0003	0.0003	0.0003	0.0003	0.0053	0.0003
0.0003	0.0003	0.0056	0.0003	0.0003	0.0063
0.0003	0.0003	0.0003	0.0003	0.0003	0.0003
0.0003	0.0003	0.0003	0.0003	0.0003	0.0003

0.0003	0.0003	0.0003	0.0003	0.0003	0.0003
0.0003	0.0003	0.0063	0.0003	0.0092	0.0003
0.0003	0.0003	0.0059	0.0003	0.0071	0.0003
0.0609	0.1286	0.0411	0.0291	0.1438	0.0442
0.0130	0.0214	0.0097	0.0003	0.0327	0.0242
0.3277	0.3613	0.1906	0.1286	0.4019	0.4124
1.7141	1.7603	1.8686	1.3282	1.9756	2.1985
0.0003	0.0003	0.0003	0.0003	0.0136	0.0163
0.0113	0.0116	0.0130	0.0142	0.0165	0.0162
0.1278	0.0548	0.0701	0.0635	0.1112	0.6085
0.1428	0.0003	0.1200	0.0911	0.3002	0.5377
0.0003	0.0239	0.0139	0.0117	0.0210	0.0003
0.0003	0.0003	0.0003	0.0003	0.0003	0.0003
0.7427	0.9994	0.6357	0.7095	1.5102	1.4356
0.0003	0.0003	0.0003	0.0003	0.0003	0.0003
0.0003	0.0003	0.0003	0.0003	0.0003	0.0003
1.4502	1.7041	1.7066	1.4343	1.6724	1.9912
0.0003	0.0003	0.0003	0.0003	0.0003	0.0003
0.0314	0.0447	0.0480	0.0448	0.0791	0.0604
0.0003	0.0003	0.0003	0.0003	0.0003	0.0003
0.0003	0.0003	0.0003	0.0003	0.0003	0.0003
0.0003	0.0003	0.0003	0.0003	0.0003	0.0003
0.0003	0.0003	0.0003	0.0003	0.0003	0.0003
0.0003	0.0003	0.0003	0.0003	0.0003	0.0003
0.0003	0.0003	0.0003	0.0003	0.0003	0.0003
0.0003	0.0003	0.0003	0.0003	0.0003	0.0003
0.0003	0.0003	0.0003	0.0003	0.0003	0.0003
0.0045	0.0003	0.0101	0.0003	0.0003	0.0003
0.0003	0.0003	0.0003	0.0003	0.0003	0.0003
0.0003	0.0003	0.0003	0.0003	0.0003	0.0003
0.0003	0.0003	0.0003	0.0003	0.0003	0.0003
0.0003	0.0003	0.0003	0.0003	0.0003	0.0003
0.0003	0.0003	0.0003	0.0003	0.0003	0.0003
0.0003	0.0003	0.0003	0.0003	0.0003	0.0003

0.0003	0.0337	0.0003	0.0003	0.0003	0.0003
0.0003	0.0003	0.0003	0.0003	0.0003	0.0003
0.0003	0.0003	0.0003	0.0003	0.0003	0.0003
0.0003	0.0003	0.0003	0.0003	0.0003	0.0003
0.0003	0.0003	0.0003	0.0003	0.0003	0.0003
0.0003	0.0003	0.0003	0.0003	0.0003	0.0003
0.0003	0.0003	0.0003	0.0003	0.0003	0.0003
0.0570	0.0532	0.0386	0.0686	0.0428	0.0285

CA 13 VEST IL + AA 3 63	CA 13 VULV IL + AA 1 64	CA 13 VULV IL + AA 2 65	CA 13 VULV IL + AA 3 66	CA 13 VEST POLY IC + AA 1 67
0.0003	0.0003	0.0003	0.0003	0.0003
0.0003	0.0618	0.0387	0.0436	0.0118
0.0003	0.0003	0.0003	0.0003	0.0003
1.1408	4.3976	3.7701	4.1439	0.9855
1.6008	3.3236	3.0065	2.5904	1.4040
0.0003	0.0123	0.0003	0.0003	0.0003
0.0003	0.1107	0.0003	0.1032	0.0003
0.0003	0.0003	0.0003	0.0003	0.0003
0.0003	0.0003	0.0115	0.0149	0.0003
120.8351	313.4432	272.6836	373.2044	104.1939
0.2707	1.1809	0.8237	0.9439	0.1981
6.2896	14.6648	12.3857	13.2258	5.2323
0.0003	0.0003	0.0003	0.0003	0.0003
34.0257	91.5744	79.6639	76.0890	26.0402
0.0003	0.0003	0.0003	0.0003	0.0003
0.0003	0.0003	0.0003	0.0003	0.0003
0.1907	0.7075	0.7087	0.9661	0.1776
0.0003	0.0003	0.0003	0.0003	0.0003
13.9383	45.4200	37.9506	34.4917	11.4228
0.0003	0.0003	0.0003	0.0003	0.0003
0.0734	0.1348	0.0998	0.1020	0.0868
2.9495	5.8746	2.7547	3.1194	3.3495
0.0003	0.2190	0.0003	0.0003	0.0003
0.0003	0.0003	0.0003	0.0003	0.0003
0.0208	0.0864	0.0757	0.0953	0.0156
0.8470	3.4160	2.8318	3.9134	0.5012
6.4732	16.8576	15.6990	15.8891	6.8096
0.0679	0.1009	0.0836	0.0866	0.0553
0.0003	0.0003	0.0003	0.0003	0.0003
0.0003	0.0003	0.0003	0.0003	0.0003
0.0326	0.0003	0.1677	0.2163	0.0318
0.0003	0.3825	0.3883	0.3864	0.0739
1.5331	8.7572	7.1134	7.3906	1.3899

0.0003	0.0003	0.0003	0.0003	0.0003
0.0003	0.0003	0.0003	0.0003	0.0003
0.1091	0.0003	0.0769	0.1323	0.0557
0.1120	0.3067	0.3000	0.2718	0.0465
0.0003	0.0003	0.0003	0.0003	0.0003
0.0003	0.0003	0.0003	0.0003	0.0003
0.0003	0.0003	0.0003	0.0003	0.0003
0.0003	0.0003	0.0003	0.0003	0.0003
0.7443	0.2801	0.2004	0.2519	0.6210
0.0003	0.0003	0.0003	0.0003	0.0003
0.0003	0.0003	0.0003	0.0083	0.0003
0.0055	0.0003	0.0003	0.0387	0.0099
0.0003	0.0003	0.0003	0.0003	0.0003
0.0003	0.0003	0.0003	0.0003	0.0003
0.0003	0.0003	0.0003	0.0003	0.0003
0.0337	0.0003	0.0003	0.0095	0.0393
0.6410	0.2515	0.1697	0.2243	0.6895
0.9446	0.3517	0.2423	0.3408	0.8462
0.0003	0.0003	0.0003	0.0003	0.0003
0.1368	0.0003	0.0003	0.0003	0.1331
1.6616	1.2392	0.6280	1.1566	3.7242
0.0003	0.0003	0.0003	0.0003	0.0003
0.0003	0.0003	0.0003	0.0003	0.0003
0.0003	0.0003	0.0003	0.0003	0.0003
0.0003	0.0657	0.0983	0.0627	0.0217
0.0003	0.0003	0.0003	0.0115	0.0111
0.1624	0.1148	0.1257	0.1286	0.2417
0.0003	0.0003	0.0003	0.0003	0.0003
3.3655	1.7095	1.4470	1.6975	21.0734
2.6133	0.7708	0.8367	0.7422	13.0299
1.8765	0.5571	0.7369	0.6132	5.0717
37.8409	83.4643	72.5515	75.4540	113.6065
7.7070	1.3494	1.3928	1.3486	48.7518
31.4115	21.6751	20.7820	19.3153	159.3089

0.0003	0.0003	0.0003	0.0003	2.4723
110.9448	92.5075	71.9397	86.5908	68.7107
2.1617	1.3912	1.1904	0.7624	4.0832
0.0818	0.0003	0.0003	0.0003	0.2105
0.0255	0.0003	0.0003	0.0003	0.0617
0.0003	0.0003	0.0003	0.0003	0.0003
0.1333	0.5236	0.4988	0.5535	0.5868
0.0896	0.0003	0.0003	0.0003	0.2519
0.3593	0.2143	0.1152	0.1054	0.4293
0.1973	0.0652	0.0532	0.0774	0.3726
0.3631	0.1938	0.2190	0.2099	0.3800
0.0491	0.0321	0.0003	0.0250	0.0609
0.1871	0.0721	0.0888	0.0563	0.1467
0.1833	0.1148	0.1241	0.0984	0.1668
0.1867	0.1090	0.1280	0.0836	0.1709
0.4662	1.5790	1.6531	1.6731	0.8497
0.1154	0.0913	0.1021	0.1260	0.2815
0.4170	0.2326	0.2331	0.1600	0.4307
0.0458	0.0003	0.0003	0.0003	0.0700
0.3401	0.2383	0.2189	0.2224	0.4415
0.0532	0.0493	0.0496	0.0475	0.0466
0.0261	0.0003	0.0256	0.0145	0.0136
0.0300	0.0347	0.0274	0.0244	0.0298
0.0003	0.0003	0.0003	0.0003	0.0003
0.0176	0.0003	0.0003	0.0003	0.0003
0.0003	0.0003	0.0256	0.0003	0.0003
0.0003	0.0003	0.0003	0.0003	0.0003
0.0180	0.0003	0.0003	0.0003	0.0003
0.0003	0.0003	0.0003	0.0003	0.0003
0.0003	0.0003	0.0003	0.0003	0.0003
0.0003	0.0003	0.0003	0.0003	0.0003
0.0003	0.0071	0.0091	0.0003	0.0003
0.0003	0.0054	0.0003	0.0003	0.0003
0.0003	0.0003	0.0003	0.0003	0.0003

0.0003	0.0003	0.0003	0.0003	0.0003
0.0003	0.0072	0.0003	0.0003	0.0093
0.0067	0.0003	0.0003	0.0003	0.0057
0.0270	0.0280	0.0153	0.0144	0.2307
0.0133	0.0003	0.0003	0.0003	0.0546
0.3030	0.0564	0.0429	0.0761	0.7251
2.3104	0.9296	0.7025	1.0761	1.6715
0.0169	0.0003	0.0003	0.0003	0.0264
0.0242	0.0003	0.0100	0.0122	0.0099
0.4032	0.0915	0.1089	0.0837	0.0672
0.5317	0.1563	0.2164	0.1918	0.3551
0.0355	0.0202	0.0003	0.0003	0.0121
0.0003	0.0003	0.0003	0.0003	0.0003
1.1905	0.7446	0.6250	0.5480	2.4468
0.0003	0.0003	0.0003	0.0003	0.0003
0.0003	0.4017	0.5874	0.0003	0.0003
2.0015	1.2263	1.3996	2.1442	1.8571
0.0003	0.0003	0.0003	0.0003	0.0003
0.0627	0.0613	0.0486	0.0469	0.0660
0.0003	0.0003	0.0003	0.0003	0.0003
0.0003	0.0003	0.0003	0.0003	0.0003
0.0003	0.0003	0.0003	0.0003	0.0391
0.0003	0.0003	0.0003	0.0003	0.0003
0.0003	0.0003	0.0003	0.0003	0.0003
0.0232	0.0264	0.0003	0.0281	0.0003
0.0003	0.0003	0.0003	0.0003	0.0003
0.0003	0.0003	0.0003	0.0003	0.0003
0.0003	0.0003	0.0003	0.0003	0.0003
0.0003	0.0003	0.0003	0.0003	0.0003
0.0003	0.0003	0.0003	0.0003	0.0003
0.0003	0.0003	0.0003	0.0003	0.0003
0.0003	0.0003	0.0003	0.0003	0.0003
0.0003	0.0003	0.0003	0.0003	0.0003
0.0003	0.0003	0.0003	0.0003	0.0003

0.0372	0.0480	0.0433	0.0409	0.0003
0.0003	0.0003	0.0003	0.0003	0.0003
0.0003	0.0003	0.0003	0.0003	0.0003
0.0003	0.0003	0.0003	0.0003	0.0003
0.0003	0.0003	0.0003	0.0003	0.0003
0.0003	0.0003	0.0003	0.0003	0.0003
0.0003	0.0003	0.0003	0.0003	0.0003
0.0701	0.0344	0.0433	0.0145	0.0603

CA 13 VEST POLY IC + AA 2 68	CA 13 VEST POLY IC + AA 3 69	CA 13 VULV POLY IC + AA 1 70	CA 13 VULV POLY IC + AA 2 71
0.0003	0.0003	0.0003	0.0003
0.0003	0.0003	0.0268	0.0254
0.0003	0.0003	0.0003	0.0003
2.2107	2.2315	1.7631	1.8418
1.7478	1.5452	2.4839	2.2172
0.0003	0.0003	0.0003	0.0003
0.0003	0.0003	0.0003	0.0549
0.0003	0.0003	0.0003	0.0003
0.0003	0.0003	0.0003	0.0003
121.1610	137.6854	192.0152	189.3788
0.3838	0.2671	0.4764	0.4995
7.9191	6.8391	11.1779	9.7604
0.0003	0.0003	0.0003	0.0003
34.7161	28.3240	58.4312	61.1373
0.0003	0.0003	0.0003	0.0003
0.0003	0.0003	0.0003	0.0003
0.2681	0.2760	0.4577	0.4967
0.0003	0.0003	0.0003	0.0003
14.8455	12.1531	29.4250	28.4060
0.0003	0.0003	0.0003	0.0003
0.0771	0.0535	0.0985	0.0847
2.7486	2.2995	4.1608	2.7231
0.0003	0.0837	0.0003	0.0003
0.0003	0.0003	0.0003	0.0003
0.0003	0.0326	0.0403	0.0364
0.7807	0.7199	1.7519	1.8259
8.5963	7.9943	12.6390	11.2486
0.0727	0.0507	0.0741	0.0664
0.0003	0.0003	0.0003	0.0003
0.0003	0.0003	0.0003	0.0003
0.0557	0.0429	0.1057	0.1050
0.0815	0.0003	0.2869	0.2459
2.2166	1.5980	7.7619	8.0883

0.0003	0.0003	0.0003	0.0003
0.0003	0.0003	0.0003	0.0003
0.0583	0.0561	0.1067	0.1025
0.1540	0.1178	0.1054	0.0003
0.0003	0.0003	0.0003	0.0003
0.0093	0.0003	0.0065	0.0003
0.0003	0.0003	0.0003	0.0003
0.0003	0.0003	0.0003	0.0003
0.7531	0.6024	0.5381	0.5041
0.0059	0.0003	0.0003	0.0003
0.0003	0.0003	0.0003	0.0003
0.0003	0.0003	0.0154	0.0181
0.0003	0.0003	0.0003	0.0003
0.0003	0.0003	0.0003	0.0003
0.0003	0.0003	0.0003	0.0003
0.0003	0.0271	0.0111	0.0211
0.6523	0.5827	0.5807	0.4091
0.8930	0.8266	0.7798	0.7647
0.0003	0.0003	0.0003	0.0003
0.0003	0.1328	0.1004	0.1889
2.4266	2.0107	3.3729	1.9310
0.0003	0.0003	0.0003	0.0003
0.0003	0.0003	0.0003	0.0003
0.0003	0.0003	0.0003	0.0003
0.0003	0.0202	0.0003	0.0441
0.0003	0.0003	0.0003	0.0143
0.0918	0.0992	0.1292	0.1056
0.0003	0.0003	0.0003	0.0003
3.6883	3.7376	2.2914	1.8206
2.2127	1.6567	0.9090	0.7861
1.3692	1.0804	0.7332	0.7075
53.9649	59.4777	38.2600	54.3885
9.4604	8.8305	4.5376	2.4917
46.3114	47.1575	22.2290	16.8291

0.0003	0.0003	0.0003	0.0003
114.8663	101.9397	109.1483	119.5393
3.4474	3.0299	4.1275	3.5112
0.0757	0.0789	0.0867	0.0593
0.0003	0.0366	0.0003	0.0273
0.0003	0.0003	0.0003	0.0003
0.3673	0.4140	0.2894	0.3446
0.1312	0.0706	0.1023	0.0003
0.4105	0.4538	0.4011	0.2453
0.2315	0.1875	0.1433	0.1113
0.2238	0.2664	0.1996	0.2338
0.0309	0.0463	0.0187	0.0295
0.1230	0.1126	0.0613	0.0615
0.0924	0.0966	0.0824	0.1284
0.1385	0.1374	0.1340	0.1058
0.8412	0.7287	0.7750	1.2048
0.1985	0.1844	0.1300	0.1384
0.3166	0.3587	0.3006	0.2905
0.0003	0.0003	0.0003	0.0003
0.2526	0.2781	0.2313	0.2822
0.0369	0.0313	0.0425	0.0003
0.0146	0.0003	0.0326	0.0209
0.0200	0.0229	0.0236	0.0235
0.0003	0.0003	0.0003	0.0003
0.0003	0.0003	0.0003	0.0003
0.0003	0.0003	0.0003	0.0003
0.0003	0.0003	0.0003	0.0003
0.0199	0.0003	0.0003	0.0003
0.0003	0.0192	0.0229	0.0003
0.0003	0.0003	0.0003	0.0003
0.0033	0.0003	0.0003	0.0003
0.0048	0.0003	0.0003	0.0074
0.0003	0.0067	0.0003	0.0003
0.0003	0.0003	0.0003	0.0003

0.0035	0.0003	0.0003	0.0003
0.0003	0.0053	0.0080	0.0053
0.0003	0.0054	0.0003	0.0003
0.0722	0.0644	0.0426	0.0258
0.0123	0.0186	0.0120	0.0003
0.3111	0.2852	0.2120	0.1500
1.6979	1.7610	1.8111	1.8334
0.0003	0.0138	0.0003	0.0003
0.0099	0.0126	0.0150	0.0162
0.1705	0.0916	0.2005	0.2460
0.1855	0.1149	0.2539	0.1129
0.0212	0.0003	0.0003	0.0003
0.0003	0.0003	0.0003	0.0003
0.8846	0.6773	0.4972	0.5501
0.0003	0.0003	0.0003	0.0003
0.9241	0.0003	0.0003	0.0003
2.1767	1.6264	1.8410	1.9358
0.0003	0.0003	0.0003	0.0003
0.0453	0.0432	0.0003	0.0462
0.0003	0.0003	0.0003	0.0003
0.0003	0.0003	0.0003	0.0003
0.0003	0.0003	0.0003	0.0003
0.0003	0.0003	0.0003	0.0003
0.0003	0.0003	0.0003	0.0003
0.0003	0.0260	0.0003	0.0363
0.0003	0.0003	0.0003	0.0003
0.0003	0.0003	0.0003	0.0003
0.0003	0.0083	0.0003	0.0090
0.0003	0.0003	0.0003	0.0003
0.0003	0.0003	0.0003	0.0003
0.0003	0.0003	0.0003	0.0003
0.0003	0.0003	0.0003	0.0003
0.0003	0.0003	0.0003	0.0003
0.0003	0.0003	0.0003	0.0003

0.0437	0.0003	0.0308	0.0346
0.0003	0.0003	0.0003	0.0003
0.0003	0.0003	0.0003	0.0003
0.0003	0.0003	0.0003	0.0003
0.0003	0.0003	0.0003	0.0003
0.0003	0.0003	0.0003	0.0003
0.0003	0.0003	0.0003	0.0003
0.0594	0.0451	0.0359	0.0579

CA 13 VULV POLY IC + AA 3 72	CA 14 VEST AA 1 91	CA 14 VEST AA 2 92	CA 14 VEST AA 3 93	CA 14 VULV AA 1 94	CA 14 VULV AA 2 95
0.0003	0.0003	0.0003	0.0003	0.0003	0.0003
0.0341	0.0003	0.0003	0.0003	0.0003	0.0003
0.0003	0.0003	0.0003	0.0003	0.0003	0.0003
2.5740	0.0003	0.1320	0.1238	0.0003	0.0003
2.9191	0.5380	0.5955	0.5743	0.4089	0.4161
0.0057	0.0003	0.0003	0.0003	0.0003	0.0003
0.0003	0.0003	0.0003	0.0003	0.0003	0.0003
0.0003	0.0003	0.0003	0.0003	0.0003	0.0003
0.0143	0.0003	0.0003	0.0003	0.0003	0.0003
349.3755	39.7671	45.9414	42.7662	29.7449	33.8308
0.4439	0.0547	0.0534	0.0548	0.0895	0.0610
11.5664	1.7799	1.8076	1.6139	1.3167	1.3791
11.6376	0.0003	0.0003	0.0003	0.7230	0.0003
72.2064	7.0464	8.1178	4.8661	6.8183	7.3242
0.0003	0.0003	0.0003	0.0003	0.0003	0.0003
0.0003	0.0003	0.0003	0.0003	0.0003	0.0003
0.6819	0.0003	0.0578	0.0764	0.0003	0.0003
0.0003	0.0003	0.0003	0.0003	0.0003	0.0003
36.0844	3.1001	3.3874	2.3144	3.1616	3.4741
0.0003	0.0003	0.0003	0.0003	0.0003	0.0003
0.1182	0.0527	0.0605	0.0526	0.0465	0.0435
3.1954	2.2521	1.9201	2.2415	2.1601	2.2745
0.0003	0.0003	0.0003	0.0003	0.0003	0.0003
0.0003	0.0003	0.0003	0.0003	0.0003	0.0003
0.0460	0.0003	0.0003	0.0003	0.0003	0.0003
2.4124	0.1895	0.2058	0.2086	0.1557	0.1420
13.4006	1.7980	2.0140	1.6975	1.3767	1.3032
0.0862	0.0550	0.0382	0.0502	0.0339	0.0452
0.0003	0.0003	0.0003	0.0003	0.0003	0.0003
0.0003	0.0003	0.0003	0.0003	0.0003	0.0003
0.1337	0.0195	0.0003	0.0383	0.0003	0.0136
0.2550	0.1608	0.1620	0.1578	0.0584	0.0835
9.4103	0.4756	0.4963	0.4406	2.3000	2.3696

0.0003	0.0003	0.0003	0.0003	0.0003	0.0003
0.0003	0.0003	0.0003	0.0003	0.0003	0.0003
0.1404	0.0791	0.0740	0.0003	0.0649	0.0456
0.1606	0.0003	0.0003	0.0003	0.0225	0.0003
0.0003	0.0003	0.0003	0.0003	0.0003	0.0003
0.0003	0.0003	0.0003	0.0003	0.0003	0.0003
0.0003	0.0003	0.0003	0.0003	0.0003	0.0003
0.0003	0.0003	0.0003	0.0003	0.0003	0.0003
0.5046	0.6614	0.5768	0.5260	0.5383	0.6298
0.0003	0.0003	0.0195	0.0003	0.0003	0.0094
0.0003	0.0003	0.0003	0.0003	0.0003	0.0003
0.0003	0.0003	0.0003	0.0003	0.0003	0.0003
0.0003	0.0003	0.0003	0.0003	0.0003	0.0003
0.0003	0.0003	0.0003	0.0003	0.0003	0.0003
0.0003	0.0003	0.0003	0.0003	0.0003	0.0003
0.0003	0.0091	0.0185	0.0111	0.0200	0.0003
0.4168	0.6278	0.4855	0.3983	0.4917	0.5536
0.7728	0.8210	1.5710	0.7415	1.5243	0.9767
0.0003	0.0003	0.0003	0.0003	0.0003	0.0003
0.0003	0.1043	0.1034	0.0964	0.1333	0.1749
1.4915	2.3968	3.7780	2.5875	3.6353	1.7509
0.0003	0.0003	0.0003	0.0003	0.0003	0.0003
0.0310	0.0003	0.0208	0.0003	0.0003	0.0003
0.0003	0.0003	0.0003	0.0003	0.0003	0.0003
0.0617	0.0003	0.0003	0.0003	0.0003	0.0003
0.0003	0.0003	0.0003	0.0091	0.0328	0.0349
0.0791	0.1917	0.2517	0.2179	0.3196	0.4580
0.0003	0.0003	0.0003	0.0003	0.0003	0.0003
1.4806	4.1335	3.0279	2.8726	67.8047	105.1964
0.6024	2.3162	1.8689	1.4820	0.0003	55.3224
0.5253	1.1090	1.1844	0.9719	17.4872	24.7426
47.7604	20.0501	17.5126	14.1222	158.1738	237.7853
1.9133	18.2721	15.6060	8.7438	82.4200	110.0994
13.6251	47.3890	29.9396	16.5074	215.8396	277.0388

0.0003	1.5309	1.6782	0.0003	0.0003	0.0003
117.5572	74.8176	87.5341	93.5474	10.1947	11.1636
2.6830	0.9687	1.0543	0.7506	4.0465	4.5377
0.0003	0.0723	0.0753	0.0570	0.2678	0.2849
0.0217	0.0423	0.0484	0.0262	0.0904	0.1556
0.0003	0.0003	0.0003	0.0003	0.0003	0.0003
0.4010	0.2303	0.1852	0.2141	0.2441	0.2567
0.0575	0.2398	0.0003	0.1856	0.0003	0.1438
0.2990	0.3801	0.3874	0.4024	0.1864	0.1351
0.1410	0.4812	0.4556	0.3982	0.5006	0.5664
0.0003	0.5708	0.5073	0.5240	0.3926	0.4621
0.0003	0.0881	0.1035	0.0651	0.0300	0.0552
0.0627	0.2857	0.3049	0.2856	0.1245	0.1768
0.0830	0.2230	0.2159	0.2642	0.1179	0.1869
0.0897	0.1978	0.2270	0.2509	0.1666	0.2507
1.4026	0.6220	0.5321	0.5254	0.4431	0.5863
0.1054	0.2469	0.0003	0.2171	0.1987	0.2716
0.2948	0.3960	0.3636	0.4340	0.2041	0.2195
0.0637	0.0003	0.0687	0.0003	0.0003	0.0740
0.2088	0.7347	0.7066	0.6734	0.4344	0.5902
0.0476	0.0723	0.0416	0.0346	0.0271	0.0452
0.0132	0.0419	0.0003	0.0162	0.0003	0.0193
0.0214	0.0725	0.0548	0.0611	0.0403	0.0407
0.0003	0.0003	0.0003	0.0003	0.0003	0.0003
0.0003	0.0003	0.0003	0.0003	0.0003	0.0003
0.0003	0.0003	0.0003	0.0003	0.0003	0.0003
0.0003	0.0003	0.0003	0.0003	0.0003	0.0003
0.0003	0.0003	0.0189	0.0003	0.0003	0.0446
0.0003	0.0385	0.0245	0.0003	0.1253	0.1514
0.0003	0.0003	0.0003	0.0003	0.0003	0.0003
0.0003	0.0003	0.0041	0.0033	0.0003	0.0003
0.0003	0.0135	0.0103	0.0123	0.0003	0.0029
0.0003	0.0003	0.0003	0.0003	0.0003	0.0003
0.0003	0.0003	0.0003	0.0003	0.0003	0.0003

0.0003	0.0003	0.0003	0.0153	0.0003	0.0061
0.0055	0.0069	0.0003	0.0003	0.0063	0.0003
0.0003	0.0003	0.0060	0.0082	0.0003	0.0059
0.0222	0.1517	0.1080	0.0591	0.2348	0.5746
0.0003	0.0338	0.0174	0.0162	0.1493	0.1821
0.1113	0.6009	0.4237	0.3380	1.1204	1.4149
1.5655	1.8505	1.7543	1.8092	1.7734	2.0115
0.0003	0.0174	0.0003	0.0102	0.0290	0.0138
0.0177	0.0351	0.0262	0.0211	0.0101	0.0106
0.1085	0.1841	0.1968	0.1443	0.0003	0.0864
0.1500	0.1872	0.1715	0.0003	0.3248	0.3996
0.0003	0.0003	0.0003	0.0003	0.0003	0.0003
0.0003	0.0003	0.0003	0.0003	0.0003	0.0003
0.4933	1.2776	1.1550	1.0084	5.2299	6.6031
0.0003	0.0003	0.0003	0.0003	0.0003	0.0003
0.3332	0.0003	0.0003	0.0003	0.2819	0.0003
1.8636	1.7618	1.5492	1.4372	1.6381	1.8052
0.0003	0.0003	0.0003	0.0003	0.0003	0.0003
0.0003	0.0432	0.0466	0.0273	0.0379	0.0428
0.0003	0.0003	0.0003	0.0003	0.0003	0.0003
0.0003	0.0003	0.0003	0.0003	0.0003	0.0003
0.0003	0.0003	0.0003	0.0003	0.0003	0.0003
0.0003	0.0003	0.0003	0.0003	0.0003	0.0003
0.0003	0.0003	0.0003	0.0003	0.0003	0.0003
0.0003	0.0003	0.0003	0.0150	0.0164	0.0003
0.0003	0.0003	0.0003	0.0003	0.0003	0.0003
0.0003	0.0003	0.0003	0.0003	0.0003	0.0003
0.0003	0.0099	0.0052	0.0128	0.0056	0.0003
0.0003	0.0003	0.0003	0.0003	0.0003	0.0003
0.0003	0.0003	0.0003	0.0003	0.0003	0.0003
0.0003	0.0003	0.0077	0.0003	0.0003	0.0003
0.0003	0.0003	0.0003	0.0003	0.0003	0.0003
0.0003	0.0003	0.0003	0.0003	0.0003	0.0003
0.0003	0.0003	0.0003	0.0003	0.0003	0.0003

0.0436	0.0003	0.0326	0.0003	0.0003	0.0003
0.0003	0.0003	0.0003	0.0003	0.0003	0.0003
0.0003	0.0003	0.0003	0.0003	0.0003	0.0003
0.0003	0.0003	0.0003	0.0003	0.0003	0.0003
0.0003	0.0003	0.0003	0.0003	0.0003	0.0003
0.0003	0.0003	0.0003	0.0003	0.0003	0.0003
0.0003	0.0003	0.0003	0.0003	0.0003	0.0003
0.0591	0.1048	0.1018	0.1328	0.0003	0.0327

CA 14 VULV AA 3 96	CA 14 VEST IL + AA 1 97	CA 14 VEST IL + AA 2 98	CA 14 VEST IL + AA 3 99	CA 14 VULV IL + AA 1 100
0.0003	0.0003	0.0003	0.0003	0.0003
0.0003	0.0139	0.0113	0.0125	0.0003
0.0003	0.0003	0.0003	0.0003	0.0003
0.0003	1.2648	1.0978	0.9702	0.0003
0.3963	1.7374	1.3339	1.4410	1.2449
0.0003	0.0003	0.0003	0.0003	0.0003
0.0003	0.0003	0.0339	0.0003	0.0287
0.0003	0.0003	0.0003	0.0003	0.0003
0.0003	0.0003	0.0003	0.0003	0.0003
33.4917	147.2521	116.5511	118.5958	95.4438
0.0709	0.1716	0.1736	0.1727	0.1423
1.4206	7.3620	6.1212	6.1217	4.3557
0.0003	0.0003	0.0003	3.1566	3.5781
6.7759	33.6194	27.9315	29.0365	26.2038
0.0003	0.0003	0.0003	0.0003	0.0003
0.0003	0.0003	0.0003	0.0003	0.0003
0.0003	0.0003	0.1848	0.1456	0.0003
0.0003	0.0003	0.0003	0.0003	0.0003
3.3167	13.6091	11.7892	12.0155	12.5799
0.0003	0.0003	0.0003	0.0003	0.0003
0.0445	0.0942	0.0549	0.0589	0.0781
2.2969	3.2484	2.1171	2.4622	2.4944
0.0003	0.0003	0.0003	0.0003	0.0003
0.0003	0.0003	0.0003	0.0003	0.0003
0.0003	0.0257	0.0220	0.0279	0.0003
0.1819	1.0866	0.9553	0.9571	0.5666
1.2021	7.9222	6.9120	7.1567	5.1184
0.0389	0.0530	0.0409	0.0554	0.0439
0.0003	0.0003	0.0003	0.0003	0.0003
0.0003	0.0003	0.0003	0.0003	0.0003
0.0003	0.0003	0.0003	0.0003	0.0003
0.0755	0.1374	0.1040	0.1272	0.1090
2.2064	1.2759	1.1803	1.4268	4.8784

0.0003	0.0003	0.0003	0.0003	0.0003
0.0003	0.0003	0.0003	0.0003	0.0003
0.0357	0.0003	0.0729	0.1019	0.0637
0.0286	0.0003	0.0003	0.0314	0.0003
0.0003	0.0003	0.0003	0.0003	0.0003
0.0003	0.0003	0.0003	0.0003	0.0003
0.0003	0.0003	0.0003	0.0003	0.0003
0.0003	0.0003	0.0003	0.0003	0.0003
0.5254	0.6068	0.5018	0.5409	0.6133
0.0003	0.0128	0.0003	0.0071	0.0003
0.0003	0.0003	0.0003	0.0003	0.0003
0.0052	0.0003	0.0003	0.0090	0.0003
0.0003	0.0003	0.0003	0.0003	0.0003
0.0003	0.0003	0.0003	0.0003	0.0003
0.0003	0.0003	0.0003	0.0003	0.0003
0.0003	0.0207	0.0184	0.0180	0.0448
0.4374	0.4181	0.4735	0.5934	0.5493
1.5131	0.8151	0.6824	0.8365	0.7362
0.0003	0.0003	0.0003	0.0003	0.0003
0.1227	0.0003	0.1007	0.1992	0.1265
1.9176	4.2670	2.4748	2.5842	4.6853
0.0003	0.0003	0.0003	0.0003	0.0003
0.0003	0.0003	0.0003	0.0003	0.0003
0.0003	0.0003	0.0003	0.0003	0.0003
0.0003	0.0280	0.0003	0.0003	0.0177
0.0175	0.0003	0.0071	0.0003	0.0180
0.2579	0.1890	0.1393	0.1733	0.1573
0.0003	0.0003	0.0003	0.0003	0.0003
55.5052	4.1600	3.4044	3.4936	17.0447
30.8973	1.8430	1.4721	1.5522	13.8284
14.9269	1.0746	0.9373	1.1067	4.7359
147.8861	61.9230	46.0744	44.9516	153.3925
87.8856	15.3682	12.1539	11.2871	87.8762
220.8866	59.3977	48.9172	42.2438	208.7813

0.0003	0.0003	1.1743	0.0003	0.0003
12.7957	112.4244	79.3508	112.6060	31.3905
4.6840	3.1005	2.1652	3.2197	8.8007
0.2371	0.0836	0.0624	0.0370	0.1467
0.1527	0.0310	0.0003	0.0420	0.0822
0.0003	0.0003	0.0003	0.0003	0.0003
0.1833	0.6469	0.4038	0.4813	0.6075
0.1279	0.2167	0.1807	0.2245	0.1487
0.1599	0.7060	0.5750	0.6826	0.2334
0.5167	0.3894	0.2571	0.3799	0.5275
0.3415	0.5280	0.4683	0.5334	0.2122
0.0407	0.0763	0.0680	0.0827	0.0376
0.1963	0.2361	0.2104	0.2367	0.1364
0.1567	0.2003	0.1754	0.2035	0.0998
0.1640	0.2382	0.2248	0.1939	0.1495
0.3914	0.9872	0.8355	0.7376	0.6078
0.2170	0.2453	0.2499	0.2238	0.1280
0.2545	0.4835	0.3618	0.4382	0.2152
0.0003	0.0830	0.0642	0.0665	0.0336
0.5176	0.5175	0.5317	0.5788	0.4630
0.0003	0.0506	0.0449	0.0340	0.0434
0.0003	0.0274	0.0003	0.0207	0.0167
0.0359	0.0515	0.0565	0.0556	0.0225
0.0003	0.0003	0.0003	0.0003	0.0003
0.0003	0.0003	0.0003	0.0003	0.0003
0.0003	0.0003	0.0003	0.0003	0.0003
0.0003	0.0003	0.0003	0.0003	0.0003
0.0003	0.0003	0.0272	0.0003	0.0341
0.1286	0.0481	0.0278	0.0392	0.1562
0.0003	0.0003	0.0003	0.0003	0.0003
0.0003	0.0003	0.0003	0.0003	0.0032
0.0003	0.0144	0.0108	0.0105	0.0037
0.0003	0.0003	0.0003	0.0069	0.0003
0.0003	0.0003	0.0003	0.0003	0.0003

0.0003	0.0077	0.0046	0.0046	0.0003
0.0097	0.0081	0.0003	0.0003	0.0066
0.0003	0.0071	0.0066	0.0105	0.0057
0.5093	0.0906	0.0933	0.0874	0.1843
0.1029	0.0225	0.0096	0.0219	0.1012
1.0176	0.3855	0.3427	0.4134	1.0799
1.8313	1.6958	1.6681	1.9020	1.6200
0.0202	0.0156	0.0143	0.0142	0.0175
0.0078	0.0247	0.0188	0.0304	0.0072
0.1434	0.0989	0.0003	0.1714	0.0812
0.0003	0.2096	0.1473	0.1851	0.0003
0.0003	0.0089	0.0003	0.0003	0.0003
0.0003	0.0003	0.0003	0.0003	0.0003
4.3042	1.4568	0.9656	1.0918	2.2757
0.0003	0.0003	0.0003	0.0003	0.0003
0.0003	0.0003	0.0003	0.0003	0.3157
1.1370	1.6674	1.3679	1.6550	1.7008
0.0003	0.0003	0.0003	0.0003	0.0003
0.0299	0.0501	0.0477	0.0432	0.0623
0.0003	0.0003	0.0003	0.0003	0.0003
0.0003	0.0003	0.0003	0.0003	0.0003
0.0003	0.0003	0.0230	0.0003	0.0003
0.0003	0.0003	0.0003	0.0003	0.0003
0.0003	0.0003	0.0003	0.0003	0.0003
0.0165	0.0003	0.0003	0.0003	0.0003
0.0003	0.0003	0.0003	0.0003	0.0003
0.0003	0.0003	0.0003	0.0003	0.0003
0.0003	0.0075	0.0074	0.0003	0.0003
0.0003	0.0003	0.0003	0.0003	0.0003
0.0003	0.0003	0.0003	0.0003	0.0003
0.0003	0.0003	0.0003	0.0003	0.0003
0.0003	0.0003	0.0003	0.0003	0.0003
0.0003	0.0003	0.0003	0.0003	0.0003
0.0003	0.0003	0.0003	0.0003	0.0003

0.0003	0.0003	0.0003	0.0003	0.0003
0.0003	0.0003	0.0003	0.0003	0.0003
0.0003	0.0003	0.0003	0.0003	0.0003
0.0003	0.0003	0.0003	0.0003	0.0003
0.0003	0.0003	0.0003	0.0003	0.0003
0.0003	0.0003	0.0003	0.0003	0.0003
0.0003	0.0003	0.0003	0.0003	0.0003
0.0003	0.1054	0.0841	0.1128	0.0335

CA 14 VULV IL + AA 2 101	CA 14 VULV IL + AA 3 102	CA 14 VEST POLY IC + AA 1 103	CA 14 VEST POLY IC + AA 2 104
0.0003	0.0003	0.0003	0.0003
0.0084	0.0070	0.0094	0.0003
0.0003	0.0003	0.0003	0.0003
0.3480	0.4157	0.8205	1.9133
1.3307	1.1580	1.1894	1.3934
0.0003	0.0003	0.0003	0.0003
0.0313	0.0261	0.0003	0.0370
0.0003	0.0003	0.0003	0.0003
0.0003	0.0003	0.0003	0.0003
110.3512	90.7646	110.0082	123.9839
0.1815	0.1533	0.2181	0.1829
5.2009	4.4244	5.1885	6.5991
0.0003	0.0003	3.2747	3.2308
27.4237	25.7257	25.3351	24.8934
0.0003	0.0003	0.0003	0.0003
0.0003	0.0003	0.0003	0.0003
0.0003	0.1127	0.1506	0.2065
0.0003	0.0003	0.0003	0.0003
12.0146	11.0096	11.2065	11.0053
0.0003	0.0003	0.0003	0.0003
0.0626	0.0668	0.0942	0.0689
3.0700	3.1520	3.3381	2.8033
0.0003	0.0003	0.0003	0.0003
0.0003	0.0003	0.0003	0.0003
0.0003	0.0003	0.0158	0.0582
0.6678	0.5693	0.6487	0.9115
5.9310	5.0099	6.0312	7.4834
0.0550	0.0522	0.0708	0.0573
0.0003	0.0003	0.0003	0.0003
0.0003	0.0003	0.0003	0.0003
0.0341	0.0003	0.0003	0.0660
0.1376	0.1112	0.0920	0.1221
5.8779	5.7200	0.9968	1.2934

0.0003	0.0003	0.0003	0.0003
0.0003	0.0003	0.0003	0.0003
0.0003	0.0843	0.0680	0.0720
0.0003	0.0351	0.0003	0.0417
0.0003	0.0003	0.0003	0.0003
0.0003	0.0003	0.0003	0.0064
0.0003	0.0003	0.0003	0.0003
0.0003	0.0003	0.0003	0.0003
0.7669	0.7195	0.7900	0.8354
0.0003	0.0003	0.0003	0.0166
0.0003	0.0003	0.0003	0.0003
0.0130	0.0162	0.0003	0.0003
0.0003	0.0003	0.0003	0.0003
0.0003	0.0003	0.0003	0.0003
0.0003	0.0003	0.0003	0.0003
0.0399	0.0485	0.0382	0.0003
0.5778	0.6082	0.6544	0.7230
0.9262	1.8749	1.0399	1.0810
0.0003	0.0058	0.0003	0.0895
0.0003	0.0003	0.1684	0.0003
7.3918	6.4756	3.0272	4.6310
0.0003	0.0003	0.0003	0.0003
0.0003	0.0003	0.0003	0.0003
0.0003	0.0003	0.0003	0.0003
0.0003	0.0003	0.0003	0.0003
0.0369	0.0244	0.0304	0.0130
0.1236	0.1649	0.5244	0.2133
0.0003	0.0003	0.0003	0.0003
19.4576	17.7209	33.1926	5.5147
14.3617	10.9591	18.5914	2.9762
4.3899	3.7724	8.2545	1.4339
165.9658	157.5945	154.3721	104.2383
102.4818	108.7374	70.9681	24.6235
238.6905	228.2773	201.5591	110.3805

0.0003	0.0003	0.0003	0.0003
34.1911	45.8297	47.4992	92.0768
9.5496	11.1168	4.3444	4.7574
0.1154	0.1219	0.2704	0.0963
0.1267	0.1137	0.2028	0.0912
0.0003	0.0003	0.0003	0.0003
0.6348	0.5974	1.2853	1.2425
0.0003	0.1647	0.2703	0.2712
0.2613	0.3199	0.6520	0.8838
0.5446	0.6093	0.8176	0.5744
0.2659	0.2712	1.2306	0.6828
0.0526	0.0432	0.1483	0.0502
0.1412	0.1753	0.3842	0.3089
0.1338	0.1096	0.4233	0.2086
0.1686	0.1172	0.3926	0.2776
0.7399	0.6608	2.1210	2.4174
0.2177	0.2188	0.5159	0.3792
0.2392	0.2695	0.7301	0.6079
0.0393	0.0490	0.1394	0.1649
0.6281	0.6604	1.5794	0.7927
0.0254	0.0648	0.0607	0.0885
0.0003	0.0003	0.0224	0.0416
0.0347	0.0327	0.0828	0.0583
0.0003	0.0003	0.0003	0.0003
0.0003	0.0003	0.0003	0.0003
0.0003	0.0003	0.0003	0.0003
0.0003	0.0003	0.0003	0.0003
0.0003	0.0003	0.0003	0.0003
0.1780	0.1742	0.0620	0.0333
0.0003	0.0003	0.0003	0.0003
0.0003	0.0003	0.0062	0.0003
0.0003	0.0003	0.0172	0.0003
0.0003	0.0003	0.0003	0.0003
0.0003	0.0003	0.0003	0.0003

0.0003	0.0003	0.0003	0.0003
0.0107	0.0003	0.0103	0.0064
0.0100	0.0110	0.0003	0.0093
0.4987	0.4040	0.4615	0.1742
0.1443	0.1273	0.1132	0.0288
1.4146	1.2785	1.1228	0.6305
2.4778	2.7473	2.1619	2.2022
0.0299	0.0303	0.0310	0.0003
0.0003	0.0145	0.0326	0.0237
0.0003	0.0003	0.2303	0.1951
0.7183	0.0003	0.3251	0.2737
0.0003	0.0148	0.0176	0.0197
0.0003	0.0003	0.0003	0.0003
1.8524	1.6050	5.5694	1.5494
0.0003	0.0003	0.0003	0.0003
0.4107	0.4039	0.0003	0.0003
1.7448	2.2738	2.0522	1.7918
0.0003	0.0003	0.0003	0.0003
0.0590	0.0844	0.0518	0.0592
0.0003	0.0003	0.0003	0.0003
0.0003	0.0003	0.0003	0.0003
0.0003	0.0003	0.0003	0.0003
0.0003	0.0003	0.0003	0.0003
0.0003	0.0003	0.0003	0.0003
0.0220	0.0104	0.0003	0.0003
0.0003	0.0003	0.0003	0.0003
0.0003	0.0003	0.0003	0.0003
0.0089	0.0003	0.0117	0.0108
0.0003	0.0003	0.0003	0.0003
0.0003	0.0003	0.0003	0.0003
0.0003	0.0003	0.0003	0.0003
0.0003	0.0003	0.0003	0.0003
0.0003	0.0003	0.0003	0.0003
0.0003	0.0003	0.0003	0.0003

0.0003	0.0003	0.0178	0.0299
0.0003	0.0003	0.0003	0.0003
0.0003	0.0003	0.0003	0.0003
0.0003	0.0003	0.0003	0.0003
0.0003	0.0003	0.0003	0.0003
0.0003	0.0003	0.0003	0.0003
0.0003	0.0003	0.0003	0.0003
0.0131	0.0272	0.1209	0.1234

CA 14 VEST POLY IC + AA 3 105	CA 14 VULV POLY IC + AA 1 106	CA 14 VULV POLY IC + AA 2 107	CA 14 VULV POLY IC + AA 3 108
0.0003	0.0003	0.0003	0.0003
0.0003	0.0064	0.0003	0.0003
0.0003	0.0003	0.0003	0.0003
1.9977	0.0003	0.0003	0.0003
1.4761	0.5383	0.6461	0.6107
0.0003	0.0003	0.0003	0.0003
0.0364	0.0003	0.0003	0.0003
0.0003	0.0003	0.0003	0.0003
0.0003	0.0003	0.0003	0.0003
137.3837	40.3557	53.5616	42.5389
0.2014	0.0927	0.1122	0.0831
5.8656	1.7634	2.1382	1.6604
0.0003	0.0003	1.3876	0.0003
21.2199	9.3770	12.4557	8.1861
0.0003	0.0003	0.0003	0.0003
0.0003	0.0003	0.0003	0.0003
0.2636	0.0003	0.0003	0.0003
0.0003	0.0003	0.0003	0.0003
8.8644	4.7248	5.7449	3.8508
0.0003	0.0003	0.0003	0.0003
0.0567	0.0666	0.0825	0.0498
2.5778	2.3975	2.2036	2.1393
0.0003	0.0003	0.0003	0.0003
0.0003	0.0003	0.0003	0.0003
0.0497	0.0003	0.0003	0.0003
0.9622	0.2195	0.2600	0.1434
7.4902	1.7319	2.4389	2.1916
0.0567	0.0444	0.0486	0.0369
0.0003	0.0003	0.0003	0.0003
0.0003	0.0003	0.0003	0.0003
0.0608	0.0003	0.0003	0.0003
0.0898	0.0770	0.0885	0.0913
1.3109	2.6036	3.7664	3.2520

0.0003	0.0003	0.0003	0.0003
0.0003	0.0003	0.0003	0.0003
0.0646	0.0003	0.0397	0.0685
0.0542	0.0282	0.0343	0.0190
0.0003	0.0003	0.0003	0.0003
0.0003	0.0003	0.0003	0.0003
0.0003	0.0003	0.0003	0.0003
0.0003	0.0003	0.0003	0.0003
0.5511	0.6677	0.8502	0.6812
0.0003	0.0003	0.0003	0.0003
0.0003	0.0003	0.0003	0.0003
0.0049	0.0003	0.0003	0.0042
0.0003	0.0003	0.0003	0.0003
0.0003	0.0003	0.0003	0.0003
0.0003	0.0003	0.0003	0.0003
0.0003	0.0319	0.0354	0.0371
0.5201	0.5021	0.6158	0.6111
1.8254	0.7966	1.0974	0.9556
0.0003	0.0003	0.0003	0.0003
0.1131	0.1459	0.0003	0.1221
3.1279	4.3349	5.5784	2.4815
0.0003	0.0003	0.0003	0.0003
0.0003	0.0003	0.0003	0.0003
0.0003	0.0003	0.0003	0.0003
0.0284	0.0003	0.0003	0.0003
0.0003	0.0003	0.0300	0.0209
0.2547	0.2716	0.3216	0.2536
0.0003	0.0003	0.0003	0.0003
3.7614	64.5211	70.9527	29.2324
1.8531	33.6797	38.4697	17.2528
0.9095	16.4346	21.0616	8.1293
85.4315	168.9978	202.2518	121.2963
12.8127	95.7154	143.0196	83.1974
61.9175	249.6618	297.4458	214.4647

0.0003	0.0003	0.0003	0.0003
89.5535	15.6954	15.8493	19.7949
3.5212	4.7247	6.2854	4.7108
0.1210	0.2971	0.3394	0.2046
0.0288	0.1313	0.1337	0.1184
0.0003	0.0003	0.0003	0.0003
0.9928	0.2222	0.2935	0.3011
0.1567	0.1490	0.1650	0.1180
0.7684	0.1606	0.1400	0.1438
0.4046	0.6420	0.7900	0.5029
0.6876	0.3534	0.4241	0.2595
0.0876	0.0305	0.0629	0.0266
0.3108	0.1133	0.1879	0.1214
0.1890	0.1442	0.2109	0.1385
0.2068	0.1440	0.1657	0.1176
1.9806	0.4655	0.4955	0.4834
0.3401	0.1975	0.2678	0.1801
0.5564	0.1741	0.3147	0.2495
0.1054	0.0003	0.0422	0.0003
0.5786	0.4841	0.6794	0.5495
0.0517	0.0401	0.0418	0.0579
0.0003	0.0229	0.0177	0.0003
0.0658	0.0292	0.0300	0.0270
0.0003	0.0003	0.0003	0.0003
0.0003	0.0003	0.0003	0.0003
0.0003	0.0003	0.0003	0.0003
0.0003	0.0003	0.0003	0.0003
0.0003	0.0250	0.0003	0.0003
0.0325	0.1212	0.1349	0.1106
0.0003	0.0003	0.0003	0.0003
0.0003	0.0003	0.0003	0.0003
0.0209	0.0074	0.0041	0.0003
0.0003	0.0003	0.0028	0.0003
0.0003	0.0003	0.0003	0.0003

0.0088	0.0062	0.0003	0.0003
0.0003	0.0003	0.0109	0.0003
0.0003	0.0003	0.0003	0.0003
0.0970	0.5030	0.6802	0.4087
0.0198	0.1423	0.1417	0.1262
0.3806	1.0185	1.4225	1.2576
1.7325	1.7469	2.1515	1.6936
0.0003	0.0184	0.0273	0.0223
0.0206	0.0053	0.0003	0.0154
0.0847	0.1384	0.0737	0.0817
0.1395	0.0003	0.6125	0.4775
0.0003	0.0160	0.0003	0.0003
0.0003	0.0003	0.0003	0.0003
1.2719	4.5253	4.6629	2.2526
0.0003	0.0003	0.0003	0.0003
0.0003	0.0003	0.3289	0.0003
1.5518	1.8166	2.2784	1.6037
0.0003	0.0003	0.0003	0.0003
0.0486	0.0301	0.0507	0.0420
0.0003	0.0003	0.0003	0.0003
0.0003	0.0003	0.0003	0.0003
0.0003	0.0003	0.0003	0.0003
0.0003	0.0003	0.0003	0.0003
0.0003	0.0003	0.0003	0.0003
0.0003	0.0003	0.0003	0.0003
0.0003	0.0003	0.0003	0.0003
0.0003	0.0003	0.0003	0.0003
0.0104	0.0003	0.0059	0.0003
0.0003	0.0003	0.0003	0.0003
0.0387	0.0003	0.0003	0.0003
0.0003	0.0003	0.0003	0.0003
0.0003	0.0003	0.0003	0.0003
0.0003	0.0003	0.0003	0.0003
0.0003	0.0003	0.0003	0.0003

0.0349	0.0003	0.0345	0.0383
0.0003	0.0003	0.0003	0.0003
0.0003	0.0003	0.0003	0.0003
0.0003	0.0003	0.0003	0.0003
0.0003	0.0003	0.0003	0.0003
0.0003	0.0003	0.0003	0.0003
0.0003	0.0003	0.0163	0.0003
0.0947	0.0170	0.0377	0.0331

CA 20 VEST AA 1 127	CA 20 VEST AA 2 128	CA 20 VEST AA 3 129	CA 20 VULV AA 1 130	CA 20 VULV AA 2 131	CA 20 VULV AA 3 132
0.0003	0.0003	0.0003	0.0003	0.0003	0.0003
0.0003	0.0003	0.0003	0.0003	0.0003	0.0003
0.0003	0.0003	0.0003	0.0003	0.0003	0.0003
0.0003	0.1294	0.0003	0.0003	0.0003	0.0003
0.4108	0.4221	0.3639	0.4724	0.4239	0.3455
0.0003	0.0003	0.0003	0.0003	0.0003	0.0003
0.0003	0.0003	0.0003	0.0245	0.0003	0.0003
0.0003	0.0003	0.0003	0.0003	0.0003	0.0003
0.0003	0.0003	0.0003	0.0003	0.0003	0.0003
30.0782	29.3862	31.6996	37.5268	33.5820	33.5688
0.0536	0.0650	0.1112	0.0431	0.0382	0.0338
1.3848	1.3255	1.3201	1.5729	1.5828	1.5326
0.0003	0.1414	0.1944	0.7142	0.0003	0.5579
4.8436	3.5004	3.8657	6.5768	5.4765	4.1763
0.0003	0.0003	0.0003	0.0003	0.0003	0.0003
0.0003	0.0003	0.0003	0.0003	0.0003	0.0003
0.0003	0.0433	0.0003	0.0003	0.0003	0.0003
0.0003	0.0003	0.0003	0.0003	0.0003	0.0003
2.3093	1.5453	1.6360	2.7544	2.5264	2.0105
0.0003	0.0003	0.0003	0.0003	0.0003	0.0003
0.0455	0.0380	0.0409	0.0452	0.0398	0.0384
1.9477	2.4576	2.2346	2.0144	2.2632	2.1316
0.0003	0.0003	0.0003	0.0003	0.0003	0.0003
0.0003	0.0003	0.0003	0.0003	0.0003	0.0003
0.0003	0.0003	0.0003	0.0003	0.0003	0.0003
0.1454	0.0816	0.1423	0.1539	0.2012	0.1629
1.2239	1.1872	0.9653	1.5994	1.7370	1.3150
0.0504	0.0431	0.0559	0.0522	0.0568	0.0464
0.0003	0.0003	0.0003	0.0003	0.0003	0.0003
0.0003	0.0003	0.0003	0.0003	0.0003	0.0003
0.0003	0.0003	0.0003	0.0003	0.0003	0.0003
0.1832	0.1719	0.1175	0.1404	0.2837	0.2329
0.7604	0.6940	0.7582	3.8923	4.1256	4.4501

0.0003	0.0003	0.0003	0.0003	0.0003	0.0003
0.0003	0.0003	0.0003	0.0003	0.0003	0.0003
0.0452	0.0745	0.0003	0.0003	0.0853	0.0652
0.0003	0.0285	0.0003	0.0305	0.0318	0.0353
0.0003	0.0003	0.0003	0.0003	0.0003	0.0003
0.0003	0.0003	0.0003	0.0003	0.0003	0.0003
0.0003	0.0003	0.0003	0.0003	0.0003	0.0003
0.0003	0.0003	0.0003	0.0003	0.0003	0.0003
0.4454	0.5041	0.5096	0.4836	0.5306	0.4633
0.0003	0.0003	0.0003	0.0003	0.0003	0.0003
0.0003	0.0003	0.0003	0.0003	0.0003	0.0003
0.0003	0.0172	0.0344	0.0092	0.0099	0.0428
0.0003	0.0003	0.0003	0.0003	0.0003	0.0003
0.0003	0.0003	0.0003	0.0003	0.0003	0.0003
0.0003	0.0003	0.0003	0.0003	0.0003	0.0003
0.0131	0.0116	0.0003	0.0198	0.0003	0.0106
0.3699	0.3917	0.3612	0.4543	0.3728	0.3848
0.5676	0.6126	0.6071	1.4689	0.6337	0.5354
0.0003	0.0003	0.0140	0.0003	0.0344	0.0003
0.0810	0.0003	0.0003	0.0003	0.1098	0.2290
1.7956	2.2608	1.1831	7.3261	3.2605	1.5218
0.0003	0.0003	0.0003	0.0003	0.0003	0.0003
0.0003	0.0003	0.0003	0.0003	0.0003	0.0003
0.0003	0.0003	0.0003	0.0003	0.0003	0.0003
0.0003	0.0003	0.0003	0.0003	0.0003	0.0228
0.0003	0.0003	0.0003	0.0124	0.0003	0.0003
0.1112	0.0995	0.1261	0.1266	0.0782	0.1195
0.0302	0.0296	0.0211	0.0003	0.0388	0.0416
1.6835	1.6649	1.5398	4.1866	2.0484	2.0378
1.0376	0.8632	0.9725	1.7006	0.8937	0.8787
0.7882	0.6486	0.8919	1.1552	0.8376	0.8822
10.8384	10.2456	10.2647	27.7728	18.5623	24.3322
3.6228	2.4479	2.0240	23.3858	4.8647	2.6746
5.8194	4.1950	3.4643	27.2683	5.8633	5.5257

0.6454	0.6929	0.5156	2.1901	0.7806	0.4117
81.9181	88.1030	88.2789	65.7075	98.1581	113.6819
0.6145	0.5984	0.5972	4.9528	2.2296	1.9316
0.0003	0.0003	0.0003	0.0683	0.0619	0.0003
0.0003	0.0003	0.0003	0.0427	0.0003	0.0003
0.0003	0.0003	0.0003	0.0003	0.0003	0.0003
0.0741	0.1016	0.0662	0.2124	0.1064	0.1577
0.0479	0.0499	0.0500	0.1325	0.1293	0.0821
0.1086	0.1000	0.0900	0.1919	0.1175	0.0973
0.1225	0.1164	0.0754	0.3558	0.1717	0.1467
0.2249	0.2498	0.2583	0.3568	0.2482	0.3027
0.0381	0.0161	0.0240	0.0464	0.0003	0.0003
0.1014	0.0942	0.1124	0.1420	0.0841	0.0823
0.1049	0.0705	0.1049	0.1351	0.0600	0.0744
0.0776	0.0768	0.1168	0.1104	0.0922	0.0527
0.2828	0.2490	0.2796	0.5064	0.3920	0.4827
0.0904	0.0003	0.0003	0.0003	0.1000	0.0003
0.1926	0.2159	0.2031	0.3298	0.2322	0.1744
0.0003	0.0003	0.0003	0.0245	0.0003	0.0003
0.2247	0.2408	0.2087	0.4401	0.2231	0.2359
0.0305	0.0462	0.0276	0.0322	0.0415	0.3373
0.0184	0.0325	0.0003	0.0003	0.0127	0.1484
0.0240	0.0311	0.0299	0.0313	0.0452	0.0452
0.0003	0.0003	0.0003	0.0003	0.0003	0.0003
0.0003	0.0548	0.0003	0.0003	0.0523	0.0003
0.0212	0.0234	0.0258	0.0003	0.0003	0.0402
0.0003	0.0067	0.0003	0.0003	0.0003	0.0003
0.0003	0.0003	0.0003	0.0003	0.0003	0.0003
0.0003	0.0003	0.0003	0.0387	0.0003	0.0003
0.0003	0.0003	0.0003	0.0003	0.0003	0.0003
0.0023	0.0003	0.0003	0.0003	0.0003	0.0003
0.0003	0.0087	0.0003	0.0003	0.0003	0.0079
0.0003	0.0003	0.0003	0.0003	0.0003	0.0003
0.0003	0.0003	0.0003	0.0003	0.0003	0.0003

0.0055	0.0003	0.0114	0.0003	0.0003	0.0003
0.0003	0.0042	0.0003	0.0051	0.0003	0.0235
0.0003	0.0076	0.0071	0.0055	0.0003	0.0150
0.0359	0.0349	0.0305	0.1874	0.0475	0.0120
0.0096	0.0054	0.0106	0.0396	0.0003	0.0091
0.1865	0.2121	0.1942	0.6457	0.3320	0.2127
1.2983	1.5822	1.6006	1.6610	1.9851	1.8765
0.0084	0.0003	0.0003	0.0148	0.0098	0.0003
0.0168	0.0176	0.0135	0.0133	0.0176	0.0159
0.1638	0.1289	0.0003	0.1164	0.1176	0.3298
0.1552	0.0003	0.2186	0.1632	0.1248	0.6155
0.0003	0.0003	0.0109	0.0003	0.0003	0.0332
0.0003	0.0003	0.0003	0.0003	0.0003	0.0003
0.6165	0.4650	0.4757	0.8479	0.5692	0.4954
0.0003	0.0003	0.0003	0.0003	0.0003	0.0003
0.0003	0.0003	0.0003	0.0003	0.0003	0.0003
1.3651	1.4879	1.6999	1.4457	1.6339	1.5921
0.0003	0.0003	0.0003	0.0003	0.0003	0.0003
0.0361	0.0256	0.0349	0.0430	0.0434	0.0378
0.0003	0.0003	0.0003	0.0061	0.0003	0.0003
0.0003	0.0003	0.0003	0.0003	0.0003	0.0003
0.0003	0.0003	0.0003	0.0003	0.0003	0.0003
0.0003	0.0003	0.0003	0.0003	0.0003	0.0003
0.0003	0.0003	0.0003	0.0003	0.0003	0.0003
0.0003	0.0003	0.0003	0.0003	0.0225	0.0003
0.0003	0.0003	0.0003	0.0003	0.0003	0.0003
0.0003	0.0003	0.0003	0.0003	0.0003	0.0003
0.0066	0.0061	0.0003	0.0055	0.0003	0.0060
0.0003	0.0003	0.0003	0.0003	0.0003	0.0003
0.0003	0.0003	0.0003	0.0003	0.0003	0.0003
0.0003	0.0003	0.0003	0.0003	0.0003	0.0003
0.0003	0.0003	0.0003	0.0003	0.0003	0.0003
0.0003	0.0003	0.0003	0.0003	0.0003	0.0003
0.0003	0.0003	0.0003	0.0003	0.0003	0.0003

0.0003	0.0003	0.0003	0.0003	0.0003	0.0003
0.0003	0.0003	0.0003	0.0003	0.0003	0.0003
0.0003	0.0003	0.0003	0.0003	0.0003	0.0003
0.0003	0.0003	0.0003	0.0003	0.0003	0.0003
0.0003	0.0003	0.0003	0.0003	0.0003	0.0003
0.0003	0.0003	0.0003	0.0003	0.0003	0.0003
0.0003	0.0003	0.0003	0.0003	0.0003	0.0222
0.0688	0.0837	0.0521	0.0762	0.1231	0.0756

CA 20 VEST IL + AA 1 133	CA 20 VEST IL + AA 2 134	CA 20 VEST IL + AA 3 135	CA 20 VULV IL + AA 1 136	CA 20 VULV IL + AA 2 137
0.0003	0.0003	0.0003	0.0003	0.0003
0.0081	0.0003	0.0003	0.0003	0.0184
0.0003	0.0003	0.0003	0.0003	0.0003
0.7641	0.5544	0.5089	0.5369	0.6333
0.9735	0.9585	0.8696	1.1805	1.2341
0.0003	0.0003	0.0042	0.0003	0.0003
0.0468	0.0436	0.0003	0.0546	0.0003
0.0003	0.0003	0.0003	0.0003	0.0003
0.0003	0.0003	0.0003	0.0003	0.0003
87.5997	92.1744	74.3164	110.5546	126.5702
0.2202	0.1660	0.1660	0.1911	0.1170
4.3251	4.2488	3.7285	4.8280	5.1664
2.3525	0.0003	1.8442	2.4629	0.0003
17.2642	14.3193	13.5455	18.6319	21.0494
0.0003	0.0003	0.0003	0.0003	0.0003
0.0003	0.0003	0.0003	0.0003	0.0003
0.0003	0.1199	0.0003	0.0003	0.0995
0.0003	0.0003	0.0003	0.0003	0.0003
7.4311	6.3430	5.9379	8.3475	9.5266
0.0003	0.0003	0.0003	0.0003	0.0003
0.0608	0.0492	0.0724	0.0560	0.0575
2.4173	2.4205	2.3158	2.5122	2.2893
0.0003	0.0003	0.0003	0.0003	0.0003
0.0003	0.0003	0.0003	0.0003	0.0003
0.0197	0.0242	0.0221	0.0003	0.0249
0.8835	0.6689	0.6177	1.0477	1.1120
4.7859	4.1801	4.1668	5.6924	6.1224
0.0583	0.0440	0.0368	0.1002	0.0553
0.0003	0.0003	0.0003	0.0003	0.0003
0.0003	0.0003	0.0003	0.0003	0.0003
0.0560	0.0546	0.0351	0.0781	0.0570
0.1490	0.1634	0.1446	0.3273	0.2352
1.8647	1.9608	1.2261	8.0358	8.6595

0.0003	0.0003	0.0003	0.0003	0.0003
0.0003	0.0003	0.0003	0.0003	0.0003
0.0778	0.1026	0.0003	0.0623	0.0475
0.0003	0.0003	0.0003	0.0003	0.0003
0.0003	0.0003	0.0003	0.0003	0.0003
0.0003	0.0003	0.0003	0.0003	0.0003
0.0003	0.0003	0.0003	0.0003	0.0003
0.0003	0.0003	0.0003	0.0003	0.0003
0.4608	0.4693	0.5042	0.5384	0.5358
0.0003	0.0003	0.0080	0.0003	0.0003
0.0003	0.0003	0.0003	0.0003	0.0003
0.0003	0.0003	0.0003	0.0003	0.0003
0.0003	0.0003	0.0003	0.0003	0.0003
0.0003	0.0003	0.0003	0.0003	0.0003
0.0003	0.0003	0.0003	0.0003	0.0003
0.0152	0.0093	0.0085	0.0003	0.0295
0.4384	0.4478	0.5253	0.4897	0.5540
0.5605	0.5603	0.5596	0.5701	0.5955
0.0756	0.0003	0.0003	0.2288	0.0003
0.0897	0.0003	0.0871	0.0003	0.0599
0.6610	0.7244	0.5981	1.9383	4.4797
0.0003	0.0003	0.0003	0.0003	0.0003
0.0003	0.0003	0.0003	0.0003	0.0003
0.0003	0.0003	0.0003	0.0003	0.0003
0.0003	0.0003	0.0003	0.0165	0.0003
0.0003	0.0003	0.0003	0.0003	0.0003
0.0659	0.0741	0.0661	0.0474	0.0954
0.0296	0.0258	0.0213	0.0406	0.0339
1.7576	1.8919	1.6831	2.3552	2.4646
0.6020	0.7029	0.6446	0.6621	0.7060
0.4567	0.4501	0.4642	0.6211	0.5580
17.6465	15.3876	13.3546	28.9324	22.8526
1.3371	1.3782	1.0565	1.3760	2.9469
4.9837	4.1766	4.1887	5.8113	5.6952

0.2176	0.1559	0.1748	0.0003	0.4162
104.0099	101.6946	91.1867	119.1532	123.1922
1.1211	0.5269	0.7853	3.9721	6.0327
0.0003	0.0003	0.0003	0.0003	0.0478
0.0003	0.0003	0.0003	0.0003	0.0003
0.0189	0.0003	0.0003	0.0003	0.0003
0.0484	0.0867	0.0003	0.1182	0.1642
0.0003	0.0003	0.0003	0.0003	0.0696
0.0935	0.0821	0.0489	0.1143	0.1699
0.0555	0.0543	0.0300	0.0613	0.0840
0.2660	0.2409	0.2053	0.2600	0.2042
0.0221	0.0119	0.0202	0.0288	0.0188
0.0655	0.0976	0.0874	0.0757	0.0899
0.0614	0.0709	0.0535	0.0672	0.0665
0.0819	0.0769	0.0564	0.0707	0.0655
0.2074	0.2099	0.1619	0.4107	0.3250
0.0812	0.0878	0.0650	0.0724	0.0573
0.1514	0.1082	0.1193	0.1185	0.1613
0.0003	0.0003	0.0003	0.0504	0.0003
0.1302	0.0951	0.0874	0.1215	0.1270
0.0452	0.0146	0.0003	0.0264	0.0161
0.0187	0.0142	0.0219	0.0003	0.0003
0.0254	0.0235	0.0240	0.0289	0.0263
0.0003	0.0003	0.0003	0.0003	0.0003
0.0003	0.0003	0.0003	0.0003	0.0692
0.0256	0.0108	0.0203	0.0276	0.0316
0.0003	0.0039	0.0003	0.0003	0.0003
0.0003	0.0080	0.0003	0.0003	0.0003
0.0003	0.0091	0.0003	0.0003	0.0003
0.0003	0.0003	0.0003	0.0003	0.0003
0.0003	0.0003	0.0003	0.0003	0.0003
0.0003	0.0003	0.0032	0.0079	0.0042
0.0003	0.0003	0.0003	0.0003	0.0036
0.0003	0.0003	0.0003	0.0003	0.0003

0.0003	0.0003	0.0003	0.0003	0.0003
0.0024	0.0003	0.0039	0.0055	0.0003
0.0003	0.0038	0.0003	0.0003	0.0056
0.0241	0.0239	0.0330	0.0409	0.0367
0.0051	0.0003	0.0003	0.0074	0.0003
0.0923	0.1120	0.1398	0.1982	0.2576
1.3264	1.6933	1.6818	2.1523	1.8364
0.0003	0.0003	0.0003	0.0096	0.0203
0.0132	0.0164	0.0164	0.0119	0.0145
0.1557	0.0776	0.1326	0.1450	0.1138
0.1583	0.0771	0.0003	0.0951	0.1467
0.0232	0.0003	0.0102	0.0433	0.0003
0.0003	0.0003	0.0003	0.0003	0.0003
0.6400	0.5212	0.6042	0.5744	0.5957
0.0003	0.0003	0.0003	0.0003	0.0003
0.0003	0.0003	0.0003	0.0003	0.0003
1.4847	1.6843	1.1888	1.6643	1.3925
0.0003	0.0003	0.0003	0.0003	0.0003
0.0526	0.0494	0.0496	0.0505	0.0558
0.0003	0.0003	0.0003	0.0076	0.0003
0.0003	0.0003	0.0003	0.0003	0.0003
0.0003	0.0003	0.0003	0.0003	0.0003
0.0003	0.0003	0.0003	0.0003	0.0003
0.0003	0.0003	0.0003	0.0003	0.0003
0.0144	0.0003	0.0003	0.0003	0.0003
0.0003	0.0003	0.0003	0.0003	0.0003
0.0003	0.0003	0.0003	0.0003	0.0003
0.0055	0.0057	0.0039	0.0003	0.0056
0.0003	0.0003	0.0003	0.0003	0.0003
0.0003	0.0003	0.0003	0.0003	0.0003
0.0003	0.0003	0.0003	0.0003	0.0003
0.0003	0.0003	0.0003	0.0003	0.0003
0.0003	0.0003	0.0003	0.0003	0.0003
0.0003	0.0003	0.0003	0.0003	0.0003

0.0003	0.0003	0.0003	0.0003	0.0003
0.0003	0.0003	0.0003	0.0003	0.0003
0.0003	0.0003	0.0003	0.0003	0.0003
0.0003	0.0003	0.0003	0.0003	0.0003
0.0003	0.0003	0.0003	0.0003	0.0003
0.0003	0.0003	0.0003	0.0003	0.0003
0.0003	0.0003	0.0003	0.0003	0.0003
0.0539	0.0734	0.0721	0.0949	0.0887

CA 20 VULV IL + AA 3 138	CA 20 VEST POLY IC + AA 1 139	CA 20 VEST POLY IC + AA 2 140	CA 20 VEST POLY IC + AA 3 141
0.0003	0.0003	0.0003	0.0003
0.0003	0.0003	0.0121	0.0082
0.0003	0.0003	0.0003	0.0003
0.6210	2.0795	3.3438	3.2116
1.4370	0.9819	1.2020	1.4420
0.0003	0.0071	0.0104	0.0129
0.0645	0.0314	0.0003	0.0523
0.0003	0.0003	0.0003	0.0003
0.0003	0.0003	0.0003	0.0003
107.8221	127.9464	113.0527	139.6091
0.1695	0.6078	0.8966	1.0222
5.6625	5.3578	6.6578	6.8993
0.0003	0.0003	0.0003	2.1554
17.7861	16.6465	16.9013	18.0435
0.0003	0.0003	0.0003	0.0003
0.0003	0.0003	0.0003	0.0003
0.0003	0.1899	0.2428	0.2272
0.0003	0.0003	0.0003	0.0003
8.0702	7.7795	7.3127	7.8327
0.0003	0.0003	0.0003	0.0003
0.0444	0.0500	0.0477	0.0522
2.2694	2.4755	2.3946	3.9201
0.0003	0.0003	0.1363	0.0848
0.0003	0.0003	0.0003	0.0003
0.0209	0.0395	0.0702	0.0736
1.3694	0.6926	0.8216	0.9887
5.8692	5.3641	6.8742	8.5469
0.0533	0.0396	0.0593	0.0485
0.0003	0.0003	0.0003	0.0003
0.0003	0.0003	0.0003	0.0003
0.0804	0.0401	0.0682	0.0474
0.2584	0.0734	0.0842	0.0718
10.5481	2.0026	1.9764	2.0493

0.0003	0.0003	0.0003	0.0003
0.0003	0.0003	0.0003	0.0003
0.0814	0.0829	0.0705	0.0831
0.0003	0.0960	0.0828	0.1394
0.0003	0.0003	0.0003	0.0003
0.0003	0.0003	0.0003	0.0003
0.0003	0.0003	0.0003	0.0003
0.0003	0.0003	0.0003	0.0003
0.4695	0.6710	0.6142	0.5316
0.0003	0.0003	0.0003	0.0003
0.0003	0.0003	0.0003	0.0003
0.0003	0.0003	0.0003	0.0003
0.0003	0.0003	0.0003	0.0003
0.0003	0.0003	0.0003	0.0003
0.0003	0.0003	0.0003	0.0003
0.0139	0.0220	0.0083	0.0003
0.5178	0.5933	0.4624	0.3958
0.5596	0.9310	0.7971	0.6429
0.0003	0.0003	0.0718	0.0003
0.0844	0.1070	0.0003	0.1867
1.3385	6.0907	1.8188	2.7335
0.0003	0.0003	0.0003	0.0236
0.0003	0.0003	0.0003	0.0003
0.0003	0.0003	0.0003	0.0003
0.0003	0.0351	0.0541	0.0003
0.0003	0.0003	0.0003	0.0003
0.0471	0.1600	0.0944	0.1112
0.0284	0.0003	0.0342	0.0325
1.7033	4.5617	2.2022	2.2957
0.5157	1.3504	0.5371	0.6716
0.3711	1.0446	0.5476	0.6617
21.4682	90.2355	40.8110	114.9058
1.1088	9.0482	2.3085	1.4920
4.7058	45.5583	36.5164	37.1590

0.1339	0.0003	0.0003	0.0003
114.7004	94.8700	94.5006	77.4778
3.3529	3.8618	2.7107	2.0863
0.0003	0.0733	0.0003	0.0003
0.0003	0.0003	0.0003	0.0003
0.0003	0.0003	0.0003	0.0003
0.0965	0.5076	0.5803	0.6446
0.0003	0.0003	0.0003	0.0003
0.1048	0.3577	0.2335	0.2179
0.0003	0.1286	0.0731	0.0428
0.2140	0.4155	0.3005	0.2970
0.0003	0.0376	0.0283	0.0142
0.0629	0.1215	0.0808	0.0688
0.0537	0.0841	0.0649	0.0611
0.0483	0.1056	0.0003	0.0003
0.3439	1.2875	1.3482	1.4855
0.0003	0.1521	0.1067	0.0568
0.1495	0.2427	0.2010	0.2118
0.0003	0.0357	0.0003	0.0616
0.1068	0.2559	0.1430	0.1444
0.0271	0.0332	0.0346	0.0003
0.0003	0.0128	0.0003	0.0003
0.0312	0.0262	0.0257	0.0220
0.0003	0.0003	0.0003	0.0003
0.0003	0.0003	0.0003	0.0003
0.0003	0.0003	0.0003	0.0003
0.0003	0.0003	0.0003	0.0003
0.0003	0.0003	0.0003	0.0003
0.0003	0.0003	0.0429	0.0288
0.0003	0.0003	0.0003	0.0003
0.0003	0.0003	0.0003	0.0003
0.0003	0.0003	0.0103	0.0003
0.0003	0.0003	0.0003	0.0003
0.0003	0.0003	0.0003	0.0003

0.0003	0.0044	0.0003	0.0003
0.0003	0.0003	0.0081	0.0031
0.0003	0.0066	0.0003	0.0003
0.0149	0.0730	0.0216	0.0275
0.0040	0.0197	0.0043	0.0003
0.1662	0.3499	0.1412	0.1251
1.8515	1.9833	1.9108	1.7425
0.0003	0.0151	0.0003	0.0003
0.0153	0.0159	0.0152	0.0166
0.0686	0.0003	0.1153	0.1685
0.0003	0.0003	0.1173	0.2121
0.0102	0.0146	0.0003	0.0003
0.0003	0.0003	0.0003	0.0003
0.4481	0.6727	0.3704	0.5192
0.0003	0.0003	0.0003	0.0003
0.0003	0.0003	0.0003	0.0003
1.5300	1.8524	1.9416	1.4279
0.0003	0.0003	0.0003	0.0003
0.0440	0.0426	0.0439	0.0284
0.0003	0.0003	0.0003	0.0003
0.0003	0.0003	0.0003	0.0003
0.0316	0.0003	0.0003	0.0003
0.0003	0.0003	0.0003	0.0003
0.0003	0.0003	0.0003	0.0003
0.0003	0.0003	0.0003	0.0237
0.0003	0.0003	0.0003	0.0003
0.0003	0.0003	0.0003	0.0003
0.0074	0.0100	0.0003	0.0003
0.0003	0.0003	0.0003	0.0003
0.0003	0.0003	0.0003	0.0003
0.0003	0.0003	0.0003	0.0003
0.0003	0.0003	0.0003	0.0003
0.0003	0.0003	0.0003	0.0003
0.0003	0.0003	0.0003	0.0003

0.0003	0.0232	0.0003	0.0003
0.0003	0.0003	0.0003	0.0003
0.0003	0.0003	0.0003	0.0003
0.0003	0.0003	0.0003	0.0003
0.0003	0.0003	0.0003	0.0003
0.0003	0.0003	0.0003	0.0003
0.0003	0.0003	0.0003	0.0003
0.0882	0.0554	0.0939	0.0574

CA 20 VULV POLY IC + AA 1 142	CA 20 VULV POLY IC + AA 2 143	CA 20 VULV POLY IC + AA 3 144	CO 21 VEST AA 1 163	CO 21 VEST AA 2 164
0.0003	0.0003	0.0003	0.0003	0.0003
0.0098	0.0003	0.0003	0.0003	0.0003
0.0003	0.0003	0.0003	0.0003	0.0003
1.3377	3.0220	2.6706	0.0003	0.1285
1.6344	1.5050	1.8581	0.3499	0.3681
0.0003	0.0003	0.0003	0.0022	0.0003
0.0362	0.0609	0.0003	0.0003	0.0003
0.0003	0.0003	0.0003	0.0003	0.0003
0.0003	0.0112	0.0137	0.0003	0.0003
141.8565	159.5303	218.3365	22.9435	27.0482
0.2985	0.4778	0.3743	0.0537	0.0504
6.3120	8.2415	7.9193	1.1425	1.2051
2.4779	0.0003	0.0003	0.4038	0.3687
20.7863	24.5302	20.5822	3.9199	4.1546
0.0003	0.0003	0.0003	0.0003	0.0003
0.0003	0.0003	0.0003	0.0003	0.0003
0.0895	0.3691	0.3522	0.0003	0.0003
0.0003	0.0003	0.0003	0.0003	0.0003
8.2823	11.2829	9.0705	1.8459	2.2093
0.0003	0.0003	0.0003	0.0003	0.0003
0.0505	0.0465	0.0660	0.0461	0.0574
2.2282	6.2131	6.0396	1.7325	1.9261
0.0003	0.0003	0.0003	0.0003	0.0003
0.0003	0.0003	0.0003	0.0003	0.0003
0.0417	0.0556	0.0522	0.0003	0.0098
1.0173	1.7675	1.3653	0.1890	0.1960
7.2628	8.5702	9.3217	1.1722	1.0160
0.0586	0.0787	0.0466	0.0444	0.0602
0.0003	0.0003	0.0003	0.0003	0.0003
0.0003	0.0003	0.0003	0.0003	0.0003
0.0563	0.1026	0.0789	0.0003	0.0003
0.1115	0.1850	0.1303	0.1543	0.1535
9.6799	11.2379	12.2228	0.3351	0.3646

0.0003	0.0003	0.0003	0.0003	0.0003
0.0003	0.0003	0.0003	0.0003	0.0003
0.0628	0.0003	0.0687	0.0003	0.0489
0.0277	0.1117	0.0560	0.0291	0.0003
0.0003	0.0003	0.0003	0.0003	0.0003
0.0003	0.0003	0.0003	0.0003	0.0003
0.0003	0.0003	0.0003	0.0003	0.0003
0.0003	0.0003	0.0003	0.0003	0.0003
0.5561	0.6181	0.5840	0.4398	0.4416
0.0003	0.0003	0.0003	0.0003	0.0064
0.0003	0.0003	0.0003	0.0003	0.0003
0.0003	0.0003	0.0003	0.0003	0.0003
0.0003	0.0003	0.0003	0.0003	0.0003
0.0003	0.0003	0.0003	0.0003	0.0003
0.0003	0.0003	0.0003	0.0003	0.0003
0.0362	0.0124	0.0003	0.0003	0.0003
0.5939	0.5831	0.6638	0.3335	0.3847
0.8111	0.8855	0.6700	0.5873	0.6874
0.0131	0.4626	0.0003	0.0003	0.0003
0.0003	0.0003	0.0003	0.0588	0.0884
11.8448	3.9421	5.1213	0.9568	1.4916
0.0003	0.0003	0.0003	0.0003	0.0003
0.0003	0.0003	0.0003	0.0003	0.0003
0.0003	0.0003	0.0003	0.0003	0.0003
0.0003	0.0532	0.0003	0.0003	0.0145
0.0123	0.0003	0.0003	0.0003	0.0003
0.1092	0.0881	0.0873	0.1359	0.1579
0.0003	0.0556	0.0413	0.1042	0.0952
7.1914	2.9043	3.3361	1.6676	1.7692
2.5501	0.6941	0.7840	0.5858	0.6831
1.2537	0.5724	0.6952	0.4128	0.3702
95.9848	100.3322	82.3904	11.0421	10.6937
26.4542	3.3426	2.8399	1.3045	1.2687
68.2170	30.4691	27.1449	5.0416	5.1663

0.0003	0.0003	0.0003	0.4936	0.5924
61.7919	102.8366	102.7018	60.4627	64.7408
10.8016	9.0607	9.4056	0.4582	0.4597
0.0985	0.0550	0.0410	0.0003	0.0003
0.0402	0.0135	0.0003	0.0193	0.0003
0.0003	0.0003	0.0003	0.0003	0.0003
0.9621	0.6536	0.6764	0.0435	0.0003
0.1321	0.0003	0.0590	0.0331	0.0003
0.4981	0.4129	0.4770	0.1084	0.1027
0.2908	0.1110	0.0928	0.1007	0.1340
0.3924	0.3019	0.3154	0.3494	0.3093
0.0261	0.0230	0.0238	0.0256	0.0414
0.1404	0.0752	0.0793	0.0901	0.1286
0.0870	0.0707	0.0561	0.0892	0.1050
0.0003	0.0003	0.0003	0.0717	0.0954
1.5189	1.5617	1.5081	0.3866	0.4609
0.1106	0.0763	0.0460	0.1099	0.1314
0.3722	0.2740	0.2917	0.1829	0.2351
0.1145	0.0529	0.0446	0.0003	0.0003
0.3078	0.1543	0.1625	0.2881	0.2689
0.0276	0.0446	0.0378	0.0186	0.0571
0.0003	0.0192	0.0260	0.0003	0.0274
0.0293	0.0296	0.0357	0.0330	0.0342
0.0003	0.0003	0.0003	0.0073	0.0003
0.0003	0.0003	0.0003	0.0003	0.0186
0.0003	0.0003	0.0003	0.0306	0.0216
0.0003	0.0003	0.0003	0.0003	0.0003
0.0003	0.0003	0.0003	0.0003	0.0003
0.0003	0.0200	0.0223	0.0068	0.0003
0.0003	0.0003	0.0003	0.0003	0.0003
0.0029	0.0023	0.0003	0.0020	0.0003
0.0003	0.0087	0.0068	0.0003	0.0073
0.0003	0.0003	0.0071	0.0003	0.0003
0.0003	0.0003	0.0003	0.0003	0.0003

0.0086	0.0040	0.0003	0.0003	0.0081
0.0044	0.0077	0.0003	0.0037	0.0003
0.0003	0.0193	0.0065	0.0003	0.0003
0.1560	0.0374	0.0342	0.0476	0.0380
0.0347	0.0003	0.0003	0.0003	0.0097
0.6045	0.2128	0.1810	0.1872	0.2009
1.7687	1.8948	1.6688	1.5738	1.7743
0.0256	0.0003	0.0003	0.0111	0.0133
0.0160	0.0122	0.0180	0.0230	0.0242
0.0738	0.1088	0.0003	0.0642	0.1245
0.2609	0.2164	0.0992	0.0912	0.1584
0.0003	0.0139	0.0003	0.0003	0.0003
0.0003	0.0003	0.0003	0.0003	0.0003
0.6509	0.4687	0.4952	0.7592	0.6970
0.0003	0.0003	0.0003	0.0003	0.0003
0.0003	0.2007	0.0003	0.0003	0.0003
1.5063	1.6356	1.4019	1.3751	1.5316
0.0003	0.0003	0.0471	0.0003	0.0003
0.0384	0.0430	0.0352	0.0355	0.0336
0.0003	0.0003	0.0088	0.0003	0.0003
0.0003	0.0003	0.0003	0.0003	0.0003
0.0003	0.0003	0.0003	0.0003	0.0003
0.0003	0.0003	0.0003	0.0003	0.0003
0.0003	0.0003	0.0003	0.0003	0.0003
0.0003	0.0003	0.0003	0.0003	0.0003
0.0003	0.0003	0.0003	0.0003	0.0003
0.0003	0.0003	0.0003	0.0003	0.0003
0.0064	0.0003	0.0065	0.0050	0.0003
0.0003	0.0003	0.0003	0.0003	0.0003
0.0003	0.0003	0.0003	0.0003	0.0003
0.0003	0.0003	0.0003	0.0003	0.0003
0.0003	0.0003	0.0003	0.0003	0.0003
0.0003	0.0003	0.0003	0.0003	0.0003
0.0003	0.0003	0.0003	0.0003	0.0003

0.0003	0.0003	0.0003	0.0003	0.0003
0.0003	0.0003	0.0003	0.0003	0.0003
0.0003	0.0003	0.0003	0.0003	0.0003
0.0003	0.0003	0.0003	0.0003	0.0003
0.0003	0.0003	0.0003	0.0003	0.0003
0.0003	0.0003	0.0003	0.0003	0.0003
0.0003	0.0003	0.0003	0.0003	0.0003
0.0529	0.0794	0.0741	0.0680	0.1144

CO 21 VEST AA 3 165	CO 21 VULV AA 1 166	CO 21 VULV AA 2 167	CO 21 VULV AA 3 168	CO 21 VEST IL + AA 1 169	CO 21 VEST IL + AA 2 170
0.0003	0.0003	0.0003	0.0003	0.0003	0.0003
0.0003	0.0003	0.0003	0.0003	0.0063	0.0065
0.0003	0.0003	0.0003	0.0003	0.0003	0.0003
0.0003	0.0003	0.0003	0.0003	0.3912	0.4013
0.2791	0.5698	0.3797	0.4973	0.4724	0.5434
0.0003	0.0003	0.0003	0.0003	0.0003	0.0003
0.0003	0.0397	0.0003	0.0003	0.0003	0.0514
0.0003	0.0003	0.0003	0.0003	0.0003	0.0003
0.0003	0.0003	0.0003	0.0003	0.0003	0.0003
20.5701	37.6557	33.5841	30.6508	50.7834	48.2206
0.0739	0.0416	0.0507	0.0494	0.0774	0.1437
0.9504	1.5923	1.5001	1.4412	2.1345	2.2777
0.3094	0.6354	0.0003	0.0003	0.8706	0.7727
0.0003	5.2398	4.0261	4.3134	7.4686	6.6212
0.0003	0.0003	0.0003	0.0003	0.0003	0.0003
0.0003	0.0003	0.0003	0.0003	0.0003	0.0003
0.0003	0.0662	0.0855	0.0764	0.0003	0.0003
0.0003	0.0003	0.0003	0.0003	0.0003	0.0003
1.3158	2.5945	2.0237	2.1991	3.2903	3.3163
0.0003	0.0003	0.0003	0.0003	0.0003	0.0003
0.0353	0.0518	0.0316	0.0380	0.0513	0.0416
1.5699	1.6985	1.9848	2.1509	2.0387	2.4450
0.0003	0.0003	0.0003	0.0003	0.0003	0.0003
0.0003	0.0003	0.0003	0.0003	0.0003	0.0003
0.0003	0.0003	0.0003	0.0003	0.0181	0.0148
0.1561	0.2477	0.2190	0.2028	0.4596	0.4201
0.7013	1.4749	1.6342	1.3739	2.5172	2.5084
0.0313	0.0519	0.0640	0.0644	0.0423	0.0472
0.0003	0.0003	0.0003	0.0003	0.0003	0.0003
0.0003	0.0003	0.0003	0.0003	0.0003	0.0003
0.0003	0.0003	0.0285	0.0003	0.0003	0.0003
0.1130	0.2841	0.4028	0.2971	0.2010	0.2163
0.2945	2.3719	2.3062	2.3645	0.6364	0.6411

0.0003	0.0003	0.0003	0.0003	0.0003	0.0003
0.0003	0.0003	0.0003	0.0003	0.0003	0.0003
0.0003	0.0003	0.0597	0.0003	0.0701	0.0003
0.0168	0.0003	0.0003	0.0254	0.0185	0.0003
0.0003	0.0003	0.0003	0.0003	0.0003	0.0003
0.0003	0.0003	0.0021	0.0003	0.0003	0.0003
0.0003	0.0003	0.0003	0.0003	0.0003	0.0003
0.0003	0.0003	0.0003	0.0003	0.0003	0.0003
0.3600	0.4380	0.5393	0.5711	0.3815	0.4088
0.0003	0.0003	0.0003	0.0003	0.0003	0.0003
0.0003	0.0003	0.0003	0.0003	0.0003	0.0003
0.0049	0.0003	0.0114	0.0003	0.0003	0.0072
0.0003	0.0003	0.0003	0.0003	0.0003	0.0003
0.0003	0.0003	0.0003	0.0003	0.0003	0.0003
0.0003	0.0003	0.0003	0.0003	0.0003	0.0003
0.0003	0.0003	0.0114	0.0003	0.0082	0.0003
0.2892	0.3148	0.3575	0.3612	0.3082	0.3009
0.3842	0.5246	0.5408	0.6424	0.4428	0.4945
0.0003	0.0003	0.0694	0.0003	0.0003	0.0003
0.0003	0.0003	0.0003	0.1644	0.1416	0.3372
0.4087	1.2156	1.1115	1.3797	0.3581	1.5028
0.0003	0.0003	0.0003	0.0003	0.0003	0.0003
0.0223	0.0003	0.0003	0.0003	0.0003	0.0330
0.0003	0.0003	0.0003	0.0003	0.0003	0.0003
0.0003	0.0181	0.0003	0.0168	0.0003	0.0003
0.0095	0.0003	0.0003	0.0003	0.0003	0.0003
0.1298	0.1024	0.0667	0.0934	0.1017	0.0874
0.0803	0.0592	0.0430	0.0491	0.0742	0.0793
1.4665	1.6212	1.2988	1.4542	1.1541	1.5191
0.4573	0.6489	0.4970	0.7268	0.4668	0.5969
0.3340	0.6693	0.5033	0.6207	0.3057	0.4347
9.6900	18.3291	12.9661	14.3675	14.4587	16.0728
0.6520	1.9720	1.2652	1.8702	0.7758	0.8592
3.3690	4.0469	2.9490	3.7842	4.2876	4.2887

0.2270	0.4606	0.3295	0.4972	0.1197	0.1920
56.7534	88.4173	81.5557	103.9784	70.4606	73.3416
0.3995	2.4293	1.9742	1.3005	0.2405	0.5390
0.0003	0.0003	0.0003	0.0003	0.0003	0.0003
0.0003	0.0003	0.0003	0.0003	0.0003	0.0003
0.0003	0.0003	0.0003	0.0003	0.0003	0.0003
0.0003	0.2067	0.1559	0.1720	0.0617	0.0608
0.0003	0.0539	0.0373	0.0915	0.0003	0.0003
0.0476	0.0949	0.0821	0.1097	0.0003	0.0237
0.1093	0.1463	0.0895	0.1173	0.0566	0.0003
0.3282	0.1864	0.2043	0.1969	0.2377	0.2361
0.0342	0.0162	0.0162	0.0261	0.0337	0.0354
0.0948	0.0540	0.0529	0.0722	0.0917	0.0506
0.0856	0.0623	0.0752	0.0697	0.0765	0.0615
0.0518	0.0496	0.0756	0.1275	0.0745	0.0651
0.2224	0.6867	0.5242	0.6176	0.3814	0.4507
0.0808	0.0854	0.0003	0.0687	0.0383	0.0949
0.1682	0.1857	0.1490	0.1793	0.1344	0.1348
0.0317	0.0003	0.0003	0.0003	0.0003	0.0003
0.2409	0.2556	0.1824	0.2237	0.2017	0.1996
0.0296	0.0321	0.0190	0.0483	0.0501	0.0286
0.0200	0.0123	0.0003	0.0296	0.0310	0.0251
0.0271	0.0204	0.0229	0.0256	0.0230	0.0312
0.0003	0.0003	0.0003	0.0003	0.0003	0.0003
0.0003	0.0003	0.0003	0.0003	0.0003	0.0003
0.0161	0.0003	0.0003	0.0192	0.0214	0.0003
0.0003	0.0003	0.0003	0.0003	0.0003	0.0003
0.0003	0.0003	0.0003	0.0003	0.0003	0.0003
0.0003	0.0003	0.0003	0.0003	0.0003	0.0003
0.0003	0.0003	0.0003	0.0003	0.0003	0.0003
0.0003	0.0026	0.0003	0.0003	0.0003	0.0003
0.0045	0.0003	0.0044	0.0059	0.0062	0.0003
0.0003	0.0003	0.0003	0.0003	0.0003	0.0003
0.0003	0.0003	0.0003	0.0003	0.0003	0.0003

0.0003	0.0003	0.0003	0.0003	0.0003	0.0003
0.0003	0.0047	0.0003	0.0066	0.0003	0.0003
0.0003	0.0003	0.0003	0.0076	0.0063	0.0003
0.0219	0.0337	0.0216	0.0348	0.0179	0.0188
0.0003	0.0003	0.0086	0.0003	0.0048	0.0048
0.1332	0.2641	0.2319	0.2967	0.0655	0.0915
1.2589	1.4521	1.6243	2.1360	1.2583	1.3833
0.0003	0.0003	0.0099	0.0003	0.0003	0.0003
0.0148	0.0131	0.0167	0.0151	0.0158	0.0199
0.1223	0.1189	0.0968	0.2401	0.1135	0.2095
0.1266	0.1676	0.0751	0.2932	0.2375	0.2988
0.0003	0.0003	0.0003	0.0003	0.0003	0.0003
0.0003	0.0003	0.0003	0.0003	0.0003	0.0003
0.5270	0.6633	0.4754	0.5166	0.5725	0.5517
0.0003	0.0003	0.0003	0.0003	0.0003	0.0003
0.0003	0.0003	0.0003	0.0003	0.0003	0.0003
1.0831	0.8024	1.2001	1.4712	0.9457	1.8329
0.0003	0.0003	0.0003	0.0003	0.0003	0.0003
0.0279	0.0384	0.0364	0.0451	0.0307	0.0384
0.0003	0.0003	0.0075	0.0047	0.0003	0.0003
0.0003	0.0003	0.0003	0.0003	0.0003	0.0003
0.0003	0.0003	0.0003	0.0003	0.0003	0.0003
0.0003	0.0003	0.0003	0.0003	0.0003	0.0003
0.0003	0.0003	0.0003	0.0003	0.0003	0.0003
0.0003	0.0003	0.0156	0.0003	0.0003	0.0003
0.0003	0.0003	0.0003	0.0003	0.0003	0.0003
0.0003	0.0003	0.0003	0.0003	0.0003	0.0003
0.0037	0.0003	0.0051	0.0065	0.0003	0.0003
0.0003	0.0003	0.0003	0.0003	0.0003	0.0003
0.0003	0.0003	0.0003	0.0003	0.0003	0.0003
0.0003	0.0003	0.0003	0.0003	0.0003	0.0003
0.0003	0.0003	0.0003	0.0003	0.0003	0.0003
0.0003	0.0003	0.0003	0.0003	0.0003	0.0003
0.0003	0.0003	0.0003	0.0003	0.0003	0.0003

0.0003	0.0003	0.0003	0.0003	0.0003	0.0003
0.0003	0.0003	0.0003	0.0003	0.0003	0.0003
0.0003	0.0003	0.0003	0.0003	0.0003	0.0003
0.0003	0.0003	0.0003	0.0003	0.0003	0.0003
0.0003	0.0003	0.0003	0.0003	0.0003	0.0003
0.0003	0.0003	0.0003	0.0003	0.0003	0.0003
0.0003	0.0003	0.0003	0.0003	0.0003	0.0003
0.0528	0.0673	0.0749	0.0846	0.0533	0.0619

CO 21 VEST IL + AA 3 171	CO 21 VULV IL + AA 1 172	CO 21 VULV IL + AA 2 173	CO 21 VULV IL + AA 3 174	CO 21 VEST POLY IC + AA 1 175
0.0003	0.0003	0.0003	0.0003	0.0003
0.0003	0.0003	0.0226	0.0131	0.0003
0.0003	0.0003	0.0003	0.0003	0.0003
0.2960	1.0458	0.9267	0.6639	0.8498
0.4269	1.3335	1.1838	1.0639	0.7043
0.0003	0.0003	0.0003	0.0003	0.0003
0.0372	0.0003	0.0347	0.0003	0.0500
0.0003	0.0003	0.0003	0.0003	0.0003
0.0003	0.0054	0.0003	0.0003	0.0003
39.2664	105.2333	103.8878	92.4677	59.8417
0.0686	0.1426	0.1142	0.0710	0.2492
1.7556	5.8100	4.9335	4.3247	2.4977
0.0003	0.0003	0.0003	0.0003	0.0003
6.0536	18.9692	19.6248	18.3463	9.6045
0.0003	0.0003	0.0003	0.0003	0.0003
0.0003	0.0003	0.0003	0.0003	0.0003
0.0003	0.2541	0.2290	0.1902	0.0003
0.0003	0.0003	0.0003	0.0003	0.0003
2.7391	8.7670	8.1014	7.8758	4.3122
0.0003	0.0003	0.0003	0.0003	0.0003
0.0489	0.0433	0.0463	0.0460	0.0554
1.8946	2.3088	1.8188	2.0696	2.3971
0.0003	0.0003	0.0003	0.0003	0.0003
0.0003	0.0003	0.0003	0.0003	0.0003
0.0114	0.0346	0.0382	0.0221	0.0295
0.3168	1.8740	1.5869	1.1429	0.5645
1.9323	6.1162	5.3889	5.4091	3.1703
0.0405	0.0671	0.0462	0.0548	0.1323
0.0003	0.0003	0.0003	0.0003	0.0003
0.0003	0.0003	0.0003	0.0003	0.0003
0.0003	0.0003	0.0747	0.0651	0.0003
0.1427	0.4053	0.3797	0.2860	0.1410
0.4706	7.0146	4.3983	4.0439	0.7266

0.0003	0.0003	0.0003	0.0003	0.0003
0.0003	0.0003	0.0003	0.0003	0.0003
0.0421	0.1062	0.0577	0.0510	0.0467
0.0003	0.0003	0.0003	0.0003	0.0220
0.0003	0.0003	0.0003	0.0003	0.0003
0.0003	0.0003	0.0003	0.0003	0.0003
0.0003	0.0003	0.0003	0.0003	0.0003
0.0003	0.0003	0.0003	0.0003	0.0003
0.3346	0.4241	0.3771	0.4795	0.4513
0.0003	0.0003	0.0003	0.0003	0.0003
0.0003	0.0084	0.0054	0.0119	0.0003
0.0003	0.0078	0.0003	0.0003	0.0003
0.0003	0.0003	0.0003	0.3699	0.0003
0.0003	0.0003	0.0003	0.0003	0.0003
0.0003	0.0003	0.0003	0.0003	0.0003
0.0044	0.0003	0.0086	0.0003	0.0003
0.2872	0.3759	0.2792	0.3085	0.3526
0.4268	0.4045	0.3621	0.4724	0.6217
0.0003	0.0003	0.0003	0.0003	0.3557
0.0718	0.1128	0.0003	0.0003	0.1373
0.2565	0.2551	0.4070	0.3483	0.7228
0.0003	0.0003	0.0003	0.0003	0.0003
0.0003	0.0003	0.0003	0.0003	0.0003
0.0003	0.0003	0.0003	0.0003	0.0003
0.0003	0.0003	0.0003	0.0165	0.0216
0.0003	0.0025	0.0003	0.0003	0.0003
0.0738	0.0627	0.0610	0.0336	0.1092
0.0620	0.0322	0.0388	0.0307	0.0910
1.1025	1.0843	1.0853	1.0028	1.7704
0.4299	0.3547	0.2840	0.3979	0.7209
0.3051	0.3405	0.3050	0.2905	0.5089
12.0960	23.6109	25.0423	20.0369	32.7792
0.6595	0.6769	0.6104	0.6579	1.2155
3.6928	4.9455	5.6339	3.4428	9.8611

0.1592	0.0842	0.0003	0.0003	0.0003
63.7185	82.4334	80.4568	92.7113	83.3282
0.5572	1.2505	1.1396	1.1478	0.5163
0.0003	0.0003	0.0003	0.0003	0.0003
0.0003	0.0003	0.0003	0.0003	0.0003
0.0003	0.0003	0.0003	0.0003	0.0003
0.0003	0.1320	0.1502	0.0909	0.0003
0.0003	0.0003	0.0003	0.0003	0.0003
0.0003	0.0398	0.0407	0.0514	0.0428
0.0003	0.0003	0.0003	0.0003	0.0614
0.1767	0.1206	0.1255	0.0979	0.2529
0.0182	0.0188	0.0003	0.0003	0.0428
0.0730	0.0391	0.0624	0.0341	0.1081
0.0415	0.0382	0.0003	0.0469	0.0699
0.0689	0.0003	0.0421	0.0447	0.0893
0.3077	0.3804	0.4158	0.2902	0.7097
0.0580	0.0003	0.0003	0.0366	0.1083
0.1391	0.1464	0.1582	0.0977	0.1382
0.0003	0.0003	0.0003	0.0003	0.0003
0.1930	0.0962	0.1209	0.1092	0.1986
0.0462	0.0433	0.0501	0.0085	0.0275
0.0348	0.0303	0.0332	0.0189	0.0224
0.0176	0.0189	0.0191	0.0114	0.0258
0.0071	0.0003	0.0003	0.0003	0.0082
0.0003	0.0003	0.0003	0.0003	0.0003
0.0179	0.0003	0.0003	0.0003	0.0003
0.0003	0.0003	0.0003	0.0003	0.0003
0.0003	0.0003	0.0003	0.0003	0.0003
0.0003	0.0121	0.0003	0.0003	0.0003
0.0003	0.0003	0.0003	0.0003	0.0003
0.0003	0.0003	0.0003	0.0003	0.0003
0.0047	0.0069	0.0051	0.0003	0.0003
0.0003	0.0003	0.0003	0.0003	0.0003
0.0003	0.0003	0.0003	0.0003	0.0003

0.0003	0.0003	0.0003	0.0003	0.0003
0.0084	0.0003	0.0003	0.0023	0.0047
0.0003	0.0003	0.0003	0.0049	0.0003
0.0112	0.0171	0.0135	0.0131	0.0278
0.0003	0.0003	0.0003	0.0031	0.0003
0.0774	0.1097	0.0742	0.1129	0.1200
1.3299	1.2935	1.3288	1.4021	1.4804
0.0003	0.0003	0.0003	0.0003	0.0003
0.0148	0.0093	0.0064	0.0082	0.0177
0.0707	0.1159	0.1645	0.0003	0.1705
0.2177	0.1215	0.2412	0.1157	0.2568
0.0003	0.0003	0.0003	0.0003	0.0003
0.0003	0.0003	0.0003	0.0003	0.0003
0.5468	0.5062	0.5247	0.4625	0.8052
0.0003	0.0003	0.0003	0.0003	0.0003
0.0003	0.0003	0.0003	0.0003	0.2311
1.5333	1.4235	1.3746	1.4660	1.6781
0.0003	0.0523	0.0003	0.0518	0.0003
0.0334	0.0385	0.0321	0.0380	0.0353
0.0003	0.0003	0.0003	0.0003	0.0003
0.0003	0.0003	0.0003	0.0003	0.0003
0.0003	0.0003	0.0003	0.0003	0.0003
0.0003	0.0003	0.0003	0.0003	0.0003
0.0003	0.0003	0.0003	0.0003	0.0003
0.0003	0.0003	0.0003	0.0003	0.0003
0.0003	0.0003	0.0003	0.0003	0.0003
0.0003	0.0003	0.0003	0.0003	0.0003
0.0003	0.0003	0.0046	0.0031	0.0049
0.0003	0.0003	0.0003	0.0003	0.0003
0.0003	0.0003	0.0003	0.0003	0.0003
0.0003	0.0003	0.0003	0.0003	0.0003
0.0003	0.0003	0.0003	0.0003	0.0003
0.0003	0.0003	0.0003	0.0003	0.0003
0.0003	0.0003	0.0003	0.0003	0.0003

0.0003	0.0003	0.0003	0.0003	0.0003
0.0003	0.0003	0.0003	0.0003	0.0003
0.0003	0.0003	0.0003	0.0003	0.0003
0.0003	0.0003	0.0003	0.0003	0.0003
0.0003	0.0003	0.0003	0.0003	0.0003
0.0003	0.0003	0.0003	0.0003	0.0003
0.0003	0.0003	0.0003	0.0203	0.0003
0.0540	0.0475	0.0533	0.0496	0.0649

CO 21 VEST POLY IC + AA 2 176	CO 21 VEST POLY IC + AA 3 177	CO 21 VULV POLY IC + AA 1 178	CO 21 VULV POLY IC + AA 2 179
0.0003	0.0003	0.0003	0.0003
0.0003	0.0003	0.0309	0.0434
0.0003	0.0003	0.0003	0.0003
0.4773	0.5885	4.2978	5.0615
0.4756	0.5617	1.9739	1.8682
0.0003	0.0003	0.0089	0.0095
0.0003	0.0003	0.0003	0.0003
0.0049	0.0003	0.0003	0.0003
0.0003	0.0003	0.0143	0.0108
49.0631	52.1605	2811.0485	144.3045
0.1850	0.2045	0.5851	0.8200
2.2152	2.0492	9.6363	9.9940
0.5608	0.0003	0.0003	0.0003
5.3668	6.1987	32.0798	26.8988
0.0003	0.0003	0.0003	0.0003
0.0003	0.0003	0.0003	0.0003
0.0003	0.0003	0.6067	0.8207
0.0003	0.0003	0.0003	0.0003
2.5130	2.7244	14.2161	11.4096
0.0003	0.0003	0.0003	0.0003
0.0362	0.0473	0.0634	0.0429
2.1557	2.0322	5.6732	6.9461
0.0474	0.0003	0.0003	0.1464
0.0003	0.0003	0.0003	0.0003
0.0177	0.0207	0.1447	0.1474
0.3159	0.3893	2.3779	2.2901
2.2826	2.5026	12.2089	12.0437
0.0496	0.1341	0.0562	0.0489
0.0003	0.0003	0.0003	0.0003
0.0003	0.0003	0.0003	0.0003
0.0003	0.0003	0.1201	0.1673
0.1337	0.1000	0.2032	0.2621
0.5219	0.4765	5.5370	9.0153

0.0003	0.0003	0.0003	0.0003
0.0003	0.0003	0.0003	0.0003
0.0389	0.0513	0.0440	0.0003
0.0458	0.0220	0.1313	0.1288
0.0003	0.0003	0.0003	0.0003
0.0003	0.0003	0.0003	0.0003
0.0003	0.0003	0.0003	0.0003
0.0003	0.0003	0.0003	0.0003
0.4641	0.3682	0.5352	0.5218
0.0003	0.0003	0.0068	0.0003
0.0003	0.0003	0.0003	0.0083
0.0003	0.0003	0.0003	0.0028
0.0003	0.0003	0.0003	0.0003
0.0003	0.0003	0.0003	0.0003
0.0003	0.0003	0.0003	0.0003
0.0003	0.0003	0.0003	0.0003
0.3459	0.2274	0.4546	0.4207
0.5385	0.4176	0.7319	0.7196
0.0003	0.1530	0.1020	0.0003
0.0003	0.0792	0.0003	0.0003
0.4404	0.4079	1.6834	0.7925
0.0003	0.0003	0.0003	0.0003
0.0003	0.0003	0.0003	0.0003
0.0003	0.0003	0.0003	0.0003
0.0003	0.0003	0.1082	0.0003
0.0003	0.0003	0.0057	0.0003
0.1043	0.0780	0.0725	0.0840
0.0845	0.0764	0.0476	0.0490
1.4077	1.2474	1.5286	1.4687
0.4409	0.3823	0.4806	0.3670
0.3214	0.2607	0.4085	0.4337
27.6067	28.8018	116.2636	127.0834
0.6168	0.5511	1.4154	0.6626
8.0298	7.9568	31.2066	32.5678

0.0003	0.0003	0.0003	0.0003
59.2215	48.4941	75.7401	87.8707
0.5071	0.2391	4.7349	3.4554
0.0207	0.0003	0.0003	0.0003
0.0003	0.0003	0.0003	0.0003
0.0003	0.0003	0.0003	0.0233
0.0986	0.0905	1.0409	1.1758
0.0003	0.0003	0.0003	0.0003
0.0273	0.0003	0.4253	0.2830
0.0003	0.0003	0.0736	0.0531
0.2196	0.1976	0.1728	0.2067
0.0147	0.0401	0.0003	0.0003
0.0653	0.0699	0.0500	0.0577
0.0788	0.0564	0.0775	0.0003
0.0692	0.0463	0.0003	0.0003
0.6003	0.4767	2.8204	2.7824
0.0003	0.0003	0.0836	0.0574
0.1288	0.1400	0.2740	0.2110
0.0003	0.0003	0.0003	0.0627
0.1665	0.1372	0.1415	0.1605
0.0003	0.0368	0.0003	0.0180
0.0184	0.0003	0.0246	0.0003
0.0256	0.0235	0.0234	0.0255
0.0057	0.0003	0.0003	0.0003
0.0003	0.0003	0.0003	0.0003
0.0146	0.0316	0.0003	0.0003
0.0003	0.0003	0.0003	0.0003
0.0003	0.0003	0.0003	0.0003
0.0120	0.0092	0.0278	0.0176
0.0003	0.0003	0.0003	0.0003
0.0003	0.0003	0.0003	0.0003
0.0041	0.0049	0.0068	0.0061
0.0003	0.0046	0.0003	0.0003
0.0003	0.0003	0.0003	0.0003

0.0003	0.0003	0.0003	0.0003
0.0003	0.0003	0.0003	0.0003
0.0003	0.0003	0.0091	0.0090
0.0109	0.0193	0.0265	0.0191
0.0003	0.0003	0.0003	0.0003
0.0638	0.0539	0.1854	0.1204
1.5580	1.0225	1.6003	1.6139
0.0003	0.0003	0.0003	0.0003
0.0185	0.0130	0.0134	0.0118
0.1350	0.1171	0.1049	0.1685
0.1607	0.2317	0.0003	0.1968
0.0003	0.0263	0.0160	0.0003
0.0003	0.0003	0.0003	0.0003
0.5227	0.4166	0.4840	0.4380
0.0003	0.0003	0.0003	0.0003
0.0003	0.0003	0.0003	0.0003
1.5743	1.0807	1.4229	1.7144
0.0003	0.0003	0.0003	0.0003
0.0302	0.0320	0.0407	0.0221
0.0039	0.0003	0.0003	0.0003
0.0003	0.0003	0.0003	0.0003
0.0003	0.0003	0.0003	0.0003
0.0003	0.0003	0.0003	0.0003
0.0003	0.0003	0.0003	0.0003
0.0003	0.0003	0.0003	0.0003
0.0003	0.0003	0.0003	0.0003
0.0003	0.0003	0.0003	0.0003
0.0039	0.0035	0.0046	0.0003
0.0003	0.0003	0.0003	0.0003
0.0003	0.0003	0.0003	0.0003
0.0003	0.0003	0.0003	0.0003
0.0003	0.0003	0.0003	0.0003
0.0003	0.0003	0.0003	0.0003
0.0003	0.0003	0.0003	0.0003

0.0003	0.0003	0.0289	0.0296
0.0003	0.0003	0.0003	0.0003
0.0003	0.0003	0.0003	0.0003
0.0003	0.0003	0.0003	0.0003
0.0003	0.0003	0.0003	0.0003
0.0003	0.0003	0.0003	0.0003
0.0003	0.0003	0.0003	0.0003
0.0504	0.0315	0.0617	0.0652

CO 21 VULV POLY IC + AA 3 180	CO 23 VEST AA 1 199	CO 23 VEST AA 2 200	CO 23 VEST AA 3 201	CO 23 VULV AA 1 202	CO 23 VULV AA 2 203
0.0003	0.0003	0.0003	0.0003	0.0003	0.0003
0.0359	0.0003	0.0003	0.0003	0.0003	0.0003
0.0003	0.0003	0.0003	0.0003	0.0003	0.0003
6.4515	0.1703	0.0003	0.0003	0.0003	0.0003
1.8461	0.4015	0.5397	0.4028	0.4329	0.4334
0.0063	0.0003	0.0003	0.0003	0.0003	0.0003
0.0796	0.0003	0.0003	0.0003	0.0360	0.0003
0.0003	0.0003	0.0003	0.0003	0.0003	0.0003
0.0003	0.0003	0.0003	0.0003	0.0003	0.0003
119.7472	33.5287	37.0611	38.9080	32.9954	30.2252
0.7703	0.0396	0.0664	0.0811	0.0414	0.0731
10.6767	1.4484	1.4433	1.7365	1.3200	1.3620
0.0003	0.0003	0.0003	0.3750	0.5538	0.1562
28.7794	4.4045	3.4672	3.4358	4.9868	3.6191
0.0003	0.0003	0.0003	0.0003	0.0003	0.0003
0.0003	0.0003	0.0003	0.0003	0.0003	0.0003
0.7811	0.0786	0.0915	0.0795	0.0003	0.0003
0.0003	0.0003	0.0003	0.0003	0.0003	0.0003
13.4709	2.2601	1.5316	1.3977	2.1203	1.5788
0.0003	0.0003	0.0003	0.0003	0.0003	0.0003
0.0555	0.0527	0.0467	0.0413	0.0438	0.0333
7.9220	1.9674	2.3764	2.1901	2.1143	1.7613
0.0003	0.0003	0.0003	0.0003	0.0003	0.0003
0.0003	0.0003	0.0003	0.0003	0.0003	0.0003
0.2265	0.0003	0.0003	0.0003	0.0003	0.0003
2.9048	0.3397	0.2828	0.3278	0.1311	0.1855
12.7664	1.4845	1.5022	1.7830	1.2906	1.1716
0.0615	0.0534	0.0352	0.0439	0.0566	0.0564
0.0003	0.0003	0.0003	0.0003	0.0003	0.0003
0.0003	0.0003	0.0003	0.0003	0.0003	0.0003
0.1744	0.0003	0.0003	0.0003	0.0003	0.0003
0.2432	0.1651	0.1821	0.1596	0.4062	0.4289
9.0563	1.2347	1.2599	1.1410	2.5759	2.7835

0.0003	0.0003	0.0003	0.0003	0.0003	0.0003
0.0003	0.0003	0.0003	0.0003	0.0003	0.0003
0.1301	0.0626	0.0767	0.0893	0.0511	0.0003
0.1482	0.0003	0.0213	0.0003	0.0003	0.0208
0.0003	0.0003	0.0003	0.0003	0.0003	0.0003
0.0003	0.0003	0.0003	0.0003	0.0003	0.0003
0.0003	0.0003	0.0003	0.0003	0.0003	0.0003
0.0003	0.0003	0.0003	0.0003	0.0003	0.0003
0.6035	0.4437	0.4502	0.4395	0.3490	0.3656
0.0003	0.0003	0.0003	0.0112	0.0003	0.0003
0.0059	0.0003	0.0003	0.0003	0.0003	0.0003
0.0003	0.0003	0.0121	0.0228	0.0003	0.0341
0.0003	0.0003	0.0003	0.0003	0.0003	0.0003
0.0003	0.0003	0.0003	0.0003	0.0003	0.0003
0.0003	0.0003	0.0003	0.0003	0.0003	0.0003
0.0003	0.0003	0.0003	0.0003	0.0003	0.0003
0.4557	0.4259	0.3617	0.3624	0.2038	0.1834
0.7194	0.6938	0.4898	0.5433	0.2540	0.2198
0.0003	0.0078	0.0003	0.0003	0.0104	0.0003
0.0003	0.1738	0.1125	0.1416	0.0003	0.1352
0.7341	7.7655	6.4694	4.6497	0.1710	0.8093
0.0003	0.0003	0.0003	0.0003	0.0003	0.0003
0.0003	0.0003	0.0003	0.0608	0.0003	0.0403
0.0003	0.0003	0.0003	0.0003	0.0003	0.0003
0.0860	0.0158	0.0196	0.0003	0.0003	0.0003
0.0003	0.0003	0.0003	0.0088	0.0003	0.0003
0.0695	0.1026	0.1376	0.1341	0.0605	0.0991
0.0422	0.0517	0.0643	0.0600	0.0311	0.0405
1.3398	1.6946	1.9576	1.9773	1.3247	1.8608
0.3443	0.8401	0.8133	0.6381	0.7454	0.8138
0.4187	0.5098	0.5883	0.4012	0.6553	0.6956
118.6253	22.1264	22.3847	22.6323	15.8898	17.5819
0.6233	6.0931	4.1522	2.8661	0.7972	0.7981
29.1603	11.2258	7.6244	6.1914	3.6435	4.4612

0.0003	0.7669	0.9201	0.4605	0.0003	0.0003
82.3493	58.8766	57.3338	51.6780	75.6278	75.5571
3.0717	1.7334	1.5234	1.2139	0.7705	1.4666
0.0003	0.0003	0.0746	0.0003	0.0003	0.0390
0.0003	0.0003	0.0174	0.0182	0.0003	0.0003
0.0003	0.0003	0.0003	0.0003	0.0003	0.0003
0.9463	0.1648	0.1971	0.1699	0.1233	0.1204
0.0003	0.0872	0.0515	0.0591	0.0003	0.0003
0.3036	0.1394	0.1473	0.1727	0.0328	0.0438
0.0614	0.1635	0.1498	0.1180	0.0003	0.0003
0.1665	0.3128	0.3688	0.3613	0.2217	0.2548
0.0003	0.0196	0.0242	0.0145	0.0191	0.0359
0.0298	0.0898	0.1181	0.0745	0.0738	0.0863
0.0365	0.1070	0.1039	0.0968	0.0980	0.0739
0.0003	0.0974	0.0988	0.0832	0.0513	0.0902
2.6693	0.8647	0.8981	0.8322	0.3812	0.4569
0.0412	0.1183	0.1888	0.0003	0.0696	0.0665
0.2469	0.2645	0.2783	0.2462	0.1519	0.1668
0.0697	0.0003	0.0003	0.0547	0.0341	0.0003
0.1367	0.5484	0.4447	0.4019	0.1786	0.1917
0.0309	0.0376	0.0279	0.0232	0.0003	0.0236
0.0168	0.0003	0.0003	0.0159	0.0003	0.0003
0.0273	0.0349	0.0361	0.0370	0.0347	0.0562
0.0064	0.0003	0.0003	0.0003	0.0003	0.0003
0.0003	0.0003	0.0003	0.0003	0.0003	0.0461
0.0003	0.0003	0.0003	0.0003	0.0274	0.0259
0.0020	0.0003	0.0003	0.0003	0.0003	0.0003
0.0003	0.0003	0.0003	0.0003	0.0003	0.0003
0.0186	0.0003	0.0003	0.0003	0.0003	0.0003
0.0003	0.0003	0.0003	0.0003	0.0003	0.0003
0.0003	0.0020	0.0003	0.0003	0.0003	0.0003
0.0003	0.0076	0.0003	0.0084	0.0003	0.0049
0.0003	0.0003	0.0003	0.0003	0.0003	0.0003
0.0003	0.0003	0.0003	0.0003	0.0003	0.0003

0.0003	0.0135	0.0081	0.0085	0.0003	0.0003
0.0003	0.0003	0.0003	0.0060	0.0003	0.0003
0.0051	0.0086	0.0084	0.0003	0.0003	0.0003
0.0063	0.0568	0.0441	0.0393	0.0174	0.0294
0.0003	0.0185	0.0103	0.0089	0.0003	0.0003
0.1324	0.3821	0.3601	0.2922	0.0974	0.1159
1.6973	1.4417	1.4232	1.5697	1.1813	1.4291
0.0003	0.0063	0.0120	0.0003	0.0003	0.0003
0.0159	0.0271	0.0313	0.0274	0.0003	0.0082
0.1903	0.1223	0.0003	0.2105	0.1199	0.0705
0.3024	0.0003	0.1844	0.1245	0.1565	0.2333
0.0003	0.0145	0.0003	0.0003	0.0003	0.0003
0.0003	0.0003	0.0003	0.0003	0.0003	0.0003
0.3516	0.6775	0.6260	0.5775	0.4965	0.5665
0.0003	0.0003	0.0003	0.0003	0.0003	0.0003
0.0003	0.0003	0.0003	0.0003	0.0003	0.0003
1.5630	1.2289	1.2536	1.4085	1.1692	1.2757
0.0003	0.0003	0.0003	0.0534	0.0374	0.0003
0.0288	0.0264	0.0278	0.0272	0.0298	0.0302
0.0003	0.0003	0.0003	0.0003	0.0003	0.0003
0.0003	0.0003	0.0003	0.0003	0.0003	0.0003
0.0003	0.0003	0.0206	0.0003	0.0003	0.0003
0.0003	0.0003	0.0003	0.0003	0.0003	0.0003
0.0003	0.0003	0.0003	0.0003	0.0003	0.0003
0.0003	0.0003	0.0003	0.0133	0.0003	0.0134
0.0003	0.0003	0.0003	0.0003	0.0003	0.0003
0.0003	0.0003	0.0003	0.0003	0.0003	0.0003
0.0003	0.0003	0.0124	0.0089	0.0003	0.0090
0.0003	0.0003	0.0003	0.0003	0.0003	0.0003
0.0003	0.0003	0.0003	0.0003	0.0003	0.0003
0.0003	0.0003	0.0064	0.0003	0.0003	0.0003
0.0003	0.0003	0.0003	0.0003	0.0003	0.0003
0.0047	0.0003	0.0003	0.0003	0.0003	0.0003
0.0003	0.0003	0.0003	0.0003	0.0003	0.0003

0.0293	0.0003	0.0003	0.0003	0.0003	0.0003
0.0003	0.0003	0.0003	0.0003	0.0003	0.0003
0.0003	0.0003	0.0003	0.0003	0.0003	0.0003
0.0003	0.0003	0.0003	0.0003	0.0003	0.0003
0.0003	0.0003	0.0003	0.0003	0.0003	0.0003
0.0157	0.0003	0.0003	0.0003	0.0003	0.0213
0.0003	0.0003	0.0003	0.0003	0.0003	0.0003
0.0668	0.1299	0.1361	0.1248	0.0640	0.0703

CO 23 VULV AA 3 204	CO 23 VEST IL + AA 1 205	CO 23 VEST IL + AA 2 206	CO 23 VEST IL + AA 3 207	CO 23 VULV IL + AA 1 208
0.0003	0.0003	0.0003	0.0003	0.0003
0.0003	0.0138	0.0151	0.0003	0.0247
0.0003	0.0003	0.0003	0.0003	0.0003
0.0003	0.7008	0.5942	0.5311	0.8904
0.5425	1.2707	0.9513	0.8245	1.2244
0.0003	0.0003	0.0003	0.0003	0.0003
0.0003	0.0003	0.0003	0.0003	0.0516
0.0003	0.0003	0.0003	0.0003	0.0003
0.0003	0.0003	0.0003	0.0003	0.0003
34.5548	95.4034	86.1373	84.6647	110.1927
0.0876	0.1323	0.0963	0.1226	0.1137
1.5033	4.8912	3.9650	4.3046	4.9024
0.0003	0.0003	1.4808	0.0003	0.0003
3.7631	17.4533	12.3641	10.1280	26.3069
0.0003	0.0003	0.0003	0.0003	0.0003
0.0003	0.0003	0.0003	0.0003	0.0003
0.0683	0.0003	0.0003	0.1520	0.1895
0.0003	0.0003	0.0003	0.0003	0.3891
2.0596	7.2468	5.6075	4.3849	11.8381
0.2420	0.0003	0.0003	0.0003	0.0003
0.0470	0.0688	0.0584	0.0551	0.0517
2.0739	2.5347	1.9508	2.2866	2.0879
0.0003	0.0003	0.0003	0.0003	0.0003
0.0003	0.0003	0.0003	0.0003	0.0003
0.0003	0.0185	0.0143	0.0170	0.0235
0.1338	0.8963	0.7392	0.7512	1.7495
1.4228	5.6971	4.3724	4.1539	5.6759
0.0514	0.0655	0.0362	0.0577	0.0617
0.0003	0.0003	0.0003	0.0003	0.0003
0.0003	0.0003	0.0003	0.0003	0.0003
0.0003	0.0594	0.0528	0.0393	0.1015
0.2815	0.2426	0.1881	0.1830	0.4113
2.5698	3.3528	2.6237	3.2051	7.6833

0.0003	0.0003	0.0003	0.0003	0.0003
0.0003	0.0003	0.0003	0.0003	0.0003
0.0790	0.0974	0.0714	0.1493	0.1235
0.0285	0.0003	0.0323	0.0245	0.0003
0.0003	0.0003	0.0003	0.0003	0.0003
0.0003	0.0003	0.0003	0.0003	0.0003
0.0003	0.0003	0.0003	0.0003	0.0003
0.0003	0.0003	0.0003	0.0003	0.0003
0.4492	0.4911	0.4116	0.4954	0.2568
0.0003	0.0003	0.0003	0.0003	0.0003
0.0003	0.0003	0.0003	0.0003	0.0003
0.0138	0.0003	0.0003	0.0368	0.0003
0.0003	0.0003	0.0003	0.0003	0.0003
0.0003	0.0003	0.0003	0.0003	0.0003
0.0003	0.0003	0.0003	0.0003	0.0018
0.0003	0.0135	0.0003	0.0065	0.0003
0.2828	0.4732	0.3820	0.4308	0.1391
0.3823	0.6125	1.1026	0.4653	0.1662
0.0003	0.0003	0.0003	0.0102	0.0101
0.0003	0.1310	0.0882	0.1243	0.0789
0.3189	5.9398	3.5417	3.1387	0.6593
0.0003	0.0003	0.0003	0.0003	0.0003
0.0003	0.0774	0.0003	0.0003	0.0330
0.0003	0.0003	0.0003	0.0003	0.0003
0.0003	0.0182	0.0003	0.0003	0.0003
0.0003	0.0003	0.0003	0.0003	0.0003
0.1293	0.0724	0.0497	0.0703	0.0408
0.0366	0.0442	0.0316	0.0402	0.0292
2.2619	1.5484	1.2263	1.4904	1.2825
1.2716	0.4809	0.3146	0.4080	0.4197
1.2209	0.3050	0.1930	0.2075	0.3947
19.6321	24.2393	19.3706	26.5248	19.5451
1.1222	1.5187	0.9720	1.1184	0.6233
4.2457	6.7944	4.8108	6.8096	3.8528

0.0003	0.2579	0.1973	0.0003	0.0003
111.3216	73.4199	56.0611	63.6092	74.3399
1.7823	2.8682	1.2526	1.9755	0.6820
0.0648	0.0244	0.0003	0.0003	0.0003
0.0435	0.0003	0.0003	0.0003	0.0003
0.0003	0.0003	0.0003	0.0003	0.0003
0.0968	0.1080	0.1102	0.1817	0.1102
0.0387	0.0003	0.0003	0.0279	0.0003
0.0418	0.1317	0.0889	0.1165	0.0003
0.0529	0.0769	0.0552	0.0706	0.0394
0.3092	0.2527	0.1723	0.2736	0.1839
0.0196	0.0225	0.0106	0.0322	0.0116
0.0945	0.0329	0.0535	0.0793	0.0566
0.0960	0.0845	0.0508	0.0794	0.0628
0.1306	0.0430	0.0452	0.0584	0.0736
0.5189	0.5398	0.4211	0.5129	0.5268
0.0868	0.0997	0.0811	0.1248	0.0721
0.1974	0.1879	0.1469	0.1948	0.1028
0.0376	0.0003	0.0317	0.0003	0.0003
0.2387	0.2046	0.1334	0.2972	0.1340
0.0252	0.0169	0.0176	0.0275	0.0253
0.0225	0.0146	0.0003	0.0003	0.0240
0.0504	0.0193	0.0228	0.0308	0.0320
0.0003	0.0003	0.0003	0.0003	0.0003
0.0003	0.0165	0.0218	0.0581	0.0003
0.0348	0.0177	0.0003	0.0003	0.0311
0.0003	0.0003	0.0003	0.0003	0.0003
0.0003	0.0003	0.0003	0.0003	0.0003
0.0003	0.0003	0.0003	0.0003	0.0106
0.0003	0.0003	0.0003	0.0003	0.0003
0.0003	0.0003	0.0003	0.0003	0.0003
0.0062	0.0003	0.0003	0.0093	0.0003
0.0003	0.0003	0.0003	0.0003	0.0003
0.0003	0.0003	0.0003	0.0003	0.0003

0.0091	0.0003	0.0003	0.0050	0.0049
0.0070	0.0003	0.0003	0.0034	0.0035
0.0003	0.0080	0.0077	0.0063	0.0055
0.0199	0.0180	0.0199	0.0215	0.0087
0.0075	0.0091	0.0003	0.0102	0.0003
0.1753	0.1924	0.1796	0.1621	0.0455
1.4878	1.5253	1.2351	1.7045	0.8113
0.0003	0.0003	0.0093	0.0058	0.0003
0.0068	0.0220	0.0224	0.0275	0.0088
0.1298	0.1659	0.0754	0.1572	0.1225
0.1529	0.2540	0.1611	0.0893	0.1424
0.0209	0.0003	0.0003	0.0198	0.0260
0.0003	0.0003	0.0003	0.0003	0.0003
0.6556	0.3851	0.3243	0.3798	0.5686
0.0003	0.0003	0.0003	0.0003	0.0003
0.0003	0.0003	0.0003	0.0003	0.0003
0.9735	1.5564	1.2598	1.1116	1.3363
0.0003	0.0003	0.0003	0.0003	0.0003
0.0385	0.0331	0.0269	0.0289	0.0388
0.0003	0.0003	0.0003	0.0003	0.0003
0.0003	0.0003	0.0003	0.0003	0.0003
0.0003	0.0003	0.0003	0.0003	0.0003
0.0003	0.0003	0.0003	0.0003	0.0003
0.0003	0.0003	0.0003	0.0003	0.0003
0.0003	0.0003	0.0003	0.0003	0.0003
0.0003	0.0003	0.0003	0.0003	0.0003
0.0003	0.0003	0.0003	0.0003	0.0003
0.0003	0.0003	0.0088	0.0119	0.0003
0.0003	0.0003	0.0003	0.0003	0.0003
0.0003	0.0003	0.0003	0.0003	0.0003
0.0003	0.0003	0.0003	0.0003	0.0003
0.0003	0.0003	0.0003	0.0003	0.0003
0.0003	0.0003	0.0003	0.0036	0.0003
0.0003	0.0003	0.0003	0.0003	0.0003

0.0003	0.0003	0.0003	0.0003	0.0194
0.0003	0.0003	0.0003	0.0003	0.0003
0.0003	0.0003	0.0003	0.0003	0.0003
0.0003	0.0003	0.0003	0.0003	0.0003
0.0003	0.0003	0.0003	0.0003	0.0003
0.0003	0.0003	0.0003	0.0003	0.0003
0.0003	0.0003	0.0003	0.0003	0.0003
0.0922	0.0985	0.1022	0.1217	0.0345

CO 23 VULV IL + AA 2 209	CO 23 VULV IL + AA 3 210	CO 23 VEST POLY IC + AA 1 211	CO 23 VEST POLY IC + AA 2 212
0.0003	0.0003	0.0003	0.0003
0.0222	0.0003	0.0003	0.0003
0.0003	0.0003	0.0003	0.0003
0.8839	0.6808	0.3035	0.2417
1.4729	1.2597	0.7910	0.6966
0.0003	0.0003	0.0003	0.0003
0.0003	0.0003	0.0003	0.0003
0.0003	0.0003	0.0003	0.0003
0.0003	0.0069	0.0003	0.0003
99.5494	107.5370	65.2767	59.3990
0.1175	0.1167	0.1260	0.1358
5.4159	5.0523	3.0688	2.7058
0.0003	0.0003	0.0003	0.0003
19.5459	19.1271	6.7603	5.9405
0.0003	0.0003	0.0003	0.0003
0.0003	0.0003	0.0003	0.0003
0.2313	0.1987	0.1026	0.1217
0.0003	0.0003	0.0003	0.0003
8.4923	8.2862	3.1110	2.6628
0.0003	0.0003	0.0003	0.0003
0.0513	0.0475	0.0412	0.0420
2.4058	2.4603	2.1940	2.4526
0.0003	0.0003	0.0003	0.0003
0.0003	0.0003	0.0003	0.0003
0.0232	0.0225	0.0003	0.0003
1.4091	1.3773	0.4406	0.3894
5.7593	4.9877	3.1265	2.9026
0.0655	0.0625	0.0468	0.0416
0.0003	0.0003	0.0003	0.0003
0.0003	0.0003	0.0003	0.0003
0.1105	0.0774	0.0003	0.0386
0.4205	0.3018	0.1457	0.1372
9.9145	7.5283	2.2986	2.1331

0.0003	0.0003	0.0003	0.0003
0.0003	0.0003	0.0003	0.0003
0.1403	0.0003	0.0746	0.1182
0.0003	0.0003	0.0003	0.0283
0.0003	0.0003	0.0003	0.0003
0.0003	0.0003	0.0003	0.0035
0.0003	0.0003	0.0003	0.0003
0.0003	0.0003	0.0003	0.0003
0.3159	0.3897	0.5302	0.5299
0.0003	0.0003	0.0003	0.0003
0.0046	0.0003	0.0003	0.0003
0.0055	0.0086	0.0245	0.0085
0.0003	0.0003	0.0003	0.0003
0.0003	0.0003	0.0003	0.0003
0.0003	0.0003	0.0003	0.0003
0.0003	0.0003	0.0003	0.0080
0.0889	0.2332	0.5479	0.4646
0.1923	0.2355	0.6561	0.6193
0.0003	0.0003	0.0141	0.0003
0.0003	0.0003	0.1264	0.1175
0.5405	0.6989	7.2939	8.0525
0.0003	0.0003	0.0003	0.0003
0.0003	0.0572	0.0451	0.0003
0.0003	0.0003	0.0003	0.0003
0.0003	0.0003	0.0003	0.0003
0.0003	0.0003	0.0003	0.0003
0.0863	0.0760	0.1213	0.0992
0.0257	0.0313	0.0436	0.0432
1.3231	1.4615	2.0941	1.7052
0.4638	0.4756	0.6695	0.6152
0.3725	0.4060	0.4124	0.4878
17.6839	19.3571	27.8531	35.0039
0.5971	0.6354	3.6338	1.9584
3.5786	4.0940	12.8139	9.7395

0.0835	0.0003	0.6112	0.0003
83.4568	84.7990	56.2343	61.7921
0.6817	0.4441	2.6947	2.7467
0.0003	0.0003	0.0003	0.0619
0.0003	0.0003	0.0003	0.0003
0.0003	0.0003	0.0003	0.0003
0.0976	0.0003	0.1634	0.2516
0.0003	0.0168	0.0003	0.0003
0.0003	0.0003	0.1526	0.1856
0.0003	0.0003	0.1065	0.0737
0.1718	0.1795	0.2740	0.3124
0.0003	0.0003	0.0227	0.0235
0.0770	0.0576	0.0858	0.1064
0.0610	0.0607	0.0540	0.0976
0.0801	0.0533	0.0891	0.1152
0.4200	0.5141	0.9826	1.0401
0.0504	0.0301	0.0003	0.1117
0.0994	0.1377	0.2465	0.2558
0.0003	0.0333	0.0481	0.0003
0.1459	0.1004	0.3153	0.2512
0.0184	0.0510	0.0231	0.0375
0.0225	0.0147	0.0003	0.0003
0.0335	0.0286	0.0287	0.0303
0.0003	0.0003	0.0003	0.0003
0.0143	0.0003	0.0003	0.0003
0.0003	0.0361	0.0003	0.0414
0.0003	0.0003	0.0003	0.0003
0.0003	0.0003	0.0003	0.0003
0.0003	0.0003	0.0003	0.0003
0.0003	0.0003	0.0003	0.0003
0.0003	0.0003	0.0003	0.0035
0.0003	0.0042	0.0003	0.0044
0.0003	0.0003	0.0003	0.0003
0.0003	0.0003	0.0003	0.0003

0.0003	0.0003	0.0003	0.0003
0.0046	0.0003	0.0054	0.0054
0.0003	0.0003	0.0053	0.0078
0.0147	0.0154	0.0351	0.0264
0.0003	0.0003	0.0003	0.0003
0.0582	0.0718	0.3278	0.2598
1.0200	1.4166	1.5491	1.5338
0.0003	0.0003	0.0003	0.0003
0.0003	0.0003	0.0192	0.0247
0.0003	0.1285	0.1250	0.1821
0.0003	0.2410	0.1556	0.0003
0.0003	0.0188	0.0003	0.0003
0.0003	0.0003	0.0003	0.0003
0.4932	0.5554	0.5335	0.5543
0.0003	0.0003	0.0003	0.0003
0.0003	0.0003	0.0003	0.0003
1.4683	1.5827	1.6615	1.5094
0.0375	0.0003	0.0003	0.0003
0.0314	0.0352	0.0362	0.0293
0.0003	0.0003	0.0037	0.0003
0.0003	0.0003	0.0003	0.0003
0.0003	0.0003	0.0003	0.0003
0.0003	0.0003	0.0003	0.0003
0.0003	0.0003	0.0003	0.0003
0.0003	0.0003	0.0003	0.0003
0.0003	0.0003	0.0003	0.0003
0.0003	0.0003	0.0003	0.0003
0.0003	0.0003	0.0058	0.0097
0.0003	0.0003	0.0003	0.0003
0.0003	0.0003	0.0003	0.0003
0.0003	0.0003	0.0003	0.0003
0.0003	0.0003	0.0003	0.0003
0.0003	0.0003	0.0003	0.0003
0.0003	0.0003	0.0003	0.0003

0.0003	0.0213	0.0003	0.0003
0.0003	0.0003	0.0003	0.0003
0.0003	0.0003	0.0003	0.0003
0.0003	0.0003	0.0003	0.0003
0.0003	0.0003	0.0003	0.0003
0.0003	0.0003	0.0003	0.0003
0.0003	0.0003	0.0003	0.0003
0.0510	0.0578	0.1262	0.1057

CO 23 VEST POLY IC + AA 3 213	CO 23 VULV POLY IC + AA 1 214	CO 23 VULV POLY IC + AA 2 215	CO 23 VULV POLY IC + AA 3 216
0.0003	0.0003	0.0003	0.0003
0.0003	0.0226	0.0003	0.0200
0.0003	0.0003	0.0003	0.0003
0.3437	2.0502	1.5783	1.9557
0.5574	1.4660	1.4016	1.0674
0.0003	0.0003	0.0003	0.0003
0.0003	0.0003	0.0003	0.0003
0.0003	0.0003	0.0003	0.0003
0.0003	0.0003	0.0003	0.0003
50.6186	124.0199	130.6424	104.3345
0.0885	0.2686	0.2432	0.2328
2.3281	6.4453	6.3240	5.9080
0.0003	0.0003	0.0003	0.0003
5.4703	18.9946	19.3302	18.3069
0.0003	0.0003	0.0003	0.0003
0.0003	0.0003	0.0003	0.0003
0.1280	0.3613	0.2726	0.3146
0.0003	0.0003	0.0003	0.0003
2.3390	9.1541	8.4981	7.8618
0.0003	0.0003	0.0003	0.0003
0.0346	0.0476	0.0544	0.0397
2.2925	2.7122	2.3903	2.4533
0.0003	0.0003	0.0003	0.0003
0.0003	0.0003	0.0003	0.0003
0.0003	0.0445	0.0404	0.0432
0.4214	1.3196	1.2583	1.2454
2.4427	7.0202	6.2651	7.0306
0.0435	0.0661	0.0470	0.0486
0.0003	0.0003	0.0003	0.0003
0.0003	0.0003	0.0003	0.0003
0.0258	0.0559	0.0851	0.0624
0.1443	0.2341	0.2252	0.1779
1.8939	10.1496	7.7763	9.4850

0.0003	0.0003	0.0003	0.0003
0.0003	0.0003	0.0003	0.0003
0.0555	0.1070	0.0514	0.0494
0.0003	0.0704	0.0709	0.0503
0.0003	0.0003	0.0003	0.0003
0.0003	0.0003	0.0003	0.0003
0.0003	0.0003	0.0003	0.0003
0.0003	0.0003	0.0003	0.0003
0.5025	0.5365	0.4799	0.5457
0.0003	0.0003	0.0003	0.0003
0.0003	0.0003	0.0003	0.0003
0.0358	0.0049	0.0003	0.0116
0.0003	0.0003	0.0003	0.0003
0.0003	0.0003	0.0003	0.0003
0.0003	0.0003	0.0003	0.0003
0.0003	0.0003	0.0003	0.0003
0.4289	0.4030	0.2510	0.3398
0.6440	0.5764	0.4704	0.5662
0.0003	0.0003	0.0096	0.0003
0.1038	0.0003	0.0003	0.1053
5.4592	0.6334	0.3339	0.5816
0.0003	0.0003	0.0003	0.0003
0.0629	0.0458	0.0312	0.0003
0.0003	0.0003	0.0003	0.0003
0.0003	0.0269	0.0003	0.0380
0.0003	0.0042	0.0003	0.0003
0.0812	0.0863	0.0811	0.0941
0.0376	0.0310	0.0361	0.0312
1.4954	1.7155	1.6003	1.8414
0.5817	0.5060	0.4707	0.5495
0.3456	0.4701	0.4679	0.5416
30.2090	59.4895	54.5804	56.9888
1.5107	0.9226	0.7183	0.9390
8.3246	14.9487	13.3536	14.6593

0.0003	0.0003	0.0003	0.0003
61.1653	94.7554	84.0696	96.0134
2.1967	2.6305	1.7775	1.6578
0.0003	0.0406	0.0456	0.0536
0.0003	0.0003	0.0003	0.0003
0.0003	0.0003	0.0003	0.0003
0.1596	0.2590	0.3308	0.3384
0.0003	0.0003	0.0003	0.0003
0.1185	0.1173	0.1032	0.1724
0.0705	0.0576	0.0003	0.0570
0.2543	0.2020	0.2098	0.2505
0.0236	0.0289	0.0240	0.0353
0.0831	0.0652	0.0544	0.0760
0.0840	0.0442	0.0504	0.0502
0.0827	0.0800	0.0003	0.0928
0.8351	1.2453	1.1048	1.3336
0.0606	0.1470	0.1014	0.0811
0.2499	0.1388	0.1953	0.2481
0.0003	0.0003	0.0003	0.0003
0.2701	0.1547	0.1728	0.1958
0.0471	0.0429	0.0257	0.0262
0.0247	0.0325	0.0174	0.0003
0.0262	0.0318	0.0314	0.0378
0.0061	0.0003	0.0003	0.0003
0.0003	0.0003	0.0003	0.0003
0.0003	0.0286	0.0003	0.0003
0.0003	0.0003	0.0003	0.0003
0.0003	0.0003	0.0003	0.0003
0.0003	0.0003	0.0003	0.0161
0.0003	0.0003	0.0003	0.0003
0.0003	0.0003	0.0024	0.0003
0.0003	0.0069	0.0084	0.0092
0.0003	0.0003	0.0003	0.0003
0.0003	0.0003	0.0003	0.0003

0.0003	0.0003	0.0003	0.0003
0.0003	0.0003	0.0034	0.0003
0.0083	0.0003	0.0003	0.0003
0.0262	0.0145	0.0108	0.0201
0.0003	0.0003	0.0003	0.0003
0.2208	0.1391	0.0830	0.1334
1.6934	1.5524	1.3084	1.5964
0.0003	0.0003	0.0003	0.0040
0.0208	0.0150	0.0122	0.0075
0.1146	0.1700	0.1088	0.0602
0.1023	0.2691	0.1973	0.1468
0.0003	0.0139	0.0003	0.0003
0.0046	0.0003	0.0003	0.0003
0.4191	0.4021	0.5094	0.4840
0.0003	0.0003	0.0003	0.0003
0.0003	0.0003	0.0003	0.0003
1.5088	1.5356	1.4750	1.6512
0.0003	0.0003	0.0003	0.0003
0.0257	0.0353	0.0456	0.0456
0.0003	0.0003	0.0003	0.0003
0.0003	0.0003	0.0003	0.0003
0.0003	0.0003	0.0003	0.0003
0.0003	0.0003	0.0003	0.0003
0.0003	0.0003	0.0003	0.0003
0.0188	0.0003	0.0229	0.0188
0.0003	0.0003	0.0003	0.0003
0.0003	0.0003	0.0003	0.0003
0.0117	0.0024	0.0047	0.0059
0.0003	0.0003	0.0003	0.0003
0.0003	0.0003	0.0003	0.0003
0.0003	0.0003	0.0003	0.0003
0.0003	0.0003	0.0003	0.0003
0.0003	0.0003	0.0003	0.0003
0.0003	0.0003	0.0003	0.0003

0.0003	0.0003	0.0183	0.0231
0.0003	0.0003	0.0003	0.0003
0.0003	0.0003	0.0003	0.0003
0.0003	0.0003	0.0003	0.0003
0.0003	0.0003	0.0003	0.0003
0.0003	0.0003	0.0003	0.0003
0.0003	0.0003	0.0003	0.0003
0.1085	0.0578	0.0456	0.0422
